# Potential biomarkers as a predictive factor of response to primary chemotherapy in breast cancer patients

**DOI:** 10.1590/1414-431X2024e13599

**Published:** 2024-10-07

**Authors:** F.O. Buono, R.D.S. Pugliese, W.A. da Silveira, D.P.C. Tirapelli, F.J.C. dos Reis, J.M. de Andrade, H.H.A. Carrara, D.G. Tiezzi

**Affiliations:** 1 Departamento de Ginecologia e Obstetrícia, Faculdade de Medicina de Ribeirão Preto, Universidade de São Paulo, Ribeirão Preto, SP, Brasil; 2 Departamento de Cirurgia e Anatomia, Faculdade de Medicina de Ribeirão Preto, Universidade de São Paulo, Ribeirão Preto, SP, Brasil; 3 Science Centre, Staffordshire University, Stoke-on-Trent, Staffordshire, England, UK; 4 Laboratório de Ciência de Dados Translacionais, Faculdade de Medicina de Ribeirão Preto, Universidade de São Paulo, Ribeirão Preto, SP, Brasil

**Keywords:** Breast cancer, microRNAs, OncomiR, Primary therapy, TCGA, Bioinformatics analysis

## Abstract

In this study, we identified miRNAs and their potential mRNA targets that are intricately linked to primary chemotherapy response in patients with invasive ductal carcinomas. A cohort of individuals diagnosed with advanced invasive breast ductal carcinoma who underwent primary chemotherapy served as the cornerstone of our study. We conducted a comparative analysis of microRNA expression among patients who either responded or did not respond to primary systemic therapy. To analyze the correlation between the expression of the whole transcriptome and the 24 differentially expressed (DE) miRNAs, we harnessed the extensive repository of The Cancer Genome Atlas (TCGA) database. We mapped molecular mechanisms associated with these miRNAs and their targets from TCGA breast carcinomas. The resultant expression profile of the 24 DE miRNAs emerged as a potent and promising predictive model, offering insights into the intricate dynamics of chemotherapy responsiveness of advanced breast tumors. The discriminative analysis based on the principal component analysis identified the most representative miRNAs across breast cancer samples (miR-210, miR-197, miR-328, miR-519a, and miR-628). Moreover, the consensus clustering generated four possible clusters of TCGA patients. Further studies should be conducted to advance these findings.

## Introduction

Despite advances in the early detection and therapeutic intervention of breast cancer, the reported prevalence of locally advanced breast cancer (LABC) and metastatic breast cancer (MBC) increased from 10.5 to 12% in developed countries (1). In developing countries such as Brazil, the prevalence of LABC and MBC is even higher and can reach up to 40% (1,2)

LABC and MBC are life-threatening conditions, and their primary treatment is based on chemotherapy (3,4). Recent studies have shown that the response to primary chemotherapy significantly affects disease-free and disease-specific survival in LABC (5). LABC patients who achieve a complete pathological response (pCR) experience a significant improvement in overall survival. The impact of pCR on survival is most remarkable in aggressive biological subtypes, such as HER2 and triple-negative types (3,4). Furthermore, neoadjuvant chemotherapy (NACT) enables surgery on inoperable tumors and increases the rates of breast-conserving surgeries (6).

Specific molecules have been studied and proposed as biological markers and prognostic predictors (7). Accordingly, miRNAs have been used as pivotal regulators of carcinogenic processes (8). miRNAs regulate several cancer pathways, promoting proliferation, invasion, and metastasis (9). Some studies have found that miRNAs could lead to resistance to chemotherapy in breast cancer (10). The association between primary chemotherapy and miRNA expression in LABC patients was investigated, and some miRNA expressions decreased in patients who achieved pCR (11). Using biomarkers to predict therapy response may improve the personalization of treatment and minimize adverse effects (12).

We aimed to identify a set of miRNAs associated with the response to primary chemotherapy in patients with LABC and MBC based on an *in silico* analysis of publicly available databases to validate our data that identified potential miRNAs and their mRNA targets involved in the primary chemotherapy response.

## Material and Methods

### Patient details

The inclusion criteria for this prospective study were patients diagnosed with invasive breast ductal carcinoma stage IIb-IV (13) who underwent primary chemotherapy. Tumor samples were collected before any treatment from 33 patients through ultrasound-guided core needle biopsy at Hospital das Clínicas - Ribeirão Preto Medical School (HCRP-USP). The institution's ethics committee approved the study under guideline 2467/09. Free and informed consent was obtained before the collection of samples.

### RNA extraction

Total RNA was extracted from samples using phosphate-buffered saline (PBS) and Trizol^®^ (Invitrogen, USA). RNA was precipitated with 100% isopropyl alcohol. The MirVana RNA (ThermoFisher Scientific, USA) extraction protocol was used for purification. The RNAs were purified in one fraction without separation to enrich small RNAs.

### cDNA synthesis

Total RNA was subjected to reverse transcription using the commercial TaqMan microRNA Reverse Transcription Kit (Applied Biosystems, USA) and a set of primer oligonucleotides specific to human microRNAs. Following the manufacturer's instructions, reverse transcription was conducted using the Megaplex^TM^ RT Primers kit, Human Pool (Applied Biosystems). A pre-amplification reaction was conducted for each sample using Megaplex^TM^ PreAmp Primers, Human Pool A (Applied Biosystems).

### microRNA quantification

To quantify microRNA expression, quantitative polymerase chain reaction (PCRq) was conducted using TaqMan^®^ Low Density Arrays (TLDA). We used the TaqMan^®^ Array Human MicroRNA Cards Set v3.0 kit (Applied Biosystems) with TaqMan^®^ Universal PCR Master Mix and NoAmpErase^®^ 2X. The Kit TaqMan^®^ Array Human MicroRNA Cards Set v3.0 is an assay used to analyze the expression of 384 mature microRNAs. We used the miRNAs U6 snRNA-001973, RNU44-001094, and RNU48-001006 as housekeeping miRNAs to estimate delta-delta Ct (ddCt), and submitted the expression matrix for quantile normalization.

### Chemotherapy protocol

All patients underwent the treatment scheme proposed by the protocol of the Breast Disease and Gynecological Oncology Sector of HCRP-USP. Patients in stages II and III (n=33) received NACT with epirubicin cyclophosphamide-docetaxel (EC-T), four cycles of epirubicin 75 mg/m^2^ and cyclophosphamide (600 mg/m^2^) in intravenous D1 infusions every 21 days, followed by four cycles of docetaxel (100 mg/m^2^). Patients with HER2+ tumors received trastuzumab in association with docetaxel (EC-TH).

### Bioinformatics analysis

We conducted comparative expression analysis in patients that achieved pCR, defined as the absence of residual invasive neoplasia at the primary site and axilla, and patients with residual disease (RD) after primary chemotherapy. The HTqPCR library (14) was used for normalization, and the miRNAs U6 snRNA-001973, RNU44-001094, and RNU48-001006 were used as housekeeping miRNAs to estimate ddCt. The *limma* library (15) for the differential expression model was adjusted by estrogen receptor (ER) and human epidermal growth factor receptor 2 (HER2) expression. We considered differentially expressed (DE) miRNAs (DEMI) with a fold change >1 and a P-value adjusted for multiple testing using the false discovery ratio (FDR) of less than 0.05. Statistical analyses and visualizations were conducted using R software (R Core Team, 2021).

### *In silico* analyses

We used publicly available data from The Cancer Genome Atlas (TCGA) for comprehensive molecular analyses (https://portal.gdc.cancer.gov/), the normalized data from patients (n=1061) with breast cancer of global expression of genes (RNAseq) from Broad Firehose Genome Data Analysis Center (GDAC) (https://gdac.broadinstitute.org/), and miRNAs (miRNAseq) from the TCGA repository (https://portal.gdc.cancer.gov/) for correlation analyses between miRNA and mRNA expression. A significant anti-correlation (Pearson) was considered for r<−0.3 and adjusted P-value (FDR) <0.05.

The clinical and pathological data were downloaded from cBioPortal (https://www.cbioportal.org/). We reclassified the ER status as positive or negative based on the immunohistochemistry (IHC) and the ER alpha expression from the Reverse Phase Protein Analysis (RPPA) data downloaded from the Broad Firehose GDAC repository. The ROC curve discriminated the tumor ER status with an area under the curve (AUC)=0.95. The best cut-off point to discriminate between ER-negative and -positive tumors was an ER alpha expression below -1.1 in the RPPA data.

We used multiMiR tools (16) to predict and validate miRNA-mRNA interactions proposed by the previous correlation analysis. This tool uses a series of databases (miRecords, miRTarbase, TarBase, Diana tools, EIMMo, MicroCosm, miRanda, miRDB, PicTar, PITA, and TargetScan) for statistical inference. In the over-representation analysis, we used hallmark gene sets to identify the genes associated with the process leading to breast cancer. This was conducted using the R packages ReactomePA (17) to map molecular mechanisms associated with these miRNAs. The analyses were conducted at a significance level of 5% (q value <0.05).

### miRNAs analyses

For miRNA expression analyses, we normalized the data from TCGA using Log10. We compared the 24 miRNAs expressions between normal mammary tissue and paired tumor samples (n=103). We used the Shapiro-Wilk test to evaluate the normal distribution of the data. The majority of miRNAs had a normal distribution. We used the diptest function in R to test these miRNAs with non-normality to evaluate whether the data distribution was unimodal or bimodal. The analyses were conducted at a significance level of 5%. We employed consensus clustering (18), a method that provides quantitative evidence for determining the number and patients of possible clusters. Clinical data of TCGA breast cancer patients were obtained from cBioPortal for Cancer Genomics (https://www.cbioportal.org/). We obtained the histological grades from Heng et al. (19). The pathological stage data were converted to the first edition from American Joint Committee on Cancer (AJCC) (13) for standardization. We considered patients as HER2-positive if they were IHC 3+ or IHC 2+ and positive *in situ* hybridization (ISH). We also used data of breast cancer patients from the RPPA and Mutation Annotation Format (MAF) from the TCGA.

The analysis of the association between miRNA expression and clinical features was conducted using the parametric Student's *t*-test and non-parametric Wilcoxon test for binary variables. Analysis of variance (ANOVA) or the Kruskal-Wallis test was used for variables with more than two groups. We also applied Fisher's exact test to determine the dispersion value for two nominal categorical variables. The analyses were performed at the 5% significance level. We used principal component analysis (PCA) to obtain the most representative miRNA based on linear combinations of variables with the prcomp function. The ConsensusClusterPlus and Dunn Index (clValid) (20) were used to analyze and validate cluster samples.

## Results

### Patient characteristics

A total of 33 patients were included, and the mean age was 52.6±6 years. The most frequent clinical stage was III (n=22); six patients were in stage II and five patients were in stage IV. According to the pathological features, most tumors were classified as grade 2 (n=21), five were classified as grade 1, and seven as grade 3. Based on IHC analysis, 20 tumors were considered positive for ER, 18 for progesterone receptor (PR), and 15 for HER2. A total of 10 patients experienced pCR (30%).

### miRNAs analyses

**Figure 1:**
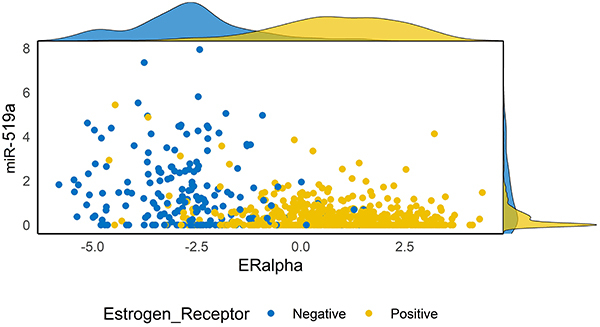
Scatter plot with a marginal density map with estrogen receptor (ER) expression associated with miR-519a expression (P<0.0001) in the y-axis and ERalpha expression (P=0.0251) in the x-axis. miR-519a and ERalpha expression are inversely correlated (rho=-0.4, P<0.001). The Cancer Genome Atlas (TCGA) cohort (n=1061).

Using differential expression analysis of 384 miRNAs, we compared patients with pathologic complete response (pCR) to those with residual disease (RD). We identified 24 DE miRNAs: let-7a, let-7c, miR-23a, miR-23b, miR-29c, miR-107, miR-125a-3p, miR-125a-5p, miR-127, miR-132, miR-182, miR- 193a, miR-197, miR-210, miR-221, miR-328, miR-330, miR-495, miR-512, miR-517a, miR-517c, miR-519a, miR-532, and miR-628. To validate these DE miRNAs, we used the publicly available data from TCGA. We compared the miRNA expression in paired samples between tumor tissue and adjacent normal mammary glands. For miRNA expression analyses, we verified that the miRNAs miR-107 (P.adj<0.0001), miR-182 (P.adj<0.0001), miR-210 (P.adj<0.0001), miR-330 (P.adj<0.0001), and miR-628 (P.adj<0.0001) were overexpressed in tumor tissue, whereas let-7a (P.adj=0.04), let-7c (P.adj<0.0001), miR-127 (P.adj<0.0001), miR-193a (P.adj<0.0001), miR- 221 (P.adj <0.0001), miR-328 (P.adj<0.0001), miR-495 (P.adj<0.0001), and miR-517a (P.adj=0.04) were underexpressed. The expression of miR-23a, miR-23b, miR-29c, miR-125a-3p, miR-125a-5p, miR-132, miR-197, miR-512, miR-517c, miR-532, and miR-519a were not associated with the malignant phenotype. However, we have identified that miR-519a had a bimodal distribution (P<0.0001), and its expression was highly associated with ER expression. In Figure 1, the miR-519a and ERα expressions were inversely proportional, making it specific to a group of ER-negative tumors.

### Correlation between miRNAs-RNAm

**Figure 2:**
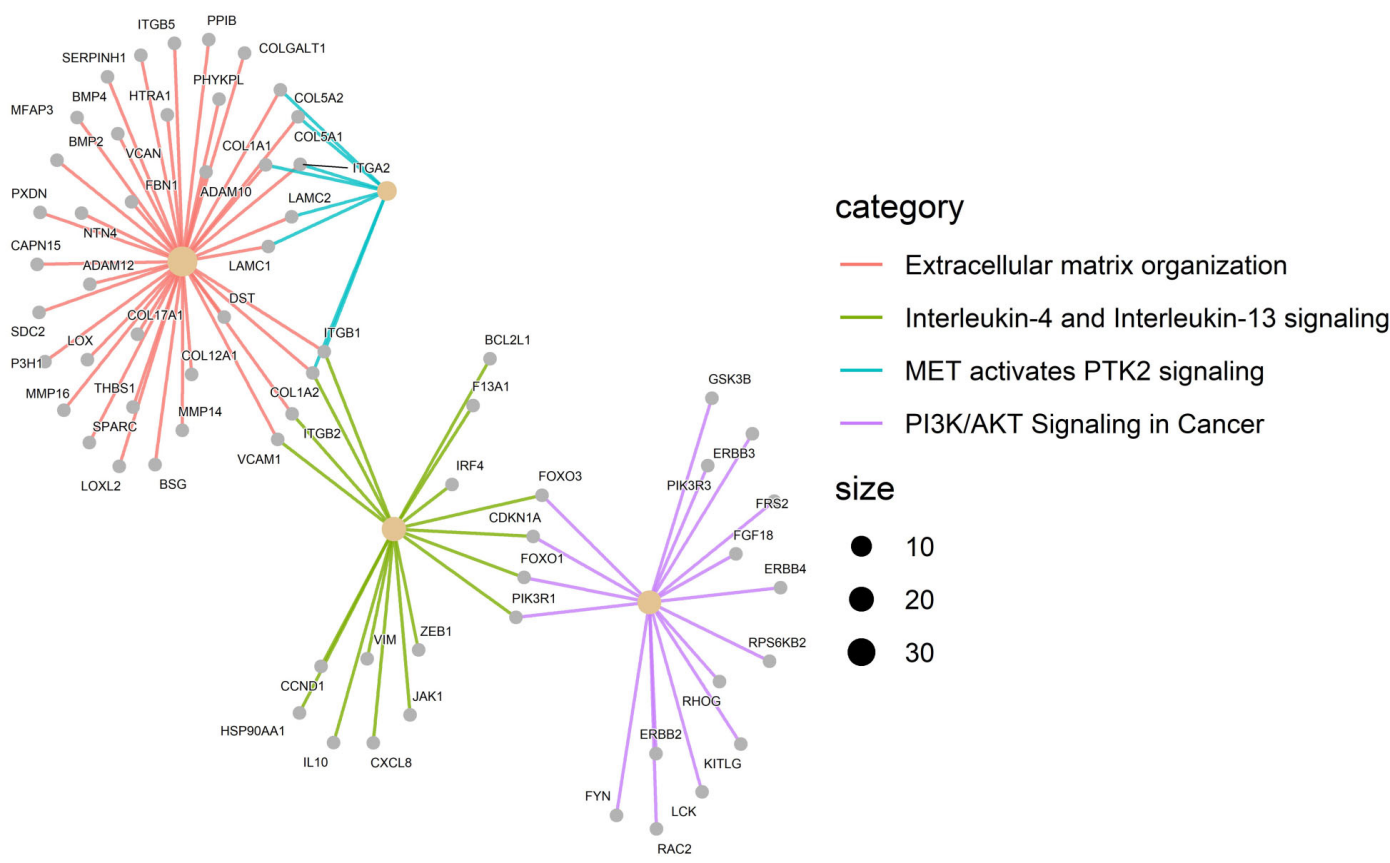
Signaling pathways of genes related to differentially expressed (DE) miRNAs in estrogen receptor (ER)-negative breast cancer samples (TCGA, n=240), using the Reactome database (R).

**Figure 3:**
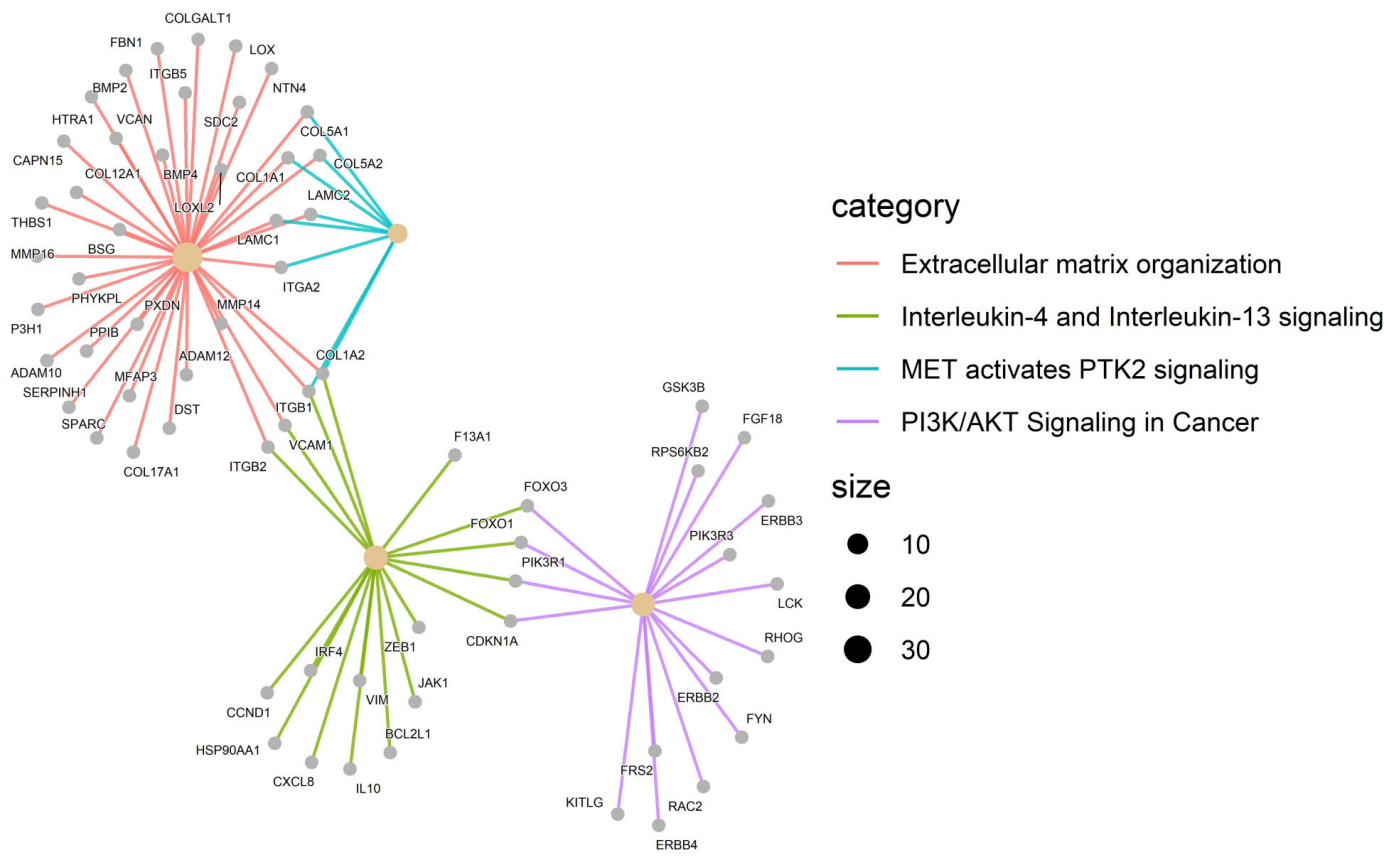
Signaling pathways of genes related to differentially expressed (DE) miRNAs in estrogen receptor (ER)-positive breast cancer samples (TCGA, n=772), using the database of Reactome (R).

We selected the DE miRNAs in tumors and compared them with the matched normal mammary gland samples for mRNA target prediction. The TCGA mRNA and miRNA database was used, and anti-correlation was conducted independently in ER-negative and -positive tumors. Accordingly, we analyzed the data on the multiMiR tool to predict integration among miRNA and mRNA (Supplementary Tables S1 and S2). Furthermore, we mapped the molecular mechanisms associated with these miRNAs and their targets. The reactome database revealed four significant signaling pathways associated with cancer among ER-negative and ER-positive samples, extracellular matrix organization (q value=0.04), interleukin-4, and interleukin-13 signaling (q value=0.06), PI3K/AKT signaling in cancer (q value=0.07), and MET-activated PTK2 signaling (q value=0.07) (Figures 2 and 3). The signaling pathways determined for the ER-positive samples were PIP3 activates AKT signaling (q value=0.002) and signaling by neurotrophic tyrosine receptor kinases (NTRKs) (q value=0.09) (Figure 3 and Supplementary Tables S3 and S4). ER-positive and ER-negative samples share 342 genes in their pathways. For example, FOXO1 is regulated by miR-107 and miR-210, FOXO3 is regulated by miR-221, SOX11 is regulated by let-7a, PIK3R1 is regulated by miR-107 and miR-221, RUNX1 is regulated by mioR-107, ETS1 is regulated by miR-182, and COL1A2 is regulated by miR-197 and miR-532.

### Discriminative analysis

**Figure 4:**
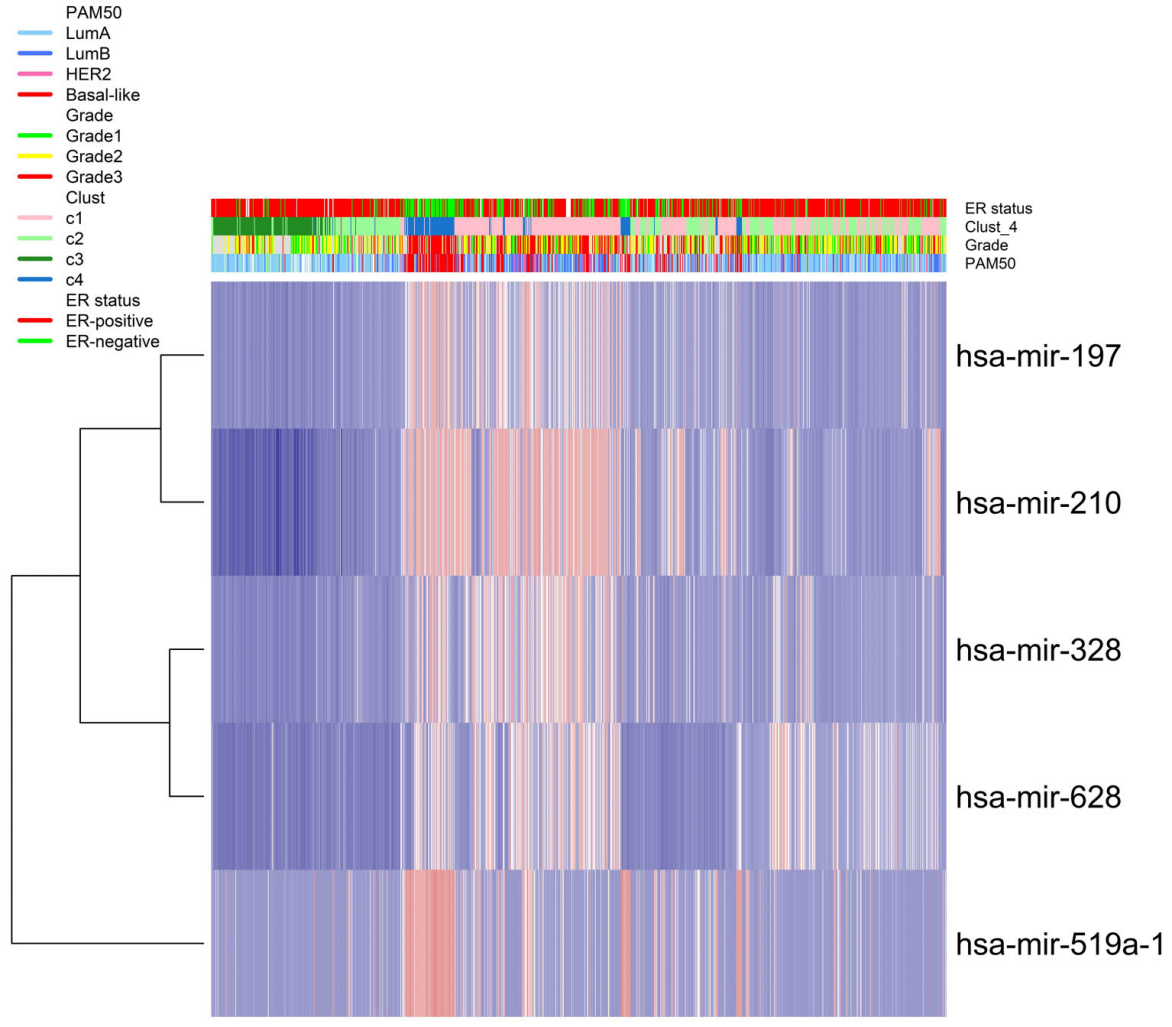
Hierarchical clustering of four possible clusters by comparing clinical features of estrogen receptor (ER)-negative breast cancer patients from TCGA with the most representative differentially expressed (DE) miRNAs.

A comprehensive biological investigation of 14 DE miRNAs (let-7a, let-7c, miR-107, miR-127, miR-182, miR-193a, miR-210, miR-221, miR-330, miR-328, miR-495, miR-517a, miR-519a, and miR-628) was conducted by integrating clinical, pathological, and molecular TCGA data. The discriminative analysis based on PCA identified the most representative miRNAs across breast cancer samples (miR-210, miR-197, miR-328, miR-519a, and miR-628). Moreover, the consensus clustering generated four possible miR clusters from the TCGA samples (Figure 4).

In Figure 4, we found that the c1 cluster is related to the overexpression of miR-210. This cluster is not associated with a subtype. The overexpression of miR-519a is associated with the c4 cluster, which is related to the basal-like subtype (P-value <0.0001). We also found that the bimodal distribution of the miR-519a was associated with the ER status (P-value <0.0001). Likewise, clusters 2 and 3 were associated with ER-positive samples, related to the luminal A subtype (P-value <0.0001).

**Figure 5:**
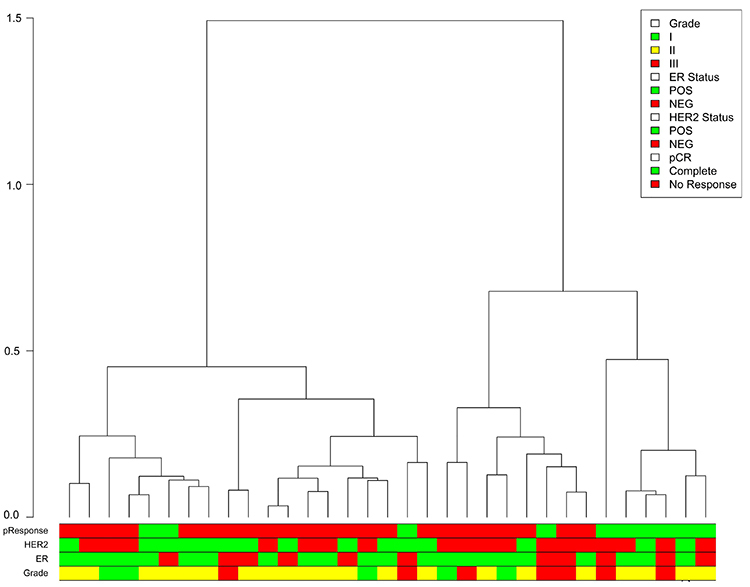
Dendrogram of the samples divided into two clusters according to the most representative miRNAs among breast carcinoma patients at the Hospital das Clínicas (HCRP-USP) in Ribeirão Preto (n=33). The metric for evaluating the clustering algorithms was the Dunn index (DI=1.07).

We conducted hierarchical clustering analysis on 33 breast cancer samples from HCRP using the expression of the 5 DE miRNAs, by separating the tumor samples into two large clusters with distinct complete pathological response rates (P=0.02). Applying the hierarchical clustering to the HCRP dataset based on the selected miRs generated two distinct clusters with significant clinical implications (DI=1.07) (Figure 5).

## Discussion

We identified 24 DE miRNAs in breast carcinomas by comparing patients who responded or not to primary chemotherapy. Integrative analysis of the TCGA database confirmed 13 DE miRNAs in carcinoma samples compared to matched normal mammary glands. Notably, miR-519a was specifically expressed in tumor tissue and associated with ER-negative status, indicating its relevance to the malignant phenotype and potential involvement in chemotherapy resistance. Further analysis of target gene expression in ER-positive and ER-negative samples from the TCGA dataset suggested that these miRNAs may regulate the PI3K/AKT signaling pathway in breast cancer. Gene expression profiles in both ER-negative and ER-positive samples were associated with extracellular matrix organization, interleukin-4, and interleukin-13 signaling, and MET-activated PTK2 signaling. Additionally, in ER-positive samples, enrichment of PIP3-activated AKT signaling and NTRK pathway signaling was observed. Discriminative analysis highlighted miR-519a, miR-210, miR-197, miR-628, and miR-328 as the most representative miRNAs in breast cancer. Notably, miR-519a expression was significantly higher in ER-negative breast cancer compared to ER-positive breast cancer.

Although the expression of miR-519a in cancer has been poorly studied, some reports suggest that it is associated with apoptosis resistance, tumorigenesis, cellular viability, and cell cycle progression (21,22). Its expression can regulate apoptosis in breast cancer cells as this miRNA leads to resistance to programmed cell death and evasion from the immune response (21). Additionally, miR-519a is a direct regulator of the phosphatase and tensin homolog (*PTEN*) gene. The high expression of this gene can inhibit cell viability, proliferation, and colony formation, and promote G0/G1 cell cycle phases (22). *PTEN* also negatively regulates the PI3/AKT/mTOR pathway, which controls the cancer cell growth factor response for metastasis and chemotherapy resistance (23). Tu et al. (22) demonstrated that the overexpression of miR-519a increases AKT functionality. increasing drug resistance, tumorigenesis, and metastasis (23). Moreover, Breunig et al. (21) revealed that the overexpression of miR-519a in breast carcinoma is associated with mutated *TP53* gene. They also found that ER-negative breast cancer is strongly associated with *TP53* mutations. In our study with HCRP samples, we found a low expression of this miRNA and a non-association with estrogen receptors, possibly owing to the small number of samples.

miR-210 has been implicated with the cell cycle, cell survival, mitochondrial metabolism, DNA damage repair, immune response, angiogenesis, anti-apoptotic process, and hypoxia by acting as target hypoxia-inducible factor 1α (HIF-1α) (24). The overexpression of this miRNA was studied in numerous types of cancer, including glioblastoma, colorectal, bone, lung, prostate, ovarian, and breast cancer (25,26). In breast cancer, miR-210 has been associated with poor prognosis, which could lead to invasion, early metastasis, recurrence, shorter survival, and tamoxifen resistance, characteristics commonly found in aggressive phenotypes. In several studies, this miRNA has been associated with basal-like or triple-negative subtypes (26). In our study, miR-210 was associated with chemotherapy resistance. Evangelista et al. (25) demonstrated that the *RUNX* Family Transcription Factor 3 *(RUNX3*) targets miR-210. Our study identified the gene *RUNX1* Partner Transcriptional Co-Repressor 1 (*RUX1T1*) as a target of miR-210. This family is associated with cell invasion, migration, and proliferation. *RUNX3* was demonstrated to be a tumor suppressor and associated with ER signaling in breast cancer.

Another miR-210 target is Homeobox A1 (*HOXA*), which regulates ER and plays a role in tamoxifen resistance. ER binds to lysine (K)-specific demethylase 3 (KDM3A). This complex indirectly regulates the HOXA1 transcriptional locus, leading to higher ER activation in the presence of tamoxifen. Our study indicated that HOXA3 and HOXA9 are miR-210 targets. ER-positive breast cancer patients treated with tamoxifen have a poor prognosis and higher recurrence risk associated with the overexpression of miR-210 (27). Egeland et al. (27) demonstrated that miR-519a and miR-210 are indirectly related to estrogen receptor gene (*ESR1*) expression. In their network analysis, they observed that tamoxifen resistance is not always associated with a mutation.

Some studies have demonstrated that miR-197, alone or in conjunction with other miRNAs, has an essential role in different tumors (28). In non-small-cell lung cancer, this miRNA was identified as a great candidate for therapeutic targets; it regulates the p53 pathway, inhibiting apoptosis, and favors uncontrolled cell proliferation. This miRNA modulates the expression of *NOXA* and *BMF* (29). In hepatocellular carcinoma, miR-197 is overexpressed and related to cell migration, invasion, and metastasis. In thyroid cancer, this miRNA is associated with carcinogenesis. Furthermore, in pancreatic cancer, miR-197 promotes invasion, cell migration, metastasis, and epithelial-mesenchymal transition (28,30). The molecular role of miR-197 in breast cancer has not been studied extensively. However, some trials suggest that this miRNA can lead to chemoresistance in triple-negative breast cancer (31). In our study, miR-197 was associated with chemotherapy resistance.

miR-628 is known to be a potential biomarker in different types of cancer, such as cervical carcinoma (32), prostate cancer (33), and breast cancer (34). In prostate cancer, the underexpression of this miRNA can lead to proliferation and invasion of tumor cells; in the same study, the expression of miR-628 was inversely proportional to that of the protein FGFR2, and was responsible for cell proliferation, migration, and invasion (33). Moreover, miR-628 was studied in the triple-negative cell line, and its overexpression reduces cell proliferation and migration and its inhibition promotes cell metastasis. *Smad3*, a growth factor, was studied as a target of this miRNA, and its expression is inversely proportional to that of the latter (34). In our study, miR-628 was associated with *Smad6.*

miR-328 is known as a tumor suppressor in several cancers. In hepatocellular carcinoma, the overexpression of this miRNA inhibited cell proliferation and invasion, leading to apoptosis by targeting AKT. In colorectal cancer and urothelial cancer, miR-328 inhibits metastasis and cell proliferation by negatively modulating phosphorylation in the PI3K/AKT pathway (35,36). However, in ovarian cancer and head and neck squamous cell carcinoma, this miRNA promotes cell proliferation, migration, and invasion (35). In breast cancer, miR-328 and miR-519d were studied together, and both miRNAs have low expression levels and suppress cancer function by targeting Ki-67 and hindering the cell cycle and proliferation (37). Another study in breast cancer showed that miR-328 is overexpressed in the triple-negative subtype. Shi et al. (36) demonstrated that this miRNA binds to *PTEN* through base complementarity and consequently acts as a tumor suppressor in breast cancer. In our study, miR-328 was overexpressed in patients that achieved pCR.

Basal-like and triple-negative cancers are aggressive subtypes of breast cancer. IHC and ISH can be used to diagnose triple-negative breast cancer, and basal-like breast cancer is diagnosed by microarray and next-generation sequencing (NGS). Thus, some divergence can be observed between them, as 77% of basal-like tumors are triple-negative tumors. The majority of basal-like tumors express the *EGRF* gene. The expression of this gene leads to less responsiveness to chemotherapy and overall survival (OS). Moreover, triple-negative breast cancer has higher PI3K, mTOR activation, and AKT phosphorylation levels than the other subtypes. In triple-negative breast cancer, low expression of *PTEN* is correlated with an activation of the PI3K/AKT pathway (38). The PI3K/AKT pathway promotes the activation of estrogen-independent ER transcriptional activity. Conversely, ER promotes the transcription of insulin growth factor receptor (IGF-1R), which activates PI3K. ER levels are inversely proportional to PI3K in ER-positive breast cancer samples. Thus, the lower the *PTEN* expression, the lower the ER level, which can lead to poorer outcomes. The activation of the PI3K/AKT pathway can lead to endocrine treatment resistance due to the promotion of lower ER levels (39). Accordingly, in our study, we noticed the association of the basal-like subtype and negative ER status with the overexpression of miR-519a.

Considerable research has been conducted to improve the treatment of breast cancer. Efforts have been made to develop drugs targeting the PI3K/AKT signaling pathway. Most of these studies are in the clinical trial phase. GDC-0980 (apitolisib, RG7422) inhibits PI3K and mTOR. Preclinical studies have indicated the efficacy of this drug in numerous solid tumors. GDC-0032 (taselisib) is a selective inhibitor of p110α, p110δ, and p110γ isoforms of class IA PI3K. One study demonstrated that taselisib with fulvestrant was tolerated in HR-positive and HER2-negative advanced and recurrent breast cancer. The PR patients had mutated PI3KCA; in that way, this drug was expected to be effective (40). Our study inferred that these drugs can benefit some ER-negative breast tumor patients with miR-519a overexpression once this biomarker is closely associated with the PI3K/AKT pathway.

The study limitation is the few patients that were available in the HCRP and were considered a convenience sample, which do not represent the heterogeneity of breast cancer. However, the expression analysis was conducted using the TLDA methodology, which is highly reliable. Additionally, we extrapolated the findings to the TCGA cohort of breast cancer to search for a biological meaning to visualize the association between 24 DE miRNAs and genes, biomarkers, and signaling pathways. According to TCGA guidelines, this database and dataset have no current limitations.

### Conclusion

We found that complete pathological response to primary chemotherapy is associated with different tumor miRNA expression patterns. The expression profile of 24 DE miRNAs is a potential predictive model of response to chemotherapy in advanced breast tumors. A detailed study of the mechanism of action and integration may provide relevant information regarding the resistance to chemotherapy in breast cancer. *In silico* analysis of many breast cancer samples helped us identify miR-519a, miR-210, miR-197, miR-628, and miR-328 as potential targets for clinical investigation. Further studies should be conducted to elucidate these findings.

## Data Availability

The data used for this study are available upon request. The codes used are available at https://github.com/lab-tds/brca_miR.

## Supplementary Material

Click to view [zip].
