# Supplementary material for: Potential biomarkers as a predictive factor of response to primary chemotherapy in breast cancer patients
Source: Braz J Med Biol Res. 2024 Oct 7;57:e13599. doi: 10.1590/1414-431X2024e13599 (PMC11463908; doi:10.1590/1414-431X2024e13599)
Supplement: Supplementary file 1 [file 1414-431X-bjmbr-57-e13599-suppl.zip › 13559_Supplementary Table S1.docx]

**Supplementary Table S1.** Prediction of MicroRNA-Target Interactions in Breast Cancer Estrogen Receptor-Negative Patients Using the multiMiR R Package and Database.

| **GeneId** | **rho** | **P_value** | **database** | **mature_mirna** | **target_entrez** | **target_ensembl** | **experiment** | **support_type** | **pubmed_id** | **type** |
| --- | --- | --- | --- | --- | --- | --- | --- | --- | --- | --- |
| CKAP4 | -0.281 | 1.1E-05 | tarbase | MIMAT0000062 | 10970 | ENSG00000136026 | Degradome sequencing | positive |  | validated |
| GLRX3 | -0.264 | 3.7E-05 | tarbase | MIMAT0000062 | 10539 | ENSG00000108010 | Degradome sequencing | positive |  | validated |
| MYBL2 | -0.254 | 7.1E-05 | tarbase | MIMAT0000062 | 4605 | ENSG00000101057 | Degradome sequencing | positive |  | validated |
| RPS6KB2 | -0.286 | 7.6E-06 | tarbase | MIMAT0000062 | 6199 | ENSG00000175634 | Degradome sequencing//Degradome sequencing | positive |  | validated |
| SLC5A6 | -0.265 | 3.4E-05 | mirtarbase | MIMAT0000062 | 8884 | ENSG00000138074 | PAR-CLIP | Functional MTI (Weak) | 24398324 | validated |
| SLC5A6 | -0.265 | 3.4E-05 | mirtarbase | MIMAT0000062 | 8884 | ENSG00000138074 | PAR-CLIP | Functional MTI (Weak) | 21572407 | validated |
| SLC5A6 | -0.265 | 3.4E-05 | tarbase | MIMAT0000062 | 8884 | ENSG00000138074 | Degradome sequencing//Degradome sequencing//Degradome sequencing//Degradome sequencing//Degradome sequencing//Degradome sequencing//Degradome sequencing//Degrad | positive |  | validated |
| SOX11 | -0.325 | 3.2E-07 | tarbase | MIMAT0000062 | 6664 | ENSG00000176887 | Degradome sequencing | positive |  | validated |
| BLM | -0.276 | 1.5E-05 | tarbase | MIMAT0000064 | 641 | ENSG00000197299 | Degradome sequencing | positive |  | validated |
| BRD2 | -0.252 | 8.2E-05 | mirtarbase | MIMAT0000064 | 6046 | ENSG00000230678 | CLASH | Functional MTI (Weak) | 23622248 | validated |
| BRD2 | -0.252 | 8.2E-05 | mirtarbase | MIMAT0000064 | 6046 | ENSG00000234507 | CLASH | Functional MTI (Weak) | 23622248 | validated |
| BRD2 | -0.252 | 8.2E-05 | mirtarbase | MIMAT0000064 | 6046 | ENSG00000235307 | CLASH | Functional MTI (Weak) | 23622248 | validated |
| BRD2 | -0.252 | 8.2E-05 | mirtarbase | MIMAT0000064 | 6046 | ENSG00000236227 | CLASH | Functional MTI (Weak) | 23622248 | validated |
| BRD2 | -0.252 | 8.2E-05 | mirtarbase | MIMAT0000064 | 6046 | ENSG00000215077 | CLASH | Functional MTI (Weak) | 23622248 | validated |
| BRD2 | -0.252 | 8.2E-05 | mirtarbase | MIMAT0000064 | 6046 | ENSG00000234704 | CLASH | Functional MTI (Weak) | 23622248 | validated |
| BRD2 | -0.252 | 8.2E-05 | mirtarbase | MIMAT0000064 | 6046 | ENSG00000204256 | CLASH | Functional MTI (Weak) | 23622248 | validated |
| BRI3BP | -0.283 | 9.5E-06 | mirtarbase | MIMAT0000064 | 140707 | ENSG00000184992 | HITS-CLIP | Functional MTI (Weak) | 23706177 | validated |
| CCNB2 | -0.323 | 3.8E-07 | mirtarbase | MIMAT0000064 | 9133 | ENSG00000157456 | CLASH | Functional MTI (Weak) | 23622248 | validated |
| CCNB2 | -0.323 | 3.8E-07 | tarbase | MIMAT0000064 | 9133 | ENSG00000157456 | Degradome sequencing | positive |  | validated |
| CCNE2 | -0.279 | 1.2E-05 | tarbase | MIMAT0000064 | 9134 | ENSG00000175305 | Degradome sequencing//Degradome sequencing | positive |  | validated |
| CCNF | -0.269 | 2.6E-05 | mirtarbase | MIMAT0000064 | 899 | ENSG00000162063 | CLASH | Functional MTI (Weak) | 23622248 | validated |
| CCNF | -0.269 | 2.6E-05 | tarbase | MIMAT0000064 | 899 | ENSG00000162063 | Degradome sequencing//Degradome sequencing | positive |  | validated |
| CELSR3 | -0.260 | 4.7E-05 | tarbase | MIMAT0000064 | 1951 | ENSG00000008300 | Degradome sequencing | positive |  | validated |
| CENPA | -0.251 | 9.1E-05 | tarbase | MIMAT0000064 | 1058 | ENSG00000115163 | Degradome sequencing | positive |  | validated |
| CENPQ | -0.277 | 1.4E-05 | tarbase | MIMAT0000064 | 55166 | ENSG00000031691 | Degradome sequencing | positive |  | validated |
| CKAP2L | -0.350 | 3.2E-08 | tarbase | MIMAT0000064 | 150468 | ENSG00000169607 | Degradome sequencing | positive |  | validated |
| CXCL8 | -0.264 | 3.6E-05 | mirtarbase | MIMAT0000064 | 3576 | ENSG00000169429 | PAR-CLIP | Functional MTI (Weak) | 26701625 | validated |
| DDX18 | -0.263 | 4.0E-05 | mirtarbase | MIMAT0000064 | 8886 | ENSG00000088205 | CLASH | Functional MTI (Weak) | 23622248 | validated |
| DHX9 | -0.256 | 6.3E-05 | mirtarbase | MIMAT0000064 | 1660 | ENSG00000135829 | CLASH | Functional MTI (Weak) | 23622248 | validated |
| DNMT1 | -0.295 | 3.6E-06 | mirtarbase | MIMAT0000064 | 1786 | ENSG00000130816 | 3'LIFE | Functional MTI (Weak) | 25074381 | validated |
| DNMT1 | -0.295 | 3.6E-06 | tarbase | MIMAT0000064 | 1786 | ENSG00000130816 | Degradome sequencing | positive |  | validated |
| ESPL1 | -0.251 | 9.1E-05 | mirtarbase | MIMAT0000064 | 9700 | ENSG00000135476 | PAR-CLIP | Functional MTI (Weak) | 23446348 | validated |
| ESPL1 | -0.251 | 9.1E-05 | mirtarbase | MIMAT0000064 | 9700 | ENSG00000135476 | PAR-CLIP | Functional MTI (Weak) | 21572407 | validated |
| ESPL1 | -0.251 | 9.1E-05 | tarbase | MIMAT0000064 | 9700 | ENSG00000135476 | Degradome sequencing//Degradome sequencing | positive |  | validated |
| FANCI | -0.329 | 2.2E-07 | mirtarbase | MIMAT0000064 | 55215 | ENSG00000140525 | CLASH | Functional MTI (Weak) | 23622248 | validated |
| FBXO22 | -0.281 | 1.1E-05 | tarbase | MIMAT0000064 | 26263 | ENSG00000167196 | Degradome sequencing//Degradome sequencing//Degradome sequencing//Degradome sequencing//Degradome sequencing | positive |  | validated |
| GFM1 | -0.291 | 5.1E-06 | tarbase | MIMAT0000064 | 85476 | ENSG00000168827 | Degradome sequencing | positive |  | validated |
| GMNN | -0.250 | 9.5E-05 | tarbase | MIMAT0000064 | 51053 | ENSG00000112312 | Degradome sequencing//Degradome sequencing | positive |  | validated |
| HELLS | -0.252 | 8.2E-05 | tarbase | MIMAT0000064 | 3070 | ENSG00000119969 | Degradome sequencing//Degradome sequencing | positive |  | validated |
| HMMR | -0.269 | 2.6E-05 | tarbase | MIMAT0000064 | 3161 | ENSG00000072571 | Degradome sequencing | positive |  | validated |
| HSP90AA1 | -0.265 | 3.5E-05 | tarbase | MIMAT0000064 | 3320 | ENSG00000080824 | Degradome sequencing | positive |  | validated |
| IARS1 | -0.258 | 5.4E-05 | tarbase | MIMAT0000064 | 3376 | ENSG00000196305 | Degradome sequencing | positive |  | validated |
| IMMT | -0.274 | 1.9E-05 | tarbase | MIMAT0000064 | 10989 | ENSG00000132305 | Degradome sequencing | positive |  | validated |
| KIF18B | -0.318 | 5.7E-07 | tarbase | MIMAT0000064 | 146909 | ENSG00000186185 | Degradome sequencing | positive |  | validated |
| KPNA2 | -0.293 | 4.4E-06 | tarbase | MIMAT0000064 | 3838 | ENSG00000182481 | Degradome sequencing//Degradome sequencing//Degradome sequencing//Degradome sequencing | positive |  | validated |
| MAL2 | -0.318 | 5.4E-07 | tarbase | MIMAT0000064 | 114569 | ENSG00000147676 | Degradome sequencing | positive |  | validated |
| MKI67 | -0.250 | 9.3E-05 | tarbase | MIMAT0000064 | 4288 | ENSG00000148773 | Degradome sequencing//Degradome sequencing | positive |  | validated |
| MSH6 | -0.265 | 3.3E-05 | tarbase | MIMAT0000064 | 2956 | ENSG00000116062 | Degradome sequencing//Degradome sequencing//Degradome sequencing//Degradome sequencing//Degradome sequencing | positive |  | validated |
| NBEAL2 | -0.268 | 2.8E-05 | tarbase | MIMAT0000064 | 23218 | ENSG00000160796 | Degradome sequencing//Degradome sequencing//Degradome sequencing//Degradome sequencing//Degradome sequencing//Degradome sequencing | positive |  | validated |
| PCNA | -0.293 | 4.1E-06 | tarbase | MIMAT0000064 | 5111 | ENSG00000132646 | Degradome sequencing | positive |  | validated |
| PHF19 | -0.252 | 8.5E-05 | tarbase | MIMAT0000064 | 26147 | ENSG00000119403 | Degradome sequencing//Degradome sequencing//Degradome sequencing//Degradome sequencing | positive |  | validated |
| RAB3IP | -0.320 | 4.7E-07 | tarbase | MIMAT0000064 | 117177 | ENSG00000127328 | Degradome sequencing//Degradome sequencing | positive |  | validated |
| RFC5 | -0.329 | 2.1E-07 | tarbase | MIMAT0000064 | 5985 | ENSG00000111445 | Degradome sequencing | positive |  | validated |
| RRM2 | -0.339 | 8.7E-08 | mirtarbase | MIMAT0000064 | 6241 | ENSG00000171848 | PAR-CLIP | Functional MTI (Weak) | 21572407 | validated |
| RRM2 | -0.339 | 8.7E-08 | tarbase | MIMAT0000064 | 6241 | ENSG00000171848 | Degradome sequencing//Degradome sequencing//Degradome sequencing | positive |  | validated |
| SGO1 | -0.288 | 6.3E-06 | tarbase | MIMAT0000064 | 151648 | ENSG00000129810 | Degradome sequencing | positive |  | validated |
| SMARCC1 | -0.258 | 5.4E-05 | mirtarbase | MIMAT0000064 | 6599 | ENSG00000173473 | CLASH | Functional MTI (Weak) | 23622248 | validated |
| SMARCC1 | -0.258 | 5.4E-05 | tarbase | MIMAT0000064 | 6599 | ENSG00000173473 | Degradome sequencing//Degradome sequencing//Degradome sequencing//Degradome sequencing//Degradome sequencing//Degradome sequencing//Degradome sequencing | positive |  | validated |
| SMC4 | -0.295 | 3.6E-06 | tarbase | MIMAT0000064 | 10051 | ENSG00000113810 | Degradome sequencing | positive |  | validated |
| SNRNP200 | -0.271 | 2.3E-05 | tarbase | MIMAT0000064 | 23020 | ENSG00000144028 | Degradome sequencing | positive |  | validated |
| SRSF1 | -0.266 | 3.1E-05 | tarbase | MIMAT0000064 | 6426 | ENSG00000136450 | Degradome sequencing//Degradome sequencing | positive |  | validated |
| SUPT16H | -0.293 | 4.1E-06 | mirtarbase | MIMAT0000064 | 11198 | ENSG00000092201 | CLASH | Functional MTI (Weak) | 23622248 | validated |
| TCF20 | -0.251 | 8.9E-05 | tarbase | MIMAT0000064 | 6942 | ENSG00000100207 | Degradome sequencing//Degradome sequencing | positive |  | validated |
| TFRC | -0.296 | 3.4E-06 | tarbase | MIMAT0000064 | 7037 | ENSG00000072274 | Degradome sequencing | positive |  | validated |
| TICRR | -0.253 | 7.4E-05 | tarbase | MIMAT0000064 | 90381 | ENSG00000140534 | Degradome sequencing | positive |  | validated |
| TSN | -0.250 | 9.3E-05 | tarbase | MIMAT0000064 | 7247 | ENSG00000211460 | Degradome sequencing | positive |  | validated |
| VRK1 | -0.264 | 3.7E-05 | tarbase | MIMAT0000064 | 7443 | ENSG00000100749 | Degradome sequencing | positive |  | validated |
| XPO1 | -0.282 | 9.9E-06 | tarbase | MIMAT0000064 | 7514 | ENSG00000082898 | Degradome sequencing//Degradome sequencing | positive |  | validated |
| ACKR3 | -0.339 | 8.7E-08 | tarbase | MIMAT0000104 | 57007 | ENSG00000144476 | Degradome sequencing | negative |  | validated |
| ADAM10 | -0.328 | 2.4E-07 | tarbase | MIMAT0000104 | 102 | ENSG00000137845 | Degradome sequencing | positive |  | validated |
| ADAM10 | -0.328 | 2.4E-07 | tarbase | MIMAT0000104 | 102 | ENSG00000137845 | Degradome sequencing | negative |  | validated |
| ADAM12 | -0.277 | 1.5E-05 | tarbase | MIMAT0000104 | 8038 | ENSG00000148848 | Degradome sequencing | negative |  | validated |
| ADGRF5 | -0.251 | 9.0E-05 | tarbase | MIMAT0000104 | 221395 | ENSG00000069122 | Degradome sequencing | positive |  | validated |
| AFF4 | -0.303 | 2.0E-06 | tarbase | MIMAT0000104 | 27125 | ENSG00000072364 | Degradome sequencing | positive |  | validated |
| AHNAK | -0.402 | 1.4E-10 | tarbase | MIMAT0000104 | 79026 | ENSG00000124942 | Degradome sequencing//Degradome sequencing | positive |  | validated |
| AKAP11 | -0.290 | 5.5E-06 | tarbase | MIMAT0000104 | 11215 | ENSG00000023516 | Degradome sequencing//Degradome sequencing//Degradome sequencing | positive |  | validated |
| ALCAM | -0.290 | 5.4E-06 | tarbase | MIMAT0000104 | 214 | ENSG00000170017 | Degradome sequencing | negative |  | validated |
| AMIGO2 | -0.271 | 2.2E-05 | tarbase | MIMAT0000104 | 347902 | ENSG00000139211 | Degradome sequencing | negative |  | validated |
| ANGPTL2 | -0.280 | 1.2E-05 | tarbase | MIMAT0000104 | 23452 | ENSG00000136859 | Degradome sequencing | negative |  | validated |
| ANKMY2 | -0.269 | 2.6E-05 | tarbase | MIMAT0000104 | 57037 | ENSG00000106524 | Degradome sequencing | positive |  | validated |
| ANKRA2 | -0.273 | 1.9E-05 | tarbase | MIMAT0000104 | 57763 | ENSG00000164331 | Degradome sequencing | positive |  | validated |
| ANKRD50 | -0.263 | 3.9E-05 | tarbase | MIMAT0000104 | 57182 | ENSG00000151458 | Degradome sequencing | positive |  | validated |
| ANTXR1 | -0.376 | 2.4E-09 | tarbase | MIMAT0000104 | 84168 | ENSG00000169604 | Degradome sequencing | negative |  | validated |
| ANTXR2 | -0.271 | 2.2E-05 | tarbase | MIMAT0000104 | 118429 | ENSG00000163297 | Degradome sequencing | negative |  | validated |
| AP3B1 | -0.331 | 1.8E-07 | tarbase | MIMAT0000104 | 8546 | ENSG00000132842 | Degradome sequencing | positive |  | validated |
| APC | -0.301 | 2.3E-06 | tarbase | MIMAT0000104 | 324 | ENSG00000134982 | Degradome sequencing | positive |  | validated |
| ARCN1 | -0.343 | 5.8E-08 | tarbase | MIMAT0000104 | 372 | ENSG00000095139 | Degradome sequencing | positive |  | validated |
| ARHGAP20 | -0.299 | 2.7E-06 | tarbase | MIMAT0000104 | 57569 | ENSG00000137727 | Degradome sequencing | negative |  | validated |
| ARHGAP32 | -0.292 | 4.6E-06 | tarbase | MIMAT0000104 | 9743 | ENSG00000134909 | Degradome sequencing//Degradome sequencing//Degradome sequencing | positive |  | validated |
| ARHGEF12 | -0.359 | 1.3E-08 | tarbase | MIMAT0000104 | 23365 | ENSG00000196914 | Degradome sequencing | positive |  | validated |
| ARID5B | -0.261 | 4.6E-05 | tarbase | MIMAT0000104 | 84159 | ENSG00000150347 | Degradome sequencing | positive |  | validated |
| ARL6IP5 | -0.273 | 1.9E-05 | tarbase | MIMAT0000104 | 10550 | ENSG00000144746 | Degradome sequencing | positive |  | validated |
| ARRDC3 | -0.312 | 9.4E-07 | tarbase | MIMAT0000104 | 57561 | ENSG00000113369 | Degradome sequencing | negative |  | validated |
| ASAH1 | -0.261 | 4.4E-05 | tarbase | MIMAT0000104 | 427 | ENSG00000104763 | Degradome sequencing | negative |  | validated |
| ATF7 | -0.319 | 5.0E-07 | tarbase | MIMAT0000104 | 11016 | ENSG00000170653 | Degradome sequencing//Degradome sequencing//Degradome sequencing | positive |  | validated |
| ATG2B | -0.280 | 1.2E-05 | tarbase | MIMAT0000104 | 55102 | ENSG00000066739 | Degradome sequencing | positive |  | validated |
| BAZ2A | -0.266 | 3.3E-05 | mirtarbase | MIMAT0000104 | 11176 | ENSG00000076108 | PAR-CLIP | Functional MTI (Weak) | 21572407 | validated |
| BAZ2A | -0.266 | 3.3E-05 | tarbase | MIMAT0000104 | 11176 | ENSG00000076108 | Degradome sequencing//Degradome sequencing//Degradome sequencing//Degradome sequencing//Degradome sequencing//Degradome sequencing | positive |  | validated |
| BAZ2B | -0.268 | 2.8E-05 | tarbase | MIMAT0000104 | 29994 | ENSG00000123636 | Degradome sequencing | positive |  | validated |
| BCAR3 | -0.272 | 2.1E-05 | tarbase | MIMAT0000104 | 8412 | ENSG00000137936 | Degradome sequencing | negative |  | validated |
| BHLHE40 | -0.338 | 9.4E-08 | tarbase | MIMAT0000104 | 8553 | ENSG00000134107 | Degradome sequencing//Degradome sequencing//Degradome sequencing | positive |  | validated |
| BICC1 | -0.282 | 1.0E-05 | tarbase | MIMAT0000104 | 80114 | ENSG00000122870 | Degradome sequencing | negative |  | validated |
| BMP4 | -0.311 | 1.0E-06 | tarbase | MIMAT0000104 | 652 | ENSG00000125378 | Degradome sequencing | negative |  | validated |
| BMPR2 | -0.299 | 2.7E-06 | tarbase | MIMAT0000104 | 659 | ENSG00000204217 | Degradome sequencing | positive |  | validated |
| BMPR2 | -0.299 | 2.7E-06 | tarbase | MIMAT0000104 | 659 | ENSG00000204217 | Degradome sequencing | negative |  | validated |
| C16orf72 | -0.304 | 1.8E-06 | mirtarbase | MIMAT0000104 | 29035 | ENSG00000182831 | PAR-CLIP | Functional MTI (Weak) | 23592263 | validated |
| C16orf72 | -0.304 | 1.8E-06 | mirtarbase | MIMAT0000104 | 29035 | ENSG00000182831 | PAR-CLIP | Functional MTI (Weak) | 24398324 | validated |
| C16orf72 | -0.304 | 1.8E-06 | mirtarbase | MIMAT0000104 | 29035 | ENSG00000182831 | PAR-CLIP//HITS-CLIP | Functional MTI (Weak) | 21572407 | validated |
| C16orf72 | -0.304 | 1.8E-06 | mirtarbase | MIMAT0000104 | 29035 | ENSG00000182831 | PAR-CLIP | Functional MTI (Weak) | 26701625 | validated |
| C16orf72 | -0.304 | 1.8E-06 | tarbase | MIMAT0000104 | 29035 | ENSG00000182831 | Degradome sequencing//Degradome sequencing//Degradome sequencing//Degradome sequencing//Degradome sequencing//Degradome sequencing//Degradome sequencing | positive |  | validated |
| CAB39 | -0.354 | 2.2E-08 | mirtarbase | MIMAT0000104 | 51719 | ENSG00000135932 | PAR-CLIP | Functional MTI (Weak) | 24398324 | validated |
| CAB39 | -0.354 | 2.2E-08 | tarbase | MIMAT0000104 | 51719 | ENSG00000135932 | Degradome sequencing | positive |  | validated |
| CALD1 | -0.288 | 6.4E-06 | tarbase | MIMAT0000104 | 800 | ENSG00000122786 | Degradome sequencing | negative |  | validated |
| CDH11 | -0.299 | 2.7E-06 | tarbase | MIMAT0000104 | 1009 | ENSG00000140937 | Degradome sequencing | negative |  | validated |
| CDH6 | -0.300 | 2.5E-06 | tarbase | MIMAT0000104 | 1004 | ENSG00000113361 | Degradome sequencing | negative |  | validated |
| CEMIP2 | -0.277 | 1.5E-05 | tarbase | MIMAT0000104 | 23670 | ENSG00000135048 | Degradome sequencing | negative |  | validated |
| CHMP3 | -0.307 | 1.4E-06 | tarbase | MIMAT0000104 | 51652 | ENSG00000115561 | Degradome sequencing//Degradome sequencing//Degradome sequencing//Degradome sequencing | positive |  | validated |
| CHP1 | -0.382 | 1.3E-09 | tarbase | MIMAT0000104 | 11261 | ENSG00000187446 | Degradome sequencing//Degradome sequencing | positive |  | validated |
| CHST15 | -0.271 | 2.2E-05 | tarbase | MIMAT0000104 | 51363 | ENSG00000182022 | Degradome sequencing | negative |  | validated |
| CITED2 | -0.261 | 4.6E-05 | tarbase | MIMAT0000104 | 10370 | ENSG00000164442 | Degradome sequencing | positive |  | validated |
| CLCN3 | -0.274 | 1.8E-05 | tarbase | MIMAT0000104 | 1182 | ENSG00000109572 | Degradome sequencing//Degradome sequencing//Degradome sequencing//Degradome sequencing//Degradome sequencing | positive |  | validated |
| CLIP1 | -0.286 | 7.2E-06 | mirtarbase | MIMAT0000104 | 6249 | ENSG00000130779 | PAR-CLIP | Functional MTI (Weak) | 21572407 | validated |
| CLIP1 | -0.286 | 7.2E-06 | tarbase | MIMAT0000104 | 6249 | ENSG00000130779 | Degradome sequencing | positive |  | validated |
| CLTC | -0.258 | 5.6E-05 | tarbase | MIMAT0000104 | 1213 | ENSG00000141367 | Degradome sequencing | positive |  | validated |
| CNTN3 | -0.273 | 1.8E-05 | tarbase | MIMAT0000104 | 5067 | ENSG00000113805 | Degradome sequencing | positive |  | validated |
| COBLL1 | -0.258 | 5.6E-05 | tarbase | MIMAT0000104 | 22837 | ENSG00000082438 | Degradome sequencing | positive |  | validated |
| COL1A1 | -0.251 | 9.1E-05 | tarbase | MIMAT0000104 | 1277 | ENSG00000108821 | Degradome sequencing | positive |  | validated |
| COL5A1 | -0.278 | 1.3E-05 | tarbase | MIMAT0000104 | 1289 | ENSG00000130635 | Degradome sequencing | negative |  | validated |
| CPEB4 | -0.292 | 4.6E-06 | tarbase | MIMAT0000104 | 80315 | ENSG00000113742 | Degradome sequencing | positive |  | validated |
| CSNK1A1 | -0.271 | 2.2E-05 | tarbase | MIMAT0000104 | 1452 | ENSG00000113712 | Degradome sequencing//Degradome sequencing//Degradome sequencing | positive |  | validated |
| CTNNA1 | -0.261 | 4.5E-05 | tarbase | MIMAT0000104 | 1495 | ENSG00000044115 | Degradome sequencing//Degradome sequencing//Degradome sequencing | positive |  | validated |
| CTNND1 | -0.332 | 1.6E-07 | tarbase | MIMAT0000104 | 1500 | ENSG00000198561 | Degradome sequencing//Degradome sequencing | positive |  | validated |
| CXADR | -0.260 | 5.0E-05 | tarbase | MIMAT0000104 | 1525 | ENSG00000154639 | Degradome sequencing | negative |  | validated |
| CYBRD1 | -0.399 | 2.1E-10 | tarbase | MIMAT0000104 | 79901 | ENSG00000071967 | Degradome sequencing | negative |  | validated |
| DAAM1 | -0.291 | 5.1E-06 | tarbase | MIMAT0000104 | 23002 | ENSG00000100592 | Degradome sequencing | negative |  | validated |
| DCLK1 | -0.272 | 2.0E-05 | tarbase | MIMAT0000104 | 9201 | ENSG00000133083 | Degradome sequencing | positive |  | validated |
| DHCR24 | -0.379 | 1.7E-09 | tarbase | MIMAT0000104 | 1718 | ENSG00000116133 | Degradome sequencing | positive |  | validated |
| DNAJB9 | -0.338 | 9.9E-08 | tarbase | MIMAT0000104 | 4189 | ENSG00000128590 | Degradome sequencing | negative |  | validated |
| DPP8 | -0.276 | 1.6E-05 | tarbase | MIMAT0000104 | 54878 | ENSG00000074603 | Degradome sequencing//Degradome sequencing//Degradome sequencing | positive |  | validated |
| DST | -0.287 | 7.0E-06 | mirtarbase | MIMAT0000104 | 667 | ENSG00000151914 | PAR-CLIP | Functional MTI (Weak) | 22012620 | validated |
| DST | -0.287 | 7.0E-06 | tarbase | MIMAT0000104 | 667 | ENSG00000151914 | Degradome sequencing | positive |  | validated |
| DUSP10 | -0.255 | 6.8E-05 | tarbase | MIMAT0000104 | 11221 | ENSG00000143507 | Degradome sequencing | negative |  | validated |
| DUSP4 | -0.254 | 7.1E-05 | tarbase | MIMAT0000104 | 1846 | ENSG00000120875 | Degradome sequencing | negative |  | validated |
| EDIL3 | -0.353 | 2.4E-08 | tarbase | MIMAT0000104 | 10085 | ENSG00000164176 | Degradome sequencing | positive |  | validated |
| ERBIN | -0.286 | 7.5E-06 | tarbase | MIMAT0000104 | 55914 | ENSG00000112851 | Degradome sequencing | positive |  | validated |
| ETV1 | -0.268 | 2.9E-05 | tarbase | MIMAT0000104 | 2115 | ENSG00000006468 | Degradome sequencing | positive |  | validated |
| ETV1 | -0.268 | 2.9E-05 | tarbase | MIMAT0000104 | 2115 | ENSG00000006468 | Degradome sequencing | negative |  | validated |
| F13A1 | -0.312 | 9.2E-07 | tarbase | MIMAT0000104 | 2162 | ENSG00000124491 | Degradome sequencing | negative |  | validated |
| F3 | -0.257 | 6.0E-05 | tarbase | MIMAT0000104 | 2152 | ENSG00000117525 | Degradome sequencing | negative |  | validated |
| FAM114A1 | -0.270 | 2.3E-05 | tarbase | MIMAT0000104 | 92689 | ENSG00000197712 | Degradome sequencing | negative |  | validated |
| FBXL7 | -0.256 | 6.4E-05 | tarbase | MIMAT0000104 | 23194 | ENSG00000183580 | Degradome sequencing | positive |  | validated |
| FCF1 | -0.263 | 4.0E-05 | mirtarbase | MIMAT0000104 | 51077 | ENSG00000119616 | PAR-CLIP | Functional MTI (Weak) | 21572407 | validated |
| FCF1 | -0.263 | 4.0E-05 | tarbase | MIMAT0000104 | 51077 | ENSG00000119616 | Degradome sequencing | positive |  | validated |
| FEM1C | -0.335 | 1.2E-07 | tarbase | MIMAT0000104 | 56929 | ENSG00000145780 | Degradome sequencing | positive |  | validated |
| FERMT2 | -0.256 | 6.2E-05 | tarbase | MIMAT0000104 | 10979 | ENSG00000073712 | Degradome sequencing//Degradome sequencing//Degradome sequencing | positive |  | validated |
| FIBIN | -0.260 | 4.7E-05 | tarbase | MIMAT0000104 | 387758 | ENSG00000176971 | Degradome sequencing | positive |  | validated |
| FLT1 | -0.301 | 2.3E-06 | tarbase | MIMAT0000104 | 2321 | ENSG00000102755 | Degradome sequencing | positive |  | validated |
| FNDC3A | -0.282 | 9.7E-06 | tarbase | MIMAT0000104 | 22862 | ENSG00000102531 | Degradome sequencing | positive |  | validated |
| FNIP1 | -0.290 | 5.4E-06 | tarbase | MIMAT0000104 | 96459 | ENSG00000217128 | Degradome sequencing | positive |  | validated |
| FOXA1 | -0.297 | 3.1E-06 | tarbase | MIMAT0000104 | 3169 | ENSG00000129514 | Degradome sequencing | positive |  | validated |
| FOXO1 | -0.282 | 1.0E-05 | mirtarbase | MIMAT0000104 | 2308 | ENSG00000150907 | Luciferase reporter assay//qRT-PCR//Western blot | Functional MTI | 24374340 | validated |
| FRK | -0.287 | 6.8E-06 | tarbase | MIMAT0000104 | 2444 | ENSG00000111816 | Degradome sequencing | positive |  | validated |
| FRMD4B | -0.283 | 9.6E-06 | tarbase | MIMAT0000104 | 23150 | ENSG00000114541 | Degradome sequencing | positive |  | validated |
| FRMD6 | -0.295 | 3.7E-06 | tarbase | MIMAT0000104 | 122786 | ENSG00000139926 | Degradome sequencing | negative |  | validated |
| FRY | -0.332 | 1.6E-07 | tarbase | MIMAT0000104 | 10129 | ENSG00000073910 | Degradome sequencing | positive |  | validated |
| FRY | -0.332 | 1.6E-07 | tarbase | MIMAT0000104 | 10129 | ENSG00000073910 | Degradome sequencing | negative |  | validated |
| FSTL1 | -0.299 | 2.6E-06 | tarbase | MIMAT0000104 | 11167 | ENSG00000163430 | Degradome sequencing | negative |  | validated |
| FYCO1 | -0.342 | 7.0E-08 | tarbase | MIMAT0000104 | 79443 | ENSG00000163820 | Degradome sequencing//Degradome sequencing | positive |  | validated |
| FYCO1 | -0.342 | 7.0E-08 | tarbase | MIMAT0000104 | 79443 | ENSG00000163820 | Degradome sequencing | negative |  | validated |
| GALNT1 | -0.251 | 9.1E-05 | tarbase | MIMAT0000104 | 2589 | ENSG00000141429 | Degradome sequencing | positive |  | validated |
| GALNT7 | -0.254 | 7.2E-05 | tarbase | MIMAT0000104 | 51809 | ENSG00000109586 | Degradome sequencing//Degradome sequencing//Degradome sequencing | positive |  | validated |
| GASK1B | -0.318 | 5.4E-07 | tarbase | MIMAT0000104 | 51313 | ENSG00000164125 | Degradome sequencing | negative |  | validated |
| GCC2 | -0.284 | 8.7E-06 | tarbase | MIMAT0000104 | 9648 | ENSG00000135968 | Degradome sequencing | positive |  | validated |
| GIT2 | -0.251 | 9.0E-05 | tarbase | MIMAT0000104 | 9815 | ENSG00000139436 | Degradome sequencing | positive |  | validated |
| GNAQ | -0.260 | 4.8E-05 | tarbase | MIMAT0000104 | 2776 | ENSG00000156052 | Degradome sequencing | positive |  | validated |
| GNG12 | -0.380 | 1.5E-09 | mirtarbase | MIMAT0000104 | 55970 | ENSG00000172380 | PAR-CLIP | Functional MTI (Weak) | 20371350 | validated |
| GNG12 | -0.380 | 1.5E-09 | tarbase | MIMAT0000104 | 55970 | ENSG00000172380 | Degradome sequencing | positive |  | validated |
| GPD1L | -0.299 | 2.6E-06 | tarbase | MIMAT0000104 | 23171 | ENSG00000152642 | Degradome sequencing | negative |  | validated |
| GSK3B | -0.253 | 7.6E-05 | tarbase | MIMAT0000104 | 2932 | ENSG00000082701 | Degradome sequencing//Degradome sequencing//Degradome sequencing | positive |  | validated |
| GUCY1A2 | -0.294 | 4.1E-06 | tarbase | MIMAT0000104 | 2977 | ENSG00000152402 | Degradome sequencing | positive |  | validated |
| GUCY1A2 | -0.294 | 4.1E-06 | tarbase | MIMAT0000104 | 2977 | ENSG00000152402 | Degradome sequencing | negative |  | validated |
| HACD2 | -0.335 | 1.3E-07 | tarbase | MIMAT0000104 | 201562 | ENSG00000206527 | Degradome sequencing | positive |  | validated |
| HBP1 | -0.335 | 1.3E-07 | tarbase | MIMAT0000104 | 26959 | ENSG00000105856 | Degradome sequencing | positive |  | validated |
| HBP1 | -0.335 | 1.3E-07 | tarbase | MIMAT0000104 | 26959 | ENSG00000105856 | Degradome sequencing | negative |  | validated |
| HCFC2 | -0.341 | 7.3E-08 | mirtarbase | MIMAT0000104 | 29915 | ENSG00000111727 | PAR-CLIP | Functional MTI (Weak) | 21572407 | validated |
| HCFC2 | -0.341 | 7.3E-08 | mirtarbase | MIMAT0000104 | 29915 | ENSG00000111727 | PAR-CLIP | Functional MTI (Weak) | 20371350 | validated |
| HERC3 | -0.290 | 5.5E-06 | tarbase | MIMAT0000104 | 8916 | ENSG00000138641 | Degradome sequencing | positive |  | validated |
| HFE | -0.294 | 3.9E-06 | tarbase | MIMAT0000104 | 3077 | ENSG00000010704 | Degradome sequencing | positive |  | validated |
| HRH1 | -0.339 | 9.0E-08 | tarbase | MIMAT0000104 | 3269 | ENSG00000196639 | Degradome sequencing | negative |  | validated |
| HTRA1 | -0.302 | 2.1E-06 | tarbase | MIMAT0000104 | 5654 | ENSG00000166033 | Degradome sequencing//Degradome sequencing | positive |  | validated |
| IDH1 | -0.310 | 1.1E-06 | tarbase | MIMAT0000104 | 3417 | ENSG00000138413 | Degradome sequencing | positive |  | validated |
| IDH1 | -0.310 | 1.1E-06 | tarbase | MIMAT0000104 | 3417 | ENSG00000138413 | Degradome sequencing | negative |  | validated |
| IFNGR1 | -0.328 | 2.4E-07 | tarbase | MIMAT0000104 | 3459 | ENSG00000027697 | Degradome sequencing | negative |  | validated |
| IGFBP5 | -0.292 | 4.8E-06 | tarbase | MIMAT0000104 | 3488 | ENSG00000115461 | Degradome sequencing | negative |  | validated |
| IL6ST | -0.292 | 4.7E-06 | tarbase | MIMAT0000104 | 3572 | ENSG00000134352 | Degradome sequencing | positive |  | validated |
| IL6ST | -0.292 | 4.7E-06 | tarbase | MIMAT0000104 | 3572 | ENSG00000134352 | Degradome sequencing | negative |  | validated |
| INHBA | -0.350 | 3.2E-08 | tarbase | MIMAT0000104 | 3624 | ENSG00000122641 | Degradome sequencing | negative |  | validated |
| INPP4B | -0.251 | 9.0E-05 | tarbase | MIMAT0000104 | 8821 | ENSG00000109452 | Degradome sequencing | positive |  | validated |
| INSIG2 | -0.382 | 1.3E-09 | tarbase | MIMAT0000104 | 51141 | ENSG00000125629 | Degradome sequencing | negative |  | validated |
| IQGAP1 | -0.335 | 1.2E-07 | tarbase | MIMAT0000104 | 8826 | ENSG00000140575 | Degradome sequencing | positive |  | validated |
| IQGAP2 | -0.265 | 3.5E-05 | tarbase | MIMAT0000104 | 10788 | ENSG00000145703 | Degradome sequencing | negative |  | validated |
| ITGA2 | -0.358 | 1.5E-08 | mirtarbase | MIMAT0000104 | 3673 | ENSG00000164171 | PAR-CLIP | Functional MTI (Weak) | 21572407 | validated |
| ITGA2 | -0.358 | 1.5E-08 | tarbase | MIMAT0000104 | 3673 | ENSG00000164171 | Degradome sequencing | positive |  | validated |
| ITGA2 | -0.358 | 1.5E-08 | tarbase | MIMAT0000104 | 3673 | ENSG00000164171 | Degradome sequencing | negative |  | validated |
| ITGB5 | -0.325 | 3.0E-07 | tarbase | MIMAT0000104 | 3693 | ENSG00000082781 | Degradome sequencing | negative |  | validated |
| ITPR1 | -0.286 | 7.4E-06 | tarbase | MIMAT0000104 | 3708 | ENSG00000150995 | Degradome sequencing//Degradome sequencing | positive |  | validated |
| ITPRIPL2 | -0.270 | 2.3E-05 | tarbase | MIMAT0000104 | 162073 | ENSG00000205730 | Degradome sequencing | positive |  | validated |
| JAK1 | -0.350 | 3.1E-08 | mirtarbase | MIMAT0000104 | 3716 | ENSG00000162434 | Luciferase reporter assay//qRT-PCR//Western blot//Coimmunoprecipitation//Chromatin immunoprecipitation//Immunofluorescence microscopy | Functional MTI | 24429361 | validated |
| JAK1 | -0.350 | 3.1E-08 | tarbase | MIMAT0000104 | 3716 | ENSG00000162434 | Degradome sequencing | positive |  | validated |
| JKAMP | -0.281 | 1.1E-05 | tarbase | MIMAT0000104 | 51528 | ENSG00000050130 | Degradome sequencing | positive |  | validated |
| KCTD10 | -0.287 | 6.8E-06 | tarbase | MIMAT0000104 | 83892 | ENSG00000110906 | Degradome sequencing | positive |  | validated |
| KCTD10 | -0.287 | 6.8E-06 | tarbase | MIMAT0000104 | 83892 | ENSG00000110906 | Degradome sequencing | negative |  | validated |
| KDM6A | -0.310 | 1.1E-06 | tarbase | MIMAT0000104 | 7403 | ENSG00000147050 | Degradome sequencing//Degradome sequencing | positive |  | validated |
| KITLG | -0.326 | 2.7E-07 | tarbase | MIMAT0000104 | 4254 | ENSG00000049130 | Degradome sequencing | positive |  | validated |
| KLF9 | -0.271 | 2.2E-05 | tarbase | MIMAT0000104 | 687 | ENSG00000119138 | Degradome sequencing | negative |  | validated |
| KLHL2 | -0.364 | 7.9E-09 | tarbase | MIMAT0000104 | 11275 | ENSG00000109466 | Degradome sequencing | negative |  | validated |
| KLHL28 | -0.287 | 6.9E-06 | tarbase | MIMAT0000104 | 54813 | ENSG00000179454 | Degradome sequencing//Degradome sequencing | positive |  | validated |
| KLHL4 | -0.254 | 7.1E-05 | tarbase | MIMAT0000104 | 56062 | ENSG00000102271 | Degradome sequencing | positive |  | validated |
| KTN1 | -0.283 | 9.4E-06 | tarbase | MIMAT0000104 | 3895 | ENSG00000126777 | Degradome sequencing//Degradome sequencing//Degradome sequencing | positive |  | validated |
| LATS2 | -0.271 | 2.2E-05 | mirtarbase | MIMAT0000104 | 26524 | ENSG00000150457 | Luciferase reporter assay//qRT-PCR//Western blot | Functional MTI | 25824045 | validated |
| LATS2 | -0.271 | 2.2E-05 | tarbase | MIMAT0000104 | 26524 | ENSG00000150457 | Degradome sequencing//Degradome sequencing//Degradome sequencing | positive |  | validated |
| LIPH | -0.299 | 2.7E-06 | tarbase | MIMAT0000104 | 200879 | ENSG00000163898 | Degradome sequencing | positive |  | validated |
| LRIG1 | -0.322 | 4.0E-07 | tarbase | MIMAT0000104 | 26018 | ENSG00000144749 | Degradome sequencing//Degradome sequencing | positive |  | validated |
| LRP10 | -0.258 | 5.4E-05 | tarbase | MIMAT0000104 | 26020 | ENSG00000197324 | Degradome sequencing | positive |  | validated |
| MAP1B | -0.277 | 1.5E-05 | tarbase | MIMAT0000104 | 4131 | ENSG00000131711 | Degradome sequencing//Degradome sequencing | positive |  | validated |
| MAP1B | -0.277 | 1.5E-05 | tarbase | MIMAT0000104 | 4131 | ENSG00000131711 | Degradome sequencing | negative |  | validated |
| MAP3K1 | -0.277 | 1.5E-05 | tarbase | MIMAT0000104 | 4214 | ENSG00000095015 | Degradome sequencing | negative |  | validated |
| MAP3K5 | -0.304 | 1.8E-06 | tarbase | MIMAT0000104 | 4217 | ENSG00000197442 | Degradome sequencing | negative |  | validated |
| MBNL2 | -0.284 | 8.5E-06 | tarbase | MIMAT0000104 | 10150 | ENSG00000139793 | Degradome sequencing | negative |  | validated |
| MFAP3 | -0.313 | 8.3E-07 | tarbase | MIMAT0000104 | 4238 | ENSG00000037749 | Degradome sequencing | negative |  | validated |
| MLPH | -0.340 | 8.4E-08 | tarbase | MIMAT0000104 | 79083 | ENSG00000115648 | Degradome sequencing | negative |  | validated |
| MYADM | -0.278 | 1.4E-05 | tarbase | MIMAT0000104 | 91663 | ENSG00000179820 | Degradome sequencing//Degradome sequencing | positive |  | validated |
| MYO5C | -0.265 | 3.3E-05 | tarbase | MIMAT0000104 | 55930 | ENSG00000128833 | Degradome sequencing | positive |  | validated |
| MYOF | -0.352 | 2.6E-08 | tarbase | MIMAT0000104 | 26509 | ENSG00000138119 | Degradome sequencing | positive |  | validated |
| MYOF | -0.352 | 2.6E-08 | tarbase | MIMAT0000104 | 26509 | ENSG00000138119 | Degradome sequencing | negative |  | validated |
| N4BP1 | -0.254 | 7.2E-05 | mirtarbase | MIMAT0000104 | 9683 | ENSG00000102921 | PAR-CLIP | Functional MTI (Weak) | 20371350 | validated |
| N4BP1 | -0.254 | 7.2E-05 | tarbase | MIMAT0000104 | 9683 | ENSG00000102921 | Degradome sequencing//Degradome sequencing//Degradome sequencing//Degradome sequencing//Degradome sequencing//Degradome sequencing//Degradome sequencing//Degrad | positive |  | validated |
| NCEH1 | -0.270 | 2.4E-05 | tarbase | MIMAT0000104 | 57552 | ENSG00000144959 | Degradome sequencing | negative |  | validated |
| NCKAP1 | -0.277 | 1.4E-05 | tarbase | MIMAT0000104 | 10787 | ENSG00000061676 | Degradome sequencing | positive |  | validated |
| NEDD4 | -0.303 | 1.9E-06 | tarbase | MIMAT0000104 | 4734 | ENSG00000069869 | Degradome sequencing | negative |  | validated |
| NPTN | -0.361 | 1.1E-08 | tarbase | MIMAT0000104 | 27020 | ENSG00000156642 | Degradome sequencing | negative |  | validated |
| NRIP1 | -0.265 | 3.5E-05 | tarbase | MIMAT0000104 | 8204 | ENSG00000180530 | Degradome sequencing//Degradome sequencing//Degradome sequencing | positive |  | validated |
| NRP1 | -0.297 | 3.1E-06 | tarbase | MIMAT0000104 | 8829 | ENSG00000099250 | Degradome sequencing//Degradome sequencing | positive |  | validated |
| NRXN3 | -0.262 | 3.9E-05 | tarbase | MIMAT0000104 | 9369 | ENSG00000021645 | Degradome sequencing | negative |  | validated |
| NT5E | -0.259 | 5.3E-05 | tarbase | MIMAT0000104 | 4907 | ENSG00000135318 | Degradome sequencing | negative |  | validated |
| NTN4 | -0.409 | 6.2E-11 | tarbase | MIMAT0000104 | 59277 | ENSG00000074527 | Degradome sequencing | positive |  | validated |
| NUAK1 | -0.347 | 4.2E-08 | tarbase | MIMAT0000104 | 9891 | ENSG00000074590 | Degradome sequencing | positive |  | validated |
| NUMB | -0.329 | 2.2E-07 | mirtarbase | MIMAT0000104 | 8650 | ENSG00000133961 | PAR-CLIP | Functional MTI (Weak) | 21572407 | validated |
| PAFAH1B2 | -0.250 | 9.2E-05 | mirtarbase | MIMAT0000104 | 5049 | ENSG00000168092 | PAR-CLIP | Functional MTI (Weak) | 23446348 | validated |
| PAFAH1B2 | -0.250 | 9.2E-05 | mirtarbase | MIMAT0000104 | 5049 | ENSG00000168092 | PAR-CLIP | Functional MTI (Weak) | 20371350 | validated |
| PAFAH1B2 | -0.250 | 9.2E-05 | tarbase | MIMAT0000104 | 5049 | ENSG00000168092 | Degradome sequencing//Degradome sequencing//Degradome sequencing//Degradome sequencing//Degradome sequencing | positive |  | validated |
| PCDH7 | -0.382 | 1.3E-09 | tarbase | MIMAT0000104 | 5099 | ENSG00000169851 | Degradome sequencing | positive |  | validated |
| PDS5B | -0.266 | 3.2E-05 | tarbase | MIMAT0000104 | 23047 | ENSG00000083642 | Degradome sequencing | positive |  | validated |
| PDZD2 | -0.285 | 7.9E-06 | tarbase | MIMAT0000104 | 23037 | ENSG00000133401 | Degradome sequencing | negative |  | validated |
| PERP | -0.270 | 2.4E-05 | tarbase | MIMAT0000104 | 64065 | ENSG00000112378 | Degradome sequencing//Degradome sequencing//Degradome sequencing//Degradome sequencing | positive |  | validated |
| PGM2L1 | -0.256 | 6.3E-05 | tarbase | MIMAT0000104 | 283209 | ENSG00000165434 | Degradome sequencing | positive |  | validated |
| PGM2L1 | -0.256 | 6.3E-05 | tarbase | MIMAT0000104 | 283209 | ENSG00000165434 | Degradome sequencing | negative |  | validated |
| PHACTR2 | -0.284 | 8.4E-06 | tarbase | MIMAT0000104 | 9749 | ENSG00000112419 | Degradome sequencing | positive |  | validated |
| PHF20 | -0.292 | 4.6E-06 | tarbase | MIMAT0000104 | 51230 | ENSG00000025293 | Degradome sequencing | positive |  | validated |
| PHLDB2 | -0.305 | 1.6E-06 | tarbase | MIMAT0000104 | 90102 | ENSG00000144824 | Degradome sequencing | negative |  | validated |
| PIK3R1 | -0.355 | 2.0E-08 | mirtarbase | MIMAT0000104 | 5295 | ENSG00000145675 | PAR-CLIP | Functional MTI (Weak) | 23446348 | validated |
| PIK3R1 | -0.355 | 2.0E-08 | mirtarbase | MIMAT0000104 | 5295 | ENSG00000145675 | PAR-CLIP//HITS-CLIP | Functional MTI (Weak) | 21572407 | validated |
| PIK3R1 | -0.355 | 2.0E-08 | mirtarbase | MIMAT0000104 | 5295 | ENSG00000145675 | HITS-CLIP | Functional MTI (Weak) | 23824327 | validated |
| PIK3R1 | -0.355 | 2.0E-08 | mirtarbase | MIMAT0000104 | 5295 | ENSG00000145675 | HITS-CLIP | Functional MTI (Weak) | 23313552 | validated |
| PJA2 | -0.343 | 6.0E-08 | tarbase | MIMAT0000104 | 9867 | ENSG00000198961 | Degradome sequencing | negative |  | validated |
| PLAU | -0.290 | 5.3E-06 | tarbase | MIMAT0000104 | 5328 | ENSG00000122861 | Degradome sequencing | negative |  | validated |
| PLPP3 | -0.257 | 6.1E-05 | tarbase | MIMAT0000104 | 8613 | ENSG00000162407 | Degradome sequencing//Degradome sequencing | positive |  | validated |
| PLPP4 | -0.276 | 1.6E-05 | tarbase | MIMAT0000104 | 196051 | ENSG00000203805 | Degradome sequencing | negative |  | validated |
| PLSCR4 | -0.289 | 5.9E-06 | tarbase | MIMAT0000104 | 57088 | ENSG00000114698 | Degradome sequencing//Degradome sequencing//Degradome sequencing | positive |  | validated |
| PMEPA1 | -0.302 | 2.2E-06 | tarbase | MIMAT0000104 | 56937 | ENSG00000124225 | Degradome sequencing | negative |  | validated |
| PRICKLE2 | -0.329 | 2.1E-07 | tarbase | MIMAT0000104 | 166336 | ENSG00000163637 | Degradome sequencing | negative |  | validated |
| PRKAA1 | -0.371 | 3.8E-09 | tarbase | MIMAT0000104 | 5562 | ENSG00000132356 | Degradome sequencing//Degradome sequencing//Degradome sequencing//Degradome sequencing//Degradome sequencing | positive |  | validated |
| PRKG1 | -0.343 | 5.1E-08 | tarbase | MIMAT0000104 | 5592 | ENSG00000185532 | Degradome sequencing | positive |  | validated |
| PRRC1 | -0.307 | 1.4E-06 | tarbase | MIMAT0000104 | 133619 | ENSG00000164244 | Degradome sequencing//Degradome sequencing | positive |  | validated |
| PRSS23 | -0.313 | 8.3E-07 | tarbase | MIMAT0000104 | 11098 | ENSG00000150687 | Degradome sequencing | negative |  | validated |
| PTGFRN | -0.255 | 6.8E-05 | tarbase | MIMAT0000104 | 5738 | ENSG00000134247 | Degradome sequencing//Degradome sequencing//Degradome sequencing | positive |  | validated |
| PTPRB | -0.268 | 2.7E-05 | tarbase | MIMAT0000104 | 5787 | ENSG00000127329 | Degradome sequencing | positive |  | validated |
| PTPRG | -0.290 | 5.3E-06 | tarbase | MIMAT0000104 | 5793 | ENSG00000144724 | Degradome sequencing | negative |  | validated |
| PTPRT | -0.262 | 4.1E-05 | tarbase | MIMAT0000104 | 11122 | ENSG00000196090 | Degradome sequencing | positive |  | validated |
| PXN | -0.277 | 1.4E-05 | tarbase | MIMAT0000104 | 5829 | ENSG00000089159 | Degradome sequencing | positive |  | validated |
| RAB11A | -0.271 | 2.2E-05 | tarbase | MIMAT0000104 | 8766 | ENSG00000103769 | Degradome sequencing | positive |  | validated |
| RAB11FIP5 | -0.250 | 9.2E-05 | tarbase | MIMAT0000104 | 26056 | ENSG00000135631 | Degradome sequencing | positive |  | validated |
| RAB14 | -0.301 | 2.3E-06 | tarbase | MIMAT0000104 | 51552 | ENSG00000119396 | Degradome sequencing | positive |  | validated |
| RAB27B | -0.334 | 1.4E-07 | tarbase | MIMAT0000104 | 5874 | ENSG00000041353 | Degradome sequencing | negative |  | validated |
| RGS4 | -0.260 | 4.7E-05 | tarbase | MIMAT0000104 | 5999 | ENSG00000117152 | Degradome sequencing | positive |  | validated |
| RIN2 | -0.385 | 9.1E-10 | tarbase | MIMAT0000104 | 54453 | ENSG00000132669 | Degradome sequencing | positive |  | validated |
| RIN2 | -0.385 | 9.1E-10 | tarbase | MIMAT0000104 | 54453 | ENSG00000132669 | Degradome sequencing | negative |  | validated |
| RNF103 | -0.284 | 8.6E-06 | tarbase | MIMAT0000104 | 7844 | ENSG00000239305 | Degradome sequencing//Degradome sequencing | positive |  | validated |
| RNF111 | -0.304 | 1.8E-06 | tarbase | MIMAT0000104 | 54778 | ENSG00000157450 | Degradome sequencing | positive |  | validated |
| RNF217 | -0.251 | 9.1E-05 | tarbase | MIMAT0000104 | 154214 | ENSG00000146373 | Degradome sequencing//Degradome sequencing//Degradome sequencing//Degradome sequencing | positive |  | validated |
| RP2 | -0.263 | 3.9E-05 | tarbase | MIMAT0000104 | 6102 | ENSG00000102218 | Degradome sequencing | negative |  | validated |
| RUNX1 | -0.304 | 1.8E-06 | tarbase | MIMAT0000104 | 861 | ENSG00000159216 | Degradome sequencing | positive |  | validated |
| RUNX1T1 | -0.303 | 1.8E-06 | mirtarbase | MIMAT0000104 | 862 | ENSG00000079102 | PAR-CLIP | Functional MTI (Weak) | 21572407 | validated |
| SALL1 | -0.254 | 6.7E-05 | mirtarbase | MIMAT0000104 | 6299 | ENSG00000103449 | PAR-CLIP | Functional MTI (Weak) | 21572407 | validated |
| SALL1 | -0.254 | 6.7E-05 | mirtarbase | MIMAT0000104 | 6299 | ENSG00000103449 | PAR-CLIP | Functional MTI (Weak) | 20371350 | validated |
| SALL1 | -0.254 | 6.7E-05 | tarbase | MIMAT0000104 | 6299 | ENSG00000103449 | Degradome sequencing//Degradome sequencing//Degradome sequencing//Degradome sequencing | positive |  | validated |
| SALL1 | -0.254 | 6.7E-05 | tarbase | MIMAT0000104 | 6299 | ENSG00000103449 | Degradome sequencing | negative |  | validated |
| SEC23A | -0.253 | 7.7E-05 | tarbase | MIMAT0000104 | 10484 | ENSG00000100934 | Degradome sequencing | positive |  | validated |
| SEC24A | -0.298 | 2.9E-06 | tarbase | MIMAT0000104 | 10802 | ENSG00000113615 | Degradome sequencing | negative |  | validated |
| SEC24D | -0.309 | 1.2E-06 | tarbase | MIMAT0000104 | 9871 | ENSG00000150961 | Degradome sequencing | positive |  | validated |
| SEC24D | -0.309 | 1.2E-06 | tarbase | MIMAT0000104 | 9871 | ENSG00000150961 | Degradome sequencing | negative |  | validated |
| SEL1L | -0.335 | 1.3E-07 | tarbase | MIMAT0000104 | 6400 | ENSG00000071537 | Degradome sequencing | positive |  | validated |
| SEMA3C | -0.322 | 4.1E-07 | tarbase | MIMAT0000104 | 10512 | ENSG00000075223 | Degradome sequencing//Degradome sequencing | positive |  | validated |
| SEMA5A | -0.273 | 2.0E-05 | tarbase | MIMAT0000104 | 9037 | ENSG00000112902 | Degradome sequencing | negative |  | validated |
| SEPTIN2 | -0.255 | 6.7E-05 | tarbase | MIMAT0000104 | 4735 | ENSG00000168385 | Degradome sequencing | positive |  | validated |
| SERINC5 | -0.276 | 1.6E-05 | tarbase | MIMAT0000104 | 256987 | ENSG00000164300 | Degradome sequencing | negative |  | validated |
| SGIP1 | -0.276 | 1.6E-05 | tarbase | MIMAT0000104 | 84251 | ENSG00000118473 | Degradome sequencing | negative |  | validated |
| SGMS2 | -0.291 | 4.9E-06 | tarbase | MIMAT0000104 | 166929 | ENSG00000164023 | Degradome sequencing//Degradome sequencing//Degradome sequencing | positive |  | validated |
| SH3BP4 | -0.258 | 5.7E-05 | tarbase | MIMAT0000104 | 23677 | ENSG00000130147 | Degradome sequencing//Degradome sequencing | positive |  | validated |
| SH3D19 | -0.301 | 2.3E-06 | tarbase | MIMAT0000104 | 152503 | ENSG00000109686 | Degradome sequencing | positive |  | validated |
| SLC1A1 | -0.293 | 4.2E-06 | tarbase | MIMAT0000104 | 6505 | ENSG00000106688 | Degradome sequencing | negative |  | validated |
| SLC24A2 | -0.326 | 2.4E-07 | tarbase | MIMAT0000104 | 25769 | ENSG00000155886 | Degradome sequencing | positive |  | validated |
| SLC35D1 | -0.286 | 7.5E-06 | tarbase | MIMAT0000104 | 23169 | ENSG00000116704 | Degradome sequencing | positive |  | validated |
| SLC38A2 | -0.352 | 2.5E-08 | tarbase | MIMAT0000104 | 54407 | ENSG00000134294 | Degradome sequencing | negative |  | validated |
| SLC41A2 | -0.326 | 2.9E-07 | tarbase | MIMAT0000104 | 84102 | ENSG00000136052 | Degradome sequencing//Degradome sequencing | positive |  | validated |
| SLC46A3 | -0.369 | 5.0E-09 | tarbase | MIMAT0000104 | 283537 | ENSG00000139508 | Degradome sequencing | negative |  | validated |
| SLIT2 | -0.258 | 5.2E-05 | tarbase | MIMAT0000104 | 9353 | ENSG00000145147 | Degradome sequencing//Degradome sequencing//Degradome sequencing | positive |  | validated |
| SLMAP | -0.330 | 2.0E-07 | tarbase | MIMAT0000104 | 7871 | ENSG00000163681 | Degradome sequencing | positive |  | validated |
| SP1 | -0.254 | 7.3E-05 | tarbase | MIMAT0000104 | 6667 | ENSG00000185591 | Degradome sequencing//Degradome sequencing//Degradome sequencing//Degradome sequencing | positive |  | validated |
| SPARC | -0.258 | 5.5E-05 | tarbase | MIMAT0000104 | 6678 | ENSG00000113140 | Degradome sequencing | negative |  | validated |
| SPOPL | -0.299 | 2.8E-06 | tarbase | MIMAT0000104 | 339745 | ENSG00000144228 | Degradome sequencing | negative |  | validated |
| STEAP4 | -0.270 | 2.4E-05 | tarbase | MIMAT0000104 | 79689 | ENSG00000127954 | Degradome sequencing | positive |  | validated |
| STXBP5 | -0.261 | 4.4E-05 | tarbase | MIMAT0000104 | 134957 | ENSG00000164506 | Degradome sequencing | positive |  | validated |
| SYTL2 | -0.382 | 1.3E-09 | tarbase | MIMAT0000104 | 54843 | ENSG00000137501 | Degradome sequencing | negative |  | validated |
| TBCEL | -0.277 | 1.5E-05 | tarbase | MIMAT0000104 | 219899 | ENSG00000154114 | Degradome sequencing | positive |  | validated |
| TC2N | -0.262 | 4.2E-05 | tarbase | MIMAT0000104 | 123036 | ENSG00000165929 | Degradome sequencing | positive |  | validated |
| TCF4 | -0.277 | 1.5E-05 | tarbase | MIMAT0000104 | 6925 | ENSG00000196628 | Degradome sequencing | positive |  | validated |
| TENM4 | -0.269 | 2.6E-05 | tarbase | MIMAT0000104 | 26011 | ENSG00000149256 | Degradome sequencing | negative |  | validated |
| TGFBR1 | -0.309 | 1.2E-06 | tarbase | MIMAT0000104 | 7046 | ENSG00000106799 | Degradome sequencing | negative |  | validated |
| TGFBR2 | -0.254 | 7.4E-05 | tarbase | MIMAT0000104 | 7048 | ENSG00000163513 | Degradome sequencing | positive |  | validated |
| THBS1 | -0.281 | 1.1E-05 | tarbase | MIMAT0000104 | 7057 | ENSG00000137801 | Degradome sequencing | negative |  | validated |
| THSD4 | -0.315 | 7.4E-07 | tarbase | MIMAT0000104 | 79875 | ENSG00000187720 | Degradome sequencing | negative |  | validated |
| TJP1 | -0.259 | 5.3E-05 | mirtarbase | MIMAT0000104 | 7082 | ENSG00000104067 | PAR-CLIP | Functional MTI (Weak) | 21572407 | validated |
| TJP1 | -0.259 | 5.3E-05 | mirtarbase | MIMAT0000104 | 7082 | ENSG00000277401 | PAR-CLIP | Functional MTI (Weak) | 21572407 | validated |
| TMBIM6 | -0.275 | 1.7E-05 | tarbase | MIMAT0000104 | 7009 | ENSG00000139644 | Degradome sequencing//Degradome sequencing//Degradome sequencing//Degradome sequencing//Degradome sequencing | positive |  | validated |
| TMCC1 | -0.304 | 1.8E-06 | tarbase | MIMAT0000104 | 23023 | ENSG00000172765 | Degradome sequencing//Degradome sequencing | positive |  | validated |
| TMEM30A | -0.275 | 1.7E-05 | tarbase | MIMAT0000104 | 55754 | ENSG00000112697 | Degradome sequencing//Degradome sequencing//Degradome sequencing | positive |  | validated |
| TMEM87A | -0.282 | 9.7E-06 | mirtarbase | MIMAT0000104 | 25963 | ENSG00000103978 | CLASH | Functional MTI (Weak) | 23622248 | validated |
| TMEM87A | -0.282 | 9.7E-06 | tarbase | MIMAT0000104 | 25963 | ENSG00000103978 | Degradome sequencing | positive |  | validated |
| TMEM87B | -0.316 | 6.7E-07 | tarbase | MIMAT0000104 | 84910 | ENSG00000153214 | Degradome sequencing | negative |  | validated |
| TNFAIP1 | -0.298 | 2.9E-06 | tarbase | MIMAT0000104 | 7126 | ENSG00000109079 | Degradome sequencing//Degradome sequencing | positive |  | validated |
| TP53INP1 | -0.280 | 1.2E-05 | tarbase | MIMAT0000104 | 94241 | ENSG00000164938 | Degradome sequencing | negative |  | validated |
| TRPM4 | -0.293 | 4.4E-06 | tarbase | MIMAT0000104 | 54795 | ENSG00000130529 | Degradome sequencing | positive |  | validated |
| TSC22D1 | -0.289 | 5.9E-06 | tarbase | MIMAT0000104 | 8848 | ENSG00000102804 | Degradome sequencing | positive |  | validated |
| TSC22D1 | -0.289 | 5.9E-06 | tarbase | MIMAT0000104 | 8848 | ENSG00000102804 | Degradome sequencing | negative |  | validated |
| TWSG1 | -0.280 | 1.2E-05 | tarbase | MIMAT0000104 | 57045 | ENSG00000128791 | Degradome sequencing | positive |  | validated |
| UBE4A | -0.259 | 5.3E-05 | tarbase | MIMAT0000104 | 9354 | ENSG00000110344 | Degradome sequencing//Degradome sequencing | positive |  | validated |
| UBL3 | -0.360 | 1.2E-08 | tarbase | MIMAT0000104 | 5412 | ENSG00000122042 | Degradome sequencing | positive |  | validated |
| UGCG | -0.289 | 6.0E-06 | tarbase | MIMAT0000104 | 7357 | ENSG00000148154 | Degradome sequencing | negative |  | validated |
| USP3 | -0.254 | 7.1E-05 | tarbase | MIMAT0000104 | 9960 | ENSG00000140455 | Degradome sequencing | positive |  | validated |
| UTRN | -0.379 | 1.7E-09 | tarbase | MIMAT0000104 | 7402 | ENSG00000152818 | Degradome sequencing | positive |  | validated |
| VCAN | -0.302 | 2.1E-06 | mirtarbase | MIMAT0000104 | 1462 | ENSG00000038427 | PAR-CLIP | Functional MTI (Weak) | 21572407 | validated |
| VCAN | -0.302 | 2.1E-06 | tarbase | MIMAT0000104 | 1462 | ENSG00000038427 | Degradome sequencing | positive |  | validated |
| VEGFC | -0.293 | 4.3E-06 | tarbase | MIMAT0000104 | 7424 | ENSG00000150630 | Degradome sequencing | positive |  | validated |
| VPS13C | -0.295 | 3.8E-06 | tarbase | MIMAT0000104 | 54832 | ENSG00000129003 | Degradome sequencing | negative |  | validated |
| VWA5A | -0.262 | 4.2E-05 | tarbase | MIMAT0000104 | 4013 | ENSG00000110002 | Degradome sequencing | negative |  | validated |
| WLS | -0.252 | 8.2E-05 | tarbase | MIMAT0000104 | 79971 | ENSG00000116729 | Degradome sequencing | positive |  | validated |
| WLS | -0.252 | 8.2E-05 | tarbase | MIMAT0000104 | 79971 | ENSG00000116729 | Degradome sequencing | negative |  | validated |
| WNT5A | -0.288 | 6.4E-06 | tarbase | MIMAT0000104 | 7474 | ENSG00000114251 | Degradome sequencing | negative |  | validated |
| WNT7B | -0.276 | 1.5E-05 | tarbase | MIMAT0000104 | 7477 | ENSG00000188064 | Degradome sequencing | negative |  | validated |
| YIPF5 | -0.353 | 2.4E-08 | tarbase | MIMAT0000104 | 81555 | ENSG00000145817 | Degradome sequencing | positive |  | validated |
| YPEL2 | -0.351 | 3.0E-08 | tarbase | MIMAT0000104 | 388403 | ENSG00000175155 | Degradome sequencing | negative |  | validated |
| ZBTB38 | -0.370 | 4.4E-09 | mirtarbase | MIMAT0000104 | 253461 | ENSG00000177311 | PAR-CLIP | Functional MTI (Weak) | 23592263 | validated |
| ZBTB38 | -0.370 | 4.4E-09 | tarbase | MIMAT0000104 | 253461 | ENSG00000177311 | Degradome sequencing | positive |  | validated |
| ZBTB38 | -0.370 | 4.4E-09 | tarbase | MIMAT0000104 | 253461 | ENSG00000177311 | Degradome sequencing | negative |  | validated |
| ZFP36L1 | -0.294 | 4.1E-06 | tarbase | MIMAT0000104 | 677 | ENSG00000185650 | Degradome sequencing | positive |  | validated |
| ZFPM2 | -0.283 | 9.3E-06 | tarbase | MIMAT0000104 | 23414 | ENSG00000169946 | Degradome sequencing//Degradome sequencing | positive |  | validated |
| ZNF148 | -0.274 | 1.8E-05 | tarbase | MIMAT0000104 | 7707 | ENSG00000163848 | Degradome sequencing | positive |  | validated |
| ZNF365 | -0.279 | 1.3E-05 | tarbase | MIMAT0000104 | 22891 | ENSG00000138311 | Degradome sequencing | negative |  | validated |
| ZNF587 | -0.295 | 3.6E-06 | tarbase | MIMAT0000104 | 84914 | ENSG00000198466 | Degradome sequencing | positive |  | validated |
| ZNF844 | -0.282 | 1.0E-05 | tarbase | MIMAT0000104 | 284391 | ENSG00000223547 | Degradome sequencing | positive |  | validated |
| ACTR3B | -0.311 | 1.0E-06 | mirtarbase | MIMAT0000446 | 57180 | ENSG00000133627 | HITS-CLIP | Functional MTI (Weak) | 24374217 | validated |
| BOLA1 | -0.255 | 6.9E-05 | mirtarbase | MIMAT0000446 | 51027 | ENSG00000178096 | HITS-CLIP | Functional MTI (Weak) | 23824327 | validated |
| HTATSF1 | -0.258 | 5.5E-05 | tarbase | MIMAT0000446 | 27336 | ENSG00000102241 | Degradome sequencing | positive |  | validated |
| RGMA | -0.306 | 1.5E-06 | mirtarbase | MIMAT0000446 | 56963 | ENSG00000182175 | Luciferase reporter assay//qRT-PCR//Western blot | Functional MTI | 24517116 | validated |
| RGMA | -0.306 | 1.5E-06 | tarbase | MIMAT0000446 | 56963 | ENSG00000182175 | Degradome sequencing | positive |  | validated |
| SFRP1 | -0.265 | 3.5E-05 | mirtarbase | MIMAT0000446 | 6422 | ENSG00000104332 | Luciferase reporter assay//qRT-PCR//Western blot | Functional MTI | 24517116 | validated |
| SFRP1 | -0.265 | 3.5E-05 | tarbase | MIMAT0000446 | 6422 | ENSG00000104332 | Degradome sequencing | positive |  | validated |
| ACSL4 | -0.280 | 1.1E-05 | tarbase | MIMAT0000259 | 2182 | ENSG00000068366 | Degradome sequencing | positive |  | validated |
| ACTG2 | -0.262 | 4.3E-05 | tarbase | MIMAT0000259 | 72 | ENSG00000163017 | Degradome sequencing | positive |  | validated |
| ADARB1 | -0.261 | 4.6E-05 | tarbase | MIMAT0000259 | 104 | ENSG00000197381 | Degradome sequencing | positive |  | validated |
| AIF1 | -0.335 | 1.2E-07 | tarbase | MIMAT0000259 | 199 | ENSG00000204472 | Degradome sequencing | positive |  | validated |
| BACH2 | -0.250 | 9.3E-05 | tarbase | MIMAT0000259 | 60468 | ENSG00000112182 | Degradome sequencing | positive |  | validated |
| BIVM | -0.263 | 3.9E-05 | tarbase | MIMAT0000259 | 54841 | ENSG00000134897 | Degradome sequencing | positive |  | validated |
| BIVM | -0.263 | 3.9E-05 | tarbase | MIMAT0000259 | 54841 | ENSG00000134897 | Degradome sequencing | negative |  | validated |
| BMP2 | -0.256 | 6.3E-05 | tarbase | MIMAT0000259 | 650 | ENSG00000125845 | Degradome sequencing | positive |  | validated |
| BOC | -0.277 | 1.4E-05 | tarbase | MIMAT0000259 | 91653 | ENSG00000144857 | Degradome sequencing | negative |  | validated |
| BTN3A3 | -0.263 | 3.9E-05 | tarbase | MIMAT0000259 | 10384 | ENSG00000111801 | Degradome sequencing | negative |  | validated |
| C1QA | -0.315 | 7.0E-07 | tarbase | MIMAT0000259 | 712 | ENSG00000173372 | Degradome sequencing | positive |  | validated |
| CAMK4 | -0.288 | 6.0E-06 | tarbase | MIMAT0000259 | 814 | ENSG00000152495 | Degradome sequencing | negative |  | validated |
| CCDC50 | -0.288 | 6.4E-06 | tarbase | MIMAT0000259 | 152137 | ENSG00000152492 | Degradome sequencing | negative |  | validated |
| CCND2 | -0.311 | 9.8E-07 | mirtarbase | MIMAT0000259 | 894 | ENSG00000118971 | Flow//Luciferase reporter assay//qRT-PCR//Western blot | Functional MTI | 22848417 | validated |
| CCND2 | -0.311 | 9.8E-07 | tarbase | MIMAT0000259 | 894 | ENSG00000118971 | Degradome sequencing//Degradome sequencing | positive |  | validated |
| CD1C | -0.254 | 7.1E-05 | tarbase | MIMAT0000259 | 911 | ENSG00000158481 | Degradome sequencing | positive |  | validated |
| CD33 | -0.324 | 3.4E-07 | tarbase | MIMAT0000259 | 945 | ENSG00000105383 | Degradome sequencing | positive |  | validated |
| CD48 | -0.275 | 1.6E-05 | tarbase | MIMAT0000259 | 962 | ENSG00000117091 | Degradome sequencing | positive |  | validated |
| CDX1 | -0.367 | 4.4E-09 | tarbase | MIMAT0000259 | 1044 | ENSG00000113722 | Degradome sequencing | positive |  | validated |
| CSK | -0.320 | 4.7E-07 | tarbase | MIMAT0000259 | 1445 | ENSG00000103653 | Degradome sequencing | positive |  | validated |
| CXorf65 | -0.250 | 8.8E-05 | tarbase | MIMAT0000259 | 158830 | ENSG00000204165 | Degradome sequencing | positive |  | validated |
| ENPP2 | -0.297 | 3.2E-06 | tarbase | MIMAT0000259 | 5168 | ENSG00000136960 | Degradome sequencing | negative |  | validated |
| EOGT | -0.296 | 3.4E-06 | tarbase | MIMAT0000259 | 285203 | ENSG00000163378 | Degradome sequencing | negative |  | validated |
| ETS1 | -0.317 | 6.2E-07 | tarbase | MIMAT0000259 | 2113 | ENSG00000134954 | Degradome sequencing | positive |  | validated |
| FAM20A | -0.266 | 3.2E-05 | tarbase | MIMAT0000259 | 54757 | ENSG00000108950 | Degradome sequencing | negative |  | validated |
| FGD2 | -0.395 | 2.9E-10 | tarbase | MIMAT0000259 | 221472 | ENSG00000146192 | Degradome sequencing | positive |  | validated |
| FMNL3 | -0.255 | 6.9E-05 | mirtarbase | MIMAT0000259 | 91010 | ENSG00000161791 | CLASH | Functional MTI (Weak) | 23622248 | validated |
| FOXF2 | -0.250 | 9.4E-05 | mirtarbase | MIMAT0000259 | 2295 | ENSG00000137273 | Luciferase reporter assay | Functional MTI | 23383207 | validated |
| FOXF2 | -0.250 | 9.4E-05 | tarbase | MIMAT0000259 | 2295 | ENSG00000137273 | Degradome sequencing//Degradome sequencing//Degradome sequencing | positive |  | validated |
| FYN | -0.276 | 1.5E-05 | tarbase | MIMAT0000259 | 2534 | ENSG00000010810 | Degradome sequencing | positive |  | validated |
| GNAI2 | -0.264 | 3.6E-05 | tarbase | MIMAT0000259 | 2771 | ENSG00000114353 | Degradome sequencing//Degradome sequencing | positive |  | validated |
| GNL1 | -0.270 | 2.3E-05 | mirtarbase | MIMAT0000259 | 2794 | ENSG00000206492 | CLASH | Functional MTI (Weak) | 23622248 | validated |
| GNL1 | -0.270 | 2.3E-05 | mirtarbase | MIMAT0000259 | 2794 | ENSG00000235986 | CLASH | Functional MTI (Weak) | 23622248 | validated |
| GNL1 | -0.270 | 2.3E-05 | mirtarbase | MIMAT0000259 | 2794 | ENSG00000228581 | CLASH | Functional MTI (Weak) | 23622248 | validated |
| GNL1 | -0.270 | 2.3E-05 | mirtarbase | MIMAT0000259 | 2794 | ENSG00000204590 | CLASH | Functional MTI (Weak) | 23622248 | validated |
| GNL1 | -0.270 | 2.3E-05 | mirtarbase | MIMAT0000259 | 2794 | ENSG00000226882 | CLASH | Functional MTI (Weak) | 23622248 | validated |
| GNL1 | -0.270 | 2.3E-05 | mirtarbase | MIMAT0000259 | 2794 | ENSG00000206412 | CLASH | Functional MTI (Weak) | 23622248 | validated |
| GNL1 | -0.270 | 2.3E-05 | mirtarbase | MIMAT0000259 | 2794 | ENSG00000229470 | CLASH | Functional MTI (Weak) | 23622248 | validated |
| GNL1 | -0.270 | 2.3E-05 | mirtarbase | MIMAT0000259 | 2794 | ENSG00000232143 | CLASH | Functional MTI (Weak) | 23622248 | validated |
| GPR15 | -0.262 | 3.8E-05 | tarbase | MIMAT0000259 | 2838 | ENSG00000154165 | Degradome sequencing | positive |  | validated |
| HCK | -0.274 | 1.8E-05 | tarbase | MIMAT0000259 | 3055 | ENSG00000101336 | Degradome sequencing | positive |  | validated |
| HLA-DMA | -0.313 | 8.3E-07 | tarbase | MIMAT0000259 | 3108 | ENSG00000204257 | Degradome sequencing | negative |  | validated |
| HLA-DPB1 | -0.320 | 4.9E-07 | tarbase | MIMAT0000259 | 3115 | ENSG00000223865 | Degradome sequencing | positive |  | validated |
| HLX | -0.275 | 1.7E-05 | tarbase | MIMAT0000259 | 3142 | ENSG00000136630 | Degradome sequencing | negative |  | validated |
| HVCN1 | -0.324 | 3.4E-07 | tarbase | MIMAT0000259 | 84329 | ENSG00000122986 | Degradome sequencing | positive |  | validated |
| IFI16 | -0.288 | 6.5E-06 | tarbase | MIMAT0000259 | 3428 | ENSG00000163565 | Degradome sequencing | negative |  | validated |
| IL10RA | -0.338 | 9.5E-08 | tarbase | MIMAT0000259 | 3587 | ENSG00000110324 | Degradome sequencing | positive |  | validated |
| IL15RA | -0.251 | 8.7E-05 | tarbase | MIMAT0000259 | 3601 | ENSG00000134470 | Degradome sequencing | positive |  | validated |
| IL16 | -0.293 | 4.3E-06 | tarbase | MIMAT0000259 | 3603 | ENSG00000172349 | Degradome sequencing | positive |  | validated |
| IL21R | -0.286 | 7.4E-06 | tarbase | MIMAT0000259 | 50615 | ENSG00000103522 | Degradome sequencing | positive |  | validated |
| IL7 | -0.291 | 5.2E-06 | tarbase | MIMAT0000259 | 3574 | ENSG00000104432 | Degradome sequencing | negative |  | validated |
| ITGB2 | -0.328 | 2.5E-07 | tarbase | MIMAT0000259 | 3689 | ENSG00000160255 | Degradome sequencing | positive |  | validated |
| JAZF1 | -0.329 | 2.1E-07 | tarbase | MIMAT0000259 | 221895 | ENSG00000153814 | Degradome sequencing//Degradome sequencing//Degradome sequencing | positive |  | validated |
| KCNT2 | -0.259 | 5.1E-05 | tarbase | MIMAT0000259 | 343450 | ENSG00000162687 | Degradome sequencing | negative |  | validated |
| LARP6 | -0.252 | 8.5E-05 | tarbase | MIMAT0000259 | 55323 | ENSG00000166173 | Degradome sequencing | positive |  | validated |
| LBH | -0.278 | 1.3E-05 | tarbase | MIMAT0000259 | 81606 | ENSG00000213626 | Degradome sequencing | positive |  | validated |
| LYSMD2 | -0.260 | 4.8E-05 | tarbase | MIMAT0000259 | 256586 | ENSG00000140280 | Degradome sequencing | positive |  | validated |
| MAP3K3 | -0.283 | 9.2E-06 | mirtarbase | MIMAT0000259 | 4215 | ENSG00000198909 | PAR-CLIP | Functional MTI (Weak) | 21572407 | validated |
| MAP7D3 | -0.268 | 2.7E-05 | tarbase | MIMAT0000259 | 79649 | ENSG00000129680 | Degradome sequencing | positive |  | validated |
| MBNL1 | -0.292 | 4.7E-06 | tarbase | MIMAT0000259 | 4154 | ENSG00000152601 | Degradome sequencing//Degradome sequencing//Degradome sequencing//Degradome sequencing//Degradome sequencing//Degradome sequencing//Degradome sequencing | positive |  | validated |
| MBNL1 | -0.292 | 4.7E-06 | tarbase | MIMAT0000259 | 4154 | ENSG00000152601 | Degradome sequencing | negative |  | validated |
| MEF2C | -0.341 | 7.4E-08 | tarbase | MIMAT0000259 | 4208 | ENSG00000081189 | Degradome sequencing | negative |  | validated |
| MORF4L1 | -0.304 | 1.8E-06 | mirtarbase | MIMAT0000259 | 10933 | ENSG00000185787 | PAR-CLIP | Functional MTI (Weak) | 21572407 | validated |
| MORF4L1 | -0.304 | 1.8E-06 | mirtarbase | MIMAT0000259 | 10933 | ENSG00000185787 | PAR-CLIP | Functional MTI (Weak) | 27292025 | validated |
| MORF4L1 | -0.304 | 1.8E-06 | tarbase | MIMAT0000259 | 10933 | ENSG00000185787 | Degradome sequencing | positive |  | validated |
| MSN | -0.301 | 2.2E-06 | tarbase | MIMAT0000259 | 4478 | ENSG00000147065 | Degradome sequencing | positive |  | validated |
| NAALADL1 | -0.340 | 7.8E-08 | tarbase | MIMAT0000259 | 10004 | ENSG00000168060 | Degradome sequencing | positive |  | validated |
| PAG1 | -0.257 | 5.8E-05 | tarbase | MIMAT0000259 | 55824 | ENSG00000076641 | Degradome sequencing | negative |  | validated |
| PALM2AKAP2 | -0.321 | 4.2E-07 | tarbase | MIMAT0000259 | 445815 | ENSG00000157654 | Degradome sequencing | positive |  | validated |
| PCSK1 | -0.260 | 4.8E-05 | tarbase | MIMAT0000259 | 5122 | ENSG00000175426 | Degradome sequencing | negative |  | validated |
| PLA2G4A | -0.254 | 7.1E-05 | tarbase | MIMAT0000259 | 5321 | ENSG00000116711 | Degradome sequencing | negative |  | validated |
| PLAG1 | -0.254 | 7.1E-05 | tarbase | MIMAT0000259 | 5324 | ENSG00000181690 | Degradome sequencing | negative |  | validated |
| PLEKHM1 | -0.308 | 1.3E-06 | tarbase | MIMAT0000259 | 9842 | ENSG00000225190 | Degradome sequencing | positive |  | validated |
| PLEKHO1 | -0.251 | 8.7E-05 | tarbase | MIMAT0000259 | 51177 | ENSG00000023902 | Degradome sequencing | negative |  | validated |
| PPM1K | -0.260 | 4.8E-05 | tarbase | MIMAT0000259 | 152926 | ENSG00000163644 | Degradome sequencing | positive |  | validated |
| PPP1R9B | -0.251 | 9.0E-05 | tarbase | MIMAT0000259 | 84687 | ENSG00000108819 | Degradome sequencing | positive |  | validated |
| PROS1 | -0.279 | 1.2E-05 | tarbase | MIMAT0000259 | 5627 | ENSG00000184500 | Degradome sequencing | negative |  | validated |
| PRSS12 | -0.255 | 7.0E-05 | tarbase | MIMAT0000259 | 8492 | ENSG00000164099 | Degradome sequencing | negative |  | validated |
| RAB3IL1 | -0.338 | 9.7E-08 | tarbase | MIMAT0000259 | 5866 | ENSG00000167994 | Degradome sequencing | positive |  | validated |
| RENBP | -0.345 | 5.0E-08 | tarbase | MIMAT0000259 | 5973 | ENSG00000102032 | Degradome sequencing | positive |  | validated |
| RGS2 | -0.285 | 8.2E-06 | mirtarbase | MIMAT0000259 | 5997 | ENSG00000116741 | HITS-CLIP | Functional MTI (Weak) | 28735896 | validated |
| RGS2 | -0.285 | 8.2E-06 | tarbase | MIMAT0000259 | 5997 | ENSG00000116741 | Degradome sequencing | positive |  | validated |
| RGS2 | -0.285 | 8.2E-06 | tarbase | MIMAT0000259 | 5997 | ENSG00000116741 | Degradome sequencing | negative |  | validated |
| RHOG | -0.254 | 7.4E-05 | tarbase | MIMAT0000259 | 391 | ENSG00000177105 | Degradome sequencing | positive |  | validated |
| RIPK3 | -0.256 | 6.5E-05 | tarbase | MIMAT0000259 | 11035 | ENSG00000129465 | Degradome sequencing | positive |  | validated |
| RNF175 | -0.348 | 3.9E-08 | tarbase | MIMAT0000259 | 285533 | ENSG00000145428 | Degradome sequencing | negative |  | validated |
| SELPLG | -0.330 | 2.0E-07 | tarbase | MIMAT0000259 | 6404 | ENSG00000110876 | Degradome sequencing | positive |  | validated |
| SH3KBP1 | -0.351 | 3.0E-08 | tarbase | MIMAT0000259 | 30011 | ENSG00000147010 | Degradome sequencing | positive |  | validated |
| SLC6A12 | -0.268 | 2.8E-05 | tarbase | MIMAT0000259 | 6539 | ENSG00000111181 | Degradome sequencing | positive |  | validated |
| SNAI2 | -0.274 | 1.7E-05 | mirtarbase | MIMAT0000259 | 6591 | ENSG00000019549 | Luciferase reporter assay | Functional MTI | 23354685 | validated |
| SNAI2 | -0.274 | 1.7E-05 | tarbase | MIMAT0000259 | 6591 | ENSG00000019549 | Degradome sequencing | positive |  | validated |
| SNAI2 | -0.274 | 1.7E-05 | tarbase | MIMAT0000259 | 6591 | ENSG00000019549 | Degradome sequencing | negative |  | validated |
| SPATA8 | -0.316 | 5.7E-07 | tarbase | MIMAT0000259 |  | ENSG00000185594 | Degradome sequencing | positive |  | validated |
| SPNS3 | -0.280 | 1.0E-05 | tarbase | MIMAT0000259 | 201305 | ENSG00000182557 | Degradome sequencing | positive |  | validated |
| SRF | -0.260 | 4.7E-05 | tarbase | MIMAT0000259 | 6722 | ENSG00000112658 | Degradome sequencing//Degradome sequencing | positive |  | validated |
| SRGN | -0.273 | 2.0E-05 | tarbase | MIMAT0000259 | 5552 | ENSG00000122862 | Degradome sequencing | positive |  | validated |
| ST8SIA4 | -0.257 | 5.8E-05 | tarbase | MIMAT0000259 | 7903 | ENSG00000113532 | Degradome sequencing | negative |  | validated |
| SYNM | -0.303 | 1.9E-06 | mirtarbase | MIMAT0000259 | 23336 | ENSG00000182253 | PAR-CLIP | Functional MTI (Weak) | 22012620 | validated |
| TAB1 | -0.257 | 6.1E-05 | tarbase | MIMAT0000259 | 10454 | ENSG00000100324 | Degradome sequencing | positive |  | validated |
| TBC1D10C | -0.304 | 1.8E-06 | tarbase | MIMAT0000259 | 374403 | ENSG00000175463 | Degradome sequencing | positive |  | validated |
| TBX21 | -0.307 | 1.5E-06 | tarbase | MIMAT0000259 | 30009 | ENSG00000073861 | Degradome sequencing | positive |  | validated |
| TCF25 | -0.255 | 7.0E-05 | tarbase | MIMAT0000259 | 22980 | ENSG00000141002 | Degradome sequencing | negative |  | validated |
| TLN1 | -0.259 | 5.2E-05 | tarbase | MIMAT0000259 | 7094 | ENSG00000137076 | Degradome sequencing | positive |  | validated |
| TOX | -0.304 | 1.6E-06 | tarbase | MIMAT0000259 | 9760 | ENSG00000198846 | Degradome sequencing | negative |  | validated |
| TREML1 | -0.264 | 3.4E-05 | tarbase | MIMAT0000259 | 340205 | ENSG00000161911 | Degradome sequencing | positive |  | validated |
| TREML2 | -0.259 | 4.9E-05 | tarbase | MIMAT0000259 | 79865 | ENSG00000112195 | Degradome sequencing | positive |  | validated |
| TSPAN4 | -0.397 | 2.4E-10 | tarbase | MIMAT0000259 | 7106 | ENSG00000214063 | Degradome sequencing | positive |  | validated |
| UBL4B | -0.261 | 4.1E-05 | tarbase | MIMAT0000259 | 164153 | ENSG00000186150 | Degradome sequencing | positive |  | validated |
| VAMP2 | -0.254 | 7.2E-05 | tarbase | MIMAT0000259 | 6844 | ENSG00000220205 | Degradome sequencing | positive |  | validated |
| VCAM1 | -0.262 | 4.3E-05 | tarbase | MIMAT0000259 | 7412 | ENSG00000162692 | Degradome sequencing | negative |  | validated |
| VIM | -0.277 | 1.5E-05 | tarbase | MIMAT0000259 | 7431 | ENSG00000026025 | Degradome sequencing | positive |  | validated |
| VIM | -0.277 | 1.5E-05 | tarbase | MIMAT0000259 | 7431 | ENSG00000026025 | Degradome sequencing | negative |  | validated |
| ZNF831 | -0.253 | 7.1E-05 | mirtarbase | MIMAT0000259 | 128611 | ENSG00000124203 | HITS-CLIP | Functional MTI (Weak) | 23824327 | validated |
| CDCA7L | -0.257 | 6.0E-05 | tarbase | MIMAT0000459 | 55536 | ENSG00000164649 | Degradome sequencing | positive |  | validated |
| GBA2 | -0.275 | 1.6E-05 | tarbase | MIMAT0000459 | 57704 | ENSG00000070610 | Degradome sequencing | positive |  | validated |
| STMN1 | -0.276 | 1.5E-05 | tarbase | MIMAT0000459 | 3925 | ENSG00000117632 | Degradome sequencing//Degradome sequencing | positive |  | validated |
| ABAT | -0.281 | 1.1E-05 | tarbase | MIMAT0000267 | 18 | ENSG00000183044 | Degradome sequencing | positive |  | validated |
| ABCA6 | -0.471 | 0.0E+00 | tarbase | MIMAT0000267 | 23460 | ENSG00000154262 | Degradome sequencing | positive |  | validated |
| ABLIM3 | -0.347 | 4.2E-08 | tarbase | MIMAT0000267 | 22885 | ENSG00000173210 | Degradome sequencing | positive |  | validated |
| ADH1A | -0.348 | 3.1E-08 | tarbase | MIMAT0000267 | 124 | ENSG00000187758 | Degradome sequencing | positive |  | validated |
| ADH1C | -0.426 | 5.3E-12 | tarbase | MIMAT0000267 | 126 | ENSG00000248144 | Degradome sequencing | positive |  | validated |
| ADIRF | -0.267 | 2.9E-05 | tarbase | MIMAT0000267 | 10974 | ENSG00000148671 | Degradome sequencing | positive |  | validated |
| ADRA2A | -0.413 | 3.6E-11 | tarbase | MIMAT0000267 | 150 | ENSG00000150594 | Degradome sequencing | positive |  | validated |
| AIF1 | -0.283 | 9.1E-06 | tarbase | MIMAT0000267 | 199 | ENSG00000204472 | Degradome sequencing | positive |  | validated |
| ALDH16A1 | -0.333 | 1.5E-07 | tarbase | MIMAT0000267 | 126133 | ENSG00000161618 | Degradome sequencing | positive |  | validated |
| ALDH1A1 | -0.388 | 6.3E-10 | tarbase | MIMAT0000267 | 216 | ENSG00000165092 | Degradome sequencing//Degradome sequencing | positive |  | validated |
| ALG13 | -0.259 | 5.1E-05 | tarbase | MIMAT0000267 | 79868 | ENSG00000101901 | Degradome sequencing | negative |  | validated |
| ANKRA2 | -0.348 | 3.9E-08 | tarbase | MIMAT0000267 | 57763 | ENSG00000164331 | Degradome sequencing | negative |  | validated |
| ARHGEF3 | -0.299 | 2.7E-06 | tarbase | MIMAT0000267 | 50650 | ENSG00000163947 | Degradome sequencing | negative |  | validated |
| ARL15 | -0.275 | 1.7E-05 | tarbase | MIMAT0000267 | 54622 | ENSG00000185305 | Degradome sequencing | positive |  | validated |
| ASAH1 | -0.255 | 6.8E-05 | tarbase | MIMAT0000267 | 427 | ENSG00000104763 | Degradome sequencing | positive |  | validated |
| BICRA | -0.287 | 6.7E-06 | tarbase | MIMAT0000267 | 29998 | ENSG00000063169 | Degradome sequencing | positive |  | validated |
| BORCS7 | -0.401 | 1.5E-10 | tarbase | MIMAT0000267 | 119032 | ENSG00000166275 | Degradome sequencing | positive |  | validated |
| BTG2 | -0.350 | 3.0E-08 | tarbase | MIMAT0000267 | 7832 | ENSG00000159388 | Degradome sequencing | positive |  | validated |
| BTK | -0.294 | 4.1E-06 | mirtarbase | MIMAT0000267 | 695 | ENSG00000010671 | ChIP-seq//Immunoblot//Luciferase reporter assay//Western blot | Functional MTI | 27756747 | validated |
| CALCRL | -0.336 | 1.2E-07 | tarbase | MIMAT0000267 | 10203 | ENSG00000064989 | Degradome sequencing | positive |  | validated |
| CAV1 | -0.290 | 5.6E-06 | tarbase | MIMAT0000267 | 857 | ENSG00000105974 | Degradome sequencing//Degradome sequencing | positive |  | validated |
| CCDC80 | -0.316 | 6.9E-07 | tarbase | MIMAT0000267 | 151887 | ENSG00000091986 | Degradome sequencing | positive |  | validated |
| CCND2 | -0.319 | 5.2E-07 | tarbase | MIMAT0000267 | 894 | ENSG00000118971 | Degradome sequencing | positive |  | validated |
| CCNDBP1 | -0.273 | 1.9E-05 | tarbase | MIMAT0000267 | 23582 | ENSG00000166946 | Degradome sequencing | positive |  | validated |
| CCNH | -0.277 | 1.4E-05 | tarbase | MIMAT0000267 | 902 | ENSG00000134480 | Degradome sequencing | positive |  | validated |
| CCNH | -0.277 | 1.4E-05 | tarbase | MIMAT0000267 | 902 | ENSG00000134480 | Degradome sequencing | negative |  | validated |
| CCRL2 | -0.266 | 3.2E-05 | tarbase | MIMAT0000267 | 9034 | ENSG00000121797 | Degradome sequencing | negative |  | validated |
| CD8A | -0.307 | 1.5E-06 | tarbase | MIMAT0000267 | 925 | ENSG00000153563 | Degradome sequencing | positive |  | validated |
| CDH17 | -0.380 | 1.2E-09 | tarbase | MIMAT0000267 | 1015 | ENSG00000079112 | Degradome sequencing//Degradome sequencing | positive |  | validated |
| CEBPA | -0.277 | 1.4E-05 | tarbase | MIMAT0000267 | 1050 | ENSG00000245848 | Degradome sequencing | positive |  | validated |
| CERT1 | -0.254 | 7.3E-05 | tarbase | MIMAT0000267 | 10087 | ENSG00000113163 | Degradome sequencing//Degradome sequencing//Degradome sequencing | positive |  | validated |
| CFD | -0.376 | 2.3E-09 | tarbase | MIMAT0000267 | 1675 | ENSG00000197766 | Degradome sequencing | positive |  | validated |
| CHST7 | -0.402 | 1.3E-10 | tarbase | MIMAT0000267 | 56548 | ENSG00000147119 | Degradome sequencing | negative |  | validated |
| CIDEC | -0.329 | 1.8E-07 | tarbase | MIMAT0000267 | 63924 | ENSG00000187288 | Degradome sequencing | positive |  | validated |
| COL17A1 | -0.282 | 9.0E-06 | tarbase | MIMAT0000267 | 1308 | ENSG00000065618 | Degradome sequencing | positive |  | validated |
| CPVL | -0.256 | 6.3E-05 | tarbase | MIMAT0000267 | 54504 | ENSG00000106066 | Degradome sequencing//Degradome sequencing | positive |  | validated |
| CROT | -0.299 | 2.6E-06 | tarbase | MIMAT0000267 | 54677 | ENSG00000005469 | Degradome sequencing | positive |  | validated |
| CSF2RA | -0.272 | 2.0E-05 | tarbase | MIMAT0000267 | 1438 | ENSG00000198223 | Degradome sequencing | positive |  | validated |
| CSRNP1 | -0.280 | 1.2E-05 | tarbase | MIMAT0000267 | 64651 | ENSG00000144655 | Degradome sequencing | negative |  | validated |
| CST3 | -0.433 | 2.4E-12 | tarbase | MIMAT0000267 | 1471 | ENSG00000101439 | Degradome sequencing | positive |  | validated |
| CTSH | -0.293 | 4.4E-06 | tarbase | MIMAT0000267 | 1512 | ENSG00000103811 | Degradome sequencing | positive |  | validated |
| CYB5D2 | -0.284 | 8.5E-06 | tarbase | MIMAT0000267 | 124936 | ENSG00000167740 | Degradome sequencing | negative |  | validated |
| CYP4F12 | -0.260 | 4.6E-05 | tarbase | MIMAT0000267 | 66002 | ENSG00000186204 | Degradome sequencing | positive |  | validated |
| DGKA | -0.291 | 5.1E-06 | tarbase | MIMAT0000267 | 1606 | ENSG00000065357 | Degradome sequencing | positive |  | validated |
| DIPK1A | -0.276 | 1.5E-05 | tarbase | MIMAT0000267 | 388650 | ENSG00000154511 | Degradome sequencing//Degradome sequencing | negative |  | validated |
| EMCN | -0.534 | 0.0E+00 | tarbase | MIMAT0000267 | 51705 | ENSG00000164035 | Degradome sequencing | positive |  | validated |
| ERO1B | -0.342 | 6.6E-08 | tarbase | MIMAT0000267 | 56605 | ENSG00000086619 | Degradome sequencing | negative |  | validated |
| FAM13B | -0.252 | 8.5E-05 | tarbase | MIMAT0000267 | 51306 | ENSG00000031003 | Degradome sequencing | positive |  | validated |
| FAM214A | -0.272 | 2.0E-05 | tarbase | MIMAT0000267 | 56204 | ENSG00000047346 | Degradome sequencing//Degradome sequencing | positive |  | validated |
| FBXL8 | -0.278 | 1.3E-05 | tarbase | MIMAT0000267 | 55336 | ENSG00000135722 | Degradome sequencing//Degradome sequencing | negative |  | validated |
| FBXO31 | -0.272 | 2.0E-05 | tarbase | MIMAT0000267 | 79791 | ENSG00000103264 | Degradome sequencing | positive |  | validated |
| FDXACB1 | -0.257 | 5.8E-05 | tarbase | MIMAT0000267 | 91893 | ENSG00000255561 | Degradome sequencing | negative |  | validated |
| FGF18 | -0.411 | 3.5E-11 | tarbase | MIMAT0000267 | 8817 | ENSG00000156427 | Degradome sequencing | positive |  | validated |
| FGL2 | -0.348 | 3.9E-08 | tarbase | MIMAT0000267 | 10875 | ENSG00000127951 | Degradome sequencing | negative |  | validated |
| FHL1 | -0.331 | 1.8E-07 | tarbase | MIMAT0000267 | 2273 | ENSG00000022267 | Degradome sequencing | negative |  | validated |
| FLT4 | -0.335 | 1.3E-07 | tarbase | MIMAT0000267 | 2324 | ENSG00000037280 | Degradome sequencing | positive |  | validated |
| FOXO1 | -0.287 | 7.0E-06 | tarbase | MIMAT0000267 | 2308 | ENSG00000150907 | Degradome sequencing | negative |  | validated |
| FOXP3 | -0.253 | 7.7E-05 | mirtarbase | MIMAT0000267 | 50943 | ENSG00000049768 | Luciferase reporter assay//qRT-PCR//Western blot | Functional MTI | 24316592 | validated |
| FYN | -0.265 | 3.4E-05 | tarbase | MIMAT0000267 | 2534 | ENSG00000010810 | Degradome sequencing | positive |  | validated |
| GASK1B | -0.287 | 6.9E-06 | tarbase | MIMAT0000267 | 51313 | ENSG00000164125 | Degradome sequencing | positive |  | validated |
| GIPC2 | -0.423 | 1.1E-11 | tarbase | MIMAT0000267 | 54810 | ENSG00000137960 | Degradome sequencing | positive |  | validated |
| GIT2 | -0.265 | 3.3E-05 | mirtarbase | MIMAT0000267 | 9815 | ENSG00000139436 | HITS-CLIP | Functional MTI (Weak) | 22473208 | validated |
| GIT2 | -0.265 | 3.3E-05 | tarbase | MIMAT0000267 | 9815 | ENSG00000139436 | Degradome sequencing | positive |  | validated |
| GLYCTK | -0.340 | 8.4E-08 | tarbase | MIMAT0000267 | 132158 | ENSG00000168237 | Degradome sequencing | positive |  | validated |
| GNPTG | -0.281 | 1.1E-05 | tarbase | MIMAT0000267 | 84572 | ENSG00000090581 | Degradome sequencing | positive |  | validated |
| HLA-DMA | -0.251 | 9.0E-05 | tarbase | MIMAT0000267 | 3108 | ENSG00000204257 | Degradome sequencing | positive |  | validated |
| HPGD | -0.307 | 1.3E-06 | tarbase | MIMAT0000267 | 3248 | ENSG00000164120 | Degradome sequencing//Degradome sequencing | positive |  | validated |
| HYAL1 | -0.307 | 1.4E-06 | tarbase | MIMAT0000267 | 3373 | ENSG00000114378 | Degradome sequencing | positive |  | validated |
| IGF1 | -0.541 | 0.0E+00 | tarbase | MIMAT0000267 | 3479 | ENSG00000017427 | Degradome sequencing | positive |  | validated |
| IGF2 | -0.314 | 7.9E-07 | tarbase | MIMAT0000267 | 3481 | ENSG00000167244 | Degradome sequencing//Degradome sequencing | positive |  | validated |
| IGFBP7 | -0.285 | 7.8E-06 | tarbase | MIMAT0000267 | 3490 | ENSG00000163453 | Degradome sequencing | positive |  | validated |
| IL10 | -0.259 | 5.2E-05 | tarbase | MIMAT0000267 | 3586 | ENSG00000136634 | Degradome sequencing | negative |  | validated |
| IL17D | -0.333 | 1.2E-07 | tarbase | MIMAT0000267 | 53342 | ENSG00000172458 | Degradome sequencing//Degradome sequencing | positive |  | validated |
| IL1R1 | -0.300 | 2.6E-06 | tarbase | MIMAT0000267 | 3554 | ENSG00000115594 | Degradome sequencing | positive |  | validated |
| IRF7 | -0.252 | 8.5E-05 | tarbase | MIMAT0000267 | 3665 | ENSG00000185507 | Degradome sequencing | negative |  | validated |
| ISCU | -0.571 | 0.0E+00 | mirtarbase | MIMAT0000267 | 23479 | ENSG00000136003 | immunoprecipitaion//Microarray//qRT-PCR | Functional MTI (Weak) | 19826008 | validated |
| ISCU | -0.571 | 0.0E+00 | mirtarbase | MIMAT0000267 | 23479 | ENSG00000136003 | Luciferase reporter assay//qRT-PCR//Western blot | Functional MTI | 19808020 | validated |
| ISCU | -0.571 | 0.0E+00 | mirtarbase | MIMAT0000267 | 23479 | ENSG00000136003 | Luciferase reporter assay//Western blot | Functional MTI | 21801864 | validated |
| ISCU | -0.571 | 0.0E+00 | mirtarbase | MIMAT0000267 | 23479 | ENSG00000136003 | CLASH | Functional MTI (Weak) | 23622248 | validated |
| ISCU | -0.571 | 0.0E+00 | mirtarbase | MIMAT0000267 | 23479 | ENSG00000136003 | qRT-PCR | Functional MTI (Weak) | 22840297 | validated |
| ISCU | -0.571 | 0.0E+00 | mirtarbase | MIMAT0000267 | 23479 | ENSG00000136003 | HITS-CLIP | Functional MTI (Weak) | 22473208 | validated |
| ISCU | -0.571 | 0.0E+00 | mirtarbase | MIMAT0000267 | 23479 | ENSG00000136003 | PAR-CLIP | Functional MTI (Weak) | 27292025 | validated |
| ISCU | -0.571 | 0.0E+00 | tarbase | MIMAT0000267 | 23479 | ENSG00000136003 | Degradome sequencing//Degradome sequencing//Degradome sequencing//Degradome sequencing//Degradome sequencing//Degradome sequencing//Degradome sequencing//Degrad | positive |  | validated |
| KCNK10 | -0.293 | 3.8E-06 | mirtarbase | MIMAT0000267 | 54207 | ENSG00000100433 | HITS-CLIP | Functional MTI (Weak) | 27418678 | validated |
| KLRG1 | -0.402 | 1.4E-10 | tarbase | MIMAT0000267 | 10219 | ENSG00000139187 | Degradome sequencing | negative |  | validated |
| LCK | -0.275 | 1.7E-05 | tarbase | MIMAT0000267 | 3932 | ENSG00000182866 | Degradome sequencing//Degradome sequencing | negative |  | validated |
| LIPE | -0.351 | 2.8E-08 | tarbase | MIMAT0000267 | 3991 | ENSG00000079435 | Degradome sequencing | negative |  | validated |
| LRPAP1 | -0.251 | 8.6E-05 | tarbase | MIMAT0000267 | 4043 | ENSG00000163956 | Degradome sequencing | positive |  | validated |
| MAN2B1 | -0.258 | 5.6E-05 | tarbase | MIMAT0000267 | 4125 | ENSG00000104774 | Degradome sequencing | positive |  | validated |
| MAN2B2 | -0.269 | 2.6E-05 | tarbase | MIMAT0000267 | 23324 | ENSG00000013288 | Degradome sequencing//Degradome sequencing | positive |  | validated |
| MCTP1 | -0.355 | 1.9E-08 | tarbase | MIMAT0000267 | 79772 | ENSG00000175471 | Degradome sequencing | positive |  | validated |
| MICAL1 | -0.326 | 2.9E-07 | tarbase | MIMAT0000267 | 64780 | ENSG00000135596 | Degradome sequencing | positive |  | validated |
| MIGA2 | -0.281 | 1.0E-05 | tarbase | MIMAT0000267 | 84895 | ENSG00000148343 | Degradome sequencing//Degradome sequencing | positive |  | validated |
| MMP28 | -0.297 | 3.2E-06 | tarbase | MIMAT0000267 | 79148 | ENSG00000271447 | Degradome sequencing | positive |  | validated |
| MMRN1 | -0.414 | 2.4E-11 | tarbase | MIMAT0000267 | 22915 | ENSG00000138722 | Degradome sequencing | positive |  | validated |
| NME3 | -0.256 | 6.3E-05 | tarbase | MIMAT0000267 | 4832 | ENSG00000103024 | Degradome sequencing | negative |  | validated |
| NOS3 | -0.272 | 2.2E-05 | tarbase | MIMAT0000267 | 4846 | ENSG00000164867 | Degradome sequencing | positive |  | validated |
| NOSTRIN | -0.310 | 1.1E-06 | tarbase | MIMAT0000267 | 115677 | ENSG00000163072 | Degradome sequencing | positive |  | validated |
| NUDT4 | -0.269 | 2.7E-05 | tarbase | MIMAT0000267 | 11163 | ENSG00000173598 | Degradome sequencing | negative |  | validated |
| OAZ1 | -0.258 | 5.5E-05 | tarbase | MIMAT0000267 | 4946 | ENSG00000104904 | Degradome sequencing | positive |  | validated |
| OLFML3 | -0.320 | 4.6E-07 | tarbase | MIMAT0000267 | 56944 | ENSG00000116774 | Degradome sequencing | positive |  | validated |
| OSR2 | -0.286 | 7.4E-06 | tarbase | MIMAT0000267 | 116039 | ENSG00000164920 | Degradome sequencing | positive |  | validated |
| PALD1 | -0.409 | 5.9E-11 | tarbase | MIMAT0000267 | 27143 | ENSG00000107719 | Degradome sequencing | negative |  | validated |
| PARP8 | -0.267 | 2.9E-05 | tarbase | MIMAT0000267 | 79668 | ENSG00000151883 | Degradome sequencing | positive |  | validated |
| PCSK5 | -0.253 | 7.8E-05 | tarbase | MIMAT0000267 | 5125 | ENSG00000099139 | Degradome sequencing | positive |  | validated |
| PDGFRL | -0.287 | 7.0E-06 | tarbase | MIMAT0000267 | 5157 | ENSG00000104213 | Degradome sequencing//Degradome sequencing | negative |  | validated |
| PHYKPL | -0.407 | 7.5E-11 | tarbase | MIMAT0000267 | 85007 | ENSG00000175309 | Degradome sequencing | positive |  | validated |
| PLA2G2A | -0.326 | 2.3E-07 | tarbase | MIMAT0000267 | 5320 | ENSG00000188257 | Degradome sequencing | positive |  | validated |
| PLPP3 | -0.316 | 6.8E-07 | tarbase | MIMAT0000267 | 8613 | ENSG00000162407 | Degradome sequencing//Degradome sequencing//Degradome sequencing | positive |  | validated |
| POC5 | -0.294 | 4.1E-06 | tarbase | MIMAT0000267 | 134359 | ENSG00000152359 | Degradome sequencing | positive |  | validated |
| POC5 | -0.294 | 4.1E-06 | tarbase | MIMAT0000267 | 134359 | ENSG00000152359 | Degradome sequencing | negative |  | validated |
| POLD4 | -0.252 | 8.1E-05 | tarbase | MIMAT0000267 | 57804 | ENSG00000175482 | Degradome sequencing | positive |  | validated |
| POU2F2 | -0.293 | 4.3E-06 | tarbase | MIMAT0000267 | 5452 | ENSG00000028277 | Degradome sequencing | positive |  | validated |
| PRSS35 | -0.282 | 8.9E-06 | tarbase | MIMAT0000267 | 167681 | ENSG00000146250 | Degradome sequencing | positive |  | validated |
| PSAP | -0.256 | 6.2E-05 | mirtarbase | MIMAT0000267 | 5660 | ENSG00000197746 | HITS-CLIP | Functional MTI (Weak) | 22473208 | validated |
| PSAP | -0.256 | 6.2E-05 | tarbase | MIMAT0000267 | 5660 | ENSG00000197746 | Degradome sequencing//Degradome sequencing//Degradome sequencing | positive |  | validated |
| PTPN6 | -0.288 | 6.2E-06 | tarbase | MIMAT0000267 | 5777 | ENSG00000111679 | Degradome sequencing | negative |  | validated |
| RAB8B | -0.262 | 4.1E-05 | tarbase | MIMAT0000267 | 51762 | ENSG00000166128 | Degradome sequencing | positive |  | validated |
| RAC2 | -0.285 | 8.2E-06 | tarbase | MIMAT0000267 | 5880 | ENSG00000128340 | Degradome sequencing | negative |  | validated |
| RAMP2 | -0.475 | 0.0E+00 | tarbase | MIMAT0000267 | 10266 | ENSG00000131477 | Degradome sequencing | negative |  | validated |
| RGS5 | -0.368 | 5.3E-09 | tarbase | MIMAT0000267 | 8490 | ENSG00000143248 | Degradome sequencing | positive |  | validated |
| RGS5 | -0.368 | 5.3E-09 | tarbase | MIMAT0000267 | 8490 | ENSG00000143248 | Degradome sequencing | negative |  | validated |
| RHOB | -0.274 | 1.9E-05 | tarbase | MIMAT0000267 | 388 | ENSG00000143878 | Degradome sequencing//Degradome sequencing//Degradome sequencing | positive |  | validated |
| RUNX1T1 | -0.261 | 4.2E-05 | mirtarbase | MIMAT0000267 | 862 | ENSG00000079102 | PAR-CLIP//HITS-CLIP | Functional MTI (Weak) | 21572407 | validated |
| RUNX1T1 | -0.261 | 4.2E-05 | mirtarbase | MIMAT0000267 | 862 | ENSG00000079102 | HITS-CLIP | Functional MTI (Weak) | 23313552 | validated |
| RUNX1T1 | -0.261 | 4.2E-05 | mirtarbase | MIMAT0000267 | 862 | ENSG00000079102 | HITS-CLIP | Functional MTI (Weak) | 27418678 | validated |
| SCAMP4 | -0.252 | 8.1E-05 | tarbase | MIMAT0000267 | 113178 | ENSG00000227500 | Degradome sequencing | positive |  | validated |
| SCN1B | -0.359 | 1.3E-08 | mirtarbase | MIMAT0000267 | 6324 | ENSG00000105711 | CLASH | Functional MTI (Weak) | 23622248 | validated |
| SELENOP | -0.358 | 1.4E-08 | tarbase | MIMAT0000267 | 6414 | ENSG00000250722 | Degradome sequencing | positive |  | validated |
| SIGLEC15 | -0.257 | 5.4E-05 | tarbase | MIMAT0000267 | 284266 | ENSG00000197046 | Degradome sequencing | positive |  | validated |
| SLC25A45 | -0.277 | 1.5E-05 | tarbase | MIMAT0000267 | 283130 | ENSG00000162241 | Degradome sequencing | positive |  | validated |
| SLC9A3R2 | -0.256 | 6.6E-05 | tarbase | MIMAT0000267 | 9351 | ENSG00000065054 | Degradome sequencing | negative |  | validated |
| SLCO2A1 | -0.401 | 1.6E-10 | tarbase | MIMAT0000267 | 6578 | ENSG00000174640 | Degradome sequencing | negative |  | validated |
| SMAP2 | -0.314 | 8.0E-07 | tarbase | MIMAT0000267 | 64744 | ENSG00000084070 | Degradome sequencing | positive |  | validated |
| ST6GALNAC1 | -0.295 | 3.3E-06 | tarbase | MIMAT0000267 | 55808 | ENSG00000070526 | Degradome sequencing//Degradome sequencing | positive |  | validated |
| ST6GALNAC3 | -0.304 | 1.8E-06 | tarbase | MIMAT0000267 | 256435 | ENSG00000184005 | Degradome sequencing | positive |  | validated |
| STAT4 | -0.348 | 3.9E-08 | tarbase | MIMAT0000267 | 6775 | ENSG00000138378 | Degradome sequencing | positive |  | validated |
| TBRG1 | -0.262 | 4.3E-05 | tarbase | MIMAT0000267 | 84897 | ENSG00000154144 | Degradome sequencing | positive |  | validated |
| TBX15 | -0.253 | 7.8E-05 | tarbase | MIMAT0000267 | 6913 | ENSG00000092607 | Degradome sequencing | positive |  | validated |
| TERF2IP | -0.280 | 1.1E-05 | tarbase | MIMAT0000267 | 54386 | ENSG00000166848 | Degradome sequencing//Degradome sequencing//Degradome sequencing//Degradome sequencing | positive |  | validated |
| TFF1 | -0.282 | 9.3E-06 | tarbase | MIMAT0000267 | 7031 | ENSG00000160182 | Degradome sequencing | negative |  | validated |
| TFF3 | -0.318 | 4.8E-07 | tarbase | MIMAT0000267 | 7033 | ENSG00000160180 | Degradome sequencing | positive |  | validated |
| TFPI | -0.273 | 1.9E-05 | tarbase | MIMAT0000267 | 7035 | ENSG00000003436 | Degradome sequencing | positive |  | validated |
| THSD7A | -0.283 | 9.5E-06 | mirtarbase | MIMAT0000267 | 221981 | ENSG00000005108 | Luciferase reporter assay//qRT-PCR//Western blot | Functional MTI | 26796133 | validated |
| THSD7A | -0.283 | 9.5E-06 | tarbase | MIMAT0000267 | 221981 | ENSG00000005108 | Degradome sequencing | positive |  | validated |
| TLDC2 | -0.266 | 3.1E-05 | tarbase | MIMAT0000267 | 140711 | ENSG00000101342 | Degradome sequencing | positive |  | validated |
| TLR4 | -0.259 | 5.0E-05 | tarbase | MIMAT0000267 | 7099 | ENSG00000136869 | Degradome sequencing | positive |  | validated |
| TLR4 | -0.259 | 5.0E-05 | tarbase | MIMAT0000267 | 7099 | ENSG00000136869 | Degradome sequencing | negative |  | validated |
| TMEM129 | -0.259 | 5.3E-05 | tarbase | MIMAT0000267 | 92305 | ENSG00000168936 | Degradome sequencing | positive |  | validated |
| TMEM163 | -0.251 | 9.1E-05 | tarbase | MIMAT0000267 | 81615 | ENSG00000152128 | Degradome sequencing | negative |  | validated |
| TNFAIP8 | -0.302 | 2.1E-06 | tarbase | MIMAT0000267 | 25816 | ENSG00000145779 | Degradome sequencing | negative |  | validated |
| TOX2 | -0.268 | 2.8E-05 | tarbase | MIMAT0000267 | 84969 | ENSG00000124191 | Degradome sequencing | positive |  | validated |
| TPK1 | -0.296 | 3.3E-06 | tarbase | MIMAT0000267 | 27010 | ENSG00000196511 | Degradome sequencing | negative |  | validated |
| TSC22D3 | -0.308 | 1.3E-06 | tarbase | MIMAT0000267 | 1831 | ENSG00000157514 | Degradome sequencing//Degradome sequencing | positive |  | validated |
| TXNIP | -0.329 | 2.3E-07 | tarbase | MIMAT0000267 | 10628 | ENSG00000265972 | Degradome sequencing | positive |  | validated |
| VAMP1 | -0.347 | 4.2E-08 | tarbase | MIMAT0000267 | 6843 | ENSG00000139190 | Degradome sequencing | positive |  | validated |
| VILL | -0.312 | 8.9E-07 | tarbase | MIMAT0000267 | 50853 | ENSG00000136059 | Degradome sequencing | positive |  | validated |
| ZC3H12D | -0.260 | 4.7E-05 | tarbase | MIMAT0000267 | 340152 | ENSG00000178199 | Degradome sequencing | positive |  | validated |
| ZCCHC24 | -0.442 | 6.1E-13 | tarbase | MIMAT0000267 | 219654 | ENSG00000165424 | Degradome sequencing | negative |  | validated |
| ZEB1 | -0.288 | 6.2E-06 | tarbase | MIMAT0000267 | 6935 | ENSG00000148516 | Degradome sequencing | positive |  | validated |
| ZFP36 | -0.304 | 1.9E-06 | tarbase | MIMAT0000267 | 7538 | ENSG00000128016 | Degradome sequencing | positive |  | validated |
| ZNF385A | -0.263 | 4.0E-05 | tarbase | MIMAT0000267 | 25946 | ENSG00000161642 | Degradome sequencing | positive |  | validated |
| ACACA | -0.257 | 5.8E-05 | tarbase | MIMAT0000278 | 31 | ENSG00000278540 | Degradome sequencing | positive |  | validated |
| ACSL3 | -0.262 | 4.1E-05 | mirtarbase | MIMAT0000278 | 2181 | ENSG00000123983 | Sequencing | Functional MTI (Weak) | 20371350 | validated |
| ACSL3 | -0.262 | 4.1E-05 | tarbase | MIMAT0000278 | 2181 | ENSG00000123983 | Degradome sequencing | positive |  | validated |
| ADIPOR1 | -0.387 | 7.7E-10 | tarbase | MIMAT0000278 | 51094 | ENSG00000159346 | Degradome sequencing//Degradome sequencing | positive |  | validated |
| ANKS1B | -0.394 | 2.5E-10 | tarbase | MIMAT0000278 | 56899 | ENSG00000185046 | Degradome sequencing | positive |  | validated |
| AP3M1 | -0.287 | 6.7E-06 | tarbase | MIMAT0000278 | 26985 | ENSG00000185009 | Degradome sequencing | positive |  | validated |
| ARHGAP12 | -0.254 | 7.2E-05 | tarbase | MIMAT0000278 | 94134 | ENSG00000165322 | Degradome sequencing | positive |  | validated |
| ARL6IP1 | -0.353 | 2.4E-08 | mirtarbase | MIMAT0000278 | 23204 | ENSG00000170540 | PAR-CLIP | Functional MTI (Weak) | 23592263 | validated |
| ARL6IP1 | -0.353 | 2.4E-08 | tarbase | MIMAT0000278 | 23204 | ENSG00000170540 | Degradome sequencing | positive |  | validated |
| ASPH | -0.272 | 2.1E-05 | tarbase | MIMAT0000278 | 444 | ENSG00000198363 | Degradome sequencing | positive |  | validated |
| ATP2A2 | -0.267 | 2.9E-05 | mirtarbase | MIMAT0000278 | 488 | ENSG00000174437 | CLASH | Functional MTI (Weak) | 23622248 | validated |
| ATP7A | -0.251 | 8.8E-05 | tarbase | MIMAT0000278 | 538 | ENSG00000165240 | Degradome sequencing | positive |  | validated |
| BCOR | -0.363 | 8.8E-09 | tarbase | MIMAT0000278 | 54880 | ENSG00000183337 | Degradome sequencing | positive |  | validated |
| BRWD1 | -0.279 | 1.2E-05 | mirtarbase | MIMAT0000278 | 54014 | ENSG00000185658 | HITS-CLIP | Functional MTI (Weak) | 23313552 | validated |
| BRWD1 | -0.279 | 1.2E-05 | tarbase | MIMAT0000278 | 54014 | ENSG00000185658 | Degradome sequencing//Degradome sequencing//Degradome sequencing | positive |  | validated |
| C16orf72 | -0.329 | 2.2E-07 | tarbase | MIMAT0000278 | 29035 | ENSG00000182831 | Degradome sequencing//Degradome sequencing//Degradome sequencing//Degradome sequencing | positive |  | validated |
| C18orf32 | -0.338 | 9.6E-08 | tarbase | MIMAT0000278 | 497661 | ENSG00000177576 | Degradome sequencing//Degradome sequencing//Degradome sequencing | positive |  | validated |
| C5orf51 | -0.270 | 2.5E-05 | mirtarbase | MIMAT0000278 | 285636 | ENSG00000205765 | PAR-CLIP | Functional MTI (Weak) | 23592263 | validated |
| CACNB4 | -0.261 | 4.5E-05 | tarbase | MIMAT0000278 | 785 | ENSG00000182389 | Degradome sequencing | positive |  | validated |
| CCP110 | -0.258 | 5.6E-05 | tarbase | MIMAT0000278 | 9738 | ENSG00000103540 | Degradome sequencing | positive |  | validated |
| CD164 | -0.337 | 1.0E-07 | tarbase | MIMAT0000278 | 8763 | ENSG00000135535 | Degradome sequencing//Degradome sequencing//Degradome sequencing//Degradome sequencing | positive |  | validated |
| CEP350 | -0.251 | 8.8E-05 | tarbase | MIMAT0000278 | 9857 | ENSG00000135837 | Degradome sequencing | positive |  | validated |
| CLCN3 | -0.425 | 8.2E-12 | tarbase | MIMAT0000278 | 1182 | ENSG00000109572 | Degradome sequencing | positive |  | validated |
| CPD | -0.321 | 4.5E-07 | tarbase | MIMAT0000278 | 1362 | ENSG00000108582 | Degradome sequencing | positive |  | validated |
| CSRNP2 | -0.259 | 5.2E-05 | tarbase | MIMAT0000278 | 81566 | ENSG00000110925 | Degradome sequencing | positive |  | validated |
| CSTF2T | -0.311 | 9.8E-07 | mirtarbase | MIMAT0000278 | 23283 | ENSG00000177613 | Sequencing//PAR-CLIP | Functional MTI (Weak) | 20371350 | validated |
| CSTF2T | -0.311 | 9.8E-07 | mirtarbase | MIMAT0000278 | 23283 | ENSG00000177613 | PAR-CLIP | Functional MTI (Weak) | 21572407 | validated |
| CSTF2T | -0.311 | 9.8E-07 | tarbase | MIMAT0000278 | 23283 | ENSG00000177613 | Degradome sequencing//Degradome sequencing | positive |  | validated |
| CUL3 | -0.316 | 6.7E-07 | tarbase | MIMAT0000278 | 8452 | ENSG00000036257 | Degradome sequencing | positive |  | validated |
| DDAH1 | -0.287 | 6.8E-06 | mirtarbase | MIMAT0000278 | 23576 | ENSG00000153904 | CLASH | Functional MTI (Weak) | 23622248 | validated |
| DDX6 | -0.301 | 2.3E-06 | mirtarbase | MIMAT0000278 | 1656 | ENSG00000110367 | PAR-CLIP | Functional MTI (Weak) | 23592263 | validated |
| DDX6 | -0.301 | 2.3E-06 | mirtarbase | MIMAT0000278 | 1656 | ENSG00000110367 | PAR-CLIP | Functional MTI (Weak) | 22012620 | validated |
| DDX6 | -0.301 | 2.3E-06 | mirtarbase | MIMAT0000278 | 1656 | ENSG00000110367 | PAR-CLIP | Functional MTI (Weak) | 20371350 | validated |
| EAF1 | -0.302 | 2.2E-06 | tarbase | MIMAT0000278 | 85403 | ENSG00000144597 | Degradome sequencing | positive |  | validated |
| EIF2AK1 | -0.250 | 9.4E-05 | mirtarbase | MIMAT0000278 | 27102 | ENSG00000086232 | Sequencing | Functional MTI (Weak) | 20371350 | validated |
| ERBB3 | -0.434 | 2.3E-12 | tarbase | MIMAT0000278 | 2065 | ENSG00000065361 | Degradome sequencing | positive |  | validated |
| ERBB4 | -0.317 | 5.5E-07 | mirtarbase | MIMAT0000278 | 2066 | ENSG00000178568 | Microarray | Functional MTI (Weak) | 22942087 | validated |
| ERBB4 | -0.317 | 5.5E-07 | tarbase | MIMAT0000278 | 2066 | ENSG00000178568 | Degradome sequencing | positive |  | validated |
| ERCC4 | -0.289 | 6.1E-06 | tarbase | MIMAT0000278 | 2072 | ENSG00000175595 | Degradome sequencing//Degradome sequencing | positive |  | validated |
| FAM214A | -0.311 | 1.0E-06 | tarbase | MIMAT0000278 | 56204 | ENSG00000047346 | Degradome sequencing//Degradome sequencing//Degradome sequencing//Degradome sequencing | positive |  | validated |
| FNIP2 | -0.279 | 1.3E-05 | tarbase | MIMAT0000278 | 57600 | ENSG00000052795 | Degradome sequencing//Degradome sequencing | positive |  | validated |
| FOXO3 | -0.299 | 2.7E-06 | mirtarbase | MIMAT0000278 | 2309 | ENSG00000118689 | qRT-PCR//ChIP//Luciferase reporter assay//Western blot//Northern blot | Functional MTI | 20388878 | validated |
| FOXO3 | -0.299 | 2.7E-06 | tarbase | MIMAT0000278 | 2309 | ENSG00000118689 | Degradome sequencing | positive |  | validated |
| FRK | -0.348 | 3.7E-08 | tarbase | MIMAT0000278 | 2444 | ENSG00000111816 | Degradome sequencing//Degradome sequencing | positive |  | validated |
| FRS2 | -0.326 | 2.9E-07 | tarbase | MIMAT0000278 | 10818 | ENSG00000166225 | Degradome sequencing | positive |  | validated |
| GGCT | -0.293 | 4.3E-06 | tarbase | MIMAT0000278 | 79017 | ENSG00000006625 | Degradome sequencing | positive |  | validated |
| GPR107 | -0.301 | 2.2E-06 | mirtarbase | MIMAT0000278 | 57720 | ENSG00000148358 | Sequencing | Functional MTI (Weak) | 20371350 | validated |
| GPR107 | -0.301 | 2.2E-06 | mirtarbase | MIMAT0000278 | 57720 | ENSG00000148358 | PAR-CLIP | Functional MTI (Weak) | 26701625 | validated |
| GSE1 | -0.422 | 1.2E-11 | tarbase | MIMAT0000278 | 23199 | ENSG00000131149 | Degradome sequencing | positive |  | validated |
| HECTD1 | -0.264 | 3.8E-05 | mirtarbase | MIMAT0000278 | 25831 | ENSG00000092148 | CLASH | Functional MTI (Weak) | 23622248 | validated |
| HMGXB4 | -0.365 | 7.4E-09 | mirtarbase | MIMAT0000278 | 10042 | ENSG00000100281 | Reporter assay |  |  |  |
| HMGXB4 | -0.365 | 7.4E-09 | tarbase | MIMAT0000278 | 10042 | ENSG00000100281 | Degradome sequencing | positive |  | validated |
| HOXC10 | -0.307 | 1.4E-06 | mirtarbase | MIMAT0000278 | 3226 | ENSG00000180818 | qRT-PCR | Non-Functional MTI (Weak) | 20516212 | validated |
| HOXC10 | -0.307 | 1.4E-06 | tarbase | MIMAT0000278 | 3226 | ENSG00000180818 | Degradome sequencing | negative |  | validated |
| KDM5B | -0.394 | 3.5E-10 | tarbase | MIMAT0000278 | 10765 | ENSG00000117139 | Degradome sequencing | positive |  | validated |
| KIF3B | -0.256 | 6.5E-05 | tarbase | MIMAT0000278 | 9371 | ENSG00000101350 | Degradome sequencing | positive |  | validated |
| KPNA1 | -0.283 | 9.5E-06 | tarbase | MIMAT0000278 | 3836 | ENSG00000114030 | Degradome sequencing | positive |  | validated |
| LMBR1 | -0.292 | 4.7E-06 | tarbase | MIMAT0000278 | 64327 | ENSG00000105983 | Degradome sequencing | positive |  | validated |
| LMBRD2 | -0.306 | 1.5E-06 | tarbase | MIMAT0000278 | 92255 | ENSG00000164187 | Degradome sequencing | positive |  | validated |
| LONP2 | -0.339 | 8.4E-08 | tarbase | MIMAT0000278 | 83752 | ENSG00000102910 | Degradome sequencing | positive |  | validated |
| MEGF9 | -0.367 | 5.8E-09 | tarbase | MIMAT0000278 | 1955 | ENSG00000106780 | Degradome sequencing//Degradome sequencing | positive |  | validated |
| MIA3 | -0.325 | 3.1E-07 | tarbase | MIMAT0000278 | 375056 | ENSG00000154305 | Degradome sequencing//Degradome sequencing//Degradome sequencing//Degradome sequencing | positive |  | validated |
| MIDN | -0.338 | 9.4E-08 | mirtarbase | MIMAT0000278 | 90007 | ENSG00000167470 | Sequencing//PAR-CLIP | Functional MTI (Weak) | 20371350 | validated |
| MIDN | -0.338 | 9.4E-08 | mirtarbase | MIMAT0000278 | 90007 | ENSG00000167470 | HITS-CLIP | Functional MTI (Weak) | 23313552 | validated |
| MIDN | -0.338 | 9.4E-08 | tarbase | MIMAT0000278 | 90007 | ENSG00000167470 | Degradome sequencing//Degradome sequencing//Degradome sequencing//Degradome sequencing//Degradome sequencing//Degradome sequencing//Degradome sequencing//Degrad | positive |  | validated |
| MIER3 | -0.335 | 1.3E-07 | tarbase | MIMAT0000278 | 166968 | ENSG00000155545 | Degradome sequencing | positive |  | validated |
| MTA3 | -0.257 | 5.9E-05 | tarbase | MIMAT0000278 | 57504 | ENSG00000057935 | Degradome sequencing | positive |  | validated |
| MYLIP | -0.325 | 3.0E-07 | mirtarbase | MIMAT0000278 | 29116 | ENSG00000007944 | PAR-CLIP | Functional MTI (Weak) | 23446348 | validated |
| MYLIP | -0.325 | 3.0E-07 | mirtarbase | MIMAT0000278 | 29116 | ENSG00000007944 | PAR-CLIP | Functional MTI (Weak) | 20371350 | validated |
| MYLIP | -0.325 | 3.0E-07 | tarbase | MIMAT0000278 | 29116 | ENSG00000007944 | Degradome sequencing//Degradome sequencing//Degradome sequencing//Degradome sequencing//Degradome sequencing//Degradome sequencing | positive |  | validated |
| PALB2 | -0.267 | 2.9E-05 | mirtarbase | MIMAT0000278 | 79728 | ENSG00000083093 | CLASH | Functional MTI (Weak) | 23622248 | validated |
| PDIK1L | -0.411 | 4.7E-11 | mirtarbase | MIMAT0000278 | 149420 | ENSG00000175087 | Sequencing | Functional MTI (Weak) | 20371350 | validated |
| PDIK1L | -0.411 | 4.7E-11 | mirtarbase | MIMAT0000278 | 149420 | ENSG00000175087 | PAR-CLIP | Functional MTI (Weak) | 21572407 | validated |
| PEX1 | -0.329 | 2.2E-07 | mirtarbase | MIMAT0000278 | 5189 | ENSG00000127980 | CLASH | Functional MTI (Weak) | 23622248 | validated |
| PEX19 | -0.252 | 8.5E-05 | mirtarbase | MIMAT0000278 | 5824 | ENSG00000162735 | CLASH | Functional MTI (Weak) | 23622248 | validated |
| PHF12 | -0.259 | 5.2E-05 | mirtarbase | MIMAT0000278 | 57649 | ENSG00000109118 | Sequencing | Functional MTI (Weak) | 20371350 | validated |
| PIK3R1 | -0.259 | 5.1E-05 | mirecords | MIMAT0000278 | 5295 | ENSG00000145675 | Western blot//Luciferase activity assay |  | 20505758 | validated |
| PIK3R1 | -0.259 | 5.1E-05 | mirtarbase | MIMAT0000278 | 5295 | ENSG00000145675 | Western blot | Functional MTI | 24147037 | validated |
| PIK3R1 | -0.259 | 5.1E-05 | mirtarbase | MIMAT0000278 | 5295 | ENSG00000145675 | Microarray//Next Generation Sequencing (NGS)//qRT-PCR | Functional MTI (Weak) | 25236949 | validated |
| PPP1R15B | -0.301 | 2.3E-06 | mirtarbase | MIMAT0000278 | 84919 | ENSG00000158615 | Sequencing | Functional MTI (Weak) | 20371350 | validated |
| PPP1R15B | -0.301 | 2.3E-06 | tarbase | MIMAT0000278 | 84919 | ENSG00000158615 | Degradome sequencing//Degradome sequencing//Degradome sequencing | positive |  | validated |
| PPP4R2 | -0.278 | 1.3E-05 | tarbase | MIMAT0000278 | 151987 | ENSG00000163605 | Degradome sequencing//Degradome sequencing//Degradome sequencing | positive |  | validated |
| PRKAA2 | -0.388 | 6.7E-10 | tarbase | MIMAT0000278 | 5563 | ENSG00000162409 | Degradome sequencing | positive |  | validated |
| PRR14L | -0.277 | 1.4E-05 | tarbase | MIMAT0000278 | 253143 | ENSG00000183530 | Degradome sequencing | positive |  | validated |
| RAB3A | -0.258 | 5.5E-05 | tarbase | MIMAT0000278 | 5864 | ENSG00000105649 | Degradome sequencing | positive |  | validated |
| RAB5B | -0.338 | 9.3E-08 | tarbase | MIMAT0000278 | 5869 | ENSG00000111540 | Degradome sequencing//Degradome sequencing | positive |  | validated |
| RAD50 | -0.271 | 2.2E-05 | tarbase | MIMAT0000278 | 10111 | ENSG00000113522 | Degradome sequencing | positive |  | validated |
| RANBP2 | -0.270 | 2.4E-05 | tarbase | MIMAT0000278 | 5903 | ENSG00000153201 | Degradome sequencing | positive |  | validated |
| RASEF | -0.333 | 1.3E-07 | tarbase | MIMAT0000278 | 158158 | ENSG00000165105 | Degradome sequencing | positive |  | validated |
| RGMB | -0.329 | 2.2E-07 | tarbase | MIMAT0000278 | 285704 | ENSG00000174136 | Degradome sequencing//Degradome sequencing | positive |  | validated |
| RNF103 | -0.456 | 0.0E+00 | tarbase | MIMAT0000278 | 7844 | ENSG00000239305 | Degradome sequencing | positive |  | validated |
| RNF20 | -0.266 | 3.2E-05 | mirtarbase | MIMAT0000278 | 56254 | ENSG00000155827 | Sequencing | Functional MTI (Weak) | 20371350 | validated |
| RREB1 | -0.419 | 1.8E-11 | tarbase | MIMAT0000278 | 6239 | ENSG00000124782 | Degradome sequencing | positive |  | validated |
| SCARB2 | -0.323 | 3.8E-07 | tarbase | MIMAT0000278 | 950 | ENSG00000138760 | Degradome sequencing | positive |  | validated |
| SCD | -0.299 | 2.7E-06 | tarbase | MIMAT0000278 | 6319 | ENSG00000099194 | Degradome sequencing | positive |  | validated |
| SCOC | -0.348 | 3.8E-08 | tarbase | MIMAT0000278 | 60592 | ENSG00000153130 | Degradome sequencing | positive |  | validated |
| SEC24B | -0.262 | 4.2E-05 | tarbase | MIMAT0000278 | 10427 | ENSG00000138802 | Degradome sequencing | positive |  | validated |
| SEC24C | -0.262 | 4.3E-05 | tarbase | MIMAT0000278 | 9632 | ENSG00000176986 | Degradome sequencing | positive |  | validated |
| SECISBP2L | -0.261 | 4.5E-05 | tarbase | MIMAT0000278 | 9728 | ENSG00000138593 | Degradome sequencing//Degradome sequencing//Degradome sequencing | positive |  | validated |
| SERINC1 | -0.333 | 1.5E-07 | tarbase | MIMAT0000278 | 57515 | ENSG00000111897 | Degradome sequencing//Degradome sequencing | positive |  | validated |
| SHLD2 | -0.384 | 9.8E-10 | mirtarbase | MIMAT0000278 | 54537 | ENSG00000122376 | PAR-CLIP | Functional MTI (Weak) | 20371350 | validated |
| SHLD2 | -0.384 | 9.8E-10 | mirtarbase | MIMAT0000278 | 54537 | ENSG00000122376 | PAR-CLIP | Functional MTI (Weak) | 26701625 | validated |
| SHLD2 | -0.384 | 9.8E-10 | tarbase | MIMAT0000278 | 54537 | ENSG00000122376 | Degradome sequencing//Degradome sequencing//Degradome sequencing | positive |  | validated |
| SIRT1 | -0.277 | 1.5E-05 | mirtarbase | MIMAT0000278 | 23411 | ENSG00000096717 | Luciferase reporter assay//QRTPCR//Western blot | Non-Functional MTI | 24892674 | validated |
| SLC10A7 | -0.279 | 1.2E-05 | mirtarbase | MIMAT0000278 | 84068 | ENSG00000120519 | HITS-CLIP | Functional MTI (Weak) | 23824327 | validated |
| SLC38A1 | -0.395 | 3.0E-10 | tarbase | MIMAT0000278 | 81539 | ENSG00000111371 | Degradome sequencing | positive |  | validated |
| SLC39A6 | -0.354 | 2.0E-08 | tarbase | MIMAT0000278 | 25800 | ENSG00000141424 | Degradome sequencing//Degradome sequencing | positive |  | validated |
| SYNJ2BP | -0.260 | 4.8E-05 | tarbase | MIMAT0000278 | 55333 | ENSG00000213463 | Degradome sequencing | positive |  | validated |
| TCF12 | -0.262 | 4.1E-05 | tarbase | MIMAT0000278 | 6938 | ENSG00000140262 | Degradome sequencing//Degradome sequencing | positive |  | validated |
| TFAP2A | -0.380 | 1.6E-09 | mirtarbase | MIMAT0000278 | 7020 | ENSG00000137203 | Sequencing//PAR-CLIP | Functional MTI (Weak) | 20371350 | validated |
| TFAP2A | -0.380 | 1.6E-09 | tarbase | MIMAT0000278 | 7020 | ENSG00000137203 | Degradome sequencing | positive |  | validated |
| TM9SF3 | -0.350 | 3.0E-08 | tarbase | MIMAT0000278 | 56889 | ENSG00000077147 | Degradome sequencing | positive |  | validated |
| TMBIM6 | -0.390 | 5.6E-10 | tarbase | MIMAT0000278 | 7009 | ENSG00000139644 | Degradome sequencing | positive |  | validated |
| TMEM59 | -0.258 | 5.4E-05 | tarbase | MIMAT0000278 | 9528 | ENSG00000116209 | Degradome sequencing | positive |  | validated |
| TSPAN13 | -0.396 | 2.8E-10 | mirtarbase | MIMAT0000278 | 27075 | ENSG00000106537 | PAR-CLIP | Functional MTI (Weak) | 21572407 | validated |
| TSPAN13 | -0.396 | 2.8E-10 | mirtarbase | MIMAT0000278 | 27075 | ENSG00000106537 | PAR-CLIP | Functional MTI (Weak) | 20371350 | validated |
| TSPAN13 | -0.396 | 2.8E-10 | tarbase | MIMAT0000278 | 27075 | ENSG00000106537 | Degradome sequencing//Degradome sequencing//Degradome sequencing | positive |  | validated |
| TULP4 | -0.261 | 4.4E-05 | tarbase | MIMAT0000278 | 56995 | ENSG00000130338 | Degradome sequencing | positive |  | validated |
| WAPL | -0.267 | 3.0E-05 | tarbase | MIMAT0000278 | 23063 | ENSG00000062650 | Degradome sequencing | positive |  | validated |
| YY1 | -0.253 | 8.0E-05 | mirtarbase | MIMAT0000278 | 7528 | ENSG00000100811 | CLASH | Functional MTI (Weak) | 23622248 | validated |
| ZBTB33 | -0.255 | 7.0E-05 | tarbase | MIMAT0000278 | 10009 | ENSG00000177485 | Degradome sequencing | positive |  | validated |
| ZC3H11A | -0.274 | 1.7E-05 | tarbase | MIMAT0000278 | 9877 | ENSG00000058673 | Degradome sequencing | positive |  | validated |
| ZFHX3 | -0.259 | 5.3E-05 | tarbase | MIMAT0000278 | 463 | ENSG00000140836 | Degradome sequencing//Degradome sequencing//Degradome sequencing//Degradome sequencing//Degradome sequencing//Degradome sequencing | positive |  | validated |
| ZFYVE16 | -0.319 | 5.2E-07 | tarbase | MIMAT0000278 | 9765 | ENSG00000039319 | Degradome sequencing//Degradome sequencing | positive |  | validated |
| ZNF616 | -0.431 | 3.7E-12 | tarbase | MIMAT0000278 | 90317 | ENSG00000204611 | Degradome sequencing | positive |  | validated |
| ZNF629 | -0.270 | 2.5E-05 | tarbase | MIMAT0000278 | 23361 | ENSG00000102870 | Degradome sequencing//Degradome sequencing | positive |  | validated |
| ZNF638 | -0.306 | 1.5E-06 | tarbase | MIMAT0000278 | 27332 | ENSG00000075292 | Degradome sequencing | positive |  | validated |
| ZNF652 | -0.407 | 7.5E-11 | mirtarbase | MIMAT0000278 | 22834 | ENSG00000198740 | Sequencing | Functional MTI (Weak) | 20371350 | validated |
| ZNF652 | -0.407 | 7.5E-11 | mirtarbase | MIMAT0000278 | 22834 | ENSG00000198740 | PAR-CLIP | Functional MTI (Weak) | 23446348 | validated |
| ZNF652 | -0.407 | 7.5E-11 | tarbase | MIMAT0000278 | 22834 | ENSG00000198740 | Degradome sequencing//Degradome sequencing | positive |  | validated |
| ZNF749 | -0.263 | 3.9E-05 | tarbase | MIMAT0000278 | 388567 | ENSG00000186230 | Degradome sequencing | positive |  | validated |
| ZNF91 | -0.418 | 2.0E-11 | tarbase | MIMAT0000278 | 7644 | ENSG00000167232 | Degradome sequencing//Degradome sequencing | positive |  | validated |
| AP1S1 | -0.263 | 3.9E-05 | tarbase | MIMAT0000752 | 1174 | ENSG00000106367 | Degradome sequencing | positive |  | validated |
| ARHGDIA | -0.264 | 3.6E-05 | tarbase | MIMAT0000752 | 396 | ENSG00000141522 | Degradome sequencing//Degradome sequencing | positive |  | validated |
| ARPC4 | -0.273 | 1.9E-05 | tarbase | MIMAT0000752 | 10093 | ENSG00000241553 | Degradome sequencing | positive |  | validated |
| BSG | -0.264 | 3.7E-05 | mirtarbase | MIMAT0000752 | 682 | ENSG00000172270 | CLASH | Functional MTI (Weak) | 23622248 | validated |
| C6orf47 | -0.259 | 5.2E-05 | mirtarbase | MIMAT0000752 | 57827 | ENSG00000226103 | PAR-CLIP | Functional MTI (Weak) | 22012620 | validated |
| C6orf47 | -0.259 | 5.2E-05 | mirtarbase | MIMAT0000752 | 57827 | ENSG00000203623 | PAR-CLIP | Functional MTI (Weak) | 22012620 | validated |
| C6orf47 | -0.259 | 5.2E-05 | mirtarbase | MIMAT0000752 | 57827 | ENSG00000204439 | PAR-CLIP | Functional MTI (Weak) | 22012620 | validated |
| C6orf47 | -0.259 | 5.2E-05 | mirtarbase | MIMAT0000752 | 57827 | ENSG00000228177 | PAR-CLIP | Functional MTI (Weak) | 22012620 | validated |
| C6orf47 | -0.259 | 5.2E-05 | mirtarbase | MIMAT0000752 | 57827 | ENSG00000226531 | PAR-CLIP | Functional MTI (Weak) | 22012620 | validated |
| C6orf47 | -0.259 | 5.2E-05 | mirtarbase | MIMAT0000752 | 57827 | ENSG00000235360 | PAR-CLIP | Functional MTI (Weak) | 22012620 | validated |
| C6orf47 | -0.259 | 5.2E-05 | mirtarbase | MIMAT0000752 | 57827 | ENSG00000228435 | PAR-CLIP | Functional MTI (Weak) | 22012620 | validated |
| CD2BP2 | -0.345 | 4.9E-08 | tarbase | MIMAT0000752 | 10421 | ENSG00000169217 | Degradome sequencing | positive |  | validated |
| CFL1 | -0.259 | 5.0E-05 | tarbase | MIMAT0000752 | 1072 | ENSG00000172757 | Degradome sequencing | positive |  | validated |
| COPS6 | -0.251 | 9.0E-05 | mirtarbase | MIMAT0000752 | 10980 | ENSG00000168090 | CLASH | Functional MTI (Weak) | 23622248 | validated |
| COX7C | -0.251 | 8.7E-05 | tarbase | MIMAT0000752 | 1350 | ENSG00000127184 | Degradome sequencing | positive |  | validated |
| DMPK | -0.325 | 3.0E-07 | tarbase | MIMAT0000752 | 1760 | ENSG00000104936 | Degradome sequencing//Degradome sequencing | positive |  | validated |
| EWSR1 | -0.299 | 2.6E-06 | mirtarbase | MIMAT0000752 | 2130 | ENSG00000182944 | CLASH | Functional MTI (Weak) | 23622248 | validated |
| EWSR1 | -0.299 | 2.6E-06 | tarbase | MIMAT0000752 | 2130 | ENSG00000182944 | Degradome sequencing | positive |  | validated |
| FAM193B | -0.306 | 1.5E-06 | mirtarbase | MIMAT0000752 | 54540 | ENSG00000146067 | PAR-CLIP | Functional MTI (Weak) | 27292025 | validated |
| FDXR | -0.254 | 7.4E-05 | tarbase | MIMAT0000752 | 2232 | ENSG00000161513 | Degradome sequencing | positive |  | validated |
| FKBP8 | -0.269 | 2.5E-05 | tarbase | MIMAT0000752 | 23770 | ENSG00000105701 | Degradome sequencing//Degradome sequencing//Degradome sequencing | positive |  | validated |
| FUS | -0.303 | 2.0E-06 | tarbase | MIMAT0000752 | 2521 | ENSG00000089280 | Degradome sequencing | positive |  | validated |
| GMPPB | -0.305 | 1.7E-06 | mirtarbase | MIMAT0000752 | 29925 | ENSG00000173540 | CLASH | Functional MTI (Weak) | 23622248 | validated |
| GTF2F1 | -0.400 | 1.8E-10 | tarbase | MIMAT0000752 | 2962 | ENSG00000125651 | Degradome sequencing | positive |  | validated |
| HEXIM1 | -0.280 | 1.2E-05 | tarbase | MIMAT0000752 | 10614 | ENSG00000186834 | Degradome sequencing | positive |  | validated |
| KRT19 | -0.295 | 3.8E-06 | tarbase | MIMAT0000752 | 3880 | ENSG00000171345 | Degradome sequencing | positive |  | validated |
| LENG9 | -0.318 | 5.4E-07 | mirtarbase | MIMAT0000752 | 94059 | ENSG00000273574 | HITS-CLIP | Functional MTI (Weak) | 23824327 | validated |
| LENG9 | -0.318 | 5.4E-07 | mirtarbase | MIMAT0000752 | 94059 | ENSG00000275183 | HITS-CLIP | Functional MTI (Weak) | 23824327 | validated |
| LENG9 | -0.318 | 5.4E-07 | mirtarbase | MIMAT0000752 | 94059 | ENSG00000278312 | HITS-CLIP | Functional MTI (Weak) | 23824327 | validated |
| LENG9 | -0.318 | 5.4E-07 | mirtarbase | MIMAT0000752 | 94059 | ENSG00000274495 | HITS-CLIP | Functional MTI (Weak) | 23824327 | validated |
| MBD3 | -0.375 | 2.7E-09 | tarbase | MIMAT0000752 | 53615 | ENSG00000071655 | Degradome sequencing | positive |  | validated |
| NELFB | -0.319 | 5.1E-07 | tarbase | MIMAT0000752 | 25920 | ENSG00000188986 | Degradome sequencing | positive |  | validated |
| P2RY11 | -0.292 | 4.2E-06 | tarbase | MIMAT0000752 | 5032 | ENSG00000244165 | Degradome sequencing//Degradome sequencing | positive |  | validated |
| PSMC3 | -0.323 | 3.6E-07 | tarbase | MIMAT0000752 | 5702 | ENSG00000165916 | Degradome sequencing | positive |  | validated |
| PTPA | -0.371 | 4.0E-09 | tarbase | MIMAT0000752 | 5524 | ENSG00000119383 | Degradome sequencing | positive |  | validated |
| RACK1 | -0.295 | 3.6E-06 | tarbase | MIMAT0000752 | 10399 | ENSG00000204628 | Degradome sequencing | positive |  | validated |
| RBM4 | -0.253 | 7.9E-05 | tarbase | MIMAT0000752 | 5936 | ENSG00000173933 | Degradome sequencing | positive |  | validated |
| RPL32 | -0.273 | 1.9E-05 | tarbase | MIMAT0000752 | 6161 | ENSG00000144713 | Degradome sequencing | positive |  | validated |
| RPL36AL | -0.358 | 1.5E-08 | tarbase | MIMAT0000752 | 6166 | ENSG00000165502 | Degradome sequencing | positive |  | validated |
| RPS14 | -0.313 | 8.2E-07 | tarbase | MIMAT0000752 | 6208 | ENSG00000164587 | Degradome sequencing | positive |  | validated |
| RPS21 | -0.260 | 4.8E-05 | tarbase | MIMAT0000752 | 6227 | ENSG00000171858 | Degradome sequencing | positive |  | validated |
| RPS9 | -0.383 | 1.2E-09 | mirtarbase | MIMAT0000752 | 6203 | ENSG00000278270 | CLASH | Functional MTI (Weak) | 23622248 | validated |
| RPS9 | -0.383 | 1.2E-09 | mirtarbase | MIMAT0000752 | 6203 | ENSG00000170889 | CLASH | Functional MTI (Weak) | 23622248 | validated |
| RPS9 | -0.383 | 1.2E-09 | mirtarbase | MIMAT0000752 | 6203 | ENSG00000274646 | CLASH | Functional MTI (Weak) | 23622248 | validated |
| RPS9 | -0.383 | 1.2E-09 | mirtarbase | MIMAT0000752 | 6203 | ENSG00000274950 | CLASH | Functional MTI (Weak) | 23622248 | validated |
| RPS9 | -0.383 | 1.2E-09 | mirtarbase | MIMAT0000752 | 6203 | ENSG00000277359 | CLASH | Functional MTI (Weak) | 23622248 | validated |
| RPS9 | -0.383 | 1.2E-09 | mirtarbase | MIMAT0000752 | 6203 | ENSG00000275323 | CLASH | Functional MTI (Weak) | 23622248 | validated |
| RPS9 | -0.383 | 1.2E-09 | mirtarbase | MIMAT0000752 | 6203 | ENSG00000274005 | CLASH | Functional MTI (Weak) | 23622248 | validated |
| RPS9 | -0.383 | 1.2E-09 | mirtarbase | MIMAT0000752 | 6203 | ENSG00000274626 | CLASH | Functional MTI (Weak) | 23622248 | validated |
| RPS9 | -0.383 | 1.2E-09 | mirtarbase | MIMAT0000752 | 6203 | ENSG00000277079 | CLASH | Functional MTI (Weak) | 23622248 | validated |
| RPS9 | -0.383 | 1.2E-09 | mirtarbase | MIMAT0000752 | 6203 | ENSG00000278081 | CLASH | Functional MTI (Weak) | 23622248 | validated |
| SBNO2 | -0.255 | 6.7E-05 | tarbase | MIMAT0000752 | 22904 | ENSG00000064932 | Degradome sequencing | positive |  | validated |
| SNAPC4 | -0.283 | 9.2E-06 | tarbase | MIMAT0000752 | 6621 | ENSG00000165684 | Degradome sequencing | positive |  | validated |
| SQSTM1 | -0.254 | 7.4E-05 | tarbase | MIMAT0000752 | 8878 | ENSG00000161011 | Degradome sequencing | positive |  | validated |
| SURF6 | -0.299 | 2.6E-06 | tarbase | MIMAT0000752 | 6838 | ENSG00000148296 | Degradome sequencing | positive |  | validated |
| TCEAL5 | -0.273 | 2.0E-05 | tarbase | MIMAT0000752 | 340543 | ENSG00000204065 | Degradome sequencing | positive |  | validated |
| TRIM11 | -0.284 | 8.4E-06 | tarbase | MIMAT0000752 | 81559 | ENSG00000154370 | Degradome sequencing | positive |  | validated |
| TRIM28 | -0.253 | 7.6E-05 | tarbase | MIMAT0000752 | 10155 | ENSG00000130726 | Degradome sequencing | positive |  | validated |
| TRIM41 | -0.273 | 1.9E-05 | tarbase | MIMAT0000752 | 90933 | ENSG00000146063 | Degradome sequencing | positive |  | validated |
| TRIR | -0.345 | 4.9E-08 | tarbase | MIMAT0000752 | 79002 | ENSG00000123144 | Degradome sequencing | positive |  | validated |
| XRCC1 | -0.264 | 3.6E-05 | tarbase | MIMAT0000752 | 7515 | ENSG00000073050 | Degradome sequencing | positive |  | validated |
| ZBTB48 | -0.251 | 9.1E-05 | tarbase | MIMAT0000752 | 3104 | ENSG00000204859 | Degradome sequencing | positive |  | validated |
| GNAQ | -0.263 | 3.9E-05 | tarbase | MIMAT0000751 | 2776 | ENSG00000156052 | Degradome sequencing | negative |  | validated |
| MIDN | -0.305 | 1.6E-06 | tarbase | MIMAT0000751 | 90007 | ENSG00000167470 | Degradome sequencing | positive |  | validated |
| RGS5 | -0.276 | 1.6E-05 | tarbase | MIMAT0000751 | 8490 | ENSG00000143248 | Degradome sequencing | positive |  | validated |
| CBX3 | -0.257 | 5.6E-05 | tarbase | MIMAT0002817 | 11335 | ENSG00000122565 | Degradome sequencing | positive |  | validated |
| CDK2AP1 | -0.251 | 8.7E-05 | tarbase | MIMAT0002817 | 8099 | ENSG00000111328 | Degradome sequencing | positive |  | validated |
| CNBP | -0.286 | 6.7E-06 | mirtarbase | MIMAT0002817 | 7555 | ENSG00000169714 | HITS-CLIP | Functional MTI (Weak) | 23313552 | validated |
| CNBP | -0.286 | 6.7E-06 | tarbase | MIMAT0002817 | 7555 | ENSG00000169714 | Degradome sequencing | positive |  | validated |
| ELAVL1 | -0.305 | 1.5E-06 | tarbase | MIMAT0002817 | 1994 | ENSG00000066044 | Degradome sequencing | positive |  | validated |
| FANCL | -0.279 | 1.2E-05 | tarbase | MIMAT0002817 | 55120 | ENSG00000115392 | Degradome sequencing | positive |  | validated |
| FOXC1 | -0.281 | 1.0E-05 | mirtarbase | MIMAT0002817 | 2296 | ENSG00000054598 | PAR-CLIP | Functional MTI (Weak) | 20371350 | validated |
| FOXC1 | -0.281 | 1.0E-05 | mirtarbase | MIMAT0002817 | 2296 | ENSG00000054598 | GFP reporter assay//qRT-PCR//Western blot | Functional MTI | 26198045 | validated |
| TC2N | -0.251 | 8.7E-05 | tarbase | MIMAT0002852 | 123036 | ENSG00000165929 | Degradome sequencing | negative |  | validated |
| CDKN1A | -0.251 | 8.3E-05 | mirtarbase | MIMAT0002869 | 1026 | ENSG00000124762 | Luciferase reporter assay//qRT-PCR//Western blot | Functional MTI | 22262409 | validated |
| CDKN1A | -0.251 | 8.3E-05 | mirtarbase | MIMAT0002869 | 1026 | ENSG00000124762 | qRT-PCR//Luciferase reporter assay//Western blot | Functional MTI | 24752803 | validated |
| CDKN1A | -0.251 | 8.3E-05 | tarbase | MIMAT0002869 | 1026 | ENSG00000124762 | Degradome sequencing//Degradome sequencing//Degradome sequencing//Degradome sequencing//Degradome sequencing//Degradome sequencing | positive |  | validated |
| CERCAM | -0.259 | 4.9E-05 | mirtarbase | MIMAT0002869 | 51148 | ENSG00000167123 | PAR-CLIP | Functional MTI (Weak) | 21572407 | validated |
| EGLN3 | -0.290 | 5.0E-06 | mirtarbase | MIMAT0002869 | 112399 | ENSG00000129521 | PAR-CLIP | Functional MTI (Weak) | 21572407 | validated |
| FBXL5 | -0.313 | 7.2E-07 | mirtarbase | MIMAT0002869 | 26234 | ENSG00000118564 | PAR-CLIP | Functional MTI (Weak) | 23592263 | validated |
| FBXL5 | -0.313 | 7.2E-07 | mirtarbase | MIMAT0002869 | 26234 | ENSG00000118564 | PAR-CLIP | Functional MTI (Weak) | 24398324 | validated |
| FBXL5 | -0.313 | 7.2E-07 | mirtarbase | MIMAT0002869 | 26234 | ENSG00000118564 | PAR-CLIP | Functional MTI (Weak) | 23446348 | validated |
| FBXL5 | -0.313 | 7.2E-07 | mirtarbase | MIMAT0002869 | 26234 | ENSG00000118564 | PAR-CLIP//HITS-CLIP | Functional MTI (Weak) | 21572407 | validated |
| FBXL5 | -0.313 | 7.2E-07 | mirtarbase | MIMAT0002869 | 26234 | ENSG00000118564 | PAR-CLIP | Functional MTI (Weak) | 20371350 | validated |
| FEM1C | -0.260 | 4.7E-05 | tarbase | MIMAT0002869 | 56929 | ENSG00000145780 | Degradome sequencing | positive |  | validated |
| KREMEN1 | -0.252 | 7.8E-05 | mirtarbase | MIMAT0002869 | 83999 | ENSG00000183762 | PAR-CLIP | Functional MTI (Weak) | 22012620 | validated |
| LIMA1 | -0.361 | 8.4E-09 | mirtarbase | MIMAT0002869 | 51474 | ENSG00000050405 | PAR-CLIP | Functional MTI (Weak) | 20371350 | validated |
| MIDN | -0.336 | 9.2E-08 | mirtarbase | MIMAT0002869 | 90007 | ENSG00000167470 | PAR-CLIP | Functional MTI (Weak) | 23592263 | validated |
| MIDN | -0.336 | 9.2E-08 | mirtarbase | MIMAT0002869 | 90007 | ENSG00000167470 | PAR-CLIP | Functional MTI (Weak) | 24398324 | validated |
| MIDN | -0.336 | 9.2E-08 | mirtarbase | MIMAT0002869 | 90007 | ENSG00000167470 | PAR-CLIP | Functional MTI (Weak) | 23446348 | validated |
| MIDN | -0.336 | 9.2E-08 | mirtarbase | MIMAT0002869 | 90007 | ENSG00000167470 | PAR-CLIP//HITS-CLIP | Functional MTI (Weak) | 21572407 | validated |
| MIDN | -0.336 | 9.2E-08 | mirtarbase | MIMAT0002869 | 90007 | ENSG00000167470 | PAR-CLIP | Functional MTI (Weak) | 20371350 | validated |
| MIDN | -0.336 | 9.2E-08 | mirtarbase | MIMAT0002869 | 90007 | ENSG00000167470 | PAR-CLIP | Functional MTI (Weak) | 27292025 | validated |
| MOCS2 | -0.266 | 3.1E-05 | mirtarbase | MIMAT0002869 | 4338 | ENSG00000164172 | HITS-CLIP | Functional MTI (Weak) | 19536157 | validated |
| MSMO1 | -0.306 | 1.4E-06 | mirtarbase | MIMAT0002869 | 6307 | ENSG00000052802 | PAR-CLIP | Functional MTI (Weak) | 21572407 | validated |
| RAB5B | -0.287 | 6.1E-06 | mirtarbase | MIMAT0002869 | 5869 | ENSG00000111540 | PAR-CLIP | Functional MTI (Weak) | 23592263 | validated |
| RAB5B | -0.287 | 6.1E-06 | mirtarbase | MIMAT0002869 | 5869 | ENSG00000111540 | PAR-CLIP | Functional MTI (Weak) | 23446348 | validated |
| RAB5B | -0.287 | 6.1E-06 | mirtarbase | MIMAT0002869 | 5869 | ENSG00000111540 | PAR-CLIP | Functional MTI (Weak) | 21572407 | validated |
| RAB5B | -0.287 | 6.1E-06 | mirtarbase | MIMAT0002869 | 5869 | ENSG00000111540 | PAR-CLIP | Functional MTI (Weak) | 20371350 | validated |
| RAB5B | -0.287 | 6.1E-06 | mirtarbase | MIMAT0002869 | 5869 | ENSG00000111540 | PAR-CLIP | Functional MTI (Weak) | 27292025 | validated |
| SEC16A | -0.285 | 7.3E-06 | mirtarbase | MIMAT0002869 | 9919 | ENSG00000148396 | PAR-CLIP | Functional MTI (Weak) | 26701625 | validated |
| AMDHD2 | -0.390 | 5.2E-10 | tarbase | MIMAT0004809 | 51005 | ENSG00000162066 | Degradome sequencing | positive |  | validated |
| ANO9 | -0.287 | 6.7E-06 | tarbase | MIMAT0004809 | 338440 | ENSG00000185101 | Degradome sequencing | positive |  | validated |
| C1orf35 | -0.283 | 9.3E-06 | tarbase | MIMAT0004809 | 79169 | ENSG00000143793 | Degradome sequencing | positive |  | validated |
| C7orf50 | -0.320 | 4.6E-07 | tarbase | MIMAT0004809 | 84310 | ENSG00000146540 | Degradome sequencing | positive |  | validated |
| CCDC137 | -0.287 | 6.9E-06 | tarbase | MIMAT0004809 | 339230 | ENSG00000185298 | Degradome sequencing | positive |  | validated |
| DAZAP1 | -0.289 | 6.0E-06 | tarbase | MIMAT0004809 | 26528 | ENSG00000071626 | Degradome sequencing | positive |  | validated |
| FARSA | -0.322 | 4.1E-07 | tarbase | MIMAT0004809 | 2193 | ENSG00000179115 | Degradome sequencing | positive |  | validated |
| FDXR | -0.316 | 6.4E-07 | mirtarbase | MIMAT0004809 | 2232 | ENSG00000161513 | PAR-CLIP | Functional MTI (Weak) | 26701625 | validated |
| INPP5E | -0.315 | 7.5E-07 | tarbase | MIMAT0004809 | 56623 | ENSG00000148384 | Degradome sequencing | positive |  | validated |
| MRPS2 | -0.297 | 3.1E-06 | tarbase | MIMAT0004809 | 51116 | ENSG00000122140 | Degradome sequencing | positive |  | validated |
| MXD4 | -0.268 | 2.8E-05 | tarbase | MIMAT0004809 | 10608 | ENSG00000123933 | Degradome sequencing | positive |  | validated |
| NLGN2 | -0.343 | 6.3E-08 | tarbase | MIMAT0004809 | 57555 | ENSG00000169992 | Degradome sequencing | positive |  | validated |
| OAZ1 | -0.339 | 8.9E-08 | tarbase | MIMAT0004809 | 4946 | ENSG00000104904 | Degradome sequencing//Degradome sequencing | positive |  | validated |
| PTGES2 | -0.310 | 1.1E-06 | tarbase | MIMAT0004809 | 80142 | ENSG00000148334 | Degradome sequencing | positive |  | validated |
| SSBP4 | -0.390 | 5.4E-10 | tarbase | MIMAT0004809 | 170463 | ENSG00000130511 | Degradome sequencing | positive |  | validated |
| ST3GAL2 | -0.263 | 3.8E-05 | tarbase | MIMAT0004809 | 6483 | ENSG00000157350 | Degradome sequencing | positive |  | validated |
| STK25 | -0.353 | 2.4E-08 | mirtarbase | MIMAT0004809 | 10494 | ENSG00000115694 | PAR-CLIP | Functional MTI (Weak) | 27292025 | validated |
| STRN4 | -0.253 | 7.8E-05 | tarbase | MIMAT0004809 | 29888 | ENSG00000090372 | Degradome sequencing | positive |  | validated |
| TRAPPC1 | -0.356 | 1.8E-08 | tarbase | MIMAT0004809 | 58485 | ENSG00000170043 | Degradome sequencing | positive |  | validated |
| TRIM41 | -0.346 | 4.4E-08 | mirtarbase | MIMAT0004809 | 90933 | ENSG00000146063 | PAR-CLIP | Functional MTI (Weak) | 26701625 | validated |
| TUBGCP6 | -0.262 | 4.2E-05 | tarbase | MIMAT0004809 | 85378 | ENSG00000128159 | Degradome sequencing | positive |  | validated |
| WDR81 | -0.261 | 4.5E-05 | tarbase | MIMAT0004809 | 124997 | ENSG00000167716 | Degradome sequencing | positive |  | validated |
