# Supplementary material for: Potential biomarkers as a predictive factor of response to primary chemotherapy in breast cancer patients
Source: Braz J Med Biol Res. 2024 Oct 7;57:e13599. doi: 10.1590/1414-431X2024e13599 (PMC11463908; doi:10.1590/1414-431X2024e13599)
Supplement: Supplementary file 1 [file 1414-431X-bjmbr-57-e13599-suppl.zip › 13559_Supplementary Table S2.docx]

**Supplementary Table S2.** Prediction of MicroRNA-Target Interactions in Breast Cancer Estrogen Receptor-Positive Patients Using the multiMiR R Package and Database.

|  | **miRNA** | **GeneId** | **rho** | **P_value** | **database** | **mature_mirna** | **target_entrez** | **target_ensembl** | **experiment** | **support_type** | **pubmed_id** | **type** |
| --- | --- | --- | --- | --- | --- | --- | --- | --- | --- | --- | --- | --- |
| 1 | hsa-let-7a | ALG3 | -0.319 | 1.1E-19 | tarbase | MIMAT0000062 | 10195 | ENSG00000214160 | Degradome sequencing//Degradome sequencing | positive |  | validated |
| 2 | hsa-let-7a | AURKA | -0.308 | 2.2E-18 | tarbase | MIMAT0000062 | 6790 | ENSG00000087586 | Degradome sequencing//Degradome sequencing//Degradome sequencing | positive |  | validated |
| 3 | hsa-let-7a | AURKB | -0.290 | 2.7E-16 | mirtarbase | MIMAT0000062 | 9212 | ENSG00000178999 | Luciferase reporter assay//Western blot | Functional MTI | 23769985 | validated |
| 4 | hsa-let-7a | AURKB | -0.290 | 2.7E-16 | tarbase | MIMAT0000062 | 9212 | ENSG00000178999 | Degradome sequencing//Degradome sequencing//Degradome sequencing | positive |  | validated |
| 5 | hsa-let-7a | CALR | -0.256 | 6.4E-13 | tarbase | MIMAT0000062 | 811 | ENSG00000179218 | Degradome sequencing | positive |  | validated |
| 6 | hsa-let-7a | CBX2 | -0.270 | 2.9E-14 | tarbase | MIMAT0000062 | 84733 | ENSG00000173894 | Degradome sequencing//Degradome sequencing | positive |  | validated |
| 7 | hsa-let-7a | CCNA2 | -0.269 | 3.4E-14 | tarbase | MIMAT0000062 | 890 | ENSG00000145386 | Degradome sequencing//Degradome sequencing//Degradome sequencing//Degradome sequencing//Degradome sequencing | positive |  | validated |
| 8 | hsa-let-7a | CCNB2 | -0.271 | 2.5E-14 | mirtarbase | MIMAT0000062 | 9133 | ENSG00000157456 | CLASH | Functional MTI (Weak) | 23622248 | validated |
| 9 | hsa-let-7a | CCNB2 | -0.271 | 2.5E-14 | tarbase | MIMAT0000062 | 9133 | ENSG00000157456 | Degradome sequencing | positive |  | validated |
| 10 | hsa-let-7a | CCNF | -0.294 | 6.7E-17 | tarbase | MIMAT0000062 | 899 | ENSG00000162063 | Degradome sequencing//Degradome sequencing | positive |  | validated |
| 11 | hsa-let-7a | CDT1 | -0.275 | 1.0E-14 | tarbase | MIMAT0000062 | 81620 | ENSG00000167513 | Degradome sequencing//Degradome sequencing | positive |  | validated |
| 12 | hsa-let-7a | CENPA | -0.273 | 1.6E-14 | tarbase | MIMAT0000062 | 1058 | ENSG00000115163 | Degradome sequencing | positive |  | validated |
| 13 | hsa-let-7a | CKAP4 | -0.261 | 2.1E-13 | tarbase | MIMAT0000062 | 10970 | ENSG00000136026 | Degradome sequencing | positive |  | validated |
| 14 | hsa-let-7a | CNOT11 | -0.270 | 2.1E-14 | tarbase | MIMAT0000062 | 55571 | ENSG00000158435 | Degradome sequencing | positive |  | validated |
| 15 | hsa-let-7a | DNAJB11 | -0.306 | 4.0E-18 | tarbase | MIMAT0000062 | 51726 | ENSG00000090520 | Degradome sequencing//Degradome sequencing//Degradome sequencing | positive |  | validated |
| 16 | hsa-let-7a | DONSON | -0.263 | 1.5E-13 | tarbase | MIMAT0000062 | 29980 | ENSG00000159147 | Degradome sequencing | positive |  | validated |
| 17 | hsa-let-7a | ECE2 | -0.360 | 0 | tarbase | MIMAT0000062 | 9718 | ENSG00000145194 | Degradome sequencing | positive |  | validated |
| 18 | hsa-let-7a | EIF1AD | -0.253 | 1.1E-12 | tarbase | MIMAT0000062 | 84285 | ENSG00000175376 | Degradome sequencing//Degradome sequencing//Degradome sequencing//Degradome sequencing | positive |  | validated |
| 19 | hsa-let-7a | ERGIC2 | -0.293 | 1.2E-16 | tarbase | MIMAT0000062 | 51290 | ENSG00000087502 | Degradome sequencing//Degradome sequencing//Degradome sequencing | positive |  | validated |
| 20 | hsa-let-7a | GARS1 | -0.281 | 2.1E-15 | tarbase | MIMAT0000062 | 2617 | ENSG00000106105 | Degradome sequencing//Degradome sequencing//Degradome sequencing//Degradome sequencing//Degradome sequencing//Degradome sequencing//Degradome sequencing//Degrad | positive |  | validated |
| 21 | hsa-let-7a | GLRX3 | -0.262 | 1.9E-13 | tarbase | MIMAT0000062 | 10539 | ENSG00000108010 | Degradome sequencing | positive |  | validated |
| 22 | hsa-let-7a | HMGA1 | -0.274 | 1.1E-14 | mirtarbase | MIMAT0000062 | 3159 | ENSG00000137309 | Luciferase reporter assay//qRT-PCR | Functional MTI | 19179606 | validated |
| 23 | hsa-let-7a | HMGA1 | -0.274 | 1.1E-14 | mirtarbase | MIMAT0000062 | 3159 | ENSG00000137309 | Luciferase reporter assay//qRT-PCR//Western blot | Functional MTI | 22139073 | validated |
| 24 | hsa-let-7a | HMGA1 | -0.274 | 1.1E-14 | mirtarbase | MIMAT0000062 | 3159 | ENSG00000137309 | CLASH | Functional MTI (Weak) | 23622248 | validated |
| 25 | hsa-let-7a | HMGA1 | -0.274 | 1.1E-14 | mirtarbase | MIMAT0000062 | 3159 | ENSG00000137309 | PAR-CLIP | Functional MTI (Weak) | 23592263 | validated |
| 26 | hsa-let-7a | HMGA1 | -0.274 | 1.1E-14 | mirtarbase | MIMAT0000062 | 3159 | ENSG00000137309 | HITS-CLIP | Functional MTI (Weak) | 23313552 | validated |
| 27 | hsa-let-7a | HMGA1 | -0.274 | 1.1E-14 | mirtarbase | MIMAT0000062 | 3159 | ENSG00000137309 | qRT-PCR//Western blot | Functional MTI | 25846193 | validated |
| 28 | hsa-let-7a | HMGA1 | -0.274 | 1.1E-14 | tarbase | MIMAT0000062 | 3159 | ENSG00000137309 | Degradome sequencing | positive |  | validated |
| 29 | hsa-let-7a | IDH2 | -0.268 | 4.9E-14 | tarbase | MIMAT0000062 | 3418 | ENSG00000182054 | Degradome sequencing | positive |  | validated |
| 30 | hsa-let-7a | JPT1 | -0.255 | 8.3E-13 | tarbase | MIMAT0000062 | 51155 | ENSG00000189159 | Degradome sequencing | positive |  | validated |
| 31 | hsa-let-7a | KCTD5 | -0.304 | 8.0E-18 | tarbase | MIMAT0000062 | 54442 | ENSG00000167977 | Degradome sequencing | positive |  | validated |
| 32 | hsa-let-7a | KPNA2 | -0.280 | 2.7E-15 | tarbase | MIMAT0000062 | 3838 | ENSG00000182481 | Degradome sequencing//Degradome sequencing//Degradome sequencing | positive |  | validated |
| 33 | hsa-let-7a | LRRC59 | -0.279 | 3.8E-15 | tarbase | MIMAT0000062 | 55379 | ENSG00000108829 | Degradome sequencing//Degradome sequencing | positive |  | validated |
| 34 | hsa-let-7a | MAPRE1 | -0.274 | 1.1E-14 | tarbase | MIMAT0000062 | 22919 | ENSG00000101367 | Degradome sequencing | positive |  | validated |
| 35 | hsa-let-7a | MEAK7 | -0.267 | 4.3E-14 | tarbase | MIMAT0000062 | 57707 | ENSG00000140950 | Degradome sequencing//Degradome sequencing//Degradome sequencing//Degradome sequencing | positive |  | validated |
| 36 | hsa-let-7a | MRPL15 | -0.272 | 1.8E-14 | mirtarbase | MIMAT0000062 | 29088 | ENSG00000137547 | CLASH | Functional MTI (Weak) | 23622248 | validated |
| 37 | hsa-let-7a | MYBL2 | -0.313 | 5.8E-19 | tarbase | MIMAT0000062 | 4605 | ENSG00000101057 | Degradome sequencing | positive |  | validated |
| 38 | hsa-let-7a | PCNA | -0.256 | 6.2E-13 | tarbase | MIMAT0000062 | 5111 | ENSG00000132646 | Degradome sequencing//Degradome sequencing | positive |  | validated |
| 39 | hsa-let-7a | PGAP6 | -0.279 | 3.5E-15 | tarbase | MIMAT0000062 | 58986 | ENSG00000129925 | Degradome sequencing | positive |  | validated |
| 40 | hsa-let-7a | RAB5IF | -0.303 | 8.4E-18 | tarbase | MIMAT0000062 | 55969 | ENSG00000101084 | Degradome sequencing | positive |  | validated |
| 41 | hsa-let-7a | RPS6KB2 | -0.272 | 1.7E-14 | tarbase | MIMAT0000062 | 6199 | ENSG00000175634 | Degradome sequencing//Degradome sequencing | positive |  | validated |
| 42 | hsa-let-7a | RRM2 | -0.290 | 2.7E-16 | mirtarbase | MIMAT0000062 | 6241 | ENSG00000171848 | CLASH | Functional MTI (Weak) | 23622248 | validated |
| 43 | hsa-let-7a | RRM2 | -0.290 | 2.7E-16 | mirtarbase | MIMAT0000062 | 6241 | ENSG00000171848 | PAR-CLIP | Functional MTI (Weak) | 21572407 | validated |
| 44 | hsa-let-7a | RRM2 | -0.290 | 2.7E-16 | mirtarbase | MIMAT0000062 | 6241 | ENSG00000171848 | qRT-PCR//Luciferase reporter assay//Western blot | Functional MTI | 23335963 | validated |
| 45 | hsa-let-7a | RRM2 | -0.290 | 2.7E-16 | tarbase | MIMAT0000062 | 6241 | ENSG00000171848 | Degradome sequencing//Degradome sequencing//Degradome sequencing | positive |  | validated |
| 46 | hsa-let-7a | SLC35A2 | -0.305 | 5.2E-18 | tarbase | MIMAT0000062 | 7355 | ENSG00000102100 | Degradome sequencing | positive |  | validated |
| 47 | hsa-let-7a | SPINDOC | -0.275 | 6.9E-15 | tarbase | MIMAT0000062 | 144097 | ENSG00000168005 | Degradome sequencing | positive |  | validated |
| 48 | hsa-let-7a | SURF4 | -0.289 | 3.7E-16 | mirtarbase | MIMAT0000062 | 6836 | ENSG00000148248 | PAR-CLIP | Functional MTI (Weak) | 20371350 | validated |
| 49 | hsa-let-7a | SURF4 | -0.289 | 3.7E-16 | mirtarbase | MIMAT0000062 | 6836 | ENSG00000280951 | PAR-CLIP | Functional MTI (Weak) | 20371350 | validated |
| 50 | hsa-let-7a | SURF4 | -0.289 | 3.7E-16 | tarbase | MIMAT0000062 | 6836 | ENSG00000148248 | Degradome sequencing//Degradome sequencing//Degradome sequencing | positive |  | validated |
| 51 | hsa-let-7a | SUV39H2 | -0.250 | 1.8E-12 | tarbase | MIMAT0000062 | 79723 | ENSG00000152455 | Degradome sequencing | positive |  | validated |
| 52 | hsa-let-7a | TK1 | -0.276 | 7.5E-15 | tarbase | MIMAT0000062 | 7083 | ENSG00000167900 | Degradome sequencing | positive |  | validated |
| 53 | hsa-let-7a | TPX2 | -0.263 | 1.4E-13 | tarbase | MIMAT0000062 | 22974 | ENSG00000088325 | Degradome sequencing | positive |  | validated |
| 54 | hsa-let-7a | TUBA1C | -0.334 | 0 | tarbase | MIMAT0000062 | 84790 | ENSG00000167553 | Degradome sequencing | positive |  | validated |
| 55 | hsa-let-7a | UHRF1 | -0.259 | 3.6E-13 | mirtarbase | MIMAT0000062 | 29128 | ENSG00000276043 | CLASH | Functional MTI (Weak) | 23622248 | validated |
| 56 | hsa-let-7a | UHRF1 | -0.259 | 3.6E-13 | mirtarbase | MIMAT0000062 | 29128 | ENSG00000276043 | Luciferase reporter assay//qRT-PCR//Western blot | Functional MTI | 26049093 | validated |
| 57 | hsa-let-7c | ABHD17C | -0.257 | 5.5E-13 | mirtarbase | MIMAT0000064 | 58489 | ENSG00000136379 | PAR-CLIP | Functional MTI (Weak) | 20371350 | validated |
| 58 | hsa-let-7c | ABHD17C | -0.257 | 5.5E-13 | tarbase | MIMAT0000064 | 58489 | ENSG00000136379 | Degradome sequencing | positive |  | validated |
| 59 | hsa-let-7c | ARID3A | -0.257 | 4.8E-13 | mirtarbase | MIMAT0000064 | 1820 | ENSG00000116017 | 3'LIFE | Functional MTI (Weak) | 25074381 | validated |
| 60 | hsa-let-7c | ARID3A | -0.257 | 4.8E-13 | mirtarbase | MIMAT0000064 | 1820 | ENSG00000116017 | PAR-CLIP | Functional MTI (Weak) | 23592263 | validated |
| 61 | hsa-let-7c | ARID3A | -0.257 | 4.8E-13 | tarbase | MIMAT0000064 | 1820 | ENSG00000116017 | Degradome sequencing//Degradome sequencing//Degradome sequencing//Degradome sequencing//Degradome sequencing//Degradome sequencing//Degradome sequencing//Degrad | positive |  | validated |
| 62 | hsa-let-7c | ARL6IP1 | -0.307 | 2.9E-18 | mirtarbase | MIMAT0000064 | 23204 | ENSG00000170540 | CLASH | Functional MTI (Weak) | 23622248 | validated |
| 63 | hsa-let-7c | ARL6IP1 | -0.307 | 2.9E-18 | tarbase | MIMAT0000064 | 23204 | ENSG00000170540 | Degradome sequencing | positive |  | validated |
| 64 | hsa-let-7c | ARMT1 | -0.319 | 8.5E-20 | tarbase | MIMAT0000064 | 79624 | ENSG00000146476 | Degradome sequencing | positive |  | validated |
| 65 | hsa-let-7c | ASPM | -0.293 | 1.2E-16 | tarbase | MIMAT0000064 | 259266 | ENSG00000066279 | Degradome sequencing | positive |  | validated |
| 66 | hsa-let-7c | ATP5F1B | -0.306 | 3.9E-18 | tarbase | MIMAT0000064 | 506 | ENSG00000110955 | Degradome sequencing | positive |  | validated |
| 67 | hsa-let-7c | AURKA | -0.388 | 0 | tarbase | MIMAT0000064 | 6790 | ENSG00000087586 | Degradome sequencing//Degradome sequencing | positive |  | validated |
| 68 | hsa-let-7c | AURKB | -0.372 | 0 | tarbase | MIMAT0000064 | 9212 | ENSG00000178999 | Degradome sequencing//Degradome sequencing | positive |  | validated |
| 69 | hsa-let-7c | BLM | -0.263 | 1.0E-13 | tarbase | MIMAT0000064 | 641 | ENSG00000197299 | Degradome sequencing | positive |  | validated |
| 70 | hsa-let-7c | BRI3BP | -0.336 | 6.9E-22 | mirtarbase | MIMAT0000064 | 140707 | ENSG00000184992 | HITS-CLIP | Functional MTI (Weak) | 23706177 | validated |
| 71 | hsa-let-7c | C18orf32 | -0.300 | 2.1E-17 | tarbase | MIMAT0000064 | 497661 | ENSG00000177576 | Degradome sequencing | positive |  | validated |
| 72 | hsa-let-7c | C1orf43 | -0.363 | 0 | tarbase | MIMAT0000064 | 25912 | ENSG00000143612 | Degradome sequencing | positive |  | validated |
| 73 | hsa-let-7c | C6orf136 | -0.310 | 1.5E-18 | tarbase | MIMAT0000064 | 221545 | ENSG00000204564 | Degradome sequencing | positive |  | validated |
| 74 | hsa-let-7c | CCNA2 | -0.343 | 0 | tarbase | MIMAT0000064 | 890 | ENSG00000145386 | Degradome sequencing//Degradome sequencing//Degradome sequencing | positive |  | validated |
| 75 | hsa-let-7c | CCNB2 | -0.374 | 0 | mirtarbase | MIMAT0000064 | 9133 | ENSG00000157456 | CLASH | Functional MTI (Weak) | 23622248 | validated |
| 76 | hsa-let-7c | CCNB2 | -0.374 | 0 | tarbase | MIMAT0000064 | 9133 | ENSG00000157456 | Degradome sequencing | positive |  | validated |
| 77 | hsa-let-7c | CCNE2 | -0.363 | 0 | tarbase | MIMAT0000064 | 9134 | ENSG00000175305 | Degradome sequencing//Degradome sequencing | positive |  | validated |
| 78 | hsa-let-7c | CCNF | -0.367 | 4.6E-26 | mirtarbase | MIMAT0000064 | 899 | ENSG00000162063 | CLASH | Functional MTI (Weak) | 23622248 | validated |
| 79 | hsa-let-7c | CCNF | -0.367 | 4.6E-26 | tarbase | MIMAT0000064 | 899 | ENSG00000162063 | Degradome sequencing//Degradome sequencing | positive |  | validated |
| 80 | hsa-let-7c | CCT8 | -0.280 | 2.8E-15 | tarbase | MIMAT0000064 | 10694 | ENSG00000156261 | Degradome sequencing | positive |  | validated |
| 81 | hsa-let-7c | CDC25A | -0.384 | 0 | mirtarbase | MIMAT0000064 | 993 | ENSG00000164045 | Immunohistochemistry//Luciferase reporter assay//qRT-PCR//Western blot | Functional MTI | 25909324 | validated |
| 82 | hsa-let-7c | CDCA8 | -0.360 | 0 | tarbase | MIMAT0000064 | 55143 | ENSG00000134690 | Degradome sequencing//Degradome sequencing | positive |  | validated |
| 83 | hsa-let-7c | CDT1 | -0.385 | 0 | tarbase | MIMAT0000064 | 81620 | ENSG00000167513 | Degradome sequencing//Degradome sequencing | positive |  | validated |
| 84 | hsa-let-7c | CENPA | -0.345 | 0 | tarbase | MIMAT0000064 | 1058 | ENSG00000115163 | Degradome sequencing | positive |  | validated |
| 85 | hsa-let-7c | CENPK | -0.302 | 1.4E-17 | tarbase | MIMAT0000064 | 64105 | ENSG00000123219 | Degradome sequencing | positive |  | validated |
| 86 | hsa-let-7c | CENPQ | -0.295 | 7.0E-17 | tarbase | MIMAT0000064 | 55166 | ENSG00000031691 | Degradome sequencing | positive |  | validated |
| 87 | hsa-let-7c | CIAO2A | -0.285 | 8.7E-16 | tarbase | MIMAT0000064 | 84191 | ENSG00000166797 | Degradome sequencing | positive |  | validated |
| 88 | hsa-let-7c | CKAP2L | -0.320 | 6.2E-20 | tarbase | MIMAT0000064 | 150468 | ENSG00000169607 | Degradome sequencing | positive |  | validated |
| 89 | hsa-let-7c | CLPTM1 | -0.268 | 5.1E-14 | tarbase | MIMAT0000064 | 1209 | ENSG00000104853 | Degradome sequencing//Degradome sequencing | positive |  | validated |
| 90 | hsa-let-7c | COPG1 | -0.365 | 0 | tarbase | MIMAT0000064 | 22820 | ENSG00000181789 | Degradome sequencing | positive |  | validated |
| 91 | hsa-let-7c | DNA2 | -0.264 | 1.0E-13 | mirtarbase | MIMAT0000064 | 1763 | ENSG00000138346 | PAR-CLIP | Functional MTI (Weak) | 23446348 | validated |
| 92 | hsa-let-7c | DNA2 | -0.264 | 1.0E-13 | tarbase | MIMAT0000064 | 1763 | ENSG00000138346 | Degradome sequencing//Degradome sequencing | positive |  | validated |
| 93 | hsa-let-7c | DNAJA3 | -0.342 | 0 | tarbase | MIMAT0000064 | 9093 | ENSG00000103423 | Degradome sequencing | positive |  | validated |
| 94 | hsa-let-7c | ECE2 | -0.276 | 6.7E-15 | tarbase | MIMAT0000064 | 9718 | ENSG00000145194 | Degradome sequencing | positive |  | validated |
| 95 | hsa-let-7c | EIF2AK1 | -0.376 | 0 | tarbase | MIMAT0000064 | 27102 | ENSG00000086232 | Degradome sequencing | positive |  | validated |
| 96 | hsa-let-7c | ELAVL1 | -0.268 | 3.4E-14 | tarbase | MIMAT0000064 | 1994 | ENSG00000066044 | Degradome sequencing | positive |  | validated |
| 97 | hsa-let-7c | ESPL1 | -0.360 | 0 | mirtarbase | MIMAT0000064 | 9700 | ENSG00000135476 | PAR-CLIP | Functional MTI (Weak) | 23446348 | validated |
| 98 | hsa-let-7c | ESPL1 | -0.360 | 0 | mirtarbase | MIMAT0000064 | 9700 | ENSG00000135476 | PAR-CLIP | Functional MTI (Weak) | 21572407 | validated |
| 99 | hsa-let-7c | ESPL1 | -0.360 | 0 | tarbase | MIMAT0000064 | 9700 | ENSG00000135476 | Degradome sequencing//Degradome sequencing | positive |  | validated |
| 100 | hsa-let-7c | ESRP1 | -0.371 | 0 | tarbase | MIMAT0000064 | 54845 | ENSG00000104413 | Degradome sequencing | positive |  | validated |
| 101 | hsa-let-7c | EXO1 | -0.313 | 5.0E-19 | tarbase | MIMAT0000064 | 9156 | ENSG00000174371 | Degradome sequencing | positive |  | validated |
| 102 | hsa-let-7c | EZH2 | -0.346 | 0 | mirtarbase | MIMAT0000064 | 2146 | ENSG00000106462 | 3'LIFE | Functional MTI (Weak) | 25074381 | validated |
| 103 | hsa-let-7c | EZH2 | -0.346 | 0 | tarbase | MIMAT0000064 | 2146 | ENSG00000106462 | Degradome sequencing | positive |  | validated |
| 104 | hsa-let-7c | FANCI | -0.337 | 0 | mirtarbase | MIMAT0000064 | 55215 | ENSG00000140525 | CLASH | Functional MTI (Weak) | 23622248 | validated |
| 105 | hsa-let-7c | FBXO45 | -0.290 | 2.5E-16 | tarbase | MIMAT0000064 | 200933 | ENSG00000174013 | Degradome sequencing//Degradome sequencing | positive |  | validated |
| 106 | hsa-let-7c | FKBP4 | -0.386 | 0 | tarbase | MIMAT0000064 | 2288 | ENSG00000004478 | Degradome sequencing | positive |  | validated |
| 107 | hsa-let-7c | FOXA1 | -0.349 | 0 | tarbase | MIMAT0000064 | 3169 | ENSG00000129514 | Degradome sequencing | positive |  | validated |
| 108 | hsa-let-7c | FOXK2 | -0.312 | 9.1E-19 | tarbase | MIMAT0000064 | 3607 | ENSG00000141568 | Degradome sequencing | positive |  | validated |
| 109 | hsa-let-7c | GDI1 | -0.340 | 0 | tarbase | MIMAT0000064 | 2664 | ENSG00000203879 | Degradome sequencing | positive |  | validated |
| 110 | hsa-let-7c | GPSM2 | -0.269 | 3.5E-14 | tarbase | MIMAT0000064 | 29899 | ENSG00000121957 | Degradome sequencing | positive |  | validated |
| 111 | hsa-let-7c | GRPEL1 | -0.293 | 1.1E-16 | tarbase | MIMAT0000064 | 80273 | ENSG00000109519 | Degradome sequencing//Degradome sequencing | positive |  | validated |
| 112 | hsa-let-7c | GRPEL2 | -0.251 | 1.9E-12 | mirtarbase | MIMAT0000064 | 134266 | ENSG00000164284 | PAR-CLIP | Functional MTI (Weak) | 23592263 | validated |
| 113 | hsa-let-7c | GRPEL2 | -0.251 | 1.9E-12 | mirtarbase | MIMAT0000064 | 134266 | ENSG00000164284 | PAR-CLIP | Functional MTI (Weak) | 24398324 | validated |
| 114 | hsa-let-7c | GRPEL2 | -0.251 | 1.9E-12 | mirtarbase | MIMAT0000064 | 134266 | ENSG00000164284 | PAR-CLIP | Functional MTI (Weak) | 23446348 | validated |
| 115 | hsa-let-7c | GRPEL2 | -0.251 | 1.9E-12 | mirtarbase | MIMAT0000064 | 134266 | ENSG00000164284 | PAR-CLIP | Functional MTI (Weak) | 21572407 | validated |
| 116 | hsa-let-7c | GRPEL2 | -0.251 | 1.9E-12 | mirtarbase | MIMAT0000064 | 134266 | ENSG00000164284 | PAR-CLIP | Functional MTI (Weak) | 20371350 | validated |
| 117 | hsa-let-7c | GRPEL2 | -0.251 | 1.9E-12 | mirtarbase | MIMAT0000064 | 134266 | ENSG00000164284 | PAR-CLIP | Functional MTI (Weak) | 26701625 | validated |
| 118 | hsa-let-7c | GRPEL2 | -0.251 | 1.9E-12 | tarbase | MIMAT0000064 | 134266 | ENSG00000164284 | Degradome sequencing//Degradome sequencing | positive |  | validated |
| 119 | hsa-let-7c | GSK3A | -0.259 | 3.2E-13 | mirtarbase | MIMAT0000064 | 2931 | ENSG00000105723 | CLASH | Functional MTI (Weak) | 23622248 | validated |
| 120 | hsa-let-7c | H2AX | -0.251 | 1.6E-12 | tarbase | MIMAT0000064 | 3014 | ENSG00000188486 | Degradome sequencing//Degradome sequencing//Degradome sequencing//Degradome sequencing | positive |  | validated |
| 121 | hsa-let-7c | H2BC13 | -0.268 | 3.9E-14 | tarbase | MIMAT0000064 | 8340 | ENSG00000185130 | Degradome sequencing | positive |  | validated |
| 122 | hsa-let-7c | H2BC5 | -0.262 | 1.7E-13 | mirtarbase | MIMAT0000064 | 3017 | ENSG00000158373 | PAR-CLIP | Functional MTI (Weak) | 24398324 | validated |
| 123 | hsa-let-7c | H2BC5 | -0.262 | 1.7E-13 | mirtarbase | MIMAT0000064 | 3017 | ENSG00000158373 | PAR-CLIP | Functional MTI (Weak) | 23446348 | validated |
| 124 | hsa-let-7c | HASPIN | -0.317 | 1.7E-19 | mirtarbase | MIMAT0000064 | 83903 | ENSG00000177602 | PAR-CLIP | Functional MTI (Weak) | 21572407 | validated |
| 125 | hsa-let-7c | HASPIN | -0.317 | 1.7E-19 | mirtarbase | MIMAT0000064 | 83903 | ENSG00000177602 | PAR-CLIP | Functional MTI (Weak) | 20371350 | validated |
| 126 | hsa-let-7c | HASPIN | -0.317 | 1.7E-19 | mirtarbase | MIMAT0000064 | 83903 | ENSG00000177602 | HITS-CLIP | Functional MTI (Weak) | 23706177 | validated |
| 127 | hsa-let-7c | HELLS | -0.290 | 2.9E-16 | tarbase | MIMAT0000064 | 3070 | ENSG00000119969 | Degradome sequencing//Degradome sequencing | positive |  | validated |
| 128 | hsa-let-7c | HMMR | -0.358 | 0 | tarbase | MIMAT0000064 | 3161 | ENSG00000072571 | Degradome sequencing | positive |  | validated |
| 129 | hsa-let-7c | HNRNPA2B1 | -0.355 | 0 | tarbase | MIMAT0000064 | 3181 | ENSG00000122566 | Degradome sequencing | positive |  | validated |
| 130 | hsa-let-7c | HPRT1 | -0.351 | 0 | tarbase | MIMAT0000064 | 3251 | ENSG00000165704 | Degradome sequencing | positive |  | validated |
| 131 | hsa-let-7c | HSBP1 | -0.273 | 1.6E-14 | tarbase | MIMAT0000064 | 3281 | ENSG00000230989 | Degradome sequencing | positive |  | validated |
| 132 | hsa-let-7c | HSDL1 | -0.254 | 1.0E-12 | tarbase | MIMAT0000064 | 83693 | ENSG00000103160 | Degradome sequencing | positive |  | validated |
| 133 | hsa-let-7c | HSP90AA1 | -0.269 | 3.9E-14 | tarbase | MIMAT0000064 | 3320 | ENSG00000080824 | Degradome sequencing | positive |  | validated |
| 134 | hsa-let-7c | HSP90B1 | -0.274 | 1.3E-14 | tarbase | MIMAT0000064 | 7184 | ENSG00000166598 | Degradome sequencing//Degradome sequencing | positive |  | validated |
| 135 | hsa-let-7c | KIF18B | -0.394 | 0 | tarbase | MIMAT0000064 | 146909 | ENSG00000186185 | Degradome sequencing | positive |  | validated |
| 136 | hsa-let-7c | KPNA2 | -0.357 | 0 | tarbase | MIMAT0000064 | 3838 | ENSG00000182481 | Degradome sequencing//Degradome sequencing//Degradome sequencing//Degradome sequencing | positive |  | validated |
| 137 | hsa-let-7c | LMNB1 | -0.307 | 3.6E-18 | tarbase | MIMAT0000064 | 4001 | ENSG00000113368 | Degradome sequencing//Degradome sequencing//Degradome sequencing | positive |  | validated |
| 138 | hsa-let-7c | LRP11 | -0.257 | 5.2E-13 | tarbase | MIMAT0000064 | 84918 | ENSG00000120256 | Degradome sequencing | positive |  | validated |
| 139 | hsa-let-7c | LRRC59 | -0.257 | 4.6E-13 | tarbase | MIMAT0000064 | 55379 | ENSG00000108829 | Degradome sequencing | positive |  | validated |
| 140 | hsa-let-7c | MAD2L1 | -0.357 | 0 | tarbase | MIMAT0000064 | 4085 | ENSG00000164109 | Degradome sequencing | positive |  | validated |
| 141 | hsa-let-7c | MARS1 | -0.260 | 2.6E-13 | tarbase | MIMAT0000064 | 4141 | ENSG00000166986 | Degradome sequencing//Degradome sequencing//Degradome sequencing//Degradome sequencing//Degradome sequencing | positive |  | validated |
| 142 | hsa-let-7c | MASTL | -0.279 | 3.7E-15 | tarbase | MIMAT0000064 | 84930 | ENSG00000120539 | Degradome sequencing | positive |  | validated |
| 143 | hsa-let-7c | MCM3 | -0.277 | 5.9E-15 | tarbase | MIMAT0000064 | 4172 | ENSG00000112118 | Degradome sequencing | positive |  | validated |
| 144 | hsa-let-7c | MCM4 | -0.253 | 1.1E-12 | tarbase | MIMAT0000064 | 4173 | ENSG00000104738 | Degradome sequencing//Degradome sequencing//Degradome sequencing//Degradome sequencing//Degradome sequencing | positive |  | validated |
| 145 | hsa-let-7c | MKI67 | -0.297 | 4.8E-17 | tarbase | MIMAT0000064 | 4288 | ENSG00000148773 | Degradome sequencing//Degradome sequencing | positive |  | validated |
| 146 | hsa-let-7c | MRPL12 | -0.260 | 2.7E-13 | mirtarbase | MIMAT0000064 | 6182 | ENSG00000262814 | PAR-CLIP | Functional MTI (Weak) | 23592263 | validated |
| 147 | hsa-let-7c | MRPL17 | -0.263 | 1.4E-13 | tarbase | MIMAT0000064 | 63875 | ENSG00000158042 | Degradome sequencing | positive |  | validated |
| 148 | hsa-let-7c | MRPL58 | -0.287 | 5.3E-16 | tarbase | MIMAT0000064 | 3396 | ENSG00000167862 | Degradome sequencing | positive |  | validated |
| 149 | hsa-let-7c | MSI2 | -0.276 | 7.8E-15 | mirtarbase | MIMAT0000064 | 124540 | ENSG00000153944 | PAR-CLIP | Functional MTI (Weak) | 21572407 | validated |
| 150 | hsa-let-7c | NAA20 | -0.264 | 1.1E-13 | mirtarbase | MIMAT0000064 | 51126 | ENSG00000173418 | PAR-CLIP | Functional MTI (Weak) | 23592263 | validated |
| 151 | hsa-let-7c | NAA20 | -0.264 | 1.1E-13 | tarbase | MIMAT0000064 | 51126 | ENSG00000173418 | Degradome sequencing | positive |  | validated |
| 152 | hsa-let-7c | NECAB3 | -0.343 | 0 | tarbase | MIMAT0000064 | 63941 | ENSG00000125967 | Degradome sequencing | positive |  | validated |
| 153 | hsa-let-7c | NIPA1 | -0.326 | 6.7E-21 | tarbase | MIMAT0000064 | 123606 | ENSG00000170113 | Degradome sequencing | positive |  | validated |
| 154 | hsa-let-7c | NUCB2 | -0.254 | 1.0E-12 | mirtarbase | MIMAT0000064 | 4925 | ENSG00000070081 | PAR-CLIP | Functional MTI (Weak) | 23592263 | validated |
| 155 | hsa-let-7c | NUDT5 | -0.251 | 1.9E-12 | tarbase | MIMAT0000064 | 11164 | ENSG00000165609 | Degradome sequencing | positive |  | validated |
| 156 | hsa-let-7c | PCNA | -0.373 | 0 | tarbase | MIMAT0000064 | 5111 | ENSG00000132646 | Degradome sequencing | positive |  | validated |
| 157 | hsa-let-7c | PDHX | -0.312 | 8.7E-19 | tarbase | MIMAT0000064 | 8050 | ENSG00000110435 | Degradome sequencing | positive |  | validated |
| 158 | hsa-let-7c | PDIA3 | -0.252 | 1.4E-12 | tarbase | MIMAT0000064 | 2923 | ENSG00000167004 | Degradome sequencing | positive |  | validated |
| 159 | hsa-let-7c | PHKA1 | -0.298 | 3.4E-17 | tarbase | MIMAT0000064 | 5255 | ENSG00000067177 | Degradome sequencing//Degradome sequencing | positive |  | validated |
| 160 | hsa-let-7c | PIGP | -0.270 | 2.7E-14 | tarbase | MIMAT0000064 | 51227 | ENSG00000185808 | Degradome sequencing | positive |  | validated |
| 161 | hsa-let-7c | PIGT | -0.290 | 2.8E-16 | tarbase | MIMAT0000064 | 51604 | ENSG00000124155 | Degradome sequencing | positive |  | validated |
| 162 | hsa-let-7c | PIGW | -0.284 | 1.3E-15 | tarbase | MIMAT0000064 | 284098 | ENSG00000277161 | Degradome sequencing | positive |  | validated |
| 163 | hsa-let-7c | PIP4K2C | -0.273 | 1.4E-14 | tarbase | MIMAT0000064 | 79837 | ENSG00000166908 | Degradome sequencing//Degradome sequencing | positive |  | validated |
| 164 | hsa-let-7c | POLQ | -0.345 | 0 | tarbase | MIMAT0000064 | 10721 | ENSG00000051341 | Degradome sequencing//Degradome sequencing | positive |  | validated |
| 165 | hsa-let-7c | PPP1CA | -0.257 | 4.8E-13 | tarbase | MIMAT0000064 | 5499 | ENSG00000172531 | Degradome sequencing//Degradome sequencing | positive |  | validated |
| 166 | hsa-let-7c | PPP1R3D | -0.281 | 2.5E-15 | tarbase | MIMAT0000064 | 5509 | ENSG00000132825 | Degradome sequencing | positive |  | validated |
| 167 | hsa-let-7c | PRKAG1 | -0.299 | 2.5E-17 | tarbase | MIMAT0000064 | 5571 | ENSG00000181929 | Degradome sequencing | positive |  | validated |
| 168 | hsa-let-7c | PSMC3IP | -0.336 | 0 | tarbase | MIMAT0000064 | 29893 | ENSG00000131470 | Degradome sequencing | positive |  | validated |
| 169 | hsa-let-7c | PSMD11 | -0.283 | 1.3E-15 | tarbase | MIMAT0000064 | 5717 | ENSG00000108671 | Degradome sequencing//Degradome sequencing | positive |  | validated |
| 170 | hsa-let-7c | PSMD8 | -0.257 | 5.5E-13 | tarbase | MIMAT0000064 | 5714 | ENSG00000099341 | Degradome sequencing | positive |  | validated |
| 171 | hsa-let-7c | RAD54B | -0.280 | 3.0E-15 | tarbase | MIMAT0000064 | 25788 | ENSG00000197275 | Degradome sequencing | positive |  | validated |
| 172 | hsa-let-7c | RAD54L | -0.352 | 0 | tarbase | MIMAT0000064 | 8438 | ENSG00000085999 | Degradome sequencing | positive |  | validated |
| 173 | hsa-let-7c | RAE1 | -0.297 | 4.1E-17 | tarbase | MIMAT0000064 | 8480 | ENSG00000101146 | Degradome sequencing | positive |  | validated |
| 174 | hsa-let-7c | RFC5 | -0.305 | 4.0E-18 | tarbase | MIMAT0000064 | 5985 | ENSG00000111445 | Degradome sequencing | positive |  | validated |
| 175 | hsa-let-7c | RNFT1 | -0.250 | 2.1E-12 | mirtarbase | MIMAT0000064 | 51136 | ENSG00000189050 | PAR-CLIP | Functional MTI (Weak) | 24398324 | validated |
| 176 | hsa-let-7c | RNFT1 | -0.250 | 2.1E-12 | tarbase | MIMAT0000064 | 51136 | ENSG00000189050 | Degradome sequencing | positive |  | validated |
| 177 | hsa-let-7c | RPN2 | -0.335 | 0 | tarbase | MIMAT0000064 | 6185 | ENSG00000118705 | Degradome sequencing//Degradome sequencing | positive |  | validated |
| 178 | hsa-let-7c | RRM2 | -0.343 | 0 | mirtarbase | MIMAT0000064 | 6241 | ENSG00000171848 | PAR-CLIP | Functional MTI (Weak) | 21572407 | validated |
| 179 | hsa-let-7c | RRM2 | -0.343 | 0 | tarbase | MIMAT0000064 | 6241 | ENSG00000171848 | Degradome sequencing//Degradome sequencing//Degradome sequencing | positive |  | validated |
| 180 | hsa-let-7c | SGO1 | -0.346 | 4.2E-23 | tarbase | MIMAT0000064 | 151648 | ENSG00000129810 | Degradome sequencing | positive |  | validated |
| 181 | hsa-let-7c | SLC35A2 | -0.284 | 1.1E-15 | tarbase | MIMAT0000064 | 7355 | ENSG00000102100 | Degradome sequencing | positive |  | validated |
| 182 | hsa-let-7c | SLC9A3R1 | -0.274 | 1.3E-14 | tarbase | MIMAT0000064 | 9368 | ENSG00000109062 | Degradome sequencing | positive |  | validated |
| 183 | hsa-let-7c | SMC4 | -0.295 | 8.2E-17 | tarbase | MIMAT0000064 | 10051 | ENSG00000113810 | Degradome sequencing | positive |  | validated |
| 184 | hsa-let-7c | SRSF1 | -0.267 | 6.0E-14 | tarbase | MIMAT0000064 | 6426 | ENSG00000136450 | Degradome sequencing//Degradome sequencing | positive |  | validated |
| 185 | hsa-let-7c | TACC3 | -0.306 | 4.6E-18 | tarbase | MIMAT0000064 | 10460 | ENSG00000013810 | Degradome sequencing | positive |  | validated |
| 186 | hsa-let-7c | TBRG4 | -0.253 | 1.2E-12 | tarbase | MIMAT0000064 | 9238 | ENSG00000136270 | Degradome sequencing | positive |  | validated |
| 187 | hsa-let-7c | TICRR | -0.296 | 5.8E-17 | tarbase | MIMAT0000064 | 90381 | ENSG00000140534 | Degradome sequencing | positive |  | validated |
| 188 | hsa-let-7c | TLCD1 | -0.334 | 0 | tarbase | MIMAT0000064 | 116238 | ENSG00000160606 | Degradome sequencing | positive |  | validated |
| 189 | hsa-let-7c | TPX2 | -0.374 | 0 | tarbase | MIMAT0000064 | 22974 | ENSG00000088325 | Degradome sequencing | positive |  | validated |
| 190 | hsa-let-7c | TUBG1 | -0.321 | 4.5E-20 | tarbase | MIMAT0000064 | 7283 | ENSG00000131462 | Degradome sequencing | positive |  | validated |
| 191 | hsa-let-7c | UBE2O | -0.320 | 7.4E-20 | tarbase | MIMAT0000064 | 63893 | ENSG00000175931 | Degradome sequencing | positive |  | validated |
| 192 | hsa-let-7c | UBE2T | -0.401 | 0 | tarbase | MIMAT0000064 | 29089 | ENSG00000077152 | Degradome sequencing | positive |  | validated |
| 193 | hsa-let-7c | UNG | -0.307 | 3.5E-18 | tarbase | MIMAT0000064 | 7374 | ENSG00000076248 | Degradome sequencing | positive |  | validated |
| 194 | hsa-let-7c | WBP11 | -0.283 | 1.4E-15 | mirtarbase | MIMAT0000064 | 51729 | ENSG00000084463 | CLASH | Functional MTI (Weak) | 23622248 | validated |
| 521 | hsa-mir-107 | AK5 | -0.307 | 2.9E-18 | tarbase | MIMAT0000104 | 26289 | ENSG00000154027 | Degradome sequencing | negative |  | validated |
| 522 | hsa-mir-107 | ANTXR1 | -0.251 | 2.0E-12 | tarbase | MIMAT0000104 | 84168 | ENSG00000169604 | Degradome sequencing | negative |  | validated |
| 523 | hsa-mir-107 | ANTXR2 | -0.259 | 3.5E-13 | tarbase | MIMAT0000104 | 118429 | ENSG00000163297 | Degradome sequencing | negative |  | validated |
| 524 | hsa-mir-107 | ARHGAP20 | -0.293 | 1.1E-16 | tarbase | MIMAT0000104 | 57569 | ENSG00000137727 | Degradome sequencing | negative |  | validated |
| 525 | hsa-mir-107 | ARHGEF12 | -0.319 | 1.1E-19 | tarbase | MIMAT0000104 | 23365 | ENSG00000196914 | Degradome sequencing | positive |  | validated |
| 526 | hsa-mir-107 | ARID5B | -0.280 | 3.1E-15 | tarbase | MIMAT0000104 | 84159 | ENSG00000150347 | Degradome sequencing | positive |  | validated |
| 527 | hsa-mir-107 | ARL6IP5 | -0.260 | 2.9E-13 | tarbase | MIMAT0000104 | 10550 | ENSG00000144746 | Degradome sequencing | positive |  | validated |
| 528 | hsa-mir-107 | ATF7 | -0.366 | 0 | tarbase | MIMAT0000104 | 11016 | ENSG00000170653 | Degradome sequencing//Degradome sequencing//Degradome sequencing | positive |  | validated |
| 529 | hsa-mir-107 | BICC1 | -0.262 | 1.3E-13 | tarbase | MIMAT0000104 | 80114 | ENSG00000122870 | Degradome sequencing | negative |  | validated |
| 530 | hsa-mir-107 | BMPR2 | -0.259 | 3.3E-13 | tarbase | MIMAT0000104 | 659 | ENSG00000204217 | Degradome sequencing | positive |  | validated |
| 531 | hsa-mir-107 | BMPR2 | -0.259 | 3.3E-13 | tarbase | MIMAT0000104 | 659 | ENSG00000204217 | Degradome sequencing | negative |  | validated |
| 532 | hsa-mir-107 | C6orf120 | -0.257 | 5.2E-13 | tarbase | MIMAT0000104 | 387263 | ENSG00000185127 | Degradome sequencing//Degradome sequencing//Degradome sequencing | positive |  | validated |
| 533 | hsa-mir-107 | CAB39 | -0.263 | 1.4E-13 | mirtarbase | MIMAT0000104 | 51719 | ENSG00000135932 | PAR-CLIP | Functional MTI (Weak) | 24398324 | validated |
| 534 | hsa-mir-107 | CAB39 | -0.263 | 1.4E-13 | tarbase | MIMAT0000104 | 51719 | ENSG00000135932 | Degradome sequencing | positive |  | validated |
| 535 | hsa-mir-107 | CALD1 | -0.268 | 4.1E-14 | tarbase | MIMAT0000104 | 800 | ENSG00000122786 | Degradome sequencing | negative |  | validated |
| 536 | hsa-mir-107 | CCDC186 | -0.268 | 4.9E-14 | tarbase | MIMAT0000104 | 55088 | ENSG00000165813 | Degradome sequencing | negative |  | validated |
| 537 | hsa-mir-107 | CD59 | -0.260 | 2.8E-13 | tarbase | MIMAT0000104 | 966 | ENSG00000085063 | Degradome sequencing//Degradome sequencing//Degradome sequencing//Degradome sequencing | positive |  | validated |
| 538 | hsa-mir-107 | CDC14B | -0.291 | 1.6E-16 | tarbase | MIMAT0000104 | 8555 | ENSG00000081377 | Degradome sequencing | negative |  | validated |
| 539 | hsa-mir-107 | CHMP3 | -0.303 | 6.9E-18 | tarbase | MIMAT0000104 | 51652 | ENSG00000115561 | Degradome sequencing//Degradome sequencing//Degradome sequencing//Degradome sequencing | positive |  | validated |
| 540 | hsa-mir-107 | CHSY1 | -0.277 | 6.6E-15 | tarbase | MIMAT0000104 | 22856 | ENSG00000131873 | Degradome sequencing | positive |  | validated |
| 541 | hsa-mir-107 | CHSY1 | -0.277 | 6.6E-15 | tarbase | MIMAT0000104 | 22856 | ENSG00000131873 | Degradome sequencing | negative |  | validated |
| 542 | hsa-mir-107 | COBLL1 | -0.255 | 7.2E-13 | tarbase | MIMAT0000104 | 22837 | ENSG00000082438 | Degradome sequencing | positive |  | validated |
| 543 | hsa-mir-107 | COL14A1 | -0.271 | 2.4E-14 | tarbase | MIMAT0000104 | 7373 | ENSG00000187955 | Degradome sequencing | positive |  | validated |
| 544 | hsa-mir-107 | CRIM1 | -0.339 | 0 | tarbase | MIMAT0000104 | 51232 | ENSG00000150938 | Degradome sequencing//Degradome sequencing | positive |  | validated |
| 545 | hsa-mir-107 | CTDSPL | -0.266 | 6.8E-14 | tarbase | MIMAT0000104 | 10217 | ENSG00000144677 | Degradome sequencing | positive |  | validated |
| 546 | hsa-mir-107 | CTNNB1 | -0.311 | 1.2E-18 | tarbase | MIMAT0000104 | 1499 | ENSG00000168036 | Degradome sequencing//Degradome sequencing//Degradome sequencing | positive |  | validated |
| 547 | hsa-mir-107 | CTNNB1 | -0.311 | 1.2E-18 | tarbase | MIMAT0000104 | 1499 | ENSG00000168036 | Degradome sequencing | negative |  | validated |
| 548 | hsa-mir-107 | CYBRD1 | -0.302 | 1.4E-17 | tarbase | MIMAT0000104 | 79901 | ENSG00000071967 | Degradome sequencing | negative |  | validated |
| 549 | hsa-mir-107 | DIXDC1 | -0.355 | 0 | tarbase | MIMAT0000104 | 85458 | ENSG00000150764 | Degradome sequencing | negative |  | validated |
| 550 | hsa-mir-107 | DST | -0.287 | 5.7E-16 | mirtarbase | MIMAT0000104 | 667 | ENSG00000151914 | PAR-CLIP | Functional MTI (Weak) | 22012620 | validated |
| 551 | hsa-mir-107 | DST | -0.287 | 5.7E-16 | tarbase | MIMAT0000104 | 667 | ENSG00000151914 | Degradome sequencing | positive |  | validated |
| 552 | hsa-mir-107 | EDIL3 | -0.253 | 1.0E-12 | tarbase | MIMAT0000104 | 10085 | ENSG00000164176 | Degradome sequencing | positive |  | validated |
| 553 | hsa-mir-107 | EIF4EBP2 | -0.254 | 9.6E-13 | tarbase | MIMAT0000104 | 1979 | ENSG00000148730 | Degradome sequencing | negative |  | validated |
| 554 | hsa-mir-107 | ETV1 | -0.254 | 9.7E-13 | tarbase | MIMAT0000104 | 2115 | ENSG00000006468 | Degradome sequencing | positive |  | validated |
| 555 | hsa-mir-107 | ETV1 | -0.254 | 9.7E-13 | tarbase | MIMAT0000104 | 2115 | ENSG00000006468 | Degradome sequencing | negative |  | validated |
| 556 | hsa-mir-107 | FAM114A1 | -0.275 | 8.8E-15 | tarbase | MIMAT0000104 | 92689 | ENSG00000197712 | Degradome sequencing | negative |  | validated |
| 557 | hsa-mir-107 | FAT1 | -0.262 | 1.6E-13 | tarbase | MIMAT0000104 | 2195 | ENSG00000083857 | Degradome sequencing | negative |  | validated |
| 558 | hsa-mir-107 | FAT4 | -0.266 | 7.0E-14 | tarbase | MIMAT0000104 | 79633 | ENSG00000196159 | Degradome sequencing | positive |  | validated |
| 559 | hsa-mir-107 | FGF1 | -0.306 | 3.9E-18 | tarbase | MIMAT0000104 | 2246 | ENSG00000113578 | Degradome sequencing | negative |  | validated |
| 560 | hsa-mir-107 | FNDC3A | -0.303 | 1.0E-17 | tarbase | MIMAT0000104 | 22862 | ENSG00000102531 | Degradome sequencing | positive |  | validated |
| 561 | hsa-mir-107 | FNDC3B | -0.270 | 2.9E-14 | tarbase | MIMAT0000104 | 64778 | ENSG00000075420 | Degradome sequencing//Degradome sequencing | positive |  | validated |
| 562 | hsa-mir-107 | FRMD6 | -0.296 | 6.0E-17 | tarbase | MIMAT0000104 | 122786 | ENSG00000139926 | Degradome sequencing | negative |  | validated |
| 563 | hsa-mir-107 | FST | -0.250 | 1.7E-12 | tarbase | MIMAT0000104 | 10468 | ENSG00000134363 | Degradome sequencing | negative |  | validated |
| 564 | hsa-mir-107 | FYCO1 | -0.301 | 1.8E-17 | tarbase | MIMAT0000104 | 79443 | ENSG00000163820 | Degradome sequencing//Degradome sequencing | positive |  | validated |
| 565 | hsa-mir-107 | FYCO1 | -0.301 | 1.8E-17 | tarbase | MIMAT0000104 | 79443 | ENSG00000163820 | Degradome sequencing | negative |  | validated |
| 566 | hsa-mir-107 | FZD1 | -0.332 | 0 | tarbase | MIMAT0000104 | 8321 | ENSG00000157240 | Degradome sequencing | negative |  | validated |
| 567 | hsa-mir-107 | GALNT7 | -0.270 | 3.3E-14 | tarbase | MIMAT0000104 | 51809 | ENSG00000109586 | Degradome sequencing//Degradome sequencing//Degradome sequencing | positive |  | validated |
| 568 | hsa-mir-107 | GNG12 | -0.409 | 0 | mirtarbase | MIMAT0000104 | 55970 | ENSG00000172380 | PAR-CLIP | Functional MTI (Weak) | 20371350 | validated |
| 569 | hsa-mir-107 | GNG12 | -0.409 | 0 | tarbase | MIMAT0000104 | 55970 | ENSG00000172380 | Degradome sequencing | positive |  | validated |
| 570 | hsa-mir-107 | GOLIM4 | -0.263 | 1.4E-13 | tarbase | MIMAT0000104 | 27333 | ENSG00000173905 | Degradome sequencing | positive |  | validated |
| 571 | hsa-mir-107 | GOLIM4 | -0.263 | 1.4E-13 | tarbase | MIMAT0000104 | 27333 | ENSG00000173905 | Degradome sequencing | negative |  | validated |
| 572 | hsa-mir-107 | GRAMD2B | -0.257 | 5.4E-13 | tarbase | MIMAT0000104 | 65983 | ENSG00000155324 | Degradome sequencing//Degradome sequencing | positive |  | validated |
| 573 | hsa-mir-107 | GUCY1A1 | -0.276 | 7.2E-15 | tarbase | MIMAT0000104 | 2982 | ENSG00000164116 | Degradome sequencing | positive |  | validated |
| 574 | hsa-mir-107 | HBP1 | -0.336 | 0 | tarbase | MIMAT0000104 | 26959 | ENSG00000105856 | Degradome sequencing | positive |  | validated |
| 575 | hsa-mir-107 | HBP1 | -0.336 | 0 | tarbase | MIMAT0000104 | 26959 | ENSG00000105856 | Degradome sequencing | negative |  | validated |
| 576 | hsa-mir-107 | HCFC2 | -0.264 | 1.2E-13 | mirtarbase | MIMAT0000104 | 29915 | ENSG00000111727 | PAR-CLIP | Functional MTI (Weak) | 21572407 | validated |
| 577 | hsa-mir-107 | HCFC2 | -0.264 | 1.2E-13 | mirtarbase | MIMAT0000104 | 29915 | ENSG00000111727 | PAR-CLIP | Functional MTI (Weak) | 20371350 | validated |
| 578 | hsa-mir-107 | HFE | -0.251 | 1.7E-12 | tarbase | MIMAT0000104 | 3077 | ENSG00000010704 | Degradome sequencing | positive |  | validated |
| 579 | hsa-mir-107 | HOXA7 | -0.260 | 2.6E-13 | tarbase | MIMAT0000104 | 3204 | ENSG00000122592 | Degradome sequencing | positive |  | validated |
| 580 | hsa-mir-107 | IL1RAP | -0.269 | 3.9E-14 | tarbase | MIMAT0000104 | 3556 | ENSG00000196083 | Degradome sequencing | positive |  | validated |
| 581 | hsa-mir-107 | IQGAP1 | -0.250 | 1.7E-12 | tarbase | MIMAT0000104 | 8826 | ENSG00000140575 | Degradome sequencing | positive |  | validated |
| 582 | hsa-mir-107 | ITGB3 | -0.258 | 4.0E-13 | tarbase | MIMAT0000104 | 3690 | ENSG00000259207 | Degradome sequencing | negative |  | validated |
| 583 | hsa-mir-107 | JMJD1C | -0.267 | 5.5E-14 | tarbase | MIMAT0000104 | 221037 | ENSG00000171988 | Degradome sequencing | negative |  | validated |
| 584 | hsa-mir-107 | KLHL9 | -0.251 | 1.9E-12 | tarbase | MIMAT0000104 | 55958 | ENSG00000198642 | Degradome sequencing//Degradome sequencing//Degradome sequencing | positive |  | validated |
| 585 | hsa-mir-107 | KMT2E | -0.255 | 8.1E-13 | tarbase | MIMAT0000104 | 55904 | ENSG00000005483 | Degradome sequencing | positive |  | validated |
| 586 | hsa-mir-107 | LRIG1 | -0.253 | 1.1E-12 | tarbase | MIMAT0000104 | 26018 | ENSG00000144749 | Degradome sequencing//Degradome sequencing | positive |  | validated |
| 587 | hsa-mir-107 | LRIG3 | -0.262 | 1.8E-13 | tarbase | MIMAT0000104 | 121227 | ENSG00000139263 | Degradome sequencing | positive |  | validated |
| 588 | hsa-mir-107 | MAML2 | -0.250 | 2.2E-12 | tarbase | MIMAT0000104 | 84441 | ENSG00000184384 | Degradome sequencing | negative |  | validated |
| 589 | hsa-mir-107 | MBNL2 | -0.333 | 0 | tarbase | MIMAT0000104 | 10150 | ENSG00000139793 | Degradome sequencing | negative |  | validated |
| 590 | hsa-mir-107 | MCFD2 | -0.305 | 5.1E-18 | tarbase | MIMAT0000104 | 90411 | ENSG00000180398 | Degradome sequencing//Degradome sequencing | positive |  | validated |
| 591 | hsa-mir-107 | NEDD4 | -0.252 | 1.2E-12 | tarbase | MIMAT0000104 | 4734 | ENSG00000069869 | Degradome sequencing | negative |  | validated |
| 592 | hsa-mir-107 | NOTCH2 | -0.302 | 1.1E-17 | mirtarbase | MIMAT0000104 | 4853 | ENSG00000134250 | Luciferase reporter assay//qRT-PCR//Western blot | Functional MTI | 23299462 | validated |
| 593 | hsa-mir-107 | NOTCH2 | -0.302 | 1.1E-17 | mirtarbase | MIMAT0000104 | 4853 | ENSG00000134250 | Western blot | Functional MTI | 23220650 | validated |
| 594 | hsa-mir-107 | NOTCH2 | -0.302 | 1.1E-17 | mirtarbase | MIMAT0000104 | 4853 | ENSG00000134250 | qRT-PCR//Western blot | Functional MTI | 23572380 | validated |
| 595 | hsa-mir-107 | NOTCH2 | -0.302 | 1.1E-17 | tarbase | MIMAT0000104 | 4853 | ENSG00000134250 | Degradome sequencing//Degradome sequencing//Degradome sequencing | positive |  | validated |
| 596 | hsa-mir-107 | NTN4 | -0.311 | 1.2E-18 | tarbase | MIMAT0000104 | 59277 | ENSG00000074527 | Degradome sequencing | positive |  | validated |
| 597 | hsa-mir-107 | OSMR | -0.256 | 6.7E-13 | tarbase | MIMAT0000104 | 9180 | ENSG00000145623 | Degradome sequencing | negative |  | validated |
| 598 | hsa-mir-107 | PCDH7 | -0.254 | 8.9E-13 | tarbase | MIMAT0000104 | 5099 | ENSG00000169851 | Degradome sequencing | positive |  | validated |
| 599 | hsa-mir-107 | PDZRN3 | -0.267 | 6.3E-14 | tarbase | MIMAT0000104 | 23024 | ENSG00000121440 | Degradome sequencing | negative |  | validated |
| 600 | hsa-mir-107 | PIK3R1 | -0.252 | 1.4E-12 | mirtarbase | MIMAT0000104 | 5295 | ENSG00000145675 | PAR-CLIP | Functional MTI (Weak) | 23446348 | validated |
| 601 | hsa-mir-107 | PIK3R1 | -0.252 | 1.4E-12 | mirtarbase | MIMAT0000104 | 5295 | ENSG00000145675 | PAR-CLIP//HITS-CLIP | Functional MTI (Weak) | 21572407 | validated |
| 602 | hsa-mir-107 | PIK3R1 | -0.252 | 1.4E-12 | mirtarbase | MIMAT0000104 | 5295 | ENSG00000145675 | HITS-CLIP | Functional MTI (Weak) | 23824327 | validated |
| 603 | hsa-mir-107 | PIK3R1 | -0.252 | 1.4E-12 | mirtarbase | MIMAT0000104 | 5295 | ENSG00000145675 | HITS-CLIP | Functional MTI (Weak) | 23313552 | validated |
| 604 | hsa-mir-107 | PJA2 | -0.266 | 6.8E-14 | tarbase | MIMAT0000104 | 9867 | ENSG00000198961 | Degradome sequencing | negative |  | validated |
| 605 | hsa-mir-107 | PLSCR4 | -0.326 | 5.2E-21 | tarbase | MIMAT0000104 | 57088 | ENSG00000114698 | Degradome sequencing//Degradome sequencing//Degradome sequencing | positive |  | validated |
| 606 | hsa-mir-107 | PPP1CB | -0.260 | 2.6E-13 | tarbase | MIMAT0000104 | 5500 | ENSG00000213639 | Degradome sequencing | positive |  | validated |
| 607 | hsa-mir-107 | PRICKLE2 | -0.270 | 2.7E-14 | tarbase | MIMAT0000104 | 166336 | ENSG00000163637 | Degradome sequencing | negative |  | validated |
| 608 | hsa-mir-107 | PRKAA1 | -0.261 | 2.1E-13 | tarbase | MIMAT0000104 | 5562 | ENSG00000132356 | Degradome sequencing//Degradome sequencing//Degradome sequencing//Degradome sequencing//Degradome sequencing | positive |  | validated |
| 609 | hsa-mir-107 | PRKCA | -0.255 | 8.0E-13 | tarbase | MIMAT0000104 | 5578 | ENSG00000154229 | Degradome sequencing | positive |  | validated |
| 610 | hsa-mir-107 | PRKG1 | -0.284 | 9.6E-16 | tarbase | MIMAT0000104 | 5592 | ENSG00000185532 | Degradome sequencing | positive |  | validated |
| 611 | hsa-mir-107 | PTEN | -0.295 | 7.1E-17 | mirtarbase | MIMAT0000104 | 5728 | ENSG00000171862 | Western blot | Non-Functional MTI | 22593189 | validated |
| 612 | hsa-mir-107 | PTEN | -0.295 | 7.1E-17 | mirtarbase | MIMAT0000104 | 5728 | ENSG00000284792 | Western blot | Non-Functional MTI | 22593189 | validated |
| 613 | hsa-mir-107 | PTEN | -0.295 | 7.1E-17 | tarbase | MIMAT0000104 | 5728 | ENSG00000171862 | Degradome sequencing//Degradome sequencing | positive |  | validated |
| 614 | hsa-mir-107 | PTEN | -0.295 | 7.1E-17 | tarbase | MIMAT0000104 | 5728 | ENSG00000171862 | Degradome sequencing | negative |  | validated |
| 615 | hsa-mir-107 | PTGFRN | -0.280 | 2.7E-15 | tarbase | MIMAT0000104 | 5738 | ENSG00000134247 | Degradome sequencing//Degradome sequencing//Degradome sequencing | positive |  | validated |
| 616 | hsa-mir-107 | RAB11FIP2 | -0.259 | 3.5E-13 | tarbase | MIMAT0000104 | 22841 | ENSG00000107560 | Degradome sequencing//Degradome sequencing | positive |  | validated |
| 617 | hsa-mir-107 | RAB27B | -0.259 | 3.1E-13 | tarbase | MIMAT0000104 | 5874 | ENSG00000041353 | Degradome sequencing | negative |  | validated |
| 618 | hsa-mir-107 | RBFOX2 | -0.259 | 3.2E-13 | tarbase | MIMAT0000104 | 23543 | ENSG00000100320 | Degradome sequencing | positive |  | validated |
| 619 | hsa-mir-107 | RECK | -0.271 | 2.2E-14 | tarbase | MIMAT0000104 | 8434 | ENSG00000122707 | Degradome sequencing | positive |  | validated |
| 620 | hsa-mir-107 | RIN2 | -0.304 | 6.8E-18 | tarbase | MIMAT0000104 | 54453 | ENSG00000132669 | Degradome sequencing | positive |  | validated |
| 621 | hsa-mir-107 | RIN2 | -0.304 | 6.8E-18 | tarbase | MIMAT0000104 | 54453 | ENSG00000132669 | Degradome sequencing | negative |  | validated |
| 622 | hsa-mir-107 | RUNX1 | -0.251 | 1.8E-12 | tarbase | MIMAT0000104 | 861 | ENSG00000159216 | Degradome sequencing | positive |  | validated |
| 623 | hsa-mir-107 | RUNX1T1 | -0.270 | 2.1E-14 | mirtarbase | MIMAT0000104 | 862 | ENSG00000079102 | PAR-CLIP | Functional MTI (Weak) | 21572407 | validated |
| 624 | hsa-mir-107 | SAV1 | -0.267 | 5.5E-14 | tarbase | MIMAT0000104 | 60485 | ENSG00000151748 | Degradome sequencing//Degradome sequencing//Degradome sequencing | positive |  | validated |
| 625 | hsa-mir-107 | SEMA3C | -0.269 | 3.5E-14 | tarbase | MIMAT0000104 | 10512 | ENSG00000075223 | Degradome sequencing//Degradome sequencing | positive |  | validated |
| 626 | hsa-mir-107 | SEMA3D | -0.251 | 1.6E-12 | tarbase | MIMAT0000104 | 223117 | ENSG00000153993 | Degradome sequencing | positive |  | validated |
| 627 | hsa-mir-107 | SEMA6D | -0.257 | 4.9E-13 | tarbase | MIMAT0000104 | 80031 | ENSG00000137872 | Degradome sequencing | positive |  | validated |
| 628 | hsa-mir-107 | SH3BGRL2 | -0.252 | 1.1E-12 | tarbase | MIMAT0000104 | 83699 | ENSG00000198478 | Degradome sequencing//Degradome sequencing | positive |  | validated |
| 629 | hsa-mir-107 | SH3D19 | -0.277 | 6.2E-15 | tarbase | MIMAT0000104 | 152503 | ENSG00000109686 | Degradome sequencing | positive |  | validated |
| 630 | hsa-mir-107 | SLC17A5 | -0.275 | 1.1E-14 | tarbase | MIMAT0000104 | 26503 | ENSG00000119899 | Degradome sequencing | positive |  | validated |
| 631 | hsa-mir-107 | SLC17A5 | -0.275 | 1.1E-14 | tarbase | MIMAT0000104 | 26503 | ENSG00000119899 | Degradome sequencing | negative |  | validated |
| 632 | hsa-mir-107 | SLC30A7 | -0.251 | 1.7E-12 | mirtarbase | MIMAT0000104 | 148867 | ENSG00000162695 | PAR-CLIP | Functional MTI (Weak) | 20371350 | validated |
| 633 | hsa-mir-107 | SLC30A7 | -0.251 | 1.7E-12 | tarbase | MIMAT0000104 | 148867 | ENSG00000162695 | Degradome sequencing//Degradome sequencing | positive |  | validated |
| 634 | hsa-mir-107 | SLC35D1 | -0.276 | 7.7E-15 | tarbase | MIMAT0000104 | 23169 | ENSG00000116704 | Degradome sequencing | positive |  | validated |
| 635 | hsa-mir-107 | SLIT2 | -0.254 | 8.2E-13 | tarbase | MIMAT0000104 | 9353 | ENSG00000145147 | Degradome sequencing//Degradome sequencing//Degradome sequencing | positive |  | validated |
| 636 | hsa-mir-107 | SNRK | -0.253 | 1.3E-12 | tarbase | MIMAT0000104 | 54861 | ENSG00000163788 | Degradome sequencing//Degradome sequencing//Degradome sequencing//Degradome sequencing//Degradome sequencing | positive |  | validated |
| 637 | hsa-mir-107 | SOCS5 | -0.278 | 4.4E-15 | tarbase | MIMAT0000104 | 9655 | ENSG00000171150 | Degradome sequencing | negative |  | validated |
| 638 | hsa-mir-107 | STXBP5 | -0.258 | 3.7E-13 | tarbase | MIMAT0000104 | 134957 | ENSG00000164506 | Degradome sequencing | positive |  | validated |
| 639 | hsa-mir-107 | SYPL1 | -0.296 | 5.7E-17 | tarbase | MIMAT0000104 | 6856 | ENSG00000008282 | Degradome sequencing//Degradome sequencing//Degradome sequencing | positive |  | validated |
| 640 | hsa-mir-107 | SYPL1 | -0.296 | 5.7E-17 | tarbase | MIMAT0000104 | 6856 | ENSG00000008282 | Degradome sequencing | negative |  | validated |
| 641 | hsa-mir-107 | TANC1 | -0.283 | 1.2E-15 | tarbase | MIMAT0000104 | 85461 | ENSG00000115183 | Degradome sequencing | negative |  | validated |
| 642 | hsa-mir-107 | TBCEL | -0.264 | 1.2E-13 | tarbase | MIMAT0000104 | 219899 | ENSG00000154114 | Degradome sequencing | positive |  | validated |
| 643 | hsa-mir-107 | TJP1 | -0.274 | 1.1E-14 | mirtarbase | MIMAT0000104 | 7082 | ENSG00000104067 | PAR-CLIP | Functional MTI (Weak) | 21572407 | validated |
| 644 | hsa-mir-107 | TJP1 | -0.274 | 1.1E-14 | mirtarbase | MIMAT0000104 | 7082 | ENSG00000277401 | PAR-CLIP | Functional MTI (Weak) | 21572407 | validated |
| 645 | hsa-mir-107 | TMEM30A | -0.279 | 3.9E-15 | tarbase | MIMAT0000104 | 55754 | ENSG00000112697 | Degradome sequencing//Degradome sequencing//Degradome sequencing | positive |  | validated |
| 646 | hsa-mir-107 | TOR1AIP1 | -0.257 | 4.8E-13 | tarbase | MIMAT0000104 | 26092 | ENSG00000143337 | Degradome sequencing | positive |  | validated |
| 647 | hsa-mir-107 | UBE2J1 | -0.263 | 1.1E-13 | tarbase | MIMAT0000104 | 51465 | ENSG00000198833 | Degradome sequencing | positive |  | validated |
| 648 | hsa-mir-107 | UBE4A | -0.252 | 1.4E-12 | tarbase | MIMAT0000104 | 9354 | ENSG00000110344 | Degradome sequencing//Degradome sequencing | positive |  | validated |
| 649 | hsa-mir-107 | UHRF1BP1L | -0.254 | 9.0E-13 | tarbase | MIMAT0000104 | 23074 | ENSG00000111647 | Degradome sequencing | positive |  | validated |
| 650 | hsa-mir-107 | UTRN | -0.296 | 5.4E-17 | tarbase | MIMAT0000104 | 7402 | ENSG00000152818 | Degradome sequencing | positive |  | validated |
| 651 | hsa-mir-107 | VGLL4 | -0.253 | 1.1E-12 | tarbase | MIMAT0000104 | 9686 | ENSG00000144560 | Degradome sequencing | positive |  | validated |
| 652 | hsa-mir-107 | VGLL4 | -0.253 | 1.1E-12 | tarbase | MIMAT0000104 | 9686 | ENSG00000144560 | Degradome sequencing | negative |  | validated |
| 653 | hsa-mir-107 | VWA5A | -0.282 | 1.6E-15 | tarbase | MIMAT0000104 | 4013 | ENSG00000110002 | Degradome sequencing | negative |  | validated |
| 654 | hsa-mir-107 | WLS | -0.293 | 1.3E-16 | tarbase | MIMAT0000104 | 79971 | ENSG00000116729 | Degradome sequencing | positive |  | validated |
| 655 | hsa-mir-107 | WLS | -0.293 | 1.3E-16 | tarbase | MIMAT0000104 | 79971 | ENSG00000116729 | Degradome sequencing | negative |  | validated |
| 656 | hsa-mir-107 | WNT5A | -0.255 | 8.0E-13 | tarbase | MIMAT0000104 | 7474 | ENSG00000114251 | Degradome sequencing | negative |  | validated |
| 657 | hsa-mir-107 | ZBTB38 | -0.317 | 1.6E-19 | mirtarbase | MIMAT0000104 | 253461 | ENSG00000177311 | PAR-CLIP | Functional MTI (Weak) | 23592263 | validated |
| 658 | hsa-mir-107 | ZBTB38 | -0.317 | 1.6E-19 | tarbase | MIMAT0000104 | 253461 | ENSG00000177311 | Degradome sequencing | positive |  | validated |
| 659 | hsa-mir-107 | ZBTB38 | -0.317 | 1.6E-19 | tarbase | MIMAT0000104 | 253461 | ENSG00000177311 | Degradome sequencing | negative |  | validated |
| 660 | hsa-mir-107 | ZFHX4 | -0.255 | 7.0E-13 | tarbase | MIMAT0000104 | 79776 | ENSG00000091656 | Degradome sequencing//Degradome sequencing | positive |  | validated |
| 661 | hsa-mir-107 | ZNF277 | -0.261 | 2.3E-13 | tarbase | MIMAT0000104 | 11179 | ENSG00000198839 | Degradome sequencing | positive |  | validated |
| 662 | hsa-mir-107 | ZYG11B | -0.262 | 1.3E-13 | tarbase | MIMAT0000104 | 79699 | ENSG00000162378 | Degradome sequencing | positive |  | validated |
| 690 | hsa-mir-127 | FKBP4 | -0.264 | 1.1E-13 | tarbase | MIMAT0000446 | 2288 | ENSG00000004478 | Degradome sequencing | positive |  | validated |
| 694 | hsa-mir-182 | ACSL4 | -0.278 | 4.6E-15 | tarbase | MIMAT0000259 | 2182 | ENSG00000068366 | Degradome sequencing | positive |  | validated |
| 695 | hsa-mir-182 | ANXA1 | -0.257 | 5.3E-13 | tarbase | MIMAT0000259 | 301 | ENSG00000135046 | Degradome sequencing | negative |  | validated |
| 696 | hsa-mir-182 | CD180 | -0.293 | 1.3E-16 | tarbase | MIMAT0000259 | 4064 | ENSG00000134061 | Degradome sequencing | positive |  | validated |
| 697 | hsa-mir-182 | CORO1C | -0.303 | 8.4E-18 | tarbase | MIMAT0000259 | 23603 | ENSG00000110880 | Degradome sequencing | positive |  | validated |
| 698 | hsa-mir-182 | DCN | -0.269 | 3.6E-14 | tarbase | MIMAT0000259 | 1634 | ENSG00000011465 | Degradome sequencing | negative |  | validated |
| 699 | hsa-mir-182 | DEPDC7 | -0.254 | 8.0E-13 | tarbase | MIMAT0000259 | 91614 | ENSG00000121690 | Degradome sequencing | negative |  | validated |
| 700 | hsa-mir-182 | DIAPH2 | -0.272 | 2.0E-14 | tarbase | MIMAT0000259 | 1730 | ENSG00000147202 | Degradome sequencing | positive |  | validated |
| 701 | hsa-mir-182 | DIAPH2 | -0.272 | 2.0E-14 | tarbase | MIMAT0000259 | 1730 | ENSG00000147202 | Degradome sequencing | negative |  | validated |
| 702 | hsa-mir-182 | DZIP1 | -0.257 | 4.1E-13 | tarbase | MIMAT0000259 | 22873 | ENSG00000134874 | Degradome sequencing | negative |  | validated |
| 703 | hsa-mir-182 | FAM20A | -0.293 | 1.3E-16 | tarbase | MIMAT0000259 | 54757 | ENSG00000108950 | Degradome sequencing | negative |  | validated |
| 704 | hsa-mir-182 | FOXN3 | -0.264 | 1.2E-13 | tarbase | MIMAT0000259 | 1112 | ENSG00000053254 | Degradome sequencing | positive |  | validated |
| 705 | hsa-mir-182 | GPNMB | -0.305 | 5.5E-18 | tarbase | MIMAT0000259 | 10457 | ENSG00000136235 | Degradome sequencing | positive |  | validated |
| 706 | hsa-mir-182 | HCK | -0.252 | 1.4E-12 | tarbase | MIMAT0000259 | 3055 | ENSG00000101336 | Degradome sequencing | positive |  | validated |
| 707 | hsa-mir-182 | HS3ST3A1 | -0.270 | 3.2E-14 | tarbase | MIMAT0000259 | 9955 | ENSG00000153976 | Degradome sequencing | positive |  | validated |
| 708 | hsa-mir-182 | HVCN1 | -0.251 | 1.6E-12 | tarbase | MIMAT0000259 | 84329 | ENSG00000122986 | Degradome sequencing | positive |  | validated |
| 709 | hsa-mir-182 | IFI16 | -0.290 | 2.4E-16 | tarbase | MIMAT0000259 | 3428 | ENSG00000163565 | Degradome sequencing | negative |  | validated |
| 710 | hsa-mir-182 | IL10RA | -0.257 | 5.2E-13 | tarbase | MIMAT0000259 | 3587 | ENSG00000110324 | Degradome sequencing | positive |  | validated |
| 711 | hsa-mir-182 | IL15RA | -0.292 | 1.6E-16 | tarbase | MIMAT0000259 | 3601 | ENSG00000134470 | Degradome sequencing | positive |  | validated |
| 712 | hsa-mir-182 | IL21R | -0.265 | 7.7E-14 | tarbase | MIMAT0000259 | 50615 | ENSG00000103522 | Degradome sequencing | positive |  | validated |
| 713 | hsa-mir-182 | IL7 | -0.261 | 1.9E-13 | tarbase | MIMAT0000259 | 3574 | ENSG00000104432 | Degradome sequencing | negative |  | validated |
| 714 | hsa-mir-182 | ITGB2 | -0.263 | 1.2E-13 | tarbase | MIMAT0000259 | 3689 | ENSG00000160255 | Degradome sequencing | positive |  | validated |
| 715 | hsa-mir-182 | LILRA6 | -0.261 | 1.9E-13 | tarbase | MIMAT0000259 | 79168 | ENSG00000244482 | Degradome sequencing | positive |  | validated |
| 716 | hsa-mir-182 | MAP7D3 | -0.262 | 1.4E-13 | tarbase | MIMAT0000259 | 79649 | ENSG00000129680 | Degradome sequencing | positive |  | validated |
| 717 | hsa-mir-182 | MORF4L1 | -0.278 | 4.7E-15 | mirtarbase | MIMAT0000259 | 10933 | ENSG00000185787 | PAR-CLIP | Functional MTI (Weak) | 21572407 | validated |
| 718 | hsa-mir-182 | MORF4L1 | -0.278 | 4.7E-15 | mirtarbase | MIMAT0000259 | 10933 | ENSG00000185787 | PAR-CLIP | Functional MTI (Weak) | 27292025 | validated |
| 719 | hsa-mir-182 | MORF4L1 | -0.278 | 4.7E-15 | tarbase | MIMAT0000259 | 10933 | ENSG00000185787 | Degradome sequencing | positive |  | validated |
| 720 | hsa-mir-182 | MOXD1 | -0.257 | 5.1E-13 | tarbase | MIMAT0000259 | 26002 | ENSG00000079931 | Degradome sequencing | negative |  | validated |
| 721 | hsa-mir-182 | MSN | -0.259 | 3.5E-13 | tarbase | MIMAT0000259 | 4478 | ENSG00000147065 | Degradome sequencing | positive |  | validated |
| 722 | hsa-mir-182 | NR3C1 | -0.262 | 1.8E-13 | mirtarbase | MIMAT0000259 | 2908 | ENSG00000113580 | HITS-CLIP | Functional MTI (Weak) | 23313552 | validated |
| 723 | hsa-mir-182 | NR3C1 | -0.262 | 1.8E-13 | tarbase | MIMAT0000259 | 2908 | ENSG00000113580 | Degradome sequencing | positive |  | validated |
| 724 | hsa-mir-182 | NR3C1 | -0.262 | 1.8E-13 | tarbase | MIMAT0000259 | 2908 | ENSG00000113580 | Degradome sequencing | negative |  | validated |
| 725 | hsa-mir-182 | PKD2 | -0.271 | 2.4E-14 | tarbase | MIMAT0000259 | 5311 | ENSG00000118762 | Degradome sequencing | positive |  | validated |
| 726 | hsa-mir-182 | PKD2 | -0.271 | 2.4E-14 | tarbase | MIMAT0000259 | 5311 | ENSG00000118762 | Degradome sequencing | negative |  | validated |
| 727 | hsa-mir-182 | PLS3 | -0.298 | 3.6E-17 | tarbase | MIMAT0000259 | 5358 | ENSG00000102024 | Degradome sequencing | positive |  | validated |
| 728 | hsa-mir-182 | RECK | -0.255 | 7.2E-13 | mirtarbase | MIMAT0000259 | 8434 | ENSG00000122707 | Luciferase reporter assay//qRT-PCR//Western blot | Functional MTI | 23333633 | validated |
| 729 | hsa-mir-182 | RECK | -0.255 | 7.2E-13 | mirtarbase | MIMAT0000259 | 8434 | ENSG00000122707 | Luciferase reporter assay | Functional MTI | 23226455 | validated |
| 730 | hsa-mir-182 | RECK | -0.255 | 7.2E-13 | mirtarbase | MIMAT0000259 | 8434 | ENSG00000122707 | Luciferase reporter assay | Functional MTI | 23383207 | validated |
| 731 | hsa-mir-182 | RECK | -0.255 | 7.2E-13 | mirtarbase | MIMAT0000259 | 8434 | ENSG00000122707 | PAR-CLIP | Functional MTI (Weak) | 21572407 | validated |
| 732 | hsa-mir-182 | RECK | -0.255 | 7.2E-13 | mirtarbase | MIMAT0000259 | 8434 | ENSG00000122707 | qRT-PCR//Western blot | Functional MTI | 26609496 | validated |
| 733 | hsa-mir-182 | RECK | -0.255 | 7.2E-13 | tarbase | MIMAT0000259 | 8434 | ENSG00000122707 | Degradome sequencing//Degradome sequencing//Degradome sequencing//Degradome sequencing//Degradome sequencing//Degradome sequencing//Degradome sequencing//Degrad | positive |  | validated |
| 734 | hsa-mir-182 | SIGLEC1 | -0.250 | 2.0E-12 | tarbase | MIMAT0000259 | 6614 | ENSG00000088827 | Degradome sequencing | positive |  | validated |
| 735 | hsa-mir-182 | SIGLEC9 | -0.264 | 9.5E-14 | tarbase | MIMAT0000259 | 27180 | ENSG00000129450 | Degradome sequencing | positive |  | validated |
| 736 | hsa-mir-182 | SLC35G1 | -0.255 | 8.4E-13 | tarbase | MIMAT0000259 | 159371 | ENSG00000176273 | Degradome sequencing | negative |  | validated |
| 737 | hsa-mir-182 | SLC36A1 | -0.273 | 1.5E-14 | tarbase | MIMAT0000259 | 206358 | ENSG00000123643 | Degradome sequencing | positive |  | validated |
| 738 | hsa-mir-182 | SLFN11 | -0.252 | 1.4E-12 | tarbase | MIMAT0000259 | 91607 | ENSG00000172716 | Degradome sequencing | positive |  | validated |
| 739 | hsa-mir-182 | SNX2 | -0.288 | 4.5E-16 | tarbase | MIMAT0000259 | 6643 | ENSG00000205302 | Degradome sequencing | positive |  | validated |
| 740 | hsa-mir-182 | SRGAP2 | -0.251 | 1.6E-12 | tarbase | MIMAT0000259 | 23380 | ENSG00000266028 | Degradome sequencing | positive |  | validated |
| 741 | hsa-mir-182 | SRGAP2 | -0.251 | 1.6E-12 | tarbase | MIMAT0000259 | 23380 | ENSG00000266028 | Degradome sequencing | negative |  | validated |
| 742 | hsa-mir-182 | SRGN | -0.256 | 5.6E-13 | tarbase | MIMAT0000259 | 5552 | ENSG00000122862 | Degradome sequencing | positive |  | validated |
| 743 | hsa-mir-182 | STARD4 | -0.252 | 1.4E-12 | tarbase | MIMAT0000259 | 134429 | ENSG00000164211 | Degradome sequencing | negative |  | validated |
| 744 | hsa-mir-182 | VGLL3 | -0.254 | 9.6E-13 | tarbase | MIMAT0000259 | 389136 | ENSG00000206538 | Degradome sequencing | positive |  | validated |
| 761 | hsa-mir-210 | ABCA6 | -0.420 | 2.2E-34 | tarbase | MIMAT0000267 | 23460 | ENSG00000154262 | Degradome sequencing | positive |  | validated |
| 762 | hsa-mir-210 | ACADL | -0.419 | 4.2E-34 | tarbase | MIMAT0000267 | 33 | ENSG00000115361 | Degradome sequencing | positive |  | validated |
| 763 | hsa-mir-210 | ACADS | -0.370 | 0 | tarbase | MIMAT0000267 | 35 | ENSG00000122971 | Degradome sequencing | positive |  | validated |
| 764 | hsa-mir-210 | ACCS | -0.312 | 8.4E-19 | tarbase | MIMAT0000267 | 84680 | ENSG00000110455 | Degradome sequencing | positive |  | validated |
| 765 | hsa-mir-210 | ACSL5 | -0.255 | 8.4E-13 | tarbase | MIMAT0000267 | 51703 | ENSG00000197142 | Degradome sequencing | positive |  | validated |
| 766 | hsa-mir-210 | ADH1A | -0.440 | 6.3E-38 | tarbase | MIMAT0000267 | 124 | ENSG00000187758 | Degradome sequencing | positive |  | validated |
| 767 | hsa-mir-210 | ADH1C | -0.450 | 8.6E-40 | tarbase | MIMAT0000267 | 126 | ENSG00000248144 | Degradome sequencing | positive |  | validated |
| 768 | hsa-mir-210 | ADRA2A | -0.260 | 2.5E-13 | tarbase | MIMAT0000267 | 150 | ENSG00000150594 | Degradome sequencing | positive |  | validated |
| 769 | hsa-mir-210 | AFAP1L2 | -0.253 | 1.2E-12 | tarbase | MIMAT0000267 | 84632 | ENSG00000169129 | Degradome sequencing | negative |  | validated |
| 770 | hsa-mir-210 | AHSA2P | -0.258 | 4.4E-13 | tarbase | MIMAT0000267 |  | ENSG00000173209 | Degradome sequencing | positive |  | validated |
| 771 | hsa-mir-210 | AKAP12 | -0.295 | 6.8E-17 | tarbase | MIMAT0000267 | 9590 | ENSG00000131016 | Degradome sequencing | positive |  | validated |
| 772 | hsa-mir-210 | AKR1C3 | -0.365 | 9.4E-26 | tarbase | MIMAT0000267 | 8644 | ENSG00000196139 | Degradome sequencing//Degradome sequencing | positive |  | validated |
| 773 | hsa-mir-210 | ALDH1A1 | -0.439 | 0 | tarbase | MIMAT0000267 | 216 | ENSG00000165092 | Degradome sequencing//Degradome sequencing | positive |  | validated |
| 774 | hsa-mir-210 | ALDH3A1 | -0.359 | 7.6E-25 | tarbase | MIMAT0000267 | 218 | ENSG00000108602 | Degradome sequencing//Degradome sequencing | positive |  | validated |
| 775 | hsa-mir-210 | ALG13 | -0.277 | 6.3E-15 | tarbase | MIMAT0000267 | 79868 | ENSG00000101901 | Degradome sequencing | negative |  | validated |
| 776 | hsa-mir-210 | ANXA1 | -0.281 | 2.3E-15 | tarbase | MIMAT0000267 | 301 | ENSG00000135046 | Degradome sequencing//Degradome sequencing | positive |  | validated |
| 777 | hsa-mir-210 | ANXA13 | -0.301 | 1.1E-17 | tarbase | MIMAT0000267 | 312 | ENSG00000104537 | Degradome sequencing | positive |  | validated |
| 778 | hsa-mir-210 | APOD | -0.337 | 0 | tarbase | MIMAT0000267 | 347 | ENSG00000189058 | Degradome sequencing | positive |  | validated |
| 779 | hsa-mir-210 | APOLD1 | -0.353 | 0 | tarbase | MIMAT0000267 | 81575 | ENSG00000178878 | Degradome sequencing//Degradome sequencing | negative |  | validated |
| 780 | hsa-mir-210 | ARGLU1 | -0.318 | 1.3E-19 | tarbase | MIMAT0000267 | 55082 | ENSG00000134884 | Degradome sequencing | positive |  | validated |
| 781 | hsa-mir-210 | ARHGEF3 | -0.294 | 1.0E-16 | tarbase | MIMAT0000267 | 50650 | ENSG00000163947 | Degradome sequencing | negative |  | validated |
| 782 | hsa-mir-210 | BBOX1 | -0.333 | 1.8E-21 | tarbase | MIMAT0000267 | 8424 | ENSG00000129151 | Degradome sequencing | positive |  | validated |
| 783 | hsa-mir-210 | BCHE | -0.283 | 1.2E-15 | tarbase | MIMAT0000267 | 590 | ENSG00000114200 | Degradome sequencing | positive |  | validated |
| 784 | hsa-mir-210 | BHLHE41 | -0.304 | 7.6E-18 | tarbase | MIMAT0000267 | 79365 | ENSG00000123095 | Degradome sequencing | positive |  | validated |
| 785 | hsa-mir-210 | BICRA | -0.316 | 2.8E-19 | tarbase | MIMAT0000267 | 29998 | ENSG00000063169 | Degradome sequencing | positive |  | validated |
| 786 | hsa-mir-210 | BMP2 | -0.315 | 3.1E-19 | tarbase | MIMAT0000267 | 650 | ENSG00000125845 | Degradome sequencing | positive |  | validated |
| 787 | hsa-mir-210 | BMP6 | -0.484 | 1.5E-46 | tarbase | MIMAT0000267 | 654 | ENSG00000153162 | Degradome sequencing | positive |  | validated |
| 788 | hsa-mir-210 | BORCS7 | -0.269 | 3.5E-14 | tarbase | MIMAT0000267 | 119032 | ENSG00000166275 | Degradome sequencing | positive |  | validated |
| 789 | hsa-mir-210 | BTG2 | -0.472 | 0 | tarbase | MIMAT0000267 | 7832 | ENSG00000159388 | Degradome sequencing | positive |  | validated |
| 790 | hsa-mir-210 | C1RL | -0.267 | 5.0E-14 | tarbase | MIMAT0000267 | 51279 | ENSG00000139178 | Degradome sequencing | positive |  | validated |
| 791 | hsa-mir-210 | CALCRL | -0.347 | 0 | tarbase | MIMAT0000267 | 10203 | ENSG00000064989 | Degradome sequencing | positive |  | validated |
| 792 | hsa-mir-210 | CALML4 | -0.304 | 7.3E-18 | tarbase | MIMAT0000267 | 91860 | ENSG00000129007 | Degradome sequencing | positive |  | validated |
| 793 | hsa-mir-210 | CAPN5 | -0.288 | 4.6E-16 | tarbase | MIMAT0000267 | 726 | ENSG00000149260 | Degradome sequencing | positive |  | validated |
| 794 | hsa-mir-210 | CAV1 | -0.440 | 0 | tarbase | MIMAT0000267 | 857 | ENSG00000105974 | Degradome sequencing//Degradome sequencing | positive |  | validated |
| 795 | hsa-mir-210 | CCDC50 | -0.308 | 2.6E-18 | tarbase | MIMAT0000267 | 152137 | ENSG00000152492 | Degradome sequencing | positive |  | validated |
| 796 | hsa-mir-210 | CCDC80 | -0.332 | 0 | tarbase | MIMAT0000267 | 151887 | ENSG00000091986 | Degradome sequencing | positive |  | validated |
| 797 | hsa-mir-210 | CCL5 | -0.253 | 1.2E-12 | tarbase | MIMAT0000267 | 6352 | ENSG00000271503 | Degradome sequencing | positive |  | validated |
| 798 | hsa-mir-210 | CCN2 | -0.299 | 2.5E-17 | tarbase | MIMAT0000267 | 1490 | ENSG00000118523 | Degradome sequencing | negative |  | validated |
| 799 | hsa-mir-210 | CCNDBP1 | -0.327 | 4.3E-21 | tarbase | MIMAT0000267 | 23582 | ENSG00000166946 | Degradome sequencing | positive |  | validated |
| 800 | hsa-mir-210 | CD8A | -0.291 | 2.1E-16 | tarbase | MIMAT0000267 | 925 | ENSG00000153563 | Degradome sequencing | positive |  | validated |
| 801 | hsa-mir-210 | CDH17 | -0.416 | 1.1E-33 | tarbase | MIMAT0000267 | 1015 | ENSG00000079112 | Degradome sequencing//Degradome sequencing | positive |  | validated |
| 802 | hsa-mir-210 | CDK10 | -0.252 | 1.6E-12 | mirtarbase | MIMAT0000267 | 8558 | ENSG00000185324 | immunoprecipitaion//Microarray//qRT-PCR | Functional MTI (Weak) | 19826008 | validated |
| 803 | hsa-mir-210 | CDKN1C | -0.367 | 0 | tarbase | MIMAT0000267 | 1028 | ENSG00000129757 | Degradome sequencing | positive |  | validated |
| 804 | hsa-mir-210 | CFD | -0.338 | 0 | tarbase | MIMAT0000267 | 1675 | ENSG00000197766 | Degradome sequencing | positive |  | validated |
| 805 | hsa-mir-210 | CFI | -0.426 | 0 | tarbase | MIMAT0000267 | 3426 | ENSG00000205403 | Degradome sequencing | positive |  | validated |
| 806 | hsa-mir-210 | CHST7 | -0.484 | 1.2E-46 | tarbase | MIMAT0000267 | 56548 | ENSG00000147119 | Degradome sequencing | negative |  | validated |
| 807 | hsa-mir-210 | CIDEC | -0.465 | 9.6E-43 | tarbase | MIMAT0000267 | 63924 | ENSG00000187288 | Degradome sequencing | positive |  | validated |
| 808 | hsa-mir-210 | CLK1 | -0.305 | 5.0E-18 | tarbase | MIMAT0000267 | 1195 | ENSG00000013441 | Degradome sequencing | positive |  | validated |
| 809 | hsa-mir-210 | COL17A1 | -0.426 | 1.8E-35 | tarbase | MIMAT0000267 | 1308 | ENSG00000065618 | Degradome sequencing | positive |  | validated |
| 810 | hsa-mir-210 | CPVL | -0.260 | 2.9E-13 | tarbase | MIMAT0000267 | 54504 | ENSG00000106066 | Degradome sequencing//Degradome sequencing | positive |  | validated |
| 811 | hsa-mir-210 | CSF2RA | -0.278 | 4.0E-15 | tarbase | MIMAT0000267 | 1438 | ENSG00000198223 | Degradome sequencing | positive |  | validated |
| 812 | hsa-mir-210 | CSRNP1 | -0.466 | 0 | tarbase | MIMAT0000267 | 64651 | ENSG00000144655 | Degradome sequencing | negative |  | validated |
| 813 | hsa-mir-210 | CST2 | -0.341 | 1.8E-22 | tarbase | MIMAT0000267 | 1470 | ENSG00000170369 | Degradome sequencing | positive |  | validated |
| 814 | hsa-mir-210 | CST3 | -0.347 | 0 | tarbase | MIMAT0000267 | 1471 | ENSG00000101439 | Degradome sequencing | positive |  | validated |
| 815 | hsa-mir-210 | CTSH | -0.307 | 3.2E-18 | tarbase | MIMAT0000267 | 1512 | ENSG00000103811 | Degradome sequencing | positive |  | validated |
| 816 | hsa-mir-210 | CXCL2 | -0.398 | 1.0E-30 | tarbase | MIMAT0000267 | 2920 | ENSG00000081041 | Degradome sequencing | positive |  | validated |
| 817 | hsa-mir-210 | CXCL6 | -0.252 | 1.1E-12 | tarbase | MIMAT0000267 | 6372 | ENSG00000124875 | Degradome sequencing | negative |  | validated |
| 818 | hsa-mir-210 | CYP4F12 | -0.316 | 2.6E-19 | tarbase | MIMAT0000267 | 66002 | ENSG00000186204 | Degradome sequencing | positive |  | validated |
| 819 | hsa-mir-210 | DHRS3 | -0.268 | 4.2E-14 | tarbase | MIMAT0000267 | 9249 | ENSG00000162496 | Degradome sequencing | positive |  | validated |
| 820 | hsa-mir-210 | DUSP6 | -0.281 | 2.2E-15 | tarbase | MIMAT0000267 | 1848 | ENSG00000139318 | Degradome sequencing | negative |  | validated |
| 821 | hsa-mir-210 | EDN1 | -0.300 | 2.1E-17 | tarbase | MIMAT0000267 | 1906 | ENSG00000078401 | Degradome sequencing | positive |  | validated |
| 822 | hsa-mir-210 | EFNB2 | -0.278 | 5.0E-15 | tarbase | MIMAT0000267 | 1948 | ENSG00000125266 | Degradome sequencing | positive |  | validated |
| 823 | hsa-mir-210 | EGR1 | -0.435 | 0 | tarbase | MIMAT0000267 | 1958 | ENSG00000120738 | Degradome sequencing | positive |  | validated |
| 824 | hsa-mir-210 | EGR1 | -0.435 | 0 | tarbase | MIMAT0000267 | 1958 | ENSG00000120738 | Degradome sequencing | negative |  | validated |
| 825 | hsa-mir-210 | EHD2 | -0.305 | 5.2E-18 | mirtarbase | MIMAT0000267 | 30846 | ENSG00000024422 | Luciferase reporter assay//Microarray//qRT-PCR//Western blot | Functional MTI | 23028679 | validated |
| 826 | hsa-mir-210 | EHD2 | -0.305 | 5.2E-18 | tarbase | MIMAT0000267 | 30846 | ENSG00000024422 | Degradome sequencing | positive |  | validated |
| 827 | hsa-mir-210 | ELK3 | -0.313 | 5.6E-19 | mirtarbase | MIMAT0000267 | 2004 | ENSG00000111145 | immunoprecipitaion//Microarray//qRT-PCR | Functional MTI (Weak) | 19826008 | validated |
| 828 | hsa-mir-210 | EMCN | -0.610 | 0 | tarbase | MIMAT0000267 | 51705 | ENSG00000164035 | Degradome sequencing | positive |  | validated |
| 829 | hsa-mir-210 | EPHA2 | -0.309 | 1.8E-18 | tarbase | MIMAT0000267 | 1969 | ENSG00000142627 | Degradome sequencing | positive |  | validated |
| 830 | hsa-mir-210 | EPHB6 | -0.367 | 0 | tarbase | MIMAT0000267 | 2051 | ENSG00000106123 | Degradome sequencing | negative |  | validated |
| 831 | hsa-mir-210 | EPHX2 | -0.310 | 1.5E-18 | tarbase | MIMAT0000267 | 2053 | ENSG00000120915 | Degradome sequencing | positive |  | validated |
| 832 | hsa-mir-210 | ERCC1 | -0.292 | 1.8E-16 | tarbase | MIMAT0000267 | 2067 | ENSG00000012061 | Degradome sequencing | negative |  | validated |
| 833 | hsa-mir-210 | ETS1 | -0.344 | 0 | tarbase | MIMAT0000267 | 2113 | ENSG00000134954 | Degradome sequencing | negative |  | validated |
| 834 | hsa-mir-210 | ETS2 | -0.433 | 0 | tarbase | MIMAT0000267 | 2114 | ENSG00000157557 | Degradome sequencing | positive |  | validated |
| 835 | hsa-mir-210 | F3 | -0.338 | 0 | tarbase | MIMAT0000267 | 2152 | ENSG00000117525 | Degradome sequencing | negative |  | validated |
| 836 | hsa-mir-210 | FBXL8 | -0.300 | 1.5E-17 | tarbase | MIMAT0000267 | 55336 | ENSG00000135722 | Degradome sequencing//Degradome sequencing | negative |  | validated |
| 837 | hsa-mir-210 | FBXO31 | -0.316 | 2.3E-19 | tarbase | MIMAT0000267 | 79791 | ENSG00000103264 | Degradome sequencing | positive |  | validated |
| 838 | hsa-mir-210 | FGF18 | -0.400 | 4.5E-31 | tarbase | MIMAT0000267 | 8817 | ENSG00000156427 | Degradome sequencing | positive |  | validated |
| 839 | hsa-mir-210 | FGF2 | -0.449 | 0 | tarbase | MIMAT0000267 | 2247 | ENSG00000138685 | Degradome sequencing | positive |  | validated |
| 840 | hsa-mir-210 | FGFBP1 | -0.275 | 6.8E-15 | tarbase | MIMAT0000267 | 9982 | ENSG00000137440 | Degradome sequencing | positive |  | validated |
| 841 | hsa-mir-210 | FGL2 | -0.284 | 1.2E-15 | tarbase | MIMAT0000267 | 10875 | ENSG00000127951 | Degradome sequencing | negative |  | validated |
| 842 | hsa-mir-210 | FHL1 | -0.353 | 0 | tarbase | MIMAT0000267 | 2273 | ENSG00000022267 | Degradome sequencing | negative |  | validated |
| 843 | hsa-mir-210 | FLT4 | -0.475 | 9.2E-45 | tarbase | MIMAT0000267 | 2324 | ENSG00000037280 | Degradome sequencing | positive |  | validated |
| 844 | hsa-mir-210 | FOXO1 | -0.291 | 2.2E-16 | tarbase | MIMAT0000267 | 2308 | ENSG00000150907 | Degradome sequencing | negative |  | validated |
| 845 | hsa-mir-210 | FYN | -0.309 | 2.0E-18 | tarbase | MIMAT0000267 | 2534 | ENSG00000010810 | Degradome sequencing | positive |  | validated |
| 846 | hsa-mir-210 | G0S2 | -0.352 | 0 | tarbase | MIMAT0000267 | 50486 | ENSG00000123689 | Degradome sequencing | positive |  | validated |
| 847 | hsa-mir-210 | GBA2 | -0.261 | 2.4E-13 | tarbase | MIMAT0000267 | 57704 | ENSG00000070610 | Degradome sequencing | positive |  | validated |
| 848 | hsa-mir-210 | GIPC2 | -0.566 | 0 | tarbase | MIMAT0000267 | 54810 | ENSG00000137960 | Degradome sequencing | positive |  | validated |
| 849 | hsa-mir-210 | GLYCTK | -0.366 | 6.1E-26 | tarbase | MIMAT0000267 | 132158 | ENSG00000168237 | Degradome sequencing | positive |  | validated |
| 850 | hsa-mir-210 | GPAT3 | -0.271 | 2.0E-14 | tarbase | MIMAT0000267 | 84803 | ENSG00000138678 | Degradome sequencing//Degradome sequencing | negative |  | validated |
| 851 | hsa-mir-210 | GPCPD1 | -0.301 | 1.7E-17 | tarbase | MIMAT0000267 | 56261 | ENSG00000125772 | Degradome sequencing | positive |  | validated |
| 852 | hsa-mir-210 | GSTK1 | -0.305 | 6.0E-18 | tarbase | MIMAT0000267 | 373156 | ENSG00000197448 | Degradome sequencing | positive |  | validated |
| 853 | hsa-mir-210 | HAND2 | -0.276 | 5.4E-15 | tarbase | MIMAT0000267 | 9464 | ENSG00000164107 | Degradome sequencing | negative |  | validated |
| 854 | hsa-mir-210 | HEY1 | -0.272 | 2.0E-14 | tarbase | MIMAT0000267 | 23462 | ENSG00000164683 | Degradome sequencing | negative |  | validated |
| 855 | hsa-mir-210 | HIF3A | -0.420 | 2.2E-34 | mirtarbase | MIMAT0000267 | 64344 | ENSG00000124440 | Luciferase reporter assay//Western blot//qRT-PCR | Functional MTI | 24927770 | validated |
| 856 | hsa-mir-210 | HNRNPA0 | -0.256 | 6.7E-13 | tarbase | MIMAT0000267 | 10949 | ENSG00000177733 | Degradome sequencing | positive |  | validated |
| 857 | hsa-mir-210 | HOXA3 | -0.367 | 4.4E-26 | mirtarbase | MIMAT0000267 | 3200 | ENSG00000105997 | immunoprecipitaion//Microarray//qRT-PCR | Functional MTI (Weak) | 19826008 | validated |
| 858 | hsa-mir-210 | HOXA9 | -0.287 | 6.0E-16 | mirtarbase | MIMAT0000267 | 3205 | ENSG00000078399 | Luciferase reporter assay | Functional MTI | 19782034 | validated |
| 859 | hsa-mir-210 | HOXA9 | -0.287 | 6.0E-16 | tarbase | MIMAT0000267 | 3205 | ENSG00000078399 | Degradome sequencing | positive |  | validated |
| 860 | hsa-mir-210 | HYAL1 | -0.511 | 0 | tarbase | MIMAT0000267 | 3373 | ENSG00000114378 | Degradome sequencing | positive |  | validated |
| 861 | hsa-mir-210 | ID1 | -0.388 | 0 | tarbase | MIMAT0000267 | 3397 | ENSG00000125968 | Degradome sequencing//Degradome sequencing | positive |  | validated |
| 862 | hsa-mir-210 | ID3 | -0.313 | 6.9E-19 | tarbase | MIMAT0000267 | 3399 | ENSG00000117318 | Degradome sequencing | positive |  | validated |
| 863 | hsa-mir-210 | IGF1 | -0.508 | 0 | tarbase | MIMAT0000267 | 3479 | ENSG00000017427 | Degradome sequencing | positive |  | validated |
| 864 | hsa-mir-210 | IGF2 | -0.433 | 0 | tarbase | MIMAT0000267 | 3481 | ENSG00000167244 | Degradome sequencing//Degradome sequencing | positive |  | validated |
| 865 | hsa-mir-210 | IGFBP7 | -0.364 | 0 | tarbase | MIMAT0000267 | 3490 | ENSG00000163453 | Degradome sequencing | positive |  | validated |
| 866 | hsa-mir-210 | IL17D | -0.370 | 2.2E-26 | tarbase | MIMAT0000267 | 53342 | ENSG00000172458 | Degradome sequencing//Degradome sequencing | positive |  | validated |
| 867 | hsa-mir-210 | IL18R1 | -0.383 | 2.0E-28 | tarbase | MIMAT0000267 | 8809 | ENSG00000115604 | Degradome sequencing | positive |  | validated |
| 868 | hsa-mir-210 | INPP1 | -0.399 | 0 | tarbase | MIMAT0000267 | 3628 | ENSG00000151689 | Degradome sequencing | negative |  | validated |
| 869 | hsa-mir-210 | IRS2 | -0.257 | 4.5E-13 | tarbase | MIMAT0000267 | 8660 | ENSG00000185950 | Degradome sequencing | negative |  | validated |
| 870 | hsa-mir-210 | ISCU | -0.435 | 0 | mirtarbase | MIMAT0000267 | 23479 | ENSG00000136003 | immunoprecipitaion//Microarray//qRT-PCR | Functional MTI (Weak) | 19826008 | validated |
| 871 | hsa-mir-210 | ISCU | -0.435 | 0 | mirtarbase | MIMAT0000267 | 23479 | ENSG00000136003 | Luciferase reporter assay//qRT-PCR//Western blot | Functional MTI | 19808020 | validated |
| 872 | hsa-mir-210 | ISCU | -0.435 | 0 | mirtarbase | MIMAT0000267 | 23479 | ENSG00000136003 | Luciferase reporter assay//Western blot | Functional MTI | 21801864 | validated |
| 873 | hsa-mir-210 | ISCU | -0.435 | 0 | mirtarbase | MIMAT0000267 | 23479 | ENSG00000136003 | CLASH | Functional MTI (Weak) | 23622248 | validated |
| 874 | hsa-mir-210 | ISCU | -0.435 | 0 | mirtarbase | MIMAT0000267 | 23479 | ENSG00000136003 | qRT-PCR | Functional MTI (Weak) | 22840297 | validated |
| 875 | hsa-mir-210 | ISCU | -0.435 | 0 | mirtarbase | MIMAT0000267 | 23479 | ENSG00000136003 | HITS-CLIP | Functional MTI (Weak) | 22473208 | validated |
| 876 | hsa-mir-210 | ISCU | -0.435 | 0 | mirtarbase | MIMAT0000267 | 23479 | ENSG00000136003 | PAR-CLIP | Functional MTI (Weak) | 27292025 | validated |
| 877 | hsa-mir-210 | ISCU | -0.435 | 0 | tarbase | MIMAT0000267 | 23479 | ENSG00000136003 | Degradome sequencing//Degradome sequencing//Degradome sequencing//Degradome sequencing//Degradome sequencing//Degradome sequencing//Degradome sequencing//Degrad | positive |  | validated |
| 878 | hsa-mir-210 | ITM2C | -0.253 | 1.2E-12 | tarbase | MIMAT0000267 | 81618 | ENSG00000135916 | Degradome sequencing | positive |  | validated |
| 879 | hsa-mir-210 | JAG2 | -0.342 | 0 | tarbase | MIMAT0000267 | 3714 | ENSG00000184916 | Degradome sequencing | positive |  | validated |
| 880 | hsa-mir-210 | JUNB | -0.434 | 0 | tarbase | MIMAT0000267 | 3726 | ENSG00000171223 | Degradome sequencing | positive |  | validated |
| 881 | hsa-mir-210 | JUNB | -0.434 | 0 | tarbase | MIMAT0000267 | 3726 | ENSG00000171223 | Degradome sequencing | negative |  | validated |
| 882 | hsa-mir-210 | KCNA1 | -0.418 | 4.4E-34 | tarbase | MIMAT0000267 | 3736 | ENSG00000111262 | Degradome sequencing | positive |  | validated |
| 883 | hsa-mir-210 | KCNK10 | -0.392 | 8.2E-30 | mirtarbase | MIMAT0000267 | 54207 | ENSG00000100433 | HITS-CLIP | Functional MTI (Weak) | 27418678 | validated |
| 884 | hsa-mir-210 | KLRG1 | -0.400 | 0 | tarbase | MIMAT0000267 | 10219 | ENSG00000139187 | Degradome sequencing | negative |  | validated |
| 885 | hsa-mir-210 | KRBOX4 | -0.344 | 7.7E-23 | tarbase | MIMAT0000267 | 55634 | ENSG00000147121 | Degradome sequencing | positive |  | validated |
| 886 | hsa-mir-210 | LAMB3 | -0.299 | 2.9E-17 | tarbase | MIMAT0000267 | 3914 | ENSG00000196878 | Degradome sequencing | positive |  | validated |
| 887 | hsa-mir-210 | LCK | -0.302 | 1.2E-17 | tarbase | MIMAT0000267 | 3932 | ENSG00000182866 | Degradome sequencing//Degradome sequencing | negative |  | validated |
| 888 | hsa-mir-210 | LIFR | -0.325 | 8.3E-21 | tarbase | MIMAT0000267 | 3977 | ENSG00000113594 | Degradome sequencing | positive |  | validated |
| 889 | hsa-mir-210 | LIPE | -0.439 | 0 | tarbase | MIMAT0000267 | 3991 | ENSG00000079435 | Degradome sequencing | negative |  | validated |
| 890 | hsa-mir-210 | LIPG | -0.352 | 6.4E-24 | tarbase | MIMAT0000267 | 9388 | ENSG00000101670 | Degradome sequencing | negative |  | validated |
| 891 | hsa-mir-210 | LRCH2 | -0.260 | 2.5E-13 | tarbase | MIMAT0000267 | 57631 | ENSG00000130224 | Degradome sequencing | positive |  | validated |
| 892 | hsa-mir-210 | LRRC8A | -0.286 | 6.6E-16 | tarbase | MIMAT0000267 | 56262 | ENSG00000136802 | Degradome sequencing | positive |  | validated |
| 893 | hsa-mir-210 | LY6D | -0.274 | 1.0E-14 | tarbase | MIMAT0000267 | 8581 | ENSG00000167656 | Degradome sequencing | negative |  | validated |
| 894 | hsa-mir-210 | MAFF | -0.290 | 2.8E-16 | tarbase | MIMAT0000267 | 23764 | ENSG00000185022 | Degradome sequencing | negative |  | validated |
| 895 | hsa-mir-210 | MALL | -0.425 | 0 | tarbase | MIMAT0000267 | 7851 | ENSG00000144063 | Degradome sequencing | negative |  | validated |
| 896 | hsa-mir-210 | MAOA | -0.324 | 1.8E-20 | tarbase | MIMAT0000267 | 4128 | ENSG00000189221 | Degradome sequencing | negative |  | validated |
| 897 | hsa-mir-210 | MAP3K8 | -0.301 | 1.1E-17 | tarbase | MIMAT0000267 | 1326 | ENSG00000107968 | Degradome sequencing | positive |  | validated |
| 898 | hsa-mir-210 | MCTP1 | -0.402 | 0 | tarbase | MIMAT0000267 | 79772 | ENSG00000175471 | Degradome sequencing | positive |  | validated |
| 899 | hsa-mir-210 | MEF2D | -0.326 | 6.1E-21 | mirtarbase | MIMAT0000267 | 4209 | ENSG00000116604 | HITS-CLIP | Functional MTI (Weak) | 22473208 | validated |
| 900 | hsa-mir-210 | MEF2D | -0.326 | 6.1E-21 | tarbase | MIMAT0000267 | 4209 | ENSG00000116604 | Degradome sequencing//Degradome sequencing | positive |  | validated |
| 901 | hsa-mir-210 | MET | -0.309 | 2.1E-18 | tarbase | MIMAT0000267 | 4233 | ENSG00000105976 | Degradome sequencing | positive |  | validated |
| 902 | hsa-mir-210 | MGAT3 | -0.405 | 0 | tarbase | MIMAT0000267 | 4248 | ENSG00000128268 | Degradome sequencing | positive |  | validated |
| 903 | hsa-mir-210 | MICAL1 | -0.296 | 5.2E-17 | tarbase | MIMAT0000267 | 64780 | ENSG00000135596 | Degradome sequencing | positive |  | validated |
| 904 | hsa-mir-210 | MIGA2 | -0.309 | 1.8E-18 | tarbase | MIMAT0000267 | 84895 | ENSG00000148343 | Degradome sequencing//Degradome sequencing | positive |  | validated |
| 905 | hsa-mir-210 | MMP28 | -0.368 | 3.9E-26 | tarbase | MIMAT0000267 | 79148 | ENSG00000271447 | Degradome sequencing | positive |  | validated |
| 906 | hsa-mir-210 | MMRN1 | -0.556 | 0 | tarbase | MIMAT0000267 | 22915 | ENSG00000138722 | Degradome sequencing | positive |  | validated |
| 907 | hsa-mir-210 | MYBPC1 | -0.297 | 3.9E-17 | tarbase | MIMAT0000267 | 4604 | ENSG00000196091 | Degradome sequencing | negative |  | validated |
| 908 | hsa-mir-210 | NEK3 | -0.265 | 9.7E-14 | tarbase | MIMAT0000267 | 4752 | ENSG00000136098 | Degradome sequencing | negative |  | validated |
| 909 | hsa-mir-210 | NLGN1 | -0.327 | 1.1E-20 | tarbase | MIMAT0000267 | 22871 | ENSG00000169760 | Degradome sequencing | positive |  | validated |
| 910 | hsa-mir-210 | NLRX1 | -0.259 | 2.6E-13 | tarbase | MIMAT0000267 | 79671 | ENSG00000160703 | Degradome sequencing | positive |  | validated |
| 911 | hsa-mir-210 | NOS3 | -0.440 | 0 | tarbase | MIMAT0000267 | 4846 | ENSG00000164867 | Degradome sequencing | positive |  | validated |
| 912 | hsa-mir-210 | NOSTRIN | -0.341 | 0 | tarbase | MIMAT0000267 | 115677 | ENSG00000163072 | Degradome sequencing | positive |  | validated |
| 913 | hsa-mir-210 | NPAS2 | -0.253 | 1.2E-12 | tarbase | MIMAT0000267 | 4862 | ENSG00000170485 | Degradome sequencing | positive |  | validated |
| 914 | hsa-mir-210 | NPHP3 | -0.291 | 1.9E-16 | tarbase | MIMAT0000267 | 27031 | ENSG00000113971 | Degradome sequencing | positive |  | validated |
| 915 | hsa-mir-210 | NPR2 | -0.269 | 3.6E-14 | tarbase | MIMAT0000267 | 4882 | ENSG00000159899 | Degradome sequencing | positive |  | validated |
| 916 | hsa-mir-210 | NR3C2 | -0.339 | 0 | tarbase | MIMAT0000267 | 4306 | ENSG00000151623 | Degradome sequencing | positive |  | validated |
| 917 | hsa-mir-210 | PALD1 | -0.467 | 0 | tarbase | MIMAT0000267 | 27143 | ENSG00000107719 | Degradome sequencing | negative |  | validated |
| 918 | hsa-mir-210 | PCOLCE2 | -0.338 | 3.8E-22 | tarbase | MIMAT0000267 | 26577 | ENSG00000163710 | Degradome sequencing | negative |  | validated |
| 919 | hsa-mir-210 | PCSK5 | -0.372 | 0 | tarbase | MIMAT0000267 | 5125 | ENSG00000099139 | Degradome sequencing | positive |  | validated |
| 920 | hsa-mir-210 | PGAP4 | -0.398 | 1.1E-30 | tarbase | MIMAT0000267 | 84302 | ENSG00000165152 | Degradome sequencing | positive |  | validated |
| 921 | hsa-mir-210 | PHYKPL | -0.440 | 0 | tarbase | MIMAT0000267 | 85007 | ENSG00000175309 | Degradome sequencing | positive |  | validated |
| 922 | hsa-mir-210 | PJVK | -0.279 | 2.9E-15 | tarbase | MIMAT0000267 | 494513 | ENSG00000204311 | Degradome sequencing//Degradome sequencing | negative |  | validated |
| 923 | hsa-mir-210 | PKDCC | -0.397 | 0 | tarbase | MIMAT0000267 | 91461 | ENSG00000162878 | Degradome sequencing | positive |  | validated |
| 924 | hsa-mir-210 | PLA2G2A | -0.334 | 1.2E-21 | tarbase | MIMAT0000267 | 5320 | ENSG00000188257 | Degradome sequencing | positive |  | validated |
| 925 | hsa-mir-210 | PLA2G4A | -0.340 | 0 | tarbase | MIMAT0000267 | 5321 | ENSG00000116711 | Degradome sequencing | positive |  | validated |
| 926 | hsa-mir-210 | PLEKHG1 | -0.276 | 7.2E-15 | tarbase | MIMAT0000267 | 57480 | ENSG00000120278 | Degradome sequencing | negative |  | validated |
| 927 | hsa-mir-210 | PLEKHM2 | -0.291 | 1.9E-16 | tarbase | MIMAT0000267 | 23207 | ENSG00000116786 | Degradome sequencing//Degradome sequencing//Degradome sequencing//Degradome sequencing | positive |  | validated |
| 928 | hsa-mir-210 | PLLP | -0.286 | 6.4E-16 | tarbase | MIMAT0000267 | 51090 | ENSG00000102934 | Degradome sequencing | positive |  | validated |
| 929 | hsa-mir-210 | PLPP3 | -0.356 | 0 | tarbase | MIMAT0000267 | 8613 | ENSG00000162407 | Degradome sequencing//Degradome sequencing//Degradome sequencing | positive |  | validated |
| 930 | hsa-mir-210 | PLSCR4 | -0.336 | 0 | tarbase | MIMAT0000267 | 57088 | ENSG00000114698 | Degradome sequencing | positive |  | validated |
| 931 | hsa-mir-210 | POLR3GL | -0.282 | 1.7E-15 | tarbase | MIMAT0000267 | 84265 | ENSG00000121851 | Degradome sequencing | positive |  | validated |
| 932 | hsa-mir-210 | POU2AF1 | -0.302 | 9.3E-18 | mirtarbase | MIMAT0000267 | 5450 | ENSG00000110777 | HITS-CLIP | Functional MTI (Weak) | 22473208 | validated |
| 933 | hsa-mir-210 | POU2AF1 | -0.302 | 9.3E-18 | tarbase | MIMAT0000267 | 5450 | ENSG00000110777 | Degradome sequencing | positive |  | validated |
| 934 | hsa-mir-210 | PPARG | -0.383 | 2.5E-28 | tarbase | MIMAT0000267 | 5468 | ENSG00000132170 | Degradome sequencing | negative |  | validated |
| 935 | hsa-mir-210 | PPM1F | -0.490 | 0 | tarbase | MIMAT0000267 | 9647 | ENSG00000100034 | Degradome sequencing | positive |  | validated |
| 936 | hsa-mir-210 | PROCR | -0.290 | 2.6E-16 | tarbase | MIMAT0000267 | 10544 | ENSG00000101000 | Degradome sequencing | positive |  | validated |
| 937 | hsa-mir-210 | PROS1 | -0.438 | 0 | tarbase | MIMAT0000267 | 5627 | ENSG00000184500 | Degradome sequencing//Degradome sequencing | positive |  | validated |
| 938 | hsa-mir-210 | PRSS35 | -0.291 | 1.5E-16 | tarbase | MIMAT0000267 | 167681 | ENSG00000146250 | Degradome sequencing | positive |  | validated |
| 939 | hsa-mir-210 | PTGS2 | -0.375 | 3.1E-27 | tarbase | MIMAT0000267 | 5743 | ENSG00000073756 | Degradome sequencing | positive |  | validated |
| 940 | hsa-mir-210 | PTGS2 | -0.375 | 3.1E-27 | tarbase | MIMAT0000267 | 5743 | ENSG00000073756 | Degradome sequencing | negative |  | validated |
| 941 | hsa-mir-210 | QTRT1 | -0.274 | 1.1E-14 | tarbase | MIMAT0000267 | 81890 | ENSG00000213339 | Degradome sequencing | negative |  | validated |
| 942 | hsa-mir-210 | RAB11B | -0.362 | 0 | tarbase | MIMAT0000267 | 9230 | ENSG00000185236 | Degradome sequencing | positive |  | validated |
| 943 | hsa-mir-210 | RAB11B | -0.362 | 0 | tarbase | MIMAT0000267 | 9230 | ENSG00000185236 | Degradome sequencing | negative |  | validated |
| 944 | hsa-mir-210 | RAC2 | -0.288 | 4.0E-16 | tarbase | MIMAT0000267 | 5880 | ENSG00000128340 | Degradome sequencing | negative |  | validated |
| 945 | hsa-mir-210 | RAMP2 | -0.500 | 0 | tarbase | MIMAT0000267 | 10266 | ENSG00000131477 | Degradome sequencing | negative |  | validated |
| 946 | hsa-mir-210 | RAPGEF1 | -0.255 | 6.8E-13 | tarbase | MIMAT0000267 | 2889 | ENSG00000107263 | Degradome sequencing | positive |  | validated |
| 947 | hsa-mir-210 | RAPGEF1 | -0.255 | 6.8E-13 | tarbase | MIMAT0000267 | 2889 | ENSG00000107263 | Degradome sequencing | negative |  | validated |
| 948 | hsa-mir-210 | RCAN2 | -0.330 | 3.7E-22 | tarbase | MIMAT0000267 | 10231 | ENSG00000172348 | Degradome sequencing | positive |  | validated |
| 949 | hsa-mir-210 | RELB | -0.293 | 1.3E-16 | tarbase | MIMAT0000267 | 5971 | ENSG00000104856 | Degradome sequencing | negative |  | validated |
| 950 | hsa-mir-210 | RGN | -0.347 | 2.5E-23 | tarbase | MIMAT0000267 | 9104 | ENSG00000130988 | Degradome sequencing | positive |  | validated |
| 951 | hsa-mir-210 | RGS2 | -0.296 | 5.8E-17 | tarbase | MIMAT0000267 | 5997 | ENSG00000116741 | Degradome sequencing | positive |  | validated |
| 952 | hsa-mir-210 | RGS2 | -0.296 | 5.8E-17 | tarbase | MIMAT0000267 | 5997 | ENSG00000116741 | Degradome sequencing | negative |  | validated |
| 953 | hsa-mir-210 | RGS5 | -0.362 | 0 | tarbase | MIMAT0000267 | 8490 | ENSG00000143248 | Degradome sequencing | positive |  | validated |
| 954 | hsa-mir-210 | RGS5 | -0.362 | 0 | tarbase | MIMAT0000267 | 8490 | ENSG00000143248 | Degradome sequencing | negative |  | validated |
| 955 | hsa-mir-210 | RHOB | -0.294 | 1.1E-16 | tarbase | MIMAT0000267 | 388 | ENSG00000143878 | Degradome sequencing//Degradome sequencing//Degradome sequencing | positive |  | validated |
| 956 | hsa-mir-210 | RHOU | -0.343 | 0 | tarbase | MIMAT0000267 | 58480 | ENSG00000116574 | Degradome sequencing | positive |  | validated |
| 957 | hsa-mir-210 | RND3 | -0.266 | 7.0E-14 | tarbase | MIMAT0000267 | 390 | ENSG00000115963 | Degradome sequencing | positive |  | validated |
| 958 | hsa-mir-210 | RNF166 | -0.348 | 2.2E-23 | tarbase | MIMAT0000267 | 115992 | ENSG00000158717 | Degradome sequencing | negative |  | validated |
| 959 | hsa-mir-210 | RPL31 | -0.269 | 3.5E-14 | tarbase | MIMAT0000267 | 6160 | ENSG00000071082 | Degradome sequencing | positive |  | validated |
| 960 | hsa-mir-210 | RUNX1T1 | -0.265 | 6.5E-14 | mirtarbase | MIMAT0000267 | 862 | ENSG00000079102 | PAR-CLIP//HITS-CLIP | Functional MTI (Weak) | 21572407 | validated |
| 961 | hsa-mir-210 | RUNX1T1 | -0.265 | 6.5E-14 | mirtarbase | MIMAT0000267 | 862 | ENSG00000079102 | HITS-CLIP | Functional MTI (Weak) | 23313552 | validated |
| 962 | hsa-mir-210 | RUNX1T1 | -0.265 | 6.5E-14 | mirtarbase | MIMAT0000267 | 862 | ENSG00000079102 | HITS-CLIP | Functional MTI (Weak) | 27418678 | validated |
| 963 | hsa-mir-210 | SAMD5 | -0.438 | 0 | tarbase | MIMAT0000267 | 389432 | ENSG00000203727 | Degradome sequencing | positive |  | validated |
| 964 | hsa-mir-210 | SCN1B | -0.336 | 0 | mirtarbase | MIMAT0000267 | 6324 | ENSG00000105711 | CLASH | Functional MTI (Weak) | 23622248 | validated |
| 965 | hsa-mir-210 | SCN2A | -0.265 | 8.0E-14 | tarbase | MIMAT0000267 | 6326 | ENSG00000136531 | Degradome sequencing | positive |  | validated |
| 966 | hsa-mir-210 | SELENOP | -0.291 | 2.0E-16 | tarbase | MIMAT0000267 | 6414 | ENSG00000250722 | Degradome sequencing | positive |  | validated |
| 967 | hsa-mir-210 | SGK2 | -0.288 | 3.3E-16 | tarbase | MIMAT0000267 | 10110 | ENSG00000101049 | Degradome sequencing | positive |  | validated |
| 968 | hsa-mir-210 | SH3RF2 | -0.280 | 2.8E-15 | tarbase | MIMAT0000267 | 153769 | ENSG00000156463 | Degradome sequencing | positive |  | validated |
| 969 | hsa-mir-210 | SIGLEC15 | -0.257 | 4.0E-13 | tarbase | MIMAT0000267 | 284266 | ENSG00000197046 | Degradome sequencing | positive |  | validated |
| 970 | hsa-mir-210 | SLC25A12 | -0.327 | 3.8E-21 | tarbase | MIMAT0000267 | 8604 | ENSG00000115840 | Degradome sequencing | negative |  | validated |
| 971 | hsa-mir-210 | SLC25A45 | -0.268 | 4.5E-14 | tarbase | MIMAT0000267 | 283130 | ENSG00000162241 | Degradome sequencing | positive |  | validated |
| 972 | hsa-mir-210 | SLCO2A1 | -0.482 | 0 | tarbase | MIMAT0000267 | 6578 | ENSG00000174640 | Degradome sequencing | negative |  | validated |
| 973 | hsa-mir-210 | SLCO3A1 | -0.298 | 3.8E-17 | mirtarbase | MIMAT0000267 | 28232 | ENSG00000176463 | HITS-CLIP | Functional MTI (Weak) | 23313552 | validated |
| 974 | hsa-mir-210 | SOWAHA | -0.253 | 1.2E-12 | tarbase | MIMAT0000267 | 134548 | ENSG00000198944 | Degradome sequencing | negative |  | validated |
| 975 | hsa-mir-210 | SOX8 | -0.532 | 1.5E-57 | tarbase | MIMAT0000267 | 30812 | ENSG00000005513 | Degradome sequencing | negative |  | validated |
| 976 | hsa-mir-210 | SPINK5 | -0.292 | 1.1E-16 | tarbase | MIMAT0000267 | 11005 | ENSG00000133710 | Degradome sequencing//Degradome sequencing | positive |  | validated |
| 977 | hsa-mir-210 | ST6GALNAC1 | -0.407 | 4.3E-32 | tarbase | MIMAT0000267 | 55808 | ENSG00000070526 | Degradome sequencing//Degradome sequencing | positive |  | validated |
| 978 | hsa-mir-210 | ST6GALNAC3 | -0.412 | 0 | tarbase | MIMAT0000267 | 256435 | ENSG00000184005 | Degradome sequencing | positive |  | validated |
| 979 | hsa-mir-210 | STAT4 | -0.264 | 9.1E-14 | tarbase | MIMAT0000267 | 6775 | ENSG00000138378 | Degradome sequencing | positive |  | validated |
| 980 | hsa-mir-210 | STAT6 | -0.356 | 0 | tarbase | MIMAT0000267 | 6778 | ENSG00000166888 | Degradome sequencing//Degradome sequencing | positive |  | validated |
| 981 | hsa-mir-210 | STK40 | -0.261 | 2.3E-13 | tarbase | MIMAT0000267 | 83931 | ENSG00000196182 | Degradome sequencing | negative |  | validated |
| 982 | hsa-mir-210 | TFPI | -0.374 | 0 | tarbase | MIMAT0000267 | 7035 | ENSG00000003436 | Degradome sequencing | positive |  | validated |
| 983 | hsa-mir-210 | THSD7A | -0.319 | 8.3E-20 | mirtarbase | MIMAT0000267 | 221981 | ENSG00000005108 | Luciferase reporter assay//qRT-PCR//Western blot | Functional MTI | 26796133 | validated |
| 984 | hsa-mir-210 | THSD7A | -0.319 | 8.3E-20 | tarbase | MIMAT0000267 | 221981 | ENSG00000005108 | Degradome sequencing | positive |  | validated |
| 985 | hsa-mir-210 | TINAGL1 | -0.422 | 0 | tarbase | MIMAT0000267 | 64129 | ENSG00000142910 | Degradome sequencing | negative |  | validated |
| 986 | hsa-mir-210 | TLE4 | -0.280 | 3.0E-15 | tarbase | MIMAT0000267 | 7091 | ENSG00000106829 | Degradome sequencing | negative |  | validated |
| 987 | hsa-mir-210 | TLE5 | -0.259 | 3.4E-13 | tarbase | MIMAT0000267 | 166 | ENSG00000104964 | Degradome sequencing | positive |  | validated |
| 988 | hsa-mir-210 | TM4SF18 | -0.296 | 6.6E-17 | tarbase | MIMAT0000267 | 116441 | ENSG00000163762 | Degradome sequencing//Degradome sequencing | positive |  | validated |
| 989 | hsa-mir-210 | TMEM129 | -0.258 | 3.7E-13 | tarbase | MIMAT0000267 | 92305 | ENSG00000168936 | Degradome sequencing | positive |  | validated |
| 990 | hsa-mir-210 | TNFAIP3 | -0.278 | 4.7E-15 | tarbase | MIMAT0000267 | 7128 | ENSG00000118503 | Degradome sequencing | negative |  | validated |
| 991 | hsa-mir-210 | TNFRSF10D | -0.293 | 9.7E-17 | tarbase | MIMAT0000267 | 8793 | ENSG00000173530 | Degradome sequencing | negative |  | validated |
| 992 | hsa-mir-210 | TNIP2 | -0.290 | 2.5E-16 | tarbase | MIMAT0000267 | 79155 | ENSG00000168884 | Degradome sequencing//Degradome sequencing | positive |  | validated |
| 993 | hsa-mir-210 | TNNC1 | -0.258 | 3.0E-13 | tarbase | MIMAT0000267 | 7134 | ENSG00000114854 | Degradome sequencing//Degradome sequencing | positive |  | validated |
| 994 | hsa-mir-210 | TOX2 | -0.323 | 2.9E-20 | tarbase | MIMAT0000267 | 84969 | ENSG00000124191 | Degradome sequencing | positive |  | validated |
| 995 | hsa-mir-210 | TRIOBP | -0.380 | 0 | tarbase | MIMAT0000267 | 11078 | ENSG00000100106 | Degradome sequencing | positive |  | validated |
| 996 | hsa-mir-210 | TSC22D3 | -0.271 | 2.4E-14 | tarbase | MIMAT0000267 | 1831 | ENSG00000157514 | Degradome sequencing//Degradome sequencing | positive |  | validated |
| 997 | hsa-mir-210 | TTYH2 | -0.306 | 4.2E-18 | tarbase | MIMAT0000267 | 94015 | ENSG00000141540 | Degradome sequencing | negative |  | validated |
| 998 | hsa-mir-210 | TXNIP | -0.438 | 0 | tarbase | MIMAT0000267 | 10628 | ENSG00000265972 | Degradome sequencing | positive |  | validated |
| 999 | hsa-mir-210 | UBXN6 | -0.295 | 8.0E-17 | tarbase | MIMAT0000267 | 80700 | ENSG00000167671 | Degradome sequencing | positive |  | validated |
| 1000 | hsa-mir-210 | VILL | -0.486 | 0 | tarbase | MIMAT0000267 | 50853 | ENSG00000136059 | Degradome sequencing | positive |  | validated |
| 1001 | hsa-mir-210 | WNT11 | -0.341 | 0 | tarbase | MIMAT0000267 | 7481 | ENSG00000085741 | Degradome sequencing | negative |  | validated |
| 1002 | hsa-mir-210 | XAB2 | -0.276 | 7.3E-15 | tarbase | MIMAT0000267 | 56949 | ENSG00000076924 | Degradome sequencing | negative |  | validated |
| 1003 | hsa-mir-210 | ZCCHC24 | -0.471 | 0 | tarbase | MIMAT0000267 | 219654 | ENSG00000165424 | Degradome sequencing | negative |  | validated |
| 1004 | hsa-mir-210 | ZFP36 | -0.436 | 0 | tarbase | MIMAT0000267 | 7538 | ENSG00000128016 | Degradome sequencing | positive |  | validated |
| 1005 | hsa-mir-210 | ZFP36L2 | -0.386 | 0 | tarbase | MIMAT0000267 | 678 | ENSG00000152518 | Degradome sequencing | positive |  | validated |
| 1006 | hsa-mir-210 | ZNF266 | -0.281 | 2.1E-15 | tarbase | MIMAT0000267 | 10781 | ENSG00000174652 | Degradome sequencing | positive |  | validated |
| 1007 | hsa-mir-210 | ZNF362 | -0.305 | 5.0E-18 | tarbase | MIMAT0000267 | 149076 | ENSG00000160094 | Degradome sequencing | positive |  | validated |
| 1008 | hsa-mir-221 | ANKS1B | -0.252 | 1.4E-12 | tarbase | MIMAT0000278 | 56899 | ENSG00000185046 | Degradome sequencing | positive |  | validated |
| 1009 | hsa-mir-221 | ATP7A | -0.275 | 8.9E-15 | tarbase | MIMAT0000278 | 538 | ENSG00000165240 | Degradome sequencing | positive |  | validated |
| 1010 | hsa-mir-221 | CUL3 | -0.256 | 6.3E-13 | tarbase | MIMAT0000278 | 8452 | ENSG00000036257 | Degradome sequencing | positive |  | validated |
| 1011 | hsa-mir-221 | ERBB3 | -0.306 | 4.6E-18 | tarbase | MIMAT0000278 | 2065 | ENSG00000065361 | Degradome sequencing | positive |  | validated |
| 1012 | hsa-mir-221 | KLHDC10 | -0.258 | 4.5E-13 | mirtarbase | MIMAT0000278 | 23008 | ENSG00000128607 | PAR-CLIP | Functional MTI (Weak) | 26701625 | validated |
| 1013 | hsa-mir-221 | NAPEPLD | -0.251 | 1.8E-12 | tarbase | MIMAT0000278 | 222236 | ENSG00000161048 | Degradome sequencing | positive |  | validated |
| 1014 | hsa-mir-221 | PDIK1L | -0.268 | 4.5E-14 | mirtarbase | MIMAT0000278 | 149420 | ENSG00000175087 | Sequencing | Functional MTI (Weak) | 20371350 | validated |
| 1015 | hsa-mir-221 | PDIK1L | -0.268 | 4.5E-14 | mirtarbase | MIMAT0000278 | 149420 | ENSG00000175087 | PAR-CLIP | Functional MTI (Weak) | 21572407 | validated |
| 1016 | hsa-mir-221 | SHLD2 | -0.252 | 1.5E-12 | mirtarbase | MIMAT0000278 | 54537 | ENSG00000122376 | PAR-CLIP | Functional MTI (Weak) | 20371350 | validated |
| 1017 | hsa-mir-221 | SHLD2 | -0.252 | 1.5E-12 | mirtarbase | MIMAT0000278 | 54537 | ENSG00000122376 | PAR-CLIP | Functional MTI (Weak) | 26701625 | validated |
| 1018 | hsa-mir-221 | SHLD2 | -0.252 | 1.5E-12 | tarbase | MIMAT0000278 | 54537 | ENSG00000122376 | Degradome sequencing//Degradome sequencing//Degradome sequencing | positive |  | validated |
| 1019 | hsa-mir-221 | TSPAN13 | -0.259 | 3.6E-13 | mirtarbase | MIMAT0000278 | 27075 | ENSG00000106537 | PAR-CLIP | Functional MTI (Weak) | 21572407 | validated |
| 1020 | hsa-mir-221 | TSPAN13 | -0.259 | 3.6E-13 | mirtarbase | MIMAT0000278 | 27075 | ENSG00000106537 | PAR-CLIP | Functional MTI (Weak) | 20371350 | validated |
| 1021 | hsa-mir-221 | TSPAN13 | -0.259 | 3.6E-13 | tarbase | MIMAT0000278 | 27075 | ENSG00000106537 | Degradome sequencing//Degradome sequencing//Degradome sequencing | positive |  | validated |
| 1022 | hsa-mir-221 | USP37 | -0.252 | 1.4E-12 | tarbase | MIMAT0000278 | 57695 | ENSG00000135913 | Degradome sequencing | positive |  | validated |
| 1023 | hsa-mir-221 | ZFP30 | -0.264 | 1.1E-13 | mirtarbase | MIMAT0000278 | 22835 | ENSG00000120784 | PAR-CLIP | Functional MTI (Weak) | 21572407 | validated |
| 1024 | hsa-mir-221 | ZFP30 | -0.264 | 1.1E-13 | mirtarbase | MIMAT0000278 | 22835 | ENSG00000120784 | PAR-CLIP | Functional MTI (Weak) | 20371350 | validated |
| 1025 | hsa-mir-221 | ZNF91 | -0.259 | 3.4E-13 | tarbase | MIMAT0000278 | 7644 | ENSG00000167232 | Degradome sequencing//Degradome sequencing | positive |  | validated |
| 1026 | hsa-mir-328 | ALDH1L1 | -0.298 | 3.0E-17 | tarbase | MIMAT0000752 | 10840 | ENSG00000144908 | Degradome sequencing | positive |  | validated |
| 1027 | hsa-mir-328 | BTBD2 | -0.307 | 3.4E-18 | tarbase | MIMAT0000752 | 55643 | ENSG00000133243 | Degradome sequencing | positive |  | validated |
| 1028 | hsa-mir-328 | CDC42EP1 | -0.313 | 5.0E-19 | mirtarbase | MIMAT0000752 | 11135 | ENSG00000128283 | CLASH | Functional MTI (Weak) | 23622248 | validated |
| 1029 | hsa-mir-328 | DIPK1B | -0.257 | 4.5E-13 | tarbase | MIMAT0000752 | 138311 | ENSG00000165716 | Degradome sequencing | positive |  | validated |
| 1030 | hsa-mir-328 | DMPK | -0.268 | 4.1E-14 | tarbase | MIMAT0000752 | 1760 | ENSG00000104936 | Degradome sequencing//Degradome sequencing | positive |  | validated |
| 1031 | hsa-mir-328 | DMWD | -0.322 | 4.2E-20 | tarbase | MIMAT0000752 | 1762 | ENSG00000185800 | Degradome sequencing | positive |  | validated |
| 1032 | hsa-mir-328 | ERF | -0.351 | 0 | tarbase | MIMAT0000752 | 2077 | ENSG00000105722 | Degradome sequencing//Degradome sequencing//Degradome sequencing | positive |  | validated |
| 1033 | hsa-mir-328 | FAM193B | -0.288 | 4.6E-16 | mirtarbase | MIMAT0000752 | 54540 | ENSG00000146067 | PAR-CLIP | Functional MTI (Weak) | 27292025 | validated |
| 1034 | hsa-mir-328 | FKBP8 | -0.266 | 7.3E-14 | tarbase | MIMAT0000752 | 23770 | ENSG00000105701 | Degradome sequencing//Degradome sequencing//Degradome sequencing | positive |  | validated |
| 1035 | hsa-mir-328 | FUS | -0.300 | 2.2E-17 | tarbase | MIMAT0000752 | 2521 | ENSG00000089280 | Degradome sequencing | positive |  | validated |
| 1036 | hsa-mir-328 | GIGYF1 | -0.307 | 3.5E-18 | tarbase | MIMAT0000752 | 64599 | ENSG00000146830 | Degradome sequencing | positive |  | validated |
| 1037 | hsa-mir-328 | GPSM1 | -0.259 | 3.0E-13 | tarbase | MIMAT0000752 | 26086 | ENSG00000160360 | Degradome sequencing | positive |  | validated |
| 1038 | hsa-mir-328 | MBD3 | -0.306 | 3.9E-18 | tarbase | MIMAT0000752 | 53615 | ENSG00000071655 | Degradome sequencing | positive |  | validated |
| 1039 | hsa-mir-328 | NDUFA4L2 | -0.318 | 1.2E-19 | tarbase | MIMAT0000752 | 56901 | ENSG00000185633 | Degradome sequencing | positive |  | validated |
| 1040 | hsa-mir-328 | NDUFV1 | -0.279 | 3.4E-15 | tarbase | MIMAT0000752 | 4723 | ENSG00000167792 | Degradome sequencing | positive |  | validated |
| 1041 | hsa-mir-328 | NELFB | -0.289 | 3.1E-16 | tarbase | MIMAT0000752 | 25920 | ENSG00000188986 | Degradome sequencing | positive |  | validated |
| 1042 | hsa-mir-328 | P2RY11 | -0.289 | 2.9E-16 | tarbase | MIMAT0000752 | 5032 | ENSG00000244165 | Degradome sequencing//Degradome sequencing | positive |  | validated |
| 1043 | hsa-mir-328 | PIM3 | -0.254 | 9.6E-13 | tarbase | MIMAT0000752 | 415116 | ENSG00000198355 | Degradome sequencing | positive |  | validated |
| 1044 | hsa-mir-328 | RHOB | -0.309 | 1.9E-18 | tarbase | MIMAT0000752 | 388 | ENSG00000143878 | Degradome sequencing | positive |  | validated |
| 1045 | hsa-mir-328 | RPL10 | -0.271 | 2.4E-14 | tarbase | MIMAT0000752 | 6134 | ENSG00000147403 | Degradome sequencing | positive |  | validated |
| 1046 | hsa-mir-328 | RPS14 | -0.298 | 3.6E-17 | tarbase | MIMAT0000752 | 6208 | ENSG00000164587 | Degradome sequencing | positive |  | validated |
| 1047 | hsa-mir-328 | RPS9 | -0.392 | 0 | mirtarbase | MIMAT0000752 | 6203 | ENSG00000278270 | CLASH | Functional MTI (Weak) | 23622248 | validated |
| 1048 | hsa-mir-328 | RPS9 | -0.392 | 0 | mirtarbase | MIMAT0000752 | 6203 | ENSG00000170889 | CLASH | Functional MTI (Weak) | 23622248 | validated |
| 1049 | hsa-mir-328 | RPS9 | -0.392 | 0 | mirtarbase | MIMAT0000752 | 6203 | ENSG00000274646 | CLASH | Functional MTI (Weak) | 23622248 | validated |
| 1050 | hsa-mir-328 | RPS9 | -0.392 | 0 | mirtarbase | MIMAT0000752 | 6203 | ENSG00000274950 | CLASH | Functional MTI (Weak) | 23622248 | validated |
| 1051 | hsa-mir-328 | RPS9 | -0.392 | 0 | mirtarbase | MIMAT0000752 | 6203 | ENSG00000277359 | CLASH | Functional MTI (Weak) | 23622248 | validated |
| 1052 | hsa-mir-328 | RPS9 | -0.392 | 0 | mirtarbase | MIMAT0000752 | 6203 | ENSG00000275323 | CLASH | Functional MTI (Weak) | 23622248 | validated |
| 1053 | hsa-mir-328 | RPS9 | -0.392 | 0 | mirtarbase | MIMAT0000752 | 6203 | ENSG00000274005 | CLASH | Functional MTI (Weak) | 23622248 | validated |
| 1054 | hsa-mir-328 | RPS9 | -0.392 | 0 | mirtarbase | MIMAT0000752 | 6203 | ENSG00000274626 | CLASH | Functional MTI (Weak) | 23622248 | validated |
| 1055 | hsa-mir-328 | RPS9 | -0.392 | 0 | mirtarbase | MIMAT0000752 | 6203 | ENSG00000277079 | CLASH | Functional MTI (Weak) | 23622248 | validated |
| 1056 | hsa-mir-328 | RPS9 | -0.392 | 0 | mirtarbase | MIMAT0000752 | 6203 | ENSG00000278081 | CLASH | Functional MTI (Weak) | 23622248 | validated |
| 1057 | hsa-mir-328 | SBNO2 | -0.278 | 3.4E-15 | tarbase | MIMAT0000752 | 22904 | ENSG00000064932 | Degradome sequencing | positive |  | validated |
| 1058 | hsa-mir-328 | SFRP1 | -0.298 | 4.0E-17 | mirtarbase | MIMAT0000752 | 6422 | ENSG00000104332 | Immunohistochemistry//Luciferase reporter assay//Western blot | Functional MTI | 24305703 | validated |
| 1059 | hsa-mir-328 | SFRP1 | -0.298 | 4.0E-17 | tarbase | MIMAT0000752 | 6422 | ENSG00000104332 | Degradome sequencing | positive |  | validated |
| 1060 | hsa-mir-328 | SNAPC4 | -0.261 | 1.9E-13 | tarbase | MIMAT0000752 | 6621 | ENSG00000165684 | Degradome sequencing | positive |  | validated |
| 1061 | hsa-mir-328 | SRF | -0.280 | 2.8E-15 | tarbase | MIMAT0000752 | 6722 | ENSG00000112658 | Degradome sequencing | positive |  | validated |
| 1062 | hsa-mir-328 | SURF6 | -0.266 | 7.8E-14 | tarbase | MIMAT0000752 | 6838 | ENSG00000148296 | Degradome sequencing | positive |  | validated |
| 1063 | hsa-mir-328 | SYN2 | -0.325 | 2.2E-20 | tarbase | MIMAT0000752 | 6854 | ENSG00000157152 | Degradome sequencing | positive |  | validated |
| 1064 | hsa-mir-328 | TCEAL2 | -0.287 | 4.2E-16 | tarbase | MIMAT0000752 | 140597 | ENSG00000184905 | Degradome sequencing | positive |  | validated |
| 1065 | hsa-mir-328 | TCEAL5 | -0.281 | 1.8E-15 | tarbase | MIMAT0000752 | 340543 | ENSG00000204065 | Degradome sequencing | positive |  | validated |
| 1066 | hsa-mir-328 | TRIM11 | -0.252 | 1.5E-12 | tarbase | MIMAT0000752 | 81559 | ENSG00000154370 | Degradome sequencing | positive |  | validated |
| 1067 | hsa-mir-328 | TRIOBP | -0.366 | 0 | tarbase | MIMAT0000752 | 11078 | ENSG00000100106 | Degradome sequencing | positive |  | validated |
| 1068 | hsa-mir-328 | TRIR | -0.283 | 1.4E-15 | tarbase | MIMAT0000752 | 79002 | ENSG00000123144 | Degradome sequencing | positive |  | validated |
| 1069 | hsa-mir-328 | ZBTB48 | -0.251 | 1.5E-12 | tarbase | MIMAT0000752 | 3104 | ENSG00000204859 | Degradome sequencing | positive |  | validated |
| 1070 | hsa-mir-628 | AMDHD2 | -0.384 | 0 | tarbase | MIMAT0004809 | 51005 | ENSG00000162066 | Degradome sequencing | positive |  | validated |
| 1071 | hsa-mir-628 | ANO9 | -0.307 | 3.5E-18 | tarbase | MIMAT0004809 | 338440 | ENSG00000185101 | Degradome sequencing | positive |  | validated |
| 1072 | hsa-mir-628 | C1orf35 | -0.401 | 0 | tarbase | MIMAT0004809 | 79169 | ENSG00000143793 | Degradome sequencing | positive |  | validated |
| 1073 | hsa-mir-628 | C7orf50 | -0.349 | 0 | tarbase | MIMAT0004809 | 84310 | ENSG00000146540 | Degradome sequencing | positive |  | validated |
| 1074 | hsa-mir-628 | CCDC137 | -0.273 | 1.5E-14 | tarbase | MIMAT0004809 | 339230 | ENSG00000185298 | Degradome sequencing | positive |  | validated |
| 1075 | hsa-mir-628 | CTDSP1 | -0.357 | 0 | tarbase | MIMAT0004809 | 58190 | ENSG00000144579 | Degradome sequencing | positive |  | validated |
| 1076 | hsa-mir-628 | DAZAP1 | -0.334 | 0 | tarbase | MIMAT0004809 | 26528 | ENSG00000071626 | Degradome sequencing | positive |  | validated |
| 1077 | hsa-mir-628 | DBNL | -0.300 | 2.2E-17 | tarbase | MIMAT0004809 | 28988 | ENSG00000136279 | Degradome sequencing | positive |  | validated |
| 1078 | hsa-mir-628 | DMKN | -0.297 | 3.3E-17 | tarbase | MIMAT0004809 | 93099 | ENSG00000161249 | Degradome sequencing | positive |  | validated |
| 1079 | hsa-mir-628 | INPP5E | -0.384 | 0 | tarbase | MIMAT0004809 | 56623 | ENSG00000148384 | Degradome sequencing | positive |  | validated |
| 1080 | hsa-mir-628 | MRPS2 | -0.273 | 1.6E-14 | tarbase | MIMAT0004809 | 51116 | ENSG00000122140 | Degradome sequencing | positive |  | validated |
| 1081 | hsa-mir-628 | MXD4 | -0.357 | 0 | tarbase | MIMAT0004809 | 10608 | ENSG00000123933 | Degradome sequencing | positive |  | validated |
| 1082 | hsa-mir-628 | NLGN2 | -0.284 | 1.0E-15 | tarbase | MIMAT0004809 | 57555 | ENSG00000169992 | Degradome sequencing | positive |  | validated |
| 1083 | hsa-mir-628 | NPDC1 | -0.291 | 2.0E-16 | tarbase | MIMAT0004809 | 56654 | ENSG00000107281 | Degradome sequencing | positive |  | validated |
| 1084 | hsa-mir-628 | OAZ1 | -0.283 | 1.5E-15 | tarbase | MIMAT0004809 | 4946 | ENSG00000104904 | Degradome sequencing//Degradome sequencing | positive |  | validated |
| 1085 | hsa-mir-628 | PTGES2 | -0.284 | 1.1E-15 | tarbase | MIMAT0004809 | 80142 | ENSG00000148334 | Degradome sequencing | positive |  | validated |
| 1086 | hsa-mir-628 | RPL31 | -0.273 | 1.4E-14 | tarbase | MIMAT0004809 | 6160 | ENSG00000071082 | Degradome sequencing | positive |  | validated |
| 1087 | hsa-mir-628 | SKIV2L | -0.281 | 2.6E-15 | tarbase | MIMAT0004809 | 6499 | ENSG00000204351 | Degradome sequencing | positive |  | validated |
| 1088 | hsa-mir-628 | SSBP4 | -0.400 | 0 | tarbase | MIMAT0004809 | 170463 | ENSG00000130511 | Degradome sequencing | positive |  | validated |
| 1089 | hsa-mir-628 | STK25 | -0.447 | 0 | mirtarbase | MIMAT0004809 | 10494 | ENSG00000115694 | PAR-CLIP | Functional MTI (Weak) | 27292025 | validated |
| 1090 | hsa-mir-628 | STRN4 | -0.304 | 7.3E-18 | tarbase | MIMAT0004809 | 29888 | ENSG00000090372 | Degradome sequencing | positive |  | validated |
| 1091 | hsa-mir-628 | TBC1D22A | -0.258 | 4.0E-13 | tarbase | MIMAT0004809 | 25771 | ENSG00000054611 | Degradome sequencing | positive |  | validated |
| 1092 | hsa-mir-628 | TESK1 | -0.348 | 0 | tarbase | MIMAT0004809 | 7016 | ENSG00000107140 | Degradome sequencing | positive |  | validated |
| 1093 | hsa-mir-628 | TRIM11 | -0.333 | 0 | tarbase | MIMAT0004809 | 81559 | ENSG00000154370 | Degradome sequencing//Degradome sequencing//Degradome sequencing | positive |  | validated |
| 1094 | hsa-mir-628 | TRIM41 | -0.303 | 1.0E-17 | mirtarbase | MIMAT0004809 | 90933 | ENSG00000146063 | PAR-CLIP | Functional MTI (Weak) | 26701625 | validated |
| 1095 | hsa-mir-628 | TUBGCP6 | -0.357 | 0 | tarbase | MIMAT0004809 | 85378 | ENSG00000128159 | Degradome sequencing | positive |  | validated |
| 1096 | hsa-mir-628 | WDR6 | -0.281 | 2.4E-15 | tarbase | MIMAT0004809 | 11180 | ENSG00000178252 | Degradome sequencing//Degradome sequencing | positive |  | validated |
| 1097 | hsa-mir-628 | WDR81 | -0.306 | 4.1E-18 | tarbase | MIMAT0004809 | 124997 | ENSG00000167716 | Degradome sequencing | positive |  | validated |
