# Supplementary material for: Potential biomarkers as a predictive factor of response to primary chemotherapy in breast cancer patients
Source: Braz J Med Biol Res. 2024 Oct 7;57:e13599. doi: 10.1590/1414-431X2024e13599 (PMC11463908; doi:10.1590/1414-431X2024e13599)
Supplement: Supplementary file 1 [file 1414-431X-bjmbr-57-e13599-suppl.zip › 13559_Supplementary Table S3.docx]

**Supplementary Table S3.** Signaling Pathways in Breast Cancer Estrogen Receptor-Negative Patients Using the ReactomePA R Package.

| **ID** | | **Description** | **GeneRatio** | | | | **BgRatio** | | **Pvalue** | | **P.adjust** | | **Qvalue** | | **geneID** | | **Count** | |
| --- | --- | --- | --- | --- | --- | --- | --- | --- | --- | --- | --- | --- | --- | --- | --- | --- | --- | --- |
| R-HSA-76002 | Platelet activation. signaling and aggregation | | | 33/505 | | 263/10867 | | 1.8E-07 | | 0.0002 | | 0.0002 | | ADRA2A/CFD/CFL1/COL1A1/CSK/DGKA/F13A1/FYN/GNAI2/GNAQ/GNG12/IGF1/IGF2/ITPR1/LCK/MMRN1/PCDH7/PHACTR2/PIK3R1/PLA2G4A/PROS1/PSAP/PTPN6/RAB27B/RAC2/RHOB/RHOG/SELENOP/SPARC/SRGN/THBS1/TLN1/VEGFC | | 33 | |  |
| R-HSA-9012999 | | RHO GTPase cycle | | | 41/505 | | 449/10867 | | 2.5E-05 | | 0.02 | | 0.01 | | AMIGO2/ARHGAP12/ARHGAP20/ARHGAP32/ARHGDIA/ARHGEF12/ARHGEF3/CAV1/CCP110/CKAP4/CLTC/CPD/CSK/CUL3/DAAM1/DST/ERBIN/FAM13B/FERMT2/FGD2/FMNL3/FRS2/GIT2/HSP90AA1/IQGAP1/IQGAP2/KTN1/LCK/NCKAP1/PCDH7/PIK3R1/RAC2/RHOB/RHOG/RNF20/TFRC/TMEM59/TMEM87A/TNFAIP1/VIM/WDR81 | | 41 | |
| R-HSA-2219528 | | PI3K/AKT Signaling in Cancer | | | 15/505 | | 104/10867 | | 8.6E-05 | | 0.03 | | 0.03 | | CDKN1A/ERBB3/ERBB4/FGF18/FOXO1/FOXO3/FRS2/FYN/GSK3B/KITLG/LCK/PIK3R1/RAC2/RHOG/RPS6KB2 | | 15 | |
| R-HSA-114608 | | Platelet degranulation | | | 17/505 | | 129/10867 | | 9.5E-05 | | 0.03 | | 0.03 | | CFD/CFL1/F13A1/IGF1/IGF2/MMRN1/PCDH7/PHACTR2/PROS1/PSAP/RAB27B/SELENOP/SPARC/SRGN/THBS1/TLN1/VEGFC | | 17 | |
| R-HSA-76005 | | Response to elevated platelet cytosolic Ca2+ | | | 17/505 | | 134/10867 | | 1.5E-04 | | 0.03 | | 0.03 | | CFD/CFL1/F13A1/IGF1/IGF2/MMRN1/PCDH7/PHACTR2/PROS1/PSAP/RAB27B/SELENOP/SPARC/SRGN/THBS1/TLN1/VEGFC | | 17 | |
| R-HSA-114604 | | GPVI-mediated activation cascade | | | 8/505 | | 35/10867 | | 1.6E-04 | | 0.03 | | 0.03 | | COL1A1/FYN/LCK/PIK3R1/PTPN6/RAC2/RHOB/RHOG | | 8 | |
| R-HSA-194138 | | Signaling by VEGF | | | 14/505 | | 108/10867 | | 4.6E-04 | | 0.08 | | 0.07 | | CAV1/CTNNA1/CTNND1/FLT1/FLT4/FYN/HSP90AA1/ITPR1/NCKAP1/NOS3/NRP1/PIK3R1/PXN/VEGFC | | 14 | |
| R-HSA-5663202 | | Diseases of signal transduction by growth factor receptors and second messengers | | | 36/505 | | 433/10867 | | 5.0E-04 | | 0.08 | | 0.07 | | ADAM10/AP3B1/APC/CDKN1A/CLTC/CSK/CSNK1A1/DUSP10/ERBB3/ERBB4/ERBIN/FGF18/FOXO1/FOXO3/FRS2/FYN/GCC2/GSK3B/GTF2F1/HSP90AA1/IQGAP1/KITLG/KREMEN1/LCK/MSN/PIK3R1/PSMC3/RAC2/RANBP2/RHOG/RPS6KB2/SEL1L/SQSTM1/TGFBR1/TGFBR2/TLN1 | | 36 | |
| R-HSA-9696270 | | RND2 GTPase cycle | | | 8/505 | | 43/10867 | | 7.1E-04 | | 0.09 | | 0.08 | | CAV1/CKAP4/DST/FRS2/KTN1/PIK3R1/TFRC/TNFAIP1 | | 8 | |
| R-HSA-9617629 | | Regulation of FOXO transcriptional activity by acetylation | | | 4/505 | | 10/10867 | | 7.7E-04 | | 0.09 | | 0.08 | | FOXO1/FOXO3/SIRT1/TXNIP | | 4 | |
| R-HSA-202131 | | Metabolism of nitric oxide: NOS3 activation and regulation | | | 5/505 | | 17/10867 | | 8.3E-04 | | 0.09 | | 0.08 | | CAV1/DDAH1/HSP90AA1/NOS3/NOSTRIN | | 5 | |
| R-HSA-451927 | | Interleukin-2 family signaling | | | 8/505 | | 44/10867 | | 8.3E-04 | | 0.09 | | 0.08 | | CSF2RA/IL15RA/IL21R/JAK1/LCK/PIK3R1/PTPN6/STAT4 | | 8 | |
| R-HSA-1655829 | | Regulation of cholesterol biosynthesis by SREBP (SREBF) | | | 9/505 | | 55/10867 | | 8.9E-04 | | 0.09 | | 0.08 | | ACACA/INSIG2/SCD/SEC23A/SEC24A/SEC24B/SEC24C/SEC24D/SP1 | | 9 | |
| R-HSA-9013026 | | RHOB GTPase cycle | | | 10/505 | | 70/10867 | | 1.4E-03 | | 0.12 | | 0.11 | | ARHGAP32/ARHGEF12/ARHGEF3/CAV1/DAAM1/ERBIN/PCDH7/PIK3R1/RHOB/TFRC | | 10 | |
| R-HSA-6785807 | | Interleukin-4 and Interleukin-13 signaling | | | 13/505 | | 108/10867 | | 1.5E-03 | | 0.12 | | 0.11 | | CDKN1A/CXCL8/F13A1/FOXO1/FOXO3/HSP90AA1/IL10/ITGB2/JAK1/PIK3R1/VCAM1/VIM/ZEB1 | | 13 | |
| R-HSA-449147 | | Signaling by Interleukins | | | 36/505 | | 462/10867 | | 1.6E-03 | | 0.12 | | 0.11 | | BRWD1/CDKN1A/CFL1/CSF2RA/CXCL8/DUSP4/F13A1/FOXO1/FOXO3/FYN/HCK/HSP90AA1/IL10/IL10RA/IL15RA/IL16/IL1R1/IL21R/IL6ST/IL7/ITGB2/JAK1/LCK/MAP3K3/MEF2C/MSN/PIK3R1/PSMC3/PTPN6/SQSTM1/STAT4/TAB1/VAMP2/VCAM1/VIM/ZEB1 | | 36 | |
| R-HSA-9013424 | | RHOV GTPase cycle | | | 7/505 | | 38/10867 | | 1.6E-03 | | 0.12 | | 0.11 | | ARHGAP12/CCP110/CLTC/DST/GIT2/IQGAP1/PIK3R1 | | 7 | |
| R-HSA-381426 | | Regulation of Insulin-like Growth Factor (IGF) transport and uptake by Insulin-like Growth Factor Binding Proteins (IGFBPs) | | | 14/505 | | 125/10867 | | 2.0E-03 | | 0.13 | | 0.12 | | ADAM10/BMP4/CKAP4/CST3/FAM20A/FSTL1/IGF1/IGF2/IGFBP5/IGFBP7/KTN1/MIA3/PRSS23/VCAN | | 14 | |
| R-HSA-177929 | | Signaling by EGFR | | | 8/505 | | 50/10867 | | 2.0E-03 | | 0.13 | | 0.12 | | ADAM10/ADAM12/CSK/LRIG1/PAG1/PIK3R1/PXN/SH3KBP1 | | 8 | |
| R-HSA-9013106 | | RHOC GTPase cycle | | | 10/505 | | 74/10867 | | 2.1E-03 | | 0.13 | | 0.12 | | ARHGAP32/ARHGDIA/ARHGEF12/CAV1/DAAM1/ERBIN/FMNL3/IQGAP1/PIK3R1/TFRC | | 10 | |
| R-HSA-203615 | | eNOS activation | | | 4/505 | | 13/10867 | | 2.4E-03 | | 0.14 | | 0.13 | | CAV1/DDAH1/HSP90AA1/NOS3 | | 4 | |
| R-HSA-9013148 | | CDC42 GTPase cycle | | | 16/505 | | 159/10867 | | 2.9E-03 | | 0.15 | | 0.14 | | ARHGAP20/ARHGAP32/ARHGDIA/ARHGEF12/CAV1/DAAM1/FAM13B/FGD2/FMNL3/GIT2/IQGAP1/IQGAP2/KTN1/PIK3R1/TFRC/WDR81 | | 16 | |
| R-HSA-9696264 | | RND3 GTPase cycle | | | 7/505 | | 42/10867 | | 2.9E-03 | | 0.15 | | 0.14 | | CAV1/CKAP4/CPD/DST/KTN1/PIK3R1/TNFAIP1 | | 7 | |
| R-HSA-9696273 | | RND1 GTPase cycle | | | 7/505 | | 42/10867 | | 2.9E-03 | | 0.15 | | 0.14 | | CAV1/CPD/DST/FRS2/PIK3R1/TFRC/TMEM59 | | 7 | |
| R-HSA-373755 | | Semaphorin interactions | | | 9/505 | | 65/10867 | | 3.0E-03 | | 0.15 | | 0.14 | | ARHGEF12/CFL1/FYN/GSK3B/HSP90AA1/NRP1/RHOB/SEMA5A/TLN1 | | 9 | |
| R-HSA-2219530 | | Constitutive Signaling by Aberrant PI3K in Cancer | | | 10/505 | | 78/10867 | | 3.2E-03 | | 0.15 | | 0.14 | | ERBB3/ERBB4/FGF18/FRS2/FYN/KITLG/LCK/PIK3R1/RAC2/RHOG | | 10 | |
| R-HSA-983695 | | Antigen activates B Cell Receptor (BCR) leading to generation of second messengers | | | 6/505 | | 32/10867 | | 3.2E-03 | | 0.15 | | 0.14 | | BTK/FYN/ITPR1/PIK3R1/PTPN6/SH3KBP1 | | 6 | |
| R-HSA-8980692 | | RHOA GTPase cycle | | | 15/505 | | 147/10867 | | 3.4E-03 | | 0.15 | | 0.14 | | ARHGAP20/ARHGAP32/ARHGDIA/ARHGEF12/ARHGEF3/CAV1/DAAM1/ERBIN/FMNL3/IQGAP1/KTN1/PCDH7/PIK3R1/TFRC/TMEM87A | | 15 | |
| R-HSA-5694530 | | Cargo concentration in the ER | | | 6/505 | | 33/10867 | | 3.7E-03 | | 0.16 | | 0.15 | | MIA3/SEC23A/SEC24A/SEC24B/SEC24C/SEC24D | | 6 | |
| R-HSA-163685 | | Integration of energy metabolism | | | 12/505 | | 108/10867 | | 4.3E-03 | | 0.17 | | 0.16 | | ACACA/ACSL3/ACSL4/ADIPOR1/ADRA2A/GNAI2/GNAQ/GNG12/IQGAP1/ITPR1/PRKAA2/VAMP2 | | 12 | |
| R-HSA-8957275 | | Post-translational protein phosphorylation | | | 12/505 | | 108/10867 | | 4.3E-03 | | 0.17 | | 0.16 | | ADAM10/BMP4/CKAP4/CST3/FAM20A/FSTL1/IGFBP5/IGFBP7/KTN1/MIA3/PRSS23/VCAN | | 12 | |
| R-HSA-210500 | | Glutamate Neurotransmitter Release Cycle | | | 5/505 | | 24/10867 | | 4.3E-03 | | 0.17 | | 0.16 | | ARL6IP5/RAB3A/SLC1A1/SLC38A2/VAMP2 | | 5 | |
| R-HSA-5674400 | | Constitutive Signaling by AKT1 E17K in Cancer | | | 5/505 | | 25/10867 | | 5.2E-03 | | 0.18 | | 0.17 | | CDKN1A/FOXO1/FOXO3/GSK3B/RPS6KB2 | | 5 | |
| R-HSA-9664565 | | Signaling by ERBB2 KD Mutants | | | 5/505 | | 25/10867 | | 5.2E-03 | | 0.18 | | 0.17 | | ERBB3/ERBB4/ERBIN/HSP90AA1/PIK3R1 | | 5 | |
| R-HSA-983170 | | Antigen Presentation: Folding. assembly and peptide loading of class I MHC | | | 5/505 | | 25/10867 | | 5.2E-03 | | 0.18 | | 0.17 | | SEC23A/SEC24A/SEC24B/SEC24C/SEC24D | | 5 | |
| R-HSA-1433559 | | Regulation of KIT signaling | | | 4/505 | | 16/10867 | | 5.4E-03 | | 0.18 | | 0.17 | | FYN/KITLG/LCK/PTPN6 | | 4 | |
| R-HSA-399954 | | Sema3A PAK dependent Axon repulsion | | | 4/505 | | 16/10867 | | 5.4E-03 | | 0.18 | | 0.17 | | CFL1/FYN/HSP90AA1/NRP1 | | 4 | |
| R-HSA-1227990 | | Signaling by ERBB2 in Cancer | | | 5/505 | | 26/10867 | | 6.2E-03 | | 0.19 | | 0.18 | | ERBB3/ERBB4/ERBIN/HSP90AA1/PIK3R1 | | 5 | |
| R-HSA-2029480 | | Fcgamma receptor (FCGR) dependent phagocytosis | | | 10/505 | | 86/10867 | | 6.4E-03 | | 0.19 | | 0.18 | | ARPC4/BTK/CFL1/FYN/HCK/HSP90AA1/ITPR1/NCKAP1/PIK3R1/PLPP4 | | 10 | |
| R-HSA-9006931 | | Signaling by Nuclear Receptors | | | 24/505 | | 299/10867 | | 6.4E-03 | | 0.19 | | 0.18 | | ADH1A/ADH1C/ALDH1A1/CAV1/ERBB4/FOXA1/FOXO3/GNAI2/GNG12/GTF2F1/HSP90AA1/KPNA2/MYLIP/NOS3/NRIP1/PIK3R1/RUNX1/SCD/SP1/SRF/TFF1/TFF3/XPO1/YY1 | | 24 | |
| R-HSA-180292 | | GAB1 signalosome | | | 4/505 | | 17/10867 | | 6.8E-03 | | 0.19 | | 0.18 | | CSK/PAG1/PIK3R1/PXN | | 4 | |
| R-HSA-9617828 | | FOXO-mediated transcription of cell cycle genes | | | 4/505 | | 17/10867 | | 6.8E-03 | | 0.19 | | 0.18 | | CAV1/CDKN1A/FOXO1/FOXO3 | | 4 | |
| R-HSA-1500931 | | Cell-Cell communication | | | 13/505 | | 129/10867 | | 6.9E-03 | | 0.19 | | 0.18 | | CDH11/CDH17/CDH6/COL17A1/CTNNA1/CTNND1/DST/FERMT2/FYN/IQGAP1/PIK3R1/PTPN6/PXN | | 13 | |
| R-HSA-9013408 | | RHOG GTPase cycle | | | 9/505 | | 74/10867 | | 7.1E-03 | | 0.19 | | 0.18 | | ARHGAP32/ARHGDIA/CAV1/ERBIN/IQGAP2/KTN1/PIK3R1/RHOG/TFRC | | 9 | |
| R-HSA-3000170 | | Syndecan interactions | | | 5/505 | | 27/10867 | | 7.3E-03 | | 0.19 | | 0.18 | | COL1A1/COL5A1/ITGA2/ITGB5/THBS1 | | 5 | |
| R-HSA-9013404 | | RAC2 GTPase cycle | | | 10/505 | | 88/10867 | | 7.5E-03 | | 0.19 | | 0.18 | | ARHGAP32/ARHGDIA/CAV1/ERBIN/GIT2/IQGAP1/NCKAP1/PIK3R1/RAC2/TFRC | | 10 | |
| R-HSA-8864260 | | Transcriptional regulation by the AP-2 (TFAP2) family of transcription factors | | | 6/505 | | 38/10867 | | 7.6E-03 | | 0.19 | | 0.18 | | CDKN1A/CITED2/KDM5B/MYBL2/TFAP2A/YY1 | | 6 | |
| R-HSA-9013407 | | RHOH GTPase cycle | | | 6/505 | | 38/10867 | | 7.6E-03 | | 0.19 | | 0.18 | | ARHGDIA/CAV1/CSK/LCK/TFRC/TMEM59 | | 6 | |
| R-HSA-9607240 | | FLT3 Signaling | | | 6/505 | | 38/10867 | | 7.6E-03 | | 0.19 | | 0.18 | | CSK/FOXO3/FYN/HCK/LCK/PIK3R1 | | 6 | |
| R-HSA-9006936 | | Signaling by TGFB family members | | | 11/505 | | 102/10867 | | 7.6E-03 | | 0.19 | | 0.18 | | BMP2/BMPR2/FSTL1/INHBA/PMEPA1/RNF111/SP1/TGFBR1/TGFBR2/XPO1/ZFYVE16 | | 11 | |
| R-HSA-8939211 | | ESR-mediated signaling | | | 19/505 | | 223/10867 | | 7.9E-03 | | 0.19 | | 0.18 | | CAV1/ERBB4/FOXA1/FOXO3/GNAI2/GNG12/GTF2F1/HSP90AA1/KPNA2/NOS3/NRIP1/PIK3R1/RUNX1/SP1/SRF/TFF1/TFF3/XPO1/YY1 | | 19 | |
| R-HSA-112310 | | Neurotransmitter release cycle | | | 7/505 | | 51/10867 | | 8.8E-03 | | 0.20 | | 0.19 | | ABAT/ARL6IP5/RAB3A/SLC1A1/SLC38A2/SLC6A12/VAMP2 | | 7 | |
| R-HSA-9009391 | | Extra-nuclear estrogen signaling | | | 9/505 | | 77/10867 | | 9.1E-03 | | 0.20 | | 0.19 | | CAV1/FOXO3/GNAI2/GNG12/HSP90AA1/NOS3/PIK3R1/SRF/XPO1 | | 9 | |
| R-HSA-9020958 | | Interleukin-21 signaling | | | 3/505 | | 10/10867 | | 9.4E-03 | | 0.20 | | 0.19 | | IL21R/JAK1/STAT4 | | 3 | |
| R-HSA-9006925 | | Intracellular signaling by second messengers | | | 24/505 | | 309/10867 | | 9.5E-03 | | 0.20 | | 0.19 | | CAMK4/CDKN1A/ERBB3/ERBB4/FGF18/FOXO1/FOXO3/FRK/FRS2/FYN/GSK3B/ITPR1/KITLG/KPNA2/LCK/MBD3/MTA3/NEDD4/PIK3R1/PSMC3/RAC2/RHOG/RPS6KB2/SNAI2 | | 24 | |
| R-HSA-445355 | | Smooth Muscle Contraction | | | 6/505 | | 40/10867 | | 9.8E-03 | | 0.20 | | 0.19 | | ACTG2/CALD1/GUCY1A2/ITGB5/PXN/TLN1 | | 6 | |
| R-HSA-422356 | | Regulation of insulin secretion | | | 9/505 | | 78/10867 | | 9.9E-03 | | 0.20 | | 0.19 | | ACSL3/ACSL4/ADRA2A/GNAI2/GNAQ/GNG12/IQGAP1/ITPR1/VAMP2 | | 9 | |
| R-HSA-5218920 | | VEGFR2 mediated vascular permeability | | | 5/505 | | 29/10867 | | 1.0E-02 | | 0.20 | | 0.19 | | CAV1/CTNNA1/CTNND1/HSP90AA1/NOS3 | | 5 | |
| R-HSA-5683826 | | Surfactant metabolism | | | 5/505 | | 29/10867 | | 1.0E-02 | | 0.20 | | 0.19 | | ADGRF5/ADRA2A/CKAP4/CSF2RA/CTSH | | 5 | |
| R-HSA-9683701 | | Translation of Structural Proteins | | | 5/505 | | 29/10867 | | 1.0E-02 | | 0.20 | | 0.19 | | GALNT1/GSK3B/PARP8/ST3GAL2/ST6GALNAC3 | | 5 | |
| R-HSA-9614085 | | FOXO-mediated transcription | | | 8/505 | | 65/10867 | | 1.0E-02 | | 0.20 | | 0.19 | | ABCA6/CAV1/CDKN1A/CITED2/FOXO1/FOXO3/SIRT1/TXNIP | | 8 | |
| R-HSA-174414 | | Processive synthesis on the C-strand of the telomere | | | 4/505 | | 19/10867 | | 1.0E-02 | | 0.20 | | 0.19 | | BLM/PCNA/POLD4/TERF2IP | | 4 | |
| R-HSA-888590 | | GABA synthesis. release. reuptake and degradation | | | 4/505 | | 19/10867 | | 1.0E-02 | | 0.20 | | 0.19 | | ABAT/RAB3A/SLC6A12/VAMP2 | | 4 | |
| R-HSA-525793 | | Myogenesis | | | 5/505 | | 30/10867 | | 1.2E-02 | | 0.23 | | 0.21 | | BOC/CTNNA1/MEF2C/TCF12/TCF4 | | 5 | |
| R-HSA-9013149 | | RAC1 GTPase cycle | | | 16/505 | | 185/10867 | | 1.2E-02 | | 0.24 | | 0.22 | | AMIGO2/ARHGAP12/ARHGAP20/ARHGAP32/ARHGDIA/CAV1/ERBIN/FAM13B/FERMT2/GIT2/IQGAP1/IQGAP2/KTN1/NCKAP1/PIK3R1/TFRC | | 16 | |
| R-HSA-400451 | | Free fatty acids regulate insulin secretion | | | 3/505 | | 11/10867 | | 1.2E-02 | | 0.24 | | 0.22 | | ACSL3/ACSL4/GNAQ | | 3 | |
| R-HSA-1257604 | | PIP3 activates AKT signaling | | | 21/505 | | 267/10867 | | 1.3E-02 | | 0.24 | | 0.22 | | CDKN1A/ERBB3/ERBB4/FGF18/FOXO1/FOXO3/FRK/FRS2/FYN/GSK3B/KITLG/LCK/MBD3/MTA3/NEDD4/PIK3R1/PSMC3/RAC2/RHOG/RPS6KB2/SNAI2 | | 21 | |
| R-HSA-9700206 | | Signaling by ALK in cancer | | | 7/505 | | 55/10867 | | 1.3E-02 | | 0.24 | | 0.22 | | CLTC/FRS2/GCC2/MSN/PIK3R1/RANBP2/SQSTM1 | | 7 | |
| R-HSA-9725370 | | Signaling by ALK fusions and activated point mutants | | | 7/505 | | 55/10867 | | 1.3E-02 | | 0.24 | | 0.22 | | CLTC/FRS2/GCC2/MSN/PIK3R1/RANBP2/SQSTM1 | | 7 | |
| R-HSA-5339562 | | Uptake and actions of bacterial toxins | | | 5/505 | | 31/10867 | | 1.3E-02 | | 0.24 | | 0.22 | | ANTXR1/ANTXR2/HSP90AA1/VAMP1/VAMP2 | | 5 | |
| R-HSA-432722 | | Golgi Associated Vesicle Biogenesis | | | 7/505 | | 56/10867 | | 1.4E-02 | | 0.26 | | 0.24 | | AP1S1/AP3B1/CLTC/CPD/SH3D19/TFRC/VAMP2 | | 7 | |
| R-HSA-6798695 | | Neutrophil degranulation | | | 33/505 | | 480/10867 | | 1.5E-02 | | 0.26 | | 0.24 | | ADAM10/ASAH1/C1orf35/CAB39/CD33/CFD/CKAP4/CST3/CTSH/FGL2/FRK/HSP90AA1/HVCN1/IDH1/IQGAP1/IQGAP2/ITGB2/MAN2B1/NBEAL2/PAFAH1B2/PLAU/PSAP/PSMC3/PTGES2/PTPN6/PTPRB/RAB14/RAB3A/RAB5B/RHOG/TBC1D10C/TMEM30A/TRAPPC1 | | 33 | |
| R-HSA-5696399 | | Global Genome Nucleotide Excision Repair (GG-NER) | | | 9/505 | | 84/10867 | | 1.6E-02 | | 0.26 | | 0.24 | | CCNH/COPS6/ERCC4/PCNA/POLD4/RFC5/RNF111/XRCC1/YY1 | | 9 | |
| R-HSA-210990 | | PECAM1 interactions | | | 3/505 | | 12/10867 | | 1.6E-02 | | 0.26 | | 0.24 | | FYN/LCK/PTPN6 | | 3 | |
| R-HSA-2160916 | | Hyaluronan uptake and degradation | | | 3/505 | | 12/10867 | | 1.6E-02 | | 0.26 | | 0.24 | | CHP1/HMMR/HYAL1 | | 3 | |
| R-HSA-71384 | | Ethanol oxidation | | | 3/505 | | 12/10867 | | 1.6E-02 | | 0.26 | | 0.24 | | ADH1A/ADH1C/ALDH1A1 | | 3 | |
| R-HSA-8984722 | | Interleukin-35 Signalling | | | 3/505 | | 12/10867 | | 1.6E-02 | | 0.26 | | 0.24 | | IL6ST/JAK1/STAT4 | | 3 | |
| R-HSA-4420097 | | VEGFA-VEGFR2 Pathway | | | 10/505 | | 99/10867 | | 1.6E-02 | | 0.26 | | 0.24 | | CAV1/CTNNA1/CTNND1/FYN/HSP90AA1/ITPR1/NCKAP1/NOS3/PIK3R1/PXN | | 10 | |
| R-HSA-983169 | | Class I MHC mediated antigen processing & presentation | | | 27/505 | | 377/10867 | | 1.7E-02 | | 0.26 | | 0.24 | | BTK/CCNF/CUL3/FBXL5/FBXL7/FBXL8/FBXO22/FBXO31/HECTD1/HERC3/ITGB5/KLHL2/MYLIP/NEDD4/PJA2/PSMC3/RNF111/RNF217/SEC23A/SEC24A/SEC24B/SEC24C/SEC24D/TLR4/TRIM11/TRIM41/UBE4A | | 27 | |
| R-HSA-418990 | | Adherens junctions interactions | | | 5/505 | | 33/10867 | | 1.7E-02 | | 0.26 | | 0.24 | | CDH11/CDH17/CDH6/CTNNA1/CTNND1 | | 5 | |
| R-HSA-202427 | | Phosphorylation of CD3 and TCR zeta chains | | | 4/505 | | 22/10867 | | 1.7E-02 | | 0.26 | | 0.24 | | CSK/HLA-DPB1/LCK/PAG1 | | 4 | |
| R-HSA-392154 | | Nitric oxide stimulates guanylate cyclase | | | 4/505 | | 22/10867 | | 1.7E-02 | | 0.26 | | 0.24 | | GUCY1A2/ITPR1/NOS3/PRKG1 | | 4 | |
| R-HSA-9665686 | | Signaling by ERBB2 TMD/JMD mutants | | | 4/505 | | 22/10867 | | 1.7E-02 | | 0.26 | | 0.24 | | ERBB3/ERBB4/ERBIN/HSP90AA1 | | 4 | |
| R-HSA-1236394 | | Signaling by ERBB4 | | | 7/505 | | 58/10867 | | 1.7E-02 | | 0.26 | | 0.24 | | ERBB3/ERBB4/MXD4/NEDD4/PIK3R1/SPARC/STMN1 | | 7 | |
| R-HSA-174417 | | Telomere C-strand (Lagging Strand) Synthesis | | | 5/505 | | 34/10867 | | 1.9E-02 | | 0.29 | | 0.26 | | BLM/PCNA/POLD4/RFC5/TERF2IP | | 5 | |
| R-HSA-112409 | | RAF-independent MAPK1/3 activation | | | 4/505 | | 23/10867 | | 2.0E-02 | | 0.29 | | 0.27 | | DUSP10/DUSP4/IL6ST/JAK1 | | 4 | |
| R-HSA-389948 | | PD-1 signaling | | | 4/505 | | 23/10867 | | 2.0E-02 | | 0.29 | | 0.27 | | CSK/HLA-DPB1/LCK/PTPN6 | | 4 | |
| R-HSA-512988 | | Interleukin-3. Interleukin-5 and GM-CSF signaling | | | 6/505 | | 48/10867 | | 2.3E-02 | | 0.30 | | 0.28 | | CSF2RA/FYN/HCK/JAK1/PIK3R1/PTPN6 | | 6 | |
| R-HSA-9679506 | | SARS-CoV Infections | | | 13/505 | | 151/10867 | | 2.4E-02 | | 0.30 | | 0.28 | | CHMP3/CSNK1A1/GALNT1/GSK3B/HSP90AA1/IFNGR1/IL1R1/JAK1/MBD3/MTA3/PARP8/ST3GAL2/ST6GALNAC3 | | 13 | |
| R-HSA-8876198 | | RAB GEFs exchange GTP for GDP on RABs | | | 9/505 | | 90/10867 | | 2.4E-02 | | 0.30 | | 0.28 | | RAB14/RAB27B/RAB3A/RAB3IL1/RAB3IP/RAB5B/RAB8B/RIN2/TRAPPC1 | | 9 | |
| R-HSA-399955 | | SEMA3A-Plexin repulsion signaling by inhibiting Integrin adhesion | | | 3/505 | | 14/10867 | | 2.5E-02 | | 0.30 | | 0.28 | | FYN/NRP1/TLN1 | | 3 | |
| R-HSA-4839735 | | Signaling by AXIN mutants | | | 3/505 | | 14/10867 | | 2.5E-02 | | 0.30 | | 0.28 | | APC/CSNK1A1/GSK3B | | 3 | |
| R-HSA-4839744 | | Signaling by APC mutants | | | 3/505 | | 14/10867 | | 2.5E-02 | | 0.30 | | 0.28 | | APC/CSNK1A1/GSK3B | | 3 | |
| R-HSA-4839748 | | Signaling by AMER1 mutants | | | 3/505 | | 14/10867 | | 2.5E-02 | | 0.30 | | 0.28 | | APC/CSNK1A1/GSK3B | | 3 | |
| R-HSA-5358565 | | Mismatch repair (MMR) directed by MSH2:MSH6 (MutSalpha) | | | 3/505 | | 14/10867 | | 2.5E-02 | | 0.30 | | 0.28 | | MSH6/PCNA/POLD4 | | 3 | |
| R-HSA-5467337 | | APC truncation mutants have impaired AXIN binding | | | 3/505 | | 14/10867 | | 2.5E-02 | | 0.30 | | 0.28 | | APC/CSNK1A1/GSK3B | | 3 | |
| R-HSA-5467340 | | AXIN missense mutants destabilize the destruction complex | | | 3/505 | | 14/10867 | | 2.5E-02 | | 0.30 | | 0.28 | | APC/CSNK1A1/GSK3B | | 3 | |
| R-HSA-5467348 | | Truncations of AMER1 destabilize the destruction complex | | | 3/505 | | 14/10867 | | 2.5E-02 | | 0.30 | | 0.28 | | APC/CSNK1A1/GSK3B | | 3 | |
| R-HSA-6804116 | | TP53 Regulates Transcription of Genes Involved in G1 Cell Cycle Arrest | | | 3/505 | | 14/10867 | | 2.5E-02 | | 0.30 | | 0.28 | | CCNE2/CDKN1A/ZNF385A | | 3 | |
| R-HSA-69091 | | Polymerase switching | | | 3/505 | | 14/10867 | | 2.5E-02 | | 0.30 | | 0.28 | | PCNA/POLD4/RFC5 | | 3 | |
| R-HSA-69109 | | Leading Strand Synthesis | | | 3/505 | | 14/10867 | | 2.5E-02 | | 0.30 | | 0.28 | | PCNA/POLD4/RFC5 | | 3 | |
| R-HSA-877312 | | Regulation of IFNG signaling | | | 3/505 | | 14/10867 | | 2.5E-02 | | 0.30 | | 0.28 | | IFNGR1/JAK1/PTPN6 | | 3 | |
| R-HSA-9694635 | | Translation of Structural Proteins | | | 6/505 | | 49/10867 | | 2.5E-02 | | 0.30 | | 0.28 | | CSNK1A1/GALNT1/GSK3B/PARP8/ST3GAL2/ST6GALNAC3 | | 6 | |
| R-HSA-446728 | | Cell junction organization | | | 9/505 | | 91/10867 | | 2.5E-02 | | 0.30 | | 0.28 | | CDH11/CDH17/CDH6/COL17A1/CTNNA1/CTNND1/DST/FERMT2/PXN | | 9 | |
| R-HSA-6811558 | | PI5P. PP2A and IER3 Regulate PI3K/AKT Signaling | | | 10/505 | | 106/10867 | | 2.5E-02 | | 0.30 | | 0.28 | | ERBB3/ERBB4/FGF18/FRS2/FYN/KITLG/LCK/PIK3R1/RAC2/RHOG | | 10 | |
| R-HSA-202733 | | Cell surface interactions at the vascular wall | | | 12/505 | | 137/10867 | | 2.6E-02 | | 0.30 | | 0.28 | | BSG/CAV1/CD48/COL1A1/CXADR/FYN/ITGB2/LCK/PIK3R1/PROS1/PTPN6/SELPLG | | 12 | |
| R-HSA-446219 | | Synthesis of substrates in N-glycan biosythesis | | | 7/505 | | 63/10867 | | 2.6E-02 | | 0.30 | | 0.28 | | AMDHD2/GMPPB/RENBP/ST3GAL2/ST6GALNAC1/ST6GALNAC3/ST8SIA4 | | 7 | |
| R-HSA-5696397 | | Gap-filling DNA repair synthesis and ligation in GG-NER | | | 4/505 | | 25/10867 | | 2.7E-02 | | 0.30 | | 0.28 | | PCNA/POLD4/RFC5/XRCC1 | | 4 | |
| R-HSA-75105 | | Fatty acyl-CoA biosynthesis | | | 5/505 | | 37/10867 | | 2.7E-02 | | 0.30 | | 0.28 | | ACACA/ACSL3/ACSL4/HACD2/SCD | | 5 | |
| R-HSA-1227986 | | Signaling by ERBB2 | | | 6/505 | | 50/10867 | | 2.7E-02 | | 0.30 | | 0.28 | | ERBB3/ERBB4/ERBIN/FYN/HSP90AA1/PIK3R1 | | 6 | |
| R-HSA-373752 | | Netrin-1 signaling | | | 6/505 | | 50/10867 | | 2.7E-02 | | 0.30 | | 0.28 | | ABLIM3/FYN/NTN4/RGMA/RGMB/SLIT2 | | 6 | |
| R-HSA-446193 | | Biosynthesis of the N-glycan precursor (dolichol lipid-linked oligosaccharide. LLO) and transfer to a nascent protein | | | 8/505 | | 78/10867 | | 2.8E-02 | | 0.30 | | 0.28 | | ALG13/AMDHD2/GMPPB/RENBP/ST3GAL2/ST6GALNAC1/ST6GALNAC3/ST8SIA4 | | 8 | |
| R-HSA-9007101 | | Rab regulation of trafficking | | | 11/505 | | 124/10867 | | 2.9E-02 | | 0.30 | | 0.28 | | RAB11A/RAB14/RAB27B/RAB3A/RAB3IL1/RAB3IP/RAB5B/RAB8B/RIN2/TBC1D10C/TRAPPC1 | | 11 | |
| R-HSA-3772470 | | Negative regulation of TCF-dependent signaling by WNT ligand antagonists | | | 3/505 | | 15/10867 | | 3.0E-02 | | 0.30 | | 0.28 | | KREMEN1/SFRP1/WNT5A | | 3 | |
| R-HSA-4839743 | | Signaling by CTNNB1 phospho-site mutants | | | 3/505 | | 15/10867 | | 3.0E-02 | | 0.30 | | 0.28 | | APC/CSNK1A1/GSK3B | | 3 | |
| R-HSA-5339716 | | Signaling by GSK3beta mutants | | | 3/505 | | 15/10867 | | 3.0E-02 | | 0.30 | | 0.28 | | APC/CSNK1A1/GSK3B | | 3 | |
| R-HSA-5358508 | | Mismatch Repair | | | 3/505 | | 15/10867 | | 3.0E-02 | | 0.30 | | 0.28 | | MSH6/PCNA/POLD4 | | 3 | |
| R-HSA-5358747 | | S33 mutants of beta-catenin aren't phosphorylated | | | 3/505 | | 15/10867 | | 3.0E-02 | | 0.30 | | 0.28 | | APC/CSNK1A1/GSK3B | | 3 | |
| R-HSA-5358749 | | S37 mutants of beta-catenin aren't phosphorylated | | | 3/505 | | 15/10867 | | 3.0E-02 | | 0.30 | | 0.28 | | APC/CSNK1A1/GSK3B | | 3 | |
| R-HSA-5358751 | | S45 mutants of beta-catenin aren't phosphorylated | | | 3/505 | | 15/10867 | | 3.0E-02 | | 0.30 | | 0.28 | | APC/CSNK1A1/GSK3B | | 3 | |
| R-HSA-5358752 | | T41 mutants of beta-catenin aren't phosphorylated | | | 3/505 | | 15/10867 | | 3.0E-02 | | 0.30 | | 0.28 | | APC/CSNK1A1/GSK3B | | 3 | |
| R-HSA-75892 | | Platelet Adhesion to exposed collagen | | | 3/505 | | 15/10867 | | 3.0E-02 | | 0.30 | | 0.28 | | COL1A1/FYN/ITGA2 | | 3 | |
| R-HSA-9694631 | | Maturation of nucleoprotein | | | 3/505 | | 15/10867 | | 3.0E-02 | | 0.30 | | 0.28 | | CSNK1A1/GSK3B/PARP8 | | 3 | |
| R-HSA-9678108 | | SARS-CoV-1 Infection | | | 6/505 | | 51/10867 | | 3.0E-02 | | 0.30 | | 0.28 | | CHMP3/GALNT1/GSK3B/PARP8/ST3GAL2/ST6GALNAC3 | | 6 | |
| R-HSA-9013423 | | RAC3 GTPase cycle | | | 9/505 | | 94/10867 | | 3.0E-02 | | 0.30 | | 0.28 | | AMIGO2/ARHGAP32/CAV1/ERBIN/FERMT2/GIT2/NCKAP1/PIK3R1/TFRC | | 9 | |
| R-HSA-174411 | | Polymerase switching on the C-strand of the telomere | | | 4/505 | | 26/10867 | | 3.1E-02 | | 0.30 | | 0.28 | | PCNA/POLD4/RFC5/TERF2IP | | 4 | |
| R-HSA-2173788 | | Downregulation of TGF-beta receptor signaling | | | 4/505 | | 26/10867 | | 3.1E-02 | | 0.30 | | 0.28 | | PMEPA1/TGFBR1/TGFBR2/XPO1 | | 4 | |
| R-HSA-912526 | | Interleukin receptor SHC signaling | | | 4/505 | | 27/10867 | | 3.5E-02 | | 0.34 | | 0.31 | | CSF2RA/JAK1/PIK3R1/PTPN6 | | 4 | |
| R-HSA-1963642 | | PI3K events in ERBB2 signaling | | | 3/505 | | 16/10867 | | 3.6E-02 | | 0.34 | | 0.31 | | ERBB3/ERBB4/PIK3R1 | | 3 | |
| R-HSA-399956 | | CRMPs in Sema3A signaling | | | 3/505 | | 16/10867 | | 3.6E-02 | | 0.34 | | 0.31 | | FYN/GSK3B/NRP1 | | 3 | |
| R-HSA-9614657 | | FOXO-mediated transcription of cell death genes | | | 3/505 | | 16/10867 | | 3.6E-02 | | 0.34 | | 0.31 | | CITED2/FOXO1/FOXO3 | | 3 | |
| R-HSA-9665348 | | Signaling by ERBB2 ECD mutants | | | 3/505 | | 16/10867 | | 3.6E-02 | | 0.34 | | 0.31 | | ERBIN/HSP90AA1/PIK3R1 | | 3 | |
| R-HSA-1483166 | | Synthesis of PA | | | 5/505 | | 40/10867 | | 3.7E-02 | | 0.34 | | 0.32 | | GPD1L/LIPH/MIGA2/PLA2G2A/PLA2G4A | | 5 | |
| R-HSA-9013420 | | RHOU GTPase cycle | | | 5/505 | | 40/10867 | | 3.7E-02 | | 0.34 | | 0.32 | | CLTC/DST/GIT2/IQGAP1/PIK3R1 | | 5 | |
| R-HSA-199418 | | Negative regulation of the PI3K/AKT network | | | 10/505 | | 113/10867 | | 3.7E-02 | | 0.35 | | 0.32 | | ERBB3/ERBB4/FGF18/FRS2/FYN/KITLG/LCK/PIK3R1/RAC2/RHOG | | 10 | |
| R-HSA-204005 | | COPII-mediated vesicle transport | | | 7/505 | | 68/10867 | | 3.8E-02 | | 0.35 | | 0.32 | | SEC16A/SEC23A/SEC24A/SEC24B/SEC24C/SEC24D/TRAPPC1 | | 7 | |
| R-HSA-164952 | | The role of Nef in HIV-1 replication and disease pathogenesis | | | 4/505 | | 28/10867 | | 3.9E-02 | | 0.35 | | 0.33 | | AP1S1/FYN/HCK/LCK | | 4 | |
| R-HSA-201451 | | Signaling by BMP | | | 4/505 | | 28/10867 | | 3.9E-02 | | 0.35 | | 0.33 | | BMP2/BMPR2/FSTL1/ZFYVE16 | | 4 | |
| R-HSA-9013409 | | RHOJ GTPase cycle | | | 6/505 | | 55/10867 | | 4.1E-02 | | 0.36 | | 0.34 | | ARHGAP32/CAV1/FMNL3/GIT2/PIK3R1/TFRC | | 6 | |
| R-HSA-174437 | | Removal of the Flap Intermediate from the C-strand | | | 3/505 | | 17/10867 | | 4.2E-02 | | 0.36 | | 0.34 | | PCNA/POLD4/TERF2IP | | 3 | |
| R-HSA-196299 | | Beta-catenin phosphorylation cascade | | | 3/505 | | 17/10867 | | 4.2E-02 | | 0.36 | | 0.34 | | APC/CSNK1A1/GSK3B | | 3 | |
| R-HSA-2142845 | | Hyaluronan metabolism | | | 3/505 | | 17/10867 | | 4.2E-02 | | 0.36 | | 0.34 | | CHP1/HMMR/HYAL1 | | 3 | |
| R-HSA-9659787 | | Aberrant regulation of mitotic G1/S transition in cancer due to RB1 defects | | | 3/505 | | 17/10867 | | 4.2E-02 | | 0.36 | | 0.34 | | CCND2/CCNE2/CDKN1A | | 3 | |
| R-HSA-9661069 | | Defective binding of RB1 mutants to E2F1.(E2F2. E2F3) | | | 3/505 | | 17/10867 | | 4.2E-02 | | 0.36 | | 0.34 | | CCND2/CCNE2/CDKN1A | | 3 | |
| R-HSA-198933 | | Immunoregulatory interactions between a Lymphoid and a non-Lymphoid cell | | | 11/505 | | 132/10867 | | 4.3E-02 | | 0.37 | | 0.34 | | CD1C/CD33/CD8A/COL17A1/COL1A1/CXADR/ITGB2/KLRG1/TREML1/TREML2/VCAM1 | | 11 | |
| R-HSA-216083 | | Integrin cell surface interactions | | | 8/505 | | 85/10867 | | 4.3E-02 | | 0.37 | | 0.34 | | BSG/COL1A1/COL5A1/ITGA2/ITGB2/ITGB5/THBS1/VCAM1 | | 8 | |
| R-HSA-8863795 | | Downregulation of ERBB2 signaling | | | 4/505 | | 29/10867 | | 4.4E-02 | | 0.37 | | 0.34 | | ERBB3/ERBB4/ERBIN/HSP90AA1 | | 4 | |
| R-HSA-112040 | | G-protein mediated events | | | 6/505 | | 56/10867 | | 4.5E-02 | | 0.38 | | 0.35 | | CAMK4/GNAI2/GNAQ/ITPR1/KPNA2/PLA2G4A | | 6 | |
| R-HSA-166520 | | Signaling by NTRKs | | | 11/505 | | 134/10867 | | 4.7E-02 | | 0.40 | | 0.37 | | CLTC/DUSP4/F3/FRS2/FYN/MEF2C/NELFB/PCSK5/PIK3R1/SRF/TCF12 | | 11 | |
| R-HSA-1433557 | | Signaling by SCF-KIT | | | 5/505 | | 43/10867 | | 4.8E-02 | | 0.40 | | 0.37 | | FYN/KITLG/LCK/PIK3R1/PTPN6 | | 5 | |
| R-HSA-447115 | | Interleukin-12 family signaling | | | 6/505 | | 57/10867 | | 4.8E-02 | | 0.40 | | 0.37 | | CFL1/IL10/IL6ST/JAK1/MSN/STAT4 | | 6 | |
| R-HSA-5654710 | | PI-3K cascade:FGFR3 | | | 3/505 | | 18/10867 | | 4.8E-02 | | 0.40 | | 0.37 | | FGF18/FRS2/PIK3R1 | | 3 | |
| R-HSA-2424491 | | DAP12 signaling | | | 4/505 | | 30/10867 | | 4.9E-02 | | 0.40 | | 0.37 | | BTK/FYN/LCK/PIK3R1 | | 4 | |
| R-HSA-8957322 | | Metabolism of steroids | | | 12/505 | | 151/10867 | | 4.9E-02 | | 0.40 | | 0.37 | | ACACA/DHCR24/FDXR/INSIG2/MSMO1/SCD/SEC23A/SEC24A/SEC24B/SEC24C/SEC24D/SP1 | | 12 | |
| R-HSA-199992 | | trans-Golgi Network Vesicle Budding | | | 7/505 | | 72/10867 | | 4.9E-02 | | 0.40 | | 0.37 | | AP1S1/AP3B1/CLTC/CPD/SH3D19/TFRC/VAMP2 | | 7 | |
| R-HSA-9694516 | | SARS-CoV-2 Infection | | | 7/505 | | 72/10867 | | 4.9E-02 | | 0.40 | | 0.37 | | CHMP3/CSNK1A1/GALNT1/GSK3B/PARP8/ST3GAL2/ST6GALNAC3 | | 7 | |
| R-HSA-418346 | | Platelet homeostasis | | | 8/505 | | 88/10867 | | 5.2E-02 | | 0.41 | | 0.38 | | ATP2A2/GNG12/GUCY1A2/ITPR1/NOS3/PLA2G4A/PRKG1/PTPN6 | | 8 | |
| R-HSA-4641262 | | Disassembly of the destruction complex and recruitment of AXIN to the membrane | | | 4/505 | | 31/10867 | | 5.4E-02 | | 0.43 | | 0.39 | | APC/CAV1/CSNK1A1/GSK3B | | 4 | |
| R-HSA-3000171 | | Non-integrin membrane-ECM interactions | | | 6/505 | | 59/10867 | | 5.5E-02 | | 0.44 | | 0.40 | | COL1A1/COL5A1/ITGA2/ITGB5/NTN4/THBS1 | | 6 | |
| R-HSA-2172127 | | DAP12 interactions | | | 5/505 | | 45/10867 | | 5.6E-02 | | 0.44 | | 0.41 | | BTK/FYN/LCK/PIK3R1/SIGLEC15 | | 5 | |
| R-HSA-1251985 | | Nuclear signaling by ERBB4 | | | 4/505 | | 32/10867 | | 5.9E-02 | | 0.46 | | 0.42 | | ERBB4/MXD4/SPARC/STMN1 | | 4 | |
| R-HSA-2173789 | | TGF-beta receptor signaling activates SMADs | | | 4/505 | | 32/10867 | | 5.9E-02 | | 0.46 | | 0.42 | | PMEPA1/TGFBR1/TGFBR2/XPO1 | | 4 | |
| R-HSA-5626467 | | RHO GTPases activate IQGAPs | | | 4/505 | | 32/10867 | | 5.9E-02 | | 0.46 | | 0.42 | | CLIP1/CTNNA1/IQGAP1/IQGAP2 | | 4 | |
| R-HSA-168255 | | Influenza Infection | | | 12/505 | | 156/10867 | | 5.9E-02 | | 0.46 | | 0.42 | | CLTC/GTF2F1/HSP90AA1/KPNA1/KPNA2/RANBP2/RPL32/RPL36AL/RPS14/RPS21/RPS9/XPO1 | | 12 | |
| R-HSA-2132295 | | MHC class II antigen presentation | | | 10/505 | | 123/10867 | | 6.0E-02 | | 0.46 | | 0.42 | | AP1S1/CLTC/CTSH/HLA-DPB1/KIF3B/SEC23A/SEC24A/SEC24B/SEC24C/SEC24D | | 10 | |
| R-HSA-112382 | | Formation of RNA Pol II elongation complex | | | 6/505 | | 61/10867 | | 6.3E-02 | | 0.46 | | 0.42 | | AFF4/CCNH/EAF1/GTF2F1/NELFB/SUPT16H | | 6 | |
| R-HSA-75955 | | RNA Polymerase II Transcription Elongation | | | 6/505 | | 61/10867 | | 6.3E-02 | | 0.46 | | 0.42 | | AFF4/CCNH/EAF1/GTF2F1/NELFB/SUPT16H | | 6 | |
| R-HSA-2022870 | | Chondroitin sulfate biosynthesis | | | 3/505 | | 20/10867 | | 6.3E-02 | | 0.46 | | 0.42 | | CHST15/CHST7/VCAN | | 3 | |
| R-HSA-5654720 | | PI-3K cascade:FGFR4 | | | 3/505 | | 20/10867 | | 6.3E-02 | | 0.46 | | 0.42 | | FGF18/FRS2/PIK3R1 | | 3 | |
| R-HSA-69186 | | Lagging Strand Synthesis | | | 3/505 | | 20/10867 | | 6.3E-02 | | 0.46 | | 0.42 | | PCNA/POLD4/RFC5 | | 3 | |
| R-HSA-8978934 | | Metabolism of cofactors | | | 3/505 | | 20/10867 | | 6.3E-02 | | 0.46 | | 0.42 | | HSP90AA1/IDH1/NOS3 | | 3 | |
| R-HSA-9669938 | | Signaling by KIT in disease | | | 3/505 | | 20/10867 | | 6.3E-02 | | 0.46 | | 0.42 | | FYN/LCK/PIK3R1 | | 3 | |
| R-HSA-9670439 | | Signaling by phosphorylated juxtamembrane. extracellular and kinase domain KIT mutants | | | 3/505 | | 20/10867 | | 6.3E-02 | | 0.46 | | 0.42 | | FYN/LCK/PIK3R1 | | 3 | |
| R-HSA-6804756 | | Regulation of TP53 Activity through Phosphorylation | | | 8/505 | | 92/10867 | | 6.4E-02 | | 0.46 | | 0.42 | | BLM/NUAK1/PRKAA1/PRKAA2/RAD50/RFC5/SUPT16H/TP53INP1 | | 8 | |
| R-HSA-392518 | | Signal amplification | | | 4/505 | | 33/10867 | | 6.5E-02 | | 0.46 | | 0.43 | | GNAI2/GNAQ/GNG12/PLA2G4A | | 4 | |
| R-HSA-4085001 | | Sialic acid metabolism | | | 4/505 | | 33/10867 | | 6.5E-02 | | 0.46 | | 0.43 | | ST3GAL2/ST6GALNAC1/ST6GALNAC3/ST8SIA4 | | 4 | |
| R-HSA-6783783 | | Interleukin-10 signaling | | | 5/505 | | 47/10867 | | 6.6E-02 | | 0.46 | | 0.43 | | CXCL8/IL10/IL10RA/IL1R1/JAK1 | | 5 | |
| R-HSA-9020591 | | Interleukin-12 signaling | | | 5/505 | | 47/10867 | | 6.6E-02 | | 0.46 | | 0.43 | | CFL1/IL10/JAK1/MSN/STAT4 | | 5 | |
| R-HSA-9707616 | | Heme signaling | | | 5/505 | | 47/10867 | | 6.6E-02 | | 0.46 | | 0.43 | | MEF2C/NRIP1/SIRT1/TLR4/XPO1 | | 5 | |
| R-HSA-1474244 | | Extracellular matrix organization | | | 20/505 | | 301/10867 | | 6.8E-02 | | 0.48 | | 0.44 | | ADAM10/ADAM12/BMP2/BMP4/BSG/COL17A1/COL1A1/COL5A1/DST/HTRA1/ITGA2/ITGB2/ITGB5/MFAP3/NTN4/PHYKPL/SPARC/THBS1/VCAM1/VCAN | | 20 | |
| R-HSA-6791312 | | TP53 Regulates Transcription of Cell Cycle Genes | | | 5/505 | | 48/10867 | | 7.1E-02 | | 0.49 | | 0.45 | | BTG2/CCNE2/CDKN1A/PCNA/ZNF385A | | 5 | |
| R-HSA-4791275 | | Signaling by WNT in cancer | | | 4/505 | | 34/10867 | | 7.1E-02 | | 0.49 | | 0.45 | | APC/CSNK1A1/GSK3B/KREMEN1 | | 4 | |
| R-HSA-9675135 | | Diseases of DNA repair | | | 4/505 | | 34/10867 | | 7.1E-02 | | 0.49 | | 0.45 | | BLM/MSH6/PALB2/RAD50 | | 4 | |
| R-HSA-5651801 | | PCNA-Dependent Long Patch Base Excision Repair | | | 3/505 | | 21/10867 | | 7.1E-02 | | 0.49 | | 0.45 | | PCNA/POLD4/RFC5 | | 3 | |
| R-HSA-5696398 | | Nucleotide Excision Repair | | | 9/505 | | 111/10867 | | 7.3E-02 | | 0.49 | | 0.45 | | CCNH/COPS6/ERCC4/PCNA/POLD4/RFC5/RNF111/XRCC1/YY1 | | 9 | |
| R-HSA-6781827 | | Transcription-Coupled Nucleotide Excision Repair (TC-NER) | | | 7/505 | | 79/10867 | | 7.4E-02 | | 0.49 | | 0.45 | | CCNH/COPS6/ERCC4/PCNA/POLD4/RFC5/XRCC1 | | 7 | |
| R-HSA-110056 | | MAPK3 (ERK1) activation | | | 2/505 | | 10/10867 | | 7.6E-02 | | 0.49 | | 0.45 | | IL6ST/JAK1 | | 2 | |
| R-HSA-1250342 | | PI3K events in ERBB4 signaling | | | 2/505 | | 10/10867 | | 7.6E-02 | | 0.49 | | 0.45 | | ERBB4/PIK3R1 | | 2 | |
| R-HSA-168799 | | Neurotoxicity of clostridium toxins | | | 2/505 | | 10/10867 | | 7.6E-02 | | 0.49 | | 0.45 | | VAMP1/VAMP2 | | 2 | |
| R-HSA-2470946 | | Cohesin Loading onto Chromatin | | | 2/505 | | 10/10867 | | 7.6E-02 | | 0.49 | | 0.45 | | PDS5B/WAPL | | 2 | |
| R-HSA-2562578 | | TRIF-mediated programmed cell death | | | 2/505 | | 10/10867 | | 7.6E-02 | | 0.49 | | 0.45 | | RIPK3/TLR4 | | 2 | |
| R-HSA-419812 | | Calcitonin-like ligand receptors | | | 2/505 | | 10/10867 | | 7.6E-02 | | 0.49 | | 0.45 | | CALCRL/RAMP2 | | 2 | |
| R-HSA-442729 | | CREB1 phosphorylation through the activation of CaMKII/CaMKK/CaMKIV cascasde | | | 2/505 | | 10/10867 | | 7.6E-02 | | 0.49 | | 0.45 | | CAMK4/KPNA2 | | 2 | |
| R-HSA-5685939 | | HDR through MMEJ (alt-NHEJ) | | | 2/505 | | 10/10867 | | 7.6E-02 | | 0.49 | | 0.45 | | RAD50/XRCC1 | | 2 | |
| R-HSA-8853334 | | Signaling by FGFR3 fusions in cancer | | | 2/505 | | 10/10867 | | 7.6E-02 | | 0.49 | | 0.45 | | FRS2/PIK3R1 | | 2 | |
| R-HSA-8877330 | | RUNX1 and FOXP3 control the development of regulatory T lymphocytes (Tregs) | | | 2/505 | | 10/10867 | | 7.6E-02 | | 0.49 | | 0.45 | | FOXP3/RUNX1 | | 2 | |
| R-HSA-9706574 | | RHOBTB GTPase Cycle | | | 4/505 | | 35/10867 | | 7.8E-02 | | 0.49 | | 0.46 | | CUL3/HSP90AA1/RNF20/VIM | | 4 | |
| R-HSA-389357 | | CD28 dependent PI3K/Akt signaling | | | 3/505 | | 22/10867 | | 8.0E-02 | | 0.50 | | 0.46 | | FYN/LCK/PIK3R1 | | 3 | |
| R-HSA-5365859 | | RA biosynthesis pathway | | | 3/505 | | 22/10867 | | 8.0E-02 | | 0.50 | | 0.46 | | ADH1A/ADH1C/ALDH1A1 | | 3 | |
| R-HSA-5655332 | | Signaling by FGFR3 in disease | | | 3/505 | | 22/10867 | | 8.0E-02 | | 0.50 | | 0.46 | | FGF18/FRS2/PIK3R1 | | 3 | |
| R-HSA-8853338 | | Signaling by FGFR3 point mutants in cancer | | | 3/505 | | 22/10867 | | 8.0E-02 | | 0.50 | | 0.46 | | FGF18/FRS2/PIK3R1 | | 3 | |
| R-HSA-912631 | | Regulation of signaling by CBL | | | 3/505 | | 22/10867 | | 8.0E-02 | | 0.50 | | 0.46 | | FYN/HCK/PIK3R1 | | 3 | |
| R-HSA-8873719 | | RAB geranylgeranylation | | | 6/505 | | 65/10867 | | 8.1E-02 | | 0.50 | | 0.46 | | RAB11A/RAB14/RAB27B/RAB3A/RAB5B/RAB8B | | 6 | |
| R-HSA-1266695 | | Interleukin-7 signaling | | | 4/505 | | 36/10867 | | 8.4E-02 | | 0.51 | | 0.47 | | BRWD1/IL7/JAK1/PIK3R1 | | 4 | |
| R-HSA-112043 | | PLC beta mediated events | | | 5/505 | | 51/10867 | | 8.7E-02 | | 0.51 | | 0.47 | | CAMK4/GNAQ/ITPR1/KPNA2/PLA2G4A | | 5 | |
| R-HSA-180786 | | Extension of Telomeres | | | 5/505 | | 51/10867 | | 8.7E-02 | | 0.51 | | 0.47 | | BLM/PCNA/POLD4/RFC5/TERF2IP | | 5 | |
| R-HSA-373753 | | Nephrin family interactions | | | 3/505 | | 23/10867 | | 8.9E-02 | | 0.51 | | 0.47 | | FYN/IQGAP1/PIK3R1 | | 3 | |
| R-HSA-5654695 | | PI-3K cascade:FGFR2 | | | 3/505 | | 23/10867 | | 8.9E-02 | | 0.51 | | 0.47 | | FGF18/FRS2/PIK3R1 | | 3 | |
| R-HSA-9013422 | | RHOBTB1 GTPase cycle | | | 3/505 | | 23/10867 | | 8.9E-02 | | 0.51 | | 0.47 | | CUL3/RNF20/VIM | | 3 | |
| R-HSA-1059683 | | Interleukin-6 signaling | | | 2/505 | | 11/10867 | | 9.0E-02 | | 0.51 | | 0.47 | | IL6ST/JAK1 | | 2 | |
| R-HSA-196780 | | Biotin transport and metabolism | | | 2/505 | | 11/10867 | | 9.0E-02 | | 0.51 | | 0.47 | | ACACA/SLC5A6 | | 2 | |
| R-HSA-2468052 | | Establishment of Sister Chromatid Cohesion | | | 2/505 | | 11/10867 | | 9.0E-02 | | 0.51 | | 0.47 | | PDS5B/WAPL | | 2 | |
| R-HSA-2514853 | | Condensation of Prometaphase Chromosomes | | | 2/505 | | 11/10867 | | 9.0E-02 | | 0.51 | | 0.47 | | CCNB2/SMC4 | | 2 | |
| R-HSA-446107 | | Type I hemidesmosome assembly | | | 2/505 | | 11/10867 | | 9.0E-02 | | 0.51 | | 0.47 | | COL17A1/DST | | 2 | |
| R-HSA-5625970 | | RHO GTPases activate KTN1 | | | 2/505 | | 11/10867 | | 9.0E-02 | | 0.51 | | 0.47 | | KTN1/RHOG | | 2 | |
| R-HSA-5655291 | | Signaling by FGFR4 in disease | | | 2/505 | | 11/10867 | | 9.0E-02 | | 0.51 | | 0.47 | | FRS2/PIK3R1 | | 2 | |
| R-HSA-9020956 | | Interleukin-27 signaling | | | 2/505 | | 11/10867 | | 9.0E-02 | | 0.51 | | 0.47 | | IL6ST/JAK1 | | 2 | |
| R-HSA-9614399 | | Regulation of localization of FOXO transcription factors | | | 2/505 | | 11/10867 | | 9.0E-02 | | 0.51 | | 0.47 | | FOXO1/FOXO3 | | 2 | |
| R-HSA-9634285 | | Constitutive Signaling by Overexpressed ERBB2 | | | 2/505 | | 11/10867 | | 9.0E-02 | | 0.51 | | 0.47 | | ERBIN/HSP90AA1 | | 2 | |
| R-HSA-9683610 | | Maturation of nucleoprotein | | | 2/505 | | 11/10867 | | 9.0E-02 | | 0.51 | | 0.47 | | GSK3B/PARP8 | | 2 | |
| R-HSA-9018519 | | Estrogen-dependent gene expression | | | 11/505 | | 150/10867 | | 9.0E-02 | | 0.51 | | 0.47 | | ERBB4/FOXA1/GTF2F1/HSP90AA1/KPNA2/NRIP1/RUNX1/SP1/TFF1/TFF3/YY1 | | 11 | |
| R-HSA-5685942 | | HDR through Homologous Recombination (HRR) | | | 6/505 | | 67/10867 | | 9.0E-02 | | 0.51 | | 0.47 | | BLM/PALB2/PCNA/POLD4/RAD50/RFC5 | | 6 | |
| R-HSA-5083635 | | Defective B3GALTL causes Peters-plus syndrome (PpS) | | | 4/505 | | 37/10867 | | 9.1E-02 | | 0.51 | | 0.47 | | SEMA5A/THBS1/THSD4/THSD7A | | 4 | |
| R-HSA-5685938 | | HDR through Single Strand Annealing (SSA) | | | 4/505 | | 37/10867 | | 9.1E-02 | | 0.51 | | 0.47 | | BLM/ERCC4/RAD50/RFC5 | | 4 | |
| R-HSA-2428928 | | IRS-related events triggered by IGF1R | | | 5/505 | | 52/10867 | | 9.3E-02 | | 0.52 | | 0.48 | | FGF18/FRS2/IGF1/IGF2/PIK3R1 | | 5 | |
| R-HSA-449836 | | Other interleukin signaling | | | 3/505 | | 24/10867 | | 9.8E-02 | | 0.53 | | 0.48 | | IL16/JAK1/VAMP2 | | 3 | |
| R-HSA-75876 | | Synthesis of very long-chain fatty acyl-CoAs | | | 3/505 | | 24/10867 | | 9.8E-02 | | 0.53 | | 0.48 | | ACSL3/ACSL4/HACD2 | | 3 | |
| R-HSA-9634638 | | Estrogen-dependent nuclear events downstream of ESR-membrane signaling | | | 3/505 | | 24/10867 | | 9.8E-02 | | 0.53 | | 0.48 | | FOXO3/SRF/XPO1 | | 3 | |
| R-HSA-9675136 | | Diseases of DNA Double-Strand Break Repair | | | 3/505 | | 24/10867 | | 9.8E-02 | | 0.53 | | 0.48 | | BLM/PALB2/RAD50 | | 3 | |
| R-HSA-9701193 | | Defective HDR through Homologous Recombination (HRR) due to PALB2 loss of function | | | 3/505 | | 24/10867 | | 9.8E-02 | | 0.53 | | 0.48 | | BLM/PALB2/RAD50 | | 3 | |
| R-HSA-9704331 | | Defective HDR through Homologous Recombination Repair (HRR) due to PALB2 loss of BRCA1 binding function | | | 3/505 | | 24/10867 | | 9.8E-02 | | 0.53 | | 0.48 | | BLM/PALB2/RAD50 | | 3 | |
| R-HSA-9704646 | | Defective HDR through Homologous Recombination Repair (HRR) due to PALB2 loss of BRCA2/RAD51/RAD51C binding function | | | 3/505 | | 24/10867 | | 9.8E-02 | | 0.53 | | 0.48 | | BLM/PALB2/RAD50 | | 3 | |
| R-HSA-2129379 | | Molecules associated with elastic fibres | | | 4/505 | | 38/10867 | | 9.8E-02 | | 0.53 | | 0.48 | | BMP2/BMP4/ITGB5/MFAP3 | | 4 | |
| R-HSA-5173214 | | O-glycosylation of TSR domain-containing proteins | | | 4/505 | | 38/10867 | | 9.8E-02 | | 0.53 | | 0.48 | | SEMA5A/THBS1/THSD4/THSD7A | | 4 | |
| R-HSA-8950505 | | Gene and protein expression by JAK-STAT signaling after Interleukin-12 stimulation | | | 4/505 | | 38/10867 | | 9.8E-02 | | 0.53 | | 0.48 | | CFL1/IL10/MSN/STAT4 | | 4 | |
| R-HSA-2428924 | | IGF1R signaling cascade | | | 5/505 | | 53/10867 | | 9.9E-02 | | 0.53 | | 0.48 | | FGF18/FRS2/IGF1/IGF2/PIK3R1 | | 5 | |
| R-HSA-388841 | | Costimulation by the CD28 family | | | 6/505 | | 69/10867 | | 1.0E-01 | | 0.53 | | 0.49 | | CSK/FYN/HLA-DPB1/LCK/PIK3R1/PTPN6 | | 6 | |
| R-HSA-111932 | | CaMK IV-mediated phosphorylation of CREB | | | 2/505 | | 12/10867 | | 1.0E-01 | | 0.53 | | 0.49 | | CAMK4/KPNA2 | | 2 | |
| R-HSA-1474151 | | Tetrahydrobiopterin (BH4) synthesis. recycling. salvage and regulation | | | 2/505 | | 12/10867 | | 1.0E-01 | | 0.53 | | 0.49 | | HSP90AA1/NOS3 | | 2 | |
| R-HSA-2029481 | | FCGR activation | | | 2/505 | | 12/10867 | | 1.0E-01 | | 0.53 | | 0.49 | | FYN/HCK | | 2 | |
| R-HSA-389359 | | CD28 dependent Vav1 pathway | | | 2/505 | | 12/10867 | | 1.0E-01 | | 0.53 | | 0.49 | | FYN/LCK | | 2 | |
| R-HSA-430116 | | GP1b-IX-V activation signalling | | | 2/505 | | 12/10867 | | 1.0E-01 | | 0.53 | | 0.49 | | COL1A1/PIK3R1 | | 2 | |
| R-HSA-8866427 | | VLDLR internalisation and degradation | | | 2/505 | | 12/10867 | | 1.0E-01 | | 0.53 | | 0.49 | | CLTC/MYLIP | | 2 | |
| R-HSA-8866907 | | Activation of the TFAP2 (AP-2) family of transcription factors | | | 2/505 | | 12/10867 | | 1.0E-01 | | 0.53 | | 0.49 | | CITED2/TFAP2A | | 2 | |
| R-HSA-9020558 | | Interleukin-2 signaling | | | 2/505 | | 12/10867 | | 1.0E-01 | | 0.53 | | 0.49 | | JAK1/LCK | | 2 | |
| R-HSA-2404192 | | Signaling by Type 1 Insulin-like Growth Factor 1 Receptor (IGF1R) | | | 5/505 | | 54/10867 | | 1.0E-01 | | 0.53 | | 0.49 | | FGF18/FRS2/IGF1/IGF2/PIK3R1 | | 5 | |
| R-HSA-140877 | | Formation of Fibrin Clot (Clotting Cascade) | | | 4/505 | | 39/10867 | | 1.1E-01 | | 0.53 | | 0.49 | | F13A1/F3/PROS1/TFPI | | 4 | |
| R-HSA-390471 | | Association of TriC/CCT with target proteins during biosynthesis | | | 4/505 | | 39/10867 | | 1.1E-01 | | 0.53 | | 0.49 | | AP3M1/CCNE2/FBXL5/LONP2 | | 4 | |
| R-HSA-73933 | | Resolution of Abasic Sites (AP sites) | | | 4/505 | | 39/10867 | | 1.1E-01 | | 0.53 | | 0.49 | | PCNA/POLD4/RFC5/XRCC1 | | 4 | |
| R-HSA-76009 | | Platelet Aggregation (Plug Formation) | | | 4/505 | | 39/10867 | | 1.1E-01 | | 0.53 | | 0.49 | | ADRA2A/COL1A1/CSK/TLN1 | | 4 | |
| R-HSA-2029485 | | Role of phospholipids in phagocytosis | | | 3/505 | | 25/10867 | | 1.1E-01 | | 0.53 | | 0.49 | | ITPR1/PIK3R1/PLPP4 | | 3 | |
| R-HSA-418592 | | ADP signalling through P2Y purinoceptor 1 | | | 3/505 | | 25/10867 | | 1.1E-01 | | 0.53 | | 0.49 | | GNAQ/GNG12/PLA2G4A | | 3 | |
| R-HSA-5654708 | | Downstream signaling of activated FGFR3 | | | 3/505 | | 25/10867 | | 1.1E-01 | | 0.53 | | 0.49 | | FGF18/FRS2/PIK3R1 | | 3 | |
| R-HSA-69273 | | Cyclin A/B1/B2 associated events during G2/M transition | | | 3/505 | | 25/10867 | | 1.1E-01 | | 0.53 | | 0.49 | | CCNB2/CCNH/XPO1 | | 3 | |
| R-HSA-9006115 | | Signaling by NTRK2 (TRKB) | | | 3/505 | | 25/10867 | | 1.1E-01 | | 0.53 | | 0.49 | | FRS2/FYN/PIK3R1 | | 3 | |
| R-HSA-9664323 | | FCGR3A-mediated IL10 synthesis | | | 4/505 | | 40/10867 | | 1.1E-01 | | 0.56 | | 0.51 | | FYN/HCK/IL10/ITPR1 | | 4 | |
| R-HSA-446203 | | Asparagine N-linked glycosylation | | | 19/505 | | 304/10867 | | 1.2E-01 | | 0.56 | | 0.52 | | ALG13/AMDHD2/ARCN1/GMPPB/MIA3/RENBP/RNF103/SEC16A/SEC23A/SEC24A/SEC24B/SEC24C/SEC24D/SEL1L/ST3GAL2/ST6GALNAC1/ST6GALNAC3/ST8SIA4/TRAPPC1 | | 19 | |
| R-HSA-5663220 | | RHO GTPases Activate Formins | | | 10/505 | | 140/10867 | | 1.2E-01 | | 0.56 | | 0.52 | | CENPA/CENPQ/CLIP1/DAAM1/FMNL3/RANBP2/RHOB/SGO1/SRF/XPO1 | | 10 | |
| R-HSA-110373 | | Resolution of AP sites via the multiple-nucleotide patch replacement pathway | | | 3/505 | | 26/10867 | | 1.2E-01 | | 0.56 | | 0.52 | | PCNA/POLD4/RFC5 | | 3 | |
| R-HSA-3238698 | | WNT ligand biogenesis and trafficking | | | 3/505 | | 26/10867 | | 1.2E-01 | | 0.56 | | 0.52 | | WLS/WNT5A/WNT7B | | 3 | |
| R-HSA-5693554 | | Resolution of D-loop Structures through Synthesis-Dependent Strand Annealing (SDSA) | | | 3/505 | | 26/10867 | | 1.2E-01 | | 0.56 | | 0.52 | | BLM/PALB2/RAD50 | | 3 | |
| R-HSA-8856825 | | Cargo recognition for clathrin-mediated endocytosis | | | 8/505 | | 106/10867 | | 1.2E-01 | | 0.56 | | 0.52 | | CLTC/COPS6/SCARB2/SGIP1/SH3KBP1/TFRC/VAMP2/WNT5A | | 8 | |
| R-HSA-1362409 | | Mitochondrial iron-sulfur cluster biogenesis | | | 2/505 | | 13/10867 | | 1.2E-01 | | 0.56 | | 0.52 | | FDXR/ISCU | | 2 | |
| R-HSA-5140745 | | WNT5A-dependent internalization of FZD2. FZD5 and ROR2 | | | 2/505 | | 13/10867 | | 1.2E-01 | | 0.56 | | 0.52 | | CLTC/WNT5A | | 2 | |
| R-HSA-5210891 | | Uptake and function of anthrax toxins | | | 2/505 | | 13/10867 | | 1.2E-01 | | 0.56 | | 0.52 | | ANTXR1/ANTXR2 | | 2 | |
| R-HSA-68884 | | Mitotic Telophase/Cytokinesis | | | 2/505 | | 13/10867 | | 1.2E-01 | | 0.56 | | 0.52 | | PDS5B/WAPL | | 2 | |
| R-HSA-8847993 | | ERBB2 Activates PTK6 Signaling | | | 2/505 | | 13/10867 | | 1.2E-01 | | 0.56 | | 0.52 | | ERBB3/ERBB4 | | 2 | |
| R-HSA-5696400 | | Dual Incision in GG-NER | | | 4/505 | | 41/10867 | | 1.2E-01 | | 0.57 | | 0.52 | | ERCC4/PCNA/POLD4/RFC5 | | 4 | |
| R-HSA-5683057 | | MAPK family signaling cascades | | | 20/505 | | 325/10867 | | 1.2E-01 | | 0.57 | | 0.53 | | CSF2RA/CSK/CUL3/DUSP10/DUSP4/ERBB3/ERBB4/FGF18/FOXO1/FOXO3/FRS2/FYN/IL6ST/IQGAP1/JAK1/KITLG/PIK3R1/PSMC3/TLN1/XPO1 | | 20 | |
| R-HSA-170834 | | Signaling by TGF-beta Receptor Complex | | | 6/505 | | 73/10867 | | 1.2E-01 | | 0.57 | | 0.53 | | PMEPA1/RNF111/SP1/TGFBR1/TGFBR2/XPO1 | | 6 | |
| R-HSA-428157 | | Sphingolipid metabolism | | | 7/505 | | 90/10867 | | 1.2E-01 | | 0.58 | | 0.53 | | ASAH1/CERT1/GBA2/PLPP3/PSAP/SGMS2/UGCG | | 7 | |
| R-HSA-5633007 | | Regulation of TP53 Activity | | | 11/505 | | 160/10867 | | 1.3E-01 | | 0.58 | | 0.53 | | BLM/MBD3/NUAK1/PHF20/PRKAA1/PRKAA2/RAD50/RFC5/SUPT16H/TP53INP1/ZNF385A | | 11 | |
| R-HSA-112315 | | Transmission across Chemical Synapses | | | 17/505 | | 270/10867 | | 1.3E-01 | | 0.58 | | 0.53 | | ABAT/ARL6IP5/CACNB4/CAMK4/ERBB4/GNAI2/GNG12/KPNA2/NPTN/PRKAA1/PRKAA2/RAB3A/SLC1A1/SLC38A1/SLC38A2/SLC6A12/VAMP2 | | 17 | |
| R-HSA-195258 | | RHO GTPase Effectors | | | 20/505 | | 327/10867 | | 1.3E-01 | | 0.58 | | 0.53 | | ARPC4/BTK/CENPA/CENPQ/CFL1/CLIP1/CTNNA1/DAAM1/FMNL3/IQGAP1/IQGAP2/KTN1/NCKAP1/RAC2/RANBP2/RHOB/RHOG/SGO1/SRF/XPO1 | | 20 | |
| R-HSA-1538133 | | G0 and Early G1 | | | 3/505 | | 27/10867 | | 1.3E-01 | | 0.58 | | 0.53 | | CCNE2/MYBL2/PCNA | | 3 | |
| R-HSA-201556 | | Signaling by ALK | | | 3/505 | | 27/10867 | | 1.3E-01 | | 0.58 | | 0.53 | | FRS2/PIK3R1/PTPN6 | | 3 | |
| R-HSA-264876 | | Insulin processing | | | 3/505 | | 27/10867 | | 1.3E-01 | | 0.58 | | 0.53 | | ERO1B/PCSK1/VAMP2 | | 3 | |
| R-HSA-5654716 | | Downstream signaling of activated FGFR4 | | | 3/505 | | 27/10867 | | 1.3E-01 | | 0.58 | | 0.53 | | FGF18/FRS2/PIK3R1 | | 3 | |
| R-HSA-397014 | | Muscle contraction | | | 13/505 | | 197/10867 | | 1.3E-01 | | 0.58 | | 0.53 | | ACTG2/ASPH/ATP2A2/CALD1/DMPK/GUCY1A2/ITGB5/ITPR1/KCNK10/PXN/SCN1B/TLN1/VIM | | 13 | |
| R-HSA-5693579 | | Homologous DNA Pairing and Strand Exchange | | | 4/505 | | 42/10867 | | 1.3E-01 | | 0.58 | | 0.53 | | BLM/PALB2/RAD50/RFC5 | | 4 | |
| R-HSA-9035034 | | RHOF GTPase cycle | | | 4/505 | | 42/10867 | | 1.3E-01 | | 0.58 | | 0.53 | | ARHGAP12/ARHGAP32/CAV1/PIK3R1 | | 4 | |
| R-HSA-983168 | | Antigen processing: Ubiquitination & Proteasome degradation | | | 19/505 | | 309/10867 | | 1.3E-01 | | 0.58 | | 0.53 | | CCNF/CUL3/FBXL5/FBXL7/FBXL8/FBXO22/FBXO31/HECTD1/HERC3/KLHL2/MYLIP/NEDD4/PJA2/PSMC3/RNF111/RNF217/TRIM11/TRIM41/UBE4A | | 19 | |
| R-HSA-917937 | | Iron uptake and transport | | | 5/505 | | 58/10867 | | 1.3E-01 | | 0.58 | | 0.53 | | CYBRD1/FBXL5/GLRX3/HFE/TFRC | | 5 | |
| R-HSA-2500257 | | Resolution of Sister Chromatid Cohesion | | | 9/505 | | 126/10867 | | 1.3E-01 | | 0.58 | | 0.53 | | CCNB2/CENPA/CENPQ/CLIP1/PDS5B/RANBP2/SGO1/WAPL/XPO1 | | 9 | |
| R-HSA-9658195 | | Leishmania infection | | | 16/505 | | 254/10867 | | 1.3E-01 | | 0.58 | | 0.53 | | ARPC4/BTK/CALCRL/FYN/GNAI2/GNG12/GPR15/HCK/IL10/ITPR1/NCKAP1/NT5E/P2RY11/RAMP2/TXNIP/WNT5A | | 16 | |
| R-HSA-111885 | | Opioid Signalling | | | 7/505 | | 92/10867 | | 1.4E-01 | | 0.58 | | 0.53 | | CAMK4/GNAI2/GNAQ/GNG12/ITPR1/KPNA2/PLA2G4A | | 7 | |
| R-HSA-198323 | | AKT phosphorylates targets in the cytosol | | | 2/505 | | 14/10867 | | 1.4E-01 | | 0.58 | | 0.53 | | CDKN1A/GSK3B | | 2 | |
| R-HSA-200425 | | Carnitine metabolism | | | 2/505 | | 14/10867 | | 1.4E-01 | | 0.58 | | 0.53 | | ACACA/PRKAA2 | | 2 | |
| R-HSA-2024101 | | CS/DS degradation | | | 2/505 | | 14/10867 | | 1.4E-01 | | 0.58 | | 0.53 | | HYAL1/VCAN | | 2 | |
| R-HSA-209952 | | Peptide hormone biosynthesis | | | 2/505 | | 14/10867 | | 1.4E-01 | | 0.58 | | 0.53 | | INHBA/PCSK1 | | 2 | |
| R-HSA-418885 | | DCC mediated attractive signaling | | | 2/505 | | 14/10867 | | 1.4E-01 | | 0.58 | | 0.53 | | ABLIM3/FYN | | 2 | |
| R-HSA-5358606 | | Mismatch repair (MMR) directed by MSH2:MSH3 (MutSbeta) | | | 2/505 | | 14/10867 | | 1.4E-01 | | 0.58 | | 0.53 | | PCNA/POLD4 | | 2 | |
| R-HSA-6804759 | | Regulation of TP53 Activity through Association with Co-factors | | | 2/505 | | 14/10867 | | 1.4E-01 | | 0.58 | | 0.53 | | PHF20/ZNF385A | | 2 | |
| R-HSA-69166 | | Removal of the Flap Intermediate | | | 2/505 | | 14/10867 | | 1.4E-01 | | 0.58 | | 0.53 | | PCNA/POLD4 | | 2 | |
| R-HSA-8983432 | | Interleukin-15 signaling | | | 2/505 | | 14/10867 | | 1.4E-01 | | 0.58 | | 0.53 | | IL15RA/JAK1 | | 2 | |
| R-HSA-5655253 | | Signaling by FGFR2 in disease | | | 4/505 | | 43/10867 | | 1.4E-01 | | 0.58 | | 0.53 | | FGF18/FRS2/GTF2F1/PIK3R1 | | 4 | |
| R-HSA-9013406 | | RHOQ GTPase cycle | | | 5/505 | | 59/10867 | | 1.4E-01 | | 0.58 | | 0.53 | | ARHGAP32/CAV1/GIT2/IQGAP1/TFRC | | 5 | |
| R-HSA-9664407 | | Parasite infection | | | 5/505 | | 59/10867 | | 1.4E-01 | | 0.58 | | 0.53 | | ARPC4/BTK/FYN/HCK/NCKAP1 | | 5 | |
| R-HSA-9664417 | | Leishmania phagocytosis | | | 5/505 | | 59/10867 | | 1.4E-01 | | 0.58 | | 0.53 | | ARPC4/BTK/FYN/HCK/NCKAP1 | | 5 | |
| R-HSA-9664422 | | FCGR3A-mediated phagocytosis | | | 5/505 | | 59/10867 | | 1.4E-01 | | 0.58 | | 0.53 | | ARPC4/BTK/FYN/HCK/NCKAP1 | | 5 | |
| R-HSA-400042 | | Adrenaline.noradrenaline inhibits insulin secretion | | | 3/505 | | 28/10867 | | 1.4E-01 | | 0.58 | | 0.54 | | ADRA2A/GNAI2/GNG12 | | 3 | |
| R-HSA-3000178 | | ECM proteoglycans | | | 6/505 | | 76/10867 | | 1.4E-01 | | 0.59 | | 0.54 | | COL1A1/COL5A1/ITGA2/ITGB5/SPARC/VCAN | | 6 | |
| R-HSA-8856828 | | Clathrin-mediated endocytosis | | | 10/505 | | 146/10867 | | 1.4E-01 | | 0.59 | | 0.54 | | ARPC4/CLTC/COPS6/RAB5B/SCARB2/SGIP1/SH3KBP1/TFRC/VAMP2/WNT5A | | 10 | |
| R-HSA-5173105 | | O-linked glycosylation | | | 8/505 | | 111/10867 | | 1.4E-01 | | 0.60 | | 0.55 | | GALNT1/GALNT7/SEMA5A/ST3GAL2/ST6GALNAC3/THBS1/THSD4/THSD7A | | 8 | |
| R-HSA-380972 | | Energy dependent regulation of mTOR by LKB1-AMPK | | | 3/505 | | 29/10867 | | 1.5E-01 | | 0.61 | | 0.56 | | CAB39/PRKAA1/PRKAA2 | | 3 | |
| R-HSA-9615017 | | FOXO-mediated transcription of oxidative stress. metabolic and neuronal genes | | | 3/505 | | 29/10867 | | 1.5E-01 | | 0.61 | | 0.56 | | ABCA6/FOXO1/FOXO3 | | 3 | |
| R-HSA-2162123 | | Synthesis of Prostaglandins (PG) and Thromboxanes (TX) | | | 2/505 | | 15/10867 | | 1.5E-01 | | 0.61 | | 0.56 | | HPGD/PTGES2 | | 2 | |
| R-HSA-5099900 | | WNT5A-dependent internalization of FZD4 | | | 2/505 | | 15/10867 | | 1.5E-01 | | 0.61 | | 0.56 | | CLTC/WNT5A | | 2 | |
| R-HSA-5637810 | | Constitutive Signaling by EGFRvIII | | | 2/505 | | 15/10867 | | 1.5E-01 | | 0.61 | | 0.56 | | HSP90AA1/PIK3R1 | | 2 | |
| R-HSA-5637812 | | Signaling by EGFRvIII in Cancer | | | 2/505 | | 15/10867 | | 1.5E-01 | | 0.61 | | 0.56 | | HSP90AA1/PIK3R1 | | 2 | |
| R-HSA-6785631 | | ERBB2 Regulates Cell Motility | | | 2/505 | | 15/10867 | | 1.5E-01 | | 0.61 | | 0.56 | | ERBB3/ERBB4 | | 2 | |
| R-HSA-69183 | | Processive synthesis on the lagging strand | | | 2/505 | | 15/10867 | | 1.5E-01 | | 0.61 | | 0.56 | | PCNA/POLD4 | | 2 | |
| R-HSA-8866910 | | TFAP2 (AP-2) family regulates transcription of growth factors and their receptors | | | 2/505 | | 15/10867 | | 1.5E-01 | | 0.61 | | 0.56 | | TFAP2A/YY1 | | 2 | |
| R-HSA-198725 | | Nuclear Events (kinase and transcription factor activation) | | | 5/505 | | 61/10867 | | 1.5E-01 | | 0.61 | | 0.56 | | DUSP4/F3/MEF2C/SRF/TCF12 | | 5 | |
| R-HSA-2029482 | | Regulation of actin dynamics for phagocytic cup formation | | | 5/505 | | 61/10867 | | 1.5E-01 | | 0.61 | | 0.56 | | ARPC4/BTK/CFL1/HSP90AA1/NCKAP1 | | 5 | |
| R-HSA-68877 | | Mitotic Prometaphase | | | 13/505 | | 204/10867 | | 1.5E-01 | | 0.62 | | 0.57 | | CCNB2/CCP110/CENPA/CENPQ/CLIP1/HSP90AA1/PDS5B/RANBP2/SGO1/SMC4/TUBGCP6/WAPL/XPO1 | | 13 | |
| R-HSA-1566948 | | Elastic fibre formation | | | 4/505 | | 45/10867 | | 1.6E-01 | | 0.62 | | 0.57 | | BMP2/BMP4/ITGB5/MFAP3 | | 4 | |
| R-HSA-6806834 | | Signaling by MET | | | 6/505 | | 79/10867 | | 1.6E-01 | | 0.63 | | 0.58 | | COL1A1/COL5A1/ITGA2/LRIG1/PIK3R1/SH3KBP1 | | 6 | |
| R-HSA-110314 | | Recognition of DNA damage by PCNA-containing replication complex | | | 3/505 | | 30/10867 | | 1.6E-01 | | 0.63 | | 0.58 | | PCNA/POLD4/RFC5 | | 3 | |
| R-HSA-5654696 | | Downstream signaling of activated FGFR2 | | | 3/505 | | 30/10867 | | 1.6E-01 | | 0.63 | | 0.58 | | FGF18/FRS2/PIK3R1 | | 3 | |
| R-HSA-8874081 | | MET activates PTK2 signaling | | | 3/505 | | 30/10867 | | 1.6E-01 | | 0.63 | | 0.58 | | COL1A1/COL5A1/ITGA2 | | 3 | |
| R-HSA-1660662 | | Glycosphingolipid metabolism | | | 4/505 | | 46/10867 | | 1.6E-01 | | 0.63 | | 0.58 | | ASAH1/GBA2/PSAP/UGCG | | 4 | |
| R-HSA-167169 | | HIV Transcription Elongation | | | 4/505 | | 46/10867 | | 1.6E-01 | | 0.63 | | 0.58 | | CCNH/GTF2F1/NELFB/SUPT16H | | 4 | |
| R-HSA-167200 | | Formation of HIV-1 elongation complex containing HIV-1 Tat | | | 4/505 | | 46/10867 | | 1.6E-01 | | 0.63 | | 0.58 | | CCNH/GTF2F1/NELFB/SUPT16H | | 4 | |
| R-HSA-167246 | | Tat-mediated elongation of the HIV-1 transcript | | | 4/505 | | 46/10867 | | 1.6E-01 | | 0.63 | | 0.58 | | CCNH/GTF2F1/NELFB/SUPT16H | | 4 | |
| R-HSA-8854214 | | TBC/RABGAPs | | | 4/505 | | 46/10867 | | 1.6E-01 | | 0.63 | | 0.58 | | RAB11A/RAB5B/RAB8B/TBC1D10C | | 4 | |
| R-HSA-187037 | | Signaling by NTRK1 (TRKA) | | | 8/505 | | 115/10867 | | 1.7E-01 | | 0.63 | | 0.58 | | CLTC/DUSP4/F3/FRS2/MEF2C/PIK3R1/SRF/TCF12 | | 8 | |
| R-HSA-416482 | | G alpha (12/13) signalling events | | | 6/505 | | 80/10867 | | 1.7E-01 | | 0.63 | | 0.58 | | ARHGEF12/ARHGEF3/BTK/FGD2/GNG12/RHOB | | 6 | |
| R-HSA-110312 | | Translesion synthesis by REV1 | | | 2/505 | | 16/10867 | | 1.7E-01 | | 0.63 | | 0.58 | | PCNA/RFC5 | | 2 | |
| R-HSA-156711 | | Polo-like kinase mediated events | | | 2/505 | | 16/10867 | | 1.7E-01 | | 0.63 | | 0.58 | | CCNB2/MYBL2 | | 2 | |
| R-HSA-2173791 | | TGF-beta receptor signaling in EMT (epithelial to mesenchymal transition) | | | 2/505 | | 16/10867 | | 1.7E-01 | | 0.63 | | 0.58 | | TGFBR1/TGFBR2 | | 2 | |
| R-HSA-2730905 | | Role of LAT2/NTAL/LAB on calcium mobilization | | | 2/505 | | 16/10867 | | 1.7E-01 | | 0.63 | | 0.58 | | FYN/PIK3R1 | | 2 | |
| R-HSA-418457 | | cGMP effects | | | 2/505 | | 16/10867 | | 1.7E-01 | | 0.63 | | 0.58 | | ITPR1/PRKG1 | | 2 | |
| R-HSA-9703648 | | Signaling by FLT3 ITD and TKD mutants | | | 2/505 | | 16/10867 | | 1.7E-01 | | 0.63 | | 0.58 | | CDKN1A/PIK3R1 | | 2 | |
| R-HSA-975163 | | IRAK2 mediated activation of TAK1 complex upon TLR7/8 or 9 stimulation | | | 2/505 | | 16/10867 | | 1.7E-01 | | 0.63 | | 0.58 | | TAB1/TLR4 | | 2 | |
| R-HSA-69231 | | Cyclin D associated events in G1 | | | 4/505 | | 47/10867 | | 1.7E-01 | | 0.65 | | 0.60 | | CCND2/CCNE2/CCNH/CDKN1A | | 4 | |
| R-HSA-69236 | | G1 Phase | | | 4/505 | | 47/10867 | | 1.7E-01 | | 0.65 | | 0.60 | | CCND2/CCNE2/CCNH/CDKN1A | | 4 | |
| R-HSA-9679191 | | Potential therapeutics for SARS | | | 6/505 | | 81/10867 | | 1.7E-01 | | 0.65 | | 0.60 | | HSP90AA1/IFNGR1/IL1R1/JAK1/MBD3/MTA3 | | 6 | |
| R-HSA-1442490 | | Collagen degradation | | | 5/505 | | 64/10867 | | 1.8E-01 | | 0.65 | | 0.60 | | ADAM10/COL17A1/COL1A1/COL5A1/PHYKPL | | 5 | |
| R-HSA-421270 | | Cell-cell junction organization | | | 5/505 | | 64/10867 | | 1.8E-01 | | 0.65 | | 0.60 | | CDH11/CDH17/CDH6/CTNNA1/CTNND1 | | 5 | |
| R-HSA-166016 | | Toll Like Receptor 4 (TLR4) Cascade | | | 9/505 | | 135/10867 | | 1.8E-01 | | 0.65 | | 0.60 | | BTK/DUSP4/IRF7/ITGB2/MAP3K1/MEF2C/RIPK3/TAB1/TLR4 | | 9 | |
| R-HSA-5684996 | | MAPK1/MAPK3 signaling | | | 17/505 | | 286/10867 | | 1.8E-01 | | 0.65 | | 0.60 | | CSF2RA/CSK/CUL3/DUSP10/DUSP4/ERBB3/ERBB4/FGF18/FRS2/FYN/IL6ST/IQGAP1/JAK1/KITLG/PIK3R1/PSMC3/TLN1 | | 17 | |
| R-HSA-167152 | | Formation of HIV elongation complex in the absence of HIV Tat | | | 4/505 | | 48/10867 | | 1.8E-01 | | 0.65 | | 0.60 | | CCNH/GTF2F1/NELFB/SUPT16H | | 4 | |
| R-HSA-418597 | | G alpha (z) signalling events | | | 4/505 | | 48/10867 | | 1.8E-01 | | 0.65 | | 0.60 | | ADRA2A/GNAI2/GNG12/RGS4 | | 4 | |
| R-HSA-3700989 | | Transcriptional Regulation by TP53 | | | 21/505 | | 365/10867 | | 1.8E-01 | | 0.65 | | 0.60 | | BLM/BTG2/CCNE2/CCNH/CDKN1A/COX7C/FANCI/GTF2F1/MBD3/NELFB/NUAK1/PCNA/PERP/PHF20/PRKAA1/PRKAA2/RAD50/RFC5/SUPT16H/TP53INP1/ZNF385A | | 21 | |
| R-HSA-6782210 | | Gap-filling DNA repair synthesis and ligation in TC-NER | | | 5/505 | | 65/10867 | | 1.8E-01 | | 0.65 | | 0.60 | | CCNH/PCNA/POLD4/RFC5/XRCC1 | | 5 | |
| R-HSA-6796648 | | TP53 Regulates Transcription of DNA Repair Genes | | | 5/505 | | 65/10867 | | 1.8E-01 | | 0.65 | | 0.60 | | CCNH/FANCI/GTF2F1/NELFB/SUPT16H | | 5 | |
| R-HSA-5656169 | | Termination of translesion DNA synthesis | | | 3/505 | | 32/10867 | | 1.8E-01 | | 0.65 | | 0.60 | | PCNA/POLD4/RFC5 | | 3 | |
| R-HSA-69190 | | DNA strand elongation | | | 3/505 | | 32/10867 | | 1.8E-01 | | 0.65 | | 0.60 | | PCNA/POLD4/RFC5 | | 3 | |
| R-HSA-1482922 | | Acyl chain remodelling of PI | | | 2/505 | | 17/10867 | | 1.9E-01 | | 0.65 | | 0.60 | | PLA2G2A/PLA2G4A | | 2 | |
| R-HSA-1810476 | | RIP-mediated NFkB activation via ZBP1 | | | 2/505 | | 17/10867 | | 1.9E-01 | | 0.65 | | 0.60 | | DHX9/RIPK3 | | 2 | |
| R-HSA-199220 | | Vitamin B5 (pantothenate) metabolism | | | 2/505 | | 17/10867 | | 1.9E-01 | | 0.65 | | 0.60 | | ENPP2/SLC5A6 | | 2 | |
| R-HSA-264642 | | Acetylcholine Neurotransmitter Release Cycle | | | 2/505 | | 17/10867 | | 1.9E-01 | | 0.65 | | 0.60 | | RAB3A/VAMP2 | | 2 | |
| R-HSA-432142 | | Platelet sensitization by LDL | | | 2/505 | | 17/10867 | | 1.9E-01 | | 0.65 | | 0.60 | | PLA2G4A/PTPN6 | | 2 | |
| R-HSA-5602498 | | MyD88 deficiency (TLR2/4) | | | 2/505 | | 17/10867 | | 1.9E-01 | | 0.65 | | 0.60 | | BTK/TLR4 | | 2 | |
| R-HSA-5655862 | | Translesion synthesis by POLK | | | 2/505 | | 17/10867 | | 1.9E-01 | | 0.65 | | 0.60 | | PCNA/RFC5 | | 2 | |
| R-HSA-5656121 | | Translesion synthesis by POLI | | | 2/505 | | 17/10867 | | 1.9E-01 | | 0.65 | | 0.60 | | PCNA/RFC5 | | 2 | |
| R-HSA-6788467 | | IL-6-type cytokine receptor ligand interactions | | | 2/505 | | 17/10867 | | 1.9E-01 | | 0.65 | | 0.60 | | IL6ST/JAK1 | | 2 | |
| R-HSA-8934593 | | Regulation of RUNX1 Expression and Activity | | | 2/505 | | 17/10867 | | 1.9E-01 | | 0.65 | | 0.60 | | CCND2/RUNX1 | | 2 | |
| R-HSA-9034015 | | Signaling by NTRK3 (TRKC) | | | 2/505 | | 17/10867 | | 1.9E-01 | | 0.65 | | 0.60 | | NELFB/PIK3R1 | | 2 | |
| R-HSA-937072 | | TRAF6-mediated induction of TAK1 complex within TLR4 complex | | | 2/505 | | 17/10867 | | 1.9E-01 | | 0.65 | | 0.60 | | TAB1/TLR4 | | 2 | |
| R-HSA-6782135 | | Dual incision in TC-NER | | | 5/505 | | 66/10867 | | 1.9E-01 | | 0.66 | | 0.61 | | CCNH/ERCC4/PCNA/POLD4/RFC5 | | 5 | |
| R-HSA-69563 | | p53-Dependent G1 DNA Damage Response | | | 5/505 | | 66/10867 | | 1.9E-01 | | 0.66 | | 0.61 | | CCNE2/CDKN1A/PHF20/PSMC3/ZNF385A | | 5 | |
| R-HSA-69580 | | p53-Dependent G1/S DNA damage checkpoint | | | 5/505 | | 66/10867 | | 1.9E-01 | | 0.66 | | 0.61 | | CCNE2/CDKN1A/PHF20/PSMC3/ZNF385A | | 5 | |
| R-HSA-5693538 | | Homology Directed Repair | | | 9/505 | | 138/10867 | | 1.9E-01 | | 0.66 | | 0.61 | | BLM/ERCC4/PALB2/PCNA/POLD4/PPP4R2/RAD50/RFC5/XRCC1 | | 9 | |
| R-HSA-674695 | | RNA Polymerase II Pre-transcription Events | | | 6/505 | | 84/10867 | | 2.0E-01 | | 0.66 | | 0.61 | | AFF4/CCNH/EAF1/GTF2F1/NELFB/SUPT16H | | 6 | |
| R-HSA-113418 | | Formation of the Early Elongation Complex | | | 3/505 | | 33/10867 | | 2.0E-01 | | 0.66 | | 0.61 | | CCNH/GTF2F1/NELFB | | 3 | |
| R-HSA-167158 | | Formation of the HIV-1 Early Elongation Complex | | | 3/505 | | 33/10867 | | 2.0E-01 | | 0.66 | | 0.61 | | CCNH/GTF2F1/NELFB | | 3 | |
| R-HSA-352230 | | Amino acid transport across the plasma membrane | | | 3/505 | | 33/10867 | | 2.0E-01 | | 0.66 | | 0.61 | | SLC38A1/SLC38A2/SLC6A12 | | 3 | |
| R-HSA-389356 | | CD28 co-stimulation | | | 3/505 | | 33/10867 | | 2.0E-01 | | 0.66 | | 0.61 | | FYN/LCK/PIK3R1 | | 3 | |
| R-HSA-5693568 | | Resolution of D-loop Structures through Holliday Junction Intermediates | | | 3/505 | | 33/10867 | | 2.0E-01 | | 0.66 | | 0.61 | | BLM/PALB2/RAD50 | | 3 | |
| R-HSA-8964043 | | Plasma lipoprotein clearance | | | 3/505 | | 33/10867 | | 2.0E-01 | | 0.66 | | 0.61 | | CLTC/MYLIP/NCEH1 | | 3 | |
| R-HSA-1793185 | | Chondroitin sulfate/dermatan sulfate metabolism | | | 4/505 | | 50/10867 | | 2.0E-01 | | 0.66 | | 0.61 | | CHST15/CHST7/HYAL1/VCAN | | 4 | |
| R-HSA-5632684 | | Hedgehog 'on' state | | | 6/505 | | 85/10867 | | 2.0E-01 | | 0.66 | | 0.61 | | BOC/CSNK1A1/CUL3/NUMB/PSMC3/SPOPL | | 6 | |
| R-HSA-1369062 | | ABC transporters in lipid homeostasis | | | 2/505 | | 18/10867 | | 2.0E-01 | | 0.66 | | 0.61 | | ABCA6/PEX19 | | 2 | |
| R-HSA-181429 | | Serotonin Neurotransmitter Release Cycle | | | 2/505 | | 18/10867 | | 2.0E-01 | | 0.66 | | 0.61 | | RAB3A/VAMP2 | | 2 | |
| R-HSA-181430 | | Norepinephrine Neurotransmitter Release Cycle | | | 2/505 | | 18/10867 | | 2.0E-01 | | 0.66 | | 0.61 | | RAB3A/VAMP2 | | 2 | |
| R-HSA-1912420 | | Pre-NOTCH Processing in Golgi | | | 2/505 | | 18/10867 | | 2.0E-01 | | 0.66 | | 0.61 | | ATP2A2/SEL1L | | 2 | |
| R-HSA-446353 | | Cell-extracellular matrix interactions | | | 2/505 | | 18/10867 | | 2.0E-01 | | 0.66 | | 0.61 | | FERMT2/PXN | | 2 | |
| R-HSA-5603041 | | IRAK4 deficiency (TLR2/4) | | | 2/505 | | 18/10867 | | 2.0E-01 | | 0.66 | | 0.61 | | BTK/TLR4 | | 2 | |
| R-HSA-5635838 | | Activation of SMO | | | 2/505 | | 18/10867 | | 2.0E-01 | | 0.66 | | 0.61 | | BOC/CSNK1A1 | | 2 | |
| R-HSA-6804114 | | TP53 Regulates Transcription of Genes Involved in G2 Cell Cycle Arrest | | | 2/505 | | 18/10867 | | 2.0E-01 | | 0.66 | | 0.61 | | PCNA/ZNF385A | | 2 | |
| R-HSA-8964315 | | G beta:gamma signalling through BTK | | | 2/505 | | 18/10867 | | 2.0E-01 | | 0.66 | | 0.61 | | BTK/GNG12 | | 2 | |
| R-HSA-936964 | | Activation of IRF3/IRF7 mediated by TBK1/IKK epsilon | | | 2/505 | | 18/10867 | | 2.0E-01 | | 0.66 | | 0.61 | | IRF7/TLR4 | | 2 | |
| R-HSA-69615 | | G1/S DNA Damage Checkpoints | | | 5/505 | | 68/10867 | | 2.1E-01 | | 0.68 | | 0.62 | | CCNE2/CDKN1A/PHF20/PSMC3/ZNF385A | | 5 | |
| R-HSA-167238 | | Pausing and recovery of Tat-mediated HIV elongation | | | 3/505 | | 34/10867 | | 2.1E-01 | | 0.68 | | 0.62 | | GTF2F1/NELFB/SUPT16H | | 3 | |
| R-HSA-167243 | | Tat-mediated HIV elongation arrest and recovery | | | 3/505 | | 34/10867 | | 2.1E-01 | | 0.68 | | 0.62 | | GTF2F1/NELFB/SUPT16H | | 3 | |
| R-HSA-5693537 | | Resolution of D-Loop Structures | | | 3/505 | | 34/10867 | | 2.1E-01 | | 0.68 | | 0.62 | | BLM/PALB2/RAD50 | | 3 | |
| R-HSA-5620920 | | Cargo trafficking to the periciliary membrane | | | 4/505 | | 51/10867 | | 2.1E-01 | | 0.68 | | 0.63 | | INPP5E/RAB11A/RAB3IP/RP2 | | 4 | |
| R-HSA-110320 | | Translesion Synthesis by POLH | | | 2/505 | | 19/10867 | | 2.2E-01 | | 0.69 | | 0.64 | | PCNA/RFC5 | | 2 | |
| R-HSA-1236382 | | Constitutive Signaling by Ligand-Responsive EGFR Cancer Variants | | | 2/505 | | 19/10867 | | 2.2E-01 | | 0.69 | | 0.64 | | HSP90AA1/PIK3R1 | | 2 | |
| R-HSA-1482925 | | Acyl chain remodelling of PG | | | 2/505 | | 19/10867 | | 2.2E-01 | | 0.69 | | 0.64 | | PLA2G2A/PLA2G4A | | 2 | |
| R-HSA-202430 | | Translocation of ZAP-70 to Immunological synapse | | | 2/505 | | 19/10867 | | 2.2E-01 | | 0.69 | | 0.64 | | HLA-DPB1/LCK | | 2 | |
| R-HSA-2995383 | | Initiation of Nuclear Envelope (NE) Reformation | | | 2/505 | | 19/10867 | | 2.2E-01 | | 0.69 | | 0.64 | | CCNB2/VRK1 | | 2 | |
| R-HSA-422085 | | Synthesis. secretion. and deacylation of Ghrelin | | | 2/505 | | 19/10867 | | 2.2E-01 | | 0.69 | | 0.64 | | IGF1/PCSK1 | | 2 | |
| R-HSA-5637815 | | Signaling by Ligand-Responsive EGFR Variants in Cancer | | | 2/505 | | 19/10867 | | 2.2E-01 | | 0.69 | | 0.64 | | HSP90AA1/PIK3R1 | | 2 | |
| R-HSA-8963889 | | Assembly of active LPL and LIPC lipase complexes | | | 2/505 | | 19/10867 | | 2.2E-01 | | 0.69 | | 0.64 | | CIDEC/PCSK5 | | 2 | |
| R-HSA-8964038 | | LDL clearance | | | 2/505 | | 19/10867 | | 2.2E-01 | | 0.69 | | 0.64 | | CLTC/NCEH1 | | 2 | |
| R-HSA-9703465 | | Signaling by FLT3 fusion proteins | | | 2/505 | | 19/10867 | | 2.2E-01 | | 0.69 | | 0.64 | | CDKN1A/PIK3R1 | | 2 | |
| R-HSA-432720 | | Lysosome Vesicle Biogenesis | | | 3/505 | | 35/10867 | | 2.2E-01 | | 0.69 | | 0.64 | | AP1S1/CLTC/VAMP2 | | 3 | |
| R-HSA-75153 | | Apoptotic execution phase | | | 4/505 | | 52/10867 | | 2.2E-01 | | 0.69 | | 0.64 | | APC/KPNA1/TJP1/VIM | | 4 | |
| R-HSA-69242 | | S Phase | | | 10/505 | | 162/10867 | | 2.2E-01 | | 0.69 | | 0.64 | | CCNE2/CCNH/CDKN1A/GSK3B/PCNA/PDS5B/POLD4/PSMC3/RFC5/WAPL | | 10 | |
| R-HSA-174824 | | Plasma lipoprotein assembly. remodeling. and clearance | | | 5/505 | | 70/10867 | | 2.3E-01 | | 0.70 | | 0.65 | | CIDEC/CLTC/MYLIP/NCEH1/PCSK5 | | 5 | |
| R-HSA-400253 | | Circadian Clock | | | 5/505 | | 70/10867 | | 2.3E-01 | | 0.70 | | 0.65 | | BHLHE40/MEF2C/NRIP1/RBM4/SIRT1 | | 5 | |
| R-HSA-5673001 | | RAF/MAP kinase cascade | | | 16/505 | | 280/10867 | | 2.3E-01 | | 0.71 | | 0.66 | | CSF2RA/CSK/CUL3/DUSP10/DUSP4/ERBB3/ERBB4/FGF18/FRS2/FYN/IQGAP1/JAK1/KITLG/PIK3R1/PSMC3/TLN1 | | 16 | |
| R-HSA-1222556 | | ROS and RNS production in phagocytes | | | 3/505 | | 36/10867 | | 2.3E-01 | | 0.71 | | 0.66 | | HVCN1/NOS3/RAC2 | | 3 | |
| R-HSA-167287 | | HIV elongation arrest and recovery | | | 3/505 | | 36/10867 | | 2.3E-01 | | 0.71 | | 0.66 | | GTF2F1/NELFB/SUPT16H | | 3 | |
| R-HSA-167290 | | Pausing and recovery of HIV elongation | | | 3/505 | | 36/10867 | | 2.3E-01 | | 0.71 | | 0.66 | | GTF2F1/NELFB/SUPT16H | | 3 | |
| R-HSA-5663213 | | RHO GTPases Activate WASPs and WAVEs | | | 3/505 | | 36/10867 | | 2.3E-01 | | 0.71 | | 0.66 | | ARPC4/BTK/NCKAP1 | | 3 | |
| R-HSA-6802948 | | Signaling by high-kinase activity BRAF mutants | | | 3/505 | | 36/10867 | | 2.3E-01 | | 0.71 | | 0.66 | | CSK/IQGAP1/TLN1 | | 3 | |
| R-HSA-9687139 | | Aberrant regulation of mitotic cell cycle due to RB1 defects | | | 3/505 | | 36/10867 | | 2.3E-01 | | 0.71 | | 0.66 | | CCND2/CCNE2/CDKN1A | | 3 | |
| R-HSA-2028269 | | Signaling by Hippo | | | 2/505 | | 20/10867 | | 2.4E-01 | | 0.72 | | 0.66 | | LATS2/TJP1 | | 2 | |
| R-HSA-5627117 | | RHO GTPases Activate ROCKs | | | 2/505 | | 20/10867 | | 2.4E-01 | | 0.72 | | 0.66 | | CFL1/RHOB | | 2 | |
| R-HSA-5654706 | | FRS-mediated FGFR3 signaling | | | 2/505 | | 20/10867 | | 2.4E-01 | | 0.72 | | 0.66 | | FGF18/FRS2 | | 2 | |
| R-HSA-8876384 | | Listeria monocytogenes entry into host cells | | | 2/505 | | 20/10867 | | 2.4E-01 | | 0.72 | | 0.66 | | CTNND1/SH3KBP1 | | 2 | |
| R-HSA-9013405 | | RHOD GTPase cycle | | | 4/505 | | 54/10867 | | 2.4E-01 | | 0.73 | | 0.67 | | ARHGAP12/ARHGAP32/CAV1/PIK3R1 | | 4 | |
| R-HSA-1169408 | | ISG15 antiviral mechanism | | | 5/505 | | 72/10867 | | 2.4E-01 | | 0.73 | | 0.67 | | JAK1/KPNA1/KPNA2/NEDD4/RANBP2 | | 5 | |
| R-HSA-1445148 | | Translocation of SLC2A4 (GLUT4) to the plasma membrane | | | 5/505 | | 72/10867 | | 2.4E-01 | | 0.73 | | 0.67 | | KIF3B/PRKAA2/RAB11A/RAB14/VAMP2 | | 5 | |
| R-HSA-390466 | | Chaperonin-mediated protein folding | | | 6/505 | | 91/10867 | | 2.5E-01 | | 0.74 | | 0.68 | | AP3M1/CCNE2/FBXL5/GNAQ/GNG12/LONP2 | | 6 | |
| R-HSA-877300 | | Interferon gamma signaling | | | 6/505 | | 91/10867 | | 2.5E-01 | | 0.74 | | 0.68 | | HLA-DPB1/IFNGR1/IRF7/JAK1/PTPN6/VCAM1 | | 6 | |
| R-HSA-5693532 | | DNA Double-Strand Break Repair | | | 10/505 | | 167/10867 | | 2.5E-01 | | 0.74 | | 0.69 | | BLM/ERCC4/KPNA2/PALB2/PCNA/POLD4/PPP4R2/RAD50/RFC5/XRCC1 | | 10 | |
| R-HSA-1606322 | | ZBP1(DAI) mediated induction of type I IFNs | | | 2/505 | | 21/10867 | | 2.6E-01 | | 0.75 | | 0.69 | | DHX9/RIPK3 | | 2 | |
| R-HSA-164938 | | Nef-mediates down modulation of cell surface receptors by recruiting them to clathrin adapters | | | 2/505 | | 21/10867 | | 2.6E-01 | | 0.75 | | 0.69 | | AP1S1/LCK | | 2 | |
| R-HSA-389513 | | CTLA4 inhibitory signaling | | | 2/505 | | 21/10867 | | 2.6E-01 | | 0.75 | | 0.69 | | FYN/LCK | | 2 | |
| R-HSA-416572 | | Sema4D induced cell migration and growth-cone collapse | | | 2/505 | | 21/10867 | | 2.6E-01 | | 0.75 | | 0.69 | | ARHGEF12/RHOB | | 2 | |
| R-HSA-5620916 | | VxPx cargo-targeting to cilium | | | 2/505 | | 21/10867 | | 2.6E-01 | | 0.75 | | 0.69 | | RAB11A/RAB3IP | | 2 | |
| R-HSA-5654689 | | PI-3K cascade:FGFR1 | | | 2/505 | | 21/10867 | | 2.6E-01 | | 0.75 | | 0.69 | | FRS2/PIK3R1 | | 2 | |
| R-HSA-6807004 | | Negative regulation of MET activity | | | 2/505 | | 21/10867 | | 2.6E-01 | | 0.75 | | 0.69 | | LRIG1/SH3KBP1 | | 2 | |
| R-HSA-901032 | | ER Quality Control Compartment (ERQC) | | | 2/505 | | 21/10867 | | 2.6E-01 | | 0.75 | | 0.69 | | RNF103/SEL1L | | 2 | |
| R-HSA-975155 | | MyD88 dependent cascade initiated on endosome | | | 6/505 | | 92/10867 | | 2.6E-01 | | 0.75 | | 0.69 | | DUSP4/IRF7/MAP3K1/MEF2C/TAB1/TLR4 | | 6 | |
| R-HSA-212165 | | Epigenetic regulation of gene expression | | | 9/505 | | 149/10867 | | 2.6E-01 | | 0.75 | | 0.69 | | BAZ2A/CBX3/CCNH/DNMT1/GSK3B/MBD3/MTA3/PHF19/SIRT1 | | 9 | |
| R-HSA-453279 | | Mitotic G1 phase and G1/S transition | | | 9/505 | | 149/10867 | | 2.6E-01 | | 0.75 | | 0.69 | | CCND2/CCNE2/CCNH/CDKN1A/GMNN/MYBL2/PCNA/PSMC3/RRM2 | | 9 | |
| R-HSA-162909 | | Host Interactions of HIV factors | | | 8/505 | | 130/10867 | | 2.6E-01 | | 0.75 | | 0.69 | | AP1S1/FYN/HCK/KPNA1/LCK/PSMC3/RANBP2/XPO1 | | 8 | |
| R-HSA-111465 | | Apoptotic cleavage of cellular proteins | | | 3/505 | | 38/10867 | | 2.6E-01 | | 0.75 | | 0.69 | | APC/TJP1/VIM | | 3 | |
| R-HSA-9675126 | | Diseases of mitotic cell cycle | | | 3/505 | | 38/10867 | | 2.6E-01 | | 0.75 | | 0.69 | | CCND2/CCNE2/CDKN1A | | 3 | |
| R-HSA-9662851 | | Anti-inflammatory response favouring Leishmania parasite infection | | | 10/505 | | 169/10867 | | 2.6E-01 | | 0.75 | | 0.69 | | CALCRL/FYN/GNAI2/GNG12/GPR15/HCK/IL10/ITPR1/P2RY11/RAMP2 | | 10 | |
| R-HSA-9664433 | | Leishmania parasite growth and survival | | | 10/505 | | 169/10867 | | 2.6E-01 | | 0.75 | | 0.69 | | CALCRL/FYN/GNAI2/GNG12/GPR15/HCK/IL10/ITPR1/P2RY11/RAMP2 | | 10 | |
| R-HSA-5578775 | | Ion homeostasis | | | 4/505 | | 56/10867 | | 2.6E-01 | | 0.75 | | 0.69 | | ASPH/ATP2A2/DMPK/ITPR1 | | 4 | |
| R-HSA-168181 | | Toll Like Receptor 7/8 (TLR7/8) Cascade | | | 6/505 | | 93/10867 | | 2.6E-01 | | 0.75 | | 0.70 | | DUSP4/IRF7/MAP3K1/MEF2C/TAB1/TLR4 | | 6 | |
| R-HSA-983705 | | Signaling by the B Cell Receptor (BCR) | | | 7/505 | | 112/10867 | | 2.6E-01 | | 0.76 | | 0.70 | | BTK/FYN/ITPR1/PIK3R1/PSMC3/PTPN6/SH3KBP1 | | 7 | |
| R-HSA-196854 | | Metabolism of vitamins and cofactors | | | 11/505 | | 190/10867 | | 2.7E-01 | | 0.76 | | 0.70 | | ACACA/ENPP2/HSP90AA1/IDH1/LRP10/MOCS2/NOS3/NT5E/PARP8/SLC5A6/TPK1 | | 11 | |
| R-HSA-5693567 | | HDR through Homologous Recombination (HRR) or Single Strand Annealing (SSA) | | | 8/505 | | 132/10867 | | 2.7E-01 | | 0.76 | | 0.70 | | BLM/ERCC4/PALB2/PCNA/POLD4/PPP4R2/RAD50/RFC5 | | 8 | |
| R-HSA-110313 | | Translesion synthesis by Y family DNA polymerases bypasses lesions on DNA template | | | 3/505 | | 39/10867 | | 2.7E-01 | | 0.76 | | 0.70 | | PCNA/POLD4/RFC5 | | 3 | |
| R-HSA-111996 | | Ca-dependent events | | | 3/505 | | 39/10867 | | 2.7E-01 | | 0.76 | | 0.70 | | CAMK4/KPNA2/PLA2G4A | | 3 | |
| R-HSA-5693616 | | Presynaptic phase of homologous DNA pairing and strand exchange | | | 3/505 | | 39/10867 | | 2.7E-01 | | 0.76 | | 0.70 | | BLM/RAD50/RFC5 | | 3 | |
| R-HSA-9031628 | | NGF-stimulated transcription | | | 3/505 | | 39/10867 | | 2.7E-01 | | 0.76 | | 0.70 | | F3/SRF/TCF12 | | 3 | |
| R-HSA-1250196 | | SHC1 events in ERBB2 signaling | | | 2/505 | | 22/10867 | | 2.7E-01 | | 0.76 | | 0.70 | | ERBB3/ERBB4 | | 2 | |
| R-HSA-140875 | | Common Pathway of Fibrin Clot Formation | | | 2/505 | | 22/10867 | | 2.7E-01 | | 0.76 | | 0.70 | | F13A1/PROS1 | | 2 | |
| R-HSA-198753 | | ERK/MAPK targets | | | 2/505 | | 22/10867 | | 2.7E-01 | | 0.76 | | 0.70 | | DUSP4/MEF2C | | 2 | |
| R-HSA-392170 | | ADP signalling through P2Y purinoceptor 12 | | | 2/505 | | 22/10867 | | 2.7E-01 | | 0.76 | | 0.70 | | GNAI2/GNG12 | | 2 | |
| R-HSA-5654712 | | FRS-mediated FGFR4 signaling | | | 2/505 | | 22/10867 | | 2.7E-01 | | 0.76 | | 0.70 | | FGF18/FRS2 | | 2 | |
| R-HSA-9613829 | | Chaperone Mediated Autophagy | | | 2/505 | | 22/10867 | | 2.7E-01 | | 0.76 | | 0.70 | | HSP90AA1/VIM | | 2 | |
| R-HSA-195721 | | Signaling by WNT | | | 18/505 | | 332/10867 | | 2.8E-01 | | 0.78 | | 0.72 | | APC/CAV1/CLTC/CSNK1A1/CUL3/DAAM1/GNG12/GSK3B/ITPR1/KREMEN1/PRKG1/PSMC3/RAC2/SFRP1/WLS/WNT5A/WNT7B/XPO1 | | 18 | |
| R-HSA-168276 | | NS1 Mediated Effects on Host Pathways | | | 3/505 | | 40/10867 | | 2.8E-01 | | 0.78 | | 0.72 | | KPNA1/KPNA2/RANBP2 | | 3 | |
| R-HSA-5654741 | | Signaling by FGFR3 | | | 3/505 | | 40/10867 | | 2.8E-01 | | 0.78 | | 0.72 | | FGF18/FRS2/PIK3R1 | | 3 | |
| R-HSA-5674135 | | MAP2K and MAPK activation | | | 3/505 | | 40/10867 | | 2.8E-01 | | 0.78 | | 0.72 | | CSK/IQGAP1/TLN1 | | 3 | |
| R-HSA-6783310 | | Fanconi Anemia Pathway | | | 3/505 | | 40/10867 | | 2.8E-01 | | 0.78 | | 0.72 | | ERCC4/FANCI/FANCL | | 3 | |
| R-HSA-141424 | | Amplification of signal from the kinetochores | | | 6/505 | | 96/10867 | | 2.9E-01 | | 0.78 | | 0.72 | | CENPA/CENPQ/CLIP1/RANBP2/SGO1/XPO1 | | 6 | |
| R-HSA-141444 | | Amplification of signal from unattached kinetochores via a MAD2 inhibitory signal | | | 6/505 | | 96/10867 | | 2.9E-01 | | 0.78 | | 0.72 | | CENPA/CENPQ/CLIP1/RANBP2/SGO1/XPO1 | | 6 | |
| R-HSA-168138 | | Toll Like Receptor 9 (TLR9) Cascade | | | 6/505 | | 96/10867 | | 2.9E-01 | | 0.78 | | 0.72 | | DUSP4/IRF7/MAP3K1/MEF2C/TAB1/TLR4 | | 6 | |
| R-HSA-8878159 | | Transcriptional regulation by RUNX3 | | | 6/505 | | 96/10867 | | 2.9E-01 | | 0.78 | | 0.72 | | BRD2/CDKN1A/FOXO3/PSMC3/RUNX1/ZFHX3 | | 6 | |
| R-HSA-199977 | | ER to Golgi Anterograde Transport | | | 9/505 | | 154/10867 | | 2.9E-01 | | 0.78 | | 0.72 | | ARCN1/MIA3/SEC16A/SEC23A/SEC24A/SEC24B/SEC24C/SEC24D/TRAPPC1 | | 9 | |
| R-HSA-1482801 | | Acyl chain remodelling of PS | | | 2/505 | | 23/10867 | | 2.9E-01 | | 0.78 | | 0.72 | | PLA2G2A/PLA2G4A | | 2 | |
| R-HSA-166663 | | Initial triggering of complement | | | 2/505 | | 23/10867 | | 2.9E-01 | | 0.78 | | 0.72 | | C1QA/CFD | | 2 | |
| R-HSA-167242 | | Abortive elongation of HIV-1 transcript in the absence of Tat | | | 2/505 | | 23/10867 | | 2.9E-01 | | 0.78 | | 0.72 | | GTF2F1/NELFB | | 2 | |
| R-HSA-212676 | | Dopamine Neurotransmitter Release Cycle | | | 2/505 | | 23/10867 | | 2.9E-01 | | 0.78 | | 0.72 | | RAB3A/VAMP2 | | 2 | |
| R-HSA-9013418 | | RHOBTB2 GTPase cycle | | | 2/505 | | 23/10867 | | 2.9E-01 | | 0.78 | | 0.72 | | CUL3/HSP90AA1 | | 2 | |
| R-HSA-168273 | | Influenza Viral RNA Transcription and Replication | | | 8/505 | | 135/10867 | | 2.9E-01 | | 0.78 | | 0.72 | | GTF2F1/HSP90AA1/RANBP2/RPL32/RPL36AL/RPS14/RPS21/RPS9 | | 8 | |
| R-HSA-162906 | | HIV Infection | | | 13/505 | | 234/10867 | | 2.9E-01 | | 0.79 | | 0.72 | | AP1S1/CCNH/CHMP3/FYN/GTF2F1/HCK/KPNA1/LCK/NELFB/PSMC3/RANBP2/SUPT16H/XPO1 | | 13 | |
| R-HSA-73894 | | DNA Repair | | | 18/505 | | 335/10867 | | 3.0E-01 | | 0.79 | | 0.73 | | BLM/CCNH/COPS6/ERCC4/FANCI/FANCL/KPNA2/MSH6/PALB2/PCNA/POLD4/PPP4R2/RAD50/RFC5/RNF111/TERF2IP/XRCC1/YY1 | | 18 | |
| R-HSA-391251 | | Protein folding | | | 6/505 | | 97/10867 | | 3.0E-01 | | 0.79 | | 0.73 | | AP3M1/CCNE2/FBXL5/GNAQ/GNG12/LONP2 | | 6 | |
| R-HSA-165159 | | MTOR signalling | | | 3/505 | | 41/10867 | | 3.0E-01 | | 0.79 | | 0.73 | | CAB39/PRKAA1/PRKAA2 | | 3 | |
| R-HSA-5654743 | | Signaling by FGFR4 | | | 3/505 | | 41/10867 | | 3.0E-01 | | 0.79 | | 0.73 | | FGF18/FRS2/PIK3R1 | | 3 | |
| R-HSA-8875878 | | MET promotes cell motility | | | 3/505 | | 41/10867 | | 3.0E-01 | | 0.79 | | 0.73 | | COL1A1/COL5A1/ITGA2 | | 3 | |
| R-HSA-69275 | | G2/M Transition | | | 11/505 | | 196/10867 | | 3.0E-01 | | 0.79 | | 0.73 | | CCNB2/CCNH/CCP110/CDKN1A/FBXL7/HMMR/HSP90AA1/MYBL2/PSMC3/TUBGCP6/XPO1 | | 11 | |
| R-HSA-68882 | | Mitotic Anaphase | | | 13/505 | | 236/10867 | | 3.0E-01 | | 0.79 | | 0.73 | | CCNB2/CENPA/CENPQ/CHMP3/CLIP1/ESPL1/PDS5B/PSMC3/RANBP2/SGO1/VRK1/WAPL/XPO1 | | 13 | |
| R-HSA-168898 | | Toll-like Receptor Cascades | | | 9/505 | | 157/10867 | | 3.1E-01 | | 0.79 | | 0.73 | | BTK/DUSP4/IRF7/ITGB2/MAP3K1/MEF2C/RIPK3/TAB1/TLR4 | | 9 | |
| R-HSA-163560 | | Triglyceride catabolism | | | 2/505 | | 24/10867 | | 3.1E-01 | | 0.79 | | 0.73 | | CAV1/LIPE | | 2 | |
| R-HSA-202040 | | G-protein activation | | | 2/505 | | 24/10867 | | 3.1E-01 | | 0.79 | | 0.73 | | GNAQ/GNG12 | | 2 | |
| R-HSA-379716 | | Cytosolic tRNA aminoacylation | | | 2/505 | | 24/10867 | | 3.1E-01 | | 0.79 | | 0.73 | | FARSA/IARS1 | | 2 | |
| R-HSA-428930 | | Thromboxane signalling through TP receptor | | | 2/505 | | 24/10867 | | 3.1E-01 | | 0.79 | | 0.73 | | GNAQ/GNG12 | | 2 | |
| R-HSA-6783589 | | Interleukin-6 family signaling | | | 2/505 | | 24/10867 | | 3.1E-01 | | 0.79 | | 0.73 | | IL6ST/JAK1 | | 2 | |
| R-HSA-977068 | | Termination of O-glycan biosynthesis | | | 2/505 | | 24/10867 | | 3.1E-01 | | 0.79 | | 0.73 | | ST3GAL2/ST6GALNAC3 | | 2 | |
| R-HSA-8978868 | | Fatty acid metabolism | | | 10/505 | | 177/10867 | | 3.1E-01 | | 0.79 | | 0.73 | | ACACA/ACSL3/ACSL4/CROT/HACD2/HPGD/PLA2G4A/PRKAA2/PTGES2/SCD | | 10 | |
| R-HSA-2555396 | | Mitotic Metaphase and Anaphase | | | 13/505 | | 237/10867 | | 3.1E-01 | | 0.79 | | 0.73 | | CCNB2/CENPA/CENPQ/CHMP3/CLIP1/ESPL1/PDS5B/PSMC3/RANBP2/SGO1/VRK1/WAPL/XPO1 | | 13 | |
| R-HSA-2173782 | | Binding and Uptake of Ligands by Scavenger Receptors | | | 3/505 | | 42/10867 | | 3.1E-01 | | 0.79 | | 0.73 | | COL1A1/HSP90AA1/SPARC | | 3 | |
| R-HSA-2426168 | | Activation of gene expression by SREBF (SREBP) | | | 3/505 | | 42/10867 | | 3.1E-01 | | 0.79 | | 0.73 | | ACACA/SCD/SP1 | | 3 | |
| R-HSA-381676 | | Glucagon-like Peptide-1 (GLP1) regulates insulin secretion | | | 3/505 | | 42/10867 | | 3.1E-01 | | 0.79 | | 0.73 | | GNG12/IQGAP1/ITPR1 | | 3 | |
| R-HSA-3928662 | | EPHB-mediated forward signaling | | | 3/505 | | 42/10867 | | 3.1E-01 | | 0.79 | | 0.73 | | ARPC4/CFL1/FYN | | 3 | |
| R-HSA-419037 | | NCAM1 interactions | | | 3/505 | | 42/10867 | | 3.1E-01 | | 0.79 | | 0.73 | | CACNB4/COL5A1/ST8SIA4 | | 3 | |
| R-HSA-166166 | | MyD88-independent TLR4 cascade | | | 6/505 | | 99/10867 | | 3.1E-01 | | 0.80 | | 0.74 | | DUSP4/IRF7/MEF2C/RIPK3/TAB1/TLR4 | | 6 | |
| R-HSA-937061 | | TRIF(TICAM1)-mediated TLR4 signaling | | | 6/505 | | 99/10867 | | 3.1E-01 | | 0.80 | | 0.74 | | DUSP4/IRF7/MEF2C/RIPK3/TAB1/TLR4 | | 6 | |
| R-HSA-1169410 | | Antiviral mechanism by IFN-stimulated genes | | | 5/505 | | 80/10867 | | 3.1E-01 | | 0.80 | | 0.74 | | JAK1/KPNA1/KPNA2/NEDD4/RANBP2 | | 5 | |
| R-HSA-438064 | | Post NMDA receptor activation events | | | 5/505 | | 80/10867 | | 3.1E-01 | | 0.80 | | 0.74 | | CAMK4/ERBB4/KPNA2/PRKAA1/PRKAA2 | | 5 | |
| R-HSA-2022090 | | Assembly of collagen fibrils and other multimeric structures | | | 4/505 | | 61/10867 | | 3.1E-01 | | 0.80 | | 0.74 | | COL17A1/COL1A1/COL5A1/DST | | 4 | |
| R-HSA-453274 | | Mitotic G2-G2/M phases | | | 11/505 | | 198/10867 | | 3.1E-01 | | 0.80 | | 0.74 | | CCNB2/CCNH/CCP110/CDKN1A/FBXL7/HMMR/HSP90AA1/MYBL2/PSMC3/TUBGCP6/XPO1 | | 11 | |
| R-HSA-373760 | | L1CAM interactions | | | 7/505 | | 119/10867 | | 3.2E-01 | | 0.80 | | 0.74 | | ALCAM/CLTC/ITGA2/MSN/NRP1/NUMB/SCN1B | | 7 | |
| R-HSA-1489509 | | DAG and IP3 signaling | | | 3/505 | | 43/10867 | | 3.2E-01 | | 0.80 | | 0.74 | | CAMK4/ITPR1/KPNA2 | | 3 | |
| R-HSA-5362517 | | Signaling by Retinoic Acid | | | 3/505 | | 43/10867 | | 3.2E-01 | | 0.80 | | 0.74 | | ADH1A/ADH1C/ALDH1A1 | | 3 | |
| R-HSA-5696395 | | Formation of Incision Complex in GG-NER | | | 3/505 | | 43/10867 | | 3.2E-01 | | 0.80 | | 0.74 | | CCNH/ERCC4/RNF111 | | 3 | |
| R-HSA-9656223 | | Signaling by RAF1 mutants | | | 3/505 | | 43/10867 | | 3.2E-01 | | 0.80 | | 0.74 | | CSK/IQGAP1/TLN1 | | 3 | |
| R-HSA-1643713 | | Signaling by EGFR in Cancer | | | 2/505 | | 25/10867 | | 3.2E-01 | | 0.80 | | 0.74 | | HSP90AA1/PIK3R1 | | 2 | |
| R-HSA-191273 | | Cholesterol biosynthesis | | | 2/505 | | 25/10867 | | 3.2E-01 | | 0.80 | | 0.74 | | DHCR24/MSMO1 | | 2 | |
| R-HSA-210991 | | Basigin interactions | | | 2/505 | | 25/10867 | | 3.2E-01 | | 0.80 | | 0.74 | | BSG/CAV1 | | 2 | |
| R-HSA-400685 | | Sema4D in semaphorin signaling | | | 2/505 | | 25/10867 | | 3.2E-01 | | 0.80 | | 0.74 | | ARHGEF12/RHOB | | 2 | |
| R-HSA-5654700 | | FRS-mediated FGFR2 signaling | | | 2/505 | | 25/10867 | | 3.2E-01 | | 0.80 | | 0.74 | | FGF18/FRS2 | | 2 | |
| R-HSA-8854691 | | Interleukin-20 family signaling | | | 2/505 | | 25/10867 | | 3.2E-01 | | 0.80 | | 0.74 | | JAK1/STAT4 | | 2 | |
| R-HSA-937041 | | IKK complex recruitment mediated by RIP1 | | | 2/505 | | 25/10867 | | 3.2E-01 | | 0.80 | | 0.74 | | RIPK3/TLR4 | | 2 | |
| R-HSA-9705462 | | Inactivation of CSF3 (G-CSF) signaling | | | 2/505 | | 25/10867 | | 3.2E-01 | | 0.80 | | 0.74 | | HCK/JAK1 | | 2 | |
| R-HSA-4086398 | | Ca2+ pathway | | | 4/505 | | 62/10867 | | 3.3E-01 | | 0.80 | | 0.74 | | GNG12/ITPR1/PRKG1/WNT5A | | 4 | |
| R-HSA-166058 | | MyD88:MAL(TIRAP) cascade initiated on plasma membrane | | | 6/505 | | 101/10867 | | 3.3E-01 | | 0.81 | | 0.74 | | BTK/DUSP4/MAP3K1/MEF2C/TAB1/TLR4 | | 6 | |
| R-HSA-168188 | | Toll Like Receptor TLR6:TLR2 Cascade | | | 6/505 | | 101/10867 | | 3.3E-01 | | 0.81 | | 0.74 | | BTK/DUSP4/MAP3K1/MEF2C/TAB1/TLR4 | | 6 | |
| R-HSA-109704 | | PI3K Cascade | | | 3/505 | | 44/10867 | | 3.4E-01 | | 0.81 | | 0.75 | | FGF18/FRS2/PIK3R1 | | 3 | |
| R-HSA-1660661 | | Sphingolipid de novo biosynthesis | | | 3/505 | | 44/10867 | | 3.4E-01 | | 0.81 | | 0.75 | | CERT1/PLPP3/SGMS2 | | 3 | |
| R-HSA-425397 | | Transport of vitamins. nucleosides. and related molecules | | | 3/505 | | 44/10867 | | 3.4E-01 | | 0.81 | | 0.75 | | SLC35D1/SLC5A6/SLCO2A1 | | 3 | |
| R-HSA-8948216 | | Collagen chain trimerization | | | 3/505 | | 44/10867 | | 3.4E-01 | | 0.81 | | 0.75 | | COL17A1/COL1A1/COL5A1 | | 3 | |
| R-HSA-1226099 | | Signaling by FGFR in disease | | | 4/505 | | 63/10867 | | 3.4E-01 | | 0.81 | | 0.75 | | FGF18/FRS2/GTF2F1/PIK3R1 | | 4 | |
| R-HSA-1834949 | | Cytosolic sensors of pathogen-associated DNA | | | 4/505 | | 63/10867 | | 3.4E-01 | | 0.81 | | 0.75 | | DHX9/IFI16/IRF7/RIPK3 | | 4 | |
| R-HSA-375165 | | NCAM signaling for neurite out-growth | | | 4/505 | | 63/10867 | | 3.4E-01 | | 0.81 | | 0.75 | | CACNB4/COL5A1/FYN/ST8SIA4 | | 4 | |
| R-HSA-9033241 | | Peroxisomal protein import | | | 4/505 | | 63/10867 | | 3.4E-01 | | 0.81 | | 0.75 | | CROT/IDH1/LONP2/PEX1 | | 4 | |
| R-HSA-913709 | | O-linked glycosylation of mucins | | | 4/505 | | 63/10867 | | 3.4E-01 | | 0.81 | | 0.75 | | GALNT1/GALNT7/ST3GAL2/ST6GALNAC3 | | 4 | |
| R-HSA-425410 | | Metal ion SLC transporters | | | 2/505 | | 26/10867 | | 3.4E-01 | | 0.82 | | 0.75 | | SLC39A6/SLC41A2 | | 2 | |
| R-HSA-901042 | | Calnexin/calreticulin cycle | | | 2/505 | | 26/10867 | | 3.4E-01 | | 0.82 | | 0.75 | | RNF103/SEL1L | | 2 | |
| R-HSA-912694 | | Regulation of IFNA signaling | | | 2/505 | | 26/10867 | | 3.4E-01 | | 0.82 | | 0.75 | | JAK1/PTPN6 | | 2 | |
| R-HSA-9660826 | | Purinergic signaling in leishmaniasis infection | | | 2/505 | | 26/10867 | | 3.4E-01 | | 0.82 | | 0.75 | | NT5E/TXNIP | | 2 | |
| R-HSA-9664424 | | Cell recruitment (pro-inflammatory response) | | | 2/505 | | 26/10867 | | 3.4E-01 | | 0.82 | | 0.75 | | NT5E/TXNIP | | 2 | |
| R-HSA-196849 | | Metabolism of water-soluble vitamins and cofactors | | | 7/505 | | 123/10867 | | 3.5E-01 | | 0.83 | | 0.76 | | ACACA/ENPP2/MOCS2/NT5E/PARP8/SLC5A6/TPK1 | | 7 | |
| R-HSA-1483255 | | PI Metabolism | | | 5/505 | | 84/10867 | | 3.5E-01 | | 0.83 | | 0.77 | | INPP4B/INPP5E/PIK3R1/RAB14/TNFAIP8 | | 5 | |
| R-HSA-6802957 | | Oncogenic MAPK signaling | | | 5/505 | | 84/10867 | | 3.5E-01 | | 0.83 | | 0.77 | | AP3B1/CSK/DUSP10/IQGAP1/TLN1 | | 5 | |
| R-HSA-168179 | | Toll Like Receptor TLR1:TLR2 Cascade | | | 6/505 | | 104/10867 | | 3.5E-01 | | 0.83 | | 0.77 | | BTK/DUSP4/MAP3K1/MEF2C/TAB1/TLR4 | | 6 | |
| R-HSA-181438 | | Toll Like Receptor 2 (TLR2) Cascade | | | 6/505 | | 104/10867 | | 3.5E-01 | | 0.83 | | 0.77 | | BTK/DUSP4/MAP3K1/MEF2C/TAB1/TLR4 | | 6 | |
| R-HSA-1630316 | | Glycosaminoglycan metabolism | | | 7/505 | | 124/10867 | | 3.5E-01 | | 0.83 | | 0.77 | | CHP1/CHST15/CHST7/HMMR/HYAL1/ST3GAL2/VCAN | | 7 | |
| R-HSA-114508 | | Effects of PIP2 hydrolysis | | | 2/505 | | 27/10867 | | 3.6E-01 | | 0.83 | | 0.77 | | DGKA/ITPR1 | | 2 | |
| R-HSA-167160 | | RNA Pol II CTD phosphorylation and interaction with CE during HIV infection | | | 2/505 | | 27/10867 | | 3.6E-01 | | 0.83 | | 0.77 | | CCNH/GTF2F1 | | 2 | |
| R-HSA-354192 | | Integrin signaling | | | 2/505 | | 27/10867 | | 3.6E-01 | | 0.83 | | 0.77 | | CSK/TLN1 | | 2 | |
| R-HSA-77075 | | RNA Pol II CTD phosphorylation and interaction with CE | | | 2/505 | | 27/10867 | | 3.6E-01 | | 0.83 | | 0.77 | | CCNH/GTF2F1 | | 2 | |
| R-HSA-425366 | | Transport of bile salts and organic acids. metal ions and amine compounds | | | 5/505 | | 85/10867 | | 3.6E-01 | | 0.83 | | 0.77 | | BSG/RUNX1/SLC39A6/SLC41A2/SLC6A12 | | 5 | |
| R-HSA-3858494 | | Beta-catenin independent WNT signaling | | | 8/505 | | 146/10867 | | 3.7E-01 | | 0.83 | | 0.77 | | CLTC/DAAM1/GNG12/ITPR1/PRKG1/PSMC3/RAC2/WNT5A | | 8 | |
| R-HSA-437239 | | Recycling pathway of L1 | | | 3/505 | | 47/10867 | | 3.7E-01 | | 0.83 | | 0.77 | | CLTC/MSN/NUMB | | 3 | |
| R-HSA-6802946 | | Signaling by moderate kinase activity BRAF mutants | | | 3/505 | | 47/10867 | | 3.7E-01 | | 0.83 | | 0.77 | | CSK/IQGAP1/TLN1 | | 3 | |
| R-HSA-6802949 | | Signaling by RAS mutants | | | 3/505 | | 47/10867 | | 3.7E-01 | | 0.83 | | 0.77 | | CSK/IQGAP1/TLN1 | | 3 | |
| R-HSA-6802955 | | Paradoxical activation of RAF signaling by kinase inactive BRAF | | | 3/505 | | 47/10867 | | 3.7E-01 | | 0.83 | | 0.77 | | CSK/IQGAP1/TLN1 | | 3 | |
| R-HSA-73762 | | RNA Polymerase I Transcription Initiation | | | 3/505 | | 47/10867 | | 3.7E-01 | | 0.83 | | 0.77 | | CCNH/MBD3/MTA3 | | 3 | |
| R-HSA-9024446 | | NR1H2 and NR1H3-mediated signaling | | | 3/505 | | 47/10867 | | 3.7E-01 | | 0.83 | | 0.77 | | MYLIP/NRIP1/SCD | | 3 | |
| R-HSA-9649948 | | Signaling downstream of RAS mutants | | | 3/505 | | 47/10867 | | 3.7E-01 | | 0.83 | | 0.77 | | CSK/IQGAP1/TLN1 | | 3 | |
| R-HSA-1482788 | | Acyl chain remodelling of PC | | | 2/505 | | 28/10867 | | 3.8E-01 | | 0.83 | | 0.77 | | PLA2G2A/PLA2G4A | | 2 | |
| R-HSA-3295583 | | TRP channels | | | 2/505 | | 28/10867 | | 3.8E-01 | | 0.83 | | 0.77 | | RIPK3/TRPM4 | | 2 | |
| R-HSA-69205 | | G1/S-Specific Transcription | | | 2/505 | | 28/10867 | | 3.8E-01 | | 0.83 | | 0.77 | | PCNA/RRM2 | | 2 | |
| R-HSA-9682385 | | FLT3 signaling in disease | | | 2/505 | | 28/10867 | | 3.8E-01 | | 0.83 | | 0.77 | | CDKN1A/PIK3R1 | | 2 | |
| R-HSA-1483115 | | Hydrolysis of LPC | | | 1/505 | | 10/10867 | | 3.8E-01 | | 0.83 | | 0.77 | | PLA2G4A | | 1 | |
| R-HSA-159740 | | Gamma-carboxylation of protein precursors | | | 1/505 | | 10/10867 | | 3.8E-01 | | 0.83 | | 0.77 | | PROS1 | | 1 | |
| R-HSA-159782 | | Removal of aminoterminal propeptides from gamma-carboxylated proteins | | | 1/505 | | 10/10867 | | 3.8E-01 | | 0.83 | | 0.77 | | PROS1 | | 1 | |
| R-HSA-164940 | | Nef mediated downregulation of MHC class I complex cell surface expression | | | 1/505 | | 10/10867 | | 3.8E-01 | | 0.83 | | 0.77 | | AP1S1 | | 1 | |
| R-HSA-1855167 | | Synthesis of pyrophosphates in the cytosol | | | 1/505 | | 10/10867 | | 3.8E-01 | | 0.83 | | 0.77 | | NUDT4 | | 1 | |
| R-HSA-193697 | | p75NTR regulates axonogenesis | | | 1/505 | | 10/10867 | | 3.8E-01 | | 0.83 | | 0.77 | | ARHGDIA | | 1 | |
| R-HSA-2151209 | | Activation of PPARGC1A (PGC-1alpha) by phosphorylation | | | 1/505 | | 10/10867 | | 3.8E-01 | | 0.83 | | 0.77 | | PRKAA2 | | 1 | |
| R-HSA-2161522 | | Abacavir transport and metabolism | | | 1/505 | | 10/10867 | | 3.8E-01 | | 0.83 | | 0.77 | | ADH1A | | 1 | |
| R-HSA-399997 | | Acetylcholine regulates insulin secretion | | | 1/505 | | 10/10867 | | 3.8E-01 | | 0.83 | | 0.77 | | GNAQ | | 1 | |
| R-HSA-428542 | | Regulation of commissural axon pathfinding by SLIT and ROBO | | | 1/505 | | 10/10867 | | 3.8E-01 | | 0.83 | | 0.77 | | SLIT2 | | 1 | |
| R-HSA-442380 | | Zinc influx into cells by the SLC39 gene family | | | 1/505 | | 10/10867 | | 3.8E-01 | | 0.83 | | 0.77 | | SLC39A6 | | 1 | |
| R-HSA-549127 | | Organic cation transport | | | 1/505 | | 10/10867 | | 3.8E-01 | | 0.83 | | 0.77 | | RUNX1 | | 1 | |
| R-HSA-8866904 | | Negative regulation of activity of TFAP2 (AP-2) family transcription factors | | | 1/505 | | 10/10867 | | 3.8E-01 | | 0.83 | | 0.77 | | TFAP2A | | 1 | |
| R-HSA-937042 | | IRAK2 mediated activation of TAK1 complex | | | 1/505 | | 10/10867 | | 3.8E-01 | | 0.83 | | 0.77 | | TAB1 | | 1 | |
| R-HSA-9702518 | | STAT5 activation downstream of FLT3 ITD mutants | | | 1/505 | | 10/10867 | | 3.8E-01 | | 0.83 | | 0.77 | | CDKN1A | | 1 | |
| R-HSA-9706019 | | RHOBTB3 ATPase cycle | | | 1/505 | | 10/10867 | | 3.8E-01 | | 0.83 | | 0.77 | | CUL3 | | 1 | |
| R-HSA-6802952 | | Signaling by BRAF and RAF1 fusions | | | 4/505 | | 67/10867 | | 3.8E-01 | | 0.83 | | 0.77 | | AP3B1/CSK/IQGAP1/TLN1 | | 4 | |
| R-HSA-112399 | | IRS-mediated signalling | | | 3/505 | | 48/10867 | | 3.9E-01 | | 0.83 | | 0.77 | | FGF18/FRS2/PIK3R1 | | 3 | |
| R-HSA-73893 | | DNA Damage Bypass | | | 3/505 | | 48/10867 | | 3.9E-01 | | 0.83 | | 0.77 | | PCNA/POLD4/RFC5 | | 3 | |
| R-HSA-450531 | | Regulation of mRNA stability by proteins that bind AU-rich elements | | | 5/505 | | 88/10867 | | 3.9E-01 | | 0.83 | | 0.77 | | ELAVL1/PSMC3/XPO1/ZFP36/ZFP36L1 | | 5 | |
| R-HSA-3906995 | | Diseases associated with O-glycosylation of proteins | | | 4/505 | | 68/10867 | | 3.9E-01 | | 0.83 | | 0.77 | | SEMA5A/THBS1/THSD4/THSD7A | | 4 | |
| R-HSA-5358351 | | Signaling by Hedgehog | | | 8/505 | | 149/10867 | | 3.9E-01 | | 0.83 | | 0.77 | | BOC/CSNK1A1/CUL3/GSK3B/NUMB/PSMC3/SEL1L/SPOPL | | 8 | |
| R-HSA-69620 | | Cell Cycle Checkpoints | | | 15/505 | | 294/10867 | | 3.9E-01 | | 0.83 | | 0.77 | | BLM/CCNB2/CCNE2/CDKN1A/CENPA/CENPQ/CLIP1/PHF20/PSMC3/RAD50/RANBP2/RFC5/SGO1/XPO1/ZNF385A | | 15 | |
| R-HSA-5213460 | | RIPK1-mediated regulated necrosis | | | 2/505 | | 29/10867 | | 3.9E-01 | | 0.83 | | 0.77 | | HSP90AA1/RIPK3 | | 2 | |
| R-HSA-5654732 | | Negative regulation of FGFR3 signaling | | | 2/505 | | 29/10867 | | 3.9E-01 | | 0.83 | | 0.77 | | FGF18/FRS2 | | 2 | |
| R-HSA-5675482 | | Regulation of necroptotic cell death | | | 2/505 | | 29/10867 | | 3.9E-01 | | 0.83 | | 0.77 | | HSP90AA1/RIPK3 | | 2 | |
| R-HSA-72086 | | mRNA Capping | | | 2/505 | | 29/10867 | | 3.9E-01 | | 0.83 | | 0.77 | | CCNH/GTF2F1 | | 2 | |
| R-HSA-9619483 | | Activation of AMPK downstream of NMDARs | | | 2/505 | | 29/10867 | | 3.9E-01 | | 0.83 | | 0.77 | | PRKAA1/PRKAA2 | | 2 | |
| R-HSA-9694548 | | Maturation of spike protein | | | 2/505 | | 29/10867 | | 3.9E-01 | | 0.83 | | 0.77 | | ST3GAL2/ST6GALNAC3 | | 2 | |
| R-HSA-2467813 | | Separation of Sister Chromatids | | | 10/505 | | 191/10867 | | 4.0E-01 | | 0.83 | | 0.77 | | CENPA/CENPQ/CLIP1/ESPL1/PDS5B/PSMC3/RANBP2/SGO1/WAPL/XPO1 | | 10 | |
| R-HSA-2672351 | | Stimuli-sensing channels | | | 6/505 | | 109/10867 | | 4.0E-01 | | 0.83 | | 0.77 | | ANO9/ASPH/CLCN3/RIPK3/TRPM4/TSC22D3 | | 6 | |
| R-HSA-156902 | | Peptide chain elongation | | | 5/505 | | 89/10867 | | 4.0E-01 | | 0.83 | | 0.77 | | RPL32/RPL36AL/RPS14/RPS21/RPS9 | | 5 | |
| R-HSA-192823 | | Viral mRNA Translation | | | 5/505 | | 89/10867 | | 4.0E-01 | | 0.83 | | 0.77 | | RPL32/RPL36AL/RPS14/RPS21/RPS9 | | 5 | |
| R-HSA-8951664 | | Neddylation | | | 12/505 | | 234/10867 | | 4.1E-01 | | 0.83 | | 0.77 | | CCNF/COPS6/CUL3/FBXL5/FBXL7/FBXL8/FBXO22/FBXO31/FEM1C/KLHL2/PSMC3/TULP4 | | 12 | |
| R-HSA-1234158 | | Regulation of gene expression by Hypoxia-inducible Factor | | | 1/505 | | 11/10867 | | 4.1E-01 | | 0.83 | | 0.77 | | CITED2 | | 1 | |
| R-HSA-159854 | | Gamma-carboxylation. transport. and amino-terminal cleavage of proteins | | | 1/505 | | 11/10867 | | 4.1E-01 | | 0.83 | | 0.77 | | PROS1 | | 1 | |
| R-HSA-196025 | | Formation of annular gap junctions | | | 1/505 | | 11/10867 | | 4.1E-01 | | 0.83 | | 0.77 | | CLTC | | 1 | |
| R-HSA-2022923 | | Dermatan sulfate biosynthesis | | | 1/505 | | 11/10867 | | 4.1E-01 | | 0.83 | | 0.77 | | VCAN | | 1 | |
| R-HSA-2206281 | | Mucopolysaccharidoses | | | 1/505 | | 11/10867 | | 4.1E-01 | | 0.83 | | 0.77 | | HYAL1 | | 1 | |
| R-HSA-351906 | | Apoptotic cleavage of cell adhesion proteins | | | 1/505 | | 11/10867 | | 4.1E-01 | | 0.83 | | 0.77 | | TJP1 | | 1 | |
| R-HSA-71288 | | Creatine metabolism | | | 1/505 | | 11/10867 | | 4.1E-01 | | 0.83 | | 0.77 | | SLC6A12 | | 1 | |
| R-HSA-75072 | | mRNA Editing | | | 1/505 | | 11/10867 | | 4.1E-01 | | 0.83 | | 0.77 | | ADARB1 | | 1 | |
| R-HSA-9013700 | | NOTCH4 Activation and Transmission of Signal to the Nucleus | | | 1/505 | | 11/10867 | | 4.1E-01 | | 0.83 | | 0.77 | | ADAM10 | | 1 | |
| R-HSA-9014325 | | TICAM1.TRAF6-dependent induction of TAK1 complex | | | 1/505 | | 11/10867 | | 4.1E-01 | | 0.83 | | 0.77 | | TAB1 | | 1 | |
| R-HSA-9028731 | | Activated NTRK2 signals through FRS2 and FRS3 | | | 1/505 | | 11/10867 | | 4.1E-01 | | 0.83 | | 0.77 | | FRS2 | | 1 | |
| R-HSA-9645460 | | Alpha-protein kinase 1 signaling pathway | | | 1/505 | | 11/10867 | | 4.1E-01 | | 0.83 | | 0.77 | | TAB1 | | 1 | |
| R-HSA-9664873 | | Pexophagy | | | 1/505 | | 11/10867 | | 4.1E-01 | | 0.83 | | 0.77 | | SQSTM1 | | 1 | |
| R-HSA-9690406 | | Transcriptional regulation of testis differentiation | | | 1/505 | | 11/10867 | | 4.1E-01 | | 0.83 | | 0.77 | | ZFPM2 | | 1 | |
| R-HSA-69206 | | G1/S Transition | | | 7/505 | | 131/10867 | | 4.1E-01 | | 0.83 | | 0.77 | | CCNE2/CCNH/CDKN1A/GMNN/PCNA/PSMC3/RRM2 | | 7 | |
| R-HSA-1482839 | | Acyl chain remodelling of PE | | | 2/505 | | 30/10867 | | 4.1E-01 | | 0.83 | | 0.77 | | PLA2G2A/PLA2G4A | | 2 | |
| R-HSA-418360 | | Platelet calcium homeostasis | | | 2/505 | | 30/10867 | | 4.1E-01 | | 0.83 | | 0.77 | | ATP2A2/ITPR1 | | 2 | |
| R-HSA-499943 | | Interconversion of nucleotide di- and triphosphates | | | 2/505 | | 30/10867 | | 4.1E-01 | | 0.83 | | 0.77 | | NME3/RRM2 | | 2 | |
| R-HSA-5357956 | | TNFR1-induced NFkappaB signaling pathway | | | 2/505 | | 30/10867 | | 4.1E-01 | | 0.83 | | 0.77 | | RACK1/TAB1 | | 2 | |
| R-HSA-9674555 | | Signaling by CSF3 (G-CSF) | | | 2/505 | | 30/10867 | | 4.1E-01 | | 0.83 | | 0.77 | | HCK/JAK1 | | 2 | |
| R-HSA-162587 | | HIV Life Cycle | | | 8/505 | | 152/10867 | | 4.1E-01 | | 0.83 | | 0.77 | | CCNH/CHMP3/GTF2F1/KPNA1/NELFB/RANBP2/SUPT16H/XPO1 | | 8 | |
| R-HSA-3214842 | | HDMs demethylate histones | | | 3/505 | | 50/10867 | | 4.1E-01 | | 0.83 | | 0.77 | | ARID5B/KDM5B/KDM6A | | 3 | |
| R-HSA-5654736 | | Signaling by FGFR1 | | | 3/505 | | 50/10867 | | 4.1E-01 | | 0.83 | | 0.77 | | FGF18/FRS2/PIK3R1 | | 3 | |
| R-HSA-2980736 | | Peptide hormone metabolism | | | 5/505 | | 91/10867 | | 4.2E-01 | | 0.83 | | 0.77 | | ERO1B/IGF1/INHBA/PCSK1/VAMP2 | | 5 | |
| R-HSA-975138 | | TRAF6 mediated induction of NFkB and MAP kinases upon TLR7/8 or 9 activation | | | 5/505 | | 91/10867 | | 4.2E-01 | | 0.83 | | 0.77 | | DUSP4/MAP3K1/MEF2C/TAB1/TLR4 | | 5 | |
| R-HSA-416476 | | G alpha (q) signalling events | | | 11/505 | | 216/10867 | | 4.2E-01 | | 0.83 | | 0.77 | | BTK/DGKA/GNAQ/GNG12/HRH1/ITPR1/P2RY11/PIK3R1/RGS2/RGS4/RGS5 | | 11 | |
| R-HSA-3928665 | | EPH-ephrin mediated repulsion of cells | | | 3/505 | | 51/10867 | | 4.2E-01 | | 0.83 | | 0.77 | | ADAM10/CLTC/FYN | | 3 | |
| R-HSA-6811436 | | COPI-independent Golgi-to-ER retrograde traffic | | | 3/505 | | 51/10867 | | 4.2E-01 | | 0.83 | | 0.77 | | GALNT1/PAFAH1B2/PLA2G4A | | 3 | |
| R-HSA-72695 | | Formation of the ternary complex. and subsequently. the 43S complex | | | 3/505 | | 51/10867 | | 4.2E-01 | | 0.83 | | 0.77 | | RPS14/RPS21/RPS9 | | 3 | |
| R-HSA-168271 | | Transport of Ribonucleoproteins into the Host Nucleus | | | 2/505 | | 31/10867 | | 4.3E-01 | | 0.83 | | 0.77 | | KPNA1/RANBP2 | | 2 | |
| R-HSA-168333 | | NEP/NS2 Interacts with the Cellular Export Machinery | | | 2/505 | | 31/10867 | | 4.3E-01 | | 0.83 | | 0.77 | | RANBP2/XPO1 | | 2 | |
| R-HSA-196807 | | Nicotinate metabolism | | | 2/505 | | 31/10867 | | 4.3E-01 | | 0.83 | | 0.77 | | NT5E/PARP8 | | 2 | |
| R-HSA-2122948 | | Activated NOTCH1 Transmits Signal to the Nucleus | | | 2/505 | | 31/10867 | | 4.3E-01 | | 0.83 | | 0.77 | | ADAM10/NUMB | | 2 | |
| R-HSA-450282 | | MAPK targets/ Nuclear events mediated by MAP kinases | | | 2/505 | | 31/10867 | | 4.3E-01 | | 0.83 | | 0.77 | | DUSP4/MEF2C | | 2 | |
| R-HSA-5260271 | | Diseases of Immune System | | | 2/505 | | 31/10867 | | 4.3E-01 | | 0.83 | | 0.77 | | BTK/TLR4 | | 2 | |
| R-HSA-5602358 | | Diseases associated with the TLR signaling cascade | | | 2/505 | | 31/10867 | | 4.3E-01 | | 0.83 | | 0.77 | | BTK/TLR4 | | 2 | |
| R-HSA-5654687 | | Downstream signaling of activated FGFR1 | | | 2/505 | | 31/10867 | | 4.3E-01 | | 0.83 | | 0.77 | | FRS2/PIK3R1 | | 2 | |
| R-HSA-5654733 | | Negative regulation of FGFR4 signaling | | | 2/505 | | 31/10867 | | 4.3E-01 | | 0.83 | | 0.77 | | FGF18/FRS2 | | 2 | |
| R-HSA-8963899 | | Plasma lipoprotein remodeling | | | 2/505 | | 31/10867 | | 4.3E-01 | | 0.83 | | 0.77 | | CIDEC/PCSK5 | | 2 | |
| R-HSA-917977 | | Transferrin endocytosis and recycling | | | 2/505 | | 31/10867 | | 4.3E-01 | | 0.83 | | 0.77 | | HFE/TFRC | | 2 | |
| R-HSA-2682334 | | EPH-Ephrin signaling | | | 5/505 | | 92/10867 | | 4.3E-01 | | 0.83 | | 0.77 | | ADAM10/ARPC4/CFL1/CLTC/FYN | | 5 | |
| R-HSA-4086400 | | PCP/CE pathway | | | 5/505 | | 92/10867 | | 4.3E-01 | | 0.83 | | 0.77 | | CLTC/DAAM1/PSMC3/RAC2/WNT5A | | 5 | |
| R-HSA-73884 | | Base Excision Repair | | | 5/505 | | 92/10867 | | 4.3E-01 | | 0.83 | | 0.77 | | PCNA/POLD4/RFC5/TERF2IP/XRCC1 | | 5 | |
| R-HSA-69618 | | Mitotic Spindle Checkpoint | | | 6/505 | | 113/10867 | | 4.3E-01 | | 0.83 | | 0.77 | | CENPA/CENPQ/CLIP1/RANBP2/SGO1/XPO1 | | 6 | |
| R-HSA-2454202 | | Fc epsilon receptor (FCERI) signaling | | | 7/505 | | 134/10867 | | 4.3E-01 | | 0.83 | | 0.77 | | BTK/FYN/ITPR1/MAP3K1/PIK3R1/PSMC3/TAB1 | | 7 | |
| R-HSA-112308 | | Presynaptic depolarization and calcium channel opening | | | 1/505 | | 12/10867 | | 4.4E-01 | | 0.83 | | 0.77 | | CACNB4 | | 1 | |
| R-HSA-1296346 | | Tandem pore domain potassium channels | | | 1/505 | | 12/10867 | | 4.4E-01 | | 0.83 | | 0.77 | | KCNK10 | | 1 | |
| R-HSA-170968 | | Frs2-mediated activation | | | 1/505 | | 12/10867 | | 4.4E-01 | | 0.83 | | 0.77 | | FRS2 | | 1 | |
| R-HSA-174403 | | Glutathione synthesis and recycling | | | 1/505 | | 12/10867 | | 4.4E-01 | | 0.83 | | 0.77 | | GGCT | | 1 | |
| R-HSA-1839130 | | Signaling by activated point mutants of FGFR3 | | | 1/505 | | 12/10867 | | 4.4E-01 | | 0.83 | | 0.77 | | FGF18 | | 1 | |
| R-HSA-190873 | | Gap junction degradation | | | 1/505 | | 12/10867 | | 4.4E-01 | | 0.83 | | 0.77 | | CLTC | | 1 | |
| R-HSA-196108 | | Pregnenolone biosynthesis | | | 1/505 | | 12/10867 | | 4.4E-01 | | 0.83 | | 0.77 | | FDXR | | 1 | |
| R-HSA-2033514 | | FGFR3 mutant receptor activation | | | 1/505 | | 12/10867 | | 4.4E-01 | | 0.83 | | 0.77 | | FGF18 | | 1 | |
| R-HSA-209822 | | Glycoprotein hormones | | | 1/505 | | 12/10867 | | 4.4E-01 | | 0.83 | | 0.77 | | INHBA | | 1 | |
| R-HSA-211979 | | Eicosanoids | | | 1/505 | | 12/10867 | | 4.4E-01 | | 0.83 | | 0.77 | | CYP4F12 | | 1 | |
| R-HSA-264870 | | Caspase-mediated cleavage of cytoskeletal proteins | | | 1/505 | | 12/10867 | | 4.4E-01 | | 0.83 | | 0.77 | | VIM | | 1 | |
| R-HSA-3371511 | | HSF1 activation | | | 1/505 | | 12/10867 | | 4.4E-01 | | 0.83 | | 0.77 | | HSP90AA1 | | 1 | |
| R-HSA-417957 | | P2Y receptors | | | 1/505 | | 12/10867 | | 4.4E-01 | | 0.83 | | 0.77 | | P2RY11 | | 1 | |
| R-HSA-71064 | | Lysine catabolism | | | 1/505 | | 12/10867 | | 4.4E-01 | | 0.83 | | 0.77 | | PHYKPL | | 1 | |
| R-HSA-73621 | | Pyrimidine catabolism | | | 1/505 | | 12/10867 | | 4.4E-01 | | 0.83 | | 0.77 | | NT5E | | 1 | |
| R-HSA-879518 | | Transport of organic anions | | | 1/505 | | 12/10867 | | 4.4E-01 | | 0.83 | | 0.77 | | SLCO2A1 | | 1 | |
| R-HSA-8851680 | | Butyrophilin (BTN) family interactions | | | 1/505 | | 12/10867 | | 4.4E-01 | | 0.83 | | 0.77 | | BTN3A3 | | 1 | |
| R-HSA-9013973 | | TICAM1-dependent activation of IRF3/IRF7 | | | 1/505 | | 12/10867 | | 4.4E-01 | | 0.83 | | 0.77 | | IRF7 | | 1 | |
| R-HSA-9027276 | | Erythropoietin activates Phosphoinositide-3-kinase (PI3K) | | | 1/505 | | 12/10867 | | 4.4E-01 | | 0.83 | | 0.77 | | PIK3R1 | | 1 | |
| R-HSA-9664420 | | Killing mechanisms | | | 1/505 | | 12/10867 | | 4.4E-01 | | 0.83 | | 0.77 | | WNT5A | | 1 | |
| R-HSA-9673324 | | WNT5:FZD7-mediated leishmania damping | | | 1/505 | | 12/10867 | | 4.4E-01 | | 0.83 | | 0.77 | | WNT5A | | 1 | |
| R-HSA-9673767 | | Signaling by PDGFRA transmembrane. juxtamembrane and kinase domain mutants | | | 1/505 | | 12/10867 | | 4.4E-01 | | 0.83 | | 0.77 | | PIK3R1 | | 1 | |
| R-HSA-9673770 | | Signaling by PDGFRA extracellular domain mutants | | | 1/505 | | 12/10867 | | 4.4E-01 | | 0.83 | | 0.77 | | PIK3R1 | | 1 | |
| R-HSA-9700645 | | ALK mutants bind TKIs | | | 1/505 | | 12/10867 | | 4.4E-01 | | 0.83 | | 0.77 | | CLTC | | 1 | |
| R-HSA-156842 | | Eukaryotic Translation Elongation | | | 5/505 | | 93/10867 | | 4.4E-01 | | 0.83 | | 0.77 | | RPL32/RPL36AL/RPS14/RPS21/RPS9 | | 5 | |
| R-HSA-2408557 | | Selenocysteine synthesis | | | 5/505 | | 93/10867 | | 4.4E-01 | | 0.83 | | 0.77 | | RPL32/RPL36AL/RPS14/RPS21/RPS9 | | 5 | |
| R-HSA-442755 | | Activation of NMDA receptors and postsynaptic events | | | 5/505 | | 93/10867 | | 4.4E-01 | | 0.83 | | 0.77 | | CAMK4/ERBB4/KPNA2/PRKAA1/PRKAA2 | | 5 | |
| R-HSA-72764 | | Eukaryotic Translation Termination | | | 5/505 | | 93/10867 | | 4.4E-01 | | 0.83 | | 0.77 | | RPL32/RPL36AL/RPS14/RPS21/RPS9 | | 5 | |
| R-HSA-2980766 | | Nuclear Envelope Breakdown | | | 3/505 | | 52/10867 | | 4.4E-01 | | 0.83 | | 0.77 | | CCNB2/RANBP2/VRK1 | | 3 | |
| R-HSA-72165 | | mRNA Splicing - Minor Pathway | | | 3/505 | | 52/10867 | | 4.4E-01 | | 0.83 | | 0.77 | | GTF2F1/SNRNP200/SRSF1 | | 3 | |
| R-HSA-9711097 | | Cellular response to starvation | | | 8/505 | | 156/10867 | | 4.4E-01 | | 0.83 | | 0.77 | | FNIP1/FNIP2/RPL32/RPL36AL/RPS14/RPS21/RPS9/SH3BP4 | | 8 | |
| R-HSA-2173796 | | SMAD2/SMAD3:SMAD4 heterotrimer regulates transcription | | | 2/505 | | 32/10867 | | 4.4E-01 | | 0.83 | | 0.77 | | RNF111/SP1 | | 2 | |
| R-HSA-397795 | | G-protein beta:gamma signalling | | | 2/505 | | 32/10867 | | 4.4E-01 | | 0.83 | | 0.77 | | BTK/GNG12 | | 2 | |
| R-HSA-456926 | | Thrombin signalling through proteinase activated receptors (PARs) | | | 2/505 | | 32/10867 | | 4.4E-01 | | 0.83 | | 0.77 | | GNAQ/GNG12 | | 2 | |
| R-HSA-167172 | | Transcription of the HIV genome | | | 4/505 | | 73/10867 | | 4.4E-01 | | 0.83 | | 0.77 | | CCNH/GTF2F1/NELFB/SUPT16H | | 4 | |
| R-HSA-5654738 | | Signaling by FGFR2 | | | 4/505 | | 73/10867 | | 4.4E-01 | | 0.83 | | 0.77 | | FGF18/FRS2/GTF2F1/PIK3R1 | | 4 | |
| R-HSA-112316 | | Neuronal System | | | 20/505 | | 410/10867 | | 4.4E-01 | | 0.83 | | 0.77 | | ABAT/ARL6IP5/CACNB4/CAMK4/ERBB4/GNAI2/GNG12/KCNK10/KPNA2/NLGN2/NPTN/NRXN3/PRKAA1/PRKAA2/RAB3A/SLC1A1/SLC38A1/SLC38A2/SLC6A12/VAMP2 | | 20 | |
| R-HSA-168164 | | Toll Like Receptor 3 (TLR3) Cascade | | | 5/505 | | 94/10867 | | 4.4E-01 | | 0.83 | | 0.77 | | DUSP4/IRF7/MEF2C/RIPK3/TAB1 | | 5 | |
| R-HSA-913531 | | Interferon Signaling | | | 10/505 | | 199/10867 | | 4.5E-01 | | 0.83 | | 0.77 | | HLA-DPB1/IFNGR1/IRF7/JAK1/KPNA1/KPNA2/NEDD4/PTPN6/RANBP2/VCAM1 | | 10 | |
| R-HSA-1660499 | | Synthesis of PIPs at the plasma membrane | | | 3/505 | | 53/10867 | | 4.5E-01 | | 0.83 | | 0.77 | | INPP4B/PIK3R1/RAB14 | | 3 | |
| R-HSA-6807505 | | RNA polymerase II transcribes snRNA genes | | | 4/505 | | 74/10867 | | 4.5E-01 | | 0.83 | | 0.77 | | GTF2F1/POU2F2/SNAPC4/SP1 | | 4 | |
| R-HSA-373080 | | Class B/2 (Secretin family receptors) | | | 5/505 | | 95/10867 | | 4.5E-01 | | 0.83 | | 0.77 | | CALCRL/GNG12/RAMP2/WNT5A/WNT7B | | 5 | |
| R-HSA-975956 | | Nonsense Mediated Decay (NMD) independent of the Exon Junction Complex (EJC) | | | 5/505 | | 95/10867 | | 4.5E-01 | | 0.83 | | 0.77 | | RPL32/RPL36AL/RPS14/RPS21/RPS9 | | 5 | |
| R-HSA-168274 | | Export of Viral Ribonucleoproteins from Nucleus | | | 2/505 | | 33/10867 | | 4.6E-01 | | 0.83 | | 0.77 | | RANBP2/XPO1 | | 2 | |
| R-HSA-180910 | | Vpr-mediated nuclear import of PICs | | | 2/505 | | 33/10867 | | 4.6E-01 | | 0.83 | | 0.77 | | KPNA1/RANBP2 | | 2 | |
| R-HSA-1839126 | | FGFR2 mutant receptor activation | | | 2/505 | | 33/10867 | | 4.6E-01 | | 0.83 | | 0.77 | | FGF18/GTF2F1 | | 2 | |
| R-HSA-2559585 | | Oncogene Induced Senescence | | | 2/505 | | 33/10867 | | 4.6E-01 | | 0.83 | | 0.77 | | ETS1/SP1 | | 2 | |
| R-HSA-2871809 | | FCERI mediated Ca+2 mobilization | | | 2/505 | | 33/10867 | | 4.6E-01 | | 0.83 | | 0.77 | | BTK/ITPR1 | | 2 | |
| R-HSA-1358803 | | Downregulation of ERBB2:ERBB3 signaling | | | 1/505 | | 13/10867 | | 4.6E-01 | | 0.83 | | 0.77 | | ERBB3 | | 1 | |
| R-HSA-140342 | | Apoptosis induced DNA fragmentation | | | 1/505 | | 13/10867 | | 4.6E-01 | | 0.83 | | 0.77 | | KPNA1 | | 1 | |
| R-HSA-1502540 | | Signaling by Activin | | | 1/505 | | 13/10867 | | 4.6E-01 | | 0.83 | | 0.77 | | INHBA | | 1 | |
| R-HSA-174430 | | Telomere C-strand synthesis initiation | | | 1/505 | | 13/10867 | | 4.6E-01 | | 0.83 | | 0.77 | | TERF2IP | | 1 | |
| R-HSA-190239 | | FGFR3 ligand binding and activation | | | 1/505 | | 13/10867 | | 4.6E-01 | | 0.83 | | 0.77 | | FGF18 | | 1 | |
| R-HSA-190372 | | FGFR3c ligand binding and activation | | | 1/505 | | 13/10867 | | 4.6E-01 | | 0.83 | | 0.77 | | FGF18 | | 1 | |
| R-HSA-190375 | | FGFR2c ligand binding and activation | | | 1/505 | | 13/10867 | | 4.6E-01 | | 0.83 | | 0.77 | | FGF18 | | 1 | |
| R-HSA-202670 | | ERKs are inactivated | | | 1/505 | | 13/10867 | | 4.6E-01 | | 0.83 | | 0.77 | | DUSP4 | | 1 | |
| R-HSA-209543 | | p75NTR recruits signalling complexes | | | 1/505 | | 13/10867 | | 4.6E-01 | | 0.83 | | 0.77 | | SQSTM1 | | 1 | |
| R-HSA-209560 | | NF-kB is activated and signals survival | | | 1/505 | | 13/10867 | | 4.6E-01 | | 0.83 | | 0.77 | | SQSTM1 | | 1 | |
| R-HSA-2892247 | | POU5F1 (OCT4). SOX2. NANOG activate genes related to proliferation | | | 1/505 | | 13/10867 | | 4.6E-01 | | 0.83 | | 0.77 | | SALL1 | | 1 | |
| R-HSA-3270619 | | IRF3-mediated induction of type I IFN | | | 1/505 | | 13/10867 | | 4.6E-01 | | 0.83 | | 0.77 | | IFI16 | | 1 | |
| R-HSA-400511 | | Synthesis. secretion. and inactivation of Glucose-dependent Insulinotropic Polypeptide (GIP) | | | 1/505 | | 13/10867 | | 4.6E-01 | | 0.83 | | 0.77 | | PCSK1 | | 1 | |
| R-HSA-425561 | | Sodium/Calcium exchangers | | | 1/505 | | 13/10867 | | 4.6E-01 | | 0.83 | | 0.77 | | SLC24A2 | | 1 | |
| R-HSA-5607763 | | CLEC7A (Dectin-1) induces NFAT activation | | | 1/505 | | 13/10867 | | 4.6E-01 | | 0.83 | | 0.77 | | ITPR1 | | 1 | |
| R-HSA-5654227 | | Phospholipase C-mediated cascade | | |  | |  | | 4.6E-01 | | 0.83 | | 0.77 | |  | |  | |
| R-HSA-5658623 | | FGFRL1 modulation of FGFR1 signaling | | | 1/505 | | 13/10867 | | 4.6E-01 | | 0.83 | | 0.77 | | FGF18 | | 1 | |
| R-HSA-75205 | | Dissolution of Fibrin Clot | | | 1/505 | | 13/10867 | | 4.6E-01 | | 0.83 | | 0.77 | | PLAU | | 1 | |
| R-HSA-9637687 | | Suppression of phagosomal maturation | | | 1/505 | | 13/10867 | | 4.6E-01 | | 0.83 | | 0.77 | | KPNA1 | | 1 | |
| R-HSA-74751 | | Insulin receptor signalling cascade | | | 3/505 | | 54/10867 | | 4.6E-01 | | 0.83 | | 0.77 | | FGF18/FRS2/PIK3R1 | | 3 | |
| R-HSA-8848021 | | Signaling by PTK6 | | | 3/505 | | 54/10867 | | 4.6E-01 | | 0.83 | | 0.77 | | ERBB3/ERBB4/PXN | | 3 | |
| R-HSA-9006927 | | Signaling by Non-Receptor Tyrosine Kinases | | | 3/505 | | 54/10867 | | 4.6E-01 | | 0.83 | | 0.77 | | ERBB3/ERBB4/PXN | | 3 | |
| R-HSA-9648025 | | EML4 and NUDC in mitotic spindle formation | | | 6/505 | | 117/10867 | | 4.6E-01 | | 0.83 | | 0.77 | | CENPA/CENPQ/CLIP1/RANBP2/SGO1/XPO1 | | 6 | |
| R-HSA-5668914 | | Diseases of metabolism | | | 12/505 | | 245/10867 | | 4.7E-01 | | 0.83 | | 0.77 | | ACACA/ALG13/CSF2RA/FDXR/HYAL1/IDH1/SEMA5A/SLC35D1/THBS1/THSD4/THSD7A/VCAN | | 12 | |
| R-HSA-162599 | | Late Phase of HIV Life Cycle | | | 7/505 | | 139/10867 | | 4.7E-01 | | 0.83 | | 0.77 | | CCNH/CHMP3/GTF2F1/NELFB/RANBP2/SUPT16H/XPO1 | | 7 | |
| R-HSA-2408522 | | Selenoamino acid metabolism | | | 6/505 | | 118/10867 | | 4.7E-01 | | 0.83 | | 0.77 | | IARS1/RPL32/RPL36AL/RPS14/RPS21/RPS9 | | 6 | |
| R-HSA-193704 | | p75 NTR receptor-mediated signalling | | | 5/505 | | 97/10867 | | 4.7E-01 | | 0.83 | | 0.77 | | ARHGDIA/ARHGEF12/ARHGEF3/FGD2/SQSTM1 | | 5 | |
| R-HSA-204998 | | Cell death signalling via NRAGE. NRIF and NADE | | | 4/505 | | 76/10867 | | 4.7E-01 | | 0.83 | | 0.77 | | ARHGEF12/ARHGEF3/FGD2/SQSTM1 | | 4 | |
| R-HSA-165054 | | Rev-mediated nuclear export of HIV RNA | | | 2/505 | | 34/10867 | | 4.7E-01 | | 0.83 | | 0.77 | | RANBP2/XPO1 | | 2 | |
| R-HSA-202433 | | Generation of second messenger molecules | | | 2/505 | | 34/10867 | | 4.7E-01 | | 0.83 | | 0.77 | | HLA-DPB1/LCK | | 2 | |
| R-HSA-5579029 | | Metabolic disorders of biological oxidation enzymes | | | 2/505 | | 34/10867 | | 4.7E-01 | | 0.83 | | 0.77 | | FDXR/SLC35D1 | | 2 | |
| R-HSA-5654727 | | Negative regulation of FGFR2 signaling | | | 2/505 | | 34/10867 | | 4.7E-01 | | 0.83 | | 0.77 | | FGF18/FRS2 | | 2 | |
| R-HSA-9639288 | | Amino acids regulate mTORC1 | | | 3/505 | | 55/10867 | | 4.7E-01 | | 0.83 | | 0.77 | | FNIP1/FNIP2/SH3BP4 | | 3 | |
| R-HSA-1474228 | | Degradation of the extracellular matrix | | | 7/505 | | 140/10867 | | 4.8E-01 | | 0.83 | | 0.77 | | ADAM10/BSG/COL17A1/COL1A1/COL5A1/HTRA1/PHYKPL | | 7 | |
| R-HSA-5620912 | | Anchoring of the basal body to the plasma membrane | | | 5/505 | | 98/10867 | | 4.8E-01 | | 0.83 | | 0.77 | | CCP110/HSP90AA1/RAB11A/RAB3IP/SEPTIN2 | | 5 | |
| R-HSA-8852276 | | The role of GTSE1 in G2/M progression after G2 checkpoint | | | 4/505 | | 77/10867 | | 4.8E-01 | | 0.83 | | 0.77 | | CCNB2/CDKN1A/HSP90AA1/PSMC3 | | 4 | |
| R-HSA-73886 | | Chromosome Maintenance | | | 7/505 | | 141/10867 | | 4.8E-01 | | 0.83 | | 0.77 | | BLM/CENPA/CENPQ/PCNA/POLD4/RFC5/TERF2IP | | 7 | |
| R-HSA-73887 | | Death Receptor Signalling | | | 7/505 | | 141/10867 | | 4.8E-01 | | 0.83 | | 0.77 | | ARHGDIA/ARHGEF12/ARHGEF3/FGD2/RACK1/SQSTM1/TAB1 | | 7 | |
| R-HSA-1250347 | | SHC1 events in ERBB4 signaling | | | 1/505 | | 14/10867 | | 4.9E-01 | | 0.83 | | 0.77 | | ERBB4 | | 1 | |
| R-HSA-162594 | | Early Phase of HIV Life Cycle | | | 1/505 | | 14/10867 | | 4.9E-01 | | 0.83 | | 0.77 | | KPNA1 | | 1 | |
| R-HSA-162658 | | Golgi Cisternae Pericentriolar Stack Reorganization | | | 1/505 | | 14/10867 | | 4.9E-01 | | 0.83 | | 0.77 | | CCNB2 | | 1 | |
| R-HSA-166786 | | Creation of C4 and C2 activators | | | 1/505 | | 14/10867 | | 4.9E-01 | | 0.83 | | 0.77 | | C1QA | | 1 | |
| R-HSA-169893 | | Prolonged ERK activation events | | | 1/505 | | 14/10867 | | 4.9E-01 | | 0.83 | | 0.77 | | FRS2 | | 1 | |
| R-HSA-170670 | | Adenylate cyclase inhibitory pathway | | | 1/505 | | 14/10867 | | 4.9E-01 | | 0.83 | | 0.77 | | GNAI2 | | 1 | |
| R-HSA-177504 | | Retrograde neurotrophin signalling | | | 1/505 | | 14/10867 | | 4.9E-01 | | 0.83 | | 0.77 | | CLTC | | 1 | |
| R-HSA-1855183 | | Synthesis of IP2. IP. and Ins in the cytosol | | | 1/505 | | 14/10867 | | 4.9E-01 | | 0.83 | | 0.77 | | INPP4B | | 1 | |
| R-HSA-190322 | | FGFR4 ligand binding and activation | | | 1/505 | | 14/10867 | | 4.9E-01 | | 0.83 | | 0.77 | | FGF18 | | 1 | |
| R-HSA-3371568 | | Attenuation phase | | | 1/505 | | 14/10867 | | 4.9E-01 | | 0.83 | | 0.77 | | HSP90AA1 | | 1 | |
| R-HSA-418359 | | Reduction of cytosolic Ca++ levels | | | 1/505 | | 14/10867 | | 4.9E-01 | | 0.83 | | 0.77 | | ATP2A2 | | 1 | |
| R-HSA-428540 | | Activation of RAC1 | | | 1/505 | | 14/10867 | | 4.9E-01 | | 0.83 | | 0.77 | | SLIT2 | | 1 | |
| R-HSA-6803205 | | TP53 regulates transcription of several additional cell death genes whose specific roles in p53-dependent apoptosis remain uncertain | | | 1/505 | | 14/10867 | | 4.9E-01 | | 0.83 | | 0.77 | | PERP | | 1 | |
| R-HSA-8849471 | | PTK6 Regulates RHO GTPases. RAS GTPase and MAP kinases | | | 1/505 | | 14/10867 | | 4.9E-01 | | 0.83 | | 0.77 | | PXN | | 1 | |
| R-HSA-9005891 | | Loss of function of MECP2 in Rett syndrome | | | 1/505 | | 14/10867 | | 4.9E-01 | | 0.83 | | 0.77 | | CAMK4 | | 1 | |
| R-HSA-9005895 | | Pervasive developmental disorders | | | 1/505 | | 14/10867 | | 4.9E-01 | | 0.83 | | 0.77 | | CAMK4 | | 1 | |
| R-HSA-9675151 | | Disorders of Developmental Biology | | | 1/505 | | 14/10867 | | 4.9E-01 | | 0.83 | | 0.77 | | CAMK4 | | 1 | |
| R-HSA-9679504 | | Translation of Replicase and Assembly of the Replication Transcription Complex | | | 1/505 | | 14/10867 | | 4.9E-01 | | 0.83 | | 0.77 | | CHMP3 | | 1 | |
| R-HSA-9694676 | | Translation of Replicase and Assembly of the Replication Transcription Complex | | | 1/505 | | 14/10867 | | 4.9E-01 | | 0.83 | | 0.77 | | CHMP3 | | 1 | |
| R-HSA-9697154 | | Disorders of Nervous System Development | | | 1/505 | | 14/10867 | | 4.9E-01 | | 0.83 | | 0.77 | | CAMK4 | | 1 | |
| R-HSA-975110 | | TRAF6 mediated IRF7 activation in TLR7/8 or 9 signaling | | | 1/505 | | 14/10867 | | 4.9E-01 | | 0.83 | | 0.77 | | IRF7 | | 1 | |
| R-HSA-202403 | | TCR signaling | | | 6/505 | | 120/10867 | | 4.9E-01 | | 0.83 | | 0.77 | | CSK/HLA-DPB1/LCK/PAG1/PIK3R1/PSMC3 | | 6 | |
| R-HSA-3301854 | | Nuclear Pore Complex (NPC) Disassembly | | | 2/505 | | 35/10867 | | 4.9E-01 | | 0.83 | | 0.77 | | CCNB2/RANBP2 | | 2 | |
| R-HSA-532668 | | N-glycan trimming in the ER and Calnexin/Calreticulin cycle | | | 2/505 | | 35/10867 | | 4.9E-01 | | 0.83 | | 0.77 | | RNF103/SEL1L | | 2 | |
| R-HSA-8953750 | | Transcriptional Regulation by E2F6 | | | 2/505 | | 35/10867 | | 4.9E-01 | | 0.83 | | 0.77 | | CBX3/RRM2 | | 2 | |
| R-HSA-9615710 | | Late endosomal microautophagy | | | 2/505 | | 35/10867 | | 4.9E-01 | | 0.83 | | 0.77 | | CHMP3/VIM | | 2 | |
| R-HSA-948021 | | Transport to the Golgi and subsequent modification | | | 9/505 | | 185/10867 | | 4.9E-01 | | 0.84 | | 0.77 | | ARCN1/MIA3/SEC16A/SEC23A/SEC24A/SEC24B/SEC24C/SEC24D/TRAPPC1 | | 9 | |
| R-HSA-8878166 | | Transcriptional regulation by RUNX2 | | | 6/505 | | 121/10867 | | 5.0E-01 | | 0.84 | | 0.77 | | BMP2/CDKN1A/COL1A1/GSK3B/PSMC3/RUNX1 | | 6 | |
| R-HSA-5218859 | | Regulated Necrosis | | | 3/505 | | 57/10867 | | 5.0E-01 | | 0.84 | | 0.77 | | CHMP3/HSP90AA1/RIPK3 | | 3 | |
| R-HSA-425407 | | SLC-mediated transmembrane transport | | | 12/505 | | 251/10867 | | 5.0E-01 | | 0.84 | | 0.77 | | BSG/RUNX1/SLC1A1/SLC24A2/SLC35D1/SLC38A1/SLC38A2/SLC39A6/SLC41A2/SLC5A6/SLC6A12/SLCO2A1 | | 12 | |
| R-HSA-176033 | | Interactions of Vpr with host cellular proteins | | | 2/505 | | 36/10867 | | 5.0E-01 | | 0.84 | | 0.77 | | KPNA1/RANBP2 | | 2 | |
| R-HSA-177243 | | Interactions of Rev with host cellular proteins | | | 2/505 | | 36/10867 | | 5.0E-01 | | 0.84 | | 0.77 | | RANBP2/XPO1 | | 2 | |
| R-HSA-72689 | | Formation of a pool of free 40S subunits | | | 5/505 | | 101/10867 | | 5.1E-01 | | 0.84 | | 0.77 | | RPL32/RPL36AL/RPS14/RPS21/RPS9 | | 5 | |
| R-HSA-9633012 | | Response of EIF2AK4 (GCN2) to amino acid deficiency | | | 5/505 | | 101/10867 | | 5.1E-01 | | 0.84 | | 0.77 | | RPL32/RPL36AL/RPS14/RPS21/RPS9 | | 5 | |
| R-HSA-166658 | | Complement cascade | | | 3/505 | | 58/10867 | | 5.1E-01 | | 0.84 | | 0.77 | | C1QA/CFD/PROS1 | | 3 | |
| R-HSA-186797 | | Signaling by PDGF | | | 3/505 | | 58/10867 | | 5.1E-01 | | 0.84 | | 0.77 | | COL5A1/PIK3R1/THBS1 | | 3 | |
| R-HSA-72187 | | mRNA 3'-end processing | | | 3/505 | | 58/10867 | | 5.1E-01 | | 0.84 | | 0.77 | | CSTF2T/SRSF1/ZC3H11A | | 3 | |
| R-HSA-72649 | | Translation initiation complex formation | | | 3/505 | | 58/10867 | | 5.1E-01 | | 0.84 | | 0.77 | | RPS14/RPS21/RPS9 | | 3 | |
| R-HSA-72702 | | Ribosomal scanning and start codon recognition | | | 3/505 | | 58/10867 | | 5.1E-01 | | 0.84 | | 0.77 | | RPS14/RPS21/RPS9 | | 3 | |
| R-HSA-211935 | | Fatty acids | | | 1/505 | | 15/10867 | | 5.1E-01 | | 0.84 | | 0.77 | | CYP4F12 | | 1 | |
| R-HSA-2214320 | | Anchoring fibril formation | | | 1/505 | | 15/10867 | | 5.1E-01 | | 0.84 | | 0.77 | | COL1A1 | | 1 | |
| R-HSA-2691230 | | Signaling by NOTCH1 HD Domain Mutants in Cancer | | | 1/505 | | 15/10867 | | 5.1E-01 | | 0.84 | | 0.77 | | ADAM10 | | 1 | |
| R-HSA-2691232 | | Constitutive Signaling by NOTCH1 HD Domain Mutants | | | 1/505 | | 15/10867 | | 5.1E-01 | | 0.84 | | 0.77 | | ADAM10 | | 1 | |
| R-HSA-354194 | | GRB2:SOS provides linkage to MAPK signaling for Integrins | | | 1/505 | | 15/10867 | | 5.1E-01 | | 0.84 | | 0.77 | | TLN1 | | 1 | |
| R-HSA-372708 | | p130Cas linkage to MAPK signaling for integrins | | | 1/505 | | 15/10867 | | 5.1E-01 | | 0.84 | | 0.77 | | TLN1 | | 1 | |
| R-HSA-430039 | | mRNA decay by 5' to 3' exoribonuclease | | | 1/505 | | 15/10867 | | 5.1E-01 | | 0.84 | | 0.77 | | DDX6 | | 1 | |
| R-HSA-549132 | | Organic cation/anion/zwitterion transport | | | 1/505 | | 15/10867 | | 5.1E-01 | | 0.84 | | 0.77 | | RUNX1 | | 1 | |
| R-HSA-5654228 | | Phospholipase C-mediated cascade | | |  | |  | | 5.1E-01 | | 0.84 | | 0.77 | |  | |  | |
| R-HSA-8875360 | | InlB-mediated entry of Listeria monocytogenes into host cell | | | 1/505 | | 15/10867 | | 5.1E-01 | | 0.84 | | 0.77 | | SH3KBP1 | | 1 | |
| R-HSA-918233 | | TRAF3-dependent IRF activation pathway | | | 1/505 | | 15/10867 | | 5.1E-01 | | 0.84 | | 0.77 | | IRF7 | | 1 | |
| R-HSA-9648895 | | Response of EIF2AK1 (HRI) to heme deficiency | | | 1/505 | | 15/10867 | | 5.1E-01 | | 0.84 | | 0.77 | | EIF2AK1 | | 1 | |
| R-HSA-9706369 | | Negative regulation of FLT3 | | | 1/505 | | 15/10867 | | 5.1E-01 | | 0.84 | | 0.77 | | CSK | | 1 | |
| R-HSA-2559586 | | DNA Damage/Telomere Stress Induced Senescence | | | 4/505 | | 80/10867 | | 5.1E-01 | | 0.84 | | 0.77 | | CCNE2/CDKN1A/RAD50/TERF2IP | | 4 | |
| R-HSA-111933 | | Calmodulin induced events | | | 2/505 | | 37/10867 | | 5.2E-01 | | 0.84 | | 0.77 | | CAMK4/KPNA2 | | 2 | |
| R-HSA-111997 | | CaM pathway | | | 2/505 | | 37/10867 | | 5.2E-01 | | 0.84 | | 0.77 | | CAMK4/KPNA2 | | 2 | |
| R-HSA-8979227 | | Triglyceride metabolism | | | 2/505 | | 37/10867 | | 5.2E-01 | | 0.84 | | 0.77 | | CAV1/LIPE | | 2 | |
| R-HSA-193648 | | NRAGE signals death through JNK | | | 3/505 | | 59/10867 | | 5.2E-01 | | 0.84 | | 0.77 | | ARHGEF12/ARHGEF3/FGD2 | | 3 | |
| R-HSA-2142753 | | Arachidonic acid metabolism | | | 3/505 | | 59/10867 | | 5.2E-01 | | 0.84 | | 0.77 | | HPGD/PLA2G4A/PTGES2 | | 3 | |
| R-HSA-380108 | | Chemokine receptors bind chemokines | | | 3/505 | | 59/10867 | | 5.2E-01 | | 0.84 | | 0.77 | | ACKR3/CCRL2/CXCL8 | | 3 | |
| R-HSA-72662 | | Activation of the mRNA upon binding of the cap-binding complex and eIFs. and subsequent binding to 43S | | | 3/505 | | 59/10867 | | 5.2E-01 | | 0.84 | | 0.77 | | RPS14/RPS21/RPS9 | | 3 | |
| R-HSA-8866654 | | E3 ubiquitin ligases ubiquitinate target proteins | | | 3/505 | | 59/10867 | | 5.2E-01 | | 0.84 | | 0.77 | | PCNA/RNF20/TMEM129 | | 3 | |
| R-HSA-9663891 | | Selective autophagy | | | 4/505 | | 81/10867 | | 5.2E-01 | | 0.84 | | 0.77 | | HSP90AA1/PRKAA2/SQSTM1/VIM | | 4 | |
| R-HSA-9020702 | | Interleukin-1 signaling | | | 5/505 | | 103/10867 | | 5.3E-01 | | 0.84 | | 0.77 | | IL1R1/MAP3K3/PSMC3/SQSTM1/TAB1 | | 5 | |
| R-HSA-1483257 | | Phospholipid metabolism | | | 10/505 | | 212/10867 | | 5.3E-01 | | 0.84 | | 0.77 | | GPD1L/INPP4B/INPP5E/LIPH/MIGA2/PIK3R1/PLA2G2A/PLA2G4A/RAB14/TNFAIP8 | | 10 | |
| R-HSA-187577 | | SCF(Skp2)-mediated degradation of p27/p21 | | | 3/505 | | 60/10867 | | 5.3E-01 | | 0.84 | | 0.77 | | CCNE2/CDKN1A/PSMC3 | | 3 | |
| R-HSA-5610783 | | Degradation of GLI2 by the proteasome | | | 3/505 | | 60/10867 | | 5.3E-01 | | 0.84 | | 0.77 | | CSNK1A1/GSK3B/PSMC3 | | 3 | |
| R-HSA-5610785 | | GLI3 is processed to GLI3R by the proteasome | | | 3/505 | | 60/10867 | | 5.3E-01 | | 0.84 | | 0.77 | | CSNK1A1/GSK3B/PSMC3 | | 3 | |
| R-HSA-977443 | | GABA receptor activation | | | 3/505 | | 60/10867 | | 5.3E-01 | | 0.84 | | 0.77 | | GNAI2/GNG12/NPTN | | 3 | |
| R-HSA-5655302 | | Signaling by FGFR1 in disease | | | 2/505 | | 38/10867 | | 5.3E-01 | | 0.84 | | 0.77 | | FRS2/PIK3R1 | | 2 | |
| R-HSA-5696394 | | DNA Damage Recognition in GG-NER | | | 2/505 | | 38/10867 | | 5.3E-01 | | 0.84 | | 0.77 | | COPS6/YY1 | | 2 | |
| R-HSA-6814122 | | Cooperation of PDCL (PhLP1) and TRiC/CCT in G-protein beta folding | | | 2/505 | | 38/10867 | | 5.3E-01 | | 0.84 | | 0.77 | | GNAQ/GNG12 | | 2 | |
| R-HSA-8939243 | | RUNX1 interacts with co-factors whose precise effect on RUNX1 targets is not known | | | 2/505 | | 38/10867 | | 5.3E-01 | | 0.84 | | 0.77 | | RUNX1/SMARCC1 | | 2 | |
| R-HSA-1362300 | | Transcription of E2F targets under negative control by p107 (RBL1) and p130 (RBL2) in complex with HDAC1 | | | 1/505 | | 16/10867 | | 5.3E-01 | | 0.84 | | 0.77 | | MYBL2 | | 1 | |
| R-HSA-139853 | | Elevation of cytosolic Ca2+ levels | | | 1/505 | | 16/10867 | | 5.3E-01 | | 0.84 | | 0.77 | | ITPR1 | | 1 | |
| R-HSA-1660516 | | Synthesis of PIPs at the early endosome membrane | | | 1/505 | | 16/10867 | | 5.3E-01 | | 0.84 | | 0.77 | | INPP4B | | 1 | |
| R-HSA-1834941 | | STING mediated induction of host immune responses | | | 1/505 | | 16/10867 | | 5.3E-01 | | 0.84 | | 0.77 | | IFI16 | | 1 | |
| R-HSA-193639 | | p75NTR signals via NF-kB | | | 1/505 | | 16/10867 | | 5.3E-01 | | 0.84 | | 0.77 | | SQSTM1 | | 1 | |
| R-HSA-1963640 | | GRB2 events in ERBB2 signaling | | | 1/505 | | 16/10867 | | 5.3E-01 | | 0.84 | | 0.77 | | ERBB4 | | 1 | |
| R-HSA-205043 | | NRIF signals cell death from the nucleus | | | 1/505 | | 16/10867 | | 5.3E-01 | | 0.84 | | 0.77 | | SQSTM1 | | 1 | |
| R-HSA-3322077 | | Glycogen synthesis | | | 1/505 | | 16/10867 | | 5.3E-01 | | 0.84 | | 0.77 | | PGM2L1 | | 1 | |
| R-HSA-391160 | | Signal regulatory protein family interactions | | | 1/505 | | 16/10867 | | 5.3E-01 | | 0.84 | | 0.77 | | PTPN6 | | 1 | |
| R-HSA-392517 | | Rap1 signalling | | | 1/505 | | 16/10867 | | 5.3E-01 | | 0.84 | | 0.77 | | PRKG1 | | 1 | |
| R-HSA-418038 | | Nucleotide-like (purinergic) receptors | | | 1/505 | | 16/10867 | | 5.3E-01 | | 0.84 | | 0.77 | | P2RY11 | | 1 | |
| R-HSA-844456 | | The NLRP3 inflammasome | | | 1/505 | | 16/10867 | | 5.3E-01 | | 0.84 | | 0.77 | | TXNIP | | 1 | |
| R-HSA-195253 | | Degradation of beta-catenin by the destruction complex | | | 4/505 | | 83/10867 | | 5.4E-01 | | 0.85 | | 0.79 | | APC/CSNK1A1/GSK3B/PSMC3 | | 4 | |
| R-HSA-69202 | | Cyclin E associated events during G1/S transition | | | 4/505 | | 83/10867 | | 5.4E-01 | | 0.85 | | 0.79 | | CCNE2/CCNH/CDKN1A/PSMC3 | | 4 | |
| R-HSA-6790901 | | rRNA modification in the nucleus and cytosol | | | 3/505 | | 61/10867 | | 5.4E-01 | | 0.85 | | 0.79 | | FCF1/RPS14/RPS9 | | 3 | |
| R-HSA-8943724 | | Regulation of PTEN gene transcription | | | 3/505 | | 61/10867 | | 5.4E-01 | | 0.85 | | 0.79 | | MBD3/MTA3/SNAI2 | | 3 | |
| R-HSA-69306 | | DNA Replication | | | 6/505 | | 128/10867 | | 5.5E-01 | | 0.86 | | 0.79 | | CCNE2/GMNN/PCNA/POLD4/PSMC3/RFC5 | | 6 | |
| R-HSA-211945 | | Phase I - Functionalization of compounds | | | 5/505 | | 106/10867 | | 5.5E-01 | | 0.86 | | 0.79 | | ADH1A/ADH1C/ALDH1A1/CYP4F12/FDXR | | 5 | |
| R-HSA-140534 | | Caspase activation via Death Receptors in the presence of ligand | | | 1/505 | | 17/10867 | | 5.5E-01 | | 0.86 | | 0.79 | | TLR4 | | 1 | |
| R-HSA-2033519 | | Activated point mutants of FGFR2 | | | 1/505 | | 17/10867 | | 5.5E-01 | | 0.86 | | 0.79 | | FGF18 | | 1 | |
| R-HSA-3781860 | | Diseases associated with N-glycosylation of proteins | | | 1/505 | | 17/10867 | | 5.5E-01 | | 0.86 | | 0.79 | | ALG13 | | 1 | |
| R-HSA-435354 | | Zinc transporters | | | 1/505 | | 17/10867 | | 5.5E-01 | | 0.86 | | 0.79 | | SLC39A6 | | 1 | |
| R-HSA-450385 | | Butyrate Response Factor 1 (BRF1) binds and destabilizes mRNA | | | 1/505 | | 17/10867 | | 5.5E-01 | | 0.86 | | 0.79 | | ZFP36L1 | | 1 | |
| R-HSA-450513 | | Tristetraprolin (TTP. ZFP36) binds and destabilizes mRNA | | | 1/505 | | 17/10867 | | 5.5E-01 | | 0.86 | | 0.79 | | ZFP36 | | 1 | |
| R-HSA-4655427 | | SUMOylation of DNA methylation proteins | | | 1/505 | | 17/10867 | | 5.5E-01 | | 0.86 | | 0.79 | | DNMT1 | | 1 | |
| R-HSA-70221 | | Glycogen breakdown (glycogenolysis) | | | 1/505 | | 17/10867 | | 5.5E-01 | | 0.86 | | 0.79 | | PGM2L1 | | 1 | |
| R-HSA-9018677 | | Biosynthesis of DHA-derived SPMs | | | 1/505 | | 17/10867 | | 5.5E-01 | | 0.86 | | 0.79 | | HPGD | | 1 | |
| R-HSA-5576891 | | Cardiac conduction | | | 6/505 | | 129/10867 | | 5.6E-01 | | 0.86 | | 0.79 | | ASPH/ATP2A2/DMPK/ITPR1/KCNK10/SCN1B | | 6 | |
| R-HSA-8853659 | | RET signaling | | | 2/505 | | 40/10867 | | 5.6E-01 | | 0.86 | | 0.80 | | FRS2/PIK3R1 | | 2 | |
| R-HSA-168142 | | Toll Like Receptor 10 (TLR10) Cascade | | | 4/505 | | 85/10867 | | 5.6E-01 | | 0.86 | | 0.80 | | DUSP4/MAP3K1/MEF2C/TAB1 | | 4 | |
| R-HSA-168176 | | Toll Like Receptor 5 (TLR5) Cascade | | | 4/505 | | 85/10867 | | 5.6E-01 | | 0.86 | | 0.80 | | DUSP4/MAP3K1/MEF2C/TAB1 | | 4 | |
| R-HSA-69656 | | Cyclin A:Cdk2-associated events at S phase entry | | | 4/505 | | 85/10867 | | 5.6E-01 | | 0.86 | | 0.80 | | CCNE2/CCNH/CDKN1A/PSMC3 | | 4 | |
| R-HSA-975871 | | MyD88 cascade initiated on plasma membrane | | | 4/505 | | 85/10867 | | 5.6E-01 | | 0.86 | | 0.80 | | DUSP4/MAP3K1/MEF2C/TAB1 | | 4 | |
| R-HSA-425393 | | Transport of inorganic cations/anions and amino acids/oligopeptides | | | 5/505 | | 108/10867 | | 5.7E-01 | | 0.87 | | 0.80 | | SLC1A1/SLC24A2/SLC38A1/SLC38A2/SLC6A12 | | 5 | |
| R-HSA-2559583 | | Cellular Senescence | | | 9/505 | | 197/10867 | | 5.7E-01 | | 0.87 | | 0.80 | | CCNE2/CDKN1A/CXCL8/ETS1/IGFBP7/MAP3K5/RAD50/SP1/TERF2IP | | 9 | |
| R-HSA-1660514 | | Synthesis of PIPs at the Golgi membrane | | | 1/505 | | 18/10867 | | 5.8E-01 | | 0.87 | | 0.81 | | INPP5E | | 1 | |
| R-HSA-1839117 | | Signaling by cytosolic FGFR1 fusion mutants | | | 1/505 | | 18/10867 | | 5.8E-01 | | 0.87 | | 0.81 | | PIK3R1 | | 1 | |
| R-HSA-210993 | | Tie2 Signaling | | | 1/505 | | 18/10867 | | 5.8E-01 | | 0.87 | | 0.81 | | PIK3R1 | | 1 | |
| R-HSA-2243919 | | Crosslinking of collagen fibrils | | | 1/505 | | 18/10867 | | 5.8E-01 | | 0.87 | | 0.81 | | COL1A1 | | 1 | |
| R-HSA-5654221 | | Phospholipase C-mediated cascade | | |  | |  | | 5.8E-01 | | 0.87 | | 0.81 | |  | |  | |
| R-HSA-5654704 | | SHC-mediated cascade:FGFR3 | | | 1/505 | | 18/10867 | | 5.8E-01 | | 0.87 | | 0.81 | | FGF18 | | 1 | |
| R-HSA-74259 | | Purine catabolism | | | 1/505 | | 18/10867 | | 5.8E-01 | | 0.87 | | 0.81 | | NT5E | | 1 | |
| R-HSA-450294 | | MAP kinase activation | | | 3/505 | | 64/10867 | | 5.8E-01 | | 0.87 | | 0.81 | | DUSP4/MEF2C/TAB1 | | 3 | |
| R-HSA-190236 | | Signaling by FGFR | | | 4/505 | | 87/10867 | | 5.8E-01 | | 0.88 | | 0.81 | | FGF18/FRS2/GTF2F1/PIK3R1 | | 4 | |
| R-HSA-375280 | | Amine ligand-binding receptors | | | 2/505 | | 42/10867 | | 5.9E-01 | | 0.88 | | 0.81 | | ADRA2A/HRH1 | | 2 | |
| R-HSA-3769402 | | Deactivation of the beta-catenin transactivating complex | | | 2/505 | | 42/10867 | | 5.9E-01 | | 0.88 | | 0.81 | | APC/XPO1 | | 2 | |
| R-HSA-379724 | | tRNA Aminoacylation | | | 2/505 | | 42/10867 | | 5.9E-01 | | 0.88 | | 0.81 | | FARSA/IARS1 | | 2 | |
| R-HSA-8856688 | | Golgi-to-ER retrograde transport | | | 6/505 | | 133/10867 | | 5.9E-01 | | 0.88 | | 0.81 | | ARCN1/GALNT1/KIF18B/KIF3B/PAFAH1B2/PLA2G4A | | 6 | |
| R-HSA-156827 | | L13a-mediated translational silencing of Ceruloplasmin expression | | | 5/505 | | 111/10867 | | 5.9E-01 | | 0.88 | | 0.81 | | RPL32/RPL36AL/RPS14/RPS21/RPS9 | | 5 | |
| R-HSA-1362277 | | Transcription of E2F targets under negative control by DREAM complex | | | 1/505 | | 19/10867 | | 6.0E-01 | | 0.88 | | 0.81 | | PCNA | | 1 | |
| R-HSA-197264 | | Nicotinamide salvaging | | | 1/505 | | 19/10867 | | 6.0E-01 | | 0.88 | | 0.81 | | PARP8 | | 1 | |
| R-HSA-3000480 | | Scavenging by Class A Receptors | | | 1/505 | | 19/10867 | | 6.0E-01 | | 0.88 | | 0.81 | | COL1A1 | | 1 | |
| R-HSA-392851 | | Prostacyclin signalling through prostacyclin receptor | | | 1/505 | | 19/10867 | | 6.0E-01 | | 0.88 | | 0.81 | | GNG12 | | 1 | |
| R-HSA-3928664 | | Ephrin signaling | | | 1/505 | | 19/10867 | | 6.0E-01 | | 0.88 | | 0.81 | | FYN | | 1 | |
| R-HSA-416700 | | Other semaphorin interactions | | | 1/505 | | 19/10867 | | 6.0E-01 | | 0.88 | | 0.81 | | SEMA5A | | 1 | |
| R-HSA-442660 | | Na+/Cl- dependent neurotransmitter transporters | | | 1/505 | | 19/10867 | | 6.0E-01 | | 0.88 | | 0.81 | | SLC6A12 | | 1 | |
| R-HSA-5576886 | | Phase 4 - resting membrane potential | | | 1/505 | | 19/10867 | | 6.0E-01 | | 0.88 | | 0.81 | | KCNK10 | | 1 | |
| R-HSA-77595 | | Processing of Intronless Pre-mRNAs | | | 1/505 | | 19/10867 | | 6.0E-01 | | 0.88 | | 0.81 | | CSTF2T | | 1 | |
| R-HSA-8851708 | | Signaling by FGFR2 IIIa TM | | | 1/505 | | 19/10867 | | 6.0E-01 | | 0.88 | | 0.81 | | GTF2F1 | | 1 | |
| R-HSA-9018678 | | Biosynthesis of specialized proresolving mediators (SPMs) | | | 1/505 | | 19/10867 | | 6.0E-01 | | 0.88 | | 0.81 | | HPGD | | 1 | |
| R-HSA-9725371 | | Nuclear events stimulated by ALK signaling in cancer | | | 1/505 | | 19/10867 | | 6.0E-01 | | 0.88 | | 0.81 | | CLTC | | 1 | |
| R-HSA-9660821 | | ADORA2B mediated anti-inflammatory cytokines production | | | 6/505 | | 134/10867 | | 6.0E-01 | | 0.88 | | 0.81 | | CALCRL/GNAI2/GNG12/GPR15/P2RY11/RAMP2 | | 6 | |
| R-HSA-3371556 | | Cellular response to heat stress | | | 4/505 | | 89/10867 | | 6.0E-01 | | 0.88 | | 0.81 | | GSK3B/HSP90AA1/RANBP2/SIRT1 | | 4 | |
| R-HSA-5687128 | | MAPK6/MAPK4 signaling | | | 4/505 | | 89/10867 | | 6.0E-01 | | 0.88 | | 0.81 | | FOXO1/FOXO3/PSMC3/XPO1 | | 4 | |
| R-HSA-168325 | | Viral Messenger RNA Synthesis | | | 2/505 | | 43/10867 | | 6.0E-01 | | 0.88 | | 0.81 | | GTF2F1/RANBP2 | | 2 | |
| R-HSA-432040 | | Vasopressin regulates renal water homeostasis via Aquaporins | | | 2/505 | | 43/10867 | | 6.0E-01 | | 0.88 | | 0.81 | | GNG12/RAB11A | | 2 | |
| R-HSA-5675221 | | Negative regulation of MAPK pathway | | | 2/505 | | 43/10867 | | 6.0E-01 | | 0.88 | | 0.81 | | DUSP10/DUSP4 | | 2 | |
| R-HSA-9646399 | | Aggrephagy | | | 2/505 | | 43/10867 | | 6.0E-01 | | 0.88 | | 0.81 | | HSP90AA1/VIM | | 2 | |
| R-HSA-977444 | | GABA B receptor activation | | | 2/505 | | 43/10867 | | 6.0E-01 | | 0.88 | | 0.81 | | GNAI2/GNG12 | | 2 | |
| R-HSA-991365 | | Activation of GABAB receptors | | | 2/505 | | 43/10867 | | 6.0E-01 | | 0.88 | | 0.81 | | GNAI2/GNG12 | | 2 | |
| R-HSA-1799339 | | SRP-dependent cotranslational protein targeting to membrane | | | 5/505 | | 112/10867 | | 6.0E-01 | | 0.88 | | 0.81 | | RPL32/RPL36AL/RPS14/RPS21/RPS9 | | 5 | |
| R-HSA-72706 | | GTP hydrolysis and joining of the 60S ribosomal subunit | | | 5/505 | | 112/10867 | | 6.0E-01 | | 0.88 | | 0.81 | | RPL32/RPL36AL/RPS14/RPS21/RPS9 | | 5 | |
| R-HSA-1474290 | | Collagen formation | | | 4/505 | | 90/10867 | | 6.1E-01 | | 0.88 | | 0.81 | | COL17A1/COL1A1/COL5A1/DST | | 4 | |
| R-HSA-1650814 | | Collagen biosynthesis and modifying enzymes | | | 3/505 | | 67/10867 | | 6.1E-01 | | 0.88 | | 0.81 | | COL17A1/COL1A1/COL5A1 | | 3 | |
| R-HSA-73856 | | RNA Polymerase II Transcription Termination | | | 3/505 | | 67/10867 | | 6.1E-01 | | 0.88 | | 0.81 | | CSTF2T/SRSF1/ZC3H11A | | 3 | |
| R-HSA-157579 | | Telomere Maintenance | | | 5/505 | | 113/10867 | | 6.1E-01 | | 0.88 | | 0.81 | | BLM/PCNA/POLD4/RFC5/TERF2IP | | 5 | |
| R-HSA-1632852 | | Macroautophagy | | | 6/505 | | 136/10867 | | 6.1E-01 | | 0.88 | | 0.81 | | CHMP3/HSP90AA1/PRKAA1/PRKAA2/SQSTM1/VIM | | 6 | |
| R-HSA-2173793 | | Transcriptional activity of SMAD2/SMAD3:SMAD4 heterotrimer | | | 2/505 | | 44/10867 | | 6.1E-01 | | 0.88 | | 0.81 | | RNF111/SP1 | | 2 | |
| R-HSA-5633008 | | TP53 Regulates Transcription of Cell Death Genes | | | 2/505 | | 44/10867 | | 6.1E-01 | | 0.88 | | 0.81 | | PERP/TP53INP1 | | 2 | |
| R-HSA-75893 | | TNF signaling | | | 2/505 | | 44/10867 | | 6.1E-01 | | 0.88 | | 0.81 | | RACK1/TAB1 | | 2 | |
| R-HSA-1181150 | | Signaling by NODAL | | | 1/505 | | 20/10867 | | 6.1E-01 | | 0.88 | | 0.81 | | FOXO3 | | 1 | |
| R-HSA-168927 | | TICAM1. RIP1-mediated IKK complex recruitment | | | 1/505 | | 20/10867 | | 6.1E-01 | | 0.88 | | 0.81 | | RIPK3 | | 1 | |
| R-HSA-190241 | | FGFR2 ligand binding and activation | | | 1/505 | | 20/10867 | | 6.1E-01 | | 0.88 | | 0.81 | | FGF18 | | 1 | |
| R-HSA-3232118 | | SUMOylation of transcription factors | | | 1/505 | | 20/10867 | | 6.1E-01 | | 0.88 | | 0.81 | | TFAP2A | | 1 | |
| R-HSA-3560783 | | Defective B4GALT7 causes EDS. progeroid type | | | 1/505 | | 20/10867 | | 6.1E-01 | | 0.88 | | 0.81 | | VCAN | | 1 | |
| R-HSA-3560801 | | Defective B3GAT3 causes JDSSDHD | | | 1/505 | | 20/10867 | | 6.1E-01 | | 0.88 | | 0.81 | | VCAN | | 1 | |
| R-HSA-418217 | | G beta:gamma signalling through PLC beta | | | 1/505 | | 20/10867 | | 6.1E-01 | | 0.88 | | 0.81 | | GNG12 | | 1 | |
| R-HSA-4420332 | | Defective B3GALT6 causes EDSP2 and SEMDJL1 | | | 1/505 | | 20/10867 | | 6.1E-01 | | 0.88 | | 0.81 | | VCAN | | 1 | |
| R-HSA-5625900 | | RHO GTPases activate CIT | | | 1/505 | | 20/10867 | | 6.1E-01 | | 0.88 | | 0.81 | | RHOB | | 1 | |
| R-HSA-5654719 | | SHC-mediated cascade:FGFR4 | | | 1/505 | | 20/10867 | | 6.1E-01 | | 0.88 | | 0.81 | | FGF18 | | 1 | |
| R-HSA-6803204 | | TP53 Regulates Transcription of Genes Involved in Cytochrome C Release | | | 1/505 | | 20/10867 | | 6.1E-01 | | 0.88 | | 0.81 | | TP53INP1 | | 1 | |
| R-HSA-6804115 | | TP53 regulates transcription of additional cell cycle genes whose exact role in the p53 pathway remain uncertain | | | 1/505 | | 20/10867 | | 6.1E-01 | | 0.88 | | 0.81 | | BTG2 | | 1 | |
| R-HSA-8964616 | | G beta:gamma signalling through CDC42 | | | 1/505 | | 20/10867 | | 6.1E-01 | | 0.88 | | 0.81 | | GNG12 | | 1 | |
| R-HSA-9013695 | | NOTCH4 Intracellular Domain Regulates Transcription | | | 1/505 | | 20/10867 | | 6.1E-01 | | 0.88 | | 0.81 | | FLT4 | | 1 | |
| R-HSA-9603798 | | Class I peroxisomal membrane protein import | | | 1/505 | | 20/10867 | | 6.1E-01 | | 0.88 | | 0.81 | | PEX19 | | 1 | |
| R-HSA-9671555 | | Signaling by PDGFR in disease | | | 1/505 | | 20/10867 | | 6.1E-01 | | 0.88 | | 0.81 | | PIK3R1 | | 1 | |
| R-HSA-71387 | | Metabolism of carbohydrates | | | 13/505 | | 295/10867 | | 6.2E-01 | | 0.88 | | 0.81 | | ALDH1A1/CHP1/CHST15/CHST7/GLYCTK/HMMR/HYAL1/MAN2B1/MAN2B2/PGM2L1/RANBP2/ST3GAL2/VCAN | | 13 | |
| R-HSA-72163 | | mRNA Splicing - Major Pathway | | | 8/505 | | 183/10867 | | 6.2E-01 | | 0.88 | | 0.81 | | CD2BP2/CSTF2T/DHX9/ELAVL1/FUS/GTF2F1/SNRNP200/SRSF1 | | 8 | |
| R-HSA-927802 | | Nonsense-Mediated Decay (NMD) | | | 5/505 | | 115/10867 | | 6.2E-01 | | 0.88 | | 0.81 | | RPL32/RPL36AL/RPS14/RPS21/RPS9 | | 5 | |
| R-HSA-975957 | | Nonsense Mediated Decay (NMD) enhanced by the Exon Junction Complex (EJC) | | | 5/505 | | 115/10867 | | 6.2E-01 | | 0.88 | | 0.81 | | RPL32/RPL36AL/RPS14/RPS21/RPS9 | | 5 | |
| R-HSA-3899300 | | SUMOylation of transcription cofactors | | | 2/505 | | 45/10867 | | 6.3E-01 | | 0.88 | | 0.81 | | NRIP1/TRIM28 | | 2 | |
| R-HSA-4615885 | | SUMOylation of DNA replication proteins | | | 2/505 | | 45/10867 | | 6.3E-01 | | 0.88 | | 0.81 | | PCNA/RANBP2 | | 2 | |
| R-HSA-3371453 | | Regulation of HSF1-mediated heat shock response | | | 3/505 | | 69/10867 | | 6.3E-01 | | 0.88 | | 0.81 | | GSK3B/RANBP2/SIRT1 | | 3 | |
| R-HSA-8948751 | | Regulation of PTEN stability and activity | | | 3/505 | | 69/10867 | | 6.3E-01 | | 0.88 | | 0.81 | | FRK/NEDD4/PSMC3 | | 3 | |
| R-HSA-9662360 | | Sensory processing of sound by inner hair cells of the cochlea | | | 3/505 | | 69/10867 | | 6.3E-01 | | 0.88 | | 0.81 | | MSN/RAB3A/VAMP2 | | 3 | |
| R-HSA-210745 | | Regulation of gene expression in beta cells | | | 1/505 | | 21/10867 | | 6.3E-01 | | 0.88 | | 0.81 | | FOXO1 | | 1 | |
| R-HSA-381771 | | Synthesis. secretion. and inactivation of Glucagon-like Peptide-1 (GLP-1) | | | 1/505 | | 21/10867 | | 6.3E-01 | | 0.88 | | 0.81 | | PCSK1 | | 1 | |
| R-HSA-445144 | | Signal transduction by L1 | | | 1/505 | | 21/10867 | | 6.3E-01 | | 0.88 | | 0.81 | | NRP1 | | 1 | |
| R-HSA-500657 | | Presynaptic function of Kainate receptors | | | 1/505 | | 21/10867 | | 6.3E-01 | | 0.88 | | 0.81 | | GNG12 | | 1 | |
| R-HSA-5218921 | | VEGFR2 mediated cell proliferation | | | 1/505 | | 21/10867 | | 6.3E-01 | | 0.88 | | 0.81 | | ITPR1 | | 1 | |
| R-HSA-5621575 | | CD209 (DC-SIGN) signaling | | | 1/505 | | 21/10867 | | 6.3E-01 | | 0.88 | | 0.81 | | FYN | | 1 | |
| R-HSA-5686938 | | Regulation of TLR by endogenous ligand | | | 1/505 | | 21/10867 | | 6.3E-01 | | 0.88 | | 0.81 | | TLR4 | | 1 | |
| R-HSA-622312 | | Inflammasomes | | | 1/505 | | 21/10867 | | 6.3E-01 | | 0.88 | | 0.81 | | TXNIP | | 1 | |
| R-HSA-70895 | | Branched-chain amino acid catabolism | | | 1/505 | | 21/10867 | | 6.3E-01 | | 0.88 | | 0.81 | | PPM1K | | 1 | |
| R-HSA-9008059 | | Interleukin-37 signaling | | | 1/505 | | 21/10867 | | 6.3E-01 | | 0.88 | | 0.81 | | PTPN6 | | 1 | |
| R-HSA-909733 | | Interferon alpha/beta signaling | | | 3/505 | | 70/10867 | | 6.4E-01 | | 0.89 | | 0.82 | | IRF7/JAK1/PTPN6 | | 3 | |
| R-HSA-6807070 | | PTEN Regulation | | | 6/505 | | 140/10867 | | 6.4E-01 | | 0.89 | | 0.82 | | FRK/MBD3/MTA3/NEDD4/PSMC3/SNAI2 | | 6 | |
| R-HSA-983712 | | Ion channel transport | | | 8/505 | | 186/10867 | | 6.4E-01 | | 0.89 | | 0.82 | | ANO9/ASPH/ATP2A2/ATP7A/CLCN3/RIPK3/TRPM4/TSC22D3 | | 8 | |
| R-HSA-446652 | | Interleukin-1 family signaling | | | 6/505 | | 141/10867 | | 6.5E-01 | | 0.89 | | 0.82 | | IL1R1/MAP3K3/PSMC3/PTPN6/SQSTM1/TAB1 | | 6 | |
| R-HSA-5357801 | | Programmed Cell Death | | | 9/505 | | 210/10867 | | 6.5E-01 | | 0.89 | | 0.82 | | APC/CHMP3/HSP90AA1/KPNA1/PSMC3/RIPK3/TJP1/TLR4/VIM | | 9 | |
| R-HSA-201681 | | TCF dependent signaling in response to WNT | | | 10/505 | | 233/10867 | | 6.5E-01 | | 0.89 | | 0.82 | | APC/CAV1/CSNK1A1/CUL3/GSK3B/KREMEN1/PSMC3/SFRP1/WNT5A/XPO1 | | 10 | |
| R-HSA-167161 | | HIV Transcription Initiation | | | 2/505 | | 47/10867 | | 6.5E-01 | | 0.89 | | 0.82 | | CCNH/GTF2F1 | | 2 | |
| R-HSA-167162 | | RNA Polymerase II HIV Promoter Escape | | | 2/505 | | 47/10867 | | 6.5E-01 | | 0.89 | | 0.82 | | CCNH/GTF2F1 | | 2 | |
| R-HSA-73776 | | RNA Polymerase II Promoter Escape | | | 2/505 | | 47/10867 | | 6.5E-01 | | 0.89 | | 0.82 | | CCNH/GTF2F1 | | 2 | |
| R-HSA-73779 | | RNA Polymerase II Transcription Pre-Initiation And Promoter Opening | | | 2/505 | | 47/10867 | | 6.5E-01 | | 0.89 | | 0.82 | | CCNH/GTF2F1 | | 2 | |
| R-HSA-75953 | | RNA Polymerase II Transcription Initiation | | | 2/505 | | 47/10867 | | 6.5E-01 | | 0.89 | | 0.82 | | CCNH/GTF2F1 | | 2 | |
| R-HSA-76042 | | RNA Polymerase II Transcription Initiation And Promoter Clearance | | | 2/505 | | 47/10867 | | 6.5E-01 | | 0.89 | | 0.82 | | CCNH/GTF2F1 | | 2 | |
| R-HSA-977606 | | Regulation of Complement cascade | | | 2/505 | | 47/10867 | | 6.5E-01 | | 0.89 | | 0.82 | | C1QA/PROS1 | | 2 | |
| R-HSA-2979096 | | NOTCH2 Activation and Transmission of Signal to the Nucleus | | | 1/505 | | 22/10867 | | 6.5E-01 | | 0.89 | | 0.82 | | ADAM10 | | 1 | |
| R-HSA-3296482 | | Defects in vitamin and cofactor metabolism | | | 1/505 | | 22/10867 | | 6.5E-01 | | 0.89 | | 0.82 | | ACACA | | 1 | |
| R-HSA-5205685 | | PINK1-PRKN Mediated Mitophagy | | | 1/505 | | 22/10867 | | 6.5E-01 | | 0.89 | | 0.82 | | SQSTM1 | | 1 | |
| R-HSA-8862803 | | Deregulated CDK5 triggers multiple neurodegenerative pathways in Alzheimer's disease models | | | 1/505 | | 22/10867 | | 6.5E-01 | | 0.89 | | 0.82 | | FOXO3 | | 1 | |
| R-HSA-8863678 | | Neurodegenerative Diseases | | | 1/505 | | 22/10867 | | 6.5E-01 | | 0.89 | | 0.82 | | FOXO3 | | 1 | |
| R-HSA-9609523 | | Insertion of tail-anchored proteins into the endoplasmic reticulum membrane | | | 1/505 | | 22/10867 | | 6.5E-01 | | 0.89 | | 0.82 | | VAMP2 | | 1 | |
| R-HSA-9617324 | | Negative regulation of NMDA receptor-mediated neuronal transmission | | | 1/505 | | 22/10867 | | 6.5E-01 | | 0.89 | | 0.82 | | CAMK4 | | 1 | |
| R-HSA-1592230 | | Mitochondrial biogenesis | | | 4/505 | | 95/10867 | | 6.5E-01 | | 0.89 | | 0.82 | | CAMK4/IMMT/MEF2C/PRKAA2 | | 4 | |
| R-HSA-72613 | | Eukaryotic Translation Initiation | | | 5/505 | | 119/10867 | | 6.5E-01 | | 0.89 | | 0.82 | | RPL32/RPL36AL/RPS14/RPS21/RPS9 | | 5 | |
| R-HSA-72737 | | Cap-dependent Translation Initiation | | | 5/505 | | 119/10867 | | 6.5E-01 | | 0.89 | | 0.82 | | RPL32/RPL36AL/RPS14/RPS21/RPS9 | | 5 | |
| R-HSA-448424 | | Interleukin-17 signaling | | | 3/505 | | 72/10867 | | 6.6E-01 | | 0.89 | | 0.82 | | DUSP4/MEF2C/TAB1 | | 3 | |
| R-HSA-3781865 | | Diseases of glycosylation | | | 6/505 | | 143/10867 | | 6.6E-01 | | 0.89 | | 0.82 | | ALG13/SEMA5A/THBS1/THSD4/THSD7A/VCAN | | 6 | |
| R-HSA-69239 | | Synthesis of DNA | | | 5/505 | | 120/10867 | | 6.6E-01 | | 0.89 | | 0.82 | | CCNE2/PCNA/POLD4/PSMC3/RFC5 | | 5 | |
| R-HSA-140837 | | Intrinsic Pathway of Fibrin Clot Formation | | | 1/505 | | 23/10867 | | 6.7E-01 | | 0.89 | | 0.82 | | PROS1 | | 1 | |
| R-HSA-171319 | | Telomere Extension By Telomerase | | | 1/505 | | 23/10867 | | 6.7E-01 | | 0.89 | | 0.82 | | TERF2IP | | 1 | |
| R-HSA-2173795 | | Downregulation of SMAD2/3:SMAD4 transcriptional activity | | | 1/505 | | 23/10867 | | 6.7E-01 | | 0.89 | | 0.82 | | RNF111 | | 1 | |
| R-HSA-450321 | | JNK (c-Jun kinases) phosphorylation and activation mediated by activated human TAK1 | | | 1/505 | | 23/10867 | | 6.7E-01 | | 0.89 | | 0.82 | | TAB1 | | 1 | |
| R-HSA-5620922 | | BBSome-mediated cargo-targeting to cilium | | | 1/505 | | 23/10867 | | 6.7E-01 | | 0.89 | | 0.82 | | RAB3IP | | 1 | |
| R-HSA-5654693 | | FRS-mediated FGFR1 signaling | | | 1/505 | | 23/10867 | | 6.7E-01 | | 0.89 | | 0.82 | | FRS2 | | 1 | |
| R-HSA-5654699 | | SHC-mediated cascade:FGFR2 | | | 1/505 | | 23/10867 | | 6.7E-01 | | 0.89 | | 0.82 | | FGF18 | | 1 | |
| R-HSA-9637690 | | Response of Mtb to phagocytosis | | | 1/505 | | 23/10867 | | 6.7E-01 | | 0.89 | | 0.82 | | KPNA1 | | 1 | |
| R-HSA-8854518 | | AURKA Activation by TPX2 | | | 3/505 | | 73/10867 | | 6.7E-01 | | 0.89 | | 0.82 | | CCP110/HMMR/HSP90AA1 | | 3 | |
| R-HSA-8939902 | | Regulation of RUNX2 expression and activity | | | 3/505 | | 73/10867 | | 6.7E-01 | | 0.89 | | 0.82 | | BMP2/GSK3B/PSMC3 | | 3 | |
| R-HSA-72172 | | mRNA Splicing | | | 8/505 | | 191/10867 | | 6.7E-01 | | 0.90 | | 0.83 | | CD2BP2/CSTF2T/DHX9/ELAVL1/FUS/GTF2F1/SNRNP200/SRSF1 | | 8 | |
| R-HSA-157858 | | Gap junction trafficking and regulation | | | 2/505 | | 49/10867 | | 6.7E-01 | | 0.90 | | 0.83 | | CLTC/TJP1 | | 2 | |
| R-HSA-6811440 | | Retrograde transport at the Trans-Golgi-Network | | | 2/505 | | 49/10867 | | 6.7E-01 | | 0.90 | | 0.83 | | GCC2/SCOC | | 2 | |
| R-HSA-418555 | | G alpha (s) signalling events | | | 6/505 | | 145/10867 | | 6.7E-01 | | 0.90 | | 0.83 | | CALCRL/GNAI2/GNG12/GPR15/P2RY11/RAMP2 | | 6 | |
| R-HSA-202424 | | Downstream TCR signaling | | | 4/505 | | 98/10867 | | 6.7E-01 | | 0.90 | | 0.83 | | HLA-DPB1/LCK/PIK3R1/PSMC3 | | 4 | |
| R-HSA-5619102 | | SLC transporter disorders | | | 4/505 | | 98/10867 | | 6.7E-01 | | 0.90 | | 0.83 | | BSG/RANBP2/SLC1A1/SLCO2A1 | | 4 | |
| R-HSA-5693607 | | Processing of DNA double-strand break ends | | | 4/505 | | 98/10867 | | 6.7E-01 | | 0.90 | | 0.83 | | BLM/PPP4R2/RAD50/RFC5 | | 4 | |
| R-HSA-3371571 | | HSF1-dependent transactivation | | | 1/505 | | 24/10867 | | 6.8E-01 | | 0.90 | | 0.83 | | HSP90AA1 | | 1 | |
| R-HSA-400508 | | Incretin synthesis. secretion. and inactivation | | | 1/505 | | 24/10867 | | 6.8E-01 | | 0.90 | | 0.83 | | PCSK1 | | 1 | |
| R-HSA-450302 | | activated TAK1 mediates p38 MAPK activation | | | 1/505 | | 24/10867 | | 6.8E-01 | | 0.90 | | 0.83 | | TAB1 | | 1 | |
| R-HSA-452723 | | Transcriptional regulation of pluripotent stem cells | | | 1/505 | | 24/10867 | | 6.8E-01 | | 0.90 | | 0.83 | | SALL1 | | 1 | |
| R-HSA-5668599 | | RHO GTPases Activate NADPH Oxidases | | | 1/505 | | 24/10867 | | 6.8E-01 | | 0.90 | | 0.83 | | RAC2 | | 1 | |
| R-HSA-8940973 | | RUNX2 regulates osteoblast differentiation | | | 1/505 | | 24/10867 | | 6.8E-01 | | 0.90 | | 0.83 | | COL1A1 | | 1 | |
| R-HSA-9620244 | | Long-term potentiation | | | 1/505 | | 24/10867 | | 6.8E-01 | | 0.90 | | 0.83 | | ERBB4 | | 1 | |
| R-HSA-982772 | | Growth hormone receptor signaling | | | 1/505 | | 24/10867 | | 6.8E-01 | | 0.90 | | 0.83 | | PTPN6 | | 1 | |
| R-HSA-1483249 | | Inositol phosphate metabolism | | | 2/505 | | 50/10867 | | 6.8E-01 | | 0.90 | | 0.83 | | INPP4B/NUDT4 | | 2 | |
| R-HSA-381038 | | XBP1(S) activates chaperone genes | | | 2/505 | | 50/10867 | | 6.8E-01 | | 0.90 | | 0.83 | | DNAJB9/TLN1 | | 2 | |
| R-HSA-1234174 | | Cellular response to hypoxia | | | 3/505 | | 75/10867 | | 6.8E-01 | | 0.90 | | 0.83 | | CITED2/EGLN3/PSMC3 | | 3 | |
| R-HSA-159236 | | Transport of Mature mRNA derived from an Intron-Containing Transcript | | | 3/505 | | 75/10867 | | 6.8E-01 | | 0.90 | | 0.83 | | RANBP2/SRSF1/ZC3H11A | | 3 | |
| R-HSA-2995410 | | Nuclear Envelope (NE) Reassembly | | | 3/505 | | 75/10867 | | 6.8E-01 | | 0.90 | | 0.83 | | CCNB2/CHMP3/VRK1 | | 3 | |
| R-HSA-9010553 | | Regulation of expression of SLITs and ROBOs | | | 7/505 | | 171/10867 | | 6.9E-01 | | 0.90 | | 0.83 | | PSMC3/RPL32/RPL36AL/RPS14/RPS21/RPS9/SLIT2 | | 7 | |
| R-HSA-427389 | | ERCC6 (CSB) and EHMT2 (G9a) positively regulate rRNA expression | | | 3/505 | | 76/10867 | | 6.9E-01 | | 0.90 | | 0.83 | | CBX3/MBD3/MTA3 | | 3 | |
| R-HSA-9659379 | | Sensory processing of sound | | | 3/505 | | 76/10867 | | 6.9E-01 | | 0.90 | | 0.83 | | MSN/RAB3A/VAMP2 | | 3 | |
| R-HSA-350562 | | Regulation of ornithine decarboxylase (ODC) | | | 2/505 | | 51/10867 | | 6.9E-01 | | 0.90 | | 0.83 | | OAZ1/PSMC3 | | 2 | |
| R-HSA-1187000 | | Fertilization | | | 1/505 | | 25/10867 | | 7.0E-01 | | 0.90 | | 0.83 | | HVCN1 | | 1 | |
| R-HSA-156588 | | Glucuronidation | | | 1/505 | | 25/10867 | | 7.0E-01 | | 0.90 | | 0.83 | | SLC35D1 | | 1 | |
| R-HSA-392451 | | G beta:gamma signalling through PI3Kgamma | | | 1/505 | | 25/10867 | | 7.0E-01 | | 0.90 | | 0.83 | | GNG12 | | 1 | |
| R-HSA-9006335 | | Signaling by Erythropoietin | | | 1/505 | | 25/10867 | | 7.0E-01 | | 0.90 | | 0.83 | | PIK3R1 | | 1 | |
| R-HSA-9013507 | | NOTCH3 Activation and Transmission of Signal to the Nucleus | | | 1/505 | | 25/10867 | | 7.0E-01 | | 0.90 | | 0.83 | | ADAM10 | | 1 | |
| R-HSA-933542 | | TRAF6 mediated NF-kB activation | | | 1/505 | | 25/10867 | | 7.0E-01 | | 0.90 | | 0.83 | | MAP3K1 | | 1 | |
| R-HSA-381070 | | IRE1alpha activates chaperones | | | 2/505 | | 52/10867 | | 7.0E-01 | | 0.91 | | 0.84 | | DNAJB9/TLN1 | | 2 | |
| R-HSA-445717 | | Aquaporin-mediated transport | | | 2/505 | | 52/10867 | | 7.0E-01 | | 0.91 | | 0.84 | | GNG12/RAB11A | | 2 | |
| R-HSA-75815 | | Ubiquitin-dependent degradation of Cyclin D | | | 2/505 | | 52/10867 | | 7.0E-01 | | 0.91 | | 0.84 | | GSK3B/PSMC3 | | 2 | |
| R-HSA-72203 | | Processing of Capped Intron-Containing Pre-mRNA | | | 10/505 | | 244/10867 | | 7.0E-01 | | 0.91 | | 0.84 | | CD2BP2/CSTF2T/DHX9/ELAVL1/FUS/GTF2F1/RANBP2/SNRNP200/SRSF1/ZC3H11A | | 10 | |
| R-HSA-5689603 | | UCH proteinases | | | 4/505 | | 102/10867 | | 7.0E-01 | | 0.91 | | 0.84 | | PSMC3/TGFBR1/TGFBR2/YY1 | | 4 | |
| R-HSA-9612973 | | Autophagy | | | 6/505 | | 151/10867 | | 7.1E-01 | | 0.91 | | 0.84 | | CHMP3/HSP90AA1/PRKAA1/PRKAA2/SQSTM1/VIM | | 6 | |
| R-HSA-74752 | | Signaling by Insulin receptor | | | 3/505 | | 78/10867 | | 7.1E-01 | | 0.91 | | 0.84 | | FGF18/FRS2/PIK3R1 | | 3 | |
| R-HSA-1971475 | | A tetrasaccharide linker sequence is required for GAG synthesis | | | 1/505 | | 26/10867 | | 7.1E-01 | | 0.91 | | 0.84 | | VCAN | | 1 | |
| R-HSA-6803529 | | FGFR2 alternative splicing | | | 1/505 | | 26/10867 | | 7.1E-01 | | 0.91 | | 0.84 | | GTF2F1 | | 1 | |
| R-HSA-382556 | | ABC-family proteins mediated transport | | | 4/505 | | 103/10867 | | 7.1E-01 | | 0.92 | | 0.84 | | ABCA6/PEX19/PSMC3/SEL1L | | 4 | |
| R-HSA-8852135 | | Protein ubiquitination | | | 3/505 | | 79/10867 | | 7.2E-01 | | 0.92 | | 0.85 | | PCNA/RNF20/TMEM129 | | 3 | |
| R-HSA-1483206 | | Glycerophospholipid biosynthesis | | | 5/505 | | 129/10867 | | 7.2E-01 | | 0.92 | | 0.85 | | GPD1L/LIPH/MIGA2/PLA2G2A/PLA2G4A | | 5 | |
| R-HSA-6781823 | | Formation of TC-NER Pre-Incision Complex | | | 2/505 | | 54/10867 | | 7.2E-01 | | 0.92 | | 0.85 | | CCNH/COPS6 | | 2 | |
| R-HSA-8854050 | | FBXL7 down-regulates AURKA during mitotic entry and in early mitosis | | | 2/505 | | 54/10867 | | 7.2E-01 | | 0.92 | | 0.85 | | FBXL7/PSMC3 | | 2 | |
| R-HSA-5617833 | | Cilium Assembly | | | 8/505 | | 201/10867 | | 7.2E-01 | | 0.92 | | 0.85 | | CCP110/HSP90AA1/INPP5E/KIF3B/RAB11A/RAB3IP/RP2/SEPTIN2 | | 8 | |
| R-HSA-418594 | | G alpha (i) signalling events | | | 13/505 | | 318/10867 | | 7.2E-01 | | 0.92 | | 0.85 | | ACKR3/ADRA2A/CAMK4/CXCL8/GNAI2/GNAQ/GNG12/ITPR1/KPNA2/PLA2G4A/PSAP/RGS4/RGS5 | | 13 | |
| R-HSA-1368108 | | BMAL1:CLOCK.NPAS2 activates circadian gene expression | | | 1/505 | | 27/10867 | | 7.2E-01 | | 0.92 | | 0.85 | | BHLHE40 | | 1 | |
| R-HSA-211976 | | Endogenous sterols | | | 1/505 | | 27/10867 | | 7.2E-01 | | 0.92 | | 0.85 | | FDXR | | 1 | |
| R-HSA-380994 | | ATF4 activates genes in response to endoplasmic reticulum stress | | | 1/505 | | 27/10867 | | 7.2E-01 | | 0.92 | | 0.85 | | CXCL8 | | 1 | |
| R-HSA-5357769 | | Caspase activation via extrinsic apoptotic signalling pathway | | | 1/505 | | 27/10867 | | 7.2E-01 | | 0.92 | | 0.85 | | TLR4 | | 1 | |
| R-HSA-5621480 | | Dectin-2 family | | | 1/505 | | 27/10867 | | 7.2E-01 | | 0.92 | | 0.85 | | FYN | | 1 | |
| R-HSA-9635486 | | Infection with Mycobacterium tuberculosis | | | 1/505 | | 27/10867 | | 7.2E-01 | | 0.92 | | 0.85 | | KPNA1 | | 1 | |
| R-HSA-1236975 | | Antigen processing-Cross presentation | | | 4/505 | | 105/10867 | | 7.3E-01 | | 0.92 | | 0.85 | | BTK/ITGB5/PSMC3/TLR4 | | 4 | |
| R-HSA-6811442 | | Intra-Golgi and retrograde Golgi-to-ER traffic | | | 8/505 | | 202/10867 | | 7.3E-01 | | 0.92 | | 0.85 | | ARCN1/GALNT1/GCC2/KIF18B/KIF3B/PAFAH1B2/PLA2G4A/SCOC | | 8 | |
| R-HSA-1852241 | | Organelle biogenesis and maintenance | | | 12/505 | | 296/10867 | | 7.3E-01 | | 0.92 | | 0.85 | | CAMK4/CCP110/HSP90AA1/IMMT/INPP5E/KIF3B/MEF2C/PRKAA2/RAB11A/RAB3IP/RP2/SEPTIN2 | | 12 | |
| R-HSA-6794361 | | Neurexins and neuroligins | | | 2/505 | | 55/10867 | | 7.3E-01 | | 0.92 | | 0.85 | | NLGN2/NRXN3 | | 2 | |
| R-HSA-5250913 | | Positive epigenetic regulation of rRNA expression | | | 4/505 | | 106/10867 | | 7.3E-01 | | 0.92 | | 0.85 | | CBX3/GSK3B/MBD3/MTA3 | | 4 | |
| R-HSA-2022854 | | Keratan sulfate biosynthesis | | | 1/505 | | 28/10867 | | 7.4E-01 | | 0.92 | | 0.85 | | ST3GAL2 | | 1 | |
| R-HSA-5620971 | | Pyroptosis | | | 1/505 | | 28/10867 | | 7.4E-01 | | 0.92 | | 0.85 | | CHMP3 | | 1 | |
| R-HSA-75067 | | Processing of Capped Intronless Pre-mRNA | | | 1/505 | | 28/10867 | | 7.4E-01 | | 0.92 | | 0.85 | | CSTF2T | | 1 | |
| R-HSA-76071 | | RNA Polymerase III Transcription Initiation From Type 3 Promoter | | | 1/505 | | 28/10867 | | 7.4E-01 | | 0.92 | | 0.85 | | SNAPC4 | | 1 | |
| R-HSA-9619665 | | EGR2 and SOX10-mediated initiation of Schwann cell myelination | | | 1/505 | | 28/10867 | | 7.4E-01 | | 0.92 | | 0.85 | | UTRN | | 1 | |
| R-HSA-168928 | | DDX58/IFIH1-mediated induction of interferon-alpha/beta | | | 3/505 | | 82/10867 | | 7.4E-01 | | 0.92 | | 0.85 | | HSP90AA1/IRF7/MAP3K1 | | 3 | |
| R-HSA-380270 | | Recruitment of mitotic centrosome proteins and complexes | | | 3/505 | | 82/10867 | | 7.4E-01 | | 0.92 | | 0.85 | | CCP110/HSP90AA1/TUBGCP6 | | 3 | |
| R-HSA-380287 | | Centrosome maturation | | | 3/505 | | 82/10867 | | 7.4E-01 | | 0.92 | | 0.85 | | CCP110/HSP90AA1/TUBGCP6 | | 3 | |
| R-HSA-9013694 | | Signaling by NOTCH4 | | | 3/505 | | 82/10867 | | 7.4E-01 | | 0.92 | | 0.85 | | ADAM10/FLT4/PSMC3 | | 3 | |
| R-HSA-2151201 | | Transcriptional activation of mitochondrial biogenesis | | | 2/505 | | 56/10867 | | 7.4E-01 | | 0.92 | | 0.85 | | CAMK4/MEF2C | | 2 | |
| R-HSA-5362768 | | Hh mutants are degraded by ERAD | | | 2/505 | | 56/10867 | | 7.4E-01 | | 0.92 | | 0.85 | | PSMC3/SEL1L | | 2 | |
| R-HSA-112314 | | Neurotransmitter receptors and postsynaptic signal transmission | | | 8/505 | | 205/10867 | | 7.4E-01 | | 0.92 | | 0.85 | | CAMK4/ERBB4/GNAI2/GNG12/KPNA2/NPTN/PRKAA1/PRKAA2 | | 8 | |
| R-HSA-3108232 | | SUMO E3 ligases SUMOylate target proteins | | | 7/505 | | 182/10867 | | 7.5E-01 | | 0.92 | | 0.85 | | BLM/DNMT1/NRIP1/PCNA/RANBP2/TFAP2A/TRIM28 | | 7 | |
| R-HSA-1296041 | | Activation of G protein gated Potassium channels | | | 1/505 | | 29/10867 | | 7.5E-01 | | 0.92 | | 0.85 | | GNG12 | | 1 | |
| R-HSA-1296059 | | G protein gated Potassium channels | | | 1/505 | | 29/10867 | | 7.5E-01 | | 0.92 | | 0.85 | | GNG12 | | 1 | |
| R-HSA-162588 | | Budding and maturation of HIV virion | | | 1/505 | | 29/10867 | | 7.5E-01 | | 0.92 | | 0.85 | | CHMP3 | | 1 | |
| R-HSA-186763 | | Downstream signal transduction | | | 1/505 | | 29/10867 | | 7.5E-01 | | 0.92 | | 0.85 | | PIK3R1 | | 1 | |
| R-HSA-390918 | | Peroxisomal lipid metabolism | | | 1/505 | | 29/10867 | | 7.5E-01 | | 0.92 | | 0.85 | | CROT | | 1 | |
| R-HSA-5205647 | | Mitophagy | | | 1/505 | | 29/10867 | | 7.5E-01 | | 0.92 | | 0.85 | | SQSTM1 | | 1 | |
| R-HSA-5601884 | | PIWI-interacting RNA (piRNA) biogenesis | | | 1/505 | | 29/10867 | | 7.5E-01 | | 0.92 | | 0.85 | | HSP90AA1 | | 1 | |
| R-HSA-8982491 | | Glycogen metabolism | | | 1/505 | | 29/10867 | | 7.5E-01 | | 0.92 | | 0.85 | | PGM2L1 | | 1 | |
| R-HSA-997272 | | Inhibition of voltage gated Ca2+ channels via Gbeta/gamma subunits | | | 1/505 | | 29/10867 | | 7.5E-01 | | 0.92 | | 0.85 | | GNG12 | | 1 | |
| R-HSA-168643 | | Nucleotide-binding domain. leucine rich repeat containing receptor (NLR) signaling pathways | | | 2/505 | | 57/10867 | | 7.5E-01 | | 0.92 | | 0.85 | | TAB1/TXNIP | | 2 | |
| R-HSA-4641258 | | Degradation of DVL | | | 2/505 | | 57/10867 | | 7.5E-01 | | 0.92 | | 0.85 | | CUL3/PSMC3 | | 2 | |
| R-HSA-69541 | | Stabilization of p53 | | | 2/505 | | 57/10867 | | 7.5E-01 | | 0.92 | | 0.85 | | PHF20/PSMC3 | | 2 | |
| R-HSA-8932339 | | ROS sensing by NFE2L2 | | | 2/505 | | 57/10867 | | 7.5E-01 | | 0.92 | | 0.85 | | CUL3/PSMC3 | | 2 | |
| R-HSA-936837 | | Ion transport by P-type ATPases | | | 2/505 | | 57/10867 | | 7.5E-01 | | 0.92 | | 0.85 | | ATP2A2/ATP7A | | 2 | |
| R-HSA-68886 | | M Phase | | | 17/505 | | 418/10867 | | 7.5E-01 | | 0.92 | | 0.85 | | CCNB2/CCP110/CENPA/CENPQ/CHMP3/CLIP1/ESPL1/HSP90AA1/PDS5B/PSMC3/RANBP2/SGO1/SMC4/TUBGCP6/VRK1/WAPL/XPO1 | | 17 | |
| R-HSA-381340 | | Transcriptional regulation of white adipocyte differentiation | | | 3/505 | | 84/10867 | | 7.6E-01 | | 0.93 | | 0.86 | | ADIRF/CEBPA/ZNF638 | | 3 | |
| R-HSA-72202 | | Transport of Mature Transcript to Cytoplasm | | | 3/505 | | 84/10867 | | 7.6E-01 | | 0.93 | | 0.86 | | RANBP2/SRSF1/ZC3H11A | | 3 | |
| R-HSA-5250941 | | Negative epigenetic regulation of rRNA expression | | | 4/505 | | 110/10867 | | 7.6E-01 | | 0.93 | | 0.86 | | BAZ2A/CCNH/DNMT1/SIRT1 | | 4 | |
| R-HSA-73854 | | RNA Polymerase I Promoter Clearance | | | 4/505 | | 110/10867 | | 7.6E-01 | | 0.93 | | 0.86 | | CBX3/CCNH/MBD3/MTA3 | | 4 | |
| R-HSA-3000157 | | Laminin interactions | | | 1/505 | | 30/10867 | | 7.6E-01 | | 0.93 | | 0.86 | | ITGA2 | | 1 | |
| R-HSA-3928663 | | EPHA-mediated growth cone collapse | | | 1/505 | | 30/10867 | | 7.6E-01 | | 0.93 | | 0.86 | | FYN | | 1 | |
| R-HSA-6804758 | | Regulation of TP53 Activity through Acetylation | | | 1/505 | | 30/10867 | | 7.6E-01 | | 0.93 | | 0.86 | | MBD3 | | 1 | |
| R-HSA-933541 | | TRAF6 mediated IRF7 activation | | | 1/505 | | 30/10867 | | 7.6E-01 | | 0.93 | | 0.86 | | IRF7 | | 1 | |
| R-HSA-351202 | | Metabolism of polyamines | | | 2/505 | | 59/10867 | | 7.7E-01 | | 0.93 | | 0.86 | | OAZ1/PSMC3 | | 2 | |
| R-HSA-5387390 | | Hh mutants abrogate ligand secretion | | | 2/505 | | 59/10867 | | 7.7E-01 | | 0.93 | | 0.86 | | PSMC3/SEL1L | | 2 | |
| R-HSA-68827 | | CDT1 association with the CDC6:ORC:origin complex | | | 2/505 | | 59/10867 | | 7.7E-01 | | 0.93 | | 0.86 | | GMNN/PSMC3 | | 2 | |
| R-HSA-983189 | | Kinesins | | | 2/505 | | 59/10867 | | 7.7E-01 | | 0.93 | | 0.86 | | KIF18B/KIF3B | | 2 | |
| R-HSA-73864 | | RNA Polymerase I Transcription | | | 4/505 | | 112/10867 | | 7.7E-01 | | 0.93 | | 0.86 | | CBX3/CCNH/MBD3/MTA3 | | 4 | |
| R-HSA-170822 | | Regulation of Glucokinase by Glucokinase Regulatory Protein | | | 1/505 | | 31/10867 | | 7.7E-01 | | 0.93 | | 0.86 | | RANBP2 | | 1 | |
| R-HSA-182971 | | EGFR downregulation | | | 1/505 | | 31/10867 | | 7.7E-01 | | 0.93 | | 0.86 | | SH3KBP1 | | 1 | |
| R-HSA-1839124 | | FGFR1 mutant receptor activation | | | 1/505 | | 31/10867 | | 7.7E-01 | | 0.93 | | 0.86 | | PIK3R1 | | 1 | |
| R-HSA-445095 | | Interaction between L1 and Ankyrins | | | 1/505 | | 31/10867 | | 7.7E-01 | | 0.93 | | 0.86 | | SCN1B | | 1 | |
| R-HSA-5619107 | | Defective TPR may confer susceptibility towards thyroid papillary carcinoma (TPC) | | | 1/505 | | 31/10867 | | 7.7E-01 | | 0.93 | | 0.86 | | RANBP2 | | 1 | |
| R-HSA-70268 | | Pyruvate metabolism | | | 1/505 | | 31/10867 | | 7.7E-01 | | 0.93 | | 0.86 | | BSG | | 1 | |
| R-HSA-8949613 | | Cristae formation | | | 1/505 | | 31/10867 | | 7.7E-01 | | 0.93 | | 0.86 | | IMMT | | 1 | |
| R-HSA-9668328 | | Sealing of the nuclear envelope (NE) by ESCRT-III | | | 1/505 | | 31/10867 | | 7.7E-01 | | 0.93 | | 0.86 | | CHMP3 | | 1 | |
| R-HSA-5610780 | | Degradation of GLI1 by the proteasome | | | 2/505 | | 60/10867 | | 7.8E-01 | | 0.93 | | 0.86 | | NUMB/PSMC3 | | 2 | |
| R-HSA-9609507 | | Protein localization | | | 6/505 | | 163/10867 | | 7.8E-01 | | 0.93 | | 0.86 | | CROT/IDH1/LONP2/PEX1/PEX19/VAMP2 | | 6 | |
| R-HSA-5628897 | | TP53 Regulates Metabolic Genes | | | 3/505 | | 87/10867 | | 7.8E-01 | | 0.93 | | 0.86 | | COX7C/PRKAA1/PRKAA2 | | 3 | |
| R-HSA-5610787 | | Hedgehog 'off' state | | | 4/505 | | 113/10867 | | 7.8E-01 | | 0.93 | | 0.86 | | CSNK1A1/GSK3B/NUMB/PSMC3 | | 4 | |
| R-HSA-2990846 | | SUMOylation | | | 7/505 | | 188/10867 | | 7.8E-01 | | 0.93 | | 0.86 | | BLM/DNMT1/NRIP1/PCNA/RANBP2/TFAP2A/TRIM28 | | 7 | |
| R-HSA-2871796 | | FCERI mediated MAPK activation | | | 1/505 | | 32/10867 | | 7.8E-01 | | 0.93 | | 0.86 | | MAP3K1 | | 1 | |
| R-HSA-381042 | | PERK regulates gene expression | | | 1/505 | | 32/10867 | | 7.8E-01 | | 0.93 | | 0.86 | | CXCL8 | | 1 | |
| R-HSA-451326 | | Activation of kainate receptors upon glutamate binding | | | 1/505 | | 32/10867 | | 7.8E-01 | | 0.93 | | 0.86 | | GNG12 | | 1 | |
| R-HSA-73863 | | RNA Polymerase I Transcription Termination | | | 1/505 | | 32/10867 | | 7.8E-01 | | 0.93 | | 0.86 | | CCNH | | 1 | |
| R-HSA-8941326 | | RUNX2 regulates bone development | | | 1/505 | | 32/10867 | | 7.8E-01 | | 0.93 | | 0.86 | | COL1A1 | | 1 | |
| R-HSA-917729 | | Endosomal Sorting Complex Required For Transport (ESCRT) | | | 1/505 | | 32/10867 | | 7.8E-01 | | 0.93 | | 0.86 | | CHMP3 | | 1 | |
| R-HSA-2565942 | | Regulation of PLK1 Activity at G2/M Transition | | | 3/505 | | 88/10867 | | 7.8E-01 | | 0.93 | | 0.86 | | CCNB2/CCP110/HSP90AA1 | | 3 | |
| R-HSA-5678895 | | Defective CFTR causes cystic fibrosis | | | 2/505 | | 61/10867 | | 7.8E-01 | | 0.93 | | 0.86 | | PSMC3/SEL1L | | 2 | |
| R-HSA-8878171 | | Transcriptional regulation by RUNX1 | | | 9/505 | | 239/10867 | | 7.9E-01 | | 0.93 | | 0.86 | | CCND2/CCNH/FOXP3/PSMC3/RUNX1/SMARCC1/TCF12/THBS1/TJP1 | | 9 | |
| R-HSA-8986944 | | Transcriptional Regulation by MECP2 | | | 2/505 | | 62/10867 | | 7.9E-01 | | 0.93 | | 0.86 | | CAMK4/MEF2C | | 2 | |
| R-HSA-163359 | | Glucagon signaling in metabolic regulation | | | 1/505 | | 33/10867 | | 7.9E-01 | | 0.93 | | 0.86 | | GNG12 | | 1 | |
| R-HSA-180746 | | Nuclear import of Rev protein | | | 1/505 | | 33/10867 | | 7.9E-01 | | 0.93 | | 0.86 | | RANBP2 | | 1 | |
| R-HSA-1980145 | | Signaling by NOTCH2 | | | 1/505 | | 33/10867 | | 7.9E-01 | | 0.93 | | 0.86 | | ADAM10 | | 1 | |
| R-HSA-420092 | | Glucagon-type ligand receptors | | | 1/505 | | 33/10867 | | 7.9E-01 | | 0.93 | | 0.86 | | GNG12 | | 1 | |
| R-HSA-445989 | | TAK1 activates NFkB by phosphorylation and activation of IKKs complex | | | 1/505 | | 33/10867 | | 7.9E-01 | | 0.93 | | 0.86 | | TAB1 | | 1 | |
| R-HSA-5654726 | | Negative regulation of FGFR1 signaling | | | 1/505 | | 33/10867 | | 7.9E-01 | | 0.93 | | 0.86 | | FRS2 | | 1 | |
| R-HSA-68962 | | Activation of the pre-replicative complex | | | 1/505 | | 33/10867 | | 7.9E-01 | | 0.93 | | 0.86 | | GMNN | | 1 | |
| R-HSA-9022692 | | Regulation of MECP2 expression and activity | | | 1/505 | | 33/10867 | | 7.9E-01 | | 0.93 | | 0.86 | | CAMK4 | | 1 | |
| R-HSA-1236974 | | ER-Phagosome pathway | | | 3/505 | | 90/10867 | | 8.0E-01 | | 0.94 | | 0.86 | | BTK/PSMC3/TLR4 | | 3 | |
| R-HSA-9616222 | | Transcriptional regulation of granulopoiesis | | | 3/505 | | 90/10867 | | 8.0E-01 | | 0.94 | | 0.86 | | CDKN1A/CEBPA/RUNX1 | | 3 | |
| R-HSA-376176 | | Signaling by ROBO receptors | | | 8/505 | | 218/10867 | | 8.0E-01 | | 0.94 | | 0.86 | | NRP1/PSMC3/RPL32/RPL36AL/RPS14/RPS21/RPS9/SLIT2 | | 8 | |
| R-HSA-159227 | | Transport of the SLBP independent Mature mRNA | | | 1/505 | | 34/10867 | | 8.0E-01 | | 0.94 | | 0.86 | | RANBP2 | | 1 | |
| R-HSA-1638074 | | Keratan sulfate/keratin metabolism | | | 1/505 | | 34/10867 | | 8.0E-01 | | 0.94 | | 0.86 | | ST3GAL2 | | 1 | |
| R-HSA-187687 | | Signalling to ERKs | | | 1/505 | | 34/10867 | | 8.0E-01 | | 0.94 | | 0.86 | | FRS2 | | 1 | |
| R-HSA-4085377 | | SUMOylation of SUMOylation proteins | | | 1/505 | | 34/10867 | | 8.0E-01 | | 0.94 | | 0.86 | | RANBP2 | | 1 | |
| R-HSA-5576892 | | Phase 0 - rapid depolarisation | | | 1/505 | | 34/10867 | | 8.0E-01 | | 0.94 | | 0.86 | | SCN1B | | 1 | |
| R-HSA-5663084 | | Diseases of carbohydrate metabolism | | | 1/505 | | 34/10867 | | 8.0E-01 | | 0.94 | | 0.86 | | HYAL1 | | 1 | |
| R-HSA-4608870 | | Asymmetric localization of PCP proteins | | | 2/505 | | 64/10867 | | 8.0E-01 | | 0.94 | | 0.87 | | PSMC3/WNT5A | | 2 | |
| R-HSA-1296065 | | Inwardly rectifying K+ channels | | | 1/505 | | 35/10867 | | 8.1E-01 | | 0.94 | | 0.87 | | GNG12 | | 1 | |
| R-HSA-159230 | | Transport of the SLBP Dependant Mature mRNA | | | 1/505 | | 35/10867 | | 8.1E-01 | | 0.94 | | 0.87 | | RANBP2 | | 1 | |
| R-HSA-196071 | | Metabolism of steroid hormones | | | 1/505 | | 35/10867 | | 8.1E-01 | | 0.94 | | 0.87 | | FDXR | | 1 | |
| R-HSA-5357905 | | Regulation of TNFR1 signaling | | | 1/505 | | 35/10867 | | 8.1E-01 | | 0.94 | | 0.87 | | RACK1 | | 1 | |
| R-HSA-5358346 | | Hedgehog ligand biogenesis | | | 2/505 | | 65/10867 | | 8.1E-01 | | 0.94 | | 0.87 | | PSMC3/SEL1L | | 2 | |
| R-HSA-9707587 | | Regulation of HMOX1 expression and activity | | | 2/505 | | 65/10867 | | 8.1E-01 | | 0.94 | | 0.87 | | CUL3/PSMC3 | | 2 | |
| R-HSA-1234176 | | Oxygen-dependent proline hydroxylation of Hypoxia-inducible Factor Alpha | | | 2/505 | | 66/10867 | | 8.2E-01 | | 0.94 | | 0.87 | | EGLN3/PSMC3 | | 2 | |
| R-HSA-211897 | | Cytochrome P450 - arranged by substrate type | | | 2/505 | | 66/10867 | | 8.2E-01 | | 0.94 | | 0.87 | | CYP4F12/FDXR | | 2 | |
| R-HSA-380320 | | Recruitment of NuMA to mitotic centrosomes | | | 3/505 | | 94/10867 | | 8.2E-01 | | 0.94 | | 0.87 | | CCP110/HSP90AA1/TUBGCP6 | | 3 | |
| R-HSA-381119 | | Unfolded Protein Response (UPR) | | | 3/505 | | 94/10867 | | 8.2E-01 | | 0.94 | | 0.87 | | CXCL8/DNAJB9/TLN1 | | 3 | |
| R-HSA-156590 | | Glutathione conjugation | | | 1/505 | | 36/10867 | | 8.2E-01 | | 0.94 | | 0.87 | | GGCT | | 1 | |
| R-HSA-390522 | | Striated Muscle Contraction | | | 1/505 | | 36/10867 | | 8.2E-01 | | 0.94 | | 0.87 | | VIM | | 1 | |
| R-HSA-6804757 | | Regulation of TP53 Degradation | | | 1/505 | | 36/10867 | | 8.2E-01 | | 0.94 | | 0.87 | | PHF20 | | 1 | |
| R-HSA-76046 | | RNA Polymerase III Transcription Initiation | | | 1/505 | | 36/10867 | | 8.2E-01 | | 0.94 | | 0.87 | | SNAPC4 | | 1 | |
| R-HSA-8956319 | | Nucleobase catabolism | | | 1/505 | | 36/10867 | | 8.2E-01 | | 0.94 | | 0.87 | | NT5E | | 1 | |
| R-HSA-69473 | | G2/M DNA damage checkpoint | | | 3/505 | | 95/10867 | | 8.2E-01 | | 0.95 | | 0.88 | | BLM/RAD50/RFC5 | | 3 | |
| R-HSA-168638 | | NOD1/2 Signaling Pathway | | | 1/505 | | 37/10867 | | 8.3E-01 | | 0.95 | | 0.88 | | TAB1 | | 1 | |
| R-HSA-176187 | | Activation of ATR in response to replication stress | | | 1/505 | | 37/10867 | | 8.3E-01 | | 0.95 | | 0.88 | | RFC5 | | 1 | |
| R-HSA-3299685 | | Detoxification of Reactive Oxygen Species | | | 1/505 | | 37/10867 | | 8.3E-01 | | 0.95 | | 0.88 | | ATP7A | | 1 | |
| R-HSA-6806003 | | Regulation of TP53 Expression and Degradation | | | 1/505 | | 37/10867 | | 8.3E-01 | | 0.95 | | 0.88 | | PHF20 | | 1 | |
| R-HSA-5658442 | | Regulation of RAS by GAPs | | | 2/505 | | 68/10867 | | 8.3E-01 | | 0.95 | | 0.88 | | CUL3/PSMC3 | | 2 | |
| R-HSA-68867 | | Assembly of the pre-replicative complex | | | 2/505 | | 68/10867 | | 8.3E-01 | | 0.95 | | 0.88 | | GMNN/PSMC3 | | 2 | |
| R-HSA-5619115 | | Disorders of transmembrane transporters | | | 6/505 | | 176/10867 | | 8.3E-01 | | 0.95 | | 0.88 | | BSG/PSMC3/RANBP2/SEL1L/SLC1A1/SLCO2A1 | | 6 | |
| R-HSA-9707564 | | Cytoprotection by HMOX1 | | | 4/505 | | 124/10867 | | 8.3E-01 | | 0.95 | | 0.88 | | COX7C/CUL3/PSMC3/TXNIP | | 4 | |
| R-HSA-3232142 | | SUMOylation of ubiquitinylation proteins | | | 1/505 | | 38/10867 | | 8.4E-01 | | 0.96 | | 0.88 | | RANBP2 | | 1 | |
| R-HSA-380259 | | Loss of Nlp from mitotic centrosomes | | | 2/505 | | 70/10867 | | 8.4E-01 | | 0.96 | | 0.89 | | CCP110/HSP90AA1 | | 2 | |
| R-HSA-380284 | | Loss of proteins required for interphase microtubule organization from the centrosome | | | 2/505 | | 70/10867 | | 8.4E-01 | | 0.96 | | 0.89 | | CCP110/HSP90AA1 | | 2 | |
| R-HSA-5689896 | | Ovarian tumor domain proteases | | | 1/505 | | 39/10867 | | 8.4E-01 | | 0.96 | | 0.89 | | APC | | 1 | |
| R-HSA-6811434 | | COPI-dependent Golgi-to-ER retrograde traffic | | | 3/505 | | 99/10867 | | 8.5E-01 | | 0.96 | | 0.89 | | ARCN1/KIF18B/KIF3B | | 3 | |
| R-HSA-109581 | | Apoptosis | | | 6/505 | | 180/10867 | | 8.5E-01 | | 0.96 | | 0.89 | | APC/KPNA1/PSMC3/TJP1/TLR4/VIM | | 6 | |
| R-HSA-70171 | | Glycolysis | | | 2/505 | | 71/10867 | | 8.5E-01 | | 0.96 | | 0.89 | | PGM2L1/RANBP2 | | 2 | |
| R-HSA-15869 | | Metabolism of nucleotides | | | 3/505 | | 100/10867 | | 8.5E-01 | | 0.96 | | 0.89 | | NME3/NT5E/RRM2 | | 3 | |
| R-HSA-5607764 | | CLEC7A (Dectin-1) signaling | | | 3/505 | | 101/10867 | | 8.5E-01 | | 0.97 | | 0.89 | | ITPR1/PSMC3/TAB1 | | 3 | |
| R-HSA-159231 | | Transport of Mature mRNA Derived from an Intronless Transcript | | | 1/505 | | 41/10867 | | 8.6E-01 | | 0.97 | | 0.89 | | RANBP2 | | 1 | |
| R-HSA-186712 | | Regulation of beta-cell development | | | 1/505 | | 41/10867 | | 8.6E-01 | | 0.97 | | 0.89 | | FOXO1 | | 1 | |
| R-HSA-3560782 | | Diseases associated with glycosaminoglycan metabolism | | | 1/505 | | 41/10867 | | 8.6E-01 | | 0.97 | | 0.89 | | VCAN | | 1 | |
| R-HSA-74158 | | RNA Polymerase III Transcription | | | 1/505 | | 41/10867 | | 8.6E-01 | | 0.97 | | 0.89 | | SNAPC4 | | 1 | |
| R-HSA-749476 | | RNA Polymerase III Abortive And Retractive Initiation | | | 1/505 | | 41/10867 | | 8.6E-01 | | 0.97 | | 0.89 | | SNAPC4 | | 1 | |
| R-HSA-212300 | | PRC2 methylates histones and DNA | | | 2/505 | | 73/10867 | | 8.6E-01 | | 0.97 | | 0.89 | | DNMT1/PHF19 | | 2 | |
| R-HSA-69017 | | CDK-mediated phosphorylation and removal of Cdc6 | | | 2/505 | | 73/10867 | | 8.6E-01 | | 0.97 | | 0.89 | | CCNE2/PSMC3 | | 2 | |
| R-HSA-8939236 | | RUNX1 regulates transcription of genes involved in differentiation of HSCs | | | 4/505 | | 130/10867 | | 8.6E-01 | | 0.97 | | 0.89 | | CCNH/PSMC3/RUNX1/TCF12 | | 4 | |
| R-HSA-6791226 | | Major pathway of rRNA processing in the nucleolus and cytosol | | | 6/505 | | 184/10867 | | 8.6E-01 | | 0.97 | | 0.89 | | FCF1/RPL32/RPL36AL/RPS14/RPS21/RPS9 | | 6 | |
| R-HSA-1980143 | | Signaling by NOTCH1 | | | 2/505 | | 74/10867 | | 8.6E-01 | | 0.97 | | 0.89 | | ADAM10/NUMB | | 2 | |
| R-HSA-606279 | | Deposition of new CENPA-containing nucleosomes at the centromere | | | 2/505 | | 74/10867 | | 8.6E-01 | | 0.97 | | 0.89 | | CENPA/CENPQ | | 2 | |
| R-HSA-774815 | | Nucleosome assembly | | | 2/505 | | 74/10867 | | 8.6E-01 | | 0.97 | | 0.89 | | CENPA/CENPQ | | 2 | |
| R-HSA-159234 | | Transport of Mature mRNAs Derived from Intronless Transcripts | | | 1/505 | | 42/10867 | | 8.6E-01 | | 0.97 | | 0.89 | | RANBP2 | | 1 | |
| R-HSA-163841 | | Gamma carboxylation. hypusine formation and arylsulfatase activation | | | 1/505 | | 42/10867 | | 8.6E-01 | | 0.97 | | 0.89 | | PROS1 | | 1 | |
| R-HSA-9711123 | | Cellular response to chemical stress | | | 5/505 | | 160/10867 | | 8.7E-01 | | 0.97 | | 0.90 | | ATP7A/COX7C/CUL3/PSMC3/TXNIP | | 5 | |
| R-HSA-975634 | | Retinoid metabolism and transport | | | 1/505 | | 44/10867 | | 8.8E-01 | | 0.98 | | 0.90 | | LRP10 | | 1 | |
| R-HSA-3108214 | | SUMOylation of DNA damage response and repair proteins | | | 2/505 | | 77/10867 | | 8.8E-01 | | 0.98 | | 0.90 | | BLM/RANBP2 | | 2 | |
| R-HSA-427413 | | NoRC negatively regulates rRNA expression | | | 3/505 | | 107/10867 | | 8.8E-01 | | 0.98 | | 0.91 | | BAZ2A/CCNH/DNMT1 | | 3 | |
| R-HSA-5619084 | | ABC transporter disorders | | | 2/505 | | 78/10867 | | 8.8E-01 | | 0.98 | | 0.91 | | PSMC3/SEL1L | | 2 | |
| R-HSA-5693606 | | DNA Double Strand Break Response | | | 2/505 | | 78/10867 | | 8.8E-01 | | 0.98 | | 0.91 | | KPNA2/RAD50 | | 2 | |
| R-HSA-1912422 | | Pre-NOTCH Expression and Processing | | | 3/505 | | 109/10867 | | 8.9E-01 | | 0.99 | | 0.91 | | ATP2A2/RUNX1/SEL1L | | 3 | |
| R-HSA-71291 | | Metabolism of amino acids and derivatives | | | 13/505 | | 374/10867 | | 8.9E-01 | | 0.99 | | 0.91 | | IARS1/OAZ1/PHYKPL/PPM1K/PSMC3/RPL32/RPL36AL/RPS14/RPS21/RPS9/SERINC1/SERINC5/SLC6A12 | | 13 | |
| R-HSA-8868773 | | rRNA processing in the nucleus and cytosol | | | 6/505 | | 194/10867 | | 8.9E-01 | | 0.99 | | 0.91 | | FCF1/RPL32/RPL36AL/RPS14/RPS21/RPS9 | | 6 | |
| R-HSA-190828 | | Gap junction trafficking | | | 1/505 | | 47/10867 | | 8.9E-01 | | 0.99 | | 0.91 | | CLTC | | 1 | |
| R-HSA-4570464 | | SUMOylation of RNA binding proteins | | | 1/505 | | 47/10867 | | 8.9E-01 | | 0.99 | | 0.91 | | RANBP2 | | 1 | |
| R-HSA-3247509 | | Chromatin modifying enzymes | | | 9/505 | | 274/10867 | | 9.0E-01 | | 0.99 | | 0.91 | | ARID5B/BRWD1/KDM5B/KDM6A/MBD3/MORF4L1/MTA3/PHF20/SMARCC1 | | 9 | |
| R-HSA-4839726 | | Chromatin organization | | | 9/505 | | 274/10867 | | 9.0E-01 | | 0.99 | | 0.91 | | ARID5B/BRWD1/KDM5B/KDM6A/MBD3/MORF4L1/MTA3/PHF20/SMARCC1 | | 9 | |
| R-HSA-69481 | | G2/M Checkpoints | | | 5/505 | | 168/10867 | | 9.0E-01 | | 0.99 | | 0.91 | | BLM/CCNB2/PSMC3/RAD50/RFC5 | | 5 | |
| R-HSA-211859 | | Biological oxidations | | | 7/505 | | 222/10867 | | 9.0E-01 | | 0.99 | | 0.91 | | ADH1A/ADH1C/ALDH1A1/CYP4F12/FDXR/GGCT/SLC35D1 | | 7 | |
| R-HSA-2559582 | | Senescence-Associated Secretory Phenotype (SASP) | | | 3/505 | | 112/10867 | | 9.0E-01 | | 0.99 | | 0.91 | | CDKN1A/CXCL8/IGFBP7 | | 3 | |
| R-HSA-6806667 | | Metabolism of fat-soluble vitamins | | | 1/505 | | 48/10867 | | 9.0E-01 | | 0.99 | | 0.91 | | LRP10 | | 1 | |
| R-HSA-2871837 | | FCERI mediated NF-kB activation | | | 2/505 | | 82/10867 | | 9.0E-01 | | 0.99 | | 0.91 | | PSMC3/TAB1 | | 2 | |
| R-HSA-5621481 | | C-type lectin receptors (CLRs) | | | 4/505 | | 142/10867 | | 9.0E-01 | | 0.99 | | 0.91 | | FYN/ITPR1/PSMC3/TAB1 | | 4 | |
| R-HSA-68875 | | Mitotic Prophase | | | 4/505 | | 142/10867 | | 9.0E-01 | | 0.99 | | 0.91 | | CCNB2/RANBP2/SMC4/VRK1 | | 4 | |
| R-HSA-1236978 | | Cross-presentation of soluble exogenous antigens (endosomes) | | | 1/505 | | 49/10867 | | 9.0E-01 | | 0.99 | | 0.91 | | PSMC3 | | 1 | |
| R-HSA-9012852 | | Signaling by NOTCH3 | | | 1/505 | | 49/10867 | | 9.0E-01 | | 0.99 | | 0.91 | | ADAM10 | | 1 | |
| R-HSA-1474165 | | Reproduction | | | 4/505 | | 143/10867 | | 9.0E-01 | | 0.99 | | 0.91 | | BLM/HVCN1/RAD50/TERF2IP | | 4 | |
| R-HSA-211733 | | Regulation of activated PAK-2p34 by proteasome mediated degradation | | | 1/505 | | 50/10867 | | 9.1E-01 | | 0.99 | | 0.91 | | PSMC3 | | 1 | |
| R-HSA-69002 | | DNA Replication Pre-Initiation | | | 2/505 | | 85/10867 | | 9.1E-01 | | 0.99 | | 0.91 | | GMNN/PSMC3 | | 2 | |
| R-HSA-6794362 | | Protein-protein interactions at synapses | | | 2/505 | | 86/10867 | | 9.1E-01 | | 0.99 | | 0.91 | | NLGN2/NRXN3 | | 2 | |
| R-HSA-912446 | | Meiotic recombination | | | 2/505 | | 86/10867 | | 9.1E-01 | | 0.99 | | 0.91 | | BLM/RAD50 | | 2 | |
| R-HSA-171306 | | Packaging Of Telomere Ends | | | 1/505 | | 52/10867 | | 9.2E-01 | | 0.99 | | 0.91 | | TERF2IP | | 1 | |
| R-HSA-180534 | | Vpu mediated degradation of CD4 | | | 1/505 | | 52/10867 | | 9.2E-01 | | 0.99 | | 0.91 | | PSMC3 | | 1 | |
| R-HSA-349425 | | Autodegradation of the E3 ubiquitin ligase COP1 | | | 1/505 | | 52/10867 | | 9.2E-01 | | 0.99 | | 0.91 | | PSMC3 | | 1 | |
| R-HSA-69601 | | Ubiquitin Mediated Degradation of Phosphorylated Cdc25A | | | 1/505 | | 52/10867 | | 9.2E-01 | | 0.99 | | 0.91 | | PSMC3 | | 1 | |
| R-HSA-69610 | | p53-Independent DNA Damage Response | | | 1/505 | | 52/10867 | | 9.2E-01 | | 0.99 | | 0.91 | | PSMC3 | | 1 | |
| R-HSA-69613 | | p53-Independent G1/S DNA damage checkpoint | | | 1/505 | | 52/10867 | | 9.2E-01 | | 0.99 | | 0.91 | | PSMC3 | | 1 | |
| R-HSA-1500620 | | Meiosis | | | 3/505 | | 118/10867 | | 9.2E-01 | | 0.99 | | 0.91 | | BLM/RAD50/TERF2IP | | 3 | |
| R-HSA-5389840 | | Mitochondrial translation elongation | | | 2/505 | | 87/10867 | | 9.2E-01 | | 0.99 | | 0.91 | | GFM1/MRPS2 | | 2 | |
| R-HSA-72312 | | rRNA processing | | | 6/505 | | 204/10867 | | 9.2E-01 | | 0.99 | | 0.91 | | FCF1/RPL32/RPL36AL/RPS14/RPS21/RPS9 | | 6 | |
| R-HSA-169911 | | Regulation of Apoptosis | | | 1/505 | | 53/10867 | | 9.2E-01 | | 0.99 | | 0.91 | | PSMC3 | | 1 | |
| R-HSA-191859 | | snRNP Assembly | | | 1/505 | | 53/10867 | | 9.2E-01 | | 0.99 | | 0.91 | | RANBP2 | | 1 | |
| R-HSA-194441 | | Metabolism of non-coding RNA | | | 1/505 | | 53/10867 | | 9.2E-01 | | 0.99 | | 0.91 | | RANBP2 | | 1 | |
| R-HSA-180585 | | Vif-mediated degradation of APOBEC3G | | | 1/505 | | 54/10867 | | 9.2E-01 | | 0.99 | | 0.91 | | PSMC3 | | 1 | |
| R-HSA-5620924 | | Intraflagellar transport | | | 1/505 | | 54/10867 | | 9.2E-01 | | 0.99 | | 0.91 | | KIF3B | | 1 | |
| R-HSA-9604323 | | Negative regulation of NOTCH4 signaling | | | 1/505 | | 54/10867 | | 9.2E-01 | | 0.99 | | 0.91 | | PSMC3 | | 1 | |
| R-HSA-9662361 | | Sensory processing of sound by outer hair cells of the cochlea | | | 1/505 | | 54/10867 | | 9.2E-01 | | 0.99 | | 0.91 | | MSN | | 1 | |
| R-HSA-157118 | | Signaling by NOTCH | | | 7/505 | | 236/10867 | | 9.3E-01 | | 0.99 | | 0.91 | | ADAM10/ATP2A2/FLT4/NUMB/PSMC3/RUNX1/SEL1L | | 7 | |
| R-HSA-1638091 | | Heparan sulfate/heparin (HS-GAG) metabolism | | | 1/505 | | 55/10867 | | 9.3E-01 | | 0.99 | | 0.91 | | VCAN | | 1 | |
| R-HSA-174113 | | SCF-beta-TrCP mediated degradation of Emi1 | | | 1/505 | | 55/10867 | | 9.3E-01 | | 0.99 | | 0.91 | | PSMC3 | | 1 | |
| R-HSA-3371497 | | HSP90 chaperone cycle for steroid hormone receptors (SHR) in the presence of ligand | | | 1/505 | | 55/10867 | | 9.3E-01 | | 0.99 | | 0.91 | | HSP90AA1 | | 1 | |
| R-HSA-429914 | | Deadenylation-dependent mRNA decay | | | 1/505 | | 55/10867 | | 9.3E-01 | | 0.99 | | 0.91 | | DDX6 | | 1 | |
| R-HSA-4641257 | | Degradation of AXIN | | | 1/505 | | 55/10867 | | 9.3E-01 | | 0.99 | | 0.91 | | PSMC3 | | 1 | |
| R-HSA-71406 | | Pyruvate metabolism and Citric Acid (TCA) cycle | | | 1/505 | | 55/10867 | | 9.3E-01 | | 0.99 | | 0.91 | | BSG | | 1 | |
| R-HSA-8941858 | | Regulation of RUNX3 expression and activity | | | 1/505 | | 55/10867 | | 9.3E-01 | | 0.99 | | 0.91 | | PSMC3 | | 1 | |
| R-HSA-69052 | | Switching of origins to a post-replicative state | | | 2/505 | | 91/10867 | | 9.3E-01 | | 0.99 | | 0.91 | | CCNE2/PSMC3 | | 2 | |
| R-HSA-70326 | | Glucose metabolism | | | 2/505 | | 91/10867 | | 9.3E-01 | | 0.99 | | 0.91 | | PGM2L1/RANBP2 | | 2 | |
| R-HSA-73772 | | RNA Polymerase I Promoter Escape | | | 2/505 | | 91/10867 | | 9.3E-01 | | 0.99 | | 0.91 | | CBX3/CCNH | | 2 | |
| R-HSA-72766 | | Translation | | | 9/505 | | 291/10867 | | 9.3E-01 | | 0.99 | | 0.91 | | FARSA/GFM1/IARS1/MRPS2/RPL32/RPL36AL/RPS14/RPS21/RPS9 | | 9 | |
| R-HSA-110330 | | Recognition and association of DNA glycosylase with site containing an affected purine | | | 1/505 | | 56/10867 | | 9.3E-01 | | 0.99 | | 0.91 | | TERF2IP | | 1 | |
| R-HSA-110331 | | Cleavage of the damaged purine | | | 1/505 | | 56/10867 | | 9.3E-01 | | 0.99 | | 0.91 | | TERF2IP | | 1 | |
| R-HSA-450408 | | AUF1 (hnRNP D0) binds and destabilizes mRNA | | | 1/505 | | 56/10867 | | 9.3E-01 | | 0.99 | | 0.91 | | PSMC3 | | 1 | |
| R-HSA-73927 | | Depurination | | | 1/505 | | 56/10867 | | 9.3E-01 | | 0.99 | | 0.91 | | TERF2IP | | 1 | |
| R-HSA-6784531 | | tRNA processing in the nucleus | | | 1/505 | | 57/10867 | | 9.3E-01 | | 0.99 | | 0.91 | | RANBP2 | | 1 | |
| R-HSA-5368287 | | Mitochondrial translation | | | 2/505 | | 93/10867 | | 9.3E-01 | | 0.99 | | 0.91 | | GFM1/MRPS2 | | 2 | |
| R-HSA-3214815 | | HDACs deacetylate histones | | | 2/505 | | 94/10867 | | 9.4E-01 | | 0.99 | | 0.91 | | MBD3/MTA3 | | 2 | |
| R-HSA-2644602 | | Signaling by NOTCH1 PEST Domain Mutants in Cancer | | | 1/505 | | 58/10867 | | 9.4E-01 | | 0.99 | | 0.91 | | ADAM10 | | 1 | |
| R-HSA-2644603 | | Signaling by NOTCH1 in Cancer | | | 1/505 | | 58/10867 | | 9.4E-01 | | 0.99 | | 0.91 | | ADAM10 | | 1 | |
| R-HSA-2644606 | | Constitutive Signaling by NOTCH1 PEST Domain Mutants | | | 1/505 | | 58/10867 | | 9.4E-01 | | 0.99 | | 0.91 | | ADAM10 | | 1 | |
| R-HSA-2894858 | | Signaling by NOTCH1 HD+PEST Domain Mutants in Cancer | | | 1/505 | | 58/10867 | | 9.4E-01 | | 0.99 | | 0.91 | | ADAM10 | | 1 | |
| R-HSA-2894862 | | Constitutive Signaling by NOTCH1 HD+PEST Domain Mutants | | | 1/505 | | 58/10867 | | 9.4E-01 | | 0.99 | | 0.91 | | ADAM10 | | 1 | |
| R-HSA-6803157 | | Antimicrobial peptides | | | 2/505 | | 95/10867 | | 9.4E-01 | | 0.99 | | 0.91 | | ATP7A/PLA2G2A | | 2 | |
| R-HSA-5676590 | | NIK-->noncanonical NF-kB signaling | | | 1/505 | | 59/10867 | | 9.4E-01 | | 0.99 | | 0.91 | | PSMC3 | | 1 | |
| R-HSA-5688426 | | Deubiquitination | | | 9/505 | | 298/10867 | | 9.4E-01 | | 0.99 | | 0.91 | | APC/CCP110/FKBP8/PSMC3/TAB1/TGFBR1/TGFBR2/USP3/YY1 | | 9 | |
| R-HSA-5607761 | | Dectin-1 mediated noncanonical NF-kB signaling | | | 1/505 | | 60/10867 | | 9.4E-01 | | 0.99 | | 0.91 | | PSMC3 | | 1 | |
| R-HSA-8936459 | | RUNX1 regulates genes involved in megakaryocyte differentiation and platelet function | | | 2/505 | | 97/10867 | | 9.4E-01 | | 0.99 | | 0.91 | | RUNX1/THBS1 | | 2 | |
| R-HSA-110328 | | Recognition and association of DNA glycosylase with site containing an affected pyrimidine | | | 1/505 | | 61/10867 | | 9.5E-01 | | 0.99 | | 0.91 | | TERF2IP | | 1 | |
| R-HSA-110329 | | Cleavage of the damaged pyrimidine | | | 1/505 | | 61/10867 | | 9.5E-01 | | 0.99 | | 0.91 | | TERF2IP | | 1 | |
| R-HSA-73928 | | Depyrimidination | | | 1/505 | | 61/10867 | | 9.5E-01 | | 0.99 | | 0.91 | | TERF2IP | | 1 | |
| R-HSA-5689880 | | Ub-specific processing proteases | | | 6/505 | | 220/10867 | | 9.5E-01 | | 0.99 | | 0.92 | | CCP110/FKBP8/PSMC3/TAB1/TGFBR1/USP3 | | 6 | |
| R-HSA-73929 | | Base-Excision Repair. AP Site Formation | | | 1/505 | | 63/10867 | | 9.5E-01 | | 1 | | 0.92 | | TERF2IP | | 1 | |
| R-HSA-174084 | | Autodegradation of Cdh1 by Cdh1:APC/C | | | 1/505 | | 64/10867 | | 9.5E-01 | | 1 | | 0.92 | | PSMC3 | | 1 | |
| R-HSA-5334118 | | DNA methylation | | | 1/505 | | 65/10867 | | 9.6E-01 | | 1 | | 0.92 | | DNMT1 | | 1 | |
| R-HSA-1296071 | | Potassium Channels | | | 2/505 | | 103/10867 | | 9.6E-01 | | 1 | | 0.92 | | GNG12/KCNK10 | | 2 | |
| R-HSA-9645723 | | Diseases of programmed cell death | | | 2/505 | | 103/10867 | | 9.6E-01 | | 1 | | 0.92 | | DNMT1/FOXO3 | | 2 | |
| R-HSA-983231 | | Factors involved in megakaryocyte development and platelet production | | | 4/505 | | 168/10867 | | 9.6E-01 | | 1 | | 0.92 | | KIF18B/KIF3B/MICAL1/ZFPM2 | | 4 | |
| R-HSA-211000 | | Gene Silencing by RNA | | | 3/505 | | 138/10867 | | 9.6E-01 | | 1 | | 0.92 | | HSP90AA1/RANBP2/TSN | | 3 | |
| R-HSA-1169091 | | Activation of NF-kappaB in B cells | | | 1/505 | | 67/10867 | | 9.6E-01 | | 1 | | 0.92 | | PSMC3 | | 1 | |
| R-HSA-174154 | | APC/C:Cdc20 mediated degradation of Securin | | | 1/505 | | 68/10867 | | 9.6E-01 | | 1 | | 0.92 | | PSMC3 | | 1 | |
| R-HSA-427359 | | SIRT1 negatively regulates rRNA expression | | | 1/505 | | 68/10867 | | 9.6E-01 | | 1 | | 0.92 | | SIRT1 | | 1 | |
| R-HSA-9670095 | | Inhibition of DNA recombination at telomere | | | 1/505 | | 68/10867 | | 9.6E-01 | | 1 | | 0.92 | | TERF2IP | | 1 | |
| R-HSA-5693571 | | Nonhomologous End-Joining (NHEJ) | | | 1/505 | | 69/10867 | | 9.6E-01 | | 1 | | 0.92 | | RAD50 | | 1 | |
| R-HSA-156580 | | Phase II - Conjugation of compounds | | | 2/505 | | 109/10867 | | 9.7E-01 | | 1 | | 0.92 | | GGCT/SLC35D1 | | 2 | |
| R-HSA-4551638 | | SUMOylation of chromatin organization proteins | | | 1/505 | | 71/10867 | | 9.7E-01 | | 1 | | 0.92 | | RANBP2 | | 1 | |
| R-HSA-68949 | | Orc1 removal from chromatin | | | 1/505 | | 71/10867 | | 9.7E-01 | | 1 | | 0.92 | | PSMC3 | | 1 | |
| R-HSA-977225 | | Amyloid fiber formation | | | 2/505 | | 111/10867 | | 9.7E-01 | | 1 | | 0.92 | | ADAM10/CST3 | | 2 | |
| R-HSA-174184 | | Cdc20:Phospho-APC/C mediated degradation of Cyclin A | | | 1/505 | | 73/10867 | | 9.7E-01 | | 1 | | 0.92 | | PSMC3 | | 1 | |
| R-HSA-9710421 | | Defective pyroptosis | | | 1/505 | | 73/10867 | | 9.7E-01 | | 1 | | 0.92 | | DNMT1 | | 1 | |
| R-HSA-174178 | | APC/C:Cdh1 mediated degradation of Cdc20 and other APC/C:Cdh1 targeted proteins in late mitosis/early G1 | | | 1/505 | | 74/10867 | | 9.7E-01 | | 1 | | 0.92 | | PSMC3 | | 1 | |
| R-HSA-179419 | | APC:Cdc20 mediated degradation of cell cycle proteins prior to satisfation of the cell cycle checkpoint | | | 1/505 | | 74/10867 | | 9.7E-01 | | 1 | | 0.92 | | PSMC3 | | 1 | |
| R-HSA-2299718 | | Condensation of Prophase Chromosomes | | | 1/505 | | 74/10867 | | 9.7E-01 | | 1 | | 0.92 | | SMC4 | | 1 | |
| R-HSA-176409 | | APC/C:Cdc20 mediated degradation of mitotic proteins | | | 1/505 | | 76/10867 | | 9.7E-01 | | 1 | | 0.92 | | PSMC3 | | 1 | |
| R-HSA-9610379 | | HCMV Late Events | | | 2/505 | | 116/10867 | | 9.7E-01 | | 1 | | 0.92 | | CHMP3/RANBP2 | | 2 | |
| R-HSA-176814 | | Activation of APC/C and APC/C:Cdc20 mediated degradation of mitotic proteins | | | 1/505 | | 77/10867 | | 9.7E-01 | | 1 | | 0.92 | | PSMC3 | | 1 | |
| R-HSA-5693565 | | Recruitment and ATM-mediated phosphorylation of repair and signaling proteins at DNA double strand breaks | | | 1/505 | | 77/10867 | | 9.7E-01 | | 1 | | 0.92 | | RAD50 | | 1 | |
| R-HSA-1221632 | | Meiotic synapsis | | | 1/505 | | 79/10867 | | 9.8E-01 | | 1 | | 0.92 | | TERF2IP | | 1 | |
| R-HSA-3214858 | | RMTs methylate histone arginines | | | 1/505 | | 79/10867 | | 9.8E-01 | | 1 | | 0.92 | | SMARCC1 | | 1 | |
| R-HSA-5617472 | | Activation of anterior HOX genes in hindbrain development during early embryogenesis | | | 2/505 | | 121/10867 | | 9.8E-01 | | 1 | | 0.92 | | KDM6A/YY1 | | 2 | |
| R-HSA-5619507 | | Activation of HOX genes during differentiation | | | 2/505 | | 121/10867 | | 9.8E-01 | | 1 | | 0.92 | | KDM6A/YY1 | | 2 | |
| R-HSA-176408 | | Regulation of APC/C activators between G1/S and early anaphase | | | 1/505 | | 81/10867 | | 9.8E-01 | | 1 | | 0.92 | | PSMC3 | | 1 | |
| R-HSA-1168372 | | Downstream signaling events of B Cell Receptor (BCR) | | | 1/505 | | 83/10867 | | 9.8E-01 | | 1 | | 0.92 | | PSMC3 | | 1 | |
| R-HSA-9609646 | | HCMV Infection | | | 3/505 | | 160/10867 | | 9.8E-01 | | 1 | | 0.92 | | CHMP3/RANBP2/TRIM28 | | 3 | |
| R-HSA-5368286 | | Mitochondrial translation initiation | | | 1/505 | | 87/10867 | | 9.8E-01 | | 1 | | 0.92 | | MRPS2 | | 1 | |
| R-HSA-5419276 | | Mitochondrial translation termination | | | 1/505 | | 87/10867 | | 9.8E-01 | | 1 | | 0.92 | | MRPS2 | | 1 | |
| R-HSA-174143 | | APC/C-mediated degradation of cell cycle proteins | | | 1/505 | | 88/10867 | | 9.9E-01 | | 1 | | 0.92 | | PSMC3 | | 1 | |
| R-HSA-453276 | | Regulation of mitotic cell cycle | | | 1/505 | | 88/10867 | | 9.9E-01 | | 1 | | 0.92 | | PSMC3 | | 1 | |
| R-HSA-6809371 | | Formation of the cornified envelope | | | 2/505 | | 130/10867 | | 9.9E-01 | | 1 | | 0.92 | | KRT19/PERP | | 2 | |
| R-HSA-375276 | | Peptide ligand-binding receptors | | | 4/505 | | 201/10867 | | 9.9E-01 | | 1 | | 0.92 | | ACKR3/CCRL2/CXCL8/PSAP | | 4 | |
| R-HSA-5250924 | | B-WICH complex positively regulates rRNA expression | | | 1/505 | | 91/10867 | | 9.9E-01 | | 1 | | 0.92 | | GSK3B | | 1 | |
| R-HSA-9609690 | | HCMV Early Events | | | 2/505 | | 135/10867 | | 9.9E-01 | | 1 | | 0.92 | | RANBP2/TRIM28 | | 2 | |
| R-HSA-163125 | | Post-translational modification: synthesis of GPI-anchored proteins | | | 1/505 | | 93/10867 | | 9.9E-01 | | 1 | | 0.92 | | CNTN3 | | 1 | |
| R-HSA-1912408 | | Pre-NOTCH Transcription and Translation | | | 1/505 | | 93/10867 | | 9.9E-01 | | 1 | | 0.92 | | RUNX1 | | 1 | |
| R-HSA-5625740 | | RHO GTPases activate PKNs | | | 1/505 | | 95/10867 | | 9.9E-01 | | 1 | | 0.92 | | RHOB | | 1 | |
| R-HSA-3214847 | | HATs acetylate histones | | | 2/505 | | 142/10867 | | 9.9E-01 | | 1 | | 0.92 | | MORF4L1/PHF20 | | 2 | |
| R-HSA-6807878 | | COPI-mediated anterograde transport | | | 1/505 | | 101/10867 | | 9.9E-01 | | 1 | | 0.92 | | ARCN1 | | 1 | |
| R-HSA-5668541 | | TNFR2 non-canonical NF-kB pathway | | | 1/505 | | 102/10867 | | 9.9E-01 | | 1 | | 0.92 | | PSMC3 | | 1 | |
| R-HSA-611105 | | Respiratory electron transport | | | 1/505 | | 103/10867 | | 9.9E-01 | | 1 | | 0.92 | | COX7C | | 1 | |
| R-HSA-500792 | | GPCR ligand binding | | | 12/505 | | 467/10867 | | 9.9E-01 | | 1 | | 0.92 | | ACKR3/ADRA2A/CALCRL/CCRL2/CXCL8/GNG12/HRH1/P2RY11/PSAP/RAMP2/WNT5A/WNT7B | | 12 | |
| R-HSA-2187338 | | Visual phototransduction | | | 1/505 | | 105/10867 | | 9.9E-01 | | 1 | | 0.92 | | LRP10 | | 1 | |
| R-HSA-5578749 | | Transcriptional regulation by small RNAs | | | 1/505 | | 106/10867 | | 9.9E-01 | | 1 | | 0.92 | | RANBP2 | | 1 | |
| R-HSA-72306 | | tRNA processing | | | 1/505 | | 107/10867 | | 9.9E-01 | | 1 | | 0.92 | | RANBP2 | | 1 | |
| R-HSA-373076 | | Class A/1 (Rhodopsin-like receptors) | | | 7/505 | | 335/10867 | | 1 | | 1 | | 0.92 | | ACKR3/ADRA2A/CCRL2/CXCL8/HRH1/P2RY11/PSAP | | 7 | |
| R-HSA-1989781 | | PPARA activates gene expression | | | 1/505 | | 117/10867 | | 1 | | 1 | | 0.92 | | SP1 | | 1 | |
| R-HSA-400206 | | Regulation of lipid metabolism by PPARalpha | | | 1/505 | | 119/10867 | | 1 | | 1 | | 0.92 | | SP1 | | 1 | |
| R-HSA-2559580 | | Oxidative Stress Induced Senescence | | | 1/505 | | 125/10867 | | 1 | | 1 | | 0.92 | | MAP3K5 | | 1 | |
| R-HSA-163200 | | Respiratory electron transport. ATP synthesis by chemiosmotic coupling. and heat production by uncoupling proteins. | | | 1/505 | | 127/10867 | | 1 | | 1 | | 0.92 | | COX7C | | 1 | |
| R-HSA-1428517 | | The citric acid (TCA) cycle and respiratory electron transport | | | 2/505 | | 178/10867 | | 1 | | 1 | | 0.92 | | BSG/COX7C | | 2 | |
| R-HSA-6805567 | | Keratinization | | | 2/505 | | 214/10867 | | 1 | | 1 | | 0.92 | | KRT19/PERP | | 2 | |
