# Supplementary material for: Potential biomarkers as a predictive factor of response to primary chemotherapy in breast cancer patients
Source: Braz J Med Biol Res. 2024 Oct 7;57:e13599. doi: 10.1590/1414-431X2024e13599 (PMC11463908; doi:10.1590/1414-431X2024e13599)
Supplement: Supplementary file 1 [file 1414-431X-bjmbr-57-e13599-suppl.zip › 13559_Supplementary Table S4.docx]

**Supplementary Table S4.** Signaling Pathways in Breast Cancer Estrogen Receptor-Positive Patients Using the ReactomePA R Package.

| **ID** | **Description** | **GeneRatio** | **BgRatio** | **Pvalue** | **P.adjust** | **Qvalue** | **geneID** | **Count** |
| --- | --- | --- | --- | --- | --- | --- | --- | --- |
| R-HSA-1474244 | Extracellular matrix organization | 41/597 | 301/10867 | 5.8E-08 | 4.1E-05 | 3.7E-05 | ADAM12/ADAM19/ADAMTS2/BMP2/CAPN5/COL10A1/COL14A1/COL17A1/COL18A1/COL1A1/COL1A2/COL3A1/COL5A1/COL5A2/COL6A3/COLGALT1/DCN/DST/FBN1/FBN2/FGF2/ITGA11/ITGB1/ITGB2/ITGB3/LAMB3/LAMC1/LOX/LOXL2/MMP14/NTN4/P3H1/PCOLCE2/PHYKPL/PRKCA/PXDN/SDC1/SERPINH1/SPARC/TIMP2/ VCAN | 41 |
| R-HSA-1474290 | Collagen formation | 20/597 | 90/10867 | 6.2E-08 | 4.1E-05 | 3.7E-05 | ADAMTS2/COL10A1/COL14A1/COL17A1/COL18A1/COL1A1/COL1A2/COL3A1/COL5A1/COL5A2/COL6A3/COLGALT1/DST/LAMB3/LOX/LOXL2/P3H1/PCOLCE2/PXDN/SERPINH1 | 20 |
| R-HSA-2022090 | Assembly of collagen fibrils and other multimeric structures | 15/597 | 61/10867 | 7.0E-07 | 2.7E-04 | 2.5E-04 | COL10A1/COL14A1/COL17A1/COL18A1/COL1A1/COL1A2/COL3A1/COL5A1/COL5A2/COL6A3/DST/LAMB3/LOX/LOXL2/PXDN | 15 |
| R-HSA-3000170 | Syndecan interactions | 10/597 | 27/10867 | 8.4E-07 | 2.7E-04 | 2.5E-04 | COL1A1/COL1A2/COL3A1/COL5A1/COL5A2/FGF2/ITGB1/ITGB3/PRKCA/SDC1 | 10 |
| R-HSA-1650814 | Collagen biosynthesis and modifying enzymes | 15/597 | 67/10867 | 2.5E-06 | 5.4E-04 | 4.9E-04 | ADAMTS2/COL10A1/COL14A1/COL17A1/COL18A1/COL1A1/COL1A2/COL3A1/COL5A1/COL5A2/COL6A3/COLGALT1/P3H1/PCOLCE2/SERPINH1 | 15 |
| R-HSA-3000171 | Non-integrin membrane-ECM interactions | 14/597 | 59/10867 | 2.6E-06 | 5.4E-04 | 4.9E-04 | COL10A1/COL1A1/COL1A2/COL3A1/COL5A1/COL5A2/FGF2/ITGB1/ITGB3/LAMB3/LAMC1/NTN4/PRKCA/SDC1 | 14 |
| R-HSA-449147 | Signaling by Interleukins | 50/597 | 462/10867 | 2.9E-06 | 5.4E-04 | 4.9E-04 | ANXA1/CCL5/COL1A2/CSF2RA/CXCL2/DUSP6/FGF2/FOXO1/FOXO3/FYN/HCK/HNRNPA2B1/HSP90AA1/HSP90B1/IL10RA/IL15RA/IL18R1/IL1RAP/IL21R/IL7/IRS2/ITGB1/ITGB2/JUNB/LCK/LIFR/LMNB1/MAOA/MAP3K8/MSN/OSMR/PIK3R1/PSMD11/PSMD7/PSMD8/PTGS2/RALA/RAPGEF1/RHOU/SDC1/SOCS5/STAT4/STAT6/STXBP2/TCP1/TNFRSF1B/TNIP2/TYK2/VAMP7/VRK3 | 50 |
| R-HSA-1257604 | PIP3 activates AKT signaling | 33/597 | 267/10867 | 1.0E-05 | 1.6E-03 | 1.5E-03 | CBX2/EGR1/ERBB3/EZH2/FGF1/FGF18/FGF2/FOXO1/FOXO3/FYN/GSK3A/GSK3B/HDAC7/IL1RAP/IRS2/LAMTOR4/LCK/MBD3/MET/NEDD4/PDGFRB/PIK3R1/PIP4K2C/PPARG/PSMD11/PSMD7/PSMD8/PTEN/RAC2/RING1/RPS6KB2/RRAGC/SALL4 | 33 |
| R-HSA-6785807 | Interleukin-4 and Interleukin-13 signaling | 18/597 | 108/10867 | 2.2E-05 | 2.8E-03 | 2.5E-03 | ANXA1/COL1A2/FGF2/FOXO1/FOXO3/HSP90AA1/HSP90B1/ITGB1/ITGB2/JUNB/MAOA/PIK3R1/PTGS2/RHOU/SOCS5/STAT6/TNFRSF1B/TYK2 | 18 |
| R-HSA-8874081 | MET activates PTK2 signaling | 9/597 | 30/10867 | 2.2E-05 | 2.8E-03 | 2.5E-03 | COL1A1/COL1A2/COL3A1/COL5A1/COL5A2/ITGB1/LAMB3/LAMC1/MET | 9 |
| R-HSA-9006925 | Intracellular signaling by second messengers | 35/597 | 309/10867 | 3.6E-05 | 4.3E-03 | 3.8E-03 | CBX2/EGR1/ERBB3/EZH2/FGF1/FGF18/FGF2/FOXO1/FOXO3/FYN/GSK3A/GSK3B/HDAC7/IL1RAP/IRS2/KPNA2/LAMTOR4/LCK/MBD3/MET/NEDD4/PDGFRB/PIK3R1/PIP4K2C/PPARG/PRKCA/PSMD11/PSMD7/PSMD8/PTEN/RAC2/RING1/RPS6KB2/RRAGC/SALL4 | 35 |
| R-HSA-2219528 | PI3K/AKT Signaling in Cancer | 17/597 | 104/10867 | 4.7E-05 | 5.1E-03 | 4.6E-03 | ERBB3/FGF1/FGF18/FGF2/FOXO1/FOXO3/FYN/GSK3A/GSK3B/IRS2/LCK/MET/PDGFRB/PIK3R1/PTEN/RAC2/RPS6KB2 | 17 |
| R-HSA-8875878 | MET promotes cell motility | 10/597 | 41/10867 | 5.5E-05 | 5.6E-03 | 5.0E-03 | COL1A1/COL1A2/COL3A1/COL5A1/COL5A2/ITGB1/LAMB3/LAMC1/MET/RAPGEF1 | 10 |
| R-HSA-5663220 | RHO GTPases Activate Formins | 20/597 | 140/10867 | 7.6E-05 | 7.2E-03 | 6.4E-03 | AURKB/BUB1/CDCA8/CENPA/CENPK/CENPQ/CENPT/DAAM1/DIAPH2/ITGB1/MAD2L1/MAPRE1/NUP160/RHOB/SGO1/SPDL1/SRF/SRGAP2/TUBA1C/ZWILCH | 20 |
| R-HSA-8948216 | Collagen chain trimerization | 10/597 | 44/10867 | 1.1E-04 | 9.2E-03 | 8.3E-03 | COL10A1/COL14A1/COL17A1/COL18A1/COL1A1/COL1A2/COL3A1/COL5A1/COL5A2/COL6A3 | 10 |
| R-HSA-166520 | Signaling by NTRKs | 19/597 | 134/10867 | 1.3E-04 | 1.0E-02 | 9.3E-03 | CLTC/DUSP6/EGR1/F3/FYN/ID1/ID3/IRS2/JUNB/MEF2D/NELFB/NRAS/PCSK5/PIK3R1/RALA/RAPGEF1/RRAD/SRF/VRK3 | 19 |
| R-HSA-1442490 | Collagen degradation | 12/597 | 64/10867 | 1.6E-04 | 1.2E-02 | 1.1E-02 | COL10A1/COL14A1/COL17A1/COL18A1/COL1A1/COL1A2/COL3A1/COL5A1/COL5A2/COL6A3/MMP14/PHYKPL | 12 |
| R-HSA-69620 | Cell Cycle Checkpoints | 32/597 | 294/10867 | 1.6E-04 | 1.2E-02 | 1.1E-02 | ANAPC2/AURKB/BLM/BUB1/CCNA2/CCNB2/CCNE2/CDC25A/CDCA8/CENPA/CENPK/CENPQ/CENPT/DNA2/EXO1/H2AX/H2BC13/H2BC5/MAD2L1/MAPRE1/MCM3/MCM4/NUP160/PSMD11/PSMD7/PSMD8/RFC5/RNF168/SGO1/SPDL1/YWHAG/ZWILCH | 32 |
| R-HSA-1474228 | Degradation of the extracellular matrix | 19/597 | 140/10867 | 2.3E-04 | 1.6E-02 | 1.4E-02 | CAPN5/COL10A1/COL14A1/COL17A1/COL18A1/COL1A1/COL1A2/COL3A1/COL5A1/COL5A2/COL6A3/DCN/FBN1/FBN2/LAMB3/LAMC1/MMP14/PHYKPL/TIMP2 | 19 |
| R-HSA-6806834 | Signaling by MET | 13/597 | 79/10867 | 3.4E-04 | 2.2E-02 | 2.0E-02 | COL1A1/COL1A2/COL3A1/COL5A1/COL5A2/ITGB1/LAMB3/LAMC1/LRIG1/MET/NRAS/PIK3R1/RAPGEF1 | 13 |
| R-HSA-9696264 | RND3 GTPase cycle | 9/597 | 42/10867 | 3.7E-04 | 2.3E-02 | 2.1E-02 | CAV1/CKAP4/DST/EPHA2/NISCH/PIK3R1/RND3/TNFAIP1/WDR6 | 9 |
| R-HSA-9696270 | RND2 GTPase cycle | 9/597 | 43/10867 | 4.5E-04 | 2.7E-02 | 2.4E-02 | CAV1/CKAP4/DST/EPHA2/NISCH/PIK3R1/TNFAIP1/UHRF1BP1L/WDR6 | 9 |
| R-HSA-187037 | Signaling by NTRK1 (TRKA) | 16/597 | 115/10867 | 5.3E-04 | 3.0E-02 | 2.7E-02 | CLTC/DUSP6/EGR1/F3/ID1/ID3/IRS2/JUNB/MEF2D/NRAS/PIK3R1/RALA/RAPGEF1/RRAD/SRF/VRK3 | 16 |
| R-HSA-216083 | Integrin cell surface interactions | 13/597 | 85/10867 | 7.0E-04 | 3.8E-02 | 3.4E-02 | COL10A1/COL18A1/COL1A1/COL1A2/COL3A1/COL5A1/COL5A2/COL6A3/FBN1/ITGA11/ITGB1/ITGB2/ITGB3 | 13 |
| R-HSA-68877 | Mitotic Prometaphase | 23/597 | 204/10867 | 8.2E-04 | 4.1E-02 | 3.6E-02 | AURKB/BUB1/CCNB2/CDCA8/CENPA/CENPK/CENPQ/CENPT/EML4/HSP90AA1/MAD2L1/MAPRE1/NCAPD2/NUP160/SGO1/SMC1A/SMC4/SPDL1/TUBA1C/TUBG1/TUBGCP6/YWHAG/ZWILCH | 23 |
| R-HSA-3000178 | ECM proteoglycans | 12/597 | 76/10867 | 8.3E-04 | 4.1E-02 | 3.6E-02 | COL1A1/COL1A2/COL3A1/COL5A1/COL5A2/COL6A3/DCN/ITGB1/ITGB3/LAMC1/SPARC/VCAN | 12 |
| R-HSA-5663202 | Diseases of signal transduction by growth factor receptors and second messengers | 40/597 | 433/10867 | 8.4E-04 | 4.1E-02 | 3.6E-02 | AMER1/CLTC/CTNNB1/DUSP6/EML4/ERBB3/ESRP1/FGF1/FGF18/FGF2/FOXO1/FOXO3/FYN/GSK3A/GSK3B/HDAC7/HEY1/HSP90AA1/IQGAP1/IRS2/ITGB3/JAG2/JUNB/LCK/MAML2/MAP3K11/MET/MIB1/MSN/NRAS/PDGFRB/PIK3R1/PPP1CB/PSMD11/PSMD7/PSMD8/PTEN/RAC2/RPS6KB2/VCL | 40 |
| R-HSA-6791312 | TP53 Regulates Transcription of Cell Cycle Genes | 9/597 | 48/10867 | 1.0E-03 | 4.8E-02 | 4.3E-02 | ARID3A/AURKA/BTG2/CCNA2/CCNE2/CNOT1/CNOT11/PCNA/TNKS1BP1 | 9 |
| R-HSA-9031628 | NGF-stimulated transcription | 8/597 | 39/10867 | 1.1E-03 | 4.8E-02 | 4.3E-02 | EGR1/F3/ID1/ID3/JUNB/MEF2D/RRAD/SRF | 8 |
| R-HSA-76002 | Platelet activation. signaling and aggregation | 27/597 | 263/10867 | 1.3E-03 | 5.4E-02 | 4.8E-02 | ADRA2A/CALU/CFD/COL1A1/COL1A2/FYN/GNB4/GNG12/IGF1/IGF2/ITGB3/LCK/MMRN1/PCDH7/PIK3R1/PLA2G4A/PRKCA/PROS1/RAB27B/RAC2/RHOB/SELENOP/SPARC/SRGN/STXBP2/TOR4A/VCL | 27 |
| R-HSA-9013420 | RHOU GTPase cycle | 8/597 | 40/10867 | 1.3E-03 | 5.4E-02 | 4.8E-02 | CLTC/DST/EPHA2/IQGAP1/PIK3R1/RHOU/SRGAP2/WDR6 | 8 |
| R-HSA-2500257 | Resolution of Sister Chromatid Cohesion | 16/597 | 126/10867 | 1.4E-03 | 5.9E-02 | 5.3E-02 | AURKB/BUB1/CCNB2/CDCA8/CENPA/CENPK/CENPQ/CENPT/MAD2L1/MAPRE1/NUP160/SGO1/SMC1A/SPDL1/TUBA1C/ZWILCH | 16 |
| R-HSA-6804756 | Regulation of TP53 Activity through Phosphorylation | 13/597 | 92/10867 | 1.5E-03 | 5.9E-02 | 5.3E-02 | AURKA/AURKB/BLM/CCNA2/DNA2/DYRK2/EXO1/PIN1/PRKAA1/PRKAG1/RFC5/TAF11/TPX2 | 13 |
| R-HSA-198725 | Nuclear Events (kinase and transcription factor activation) | 10/597 | 61/10867 | 1.7E-03 | 6.1E-02 | 5.4E-02 | DUSP6/EGR1/F3/ID1/ID3/JUNB/MEF2D/RRAD/SRF/VRK3 | 10 |
| R-HSA-8943724 | Regulation of PTEN gene transcription | 10/597 | 61/10867 | 1.7E-03 | 6.1E-02 | 5.4E-02 | CBX2/EGR1/EZH2/HDAC7/LAMTOR4/MBD3/PPARG/RING1/RRAGC/SALL4 | 10 |
| R-HSA-9012999 | RHO GTPase cycle | 40/597 | 449/10867 | 1.7E-03 | 6.1E-02 | 5.4E-02 | AKAP12/ARHGAP20/ARHGEF12/ARHGEF3/CAV1/CDC42EP1/CKAP4/CLTC/CUL3/DAAM1/DDX39B/DIAPH2/DST/EPHA2/FERMT2/HSP90AA1/IQGAP1/ITGB1/LCK/LMNB1/MAP3K11/MSI2/NISCH/OSBPL11/PCDH7/PIK3R1/PLEKHG1/RAC2/RAPGEF1/RHOB/RHOU/RND3/SRGAP2/STK38/TNFAIP1/TOR1AIP1/UHRF1BP1L/WASF1/WDR6/WDR81 | 40 |
| R-HSA-9648025 | EML4 and NUDC in mitotic spindle formation | 15/597 | 117/10867 | 1.8E-03 | 6.4E-02 | 5.8E-02 | AURKB/BUB1/CDCA8/CENPA/CENPK/CENPQ/CENPT/EML4/MAD2L1/MAPRE1/NUP160/SGO1/SPDL1/TUBA1C/ZWILCH | 15 |
| R-HSA-195258 | RHO GTPase Effectors | 31/597 | 327/10867 | 2.1E-03 | 7.2E-02 | 6.5E-02 | AURKB/BUB1/CDCA8/CENPA/CENPK/CENPQ/CENPT/CTNNB1/DAAM1/DIAPH2/H2AX/H2BC13/H2BC5/IQGAP1/ITGB1/MAD2L1/MAPRE1/NUP160/PIN1/PPP1CB/PRKCA/RAC2/RHOB/SGO1/SPDL1/SRF/SRGAP2/TUBA1C/WASF1/YWHAG/ZWILCH | 31 |
| R-HSA-141424 | Amplification of signal from the kinetochores | 13/597 | 96/10867 | 2.2E-03 | 7.2E-02 | 6.5E-02 | AURKB/BUB1/CDCA8/CENPA/CENPK/CENPQ/CENPT/MAD2L1/MAPRE1/NUP160/SGO1/SPDL1/ZWILCH | 13 |
| R-HSA-141444 | Amplification of signal from unattached kinetochores via a MAD2 inhibitory signal | 13/597 | 96/10867 | 2.2E-03 | 7.2E-02 | 6.5E-02 | AURKB/BUB1/CDCA8/CENPA/CENPK/CENPQ/CENPT/MAD2L1/MAPRE1/NUP160/SGO1/SPDL1/ZWILCH | 13 |
| R-HSA-2243919 | Crosslinking of collagen fibrils | 5/597 | 18/10867 | 2.3E-03 | 7.4E-02 | 6.6E-02 | COL1A1/COL1A2/LOX/LOXL2/PXDN | 5 |
| R-HSA-114604 | GPVI-mediated activation cascade | 7/597 | 35/10867 | 2.5E-03 | 8.0E-02 | 7.1E-02 | COL1A1/COL1A2/FYN/LCK/PIK3R1/RAC2/RHOB | 7 |
| R-HSA-76005 | Response to elevated platelet cytosolic Ca2+ | 16/597 | 134/10867 | 2.7E-03 | 8.2E-02 | 7.3E-02 | CALU/CFD/IGF1/IGF2/ITGB3/MMRN1/PCDH7/PRKCA/PROS1/RAB27B/SELENOP/SPARC/SRGN/STXBP2/TOR4A/VCL | 16 |
| R-HSA-68886 | M Phase | 37/597 | 418/10867 | 2.7E-03 | 8.2E-02 | 7.3E-02 | ANAPC2/AURKB/BUB1/CCNB2/CDCA8/CENPA/CENPK/CENPQ/CENPT/CHMP3/EML4/ESPL1/H2AX/H2BC13/H2BC5/HSP90AA1/KIF20A/LMNB1/MAD2L1/MAPRE1/MASTL/NCAPD2/NUP160/PRKCA/PSMD11/PSMD7/PSMD8/RAE1/SGO1/SMC1A/SMC4/SPDL1/TUBA1C/TUBG1/TUBGCP6/YWHAG/ZWILCH | 37 |
| R-HSA-3000480 | Scavenging by Class A Receptors | 5/597 | 19/10867 | 3.0E-03 | 8.8E-02 | 7.9E-02 | CALR/COL1A1/COL1A2/COL3A1/HSP90B1 | 5 |
| R-HSA-71384 | Ethanol oxidation | 4/597 | 12/10867 | 3.1E-03 | 8.9E-02 | 8.0E-02 | ACSS1/ADH1A/ADH1C/ALDH1A1 | 4 |
| R-HSA-2219530 | Constitutive Signaling by Aberrant PI3K in Cancer | 11/597 | 78/10867 | 3.4E-03 | 9.4E-02 | 8.4E-02 | ERBB3/FGF1/FGF18/FGF2/FYN/IRS2/LCK/MET/PDGFRB/PIK3R1/RAC2 | 11 |
| R-HSA-199418 | Negative regulation of the PI3K/AKT network | 14/597 | 113/10867 | 3.5E-03 | 9.4E-02 | 8.4E-02 | ERBB3/FGF1/FGF18/FGF2/FYN/IL1RAP/IRS2/LCK/MET/PDGFRB/PIK3R1/PIP4K2C/PTEN/RAC2 | 14 |
| R-HSA-69618 | Mitotic Spindle Checkpoint | 14/597 | 113/10867 | 3.5E-03 | 9.4E-02 | 8.4E-02 | ANAPC2/AURKB/BUB1/CDCA8/CENPA/CENPK/CENPQ/CENPT/MAD2L1/MAPRE1/NUP160/SGO1/SPDL1/ZWILCH | 14 |
| R-HSA-9020591 | Interleukin-12 signaling | 8/597 | 47/10867 | 3.7E-03 | 9.8E-02 | 8.7E-02 | HNRNPA2B1/LMNB1/MSN/RALA/STAT4/TCP1/TYK2/VAMP7 | 8 |
| R-HSA-186797 | Signaling by PDGF | 9/597 | 58/10867 | 4.1E-03 | 1.0E-01 | 9.2E-02 | COL3A1/COL5A1/COL5A2/COL6A3/NRAS/PDGFRB/PIK3R1/RAPGEF1/STAT6 | 9 |
| R-HSA-2467813 | Separation of Sister Chromatids | 20/597 | 191/10867 | 4.1E-03 | 1.0E-01 | 9.2E-02 | ANAPC2/AURKB/BUB1/CDCA8/CENPA/CENPK/CENPQ/CENPT/ESPL1/MAD2L1/MAPRE1/NUP160/PSMD11/PSMD7/PSMD8/SGO1/SMC1A/SPDL1/TUBA1C/ZWILCH | 20 |
| R-HSA-9013424 | RHOV GTPase cycle | 7/597 | 38/10867 | 4.1E-03 | 1.0E-01 | 9.2E-02 | CLTC/DST/EPHA2/IQGAP1/MAP3K11/PIK3R1/WDR6 | 7 |
| R-HSA-9683701 | Translation of Structural Proteins | 6/597 | 29/10867 | 4.3E-03 | 1.0E-01 | 9.4E-02 | GALNT1/GSK3A/GSK3B/MOGS/PRKCSH/ST6GALNAC3 | 6 |
| R-HSA-114608 | Platelet degranulation | 15/597 | 129/10867 | 4.7E-03 | 1.1E-01 | 1.0E-01 | CALU/CFD/IGF1/IGF2/ITGB3/MMRN1/PCDH7/PROS1/RAB27B/SELENOP/SPARC/SRGN/STXBP2/TOR4A/VCL | 15 |
| R-HSA-9694635 | Translation of Structural Proteins | 8/597 | 49/10867 | 4.8E-03 | 1.1E-01 | 1.0E-01 | GALNT1/GSK3A/GSK3B/MOGS/PRKCSH/RPN2/SRPK2/ST6GALNAC3 | 8 |
| R-HSA-6811558 | PI5P. PP2A and IER3 Regulate PI3K/AKT Signaling | 13/597 | 106/10867 | 5.2E-03 | 1.2E-01 | 1.1E-01 | ERBB3/FGF1/FGF18/FGF2/FYN/IL1RAP/IRS2/LCK/MET/PDGFRB/PIK3R1/PIP4K2C/RAC2 | 13 |
| R-HSA-68882 | Mitotic Anaphase | 23/597 | 236/10867 | 5.4E-03 | 1.2E-01 | 1.1E-01 | ANAPC2/AURKB/BUB1/CCNB2/CDCA8/CENPA/CENPK/CENPQ/CENPT/CHMP3/ESPL1/LMNB1/MAD2L1/MAPRE1/NUP160/PSMD11/PSMD7/PSMD8/SGO1/SMC1A/SPDL1/TUBA1C/ZWILCH | 23 |
| R-HSA-2555396 | Mitotic Metaphase and Anaphase | 23/597 | 237/10867 | 5.7E-03 | 1.2E-01 | 1.1E-01 | ANAPC2/AURKB/BUB1/CCNB2/CDCA8/CENPA/CENPK/CENPQ/CENPT/CHMP3/ESPL1/LMNB1/MAD2L1/MAPRE1/NUP160/PSMD11/PSMD7/PSMD8/SGO1/SMC1A/SPDL1/TUBA1C/ZWILCH | 23 |
| R-HSA-2024101 | CS/DS degradation | 4/597 | 14/10867 | 5.8E-03 | 1.2E-01 | 1.1E-01 | ARSB/DCN/HYAL1/VCAN | 4 |
| R-HSA-5365859 | RA biosynthesis pathway | 5/597 | 22/10867 | 5.9E-03 | 1.2E-01 | 1.1E-01 | ADH1A/ADH1C/AKR1C3/ALDH1A1/DHRS3 | 5 |
| R-HSA-5655332 | Signaling by FGFR3 in disease | 5/597 | 22/10867 | 5.9E-03 | 1.2E-01 | 1.1E-01 | FGF1/FGF18/FGF2/NRAS/PIK3R1 | 5 |
| R-HSA-8853338 | Signaling by FGFR3 point mutants in cancer | 5/597 | 22/10867 | 5.9E-03 | 1.2E-01 | 1.1E-01 | FGF1/FGF18/FGF2/NRAS/PIK3R1 | 5 |
| R-HSA-2428928 | IRS-related events triggered by IGF1R | 8/597 | 52/10867 | 7.0E-03 | 1.4E-01 | 1.3E-01 | FGF1/FGF18/FGF2/IGF1/IGF2/IRS2/NRAS/PIK3R1 | 8 |
| R-HSA-9013418 | RHOBTB2 GTPase cycle | 5/597 | 23/10867 | 7.2E-03 | 1.4E-01 | 1.3E-01 | CUL3/DDX39B/HSP90AA1/MSI2/STK38 | 5 |
| R-HSA-2173782 | Binding and Uptake of Ligands by Scavenger Receptors | 7/597 | 42/10867 | 7.3E-03 | 1.4E-01 | 1.3E-01 | CALR/COL1A1/COL1A2/COL3A1/HSP90AA1/HSP90B1/SPARC | 7 |
| R-HSA-9035034 | RHOF GTPase cycle | 7/597 | 42/10867 | 7.3E-03 | 1.4E-01 | 1.3E-01 | AKAP12/CAV1/DIAPH2/LMNB1/PIK3R1/SRGAP2/TOR1AIP1 | 7 |
| R-HSA-75892 | Platelet Adhesion to exposed collagen | 4/597 | 15/10867 | 7.6E-03 | 1.5E-01 | 1.3E-01 | COL1A1/COL1A2/FYN/ITGB1 | 4 |
| R-HSA-2428924 | IGF1R signaling cascade | 8/597 | 53/10867 | 7.9E-03 | 1.5E-01 | 1.3E-01 | FGF1/FGF18/FGF2/IGF1/IGF2/IRS2/NRAS/PIK3R1 | 8 |
| R-HSA-9679506 | SARS-CoV Infections | 16/597 | 151/10867 | 8.7E-03 | 1.6E-01 | 1.5E-01 | ATP1A2/CHMP3/FKBP4/GALNT1/GSK3A/GSK3B/HSP90AA1/ITGB1/MBD3/MOGS/NR3C1/PRKCSH/RPN2/SRPK2/ST6GALNAC3/TYK2 | 16 |
| R-HSA-2404192 | Signaling by Type 1 Insulin-like Growth Factor 1 Receptor (IGF1R) | 8/597 | 54/10867 | 8.8E-03 | 1.6E-01 | 1.5E-01 | FGF1/FGF18/FGF2/IGF1/IGF2/IRS2/NRAS/PIK3R1 | 8 |
| R-HSA-6807070 | PTEN Regulation | 15/597 | 140/10867 | 9.9E-03 | 1.8E-01 | 1.6E-01 | CBX2/EGR1/EZH2/HDAC7/LAMTOR4/MBD3/NEDD4/PPARG/PSMD11/PSMD7/PSMD8/PTEN/RING1/RRAGC/SALL4 | 15 |
| R-HSA-5654708 | Downstream signaling of activated FGFR3 | 5/597 | 25/10867 | 1.0E-02 | 1.9E-01 | 1.7E-01 | FGF1/FGF18/FGF2/NRAS/PIK3R1 | 5 |
| R-HSA-5674400 | Constitutive Signaling by AKT1 E17K in Cancer | 5/597 | 25/10867 | 1.0E-02 | 1.9E-01 | 1.7E-01 | FOXO1/FOXO3/GSK3A/GSK3B/RPS6KB2 | 5 |
| R-HSA-1566948 | Elastic fibre formation | 7/597 | 45/10867 | 1.1E-02 | 1.9E-01 | 1.7E-01 | BMP2/FBN1/FBN2/ITGB1/ITGB3/LOX/LOXL2 | 7 |
| R-HSA-446728 | Cell junction organization | 11/597 | 91/10867 | 1.1E-02 | 1.9E-01 | 1.7E-01 | CDH17/CLDN15/COL17A1/CTNNB1/DST/FERMT2/ITGB1/LAMB3/LIMS1/PRKCI/TESK1 | 11 |
| R-HSA-1500931 | Cell-Cell communication | 14/597 | 129/10867 | 1.1E-02 | 1.9E-01 | 1.7E-01 | CDH17/CLDN15/COL17A1/CTNNB1/DST/FERMT2/FYN/IQGAP1/ITGB1/LAMB3/LIMS1/PIK3R1/PRKCI/TESK1 | 14 |
| R-HSA-202131 | Metabolism of nitric oxide: NOS3 activation and regulation | 4/597 | 17/10867 | 1.2E-02 | 2.0E-01 | 1.8E-01 | CAV1/HSP90AA1/NOS3/NOSTRIN | 4 |
| R-HSA-447115 | Interleukin-12 family signaling | 8/597 | 57/10867 | 1.2E-02 | 2.0E-01 | 1.8E-01 | HNRNPA2B1/LMNB1/MSN/RALA/STAT4/TCP1/TYK2/VAMP7 | 8 |
| R-HSA-69239 | Synthesis of DNA | 13/597 | 120/10867 | 1.4E-02 | 2.2E-01 | 2.0E-01 | ANAPC2/CCNA2/CCNE2/CDT1/DNA2/MCM3/MCM4/PCNA/PSMD11/PSMD7/PSMD8/RFC5/SKP2 | 13 |
| R-HSA-1538133 | G0 and Early G1 | 5/597 | 27/10867 | 1.5E-02 | 2.2E-01 | 2.0E-01 | CCNA2/CCNE2/CDC25A/MYBL2/PCNA | 5 |
| R-HSA-5654716 | Downstream signaling of activated FGFR4 | 5/597 | 27/10867 | 1.5E-02 | 2.2E-01 | 2.0E-01 | FGF1/FGF18/FGF2/NRAS/PIK3R1 | 5 |
| R-HSA-190377 | FGFR2b ligand binding and activation | 3/597 | 10/10867 | 1.5E-02 | 2.2E-01 | 2.0E-01 | FGF1/FGF2/FGFBP1 | 3 |
| R-HSA-9617629 | Regulation of FOXO transcriptional activity by acetylation | 3/597 | 10/10867 | 1.5E-02 | 2.2E-01 | 2.0E-01 | FOXO1/FOXO3/TXNIP | 3 |
| R-HSA-446353 | Cell-extracellular matrix interactions | 4/597 | 18/10867 | 1.5E-02 | 2.2E-01 | 2.0E-01 | FERMT2/ITGB1/LIMS1/TESK1 | 4 |
| R-HSA-5654704 | SHC-mediated cascade:FGFR3 | 4/597 | 18/10867 | 1.5E-02 | 2.2E-01 | 2.0E-01 | FGF1/FGF18/FGF2/NRAS | 4 |
| R-HSA-5654710 | PI-3K cascade:FGFR3 | 4/597 | 18/10867 | 1.5E-02 | 2.2E-01 | 2.0E-01 | FGF1/FGF18/FGF2/PIK3R1 | 4 |
| R-HSA-8878166 | Transcriptional regulation by RUNX2 | 13/597 | 121/10867 | 1.5E-02 | 2.3E-01 | 2.1E-01 | BMP2/COL1A1/ESRRA/GSK3B/HAND2/HEY1/NR3C1/PSMD11/PSMD7/PSMD8/RUNX1/SKP2/WWTR1 | 13 |
| R-HSA-69242 | S Phase | 16/597 | 162/10867 | 1.6E-02 | 2.4E-01 | 2.1E-01 | ANAPC2/CCNA2/CCNE2/CDC25A/CDT1/DNA2/GSK3B/MCM3/MCM4/PCNA/PSMD11/PSMD7/PSMD8/RFC5/SKP2/SMC1A | 16 |
| R-HSA-8950505 | Gene and protein expression by JAK-STAT signaling after Interleukin-12 stimulation | 6/597 | 38/10867 | 1.6E-02 | 2.4E-01 | 2.1E-01 | HNRNPA2B1/LMNB1/MSN/RALA/STAT4/TCP1 | 6 |
| R-HSA-9607240 | FLT3 Signaling | 6/597 | 38/10867 | 1.6E-02 | 2.4E-01 | 2.1E-01 | FOXO3/FYN/HCK/LCK/NRAS/PIK3R1 | 6 |
| R-HSA-9694516 | SARS-CoV-2 Infection | 9/597 | 72/10867 | 1.7E-02 | 2.4E-01 | 2.1E-01 | CHMP3/GALNT1/GSK3A/GSK3B/MOGS/PRKCSH/RPN2/SRPK2/ST6GALNAC3 | 9 |
| R-HSA-453279 | Mitotic G1 phase and G1/S transition | 15/597 | 149/10867 | 1.7E-02 | 2.4E-01 | 2.1E-01 | CCNA2/CCNE2/CDC25A/CDKN1C/CDT1/MCM3/MCM4/MYBL2/PCNA/PSMD11/PSMD7/PSMD8/RRM2/SKP2/TK1 | 15 |
| R-HSA-202733 | Cell surface interactions at the vascular wall | 14/597 | 137/10867 | 1.8E-02 | 2.5E-01 | 2.2E-01 | CAV1/COL1A1/COL1A2/FYN/ITGB1/ITGB2/ITGB3/LCK/NRAS/PIK3R1/PROCR/PROS1/SDC1/TNFRSF10D | 14 |
| R-HSA-1793185 | Chondroitin sulfate/dermatan sulfate metabolism | 7/597 | 50/10867 | 1.9E-02 | 2.5E-01 | 2.2E-01 | ARSB/CHST7/CHSY1/DCN/HYAL1/SDC1/VCAN | 7 |
| R-HSA-186763 | Downstream signal transduction | 5/597 | 29/10867 | 2.0E-02 | 2.5E-01 | 2.2E-01 | NRAS/PDGFRB/PIK3R1/RAPGEF1/STAT6 | 5 |
| R-HSA-380972 | Energy dependent regulation of mTOR by LKB1-AMPK | 5/597 | 29/10867 | 2.0E-02 | 2.5E-01 | 2.2E-01 | CAB39/LAMTOR4/PRKAA1/PRKAG1/RRAGC | 5 |
| R-HSA-2514853 | Condensation of Prometaphase Chromosomes | 3/597 | 11/10867 | 2.0E-02 | 2.5E-01 | 2.2E-01 | CCNB2/NCAPD2/SMC4 | 3 |
| R-HSA-446107 | Type I hemidesmosome assembly | 3/597 | 11/10867 | 2.0E-02 | 2.5E-01 | 2.2E-01 | COL17A1/DST/LAMB3 | 3 |
| R-HSA-9614399 | Regulation of localization of FOXO transcription factors | 3/597 | 11/10867 | 2.0E-02 | 2.5E-01 | 2.2E-01 | FOXO1/FOXO3/YWHAG | 3 |
| R-HSA-381426 | Regulation of Insulin-like Growth Factor (IGF) transport and uptake by Insulin-like Growth Factor Binding Proteins (IGFBPs) | 13/597 | 125/10867 | 2.0E-02 | 2.5E-01 | 2.2E-01 | CALU/CKAP4/CST3/FAM20A/FBN1/FSTL1/HSP90B1/IGF1/IGF2/IGFBP7/LAMC1/PRKCSH/VCAN | 13 |
| R-HSA-9678108 | SARS-CoV-1 Infection | 7/597 | 51/10867 | 2.1E-02 | 2.5E-01 | 2.2E-01 | CHMP3/GALNT1/GSK3A/GSK3B/MOGS/PRKCSH/ST6GALNAC3 | 7 |
| R-HSA-5683057 | MAPK family signaling cascades | 27/597 | 325/10867 | 2.1E-02 | 2.5E-01 | 2.2E-01 | ABHD17C/CDC14B/CSF2RA/CUL3/DUSP6/ERBB3/FGF1/FGF18/FGF2/FOXO1/FOXO3/FYN/IQGAP1/IRS2/ITGB3/MAP3K11/MAPK6/MET/NRAS/PDGFRB/PIK3R1/PPP1CB/PSMD11/PSMD7/PSMD8/TYK2/VCL | 27 |
| R-HSA-168276 | NS1 Mediated Effects on Host Pathways | 6/597 | 40/10867 | 2.1E-02 | 2.5E-01 | 2.2E-01 | CPSF4/KPNA1/KPNA2/KPNA4/NUP160/RAE1 | 6 |
| R-HSA-190241 | FGFR2 ligand binding and activation | 4/597 | 20/10867 | 2.2E-02 | 2.5E-01 | 2.2E-01 | FGF1/FGF18/FGF2/FGFBP1 | 4 |
| R-HSA-2022870 | Chondroitin sulfate biosynthesis | 4/597 | 20/10867 | 2.2E-02 | 2.5E-01 | 2.2E-01 | CHST7/CHSY1/DCN/VCAN | 4 |
| R-HSA-2028269 | Signaling by Hippo | 4/597 | 20/10867 | 2.2E-02 | 2.5E-01 | 2.2E-01 | LATS1/SAV1/TJP1/WWTR1 | 4 |
| R-HSA-5654706 | FRS-mediated FGFR3 signaling | 4/597 | 20/10867 | 2.2E-02 | 2.5E-01 | 2.2E-01 | FGF1/FGF18/FGF2/NRAS | 4 |
| R-HSA-5654719 | SHC-mediated cascade:FGFR4 | 4/597 | 20/10867 | 2.2E-02 | 2.5E-01 | 2.2E-01 | FGF1/FGF18/FGF2/NRAS | 4 |
| R-HSA-5654720 | PI-3K cascade:FGFR4 | 4/597 | 20/10867 | 2.2E-02 | 2.5E-01 | 2.2E-01 | FGF1/FGF18/FGF2/PIK3R1 | 4 |
| R-HSA-6804115 | TP53 regulates transcription of additional cell cycle genes whose exact role in the p53 pathway remain uncertain | 4/597 | 20/10867 | 2.2E-02 | 2.5E-01 | 2.2E-01 | BTG2/CNOT1/CNOT11/TNKS1BP1 | 4 |
| R-HSA-9013695 | NOTCH4 Intracellular Domain Regulates Transcription | 4/597 | 20/10867 | 2.2E-02 | 2.5E-01 | 2.2E-01 | FLT4/HEY1/MAML2/NOTCH2 | 4 |
| R-HSA-9669938 | Signaling by KIT in disease | 4/597 | 20/10867 | 2.2E-02 | 2.5E-01 | 2.2E-01 | FYN/LCK/NRAS/PIK3R1 | 4 |
| R-HSA-9670439 | Signaling by phosphorylated juxtamembrane. extracellular and kinase domain KIT mutants | 4/597 | 20/10867 | 2.2E-02 | 2.5E-01 | 2.2E-01 | FYN/LCK/NRAS/PIK3R1 | 4 |
| R-HSA-69481 | G2/M Checkpoints | 16/597 | 168/10867 | 2.2E-02 | 2.5E-01 | 2.3E-01 | BLM/CCNB2/CDC25A/DNA2/EXO1/H2AX/H2BC13/H2BC5/MCM3/MCM4/PSMD11/PSMD7/PSMD8/RFC5/RNF168/YWHAG | 16 |
| R-HSA-5654696 | Downstream signaling of activated FGFR2 | 5/597 | 30/10867 | 2.2E-02 | 2.5E-01 | 2.3E-01 | FGF1/FGF18/FGF2/NRAS/PIK3R1 | 5 |
| R-HSA-3108232 | SUMO E3 ligases SUMOylate target proteins | 17/597 | 182/10867 | 2.2E-02 | 2.5E-01 | 2.3E-01 | AURKA/AURKB/BLM/CBX2/CDCA8/DNMT3B/HDAC7/NR3C1/NR3C2/NUP160/PCNA/PPARG/RAE1/RING1/RNF168/SMC1A/TDG | 17 |
| R-HSA-69306 | DNA Replication | 13/597 | 128/10867 | 2.3E-02 | 2.6E-01 | 2.3E-01 | ANAPC2/CCNA2/CCNE2/CDT1/DNA2/MCM3/MCM4/PCNA/PSMD11/PSMD7/PSMD8/RFC5/SKP2 | 13 |
| R-HSA-9006931 | Signaling by Nuclear Receptors | 25/597 | 299/10867 | 2.4E-02 | 2.6E-01 | 2.3E-01 | ADH1A/ADH1C/AKR1C3/ALDH1A1/APOD/CAV1/DHRS3/FKBP4/FOXA1/FOXO3/GNB4/GNG12/H2AX/H2BC13/H2BC5/HSP90AA1/KPNA2/NOS3/NRAS/PDHX/PIK3R1/RUNX1/SMC1A/SRF/USF2 | 25 |
| R-HSA-3108214 | SUMOylation of DNA damage response and repair proteins | 9/597 | 77/10867 | 2.5E-02 | 2.6E-01 | 2.4E-01 | BLM/CBX2/HDAC7/NUP160/RAE1/RING1/RNF168/SMC1A/TDG | 9 |
| R-HSA-9009391 | Extra-nuclear estrogen signaling | 9/597 | 77/10867 | 2.5E-02 | 2.6E-01 | 2.4E-01 | CAV1/FOXO3/GNB4/GNG12/HSP90AA1/NOS3/NRAS/PIK3R1/SRF | 9 |
| R-HSA-1839130 | Signaling by activated point mutants of FGFR3 | 3/597 | 12/10867 | 2.5E-02 | 2.6E-01 | 2.4E-01 | FGF1/FGF18/FGF2 | 3 |
| R-HSA-2033514 | FGFR3 mutant receptor activation | 3/597 | 12/10867 | 2.5E-02 | 2.6E-01 | 2.4E-01 | FGF1/FGF18/FGF2 | 3 |
| R-HSA-210990 | PECAM1 interactions | 3/597 | 12/10867 | 2.5E-02 | 2.6E-01 | 2.4E-01 | FYN/ITGB3/LCK | 3 |
| R-HSA-430116 | GP1b-IX-V activation signalling | 3/597 | 12/10867 | 2.5E-02 | 2.6E-01 | 2.4E-01 | COL1A1/COL1A2/PIK3R1 | 3 |
| R-HSA-373755 | Semaphorin interactions | 8/597 | 65/10867 | 2.5E-02 | 2.6E-01 | 2.4E-01 | ARHGEF12/FYN/GSK3B/HSP90AA1/ITGB1/MET/RHOB/SEMA6D | 8 |
| R-HSA-4641262 | Disassembly of the destruction complex and recruitment of AXIN to the membrane | 5/597 | 31/10867 | 2.6E-02 | 2.6E-01 | 2.4E-01 | AMER1/CAV1/CTNNB1/FZD1/GSK3B | 5 |
| R-HSA-69052 | Switching of origins to a post-replicative state | 10/597 | 91/10867 | 2.7E-02 | 2.8E-01 | 2.5E-01 | ANAPC2/CCNA2/CCNE2/CDT1/MCM3/MCM4/PSMD11/PSMD7/PSMD8/SKP2 | 10 |
| R-HSA-69206 | G1/S Transition | 13/597 | 131/10867 | 2.8E-02 | 2.8E-01 | 2.5E-01 | CCNA2/CCNE2/CDC25A/CDT1/MCM3/MCM4/PCNA/PSMD11/PSMD7/PSMD8/RRM2/SKP2/TK1 | 13 |
| R-HSA-5362517 | Signaling by Retinoic Acid | 6/597 | 43/10867 | 2.9E-02 | 2.8E-01 | 2.5E-01 | ADH1A/ADH1C/AKR1C3/ALDH1A1/DHRS3/PDHX | 6 |
| R-HSA-3700989 | Transcriptional Regulation by TP53 | 29/597 | 365/10867 | 2.9E-02 | 2.8E-01 | 2.5E-01 | ARID3A/AURKA/AURKB/BLM/BTG2/CCNA2/CCNE2/CNOT1/CNOT11/DNA2/DYRK2/EXO1/FANCI/LAMTOR4/MBD3/NELFB/PCNA/PIN1/PIP4K2C/PRKAA1/PRKAG1/PTEN/RFC5/RRAGC/TAF11/TNFRSF10D/TNKS1BP1/TPX2/YWHAG | 29 |
| R-HSA-69190 | DNA strand elongation | 5/597 | 32/10867 | 2.9E-02 | 2.8E-01 | 2.5E-01 | DNA2/MCM3/MCM4/PCNA/RFC5 | 5 |
| R-HSA-4086400 | PCP/CE pathway | 10/597 | 92/10867 | 2.9E-02 | 2.8E-01 | 2.5E-01 | CLTC/DAAM1/FZD1/PRKCA/PSMD11/PSMD7/PSMD8/RAC2/WNT11/WNT5A | 10 |
| R-HSA-2990846 | SUMOylation | 17/597 | 188/10867 | 3.0E-02 | 2.8E-01 | 2.5E-01 | AURKA/AURKB/BLM/CBX2/CDCA8/DNMT3B/HDAC7/NR3C1/NR3C2/NUP160/PCNA/PPARG/RAE1/RING1/RNF168/SMC1A/TDG | 17 |
| R-HSA-3858494 | Beta-catenin independent WNT signaling | 14/597 | 146/10867 | 3.0E-02 | 2.8E-01 | 2.5E-01 | CLTC/CTNNB1/DAAM1/FZD1/GNB4/GNG12/PRKCA/PRKG1/PSMD11/PSMD7/PSMD8/RAC2/WNT11/WNT5A | 14 |
| R-HSA-5633007 | Regulation of TP53 Activity | 15/597 | 160/10867 | 3.0E-02 | 2.8E-01 | 2.5E-01 | AURKA/AURKB/BLM/CCNA2/DNA2/DYRK2/EXO1/MBD3/PIN1/PIP4K2C/PRKAA1/PRKAG1/RFC5/TAF11/TPX2 | 15 |
| R-HSA-2979096 | NOTCH2 Activation and Transmission of Signal to the Nucleus | 4/597 | 22/10867 | 3.0E-02 | 2.8E-01 | 2.5E-01 | CNTN1/JAG2/MIB1/NOTCH2 | 4 |
| R-HSA-389357 | CD28 dependent PI3K/Akt signaling | 4/597 | 22/10867 | 3.0E-02 | 2.8E-01 | 2.5E-01 | FYN/LCK/MAP3K8/PIK3R1 | 4 |
| R-HSA-5654712 | FRS-mediated FGFR4 signaling | 4/597 | 22/10867 | 3.0E-02 | 2.8E-01 | 2.5E-01 | FGF1/FGF18/FGF2/NRAS | 4 |
| R-HSA-912631 | Regulation of signaling by CBL | 4/597 | 22/10867 | 3.0E-02 | 2.8E-01 | 2.5E-01 | FYN/HCK/PIK3R1/RAPGEF1 | 4 |
| R-HSA-190239 | FGFR3 ligand binding and activation | 3/597 | 13/10867 | 3.1E-02 | 2.8E-01 | 2.5E-01 | FGF1/FGF18/FGF2 | 3 |
| R-HSA-190372 | FGFR3c ligand binding and activation | 3/597 | 13/10867 | 3.1E-02 | 2.8E-01 | 2.5E-01 | FGF1/FGF18/FGF2 | 3 |
| R-HSA-190375 | FGFR2c ligand binding and activation | 3/597 | 13/10867 | 3.1E-02 | 2.8E-01 | 2.5E-01 | FGF1/FGF18/FGF2 | 3 |
| R-HSA-203615 | eNOS activation | 3/597 | 13/10867 | 3.1E-02 | 2.8E-01 | 2.5E-01 | CAV1/HSP90AA1/NOS3 | 3 |
| R-HSA-2892247 | POU5F1 (OCT4). SOX2. NANOG activate genes related to proliferation | 3/597 | 13/10867 | 3.1E-02 | 2.8E-01 | 2.5E-01 | FGF2/SALL1/SALL4 | 3 |
| R-HSA-5654227 | Phospholipase C-mediated cascade |  |  | 3.1E-02 | 2.8E-01 | 2.5E-01 |  |  |
| R-HSA-425397 | Transport of vitamins. nucleosides. and related molecules | 6/597 | 44/10867 | 3.2E-02 | 2.8E-01 | 2.5E-01 | APOD/SLC35A2/SLC35B4/SLC35D1/SLCO2A1/SLCO3A1 | 6 |
| R-HSA-451927 | Interleukin-2 family signaling | 6/597 | 44/10867 | 3.2E-02 | 2.8E-01 | 2.5E-01 | CSF2RA/IL15RA/IL21R/LCK/PIK3R1/STAT4 | 6 |
| R-HSA-1980145 | Signaling by NOTCH2 | 5/597 | 33/10867 | 3.3E-02 | 2.9E-01 | 2.6E-01 | CNTN1/JAG2/MAML2/MIB1/NOTCH2 | 5 |
| R-HSA-194138 | Signaling by VEGF | 11/597 | 108/10867 | 3.5E-02 | 3.0E-01 | 2.7E-01 | CAV1/CTNNB1/FLT4/FYN/HSP90AA1/ITGB3/NOS3/NRAS/PIK3R1/PRKCA/WASF1 | 11 |
| R-HSA-8957275 | Post-translational protein phosphorylation | 11/597 | 108/10867 | 3.5E-02 | 3.0E-01 | 2.7E-01 | CALU/CKAP4/CST3/FAM20A/FBN1/FSTL1/HSP90B1/IGFBP7/LAMC1/PRKCSH/VCAN | 11 |
| R-HSA-5654695 | PI-3K cascade:FGFR2 | 4/597 | 23/10867 | 3.5E-02 | 3.0E-01 | 2.7E-01 | FGF1/FGF18/FGF2/PIK3R1 | 4 |
| R-HSA-5654699 | SHC-mediated cascade:FGFR2 | 4/597 | 23/10867 | 3.5E-02 | 3.0E-01 | 2.7E-01 | FGF1/FGF18/FGF2/NRAS | 4 |
| R-HSA-4615885 | SUMOylation of DNA replication proteins | 6/597 | 45/10867 | 3.5E-02 | 3.0E-01 | 2.7E-01 | AURKA/AURKB/CDCA8/NUP160/PCNA/RAE1 | 6 |
| R-HSA-174417 | Telomere C-strand (Lagging Strand) Synthesis | 5/597 | 34/10867 | 3.7E-02 | 3.1E-01 | 2.8E-01 | BLM/DNA2/DSCC1/PCNA/RFC5 | 5 |
| R-HSA-8878159 | Transcriptional regulation by RUNX3 | 10/597 | 96/10867 | 3.8E-02 | 3.1E-01 | 2.8E-01 | CCN2/CTNNB1/FOXO3/MAML2/PSMD11/PSMD7/PSMD8/RUNX1/WWTR1/ZFHX3 | 10 |
| R-HSA-190322 | FGFR4 ligand binding and activation | 3/597 | 14/10867 | 3.8E-02 | 3.1E-01 | 2.8E-01 | FGF1/FGF18/FGF2 | 3 |
| R-HSA-3371568 | Attenuation phase | 3/597 | 14/10867 | 3.8E-02 | 3.1E-01 | 2.8E-01 | FKBP4/HSBP1/HSP90AA1 | 3 |
| R-HSA-5358565 | Mismatch repair (MMR) directed by MSH2:MSH6 (MutSalpha) | 3/597 | 14/10867 | 3.8E-02 | 3.1E-01 | 2.8E-01 | EXO1/MSH6/PCNA | 3 |
| R-HSA-6804116 | TP53 Regulates Transcription of Genes Involved in G1 Cell Cycle Arrest | 3/597 | 14/10867 | 3.8E-02 | 3.1E-01 | 2.8E-01 | ARID3A/CCNA2/CCNE2 | 3 |
| R-HSA-9027284 | Erythropoietin activates RAS | 3/597 | 14/10867 | 3.8E-02 | 3.1E-01 | 2.8E-01 | IRS2/NRAS/RAPGEF1 | 3 |
| R-HSA-163560 | Triglyceride catabolism | 4/597 | 24/10867 | 4.0E-02 | 3.1E-01 | 2.8E-01 | CAV1/LIPE/PPP1CA/PPP1CB | 4 |
| R-HSA-452723 | Transcriptional regulation of pluripotent stem cells | 4/597 | 24/10867 | 4.0E-02 | 3.1E-01 | 2.8E-01 | FGF2/HIF3A/SALL1/SALL4 | 4 |
| R-HSA-68949 | Orc1 removal from chromatin | 8/597 | 71/10867 | 4.0E-02 | 3.1E-01 | 2.8E-01 | CCNA2/CDT1/MCM3/MCM4/PSMD11/PSMD7/PSMD8/SKP2 | 8 |
| R-HSA-9706574 | RHOBTB GTPase Cycle | 5/597 | 35/10867 | 4.1E-02 | 3.1E-01 | 2.8E-01 | CUL3/DDX39B/HSP90AA1/MSI2/STK38 | 5 |
| R-HSA-69275 | G2/M Transition | 17/597 | 196/10867 | 4.2E-02 | 3.1E-01 | 2.8E-01 | AURKA/CCNA2/CCNB2/CDC25A/HMMR/HSP90AA1/MAPRE1/MYBL2/PPP1CB/PSMD11/PSMD7/PSMD8/TPX2/TUBA1C/TUBG1/TUBGCP6/YWHAG | 17 |
| R-HSA-6783783 | Interleukin-10 signaling | 6/597 | 47/10867 | 4.2E-02 | 3.1E-01 | 2.8E-01 | CCL5/CXCL2/IL10RA/PTGS2/TNFRSF1B/TYK2 | 6 |
| R-HSA-5684996 | MAPK1/MAPK3 signaling | 23/597 | 286/10867 | 4.3E-02 | 3.1E-01 | 2.8E-01 | ABHD17C/CSF2RA/CUL3/DUSP6/ERBB3/FGF1/FGF18/FGF2/FYN/IQGAP1/IRS2/ITGB3/MAP3K11/MET/NRAS/PDGFRB/PIK3R1/PPP1CB/PSMD11/PSMD7/PSMD8/TYK2/VCL | 23 |
| R-HSA-195721 | Signaling by WNT | 26/597 | 332/10867 | 4.3E-02 | 3.1E-01 | 2.8E-01 | AMER1/CAV1/CLTC/CTNNB1/CUL3/DAAM1/FZD1/GNB4/GNG12/GSK3B/H2AX/H2BC13/H2BC5/PRKCA/PRKG1/PSMD11/PSMD7/PSMD8/RAC2/SFRP1/SOX4/TLE4/TLE5/WLS/WNT11/WNT5A | 26 |
| R-HSA-2559583 | Cellular Senescence | 17/597 | 197/10867 | 4.3E-02 | 3.1E-01 | 2.8E-01 | ANAPC2/CBX2/CCNA2/CCNE2/ERF/ETS1/ETS2/EZH2/H2AX/H2BC13/H2BC5/HMGA1/ID1/IGFBP7/LMNB1/MAP4K4/RING1 | 17 |
| R-HSA-4420097 | VEGFA-VEGFR2 Pathway | 10/597 | 99/10867 | 4.5E-02 | 3.1E-01 | 2.8E-01 | CAV1/CTNNB1/FYN/HSP90AA1/ITGB3/NOS3/NRAS/PIK3R1/PRKCA/WASF1 | 10 |
| R-HSA-453274 | Mitotic G2-G2/M phases | 17/597 | 198/10867 | 4.5E-02 | 3.1E-01 | 2.8E-01 | AURKA/CCNA2/CCNB2/CDC25A/HMMR/HSP90AA1/MAPRE1/MYBL2/PPP1CB/PSMD11/PSMD7/PSMD8/TPX2/TUBA1C/TUBG1/TUBGCP6/YWHAG | 17 |
| R-HSA-5654700 | FRS-mediated FGFR2 signaling | 4/597 | 25/10867 | 4.6E-02 | 3.1E-01 | 2.8E-01 | FGF1/FGF18/FGF2/NRAS | 4 |
| R-HSA-9006335 | Signaling by Erythropoietin | 4/597 | 25/10867 | 4.6E-02 | 3.1E-01 | 2.8E-01 | IRS2/NRAS/PIK3R1/RAPGEF1 | 4 |
| R-HSA-9664565 | Signaling by ERBB2 KD Mutants | 4/597 | 25/10867 | 4.6E-02 | 3.1E-01 | 2.8E-01 | ERBB3/HSP90AA1/NRAS/PIK3R1 | 4 |
| R-HSA-983170 | Antigen Presentation: Folding. assembly and peptide loading of class I MHC | 4/597 | 25/10867 | 4.6E-02 | 3.1E-01 | 2.8E-01 | CALR/HLA-A/PDIA3/SEC23A | 4 |
| R-HSA-2162123 | Synthesis of Prostaglandins (PG) and Thromboxanes (TX) | 3/597 | 15/10867 | 4.6E-02 | 3.1E-01 | 2.8E-01 | AKR1C3/PTGES2/PTGS2 | 3 |
| R-HSA-2214320 | Anchoring fibril formation | 3/597 | 15/10867 | 4.6E-02 | 3.1E-01 | 2.8E-01 | COL1A1/COL1A2/LAMB3 | 3 |
| R-HSA-4839743 | Signaling by CTNNB1 phospho-site mutants | 3/597 | 15/10867 | 4.6E-02 | 3.1E-01 | 2.8E-01 | AMER1/CTNNB1/GSK3B | 3 |
| R-HSA-5099900 | WNT5A-dependent internalization of FZD4 | 3/597 | 15/10867 | 4.6E-02 | 3.1E-01 | 2.8E-01 | CLTC/PRKCA/WNT5A | 3 |
| R-HSA-5339716 | Signaling by GSK3beta mutants | 3/597 | 15/10867 | 4.6E-02 | 3.1E-01 | 2.8E-01 | AMER1/CTNNB1/GSK3B | 3 |
| R-HSA-5358508 | Mismatch Repair | 3/597 | 15/10867 | 4.6E-02 | 3.1E-01 | 2.8E-01 | EXO1/MSH6/PCNA | 3 |
| R-HSA-5358747 | S33 mutants of beta-catenin aren't phosphorylated | 3/597 | 15/10867 | 4.6E-02 | 3.1E-01 | 2.8E-01 | AMER1/CTNNB1/GSK3B | 3 |
| R-HSA-5358749 | S37 mutants of beta-catenin aren't phosphorylated | 3/597 | 15/10867 | 4.6E-02 | 3.1E-01 | 2.8E-01 | AMER1/CTNNB1/GSK3B | 3 |
| R-HSA-5358751 | S45 mutants of beta-catenin aren't phosphorylated | 3/597 | 15/10867 | 4.6E-02 | 3.1E-01 | 2.8E-01 | AMER1/CTNNB1/GSK3B | 3 |
| R-HSA-5358752 | T41 mutants of beta-catenin aren't phosphorylated | 3/597 | 15/10867 | 4.6E-02 | 3.1E-01 | 2.8E-01 | AMER1/CTNNB1/GSK3B | 3 |
| R-HSA-5637810 | Constitutive Signaling by EGFRvIII | 3/597 | 15/10867 | 4.6E-02 | 3.1E-01 | 2.8E-01 | HSP90AA1/NRAS/PIK3R1 | 3 |
| R-HSA-5637812 | Signaling by EGFRvIII in Cancer | 3/597 | 15/10867 | 4.6E-02 | 3.1E-01 | 2.8E-01 | HSP90AA1/NRAS/PIK3R1 | 3 |
| R-HSA-5654228 | Phospholipase C-mediated cascade |  |  | 4.6E-02 | 3.1E-01 | 2.8E-01 |  |  |
| R-HSA-9694631 | Maturation of nucleoprotein | 3/597 | 15/10867 | 4.6E-02 | 3.1E-01 | 2.8E-01 | GSK3A/GSK3B/SRPK2 | 3 |
| R-HSA-5654738 | Signaling by FGFR2 | 8/597 | 73/10867 | 4.6E-02 | 3.1E-01 | 2.8E-01 | ESRP1/FGF1/FGF18/FGF2/FGFBP1/NRAS/PIK3R1/RBFOX2 | 8 |
| R-HSA-8939902 | Regulation of RUNX2 expression and activity | 8/597 | 73/10867 | 4.6E-02 | 3.1E-01 | 2.8E-01 | BMP2/ESRRA/GSK3B/NR3C1/PSMD11/PSMD7/PSMD8/SKP2 | 8 |
| R-HSA-73886 | Chromosome Maintenance | 13/597 | 141/10867 | 4.6E-02 | 3.1E-01 | 2.8E-01 | BLM/CCNA2/CENPA/CENPK/CENPQ/CENPT/DNA2/DSCC1/H2AX/H2BC13/H2BC5/PCNA/RFC5 | 13 |
| R-HSA-112399 | IRS-mediated signalling | 6/597 | 48/10867 | 4.6E-02 | 3.1E-01 | 2.8E-01 | FGF1/FGF18/FGF2/IRS2/NRAS/PIK3R1 | 6 |
| R-HSA-168255 | Influenza Infection | 14/597 | 156/10867 | 4.8E-02 | 3.2E-01 | 2.9E-01 | CALR/CLTC/CPSF4/HSP90AA1/KPNA1/KPNA2/KPNA4/NUP160/RAE1/RPL10/RPL28/RPL31/RPS14/RPS9 | 14 |
| R-HSA-5685938 | HDR through Single Strand Annealing (SSA) | 5/597 | 37/10867 | 5.0E-02 | 3.4E-01 | 3.0E-01 | BLM/DNA2/ERCC1/EXO1/RFC5 | 5 |
| R-HSA-1227990 | Signaling by ERBB2 in Cancer | 4/597 | 26/10867 | 5.2E-02 | 3.4E-01 | 3.1E-01 | ERBB3/HSP90AA1/NRAS/PIK3R1 | 4 |
| R-HSA-174143 | APC/C-mediated degradation of cell cycle proteins | 9/597 | 88/10867 | 5.2E-02 | 3.4E-01 | 3.1E-01 | ANAPC2/AURKA/AURKB/CCNA2/MAD2L1/PSMD11/PSMD7/PSMD8/SKP2 | 9 |
| R-HSA-453276 | Regulation of mitotic cell cycle | 9/597 | 88/10867 | 5.2E-02 | 3.4E-01 | 3.1E-01 | ANAPC2/AURKA/AURKB/CCNA2/MAD2L1/PSMD11/PSMD7/PSMD8/SKP2 | 9 |
| R-HSA-4086398 | Ca2+ pathway | 7/597 | 62/10867 | 5.3E-02 | 3.4E-01 | 3.1E-01 | CTNNB1/GNB4/GNG12/PRKCA/PRKG1/WNT11/WNT5A | 7 |
| R-HSA-1433559 | Regulation of KIT signaling | 3/597 | 16/10867 | 5.4E-02 | 3.5E-01 | 3.1E-01 | FYN/LCK/PRKCA | 3 |
| R-HSA-156711 | Polo-like kinase mediated events | 3/597 | 16/10867 | 5.4E-02 | 3.5E-01 | 3.1E-01 | CCNB2/CDC25A/MYBL2 | 3 |
| R-HSA-9665348 | Signaling by ERBB2 ECD mutants | 3/597 | 16/10867 | 5.4E-02 | 3.5E-01 | 3.1E-01 | HSP90AA1/NRAS/PIK3R1 | 3 |
| R-HSA-1227986 | Signaling by ERBB2 | 6/597 | 50/10867 | 5.5E-02 | 3.5E-01 | 3.2E-01 | ERBB3/FYN/HSP90AA1/NRAS/PIK3R1/PRKCA | 6 |
| R-HSA-2129379 | Molecules associated with elastic fibres | 5/597 | 38/10867 | 5.5E-02 | 3.5E-01 | 3.2E-01 | BMP2/FBN1/FBN2/ITGB1/ITGB3 | 5 |
| R-HSA-5673001 | RAF/MAP kinase cascade | 22/597 | 280/10867 | 5.8E-02 | 3.7E-01 | 3.3E-01 | ABHD17C/CSF2RA/CUL3/DUSP6/ERBB3/FGF1/FGF18/FGF2/FYN/IQGAP1/IRS2/ITGB3/MAP3K11/MET/NRAS/PDGFRB/PIK3R1/PPP1CB/PSMD11/PSMD7/PSMD8/VCL | 22 |
| R-HSA-198933 | Immunoregulatory interactions between a Lymphoid and a non-Lymphoid cell | 12/597 | 132/10867 | 5.9E-02 | 3.7E-01 | 3.3E-01 | CD8A/COL17A1/COL1A1/COL1A2/COL3A1/HLA-A/ITGB1/ITGB2/KLRG1/NPDC1/SIGLEC1/SIGLEC9 | 12 |
| R-HSA-180786 | Extension of Telomeres | 6/597 | 51/10867 | 5.9E-02 | 3.7E-01 | 3.4E-01 | BLM/CCNA2/DNA2/DSCC1/PCNA/RFC5 | 6 |
| R-HSA-196299 | Beta-catenin phosphorylation cascade | 3/597 | 17/10867 | 6.3E-02 | 3.9E-01 | 3.5E-01 | AMER1/CTNNB1/GSK3B | 3 |
| R-HSA-2033519 | Activated point mutants of FGFR2 | 3/597 | 17/10867 | 6.3E-02 | 3.9E-01 | 3.5E-01 | FGF1/FGF18/FGF2 | 3 |
| R-HSA-3781860 | Diseases associated with N-glycosylation of proteins | 3/597 | 17/10867 | 6.3E-02 | 3.9E-01 | 3.5E-01 | ALG13/ALG3/MOGS | 3 |
| R-HSA-4655427 | SUMOylation of DNA methylation proteins | 3/597 | 17/10867 | 6.3E-02 | 3.9E-01 | 3.5E-01 | CBX2/DNMT3B/RING1 | 3 |
| R-HSA-6788467 | IL-6-type cytokine receptor ligand interactions | 3/597 | 17/10867 | 6.3E-02 | 3.9E-01 | 3.5E-01 | LIFR/OSMR/TYK2 | 3 |
| R-HSA-9034015 | Signaling by NTRK3 (TRKC) | 3/597 | 17/10867 | 6.3E-02 | 3.9E-01 | 3.5E-01 | NELFB/NRAS/PIK3R1 | 3 |
| R-HSA-9617828 | FOXO-mediated transcription of cell cycle genes | 3/597 | 17/10867 | 6.3E-02 | 3.9E-01 | 3.5E-01 | CAV1/FOXO1/FOXO3 | 3 |
| R-HSA-9614085 | FOXO-mediated transcription | 7/597 | 65/10867 | 6.5E-02 | 3.9E-01 | 3.5E-01 | ABCA6/CAV1/FOXO1/FOXO3/NR3C1/TXNIP/YWHAG | 7 |
| R-HSA-164952 | The role of Nef in HIV-1 replication and disease pathogenesis | 4/597 | 28/10867 | 6.5E-02 | 3.9E-01 | 3.5E-01 | FYN/HCK/HLA-A/LCK | 4 |
| R-HSA-69205 | G1/S-Specific Transcription | 4/597 | 28/10867 | 6.5E-02 | 3.9E-01 | 3.5E-01 | CDT1/PCNA/RRM2/TK1 | 4 |
| R-HSA-8939211 | ESR-mediated signaling | 18/597 | 223/10867 | 6.5E-02 | 3.9E-01 | 3.5E-01 | CAV1/FKBP4/FOXA1/FOXO3/GNB4/GNG12/H2AX/H2BC13/H2BC5/HSP90AA1/KPNA2/NOS3/NRAS/PIK3R1/RUNX1/SMC1A/SRF/USF2 | 18 |
| R-HSA-5654741 | Signaling by FGFR3 | 5/597 | 40/10867 | 6.6E-02 | 4.0E-01 | 3.5E-01 | FGF1/FGF18/FGF2/NRAS/PIK3R1 | 5 |
| R-HSA-5218920 | VEGFR2 mediated vascular permeability | 4/597 | 29/10867 | 7.2E-02 | 4.2E-01 | 3.8E-01 | CAV1/CTNNB1/HSP90AA1/NOS3 | 4 |
| R-HSA-5683826 | Surfactant metabolism | 4/597 | 29/10867 | 7.2E-02 | 4.2E-01 | 3.8E-01 | ADRA2A/CKAP4/CSF2RA/CTSH | 4 |
| R-HSA-9615017 | FOXO-mediated transcription of oxidative stress. metabolic and neuronal genes | 4/597 | 29/10867 | 7.2E-02 | 4.2E-01 | 3.8E-01 | ABCA6/FOXO1/FOXO3/NR3C1 | 4 |
| R-HSA-9694548 | Maturation of spike protein | 4/597 | 29/10867 | 7.2E-02 | 4.2E-01 | 3.8E-01 | MOGS/PRKCSH/RPN2/ST6GALNAC3 | 4 |
| R-HSA-165159 | MTOR signalling | 5/597 | 41/10867 | 7.2E-02 | 4.2E-01 | 3.8E-01 | CAB39/LAMTOR4/PRKAA1/PRKAG1/RRAGC | 5 |
| R-HSA-5654743 | Signaling by FGFR4 | 5/597 | 41/10867 | 7.2E-02 | 4.2E-01 | 3.8E-01 | FGF1/FGF18/FGF2/NRAS/PIK3R1 | 5 |
| R-HSA-9013423 | RAC3 GTPase cycle | 9/597 | 94/10867 | 7.3E-02 | 4.2E-01 | 3.8E-01 | CAV1/CDC42EP1/EPHA2/FERMT2/ITGB1/PIK3R1/RAPGEF1/SRGAP2/WASF1 | 9 |
| R-HSA-5654221 | Phospholipase C-mediated cascade |  |  | 7.3E-02 | 4.2E-01 | 3.8E-01 |  |  |
| R-HSA-74751 | Insulin receptor signalling cascade | 6/597 | 54/10867 | 7.4E-02 | 4.2E-01 | 3.8E-01 | FGF1/FGF18/FGF2/IRS2/NRAS/PIK3R1 | 6 |
| R-HSA-9013405 | RHOD GTPase cycle | 6/597 | 54/10867 | 7.4E-02 | 4.2E-01 | 3.8E-01 | AKAP12/CAV1/DIAPH2/LMNB1/PIK3R1/TOR1AIP1 | 6 |
| R-HSA-69473 | G2/M DNA damage checkpoint | 9/597 | 95/10867 | 7.7E-02 | 4.4E-01 | 3.9E-01 | BLM/DNA2/EXO1/H2AX/H2BC13/H2BC5/RFC5/RNF168/YWHAG | 9 |
| R-HSA-5693538 | Homology Directed Repair | 12/597 | 138/10867 | 7.7E-02 | 4.4E-01 | 3.9E-01 | BLM/CCNA2/DNA2/ERCC1/EXO1/H2AX/H2BC13/H2BC5/PCNA/POLQ/RFC5/RNF168 | 12 |
| R-HSA-9696273 | RND1 GTPase cycle | 5/597 | 42/10867 | 7.9E-02 | 4.4E-01 | 4.0E-01 | CAV1/DST/EPHA2/PIK3R1/WDR6 | 5 |
| R-HSA-2424491 | DAP12 signaling | 4/597 | 30/10867 | 8.0E-02 | 4.4E-01 | 4.0E-01 | FYN/LCK/NRAS/PIK3R1 | 4 |
| R-HSA-3000157 | Laminin interactions | 4/597 | 30/10867 | 8.0E-02 | 4.4E-01 | 4.0E-01 | COL18A1/ITGB1/LAMB3/LAMC1 | 4 |
| R-HSA-9700206 | Signaling by ALK in cancer | 6/597 | 55/10867 | 8.0E-02 | 4.4E-01 | 4.0E-01 | CLTC/EML4/JUNB/MSN/PIK3R1/VCL | 6 |
| R-HSA-9725370 | Signaling by ALK fusions and activated point mutants | 6/597 | 55/10867 | 8.0E-02 | 4.4E-01 | 4.0E-01 | CLTC/EML4/JUNB/MSN/PIK3R1/VCL | 6 |
| R-HSA-9013694 | Signaling by NOTCH4 | 8/597 | 82/10867 | 8.0E-02 | 4.4E-01 | 4.0E-01 | FLT4/HEY1/MAML2/NOTCH2/PSMD11/PSMD7/PSMD8/TACC3 | 8 |
| R-HSA-1236382 | Constitutive Signaling by Ligand-Responsive EGFR Cancer Variants | 3/597 | 19/10867 | 8.3E-02 | 4.5E-01 | 4.0E-01 | HSP90AA1/NRAS/PIK3R1 | 3 |
| R-HSA-174414 | Processive synthesis on the C-strand of the telomere | 3/597 | 19/10867 | 8.3E-02 | 4.5E-01 | 4.0E-01 | BLM/DNA2/PCNA | 3 |
| R-HSA-3928664 | Ephrin signaling | 3/597 | 19/10867 | 8.3E-02 | 4.5E-01 | 4.0E-01 | EFNB2/EPHB6/FYN | 3 |
| R-HSA-5637815 | Signaling by Ligand-Responsive EGFR Variants in Cancer | 3/597 | 19/10867 | 8.3E-02 | 4.5E-01 | 4.0E-01 | HSP90AA1/NRAS/PIK3R1 | 3 |
| R-HSA-9725371 | Nuclear events stimulated by ALK signaling in cancer | 3/597 | 19/10867 | 8.3E-02 | 4.5E-01 | 4.0E-01 | CLTC/EML4/JUNB | 3 |
| R-HSA-195253 | Degradation of beta-catenin by the destruction complex | 8/597 | 83/10867 | 8.5E-02 | 4.5E-01 | 4.1E-01 | AMER1/CTNNB1/GSK3B/PSMD11/PSMD7/PSMD8/TLE4/TLE5 | 8 |
| R-HSA-1433557 | Signaling by SCF-KIT | 5/597 | 43/10867 | 8.5E-02 | 4.5E-01 | 4.1E-01 | FYN/LCK/NRAS/PIK3R1/PRKCA | 5 |
| R-HSA-5655253 | Signaling by FGFR2 in disease | 5/597 | 43/10867 | 8.5E-02 | 4.5E-01 | 4.1E-01 | FGF1/FGF18/FGF2/NRAS/PIK3R1 | 5 |
| R-HSA-5654687 | Downstream signaling of activated FGFR1 | 4/597 | 31/10867 | 8.8E-02 | 4.7E-01 | 4.2E-01 | FGF1/FGF2/NRAS/PIK3R1 | 4 |
| R-HSA-9013026 | RHOB GTPase cycle | 7/597 | 70/10867 | 8.9E-02 | 4.7E-01 | 4.2E-01 | ARHGEF12/ARHGEF3/CAV1/DAAM1/PCDH7/PIK3R1/RHOB | 7 |
| R-HSA-5693607 | Processing of DNA double-strand break ends | 9/597 | 98/10867 | 8.9E-02 | 4.7E-01 | 4.2E-01 | BLM/CCNA2/DNA2/EXO1/H2AX/H2BC13/H2BC5/RFC5/RNF168 | 9 |
| R-HSA-6802957 | Oncogenic MAPK signaling | 8/597 | 84/10867 | 9.0E-02 | 4.7E-01 | 4.2E-01 | DUSP6/ESRP1/IQGAP1/ITGB3/MAP3K11/NRAS/PPP1CB/VCL | 8 |
| R-HSA-109704 | PI3K Cascade | 5/597 | 44/10867 | 9.2E-02 | 4.8E-01 | 4.3E-01 | FGF1/FGF18/FGF2/IRS2/PIK3R1 | 5 |
| R-HSA-3560783 | Defective B4GALT7 causes EDS. progeroid type | 3/597 | 20/10867 | 9.4E-02 | 4.8E-01 | 4.3E-01 | DCN/SDC1/VCAN | 3 |
| R-HSA-3560801 | Defective B3GAT3 causes JDSSDHD | 3/597 | 20/10867 | 9.4E-02 | 4.8E-01 | 4.3E-01 | DCN/SDC1/VCAN | 3 |
| R-HSA-4420332 | Defective B3GALT6 causes EDSP2 and SEMDJL1 | 3/597 | 20/10867 | 9.4E-02 | 4.8E-01 | 4.3E-01 | DCN/SDC1/VCAN | 3 |
| R-HSA-69186 | Lagging Strand Synthesis | 3/597 | 20/10867 | 9.4E-02 | 4.8E-01 | 4.3E-01 | DNA2/PCNA/RFC5 | 3 |
| R-HSA-1236394 | Signaling by ERBB4 | 6/597 | 58/10867 | 9.7E-02 | 5.0E-01 | 4.5E-01 | ERBB3/MXD4/NEDD4/NRAS/PIK3R1/SPARC | 6 |
| R-HSA-2172127 | DAP12 interactions | 5/597 | 45/10867 | 9.9E-02 | 5.0E-01 | 4.5E-01 | FYN/LCK/NRAS/PIK3R1/SIGLEC15 | 5 |
| R-HSA-157118 | Signaling by NOTCH | 18/597 | 236/10867 | 9.9E-02 | 5.0E-01 | 4.5E-01 | CNTN1/FLT4/H2AX/H2BC13/H2BC5/HDAC7/HEY1/JAG2/MAML2/MIB1/NOTCH2/PRKCI/PSMD11/PSMD7/PSMD8/RUNX1/TACC3/TLE4 | 18 |
| R-HSA-1169408 | ISG15 antiviral mechanism | 7/597 | 72/10867 | 9.9E-02 | 5.0E-01 | 4.5E-01 | KPNA1/KPNA2/KPNA4/NEDD4/NUP160/PIN1/RAE1 | 7 |
| R-HSA-112311 | Neurotransmitter clearance | 2/597 | 10/10867 | 1.0E-01 | 5.0E-01 | 4.5E-01 | BCHE/MAOA | 2 |
| R-HSA-1483115 | Hydrolysis of LPC | 2/597 | 10/10867 | 1.0E-01 | 5.0E-01 | 4.5E-01 | GPCPD1/PLA2G4A | 2 |
| R-HSA-381183 | ATF6 (ATF6-alpha) activates chaperone genes | 2/597 | 10/10867 | 1.0E-01 | 5.0E-01 | 4.5E-01 | CALR/HSP90B1 | 2 |
| R-HSA-390450 | Folding of actin by CCT/TriC | 2/597 | 10/10867 | 1.0E-01 | 5.0E-01 | 4.5E-01 | CCT8/TCP1 | 2 |
| R-HSA-419812 | Calcitonin-like ligand receptors | 2/597 | 10/10867 | 1.0E-01 | 5.0E-01 | 4.5E-01 | CALCRL/RAMP2 | 2 |
| R-HSA-8853334 | Signaling by FGFR3 fusions in cancer | 2/597 | 10/10867 | 1.0E-01 | 5.0E-01 | 4.5E-01 | NRAS/PIK3R1 | 2 |
| R-HSA-9020958 | Interleukin-21 signaling | 2/597 | 10/10867 | 1.0E-01 | 5.0E-01 | 4.5E-01 | IL21R/STAT4 | 2 |
| R-HSA-162909 | Host Interactions of HIV factors | 11/597 | 130/10867 | 1.0E-01 | 5.0E-01 | 4.5E-01 | FYN/HCK/HLA-A/HMGA1/KPNA1/LCK/NUP160/PSMD11/PSMD7/PSMD8/RAE1 | 11 |
| R-HSA-190236 | Signaling by FGFR | 8/597 | 87/10867 | 1.0E-01 | 5.0E-01 | 4.5E-01 | ESRP1/FGF1/FGF18/FGF2/FGFBP1/NRAS/PIK3R1/RBFOX2 | 8 |
| R-HSA-180910 | Vpr-mediated nuclear import of PICs | 4/597 | 33/10867 | 1.0E-01 | 5.0E-01 | 4.5E-01 | HMGA1/KPNA1/NUP160/RAE1 | 4 |
| R-HSA-2559585 | Oncogene Induced Senescence | 4/597 | 33/10867 | 1.0E-01 | 5.0E-01 | 4.5E-01 | ERF/ETS1/ETS2/ID1 | 4 |
| R-HSA-389356 | CD28 co-stimulation | 4/597 | 33/10867 | 1.0E-01 | 5.0E-01 | 4.5E-01 | FYN/LCK/MAP3K8/PIK3R1 | 4 |
| R-HSA-5694530 | Cargo concentration in the ER | 4/597 | 33/10867 | 1.0E-01 | 5.0E-01 | 4.5E-01 | CD59/LMAN2/MCFD2/SEC23A | 4 |
| R-HSA-8854518 | AURKA Activation by TPX2 | 7/597 | 73/10867 | 1.1E-01 | 5.0E-01 | 4.5E-01 | AURKA/HMMR/HSP90AA1/MAPRE1/TPX2/TUBG1/YWHAG | 7 |
| R-HSA-5621575 | CD209 (DC-SIGN) signaling | 3/597 | 21/10867 | 1.1E-01 | 5.0E-01 | 4.5E-01 | FYN/NRAS/RELB | 3 |
| R-HSA-5654688 | SHC-mediated cascade:FGFR1 | 3/597 | 21/10867 | 1.1E-01 | 5.0E-01 | 4.5E-01 | FGF1/FGF2/NRAS | 3 |
| R-HSA-5654689 | PI-3K cascade:FGFR1 | 3/597 | 21/10867 | 1.1E-01 | 5.0E-01 | 4.5E-01 | FGF1/FGF2/PIK3R1 | 3 |
| R-HSA-9006936 | Signaling by TGFB family members | 9/597 | 102/10867 | 1.1E-01 | 5.1E-01 | 4.6E-01 | BMP2/BMPR2/FST/FSTL1/INHBA/JUNB/PPP1CA/PPP1CB/WWTR1 | 9 |
| R-HSA-5693567 | HDR through Homologous Recombination (HRR) or Single Strand Annealing (SSA) | 11/597 | 132/10867 | 1.1E-01 | 5.1E-01 | 4.6E-01 | BLM/CCNA2/DNA2/ERCC1/EXO1/H2AX/H2BC13/H2BC5/PCNA/RFC5/RNF168 | 11 |
| R-HSA-187577 | SCF(Skp2)-mediated degradation of p27/p21 | 6/597 | 60/10867 | 1.1E-01 | 5.1E-01 | 4.6E-01 | CCNA2/CCNE2/PSMD11/PSMD7/PSMD8/SKP2 | 6 |
| R-HSA-174178 | APC/C:Cdh1 mediated degradation of Cdc20 and other APC/C:Cdh1 targeted proteins in late mitosis/early G1 | 7/597 | 74/10867 | 1.1E-01 | 5.1E-01 | 4.6E-01 | ANAPC2/AURKA/AURKB/PSMD11/PSMD7/PSMD8/SKP2 | 7 |
| R-HSA-1980143 | Signaling by NOTCH1 | 7/597 | 74/10867 | 1.1E-01 | 5.1E-01 | 4.6E-01 | CNTN1/HDAC7/HEY1/JAG2/MAML2/MIB1/TLE4 | 7 |
| R-HSA-606279 | Deposition of new CENPA-containing nucleosomes at the centromere | 7/597 | 74/10867 | 1.1E-01 | 5.1E-01 | 4.6E-01 | CENPA/CENPK/CENPQ/CENPT/H2AX/H2BC13/H2BC5 | 7 |
| R-HSA-774815 | Nucleosome assembly | 7/597 | 74/10867 | 1.1E-01 | 5.1E-01 | 4.6E-01 | CENPA/CENPK/CENPQ/CENPT/H2AX/H2BC13/H2BC5 | 7 |
| R-HSA-6802946 | Signaling by moderate kinase activity BRAF mutants | 5/597 | 47/10867 | 1.1E-01 | 5.1E-01 | 4.6E-01 | IQGAP1/ITGB3/MAP3K11/NRAS/VCL | 5 |
| R-HSA-6802949 | Signaling by RAS mutants | 5/597 | 47/10867 | 1.1E-01 | 5.1E-01 | 4.6E-01 | IQGAP1/ITGB3/MAP3K11/NRAS/VCL | 5 |
| R-HSA-6802955 | Paradoxical activation of RAF signaling by kinase inactive BRAF | 5/597 | 47/10867 | 1.1E-01 | 5.1E-01 | 4.6E-01 | IQGAP1/ITGB3/MAP3K11/NRAS/VCL | 5 |
| R-HSA-9649948 | Signaling downstream of RAS mutants | 5/597 | 47/10867 | 1.1E-01 | 5.1E-01 | 4.6E-01 | IQGAP1/ITGB3/MAP3K11/NRAS/VCL | 5 |
| R-HSA-9675135 | Diseases of DNA repair | 4/597 | 34/10867 | 1.1E-01 | 5.1E-01 | 4.6E-01 | BLM/DNA2/EXO1/MSH6 | 4 |
| R-HSA-1250196 | SHC1 events in ERBB2 signaling | 3/597 | 22/10867 | 1.2E-01 | 5.1E-01 | 4.6E-01 | ERBB3/NRAS/PRKCA | 3 |
| R-HSA-392154 | Nitric oxide stimulates guanylate cyclase | 3/597 | 22/10867 | 1.2E-01 | 5.1E-01 | 4.6E-01 | GUCY1A1/NOS3/PRKG1 | 3 |
| R-HSA-8862803 | Deregulated CDK5 triggers multiple neurodegenerative pathways in Alzheimer's disease models | 3/597 | 22/10867 | 1.2E-01 | 5.1E-01 | 4.6E-01 | CDC25A/FOXO3/LMNB1 | 3 |
| R-HSA-8863678 | Neurodegenerative Diseases | 3/597 | 22/10867 | 1.2E-01 | 5.1E-01 | 4.6E-01 | CDC25A/FOXO3/LMNB1 | 3 |
| R-HSA-9665686 | Signaling by ERBB2 TMD/JMD mutants | 3/597 | 22/10867 | 1.2E-01 | 5.1E-01 | 4.6E-01 | ERBB3/HSP90AA1/NRAS | 3 |
| R-HSA-212165 | Epigenetic regulation of gene expression | 12/597 | 149/10867 | 1.2E-01 | 5.1E-01 | 4.6E-01 | DDX21/DNMT3B/EZH2/GSK3B/H2AX/H2BC13/H2BC5/MBD3/TDG/TET1/TET3/UHRF1 | 12 |
| R-HSA-1839122 | Signaling by activated point mutants of FGFR1 | 2/597 | 11/10867 | 1.2E-01 | 5.1E-01 | 4.6E-01 | FGF1/FGF2 | 2 |
| R-HSA-2022923 | Dermatan sulfate biosynthesis | 2/597 | 11/10867 | 1.2E-01 | 5.1E-01 | 4.6E-01 | DCN/VCAN | 2 |
| R-HSA-2206281 | Mucopolysaccharidoses | 2/597 | 11/10867 | 1.2E-01 | 5.1E-01 | 4.6E-01 | ARSB/HYAL1 | 2 |
| R-HSA-2586552 | Signaling by Leptin | 2/597 | 11/10867 | 1.2E-01 | 5.1E-01 | 4.6E-01 | IRS2/SH2B1 | 2 |
| R-HSA-351906 | Apoptotic cleavage of cell adhesion proteins | 2/597 | 11/10867 | 1.2E-01 | 5.1E-01 | 4.6E-01 | CTNNB1/TJP1 | 2 |
| R-HSA-5655291 | Signaling by FGFR4 in disease | 2/597 | 11/10867 | 1.2E-01 | 5.1E-01 | 4.6E-01 | NRAS/PIK3R1 | 2 |
| R-HSA-77286 | mitochondrial fatty acid beta-oxidation of saturated fatty acids | 2/597 | 11/10867 | 1.2E-01 | 5.1E-01 | 4.6E-01 | ACADL/ACADS | 2 |
| R-HSA-8851805 | MET activates RAS signaling | 2/597 | 11/10867 | 1.2E-01 | 5.1E-01 | 4.6E-01 | MET/NRAS | 2 |
| R-HSA-8875555 | MET activates RAP1 and RAC1 | 2/597 | 11/10867 | 1.2E-01 | 5.1E-01 | 4.6E-01 | MET/RAPGEF1 | 2 |
| R-HSA-9634285 | Constitutive Signaling by Overexpressed ERBB2 | 2/597 | 11/10867 | 1.2E-01 | 5.1E-01 | 4.6E-01 | HSP90AA1/NRAS | 2 |
| R-HSA-9683610 | Maturation of nucleoprotein | 2/597 | 11/10867 | 1.2E-01 | 5.1E-01 | 4.6E-01 | GSK3A/GSK3B | 2 |
| R-HSA-512988 | Interleukin-3. Interleukin-5 and GM-CSF signaling | 5/597 | 48/10867 | 1.2E-01 | 5.2E-01 | 4.7E-01 | CSF2RA/FYN/HCK/PIK3R1/RAPGEF1 | 5 |
| R-HSA-532668 | N-glycan trimming in the ER and Calnexin/Calreticulin cycle | 4/597 | 35/10867 | 1.2E-01 | 5.3E-01 | 4.7E-01 | CALR/MOGS/PDIA3/PRKCSH | 4 |
| R-HSA-8852276 | The role of GTSE1 in G2/M progression after G2 checkpoint | 7/597 | 77/10867 | 1.3E-01 | 5.4E-01 | 4.8E-01 | CCNB2/HSP90AA1/MAPRE1/PSMD11/PSMD7/PSMD8/TUBA1C | 7 |
| R-HSA-5693532 | DNA Double-Strand Break Repair | 13/597 | 167/10867 | 1.3E-01 | 5.4E-01 | 4.8E-01 | BLM/CCNA2/DNA2/ERCC1/EXO1/H2AX/H2BC13/H2BC5/KPNA2/PCNA/POLQ/RFC5/RNF168 | 13 |
| R-HSA-373753 | Nephrin family interactions | 3/597 | 23/10867 | 1.3E-01 | 5.4E-01 | 4.8E-01 | FYN/IQGAP1/PIK3R1 | 3 |
| R-HSA-5654693 | FRS-mediated FGFR1 signaling | 3/597 | 23/10867 | 1.3E-01 | 5.4E-01 | 4.8E-01 | FGF1/FGF2/NRAS | 3 |
| R-HSA-9012852 | Signaling by NOTCH3 | 5/597 | 49/10867 | 1.3E-01 | 5.4E-01 | 4.8E-01 | HEY1/JAG2/MAML2/MIB1/TACC3 | 5 |
| R-HSA-1834949 | Cytosolic sensors of pathogen-associated DNA | 6/597 | 63/10867 | 1.3E-01 | 5.4E-01 | 4.8E-01 | CTNNB1/DHX9/IFI16/POLR3B/POLR3GL/STAT6 | 6 |
| R-HSA-375165 | NCAM signaling for neurite out-growth | 6/597 | 63/10867 | 1.3E-01 | 5.4E-01 | 4.8E-01 | COL3A1/COL5A1/COL5A2/COL6A3/FYN/NRAS | 6 |
| R-HSA-176033 | Interactions of Vpr with host cellular proteins | 4/597 | 36/10867 | 1.3E-01 | 5.4E-01 | 4.8E-01 | HMGA1/KPNA1/NUP160/RAE1 | 4 |
| R-HSA-6802948 | Signaling by high-kinase activity BRAF mutants | 4/597 | 36/10867 | 1.3E-01 | 5.4E-01 | 4.8E-01 | IQGAP1/ITGB3/NRAS/VCL | 4 |
| R-HSA-76046 | RNA Polymerase III Transcription Initiation | 4/597 | 36/10867 | 1.3E-01 | 5.4E-01 | 4.8E-01 | GTF3C4/POLR3B/POLR3GL/SNAPC4 | 4 |
| R-HSA-9687139 | Aberrant regulation of mitotic cell cycle due to RB1 defects | 4/597 | 36/10867 | 1.3E-01 | 5.4E-01 | 4.8E-01 | ANAPC2/CCNE2/CDKN1C/SKP2 | 4 |
| R-HSA-5654736 | Signaling by FGFR1 | 5/597 | 50/10867 | 1.4E-01 | 5.4E-01 | 4.8E-01 | FGF1/FGF18/FGF2/NRAS/PIK3R1 | 5 |
| R-HSA-1474151 | Tetrahydrobiopterin (BH4) synthesis. recycling. salvage and regulation | 2/597 | 12/10867 | 1.4E-01 | 5.4E-01 | 4.8E-01 | HSP90AA1/NOS3 | 2 |
| R-HSA-176974 | Unwinding of DNA | 2/597 | 12/10867 | 1.4E-01 | 5.4E-01 | 4.8E-01 | MCM3/MCM4 | 2 |
| R-HSA-190373 | FGFR1c ligand binding and activation | 2/597 | 12/10867 | 1.4E-01 | 5.4E-01 | 4.8E-01 | FGF1/FGF2 | 2 |
| R-HSA-2029481 | FCGR activation | 2/597 | 12/10867 | 1.4E-01 | 5.4E-01 | 4.8E-01 | FYN/HCK | 2 |
| R-HSA-2160916 | Hyaluronan uptake and degradation | 2/597 | 12/10867 | 1.4E-01 | 5.4E-01 | 4.8E-01 | HMMR/HYAL1 | 2 |
| R-HSA-2197563 | NOTCH2 intracellular domain regulates transcription | 2/597 | 12/10867 | 1.4E-01 | 5.4E-01 | 4.8E-01 | MAML2/NOTCH2 | 2 |
| R-HSA-3371511 | HSF1 activation | 2/597 | 12/10867 | 1.4E-01 | 5.4E-01 | 4.8E-01 | HSBP1/HSP90AA1 | 2 |
| R-HSA-381033 | ATF6 (ATF6-alpha) activates chaperones | 2/597 | 12/10867 | 1.4E-01 | 5.4E-01 | 4.8E-01 | CALR/HSP90B1 | 2 |
| R-HSA-389359 | CD28 dependent Vav1 pathway | 2/597 | 12/10867 | 1.4E-01 | 5.4E-01 | 4.8E-01 | FYN/LCK | 2 |
| R-HSA-4641265 | Repression of WNT target genes | 2/597 | 12/10867 | 1.4E-01 | 5.4E-01 | 4.8E-01 | TLE4/TLE5 | 2 |
| R-HSA-5578768 | Physiological factors | 2/597 | 12/10867 | 1.4E-01 | 5.4E-01 | 4.8E-01 | NPR2/WWTR1 | 2 |
| R-HSA-879518 | Transport of organic anions | 2/597 | 12/10867 | 1.4E-01 | 5.4E-01 | 4.8E-01 | SLCO2A1/SLCO3A1 | 2 |
| R-HSA-8963693 | Aspartate and asparagine metabolism | 2/597 | 12/10867 | 1.4E-01 | 5.4E-01 | 4.8E-01 | SLC25A12/SLC25A13 | 2 |
| R-HSA-8984722 | Interleukin-35 Signalling | 2/597 | 12/10867 | 1.4E-01 | 5.4E-01 | 4.8E-01 | STAT4/TYK2 | 2 |
| R-HSA-9027276 | Erythropoietin activates Phosphoinositide-3-kinase (PI3K) | 2/597 | 12/10867 | 1.4E-01 | 5.4E-01 | 4.8E-01 | IRS2/PIK3R1 | 2 |
| R-HSA-9673767 | Signaling by PDGFRA transmembrane. juxtamembrane and kinase domain mutants | 2/597 | 12/10867 | 1.4E-01 | 5.4E-01 | 4.8E-01 | NRAS/PIK3R1 | 2 |
| R-HSA-9673770 | Signaling by PDGFRA extracellular domain mutants | 2/597 | 12/10867 | 1.4E-01 | 5.4E-01 | 4.8E-01 | NRAS/PIK3R1 | 2 |
| R-HSA-9700645 | ALK mutants bind TKIs | 2/597 | 12/10867 | 1.4E-01 | 5.4E-01 | 4.8E-01 | CLTC/EML4 | 2 |
| R-HSA-1268020 | Mitochondrial protein import | 6/597 | 64/10867 | 1.4E-01 | 5.4E-01 | 4.8E-01 | ATP5F1B/GRPEL1/GRPEL2/SLC25A12/SLC25A13/TIMM13 | 6 |
| R-HSA-3371571 | HSF1-dependent transactivation | 3/597 | 24/10867 | 1.4E-01 | 5.4E-01 | 4.8E-01 | FKBP4/HSBP1/HSP90AA1 | 3 |
| R-HSA-429947 | Deadenylation of mRNA | 3/597 | 24/10867 | 1.4E-01 | 5.4E-01 | 4.8E-01 | CNOT1/CNOT11/TNKS1BP1 | 3 |
| R-HSA-449836 | Other interleukin signaling | 3/597 | 24/10867 | 1.4E-01 | 5.4E-01 | 4.8E-01 | SDC1/STXBP2/TYK2 | 3 |
| R-HSA-5668599 | RHO GTPases Activate NADPH Oxidases | 3/597 | 24/10867 | 1.4E-01 | 5.4E-01 | 4.8E-01 | PIN1/PRKCA/RAC2 | 3 |
| R-HSA-6783589 | Interleukin-6 family signaling | 3/597 | 24/10867 | 1.4E-01 | 5.4E-01 | 4.8E-01 | LIFR/OSMR/TYK2 | 3 |
| R-HSA-8940973 | RUNX2 regulates osteoblast differentiation | 3/597 | 24/10867 | 1.4E-01 | 5.4E-01 | 4.8E-01 | COL1A1/HEY1/WWTR1 | 3 |
| R-HSA-9675136 | Diseases of DNA Double-Strand Break Repair | 3/597 | 24/10867 | 1.4E-01 | 5.4E-01 | 4.8E-01 | BLM/DNA2/EXO1 | 3 |
| R-HSA-9701193 | Defective HDR through Homologous Recombination (HRR) due to PALB2 loss of function | 3/597 | 24/10867 | 1.4E-01 | 5.4E-01 | 4.8E-01 | BLM/DNA2/EXO1 | 3 |
| R-HSA-9704331 | Defective HDR through Homologous Recombination Repair (HRR) due to PALB2 loss of BRCA1 binding function | 3/597 | 24/10867 | 1.4E-01 | 5.4E-01 | 4.8E-01 | BLM/DNA2/EXO1 | 3 |
| R-HSA-9704646 | Defective HDR through Homologous Recombination Repair (HRR) due to PALB2 loss of BRCA2/RAD51/RAD51C binding function | 3/597 | 24/10867 | 1.4E-01 | 5.4E-01 | 4.8E-01 | BLM/DNA2/EXO1 | 3 |
| R-HSA-8852135 | Protein ubiquitination | 7/597 | 79/10867 | 1.4E-01 | 5.4E-01 | 4.8E-01 | H2BC13/H2BC5/HLA-A/OTULIN/PCNA/TMEM129/UBE2T | 7 |
| R-HSA-176187 | Activation of ATR in response to replication stress | 4/597 | 37/10867 | 1.4E-01 | 5.4E-01 | 4.8E-01 | CDC25A/MCM3/MCM4/RFC5 | 4 |
| R-HSA-8979227 | Triglyceride metabolism | 4/597 | 37/10867 | 1.4E-01 | 5.4E-01 | 4.8E-01 | CAV1/LIPE/PPP1CA/PPP1CB | 4 |
| R-HSA-1630316 | Glycosaminoglycan metabolism | 10/597 | 124/10867 | 1.4E-01 | 5.4E-01 | 4.8E-01 | ARSB/B4GALT5/CHST7/CHSY1/DCN/HMMR/HS3ST3A1/HYAL1/SDC1/VCAN | 10 |
| R-HSA-9707587 | Regulation of HMOX1 expression and activity | 6/597 | 65/10867 | 1.5E-01 | 5.4E-01 | 4.9E-01 | CUL3/KEAP1/PSMD11/PSMD7/PSMD8/SKP2 | 6 |
| R-HSA-3928665 | EPH-ephrin mediated repulsion of cells | 5/597 | 51/10867 | 1.5E-01 | 5.4E-01 | 4.9E-01 | CLTC/EFNB2/EPHA2/EPHB6/FYN | 5 |
| R-HSA-5620920 | Cargo trafficking to the periciliary membrane | 5/597 | 51/10867 | 1.5E-01 | 5.4E-01 | 4.9E-01 | CCT8/INPP5E/NPHP3/PKD2/TCP1 | 5 |
| R-HSA-6798695 | Neutrophil degranulation | 32/597 | 480/10867 | 1.5E-01 | 5.4E-01 | 4.9E-01 | AGL/ARSB/ATP11B/C1orf35/C6orf120/CAB39/CAND1/CCT8/CD59/CFD/CKAP4/CST3/CTSH/DBNL/FGL2/HLA-A/HSP90AA1/HVCN1/IQGAP1/ITGB2/NRAS/PFKL/PSMD11/PSMD7/PTGES2/SIGLEC9/STK11IP/SURF4/TIMP2/TMEM30A/TNFRSF1B/VCL | 32 |
| R-HSA-1169410 | Antiviral mechanism by IFN-stimulated genes | 7/597 | 80/10867 | 1.5E-01 | 5.4E-01 | 4.9E-01 | KPNA1/KPNA2/KPNA4/NEDD4/NUP160/PIN1/RAE1 | 7 |
| R-HSA-2559586 | DNA Damage/Telomere Stress Induced Senescence | 7/597 | 80/10867 | 1.5E-01 | 5.4E-01 | 4.9E-01 | CCNA2/CCNE2/H2AX/H2BC13/H2BC5/HMGA1/LMNB1 | 7 |
| R-HSA-111465 | Apoptotic cleavage of cellular proteins | 4/597 | 38/10867 | 1.5E-01 | 5.4E-01 | 4.9E-01 | CTNNB1/DBNL/LMNB1/TJP1 | 4 |
| R-HSA-5655302 | Signaling by FGFR1 in disease | 4/597 | 38/10867 | 1.5E-01 | 5.4E-01 | 4.9E-01 | FGF1/FGF2/NRAS/PIK3R1 | 4 |
| R-HSA-6814122 | Cooperation of PDCL (PhLP1) and TRiC/CCT in G-protein beta folding | 4/597 | 38/10867 | 1.5E-01 | 5.4E-01 | 4.9E-01 | CCT8/GNB4/GNG12/TCP1 | 4 |
| R-HSA-8939243 | RUNX1 interacts with co-factors whose precise effect on RUNX1 targets is not known | 4/597 | 38/10867 | 1.5E-01 | 5.4E-01 | 4.9E-01 | CBX2/RING1/RUNX1/SMARCC1 | 4 |
| R-HSA-9675126 | Diseases of mitotic cell cycle | 4/597 | 38/10867 | 1.5E-01 | 5.4E-01 | 4.9E-01 | ANAPC2/CCNE2/CDKN1C/SKP2 | 4 |
| R-HSA-2980766 | Nuclear Envelope Breakdown | 5/597 | 52/10867 | 1.6E-01 | 5.4E-01 | 4.9E-01 | CCNB2/LMNB1/NUP160/PRKCA/RAE1 | 5 |
| R-HSA-383280 | Nuclear Receptor transcription pathway | 5/597 | 52/10867 | 1.6E-01 | 5.4E-01 | 4.9E-01 | ESRRA/NR3C1/NR3C2/NRBP1/PPARG | 5 |
| R-HSA-75153 | Apoptotic execution phase | 5/597 | 52/10867 | 1.6E-01 | 5.4E-01 | 4.9E-01 | CTNNB1/DBNL/KPNA1/LMNB1/TJP1 | 5 |
| R-HSA-1643713 | Signaling by EGFR in Cancer | 3/597 | 25/10867 | 1.6E-01 | 5.4E-01 | 4.9E-01 | HSP90AA1/NRAS/PIK3R1 | 3 |
| R-HSA-389960 | Formation of tubulin folding intermediates by CCT/TriC | 3/597 | 25/10867 | 1.6E-01 | 5.4E-01 | 4.9E-01 | CCT8/TCP1/TUBA1C | 3 |
| R-HSA-400685 | Sema4D in semaphorin signaling | 3/597 | 25/10867 | 1.6E-01 | 5.4E-01 | 4.9E-01 | ARHGEF12/MET/RHOB | 3 |
| R-HSA-418592 | ADP signalling through P2Y purinoceptor 1 | 3/597 | 25/10867 | 1.6E-01 | 5.4E-01 | 4.9E-01 | GNB4/GNG12/PLA2G4A | 3 |
| R-HSA-69273 | Cyclin A/B1/B2 associated events during G2/M transition | 3/597 | 25/10867 | 1.6E-01 | 5.4E-01 | 4.9E-01 | CCNA2/CCNB2/CDC25A | 3 |
| R-HSA-9006115 | Signaling by NTRK2 (TRKB) | 3/597 | 25/10867 | 1.6E-01 | 5.4E-01 | 4.9E-01 | FYN/NRAS/PIK3R1 | 3 |
| R-HSA-9013507 | NOTCH3 Activation and Transmission of Signal to the Nucleus | 3/597 | 25/10867 | 1.6E-01 | 5.4E-01 | 4.9E-01 | JAG2/MIB1/TACC3 | 3 |
| R-HSA-9679191 | Potential therapeutics for SARS | 7/597 | 81/10867 | 1.6E-01 | 5.4E-01 | 4.9E-01 | ATP1A2/FKBP4/HSP90AA1/ITGB1/MBD3/NR3C1/TYK2 | 7 |
| R-HSA-1502540 | Signaling by Activin | 2/597 | 13/10867 | 1.6E-01 | 5.4E-01 | 4.9E-01 | FST/INHBA | 2 |
| R-HSA-171007 | p38MAPK events | 2/597 | 13/10867 | 1.6E-01 | 5.4E-01 | 4.9E-01 | NRAS/RALA | 2 |
| R-HSA-202670 | ERKs are inactivated | 2/597 | 13/10867 | 1.6E-01 | 5.4E-01 | 4.9E-01 | DUSP6/VRK3 | 2 |
| R-HSA-5140745 | WNT5A-dependent internalization of FZD2. FZD5 and ROR2 | 2/597 | 13/10867 | 1.6E-01 | 5.4E-01 | 4.9E-01 | CLTC/WNT5A | 2 |
| R-HSA-5210891 | Uptake and function of anthrax toxins | 2/597 | 13/10867 | 1.6E-01 | 5.4E-01 | 4.9E-01 | ANTXR1/ANTXR2 | 2 |
| R-HSA-5658623 | FGFRL1 modulation of FGFR1 signaling | 2/597 | 13/10867 | 1.6E-01 | 5.4E-01 | 4.9E-01 | FGF18/FGF2 | 2 |
| R-HSA-68884 | Mitotic Telophase/Cytokinesis | 2/597 | 13/10867 | 1.6E-01 | 5.4E-01 | 4.9E-01 | KIF20A/SMC1A | 2 |
| R-HSA-140877 | Formation of Fibrin Clot (Clotting Cascade) | 4/597 | 39/10867 | 1.6E-01 | 5.6E-01 | 5.0E-01 | F3/PROCR/PROS1/TFPI | 4 |
| R-HSA-390471 | Association of TriC/CCT with target proteins during biosynthesis | 4/597 | 39/10867 | 1.6E-01 | 5.6E-01 | 5.0E-01 | CCNE2/CCT8/SKIV2L/TCP1 | 4 |
| R-HSA-5693616 | Presynaptic phase of homologous DNA pairing and strand exchange | 4/597 | 39/10867 | 1.6E-01 | 5.6E-01 | 5.0E-01 | BLM/DNA2/EXO1/RFC5 | 4 |
| R-HSA-73933 | Resolution of Abasic Sites (AP sites) | 4/597 | 39/10867 | 1.6E-01 | 5.6E-01 | 5.0E-01 | PCNA/RFC5/TDG/UNG | 4 |
| R-HSA-76009 | Platelet Aggregation (Plug Formation) | 4/597 | 39/10867 | 1.6E-01 | 5.6E-01 | 5.0E-01 | ADRA2A/COL1A1/COL1A2/ITGB3 | 4 |
| R-HSA-157579 | Telomere Maintenance | 9/597 | 113/10867 | 1.7E-01 | 5.6E-01 | 5.0E-01 | BLM/CCNA2/DNA2/DSCC1/H2AX/H2BC13/H2BC5/PCNA/RFC5 | 9 |
| R-HSA-174411 | Polymerase switching on the C-strand of the telomere | 3/597 | 26/10867 | 1.7E-01 | 5.6E-01 | 5.0E-01 | DSCC1/PCNA/RFC5 | 3 |
| R-HSA-1971475 | A tetrasaccharide linker sequence is required for GAG synthesis | 3/597 | 26/10867 | 1.7E-01 | 5.6E-01 | 5.0E-01 | DCN/SDC1/VCAN | 3 |
| R-HSA-3238698 | WNT ligand biogenesis and trafficking | 3/597 | 26/10867 | 1.7E-01 | 5.6E-01 | 5.0E-01 | WLS/WNT11/WNT5A | 3 |
| R-HSA-5223345 | Miscellaneous transport and binding events | 3/597 | 26/10867 | 1.7E-01 | 5.6E-01 | 5.0E-01 | DMTN/LRRC8A/NIPA1 | 3 |
| R-HSA-5693554 | Resolution of D-loop Structures through Synthesis-Dependent Strand Annealing (SDSA) | 3/597 | 26/10867 | 1.7E-01 | 5.6E-01 | 5.0E-01 | BLM/DNA2/EXO1 | 3 |
| R-HSA-901042 | Calnexin/calreticulin cycle | 3/597 | 26/10867 | 1.7E-01 | 5.6E-01 | 5.0E-01 | CALR/PDIA3/PRKCSH | 3 |
| R-HSA-68867 | Assembly of the pre-replicative complex | 6/597 | 68/10867 | 1.7E-01 | 5.6E-01 | 5.0E-01 | CDT1/MCM3/MCM4/PSMD11/PSMD7/PSMD8 | 6 |
| R-HSA-69615 | G1/S DNA Damage Checkpoints | 6/597 | 68/10867 | 1.7E-01 | 5.6E-01 | 5.0E-01 | CCNA2/CCNE2/CDC25A/PSMD11/PSMD7/PSMD8 | 6 |
| R-HSA-69202 | Cyclin E associated events during G1/S transition | 7/597 | 83/10867 | 1.7E-01 | 5.6E-01 | 5.1E-01 | CCNA2/CCNE2/CDC25A/PSMD11/PSMD7/PSMD8/SKP2 | 7 |
| R-HSA-1483166 | Synthesis of PA | 4/597 | 40/10867 | 1.8E-01 | 5.7E-01 | 5.1E-01 | GPAT3/MIGA2/PLA2G2A/PLA2G4A | 4 |
| R-HSA-445355 | Smooth Muscle Contraction | 4/597 | 40/10867 | 1.8E-01 | 5.7E-01 | 5.1E-01 | ANXA1/CALD1/GUCY1A1/VCL | 4 |
| R-HSA-5674135 | MAP2K and MAPK activation | 4/597 | 40/10867 | 1.8E-01 | 5.7E-01 | 5.1E-01 | IQGAP1/ITGB3/NRAS/VCL | 4 |
| R-HSA-162594 | Early Phase of HIV Life Cycle | 2/597 | 14/10867 | 1.8E-01 | 5.7E-01 | 5.1E-01 | HMGA1/KPNA1 | 2 |
| R-HSA-198323 | AKT phosphorylates targets in the cytosol | 2/597 | 14/10867 | 1.8E-01 | 5.7E-01 | 5.1E-01 | GSK3A/GSK3B | 2 |
| R-HSA-4839735 | Signaling by AXIN mutants | 2/597 | 14/10867 | 1.8E-01 | 5.7E-01 | 5.1E-01 | AMER1/GSK3B | 2 |
| R-HSA-4839744 | Signaling by APC mutants | 2/597 | 14/10867 | 1.8E-01 | 5.7E-01 | 5.1E-01 | AMER1/GSK3B | 2 |
| R-HSA-4839748 | Signaling by AMER1 mutants | 2/597 | 14/10867 | 1.8E-01 | 5.7E-01 | 5.1E-01 | AMER1/GSK3B | 2 |
| R-HSA-5358606 | Mismatch repair (MMR) directed by MSH2:MSH3 (MutSbeta) | 2/597 | 14/10867 | 1.8E-01 | 5.7E-01 | 5.1E-01 | EXO1/PCNA | 2 |
| R-HSA-5467337 | APC truncation mutants have impaired AXIN binding | 2/597 | 14/10867 | 1.8E-01 | 5.7E-01 | 5.1E-01 | AMER1/GSK3B | 2 |
| R-HSA-5467340 | AXIN missense mutants destabilize the destruction complex | 2/597 | 14/10867 | 1.8E-01 | 5.7E-01 | 5.1E-01 | AMER1/GSK3B | 2 |
| R-HSA-5467348 | Truncations of AMER1 destabilize the destruction complex | 2/597 | 14/10867 | 1.8E-01 | 5.7E-01 | 5.1E-01 | AMER1/GSK3B | 2 |
| R-HSA-69091 | Polymerase switching | 2/597 | 14/10867 | 1.8E-01 | 5.7E-01 | 5.1E-01 | PCNA/RFC5 | 2 |
| R-HSA-69109 | Leading Strand Synthesis | 2/597 | 14/10867 | 1.8E-01 | 5.7E-01 | 5.1E-01 | PCNA/RFC5 | 2 |
| R-HSA-69166 | Removal of the Flap Intermediate | 2/597 | 14/10867 | 1.8E-01 | 5.7E-01 | 5.1E-01 | DNA2/PCNA | 2 |
| R-HSA-72202 | Transport of Mature Transcript to Cytoplasm | 7/597 | 84/10867 | 1.8E-01 | 5.7E-01 | 5.1E-01 | CPSF4/DDX39B/FYTTD1/NUP160/RAE1/SRSF1/SRSF5 | 7 |
| R-HSA-76066 | RNA Polymerase III Transcription Initiation From Type 2 Promoter | 3/597 | 27/10867 | 1.8E-01 | 5.8E-01 | 5.2E-01 | GTF3C4/POLR3B/POLR3GL | 3 |
| R-HSA-3371497 | HSP90 chaperone cycle for steroid hormone receptors (SHR) in the presence of ligand | 5/597 | 55/10867 | 1.8E-01 | 5.8E-01 | 5.2E-01 | FKBP4/HSP90AA1/NR3C1/NR3C2/TUBA1C | 5 |
| R-HSA-69656 | Cyclin A:Cdk2-associated events at S phase entry | 7/597 | 85/10867 | 1.9E-01 | 5.8E-01 | 5.2E-01 | CCNA2/CCNE2/CDC25A/PSMD11/PSMD7/PSMD8/SKP2 | 7 |
| R-HSA-400253 | Circadian Clock | 6/597 | 70/10867 | 1.9E-01 | 5.8E-01 | 5.2E-01 | BHLHE41/MEF2D/NPAS2/NR3C1/PPP1CA/PPP1CB | 6 |
| R-HSA-3560782 | Diseases associated with glycosaminoglycan metabolism | 4/597 | 41/10867 | 1.9E-01 | 5.8E-01 | 5.2E-01 | CHSY1/DCN/SDC1/VCAN | 4 |
| R-HSA-74158 | RNA Polymerase III Transcription | 4/597 | 41/10867 | 1.9E-01 | 5.8E-01 | 5.2E-01 | GTF3C4/POLR3B/POLR3GL/SNAPC4 | 4 |
| R-HSA-749476 | RNA Polymerase III Abortive And Retractive Initiation | 4/597 | 41/10867 | 1.9E-01 | 5.8E-01 | 5.2E-01 | GTF3C4/POLR3B/POLR3GL/SNAPC4 | 4 |
| R-HSA-432722 | Golgi Associated Vesicle Biogenesis | 5/597 | 56/10867 | 1.9E-01 | 5.9E-01 | 5.3E-01 | CLTC/HIP1R/SH3D19/SNX2/VAMP7 | 5 |
| R-HSA-71387 | Metabolism of carbohydrates | 20/597 | 295/10867 | 1.9E-01 | 5.9E-01 | 5.3E-01 | AGL/ALDH1A1/ARSB/B4GALT5/CHST7/CHSY1/DCN/FUT11/GLYCTK/HMMR/HS3ST3A1/HYAL1/NUP160/PFKL/PHKA1/RAE1/SDC1/SLC25A12/SLC25A13/VCAN | 20 |
| R-HSA-201451 | Signaling by BMP | 3/597 | 28/10867 | 2.0E-01 | 5.9E-01 | 5.3E-01 | BMP2/BMPR2/FSTL1 | 3 |
| R-HSA-350054 | Notch-HLH transcription pathway | 3/597 | 28/10867 | 2.0E-01 | 5.9E-01 | 5.3E-01 | HDAC7/MAML2/NOTCH2 | 3 |
| R-HSA-389957 | Prefoldin mediated transfer of substrate to CCT/TriC | 3/597 | 28/10867 | 2.0E-01 | 5.9E-01 | 5.3E-01 | CCT8/TCP1/TUBA1C | 3 |
| R-HSA-400042 | Adrenaline.noradrenaline inhibits insulin secretion | 3/597 | 28/10867 | 2.0E-01 | 5.9E-01 | 5.3E-01 | ADRA2A/GNB4/GNG12 | 3 |
| R-HSA-76061 | RNA Polymerase III Transcription Initiation From Type 1 Promoter | 3/597 | 28/10867 | 2.0E-01 | 5.9E-01 | 5.3E-01 | GTF3C4/POLR3B/POLR3GL | 3 |
| R-HSA-76071 | RNA Polymerase III Transcription Initiation From Type 3 Promoter | 3/597 | 28/10867 | 2.0E-01 | 5.9E-01 | 5.3E-01 | POLR3B/POLR3GL/SNAPC4 | 3 |
| R-HSA-9619665 | EGR2 and SOX10-mediated initiation of Schwann cell myelination | 3/597 | 28/10867 | 2.0E-01 | 5.9E-01 | 5.3E-01 | LAMC1/UTRN/WWTR1 | 3 |
| R-HSA-163841 | Gamma carboxylation. hypusine formation and arylsulfatase activation | 4/597 | 42/10867 | 2.0E-01 | 5.9E-01 | 5.3E-01 | ARSB/DHPS/EIF5A2/PROS1 | 4 |
| R-HSA-419037 | NCAM1 interactions | 4/597 | 42/10867 | 2.0E-01 | 5.9E-01 | 5.3E-01 | COL3A1/COL5A1/COL5A2/COL6A3 | 4 |
| R-HSA-5693579 | Homologous DNA Pairing and Strand Exchange | 4/597 | 42/10867 | 2.0E-01 | 5.9E-01 | 5.3E-01 | BLM/DNA2/EXO1/RFC5 | 4 |
| R-HSA-2032785 | YAP1- and WWTR1 (TAZ)-stimulated gene expression | 2/597 | 15/10867 | 2.0E-01 | 5.9E-01 | 5.3E-01 | CCN2/WWTR1 | 2 |
| R-HSA-2691230 | Signaling by NOTCH1 HD Domain Mutants in Cancer | 2/597 | 15/10867 | 2.0E-01 | 5.9E-01 | 5.3E-01 | JAG2/MIB1 | 2 |
| R-HSA-2691232 | Constitutive Signaling by NOTCH1 HD Domain Mutants | 2/597 | 15/10867 | 2.0E-01 | 5.9E-01 | 5.3E-01 | JAG2/MIB1 | 2 |
| R-HSA-3772470 | Negative regulation of TCF-dependent signaling by WNT ligand antagonists | 2/597 | 15/10867 | 2.0E-01 | 5.9E-01 | 5.3E-01 | SFRP1/WNT5A | 2 |
| R-HSA-4419969 | Depolymerisation of the Nuclear Lamina | 2/597 | 15/10867 | 2.0E-01 | 5.9E-01 | 5.3E-01 | LMNB1/PRKCA | 2 |
| R-HSA-69183 | Processive synthesis on the lagging strand | 2/597 | 15/10867 | 2.0E-01 | 5.9E-01 | 5.3E-01 | DNA2/PCNA | 2 |
| R-HSA-9648895 | Response of EIF2AK1 (HRI) to heme deficiency | 2/597 | 15/10867 | 2.0E-01 | 5.9E-01 | 5.3E-01 | EIF2AK1/EIF2S1 | 2 |
| R-HSA-8932339 | ROS sensing by NFE2L2 | 5/597 | 57/10867 | 2.0E-01 | 6.1E-01 | 5.4E-01 | CUL3/KEAP1/PSMD11/PSMD7/PSMD8 | 5 |
| R-HSA-9645723 | Diseases of programmed cell death | 8/597 | 103/10867 | 2.0E-01 | 6.1E-01 | 5.4E-01 | CDC25A/DNMT3B/EZH2/FOXO3/H2AX/H2BC13/H2BC5/LMNB1 | 8 |
| R-HSA-373760 | L1CAM interactions | 9/597 | 119/10867 | 2.1E-01 | 6.1E-01 | 5.4E-01 | CLTC/CNTN1/ITGB1/ITGB3/LAMC1/MSN/SCN1B/SCN2A/TUBA1C | 9 |
| R-HSA-2565942 | Regulation of PLK1 Activity at G2/M Transition | 7/597 | 88/10867 | 2.1E-01 | 6.1E-01 | 5.4E-01 | AURKA/CCNB2/HSP90AA1/MAPRE1/PPP1CB/TUBG1/YWHAG | 7 |
| R-HSA-9013404 | RAC2 GTPase cycle | 7/597 | 88/10867 | 2.1E-01 | 6.1E-01 | 5.4E-01 | CAV1/CDC42EP1/EPHA2/IQGAP1/ITGB1/PIK3R1/RAC2 | 7 |
| R-HSA-9656223 | Signaling by RAF1 mutants | 4/597 | 43/10867 | 2.1E-01 | 6.1E-01 | 5.4E-01 | IQGAP1/ITGB3/NRAS/VCL | 4 |
| R-HSA-174184 | Cdc20:Phospho-APC/C mediated degradation of Cyclin A | 6/597 | 73/10867 | 2.1E-01 | 6.1E-01 | 5.4E-01 | ANAPC2/CCNA2/MAD2L1/PSMD11/PSMD7/PSMD8 | 6 |
| R-HSA-69017 | CDK-mediated phosphorylation and removal of Cdc6 | 6/597 | 73/10867 | 2.1E-01 | 6.1E-01 | 5.4E-01 | ANAPC2/CCNA2/CCNE2/PSMD11/PSMD7/PSMD8 | 6 |
| R-HSA-201681 | TCF dependent signaling in response to WNT | 16/597 | 233/10867 | 2.1E-01 | 6.1E-01 | 5.4E-01 | AMER1/CAV1/CTNNB1/CUL3/FZD1/GSK3B/H2AX/H2BC13/H2BC5/PSMD11/PSMD7/PSMD8/SFRP1/SOX4/TLE4/WNT5A | 16 |
| R-HSA-162588 | Budding and maturation of HIV virion | 3/597 | 29/10867 | 2.1E-01 | 6.1E-01 | 5.4E-01 | CHMP3/VPS37B/VTA1 | 3 |
| R-HSA-5654732 | Negative regulation of FGFR3 signaling | 3/597 | 29/10867 | 2.1E-01 | 6.1E-01 | 5.4E-01 | FGF1/FGF18/FGF2 | 3 |
| R-HSA-9619483 | Activation of AMPK downstream of NMDARs | 3/597 | 29/10867 | 2.1E-01 | 6.1E-01 | 5.4E-01 | PRKAA1/PRKAG1/TUBA1C | 3 |
| R-HSA-2644602 | Signaling by NOTCH1 PEST Domain Mutants in Cancer | 5/597 | 58/10867 | 2.1E-01 | 6.1E-01 | 5.4E-01 | HDAC7/HEY1/JAG2/MAML2/MIB1 | 5 |
| R-HSA-2644603 | Signaling by NOTCH1 in Cancer | 5/597 | 58/10867 | 2.1E-01 | 6.1E-01 | 5.4E-01 | HDAC7/HEY1/JAG2/MAML2/MIB1 | 5 |
| R-HSA-2644606 | Constitutive Signaling by NOTCH1 PEST Domain Mutants | 5/597 | 58/10867 | 2.1E-01 | 6.1E-01 | 5.4E-01 | HDAC7/HEY1/JAG2/MAML2/MIB1 | 5 |
| R-HSA-2894858 | Signaling by NOTCH1 HD+PEST Domain Mutants in Cancer | 5/597 | 58/10867 | 2.1E-01 | 6.1E-01 | 5.4E-01 | HDAC7/HEY1/JAG2/MAML2/MIB1 | 5 |
| R-HSA-2894862 | Constitutive Signaling by NOTCH1 HD+PEST Domain Mutants | 5/597 | 58/10867 | 2.1E-01 | 6.1E-01 | 5.4E-01 | HDAC7/HEY1/JAG2/MAML2/MIB1 | 5 |
| R-HSA-72187 | mRNA 3'-end processing | 5/597 | 58/10867 | 2.1E-01 | 6.1E-01 | 5.4E-01 | CPSF4/DDX39B/FYTTD1/SRSF1/SRSF5 | 5 |
| R-HSA-917937 | Iron uptake and transport | 5/597 | 58/10867 | 2.1E-01 | 6.1E-01 | 5.4E-01 | CAND1/CYBRD1/GLRX3/HFE/IREB2 | 5 |
| R-HSA-162906 | HIV Infection | 16/597 | 234/10867 | 2.2E-01 | 6.1E-01 | 5.4E-01 | CHMP3/FYN/HCK/HLA-A/HMGA1/KPNA1/LCK/NELFB/NUP160/PSMD11/PSMD7/PSMD8/RAE1/TAF11/VPS37B/VTA1 | 16 |
| R-HSA-8951664 | Neddylation | 16/597 | 234/10867 | 2.2E-01 | 6.1E-01 | 5.4E-01 | CAND1/CCNF/COMMD2/CUL3/FBXL15/FBXL8/FBXO31/GAN/HIF3A/KEAP1/KLHL9/PSMD11/PSMD7/PSMD8/SKP2/SOCS5 | 16 |
| R-HSA-5687128 | MAPK6/MAPK4 signaling | 7/597 | 89/10867 | 2.2E-01 | 6.1E-01 | 5.4E-01 | CDC14B/FOXO1/FOXO3/MAPK6/PSMD11/PSMD7/PSMD8 | 7 |
| R-HSA-110312 | Translesion synthesis by REV1 | 2/597 | 16/10867 | 2.2E-01 | 6.1E-01 | 5.4E-01 | PCNA/RFC5 | 2 |
| R-HSA-1362300 | Transcription of E2F targets under negative control by p107 (RBL1) and p130 (RBL2) in complex with HDAC1 | 2/597 | 16/10867 | 2.2E-01 | 6.1E-01 | 5.4E-01 | CCNA2/MYBL2 | 2 |
| R-HSA-1834941 | STING mediated induction of host immune responses | 2/597 | 16/10867 | 2.2E-01 | 6.1E-01 | 5.4E-01 | IFI16/STAT6 | 2 |
| R-HSA-190242 | FGFR1 ligand binding and activation | 2/597 | 16/10867 | 2.2E-01 | 6.1E-01 | 5.4E-01 | FGF1/FGF2 | 2 |
| R-HSA-1963642 | PI3K events in ERBB2 signaling | 2/597 | 16/10867 | 2.2E-01 | 6.1E-01 | 5.4E-01 | ERBB3/PIK3R1 | 2 |
| R-HSA-2559584 | Formation of Senescence-Associated Heterochromatin Foci (SAHF) | 2/597 | 16/10867 | 2.2E-01 | 6.1E-01 | 5.4E-01 | HMGA1/LMNB1 | 2 |
| R-HSA-2730905 | Role of LAT2/NTAL/LAB on calcium mobilization | 2/597 | 16/10867 | 2.2E-01 | 6.1E-01 | 5.4E-01 | FYN/PIK3R1 | 2 |
| R-HSA-399954 | Sema3A PAK dependent Axon repulsion | 2/597 | 16/10867 | 2.2E-01 | 6.1E-01 | 5.4E-01 | FYN/HSP90AA1 | 2 |
| R-HSA-399956 | CRMPs in Sema3A signaling | 2/597 | 16/10867 | 2.2E-01 | 6.1E-01 | 5.4E-01 | FYN/GSK3B | 2 |
| R-HSA-5654219 | Phospholipase C-mediated cascade: FGFR1 | 2/597 | 16/10867 | 2.2E-01 | 6.1E-01 | 5.4E-01 | FGF1/FGF2 | 2 |
| R-HSA-5684264 | MAP3K8 (TPL2)-dependent MAPK1/3 activation | 2/597 | 16/10867 | 2.2E-01 | 6.1E-01 | 5.4E-01 | MAP3K8/TNIP2 | 2 |
| R-HSA-9614657 | FOXO-mediated transcription of cell death genes | 2/597 | 16/10867 | 2.2E-01 | 6.1E-01 | 5.4E-01 | FOXO1/FOXO3 | 2 |
| R-HSA-9703648 | Signaling by FLT3 ITD and TKD mutants | 2/597 | 16/10867 | 2.2E-01 | 6.1E-01 | 5.4E-01 | NRAS/PIK3R1 | 2 |
| R-HSA-179419 | APC:Cdc20 mediated degradation of cell cycle proteins prior to satisfation of the cell cycle checkpoint | 6/597 | 74/10867 | 2.2E-01 | 6.1E-01 | 5.5E-01 | ANAPC2/CCNA2/MAD2L1/PSMD11/PSMD7/PSMD8 | 6 |
| R-HSA-9013408 | RHOG GTPase cycle | 6/597 | 74/10867 | 2.2E-01 | 6.1E-01 | 5.5E-01 | CAV1/CDC42EP1/EPHA2/ITGB1/MAP3K11/PIK3R1 | 6 |
| R-HSA-2142753 | Arachidonic acid metabolism | 5/597 | 59/10867 | 2.2E-01 | 6.1E-01 | 5.5E-01 | AKR1C3/EPHX2/PLA2G4A/PTGES2/PTGS2 | 5 |
| R-HSA-8866654 | E3 ubiquitin ligases ubiquitinate target proteins | 5/597 | 59/10867 | 2.2E-01 | 6.1E-01 | 5.5E-01 | H2BC13/H2BC5/HLA-A/PCNA/TMEM129 | 5 |
| R-HSA-4090294 | SUMOylation of intracellular receptors | 3/597 | 30/10867 | 2.3E-01 | 6.2E-01 | 5.6E-01 | NR3C1/NR3C2/PPARG | 3 |
| R-HSA-6804758 | Regulation of TP53 Activity through Acetylation | 3/597 | 30/10867 | 2.3E-01 | 6.2E-01 | 5.6E-01 | MBD3/PIN1/PIP4K2C | 3 |
| R-HSA-159236 | Transport of Mature mRNA derived from an Intron-Containing Transcript | 6/597 | 75/10867 | 2.3E-01 | 6.3E-01 | 5.6E-01 | DDX39B/FYTTD1/NUP160/RAE1/SRSF1/SRSF5 | 6 |
| R-HSA-390466 | Chaperonin-mediated protein folding | 7/597 | 91/10867 | 2.3E-01 | 6.4E-01 | 5.7E-01 | CCNE2/CCT8/GNB4/GNG12/SKIV2L/TCP1/TUBA1C | 7 |
| R-HSA-176409 | APC/C:Cdc20 mediated degradation of mitotic proteins | 6/597 | 76/10867 | 2.4E-01 | 6.4E-01 | 5.7E-01 | ANAPC2/CCNA2/MAD2L1/PSMD11/PSMD7/PSMD8 | 6 |
| R-HSA-1482922 | Acyl chain remodelling of PI | 2/597 | 17/10867 | 2.4E-01 | 6.4E-01 | 5.7E-01 | PLA2G2A/PLA2G4A | 2 |
| R-HSA-174437 | Removal of the Flap Intermediate from the C-strand | 2/597 | 17/10867 | 2.4E-01 | 6.4E-01 | 5.7E-01 | DNA2/PCNA | 2 |
| R-HSA-2142845 | Hyaluronan metabolism | 2/597 | 17/10867 | 2.4E-01 | 6.4E-01 | 5.7E-01 | HMMR/HYAL1 | 2 |
| R-HSA-5655862 | Translesion synthesis by POLK | 2/597 | 17/10867 | 2.4E-01 | 6.4E-01 | 5.7E-01 | PCNA/RFC5 | 2 |
| R-HSA-5656121 | Translesion synthesis by POLI | 2/597 | 17/10867 | 2.4E-01 | 6.4E-01 | 5.7E-01 | PCNA/RFC5 | 2 |
| R-HSA-70221 | Glycogen breakdown (glycogenolysis) | 2/597 | 17/10867 | 2.4E-01 | 6.4E-01 | 5.7E-01 | AGL/PHKA1 | 2 |
| R-HSA-9018677 | Biosynthesis of DHA-derived SPMs | 2/597 | 17/10867 | 2.4E-01 | 6.4E-01 | 5.7E-01 | EPHX2/PTGS2 | 2 |
| R-HSA-9659787 | Aberrant regulation of mitotic G1/S transition in cancer due to RB1 defects | 2/597 | 17/10867 | 2.4E-01 | 6.4E-01 | 5.7E-01 | CCNE2/CDKN1C | 2 |
| R-HSA-9661069 | Defective binding of RB1 mutants to E2F1.(E2F2. E2F3) | 2/597 | 17/10867 | 2.4E-01 | 6.4E-01 | 5.7E-01 | CCNE2/CDKN1C | 2 |
| R-HSA-73884 | Base Excision Repair | 7/597 | 92/10867 | 2.4E-01 | 6.4E-01 | 5.7E-01 | H2AX/H2BC13/H2BC5/PCNA/RFC5/TDG/UNG | 7 |
| R-HSA-168271 | Transport of Ribonucleoproteins into the Host Nucleus | 3/597 | 31/10867 | 2.4E-01 | 6.4E-01 | 5.7E-01 | KPNA1/NUP160/RAE1 | 3 |
| R-HSA-1839124 | FGFR1 mutant receptor activation | 3/597 | 31/10867 | 2.4E-01 | 6.4E-01 | 5.7E-01 | FGF1/FGF2/PIK3R1 | 3 |
| R-HSA-2122948 | Activated NOTCH1 Transmits Signal to the Nucleus | 3/597 | 31/10867 | 2.4E-01 | 6.4E-01 | 5.7E-01 | CNTN1/JAG2/MIB1 | 3 |
| R-HSA-5339562 | Uptake and actions of bacterial toxins | 3/597 | 31/10867 | 2.4E-01 | 6.4E-01 | 5.7E-01 | ANTXR1/ANTXR2/HSP90AA1 | 3 |
| R-HSA-5654733 | Negative regulation of FGFR4 signaling | 3/597 | 31/10867 | 2.4E-01 | 6.4E-01 | 5.7E-01 | FGF1/FGF18/FGF2 | 3 |
| R-HSA-8963899 | Plasma lipoprotein remodeling | 3/597 | 31/10867 | 2.4E-01 | 6.4E-01 | 5.7E-01 | CIDEC/LIPG/PCSK5 | 3 |
| R-HSA-110328 | Recognition and association of DNA glycosylase with site containing an affected pyrimidine | 5/597 | 61/10867 | 2.4E-01 | 6.4E-01 | 5.7E-01 | H2AX/H2BC13/H2BC5/TDG/UNG | 5 |
| R-HSA-110329 | Cleavage of the damaged pyrimidine | 5/597 | 61/10867 | 2.4E-01 | 6.4E-01 | 5.7E-01 | H2AX/H2BC13/H2BC5/TDG/UNG | 5 |
| R-HSA-73928 | Depyrimidination | 5/597 | 61/10867 | 2.4E-01 | 6.4E-01 | 5.7E-01 | H2AX/H2BC13/H2BC5/TDG/UNG | 5 |
| R-HSA-176814 | Activation of APC/C and APC/C:Cdc20 mediated degradation of mitotic proteins | 6/597 | 77/10867 | 2.5E-01 | 6.5E-01 | 5.8E-01 | ANAPC2/CCNA2/MAD2L1/PSMD11/PSMD7/PSMD8 | 6 |
| R-HSA-1912408 | Pre-NOTCH Transcription and Translation | 7/597 | 93/10867 | 2.5E-01 | 6.5E-01 | 5.8E-01 | H2AX/H2BC13/H2BC5/MAML2/NOTCH2/PRKCI/RUNX1 | 7 |
| R-HSA-68875 | Mitotic Prophase | 10/597 | 142/10867 | 2.5E-01 | 6.5E-01 | 5.9E-01 | CCNB2/H2AX/H2BC13/H2BC5/LMNB1/MASTL/NUP160/PRKCA/RAE1/SMC4 | 10 |
| R-HSA-983168 | Antigen processing: Ubiquitination & Proteasome degradation | 20/597 | 309/10867 | 2.5E-01 | 6.5E-01 | 5.9E-01 | ANAPC2/CCNF/CUL3/FBXL15/FBXL8/FBXO31/GAN/KEAP1/KLHL9/NEDD4/PJA2/PSMD11/PSMD7/PSMD8/SKP2/TRIM11/TRIM41/UBE2J1/UBE2O/UBE4A | 20 |
| R-HSA-983169 | Class I MHC mediated antigen processing & presentation | 24/597 | 377/10867 | 2.5E-01 | 6.5E-01 | 5.9E-01 | ANAPC2/CALR/CCNF/CUL3/FBXL15/FBXL8/FBXO31/GAN/HLA-A/KEAP1/KLHL9/NEDD4/PDIA3/PJA2/PSMD11/PSMD7/PSMD8/SEC23A/SKP2/TRIM11/TRIM41/UBE2J1/UBE2O/UBE4A | 24 |
| R-HSA-389958 | Cooperation of Prefoldin and TriC/CCT in actin and tubulin folding | 3/597 | 32/10867 | 2.6E-01 | 6.5E-01 | 5.9E-01 | CCT8/TCP1/TUBA1C | 3 |
| R-HSA-5626467 | RHO GTPases activate IQGAPs | 3/597 | 32/10867 | 2.6E-01 | 6.5E-01 | 5.9E-01 | CTNNB1/IQGAP1/TUBA1C | 3 |
| R-HSA-8941326 | RUNX2 regulates bone development | 3/597 | 32/10867 | 2.6E-01 | 6.5E-01 | 5.9E-01 | COL1A1/HEY1/WWTR1 | 3 |
| R-HSA-917729 | Endosomal Sorting Complex Required For Transport (ESCRT) | 3/597 | 32/10867 | 2.6E-01 | 6.5E-01 | 5.9E-01 | CHMP3/VPS37B/VTA1 | 3 |
| R-HSA-422356 | Regulation of insulin secretion | 6/597 | 78/10867 | 2.6E-01 | 6.5E-01 | 5.9E-01 | ACSL4/ADRA2A/GNB4/GNG12/IQGAP1/PRKCA | 6 |
| R-HSA-446193 | Biosynthesis of the N-glycan precursor (dolichol lipid-linked oligosaccharide. LLO) and transfer to a nascent protein | 6/597 | 78/10867 | 2.6E-01 | 6.5E-01 | 5.9E-01 | ALG13/ALG3/AMDHD2/SLC17A5/ST6GALNAC1/ST6GALNAC3 | 6 |
| R-HSA-74752 | Signaling by Insulin receptor | 6/597 | 78/10867 | 2.6E-01 | 6.5E-01 | 5.9E-01 | FGF1/FGF18/FGF2/IRS2/NRAS/PIK3R1 | 6 |
| R-HSA-4570464 | SUMOylation of RNA binding proteins | 4/597 | 47/10867 | 2.6E-01 | 6.5E-01 | 5.9E-01 | CBX2/NUP160/RAE1/RING1 | 4 |
| R-HSA-9013148 | CDC42 GTPase cycle | 11/597 | 159/10867 | 2.6E-01 | 6.5E-01 | 5.9E-01 | ARHGAP20/ARHGEF12/CAV1/CDC42EP1/DAAM1/IQGAP1/MAP3K11/PIK3R1/PLEKHG1/SRGAP2/WDR81 | 11 |
| R-HSA-162710 | Synthesis of glycosylphosphatidylinositol (GPI) | 2/597 | 18/10867 | 2.6E-01 | 6.5E-01 | 5.9E-01 | PIGP/PIGW | 2 |
| R-HSA-210993 | Tie2 Signaling | 2/597 | 18/10867 | 2.6E-01 | 6.5E-01 | 5.9E-01 | NRAS/PIK3R1 | 2 |
| R-HSA-6804114 | TP53 Regulates Transcription of Genes Involved in G2 Cell Cycle Arrest | 2/597 | 18/10867 | 2.6E-01 | 6.5E-01 | 5.9E-01 | AURKA/PCNA | 2 |
| R-HSA-73780 | RNA Polymerase III Chain Elongation | 2/597 | 18/10867 | 2.6E-01 | 6.5E-01 | 5.9E-01 | POLR3B/POLR3GL | 2 |
| R-HSA-881907 | Gastrin-CREB signalling pathway via PKC and MAPK | 2/597 | 18/10867 | 2.6E-01 | 6.5E-01 | 5.9E-01 | NRAS/PRKCA | 2 |
| R-HSA-8964315 | G beta:gamma signalling through BTK | 2/597 | 18/10867 | 2.6E-01 | 6.5E-01 | 5.9E-01 | GNB4/GNG12 | 2 |
| R-HSA-3781865 | Diseases of glycosylation | 10/597 | 143/10867 | 2.6E-01 | 6.6E-01 | 5.9E-01 | ADAMTS2/ALG13/ALG3/CHSY1/DCN/MOGS/NOTCH2/SDC1/THSD7A/VCAN | 10 |
| R-HSA-1226099 | Signaling by FGFR in disease | 5/597 | 63/10867 | 2.6E-01 | 6.6E-01 | 5.9E-01 | FGF1/FGF18/FGF2/NRAS/PIK3R1 | 5 |
| R-HSA-73929 | Base-Excision Repair. AP Site Formation | 5/597 | 63/10867 | 2.6E-01 | 6.6E-01 | 5.9E-01 | H2AX/H2BC13/H2BC5/TDG/UNG | 5 |
| R-HSA-373080 | Class B/2 (Secretin family receptors) | 7/597 | 95/10867 | 2.7E-01 | 6.7E-01 | 6.0E-01 | CALCRL/FZD1/GNB4/GNG12/RAMP2/WNT11/WNT5A | 7 |
| R-HSA-2122947 | NOTCH1 Intracellular Domain Regulates Transcription | 4/597 | 48/10867 | 2.7E-01 | 6.7E-01 | 6.0E-01 | HDAC7/HEY1/MAML2/TLE4 | 4 |
| R-HSA-418597 | G alpha (z) signalling events | 4/597 | 48/10867 | 2.7E-01 | 6.7E-01 | 6.0E-01 | ADRA2A/GNB4/GNG12/PRKCA | 4 |
| R-HSA-1592389 | Activation of Matrix Metalloproteinases | 3/597 | 33/10867 | 2.7E-01 | 6.7E-01 | 6.0E-01 | COL18A1/MMP14/TIMP2 | 3 |
| R-HSA-1839126 | FGFR2 mutant receptor activation | 3/597 | 33/10867 | 2.7E-01 | 6.7E-01 | 6.0E-01 | FGF1/FGF18/FGF2 | 3 |
| R-HSA-392518 | Signal amplification | 3/597 | 33/10867 | 2.7E-01 | 6.7E-01 | 6.0E-01 | GNB4/GNG12/PLA2G4A | 3 |
| R-HSA-4085001 | Sialic acid metabolism | 3/597 | 33/10867 | 2.7E-01 | 6.7E-01 | 6.0E-01 | SLC17A5/ST6GALNAC1/ST6GALNAC3 | 3 |
| R-HSA-5693568 | Resolution of D-loop Structures through Holliday Junction Intermediates | 3/597 | 33/10867 | 2.7E-01 | 6.7E-01 | 6.0E-01 | BLM/DNA2/EXO1 | 3 |
| R-HSA-68962 | Activation of the pre-replicative complex | 3/597 | 33/10867 | 2.7E-01 | 6.7E-01 | 6.0E-01 | CDT1/MCM3/MCM4 | 3 |
| R-HSA-5668914 | Diseases of metabolism | 16/597 | 245/10867 | 2.7E-01 | 6.7E-01 | 6.0E-01 | ABCD4/ADAMTS2/ALG13/ALG3/ARSB/CHSY1/CSF2RA/DCN/HYAL1/MAOA/MOGS/NOTCH2/SDC1/SLC35D1/THSD7A/VCAN | 16 |
| R-HSA-4608870 | Asymmetric localization of PCP proteins | 5/597 | 64/10867 | 2.7E-01 | 6.7E-01 | 6.0E-01 | FZD1/PSMD11/PSMD7/PSMD8/WNT5A | 5 |
| R-HSA-110320 | Translesion Synthesis by POLH | 2/597 | 19/10867 | 2.8E-01 | 6.7E-01 | 6.0E-01 | PCNA/RFC5 | 2 |
| R-HSA-1362277 | Transcription of E2F targets under negative control by DREAM complex | 2/597 | 19/10867 | 2.8E-01 | 6.7E-01 | 6.0E-01 | CDC25A/PCNA | 2 |
| R-HSA-1482925 | Acyl chain remodelling of PG | 2/597 | 19/10867 | 2.8E-01 | 6.7E-01 | 6.0E-01 | PLA2G2A/PLA2G4A | 2 |
| R-HSA-2995383 | Initiation of Nuclear Envelope (NE) Reformation | 2/597 | 19/10867 | 2.8E-01 | 6.7E-01 | 6.0E-01 | CCNB2/LMNB1 | 2 |
| R-HSA-392851 | Prostacyclin signalling through prostacyclin receptor | 2/597 | 19/10867 | 2.8E-01 | 6.7E-01 | 6.0E-01 | GNB4/GNG12 | 2 |
| R-HSA-416700 | Other semaphorin interactions | 2/597 | 19/10867 | 2.8E-01 | 6.7E-01 | 6.0E-01 | ITGB1/SEMA6D | 2 |
| R-HSA-422085 | Synthesis. secretion. and deacylation of Ghrelin | 2/597 | 19/10867 | 2.8E-01 | 6.7E-01 | 6.0E-01 | BCHE/IGF1 | 2 |
| R-HSA-8851708 | Signaling by FGFR2 IIIa TM | 2/597 | 19/10867 | 2.8E-01 | 6.7E-01 | 6.0E-01 | FGF1/FGF2 | 2 |
| R-HSA-8963889 | Assembly of active LPL and LIPC lipase complexes | 2/597 | 19/10867 | 2.8E-01 | 6.7E-01 | 6.0E-01 | CIDEC/PCSK5 | 2 |
| R-HSA-9018678 | Biosynthesis of specialized proresolving mediators (SPMs) | 2/597 | 19/10867 | 2.8E-01 | 6.7E-01 | 6.0E-01 | EPHX2/PTGS2 | 2 |
| R-HSA-9703465 | Signaling by FLT3 fusion proteins | 2/597 | 19/10867 | 2.8E-01 | 6.7E-01 | 6.0E-01 | NRAS/PIK3R1 | 2 |
| R-HSA-391251 | Protein folding | 7/597 | 97/10867 | 2.8E-01 | 6.8E-01 | 6.1E-01 | CCNE2/CCT8/GNB4/GNG12/SKIV2L/TCP1/TUBA1C | 7 |
| R-HSA-5334118 | DNA methylation | 5/597 | 65/10867 | 2.9E-01 | 6.8E-01 | 6.1E-01 | DNMT3B/H2AX/H2BC13/H2BC5/UHRF1 | 5 |
| R-HSA-176408 | Regulation of APC/C activators between G1/S and early anaphase | 6/597 | 81/10867 | 2.9E-01 | 6.8E-01 | 6.1E-01 | ANAPC2/CCNA2/MAD2L1/PSMD11/PSMD7/PSMD8 | 6 |
| R-HSA-187687 | Signalling to ERKs | 3/597 | 34/10867 | 2.9E-01 | 6.8E-01 | 6.1E-01 | NRAS/RALA/RAPGEF1 | 3 |
| R-HSA-4791275 | Signaling by WNT in cancer | 3/597 | 34/10867 | 2.9E-01 | 6.8E-01 | 6.1E-01 | AMER1/CTNNB1/GSK3B | 3 |
| R-HSA-5654727 | Negative regulation of FGFR2 signaling | 3/597 | 34/10867 | 2.9E-01 | 6.8E-01 | 6.1E-01 | FGF1/FGF18/FGF2 | 3 |
| R-HSA-5693537 | Resolution of D-Loop Structures | 3/597 | 34/10867 | 2.9E-01 | 6.8E-01 | 6.1E-01 | BLM/DNA2/EXO1 | 3 |
| R-HSA-397014 | Muscle contraction | 13/597 | 197/10867 | 2.9E-01 | 6.8E-01 | 6.1E-01 | ANXA1/ATP1A2/CALD1/DMPK/GUCY1A1/KCNK10/MYBPC1/NPR2/SCN1B/SCN2A/TNNC1/VCL/WWTR1 | 13 |
| R-HSA-177929 | Signaling by EGFR | 4/597 | 50/10867 | 2.9E-01 | 6.9E-01 | 6.2E-01 | ADAM12/LRIG1/NRAS/PIK3R1 | 4 |
| R-HSA-1234176 | Oxygen-dependent proline hydroxylation of Hypoxia-inducible Factor Alpha | 5/597 | 66/10867 | 3.0E-01 | 6.9E-01 | 6.2E-01 | EGLN2/HIF3A/PSMD11/PSMD7/PSMD8 | 5 |
| R-HSA-69563 | p53-Dependent G1 DNA Damage Response | 5/597 | 66/10867 | 3.0E-01 | 6.9E-01 | 6.2E-01 | CCNA2/CCNE2/PSMD11/PSMD7/PSMD8 | 5 |
| R-HSA-69580 | p53-Dependent G1/S DNA damage checkpoint | 5/597 | 66/10867 | 3.0E-01 | 6.9E-01 | 6.2E-01 | CCNA2/CCNE2/PSMD11/PSMD7/PSMD8 | 5 |
| R-HSA-167044 | Signalling to RAS | 2/597 | 20/10867 | 3.0E-01 | 6.9E-01 | 6.2E-01 | NRAS/RALA | 2 |
| R-HSA-388844 | Receptor-type tyrosine-protein phosphatases | 2/597 | 20/10867 | 3.0E-01 | 6.9E-01 | 6.2E-01 | IL1RAP/PTPRD | 2 |
| R-HSA-418217 | G beta:gamma signalling through PLC beta | 2/597 | 20/10867 | 3.0E-01 | 6.9E-01 | 6.2E-01 | GNB4/GNG12 | 2 |
| R-HSA-5625900 | RHO GTPases activate CIT | 2/597 | 20/10867 | 3.0E-01 | 6.9E-01 | 6.2E-01 | PPP1CB/RHOB | 2 |
| R-HSA-5627117 | RHO GTPases Activate ROCKs | 2/597 | 20/10867 | 3.0E-01 | 6.9E-01 | 6.2E-01 | PPP1CB/RHOB | 2 |
| R-HSA-8876384 | Listeria monocytogenes entry into host cells | 2/597 | 20/10867 | 3.0E-01 | 6.9E-01 | 6.2E-01 | CTNNB1/MET | 2 |
| R-HSA-8964616 | G beta:gamma signalling through CDC42 | 2/597 | 20/10867 | 3.0E-01 | 6.9E-01 | 6.2E-01 | GNB4/GNG12 | 2 |
| R-HSA-8978934 | Metabolism of cofactors | 2/597 | 20/10867 | 3.0E-01 | 6.9E-01 | 6.2E-01 | HSP90AA1/NOS3 | 2 |
| R-HSA-9671555 | Signaling by PDGFR in disease | 2/597 | 20/10867 | 3.0E-01 | 6.9E-01 | 6.2E-01 | NRAS/PIK3R1 | 2 |
| R-HSA-9687136 | Aberrant regulation of mitotic exit in cancer due to RB1 defects | 2/597 | 20/10867 | 3.0E-01 | 6.9E-01 | 6.2E-01 | ANAPC2/SKP2 | 2 |
| R-HSA-3301854 | Nuclear Pore Complex (NPC) Disassembly | 3/597 | 35/10867 | 3.0E-01 | 6.9E-01 | 6.2E-01 | CCNB2/NUP160/RAE1 | 3 |
| R-HSA-8953750 | Transcriptional Regulation by E2F6 | 3/597 | 35/10867 | 3.0E-01 | 6.9E-01 | 6.2E-01 | EZH2/RING1/RRM2 | 3 |
| R-HSA-936440 | Negative regulators of DDX58/IFIH1 signaling | 3/597 | 35/10867 | 3.0E-01 | 6.9E-01 | 6.2E-01 | NLRX1/PIN1/TNFAIP3 | 3 |
| R-HSA-350562 | Regulation of ornithine decarboxylase (ODC) | 4/597 | 51/10867 | 3.1E-01 | 7.0E-01 | 6.3E-01 | OAZ1/PSMD11/PSMD7/PSMD8 | 4 |
| R-HSA-5685942 | HDR through Homologous Recombination (HRR) | 5/597 | 67/10867 | 3.1E-01 | 7.0E-01 | 6.3E-01 | BLM/DNA2/EXO1/PCNA/RFC5 | 5 |
| R-HSA-6802952 | Signaling by BRAF and RAF1 fusions | 5/597 | 67/10867 | 3.1E-01 | 7.0E-01 | 6.3E-01 | ESRP1/IQGAP1/ITGB3/NRAS/VCL | 5 |
| R-HSA-73856 | RNA Polymerase II Transcription Termination | 5/597 | 67/10867 | 3.1E-01 | 7.0E-01 | 6.3E-01 | CPSF4/DDX39B/FYTTD1/SRSF1/SRSF5 | 5 |
| R-HSA-9018519 | Estrogen-dependent gene expression | 10/597 | 150/10867 | 3.1E-01 | 7.1E-01 | 6.3E-01 | FKBP4/FOXA1/H2AX/H2BC13/H2BC5/HSP90AA1/KPNA2/RUNX1/SMC1A/USF2 | 10 |
| R-HSA-15869 | Metabolism of nucleotides | 7/597 | 100/10867 | 3.1E-01 | 7.1E-01 | 6.3E-01 | AK5/ENTPD7/GMPS/HPRT1/NUDT5/RRM2/TK1 | 7 |
| R-HSA-446203 | Asparagine N-linked glycosylation | 19/597 | 304/10867 | 3.1E-01 | 7.1E-01 | 6.3E-01 | ALG13/ALG3/AMDHD2/B4GALT5/CALR/CD59/COPG1/LMAN2/MCFD2/MGAT3/MOGS/PDIA3/PRKCSH/RPN2/SEC23A/SLC17A5/ST6GALNAC1/ST6GALNAC3/TUBA1C | 19 |
| R-HSA-1989781 | PPARA activates gene expression | 8/597 | 117/10867 | 3.1E-01 | 7.1E-01 | 6.3E-01 | ESRRA/G0S2/HMGCS1/MED14/MED16/MED20/NPAS2/PPARG | 8 |
| R-HSA-1222556 | ROS and RNS production in phagocytes | 3/597 | 36/10867 | 3.2E-01 | 7.1E-01 | 6.3E-01 | HVCN1/NOS3/RAC2 | 3 |
| R-HSA-1266695 | Interleukin-7 signaling | 3/597 | 36/10867 | 3.2E-01 | 7.1E-01 | 6.3E-01 | IL7/IRS2/PIK3R1 | 3 |
| R-HSA-5673000 | RAF activation | 3/597 | 36/10867 | 3.2E-01 | 7.1E-01 | 6.3E-01 | MAP3K11/NRAS/PPP1CB | 3 |
| R-HSA-5658442 | Regulation of RAS by GAPs | 5/597 | 68/10867 | 3.2E-01 | 7.1E-01 | 6.3E-01 | CUL3/NRAS/PSMD11/PSMD7/PSMD8 | 5 |
| R-HSA-9013149 | RAC1 GTPase cycle | 12/597 | 185/10867 | 3.2E-01 | 7.1E-01 | 6.3E-01 | ARHGAP20/CAV1/CDC42EP1/EPHA2/FERMT2/IQGAP1/ITGB1/NISCH/PIK3R1/PLEKHG1/SRGAP2/WASF1 | 12 |
| R-HSA-69601 | Ubiquitin Mediated Degradation of Phosphorylated Cdc25A | 4/597 | 52/10867 | 3.2E-01 | 7.1E-01 | 6.3E-01 | CDC25A/PSMD11/PSMD7/PSMD8 | 4 |
| R-HSA-69610 | p53-Independent DNA Damage Response | 4/597 | 52/10867 | 3.2E-01 | 7.1E-01 | 6.3E-01 | CDC25A/PSMD11/PSMD7/PSMD8 | 4 |
| R-HSA-69613 | p53-Independent G1/S DNA damage checkpoint | 4/597 | 52/10867 | 3.2E-01 | 7.1E-01 | 6.3E-01 | CDC25A/PSMD11/PSMD7/PSMD8 | 4 |
| R-HSA-75815 | Ubiquitin-dependent degradation of Cyclin D | 4/597 | 52/10867 | 3.2E-01 | 7.1E-01 | 6.3E-01 | GSK3B/PSMD11/PSMD7/PSMD8 | 4 |
| R-HSA-141405 | Inhibition of the proteolytic activity of APC/C required for the onset of anaphase by mitotic spindle checkpoint components | 2/597 | 21/10867 | 3.2E-01 | 7.1E-01 | 6.3E-01 | ANAPC2/MAD2L1 | 2 |
| R-HSA-141430 | Inactivation of APC/C via direct inhibition of the APC/C complex | 2/597 | 21/10867 | 3.2E-01 | 7.1E-01 | 6.3E-01 | ANAPC2/MAD2L1 | 2 |
| R-HSA-164938 | Nef-mediates down modulation of cell surface receptors by recruiting them to clathrin adapters | 2/597 | 21/10867 | 3.2E-01 | 7.1E-01 | 6.3E-01 | HLA-A/LCK | 2 |
| R-HSA-389513 | CTLA4 inhibitory signaling | 2/597 | 21/10867 | 3.2E-01 | 7.1E-01 | 6.3E-01 | FYN/LCK | 2 |
| R-HSA-416572 | Sema4D induced cell migration and growth-cone collapse | 2/597 | 21/10867 | 3.2E-01 | 7.1E-01 | 6.3E-01 | ARHGEF12/RHOB | 2 |
| R-HSA-445144 | Signal transduction by L1 | 2/597 | 21/10867 | 3.2E-01 | 7.1E-01 | 6.3E-01 | ITGB1/ITGB3 | 2 |
| R-HSA-500657 | Presynaptic function of Kainate receptors | 2/597 | 21/10867 | 3.2E-01 | 7.1E-01 | 6.3E-01 | GNB4/GNG12 | 2 |
| R-HSA-5218921 | VEGFR2 mediated cell proliferation | 2/597 | 21/10867 | 3.2E-01 | 7.1E-01 | 6.3E-01 | NRAS/PRKCA | 2 |
| R-HSA-5651801 | PCNA-Dependent Long Patch Base Excision Repair | 2/597 | 21/10867 | 3.2E-01 | 7.1E-01 | 6.3E-01 | PCNA/RFC5 | 2 |
| R-HSA-6807004 | Negative regulation of MET activity | 2/597 | 21/10867 | 3.2E-01 | 7.1E-01 | 6.3E-01 | LRIG1/MET | 2 |
| R-HSA-69002 | DNA Replication Pre-Initiation | 6/597 | 85/10867 | 3.2E-01 | 7.1E-01 | 6.4E-01 | CDT1/MCM3/MCM4/PSMD11/PSMD7/PSMD8 | 6 |
| R-HSA-8948751 | Regulation of PTEN stability and activity | 5/597 | 69/10867 | 3.3E-01 | 7.2E-01 | 6.4E-01 | NEDD4/PSMD11/PSMD7/PSMD8/PTEN | 5 |
| R-HSA-400206 | Regulation of lipid metabolism by PPARalpha | 8/597 | 119/10867 | 3.3E-01 | 7.2E-01 | 6.5E-01 | ESRRA/G0S2/HMGCS1/MED14/MED16/MED20/NPAS2/PPARG | 8 |
| R-HSA-140875 | Common Pathway of Fibrin Clot Formation | 2/597 | 22/10867 | 3.4E-01 | 7.4E-01 | 6.6E-01 | PROCR/PROS1 | 2 |
| R-HSA-198753 | ERK/MAPK targets | 2/597 | 22/10867 | 3.4E-01 | 7.4E-01 | 6.6E-01 | DUSP6/VRK3 | 2 |
| R-HSA-392170 | ADP signalling through P2Y purinoceptor 12 | 2/597 | 22/10867 | 3.4E-01 | 7.4E-01 | 6.6E-01 | GNB4/GNG12 | 2 |
| R-HSA-5628897 | TP53 Regulates Metabolic Genes | 6/597 | 87/10867 | 3.4E-01 | 7.4E-01 | 6.6E-01 | LAMTOR4/PRKAA1/PRKAG1/PTEN/RRAGC/YWHAG | 6 |
| R-HSA-8848021 | Signaling by PTK6 | 4/597 | 54/10867 | 3.4E-01 | 7.4E-01 | 6.6E-01 | ERBB3/GPNMB/NR3C1/NRAS | 4 |
| R-HSA-8854050 | FBXL7 down-regulates AURKA during mitotic entry and in early mitosis | 4/597 | 54/10867 | 3.4E-01 | 7.4E-01 | 6.6E-01 | AURKA/PSMD11/PSMD7/PSMD8 | 4 |
| R-HSA-9006927 | Signaling by Non-Receptor Tyrosine Kinases | 4/597 | 54/10867 | 3.4E-01 | 7.4E-01 | 6.6E-01 | ERBB3/GPNMB/NR3C1/NRAS | 4 |
| R-HSA-9604323 | Negative regulation of NOTCH4 signaling | 4/597 | 54/10867 | 3.4E-01 | 7.4E-01 | 6.6E-01 | PSMD11/PSMD7/PSMD8/TACC3 | 4 |
| R-HSA-9662361 | Sensory processing of sound by outer hair cells of the cochlea | 4/597 | 54/10867 | 3.4E-01 | 7.4E-01 | 6.6E-01 | EPS8L2/MSN/PJVK/TRIOBP | 4 |
| R-HSA-9013407 | RHOH GTPase cycle | 3/597 | 38/10867 | 3.5E-01 | 7.5E-01 | 6.7E-01 | CAV1/LCK/OSBPL11 | 3 |
| R-HSA-418346 | Platelet homeostasis | 6/597 | 88/10867 | 3.5E-01 | 7.6E-01 | 6.8E-01 | GNB4/GNG12/GUCY1A1/NOS3/PLA2G4A/PRKG1 | 6 |
| R-HSA-450531 | Regulation of mRNA stability by proteins that bind AU-rich elements | 6/597 | 88/10867 | 3.5E-01 | 7.6E-01 | 6.8E-01 | ELAVL1/PRKCA/PSMD11/PSMD7/PSMD8/ZFP36 | 6 |
| R-HSA-1638091 | Heparan sulfate/heparin (HS-GAG) metabolism | 4/597 | 55/10867 | 3.6E-01 | 7.6E-01 | 6.8E-01 | DCN/HS3ST3A1/SDC1/VCAN | 4 |
| R-HSA-429914 | Deadenylation-dependent mRNA decay | 4/597 | 55/10867 | 3.6E-01 | 7.6E-01 | 6.8E-01 | CNOT1/CNOT11/SKIV2L/TNKS1BP1 | 4 |
| R-HSA-199992 | trans-Golgi Network Vesicle Budding | 5/597 | 72/10867 | 3.6E-01 | 7.6E-01 | 6.8E-01 | CLTC/HIP1R/SH3D19/SNX2/VAMP7 | 5 |
| R-HSA-112409 | RAF-independent MAPK1/3 activation | 2/597 | 23/10867 | 3.6E-01 | 7.6E-01 | 6.8E-01 | DUSP6/TYK2 | 2 |
| R-HSA-1482801 | Acyl chain remodelling of PS | 2/597 | 23/10867 | 3.6E-01 | 7.6E-01 | 6.8E-01 | PLA2G2A/PLA2G4A | 2 |
| R-HSA-5620922 | BBSome-mediated cargo-targeting to cilium | 2/597 | 23/10867 | 3.6E-01 | 7.6E-01 | 6.8E-01 | CCT8/TCP1 | 2 |
| R-HSA-73980 | RNA Polymerase III Transcription Termination | 2/597 | 23/10867 | 3.6E-01 | 7.6E-01 | 6.8E-01 | POLR3B/POLR3GL | 2 |
| R-HSA-8956321 | Nucleotide salvage | 2/597 | 23/10867 | 3.6E-01 | 7.6E-01 | 6.8E-01 | HPRT1/TK1 | 2 |
| R-HSA-9013422 | RHOBTB1 GTPase cycle | 2/597 | 23/10867 | 3.6E-01 | 7.6E-01 | 6.8E-01 | CUL3/STK38 | 2 |
| R-HSA-9637690 | Response of Mtb to phagocytosis | 2/597 | 23/10867 | 3.6E-01 | 7.6E-01 | 6.8E-01 | GSK3A/KPNA1 | 2 |
| R-HSA-111996 | Ca-dependent events | 3/597 | 39/10867 | 3.6E-01 | 7.6E-01 | 6.8E-01 | KPNA2/PLA2G4A/PRKCA | 3 |
| R-HSA-5689896 | Ovarian tumor domain proteases | 3/597 | 39/10867 | 3.6E-01 | 7.6E-01 | 6.8E-01 | PTEN/TNFAIP3/TNIP2 | 3 |
| R-HSA-3371556 | Cellular response to heat stress | 6/597 | 89/10867 | 3.6E-01 | 7.6E-01 | 6.8E-01 | FKBP4/GSK3B/HSBP1/HSP90AA1/NUP160/RAE1 | 6 |
| R-HSA-72203 | Processing of Capped Intron-Containing Pre-mRNA | 15/597 | 244/10867 | 3.6E-01 | 7.6E-01 | 6.8E-01 | CPSF4/DDX39B/DHX9/ELAVL1/FUS/FYTTD1/HNRNPA0/HNRNPA2B1/NUP160/RAE1/SNRNP70/SRSF1/SRSF5/WBP11/XAB2 | 15 |
| R-HSA-211945 | Phase I - Functionalization of compounds | 7/597 | 106/10867 | 3.6E-01 | 7.6E-01 | 6.8E-01 | ACSS1/ADH1A/ADH1C/ALDH1A1/ALDH3A1/CYP4F12/MAOA | 7 |
| R-HSA-2151201 | Transcriptional activation of mitochondrial biogenesis | 4/597 | 56/10867 | 3.7E-01 | 7.7E-01 | 6.9E-01 | ATP5F1B/ESRRA/IDH2/MEF2D | 4 |
| R-HSA-212300 | PRC2 methylates histones and DNA | 5/597 | 73/10867 | 3.7E-01 | 7.7E-01 | 6.9E-01 | DNMT3B/EZH2/H2AX/H2BC13/H2BC5 | 5 |
| R-HSA-9710421 | Defective pyroptosis | 5/597 | 73/10867 | 3.7E-01 | 7.7E-01 | 6.9E-01 | DNMT3B/EZH2/H2AX/H2BC13/H2BC5 | 5 |
| R-HSA-72306 | tRNA processing | 7/597 | 107/10867 | 3.7E-01 | 7.7E-01 | 6.9E-01 | CPSF4/NUP160/PUS1/QTRT1/RAE1/TRMT9B/XPOT | 7 |
| R-HSA-1236974 | ER-Phagosome pathway | 6/597 | 90/10867 | 3.7E-01 | 7.7E-01 | 6.9E-01 | CALR/HLA-A/PDIA3/PSMD11/PSMD7/PSMD8 | 6 |
| R-HSA-6783310 | Fanconi Anemia Pathway | 3/597 | 40/10867 | 3.8E-01 | 7.8E-01 | 7.0E-01 | ERCC1/FANCI/UBE2T | 3 |
| R-HSA-8853659 | RET signaling | 3/597 | 40/10867 | 3.8E-01 | 7.8E-01 | 7.0E-01 | IRS2/PIK3R1/PRKCA | 3 |
| R-HSA-73894 | DNA Repair | 20/597 | 335/10867 | 3.8E-01 | 7.8E-01 | 7.0E-01 | ASCC1/BLM/CCNA2/DNA2/ERCC1/EXO1/FANCI/H2AX/H2BC13/H2BC5/KPNA2/MSH6/PCNA/POLQ/RFC5/RNF168/TDG/UBE2T/UNG/XAB2 | 20 |
| R-HSA-166208 | mTORC1-mediated signalling | 2/597 | 24/10867 | 3.8E-01 | 7.8E-01 | 7.0E-01 | LAMTOR4/RRAGC | 2 |
| R-HSA-202040 | G-protein activation | 2/597 | 24/10867 | 3.8E-01 | 7.8E-01 | 7.0E-01 | GNB4/GNG12 | 2 |
| R-HSA-379716 | Cytosolic tRNA aminoacylation | 2/597 | 24/10867 | 3.8E-01 | 7.8E-01 | 7.0E-01 | GARS1/MARS1 | 2 |
| R-HSA-428930 | Thromboxane signalling through TP receptor | 2/597 | 24/10867 | 3.8E-01 | 7.8E-01 | 7.0E-01 | GNB4/GNG12 | 2 |
| R-HSA-75876 | Synthesis of very long-chain fatty acyl-CoAs | 2/597 | 24/10867 | 3.8E-01 | 7.8E-01 | 7.0E-01 | ACSL4/ACSL5 | 2 |
| R-HSA-9634638 | Estrogen-dependent nuclear events downstream of ESR-membrane signaling | 2/597 | 24/10867 | 3.8E-01 | 7.8E-01 | 7.0E-01 | FOXO3/SRF | 2 |
| R-HSA-982772 | Growth hormone receptor signaling | 2/597 | 24/10867 | 3.8E-01 | 7.8E-01 | 7.0E-01 | IRS2/SH2B1 | 2 |
| R-HSA-4641258 | Degradation of DVL | 4/597 | 57/10867 | 3.8E-01 | 7.8E-01 | 7.0E-01 | CUL3/PSMD11/PSMD7/PSMD8 | 4 |
| R-HSA-6784531 | tRNA processing in the nucleus | 4/597 | 57/10867 | 3.8E-01 | 7.8E-01 | 7.0E-01 | CPSF4/NUP160/RAE1/XPOT | 4 |
| R-HSA-9013106 | RHOC GTPase cycle | 5/597 | 74/10867 | 3.8E-01 | 7.8E-01 | 7.0E-01 | ARHGEF12/CAV1/DAAM1/IQGAP1/PIK3R1 | 5 |
| R-HSA-1912422 | Pre-NOTCH Expression and Processing | 7/597 | 109/10867 | 3.9E-01 | 7.9E-01 | 7.1E-01 | H2AX/H2BC13/H2BC5/MAML2/NOTCH2/PRKCI/RUNX1 | 7 |
| R-HSA-159231 | Transport of Mature mRNA Derived from an Intronless Transcript | 3/597 | 41/10867 | 3.9E-01 | 7.9E-01 | 7.1E-01 | CPSF4/NUP160/RAE1 | 3 |
| R-HSA-5696400 | Dual Incision in GG-NER | 3/597 | 41/10867 | 3.9E-01 | 7.9E-01 | 7.1E-01 | ERCC1/PCNA/RFC5 | 3 |
| R-HSA-111885 | Opioid Signalling | 6/597 | 92/10867 | 3.9E-01 | 7.9E-01 | 7.1E-01 | GNB4/GNG12/KPNA2/PLA2G4A/PPP1CA/PRKCA | 6 |
| R-HSA-1234174 | Cellular response to hypoxia | 5/597 | 75/10867 | 4.0E-01 | 7.9E-01 | 7.1E-01 | EGLN2/HIF3A/PSMD11/PSMD7/PSMD8 | 5 |
| R-HSA-2995410 | Nuclear Envelope (NE) Reassembly | 5/597 | 75/10867 | 4.0E-01 | 7.9E-01 | 7.1E-01 | CCNB2/CHMP3/LMNB1/NUP160/TUBA1C | 5 |
| R-HSA-166658 | Complement cascade | 4/597 | 58/10867 | 4.0E-01 | 7.9E-01 | 7.1E-01 | CD59/CFD/CFI/PROS1 | 4 |
| R-HSA-210991 | Basigin interactions | 2/597 | 25/10867 | 4.0E-01 | 8.0E-01 | 7.2E-01 | CAV1/ITGB1 | 2 |
| R-HSA-392451 | G beta:gamma signalling through PI3Kgamma | 2/597 | 25/10867 | 4.0E-01 | 8.0E-01 | 7.2E-01 | GNB4/GNG12 | 2 |
| R-HSA-5696397 | Gap-filling DNA repair synthesis and ligation in GG-NER | 2/597 | 25/10867 | 4.0E-01 | 8.0E-01 | 7.2E-01 | PCNA/RFC5 | 2 |
| R-HSA-8854691 | Interleukin-20 family signaling | 2/597 | 25/10867 | 4.0E-01 | 8.0E-01 | 7.2E-01 | STAT4/TYK2 | 2 |
| R-HSA-9013508 | NOTCH3 Intracellular Domain Regulates Transcription | 2/597 | 25/10867 | 4.0E-01 | 8.0E-01 | 7.2E-01 | HEY1/MAML2 | 2 |
| R-HSA-9705462 | Inactivation of CSF3 (G-CSF) signaling | 2/597 | 25/10867 | 4.0E-01 | 8.0E-01 | 7.2E-01 | HCK/TYK2 | 2 |
| R-HSA-163125 | Post-translational modification: synthesis of GPI-anchored proteins | 6/597 | 93/10867 | 4.0E-01 | 8.0E-01 | 7.2E-01 | LY6D/PIGP/PIGT/PIGW/RECK/ULBP2 | 6 |
| R-HSA-442755 | Activation of NMDA receptors and postsynaptic events | 6/597 | 93/10867 | 4.0E-01 | 8.0E-01 | 7.2E-01 | KPNA2/NRAS/PPM1F/PRKAA1/PRKAG1/TUBA1C | 6 |
| R-HSA-351202 | Metabolism of polyamines | 4/597 | 59/10867 | 4.1E-01 | 8.0E-01 | 7.2E-01 | OAZ1/PSMD11/PSMD7/PSMD8 | 4 |
| R-HSA-5676590 | NIK-->noncanonical NF-kB signaling | 4/597 | 59/10867 | 4.1E-01 | 8.0E-01 | 7.2E-01 | PSMD11/PSMD7/PSMD8/RELB | 4 |
| R-HSA-68827 | CDT1 association with the CDC6:ORC:origin complex | 4/597 | 59/10867 | 4.1E-01 | 8.0E-01 | 7.2E-01 | CDT1/PSMD11/PSMD7/PSMD8 | 4 |
| R-HSA-9013406 | RHOQ GTPase cycle | 4/597 | 59/10867 | 4.1E-01 | 8.0E-01 | 7.2E-01 | CAV1/CDC42EP1/IQGAP1/SRGAP2 | 4 |
| R-HSA-983189 | Kinesins | 4/597 | 59/10867 | 4.1E-01 | 8.0E-01 | 7.2E-01 | KIF18B/KIF20A/KIF26B/TUBA1C | 4 |
| R-HSA-159234 | Transport of Mature mRNAs Derived from Intronless Transcripts | 3/597 | 42/10867 | 4.1E-01 | 8.0E-01 | 7.2E-01 | CPSF4/NUP160/RAE1 | 3 |
| R-HSA-3769402 | Deactivation of the beta-catenin transactivating complex | 3/597 | 42/10867 | 4.1E-01 | 8.0E-01 | 7.2E-01 | CTNNB1/SOX4/TLE4 | 3 |
| R-HSA-381676 | Glucagon-like Peptide-1 (GLP1) regulates insulin secretion | 3/597 | 42/10867 | 4.1E-01 | 8.0E-01 | 7.2E-01 | GNB4/GNG12/IQGAP1 | 3 |
| R-HSA-3928662 | EPHB-mediated forward signaling | 3/597 | 42/10867 | 4.1E-01 | 8.0E-01 | 7.2E-01 | EFNB2/EPHB6/FYN | 3 |
| R-HSA-5173105 | O-linked glycosylation | 7/597 | 111/10867 | 4.1E-01 | 8.0E-01 | 7.2E-01 | ADAMTS2/B4GALT5/GALNT1/GALNT7/POMK/ST6GALNAC3/THSD7A | 7 |
| R-HSA-380320 | Recruitment of NuMA to mitotic centrosomes | 6/597 | 94/10867 | 4.1E-01 | 8.0E-01 | 7.2E-01 | HSP90AA1/MAPRE1/TUBA1C/TUBG1/TUBGCP6/YWHAG | 6 |
| R-HSA-5607761 | Dectin-1 mediated noncanonical NF-kB signaling | 4/597 | 60/10867 | 4.2E-01 | 8.0E-01 | 7.2E-01 | PSMD11/PSMD7/PSMD8/RELB | 4 |
| R-HSA-5610783 | Degradation of GLI2 by the proteasome | 4/597 | 60/10867 | 4.2E-01 | 8.0E-01 | 7.2E-01 | GSK3B/PSMD11/PSMD7/PSMD8 | 4 |
| R-HSA-5610785 | GLI3 is processed to GLI3R by the proteasome | 4/597 | 60/10867 | 4.2E-01 | 8.0E-01 | 7.2E-01 | GSK3B/PSMD11/PSMD7/PSMD8 | 4 |
| R-HSA-110373 | Resolution of AP sites via the multiple-nucleotide patch replacement pathway | 2/597 | 26/10867 | 4.2E-01 | 8.0E-01 | 7.2E-01 | PCNA/RFC5 | 2 |
| R-HSA-179409 | APC-Cdc20 mediated degradation of Nek2A | 2/597 | 26/10867 | 4.2E-01 | 8.0E-01 | 7.2E-01 | ANAPC2/MAD2L1 | 2 |
| R-HSA-2173788 | Downregulation of TGF-beta receptor signaling | 2/597 | 26/10867 | 4.2E-01 | 8.0E-01 | 7.2E-01 | PPP1CA/PPP1CB | 2 |
| R-HSA-6803529 | FGFR2 alternative splicing | 2/597 | 26/10867 | 4.2E-01 | 8.0E-01 | 7.2E-01 | ESRP1/RBFOX2 | 2 |
| R-HSA-975576 | N-glycan antennae elongation in the medial/trans-Golgi | 2/597 | 26/10867 | 4.2E-01 | 8.0E-01 | 7.2E-01 | B4GALT5/MGAT3 | 2 |
| R-HSA-432040 | Vasopressin regulates renal water homeostasis via Aquaporins | 3/597 | 43/10867 | 4.2E-01 | 8.0E-01 | 7.2E-01 | GNB4/GNG12/RAB11FIP2 | 3 |
| R-HSA-6782315 | tRNA modification in the nucleus and cytosol | 3/597 | 43/10867 | 4.2E-01 | 8.0E-01 | 7.2E-01 | PUS1/QTRT1/TRMT9B | 3 |
| R-HSA-5625740 | RHO GTPases activate PKNs | 6/597 | 95/10867 | 4.2E-01 | 8.0E-01 | 7.2E-01 | H2AX/H2BC13/H2BC5/PPP1CB/RHOB/YWHAG | 6 |
| R-HSA-72163 | mRNA Splicing - Major Pathway | 11/597 | 183/10867 | 4.2E-01 | 8.0E-01 | 7.2E-01 | CPSF4/DHX9/ELAVL1/FUS/HNRNPA0/HNRNPA2B1/SNRNP70/SRSF1/SRSF5/WBP11/XAB2 | 11 |
| R-HSA-5693606 | DNA Double Strand Break Response | 5/597 | 78/10867 | 4.3E-01 | 8.0E-01 | 7.2E-01 | H2AX/H2BC13/H2BC5/KPNA2/RNF168 | 5 |
| R-HSA-110056 | MAPK3 (ERK1) activation | 1/597 | 10/10867 | 4.3E-01 | 8.0E-01 | 7.2E-01 | TYK2 | 1 |
| R-HSA-1250342 | PI3K events in ERBB4 signaling | 1/597 | 10/10867 | 4.3E-01 | 8.0E-01 | 7.2E-01 | PIK3R1 | 1 |
| R-HSA-159740 | Gamma-carboxylation of protein precursors | 1/597 | 10/10867 | 4.3E-01 | 8.0E-01 | 7.2E-01 | PROS1 | 1 |
| R-HSA-159782 | Removal of aminoterminal propeptides from gamma-carboxylated proteins | 1/597 | 10/10867 | 4.3E-01 | 8.0E-01 | 7.2E-01 | PROS1 | 1 |
| R-HSA-164940 | Nef mediated downregulation of MHC class I complex cell surface expression | 1/597 | 10/10867 | 4.3E-01 | 8.0E-01 | 7.2E-01 | HLA-A | 1 |
| R-HSA-209968 | Thyroxine biosynthesis | 1/597 | 10/10867 | 4.3E-01 | 8.0E-01 | 7.2E-01 | DIO2 | 1 |
| R-HSA-2151209 | Activation of PPARGC1A (PGC-1alpha) by phosphorylation | 1/597 | 10/10867 | 4.3E-01 | 8.0E-01 | 7.2E-01 | PRKAG1 | 1 |
| R-HSA-2161522 | Abacavir transport and metabolism | 1/597 | 10/10867 | 4.3E-01 | 8.0E-01 | 7.2E-01 | ADH1A | 1 |
| R-HSA-2465910 | MASTL Facilitates Mitotic Progression | 1/597 | 10/10867 | 4.3E-01 | 8.0E-01 | 7.2E-01 | MASTL | 1 |
| R-HSA-2470946 | Cohesin Loading onto Chromatin | 1/597 | 10/10867 | 4.3E-01 | 8.0E-01 | 7.2E-01 | SMC1A | 1 |
| R-HSA-399997 | Acetylcholine regulates insulin secretion | 1/597 | 10/10867 | 4.3E-01 | 8.0E-01 | 7.2E-01 | PRKCA | 1 |
| R-HSA-428542 | Regulation of commissural axon pathfinding by SLIT and ROBO | 1/597 | 10/10867 | 4.3E-01 | 8.0E-01 | 7.2E-01 | SLIT2 | 1 |
| R-HSA-428643 | Organic anion transporters | 1/597 | 10/10867 | 4.3E-01 | 8.0E-01 | 7.2E-01 | SLC17A5 | 1 |
| R-HSA-442729 | CREB1 phosphorylation through the activation of CaMKII/CaMKK/CaMKIV cascasde | 1/597 | 10/10867 | 4.3E-01 | 8.0E-01 | 7.2E-01 | KPNA2 | 1 |
| R-HSA-549127 | Organic cation transport | 1/597 | 10/10867 | 4.3E-01 | 8.0E-01 | 7.2E-01 | RUNX1 | 1 |
| R-HSA-5685939 | HDR through MMEJ (alt-NHEJ) | 1/597 | 10/10867 | 4.3E-01 | 8.0E-01 | 7.2E-01 | POLQ | 1 |
| R-HSA-74749 | Signal attenuation | 1/597 | 10/10867 | 4.3E-01 | 8.0E-01 | 7.2E-01 | IRS2 | 1 |
| R-HSA-8875656 | MET receptor recycling | 1/597 | 10/10867 | 4.3E-01 | 8.0E-01 | 7.2E-01 | MET | 1 |
| R-HSA-8877330 | RUNX1 and FOXP3 control the development of regulatory T lymphocytes (Tregs) | 1/597 | 10/10867 | 4.3E-01 | 8.0E-01 | 7.2E-01 | RUNX1 | 1 |
| R-HSA-8964058 | HDL remodeling | 1/597 | 10/10867 | 4.3E-01 | 8.0E-01 | 7.2E-01 | LIPG | 1 |
| R-HSA-9706019 | RHOBTB3 ATPase cycle | 1/597 | 10/10867 | 4.3E-01 | 8.0E-01 | 7.2E-01 | CUL3 | 1 |
| R-HSA-1221632 | Meiotic synapsis | 5/597 | 79/10867 | 4.4E-01 | 8.1E-01 | 7.3E-01 | H2AX/H2BC13/H2BC5/LMNB1/SMC1A | 5 |
| R-HSA-1368108 | BMAL1:CLOCK.NPAS2 activates circadian gene expression | 2/597 | 27/10867 | 4.4E-01 | 8.1E-01 | 7.3E-01 | BHLHE41/NPAS2 | 2 |
| R-HSA-912526 | Interleukin receptor SHC signaling | 2/597 | 27/10867 | 4.4E-01 | 8.1E-01 | 7.3E-01 | CSF2RA/PIK3R1 | 2 |
| R-HSA-9635486 | Infection with Mycobacterium tuberculosis | 2/597 | 27/10867 | 4.4E-01 | 8.1E-01 | 7.3E-01 | GSK3A/KPNA1 | 2 |
| R-HSA-9648002 | RAS processing | 2/597 | 27/10867 | 4.4E-01 | 8.1E-01 | 7.3E-01 | ABHD17C/NRAS | 2 |
| R-HSA-983231 | Factors involved in megakaryocyte development and platelet production | 10/597 | 168/10867 | 4.4E-01 | 8.2E-01 | 7.3E-01 | EHD1/EHD2/JMJD1C/KIF18B/KIF20A/KIF26B/MAFF/MICAL1/SH2B1/TUBA1C | 10 |
| R-HSA-8986944 | Transcriptional Regulation by MECP2 | 4/597 | 62/10867 | 4.5E-01 | 8.2E-01 | 7.3E-01 | AURKB/MET/PPARG/PTEN | 4 |
| R-HSA-8856688 | Golgi-to-ER retrograde transport | 8/597 | 133/10867 | 4.5E-01 | 8.2E-01 | 7.4E-01 | COPG1/GALNT1/KIF18B/KIF20A/KIF26B/PLA2G4A/SURF4/TUBA1C | 8 |
| R-HSA-416482 | G alpha (12/13) signalling events | 5/597 | 80/10867 | 4.5E-01 | 8.2E-01 | 7.4E-01 | ARHGEF12/ARHGEF3/GNB4/GNG12/RHOB | 5 |
| R-HSA-438064 | Post NMDA receptor activation events | 5/597 | 80/10867 | 4.5E-01 | 8.2E-01 | 7.4E-01 | KPNA2/NRAS/PRKAA1/PRKAG1/TUBA1C | 5 |
| R-HSA-202424 | Downstream TCR signaling | 6/597 | 98/10867 | 4.5E-01 | 8.2E-01 | 7.4E-01 | LCK/PIK3R1/PSMD11/PSMD7/PSMD8/PTEN | 6 |
| R-HSA-162587 | HIV Life Cycle | 9/597 | 152/10867 | 4.6E-01 | 8.2E-01 | 7.4E-01 | CHMP3/HMGA1/KPNA1/NELFB/NUP160/RAE1/TAF11/VPS37B/VTA1 | 9 |
| R-HSA-446219 | Synthesis of substrates in N-glycan biosythesis | 4/597 | 63/10867 | 4.6E-01 | 8.2E-01 | 7.4E-01 | AMDHD2/SLC17A5/ST6GALNAC1/ST6GALNAC3 | 4 |
| R-HSA-913709 | O-linked glycosylation of mucins | 4/597 | 63/10867 | 4.6E-01 | 8.2E-01 | 7.4E-01 | B4GALT5/GALNT1/GALNT7/ST6GALNAC3 | 4 |
| R-HSA-1482788 | Acyl chain remodelling of PC | 2/597 | 28/10867 | 4.6E-01 | 8.2E-01 | 7.4E-01 | PLA2G2A/PLA2G4A | 2 |
| R-HSA-9682385 | FLT3 signaling in disease | 2/597 | 28/10867 | 4.6E-01 | 8.2E-01 | 7.4E-01 | NRAS/PIK3R1 | 2 |
| R-HSA-6811434 | COPI-dependent Golgi-to-ER retrograde traffic | 6/597 | 99/10867 | 4.6E-01 | 8.2E-01 | 7.4E-01 | COPG1/KIF18B/KIF20A/KIF26B/SURF4/TUBA1C | 6 |
| R-HSA-1059683 | Interleukin-6 signaling | 1/597 | 11/10867 | 4.6E-01 | 8.2E-01 | 7.4E-01 | TYK2 | 1 |
| R-HSA-1234158 | Regulation of gene expression by Hypoxia-inducible Factor | 1/597 | 11/10867 | 4.6E-01 | 8.2E-01 | 7.4E-01 | HIF3A | 1 |
| R-HSA-1236977 | Endosomal/Vacuolar pathway | 1/597 | 11/10867 | 4.6E-01 | 8.2E-01 | 7.4E-01 | HLA-A | 1 |
| R-HSA-159854 | Gamma-carboxylation. transport. and amino-terminal cleavage of proteins | 1/597 | 11/10867 | 4.6E-01 | 8.2E-01 | 7.4E-01 | PROS1 | 1 |
| R-HSA-193048 | Androgen biosynthesis | 1/597 | 11/10867 | 4.6E-01 | 8.2E-01 | 7.4E-01 | SRD5A1 | 1 |
| R-HSA-196025 | Formation of annular gap junctions | 1/597 | 11/10867 | 4.6E-01 | 8.2E-01 | 7.4E-01 | CLTC | 1 |
| R-HSA-2468052 | Establishment of Sister Chromatid Cohesion | 1/597 | 11/10867 | 4.6E-01 | 8.2E-01 | 7.4E-01 | SMC1A | 1 |
| R-HSA-400451 | Free fatty acids regulate insulin secretion | 1/597 | 11/10867 | 4.6E-01 | 8.2E-01 | 7.4E-01 | ACSL4 | 1 |
| R-HSA-73614 | Pyrimidine salvage | 1/597 | 11/10867 | 4.6E-01 | 8.2E-01 | 7.4E-01 | TK1 | 1 |
| R-HSA-9020956 | Interleukin-27 signaling | 1/597 | 11/10867 | 4.6E-01 | 8.2E-01 | 7.4E-01 | TYK2 | 1 |
| R-HSA-9028731 | Activated NTRK2 signals through FRS2 and FRS3 | 1/597 | 11/10867 | 4.6E-01 | 8.2E-01 | 7.4E-01 | NRAS | 1 |
| R-HSA-9708530 | Regulation of BACH1 activity | 1/597 | 11/10867 | 4.6E-01 | 8.2E-01 | 7.4E-01 | SKP2 | 1 |
| R-HSA-168273 | Influenza Viral RNA Transcription and Replication | 8/597 | 135/10867 | 4.7E-01 | 8.2E-01 | 7.4E-01 | HSP90AA1/NUP160/RAE1/RPL10/RPL28/RPL31/RPS14/RPS9 | 8 |
| R-HSA-174084 | Autodegradation of Cdh1 by Cdh1:APC/C | 4/597 | 64/10867 | 4.7E-01 | 8.2E-01 | 7.4E-01 | ANAPC2/PSMD11/PSMD7/PSMD8 | 4 |
| R-HSA-421270 | Cell-cell junction organization | 4/597 | 64/10867 | 4.7E-01 | 8.2E-01 | 7.4E-01 | CDH17/CLDN15/CTNNB1/PRKCI | 4 |
| R-HSA-450294 | MAP kinase activation | 4/597 | 64/10867 | 4.7E-01 | 8.2E-01 | 7.4E-01 | DUSP6/MAP3K8/TNIP2/VRK3 | 4 |
| R-HSA-380270 | Recruitment of mitotic centrosome proteins and complexes | 5/597 | 82/10867 | 4.7E-01 | 8.2E-01 | 7.4E-01 | HSP90AA1/MAPRE1/TUBG1/TUBGCP6/YWHAG | 5 |
| R-HSA-380287 | Centrosome maturation | 5/597 | 82/10867 | 4.7E-01 | 8.2E-01 | 7.4E-01 | HSP90AA1/MAPRE1/TUBG1/TUBGCP6/YWHAG | 5 |
| R-HSA-1500620 | Meiosis | 7/597 | 118/10867 | 4.7E-01 | 8.2E-01 | 7.4E-01 | BLM/H2AX/H2BC13/H2BC5/LMNB1/PSMC3IP/SMC1A | 7 |
| R-HSA-1296041 | Activation of G protein gated Potassium channels | 2/597 | 29/10867 | 4.8E-01 | 8.2E-01 | 7.4E-01 | GNB4/GNG12 | 2 |
| R-HSA-1296059 | G protein gated Potassium channels | 2/597 | 29/10867 | 4.8E-01 | 8.2E-01 | 7.4E-01 | GNB4/GNG12 | 2 |
| R-HSA-420029 | Tight junction interactions | 2/597 | 29/10867 | 4.8E-01 | 8.2E-01 | 7.4E-01 | CLDN15/PRKCI | 2 |
| R-HSA-8863795 | Downregulation of ERBB2 signaling | 2/597 | 29/10867 | 4.8E-01 | 8.2E-01 | 7.4E-01 | ERBB3/HSP90AA1 | 2 |
| R-HSA-8982491 | Glycogen metabolism | 2/597 | 29/10867 | 4.8E-01 | 8.2E-01 | 7.4E-01 | AGL/PHKA1 | 2 |
| R-HSA-997272 | Inhibition of voltage gated Ca2+ channels via Gbeta/gamma subunits | 2/597 | 29/10867 | 4.8E-01 | 8.2E-01 | 7.4E-01 | GNB4/GNG12 | 2 |
| R-HSA-72172 | mRNA Splicing | 11/597 | 191/10867 | 4.8E-01 | 8.2E-01 | 7.4E-01 | CPSF4/DHX9/ELAVL1/FUS/HNRNPA0/HNRNPA2B1/SNRNP70/SRSF1/SRSF5/WBP11/XAB2 | 11 |
| R-HSA-437239 | Recycling pathway of L1 | 3/597 | 47/10867 | 4.8E-01 | 8.2E-01 | 7.4E-01 | CLTC/MSN/TUBA1C | 3 |
| R-HSA-69231 | Cyclin D associated events in G1 | 3/597 | 47/10867 | 4.8E-01 | 8.2E-01 | 7.4E-01 | CCNE2/CDKN1C/SKP2 | 3 |
| R-HSA-69236 | G1 Phase | 3/597 | 47/10867 | 4.8E-01 | 8.2E-01 | 7.4E-01 | CCNE2/CDKN1C/SKP2 | 3 |
| R-HSA-977606 | Regulation of Complement cascade | 3/597 | 47/10867 | 4.8E-01 | 8.2E-01 | 7.4E-01 | CD59/CFI/PROS1 | 3 |
| R-HSA-9633012 | Response of EIF2AK4 (GCN2) to amino acid deficiency | 6/597 | 101/10867 | 4.8E-01 | 8.2E-01 | 7.4E-01 | EIF2S1/RPL10/RPL28/RPL31/RPS14/RPS9 | 6 |
| R-HSA-202403 | TCR signaling | 7/597 | 120/10867 | 4.9E-01 | 8.2E-01 | 7.4E-01 | ENAH/LCK/PIK3R1/PSMD11/PSMD7/PSMD8/PTEN | 7 |
| R-HSA-111932 | CaMK IV-mediated phosphorylation of CREB | 1/597 | 12/10867 | 4.9E-01 | 8.2E-01 | 7.4E-01 | KPNA2 | 1 |
| R-HSA-1296346 | Tandem pore domain potassium channels | 1/597 | 12/10867 | 4.9E-01 | 8.2E-01 | 7.4E-01 | KCNK10 | 1 |
| R-HSA-170968 | Frs2-mediated activation | 1/597 | 12/10867 | 4.9E-01 | 8.2E-01 | 7.4E-01 | RAPGEF1 | 1 |
| R-HSA-190873 | Gap junction degradation | 1/597 | 12/10867 | 4.9E-01 | 8.2E-01 | 7.4E-01 | CLTC | 1 |
| R-HSA-196108 | Pregnenolone biosynthesis | 1/597 | 12/10867 | 4.9E-01 | 8.2E-01 | 7.4E-01 | STARD4 | 1 |
| R-HSA-209822 | Glycoprotein hormones | 1/597 | 12/10867 | 4.9E-01 | 8.2E-01 | 7.4E-01 | INHBA | 1 |
| R-HSA-211979 | Eicosanoids | 1/597 | 12/10867 | 4.9E-01 | 8.2E-01 | 7.4E-01 | CYP4F12 | 1 |
| R-HSA-264870 | Caspase-mediated cleavage of cytoskeletal proteins | 1/597 | 12/10867 | 4.9E-01 | 8.2E-01 | 7.4E-01 | DBNL | 1 |
| R-HSA-417957 | P2Y receptors | 1/597 | 12/10867 | 4.9E-01 | 8.2E-01 | 7.4E-01 | P2RY11 | 1 |
| R-HSA-6803211 | TP53 Regulates Transcription of Death Receptors and Ligands | 1/597 | 12/10867 | 4.9E-01 | 8.2E-01 | 7.4E-01 | TNFRSF10D | 1 |
| R-HSA-71064 | Lysine catabolism | 1/597 | 12/10867 | 4.9E-01 | 8.2E-01 | 7.4E-01 | PHYKPL | 1 |
| R-HSA-73817 | Purine ribonucleoside monophosphate biosynthesis | 1/597 | 12/10867 | 4.9E-01 | 8.2E-01 | 7.4E-01 | GMPS | 1 |
| R-HSA-8866427 | VLDLR internalisation and degradation | 1/597 | 12/10867 | 4.9E-01 | 8.2E-01 | 7.4E-01 | CLTC | 1 |
| R-HSA-9020558 | Interleukin-2 signaling | 1/597 | 12/10867 | 4.9E-01 | 8.2E-01 | 7.4E-01 | LCK | 1 |
| R-HSA-9664420 | Killing mechanisms | 1/597 | 12/10867 | 4.9E-01 | 8.2E-01 | 7.4E-01 | WNT5A | 1 |
| R-HSA-9673324 | WNT5:FZD7-mediated leishmania damping | 1/597 | 12/10867 | 4.9E-01 | 8.2E-01 | 7.4E-01 | WNT5A | 1 |
| R-HSA-6782135 | Dual incision in TC-NER | 4/597 | 66/10867 | 4.9E-01 | 8.2E-01 | 7.4E-01 | ERCC1/PCNA/RFC5/XAB2 | 4 |
| R-HSA-110314 | Recognition of DNA damage by PCNA-containing replication complex | 2/597 | 30/10867 | 5.0E-01 | 8.2E-01 | 7.4E-01 | PCNA/RFC5 | 2 |
| R-HSA-1482839 | Acyl chain remodelling of PE | 2/597 | 30/10867 | 5.0E-01 | 8.2E-01 | 7.4E-01 | PLA2G2A/PLA2G4A | 2 |
| R-HSA-3928663 | EPHA-mediated growth cone collapse | 2/597 | 30/10867 | 5.0E-01 | 8.2E-01 | 7.4E-01 | EPHA2/FYN | 2 |
| R-HSA-499943 | Interconversion of nucleotide di- and triphosphates | 2/597 | 30/10867 | 5.0E-01 | 8.2E-01 | 7.4E-01 | AK5/RRM2 | 2 |
| R-HSA-525793 | Myogenesis | 2/597 | 30/10867 | 5.0E-01 | 8.2E-01 | 7.4E-01 | CTNNB1/MEF2D | 2 |
| R-HSA-8866652 | Synthesis of active ubiquitin: roles of E1 and E2 enzymes | 2/597 | 30/10867 | 5.0E-01 | 8.2E-01 | 7.4E-01 | OTULIN/UBE2T | 2 |
| R-HSA-9674555 | Signaling by CSF3 (G-CSF) | 2/597 | 30/10867 | 5.0E-01 | 8.2E-01 | 7.4E-01 | HCK/TYK2 | 2 |
| R-HSA-9020702 | Interleukin-1 signaling | 6/597 | 103/10867 | 5.0E-01 | 8.2E-01 | 7.4E-01 | IL1RAP/MAP3K8/PSMD11/PSMD7/PSMD8/TNIP2 | 6 |
| R-HSA-5632684 | Hedgehog 'on' state | 5/597 | 85/10867 | 5.0E-01 | 8.2E-01 | 7.4E-01 | CUL3/DZIP1/PSMD11/PSMD7/PSMD8 | 5 |
| R-HSA-1236978 | Cross-presentation of soluble exogenous antigens (endosomes) | 3/597 | 49/10867 | 5.1E-01 | 8.2E-01 | 7.4E-01 | PSMD11/PSMD7/PSMD8 | 3 |
| R-HSA-157858 | Gap junction trafficking and regulation | 3/597 | 49/10867 | 5.1E-01 | 8.2E-01 | 7.4E-01 | CLTC/TJP1/TUBA1C | 3 |
| R-HSA-168333 | NEP/NS2 Interacts with the Cellular Export Machinery | 2/597 | 31/10867 | 5.1E-01 | 8.2E-01 | 7.4E-01 | NUP160/RAE1 | 2 |
| R-HSA-170822 | Regulation of Glucokinase by Glucokinase Regulatory Protein | 2/597 | 31/10867 | 5.1E-01 | 8.2E-01 | 7.4E-01 | NUP160/RAE1 | 2 |
| R-HSA-2022928 | HS-GAG biosynthesis | 2/597 | 31/10867 | 5.1E-01 | 8.2E-01 | 7.4E-01 | HS3ST3A1/SDC1 | 2 |
| R-HSA-445095 | Interaction between L1 and Ankyrins | 2/597 | 31/10867 | 5.1E-01 | 8.2E-01 | 7.4E-01 | SCN1B/SCN2A | 2 |
| R-HSA-450282 | MAPK targets/ Nuclear events mediated by MAP kinases | 2/597 | 31/10867 | 5.1E-01 | 8.2E-01 | 7.4E-01 | DUSP6/VRK3 | 2 |
| R-HSA-5619107 | Defective TPR may confer susceptibility towards thyroid papillary carcinoma (TPC) | 2/597 | 31/10867 | 5.1E-01 | 8.2E-01 | 7.4E-01 | NUP160/RAE1 | 2 |
| R-HSA-9668328 | Sealing of the nuclear envelope (NE) by ESCRT-III | 2/597 | 31/10867 | 5.1E-01 | 8.2E-01 | 7.4E-01 | CHMP3/TUBA1C | 2 |
| R-HSA-2029480 | Fcgamma receptor (FCGR) dependent phagocytosis | 5/597 | 86/10867 | 5.1E-01 | 8.2E-01 | 7.4E-01 | FYN/HCK/HSP90AA1/PIK3R1/WASF1 | 5 |
| R-HSA-6794362 | Protein-protein interactions at synapses | 5/597 | 86/10867 | 5.1E-01 | 8.2E-01 | 7.4E-01 | DBNL/IL1RAP/NLGN1/NLGN2/PTPRD | 5 |
| R-HSA-912446 | Meiotic recombination | 5/597 | 86/10867 | 5.1E-01 | 8.2E-01 | 7.4E-01 | BLM/H2AX/H2BC13/H2BC5/PSMC3IP | 5 |
| R-HSA-174154 | APC/C:Cdc20 mediated degradation of Securin | 4/597 | 68/10867 | 5.2E-01 | 8.2E-01 | 7.4E-01 | ANAPC2/PSMD11/PSMD7/PSMD8 | 4 |
| R-HSA-204005 | COPII-mediated vesicle transport | 4/597 | 68/10867 | 5.2E-01 | 8.2E-01 | 7.4E-01 | CD59/LMAN2/MCFD2/SEC23A | 4 |
| R-HSA-9609646 | HCMV Infection | 9/597 | 160/10867 | 5.2E-01 | 8.2E-01 | 7.4E-01 | CHMP3/EZH2/H2BC13/H2BC5/ITGB1/NUP160/RAE1/TUBA1C/VPS37B | 9 |
| R-HSA-1358803 | Downregulation of ERBB2:ERBB3 signaling | 1/597 | 13/10867 | 5.2E-01 | 8.2E-01 | 7.4E-01 | ERBB3 | 1 |
| R-HSA-1362409 | Mitochondrial iron-sulfur cluster biogenesis | 1/597 | 13/10867 | 5.2E-01 | 8.2E-01 | 7.4E-01 | ISCU | 1 |
| R-HSA-140342 | Apoptosis induced DNA fragmentation | 1/597 | 13/10867 | 5.2E-01 | 8.2E-01 | 7.4E-01 | KPNA1 | 1 |
| R-HSA-1663150 | The activation of arylsulfatases | 1/597 | 13/10867 | 5.2E-01 | 8.2E-01 | 7.4E-01 | ARSB | 1 |
| R-HSA-1679131 | Trafficking and processing of endosomal TLR | 1/597 | 13/10867 | 5.2E-01 | 8.2E-01 | 7.4E-01 | HSP90B1 | 1 |
| R-HSA-179812 | GRB2 events in EGFR signaling | 1/597 | 13/10867 | 5.2E-01 | 8.2E-01 | 7.4E-01 | NRAS | 1 |
| R-HSA-209543 | p75NTR recruits signalling complexes | 1/597 | 13/10867 | 5.2E-01 | 8.2E-01 | 7.4E-01 | PRKCI | 1 |
| R-HSA-3270619 | IRF3-mediated induction of type I IFN | 1/597 | 13/10867 | 5.2E-01 | 8.2E-01 | 7.4E-01 | IFI16 | 1 |
| R-HSA-5357786 | TNFR1-induced proapoptotic signaling | 1/597 | 13/10867 | 5.2E-01 | 8.2E-01 | 7.4E-01 | TNFAIP3 | 1 |
| R-HSA-74217 | Purine salvage | 1/597 | 13/10867 | 5.2E-01 | 8.2E-01 | 7.4E-01 | HPRT1 | 1 |
| R-HSA-75035 | Chk1/Chk2(Cds1) mediated inactivation of Cyclin B:Cdk1 complex | 1/597 | 13/10867 | 5.2E-01 | 8.2E-01 | 7.4E-01 | YWHAG | 1 |
| R-HSA-879415 | Advanced glycosylation endproduct receptor signaling | 1/597 | 13/10867 | 5.2E-01 | 8.2E-01 | 7.4E-01 | PRKCSH | 1 |
| R-HSA-8847993 | ERBB2 Activates PTK6 Signaling | 1/597 | 13/10867 | 5.2E-01 | 8.2E-01 | 7.4E-01 | ERBB3 | 1 |
| R-HSA-9637687 | Suppression of phagosomal maturation | 1/597 | 13/10867 | 5.2E-01 | 8.2E-01 | 7.4E-01 | KPNA1 | 1 |
| R-HSA-1236975 | Antigen processing-Cross presentation | 6/597 | 105/10867 | 5.2E-01 | 8.2E-01 | 7.4E-01 | CALR/HLA-A/PDIA3/PSMD11/PSMD7/PSMD8 | 6 |
| R-HSA-211733 | Regulation of activated PAK-2p34 by proteasome mediated degradation | 3/597 | 50/10867 | 5.2E-01 | 8.2E-01 | 7.4E-01 | PSMD11/PSMD7/PSMD8 | 3 |
| R-HSA-373752 | Netrin-1 signaling | 3/597 | 50/10867 | 5.2E-01 | 8.2E-01 | 7.4E-01 | FYN/NTN4/SLIT2 | 3 |
| R-HSA-5368286 | Mitochondrial translation initiation | 5/597 | 87/10867 | 5.2E-01 | 8.2E-01 | 7.4E-01 | MRPL12/MRPL15/MRPL17/MRPL58/MRPS2 | 5 |
| R-HSA-5389840 | Mitochondrial translation elongation | 5/597 | 87/10867 | 5.2E-01 | 8.2E-01 | 7.4E-01 | MRPL12/MRPL15/MRPL17/MRPL58/MRPS2 | 5 |
| R-HSA-5419276 | Mitochondrial translation termination | 5/597 | 87/10867 | 5.2E-01 | 8.2E-01 | 7.4E-01 | MRPL12/MRPL15/MRPL17/MRPL58/MRPS2 | 5 |
| R-HSA-9707564 | Cytoprotection by HMOX1 | 7/597 | 124/10867 | 5.3E-01 | 8.2E-01 | 7.4E-01 | CUL3/KEAP1/PSMD11/PSMD7/PSMD8/SKP2/TXNIP | 7 |
| R-HSA-388841 | Costimulation by the CD28 family | 4/597 | 69/10867 | 5.3E-01 | 8.2E-01 | 7.4E-01 | FYN/LCK/MAP3K8/PIK3R1 | 4 |
| R-HSA-5693571 | Nonhomologous End-Joining (NHEJ) | 4/597 | 69/10867 | 5.3E-01 | 8.2E-01 | 7.4E-01 | H2AX/H2BC13/H2BC5/RNF168 | 4 |
| R-HSA-9662360 | Sensory processing of sound by inner hair cells of the cochlea | 4/597 | 69/10867 | 5.3E-01 | 8.2E-01 | 7.4E-01 | EPS8L2/MSN/PJVK/TRIOBP | 4 |
| R-HSA-5250913 | Positive epigenetic regulation of rRNA expression | 6/597 | 106/10867 | 5.3E-01 | 8.2E-01 | 7.4E-01 | DDX21/GSK3B/H2AX/H2BC13/H2BC5/MBD3 | 6 |
| R-HSA-1474165 | Reproduction | 8/597 | 143/10867 | 5.3E-01 | 8.2E-01 | 7.4E-01 | BLM/H2AX/H2BC13/H2BC5/HVCN1/LMNB1/PSMC3IP/SMC1A | 8 |
| R-HSA-1251985 | Nuclear signaling by ERBB4 | 2/597 | 32/10867 | 5.3E-01 | 8.2E-01 | 7.4E-01 | MXD4/SPARC | 2 |
| R-HSA-2173789 | TGF-beta receptor signaling activates SMADs | 2/597 | 32/10867 | 5.3E-01 | 8.2E-01 | 7.4E-01 | PPP1CA/PPP1CB | 2 |
| R-HSA-2173796 | SMAD2/SMAD3:SMAD4 heterotrimer regulates transcription | 2/597 | 32/10867 | 5.3E-01 | 8.2E-01 | 7.4E-01 | JUNB/WWTR1 | 2 |
| R-HSA-397795 | G-protein beta:gamma signalling | 2/597 | 32/10867 | 5.3E-01 | 8.2E-01 | 7.4E-01 | GNB4/GNG12 | 2 |
| R-HSA-451326 | Activation of kainate receptors upon glutamate binding | 2/597 | 32/10867 | 5.3E-01 | 8.2E-01 | 7.4E-01 | GNB4/GNG12 | 2 |
| R-HSA-456926 | Thrombin signalling through proteinase activated receptors (PARs) | 2/597 | 32/10867 | 5.3E-01 | 8.2E-01 | 7.4E-01 | GNB4/GNG12 | 2 |
| R-HSA-5656169 | Termination of translesion DNA synthesis | 2/597 | 32/10867 | 5.3E-01 | 8.2E-01 | 7.4E-01 | PCNA/RFC5 | 2 |
| R-HSA-983695 | Antigen activates B Cell Receptor (BCR) leading to generation of second messengers | 2/597 | 32/10867 | 5.3E-01 | 8.2E-01 | 7.4E-01 | FYN/PIK3R1 | 2 |
| R-HSA-2559580 | Oxidative Stress Induced Senescence | 7/597 | 125/10867 | 5.3E-01 | 8.2E-01 | 7.4E-01 | CBX2/EZH2/H2AX/H2BC13/H2BC5/MAP4K4/RING1 | 7 |
| R-HSA-112043 | PLC beta mediated events | 3/597 | 51/10867 | 5.4E-01 | 8.2E-01 | 7.4E-01 | KPNA2/PLA2G4A/PRKCA | 3 |
| R-HSA-112310 | Neurotransmitter release cycle | 3/597 | 51/10867 | 5.4E-01 | 8.2E-01 | 7.4E-01 | ARL6IP5/MAOA/SYN2 | 3 |
| R-HSA-6811436 | COPI-independent Golgi-to-ER retrograde traffic | 3/597 | 51/10867 | 5.4E-01 | 8.2E-01 | 7.4E-01 | GALNT1/PLA2G4A/TUBA1C | 3 |
| R-HSA-72695 | Formation of the ternary complex. and subsequently. the 43S complex | 3/597 | 51/10867 | 5.4E-01 | 8.2E-01 | 7.4E-01 | EIF2S1/RPS14/RPS9 | 3 |
| R-HSA-376176 | Signaling by ROBO receptors | 12/597 | 218/10867 | 5.4E-01 | 8.2E-01 | 7.4E-01 | ENAH/PRKCA/PSMD11/PSMD7/PSMD8/RPL10/RPL28/RPL31/RPS14/RPS9/SLIT2/SRGAP2 | 12 |
| R-HSA-174824 | Plasma lipoprotein assembly. remodeling. and clearance | 4/597 | 70/10867 | 5.4E-01 | 8.2E-01 | 7.4E-01 | CIDEC/CLTC/LIPG/PCSK5 | 4 |
| R-HSA-380259 | Loss of Nlp from mitotic centrosomes | 4/597 | 70/10867 | 5.4E-01 | 8.2E-01 | 7.4E-01 | HSP90AA1/MAPRE1/TUBG1/YWHAG | 4 |
| R-HSA-380284 | Loss of proteins required for interphase microtubule organization from the centrosome | 4/597 | 70/10867 | 5.4E-01 | 8.2E-01 | 7.4E-01 | HSP90AA1/MAPRE1/TUBG1/YWHAG | 4 |
| R-HSA-156902 | Peptide chain elongation | 5/597 | 89/10867 | 5.5E-01 | 8.2E-01 | 7.4E-01 | RPL10/RPL28/RPL31/RPS14/RPS9 | 5 |
| R-HSA-192823 | Viral mRNA Translation | 5/597 | 89/10867 | 5.5E-01 | 8.2E-01 | 7.4E-01 | RPL10/RPL28/RPL31/RPS14/RPS9 | 5 |
| R-HSA-1250347 | SHC1 events in ERBB4 signaling | 1/597 | 14/10867 | 5.5E-01 | 8.2E-01 | 7.4E-01 | NRAS | 1 |
| R-HSA-162658 | Golgi Cisternae Pericentriolar Stack Reorganization | 1/597 | 14/10867 | 5.5E-01 | 8.2E-01 | 7.4E-01 | CCNB2 | 1 |
| R-HSA-169893 | Prolonged ERK activation events | 1/597 | 14/10867 | 5.5E-01 | 8.2E-01 | 7.4E-01 | RAPGEF1 | 1 |
| R-HSA-174490 | Membrane binding and targetting of GAG proteins | 1/597 | 14/10867 | 5.5E-01 | 8.2E-01 | 7.4E-01 | VPS37B | 1 |
| R-HSA-174495 | Synthesis And Processing Of GAG. GAGPOL Polyproteins | 1/597 | 14/10867 | 5.5E-01 | 8.2E-01 | 7.4E-01 | VPS37B | 1 |
| R-HSA-177504 | Retrograde neurotrophin signalling | 1/597 | 14/10867 | 5.5E-01 | 8.2E-01 | 7.4E-01 | CLTC | 1 |
| R-HSA-180336 | SHC1 events in EGFR signaling | 1/597 | 14/10867 | 5.5E-01 | 8.2E-01 | 7.4E-01 | NRAS | 1 |
| R-HSA-1855183 | Synthesis of IP2. IP. and Ins in the cytosol | 1/597 | 14/10867 | 5.5E-01 | 8.2E-01 | 7.4E-01 | INPP1 | 1 |
| R-HSA-193775 | Synthesis of bile acids and bile salts via 24-hydroxycholesterol | 1/597 | 14/10867 | 5.5E-01 | 8.2E-01 | 7.4E-01 | AKR1C3 | 1 |
| R-HSA-209952 | Peptide hormone biosynthesis | 1/597 | 14/10867 | 5.5E-01 | 8.2E-01 | 7.4E-01 | INHBA | 1 |
| R-HSA-2453864 | Retinoid cycle disease events | 1/597 | 14/10867 | 5.5E-01 | 8.2E-01 | 7.4E-01 | NAPEPLD | 1 |
| R-HSA-2474795 | Diseases associated with visual transduction | 1/597 | 14/10867 | 5.5E-01 | 8.2E-01 | 7.4E-01 | NAPEPLD | 1 |
| R-HSA-3296469 | Defects in cobalamin (B12) metabolism | 1/597 | 14/10867 | 5.5E-01 | 8.2E-01 | 7.4E-01 | ABCD4 | 1 |
| R-HSA-3656237 | Defective EXT2 causes exostoses 2 | 1/597 | 14/10867 | 5.5E-01 | 8.2E-01 | 7.4E-01 | SDC1 | 1 |
| R-HSA-3656253 | Defective EXT1 causes exostoses 1. TRPS2 and CHDS | 1/597 | 14/10867 | 5.5E-01 | 8.2E-01 | 7.4E-01 | SDC1 | 1 |
| R-HSA-399955 | SEMA3A-Plexin repulsion signaling by inhibiting Integrin adhesion | 1/597 | 14/10867 | 5.5E-01 | 8.2E-01 | 7.4E-01 | FYN | 1 |
| R-HSA-418885 | DCC mediated attractive signaling | 1/597 | 14/10867 | 5.5E-01 | 8.2E-01 | 7.4E-01 | FYN | 1 |
| R-HSA-428540 | Activation of RAC1 | 1/597 | 14/10867 | 5.5E-01 | 8.2E-01 | 7.4E-01 | SLIT2 | 1 |
| R-HSA-8849471 | PTK6 Regulates RHO GTPases. RAS GTPase and MAP kinases | 1/597 | 14/10867 | 5.5E-01 | 8.2E-01 | 7.4E-01 | NRAS | 1 |
| R-HSA-8941856 | RUNX3 regulates NOTCH signaling | 1/597 | 14/10867 | 5.5E-01 | 8.2E-01 | 7.4E-01 | MAML2 | 1 |
| R-HSA-8983432 | Interleukin-15 signaling | 1/597 | 14/10867 | 5.5E-01 | 8.2E-01 | 7.4E-01 | IL15RA | 1 |
| R-HSA-9675143 | Diseases of the neuronal system | 1/597 | 14/10867 | 5.5E-01 | 8.2E-01 | 7.4E-01 | NAPEPLD | 1 |
| R-HSA-9679504 | Translation of Replicase and Assembly of the Replication Transcription Complex | 1/597 | 14/10867 | 5.5E-01 | 8.2E-01 | 7.4E-01 | CHMP3 | 1 |
| R-HSA-9694676 | Translation of Replicase and Assembly of the Replication Transcription Complex | 1/597 | 14/10867 | 5.5E-01 | 8.2E-01 | 7.4E-01 | CHMP3 | 1 |
| R-HSA-163359 | Glucagon signaling in metabolic regulation | 2/597 | 33/10867 | 5.5E-01 | 8.2E-01 | 7.4E-01 | GNB4/GNG12 | 2 |
| R-HSA-168274 | Export of Viral Ribonucleoproteins from Nucleus | 2/597 | 33/10867 | 5.5E-01 | 8.2E-01 | 7.4E-01 | NUP160/RAE1 | 2 |
| R-HSA-180746 | Nuclear import of Rev protein | 2/597 | 33/10867 | 5.5E-01 | 8.2E-01 | 7.4E-01 | NUP160/RAE1 | 2 |
| R-HSA-418990 | Adherens junctions interactions | 2/597 | 33/10867 | 5.5E-01 | 8.2E-01 | 7.4E-01 | CDH17/CTNNB1 | 2 |
| R-HSA-420092 | Glucagon-type ligand receptors | 2/597 | 33/10867 | 5.5E-01 | 8.2E-01 | 7.4E-01 | GNB4/GNG12 | 2 |
| R-HSA-5654726 | Negative regulation of FGFR1 signaling | 2/597 | 33/10867 | 5.5E-01 | 8.2E-01 | 7.4E-01 | FGF1/FGF2 | 2 |
| R-HSA-163685 | Integration of energy metabolism | 6/597 | 108/10867 | 5.5E-01 | 8.2E-01 | 7.4E-01 | ACSL4/ADRA2A/GNB4/GNG12/IQGAP1/PRKCA | 6 |
| R-HSA-171306 | Packaging Of Telomere Ends | 3/597 | 52/10867 | 5.5E-01 | 8.2E-01 | 7.4E-01 | H2AX/H2BC13/H2BC5 | 3 |
| R-HSA-180534 | Vpu mediated degradation of CD4 | 3/597 | 52/10867 | 5.5E-01 | 8.2E-01 | 7.4E-01 | PSMD11/PSMD7/PSMD8 | 3 |
| R-HSA-349425 | Autodegradation of the E3 ubiquitin ligase COP1 | 3/597 | 52/10867 | 5.5E-01 | 8.2E-01 | 7.4E-01 | PSMD11/PSMD7/PSMD8 | 3 |
| R-HSA-445717 | Aquaporin-mediated transport | 3/597 | 52/10867 | 5.5E-01 | 8.2E-01 | 7.4E-01 | GNB4/GNG12/RAB11FIP2 | 3 |
| R-HSA-4551638 | SUMOylation of chromatin organization proteins | 4/597 | 71/10867 | 5.5E-01 | 8.2E-01 | 7.4E-01 | CBX2/NUP160/RAE1/RING1 | 4 |
| R-HSA-5689880 | Ub-specific processing proteases | 12/597 | 220/10867 | 5.5E-01 | 8.2E-01 | 7.4E-01 | CCNA2/CDC25A/FKBP8/H2BC13/H2BC5/KEAP1/PSMD11/PSMD7/PSMD8/PTEN/SKP2/USP37 | 12 |
| R-HSA-8878171 | Transcriptional regulation by RUNX1 | 13/597 | 239/10867 | 5.6E-01 | 8.2E-01 | 7.4E-01 | CBX2/H2AX/H2BC13/H2BC5/KMT2E/LIFR/PSMD11/PSMD7/PSMD8/RING1/RUNX1/SMARCC1/TJP1 | 13 |
| R-HSA-6811442 | Intra-Golgi and retrograde Golgi-to-ER traffic | 11/597 | 202/10867 | 5.6E-01 | 8.2E-01 | 7.4E-01 | COPG1/CYTH2/GALNT1/GOLIM4/KIF18B/KIF20A/KIF26B/PLA2G4A/SURF4/TUBA1C/VPS51 | 11 |
| R-HSA-8980692 | RHOA GTPase cycle | 8/597 | 147/10867 | 5.6E-01 | 8.2E-01 | 7.4E-01 | ARHGAP20/ARHGEF12/ARHGEF3/CAV1/DAAM1/IQGAP1/PCDH7/PIK3R1 | 8 |
| R-HSA-1660499 | Synthesis of PIPs at the plasma membrane | 3/597 | 53/10867 | 5.6E-01 | 8.2E-01 | 7.4E-01 | PIK3R1/PIP4K2C/PTEN | 3 |
| R-HSA-169911 | Regulation of Apoptosis | 3/597 | 53/10867 | 5.6E-01 | 8.2E-01 | 7.4E-01 | PSMD11/PSMD7/PSMD8 | 3 |
| R-HSA-1445148 | Translocation of SLC2A4 (GLUT4) to the plasma membrane | 4/597 | 72/10867 | 5.6E-01 | 8.2E-01 | 7.4E-01 | PRKAG1/RALA/TUBA1C/YWHAG | 4 |
| R-HSA-448424 | Interleukin-17 signaling | 4/597 | 72/10867 | 5.6E-01 | 8.2E-01 | 7.4E-01 | DUSP6/MAP3K8/TNIP2/VRK3 | 4 |
| R-HSA-159227 | Transport of the SLBP independent Mature mRNA | 2/597 | 34/10867 | 5.6E-01 | 8.2E-01 | 7.4E-01 | NUP160/RAE1 | 2 |
| R-HSA-165054 | Rev-mediated nuclear export of HIV RNA | 2/597 | 34/10867 | 5.6E-01 | 8.2E-01 | 7.4E-01 | NUP160/RAE1 | 2 |
| R-HSA-202433 | Generation of second messenger molecules | 2/597 | 34/10867 | 5.6E-01 | 8.2E-01 | 7.4E-01 | ENAH/LCK | 2 |
| R-HSA-4085377 | SUMOylation of SUMOylation proteins | 2/597 | 34/10867 | 5.6E-01 | 8.2E-01 | 7.4E-01 | NUP160/RAE1 | 2 |
| R-HSA-5576892 | Phase 0 - rapid depolarisation | 2/597 | 34/10867 | 5.6E-01 | 8.2E-01 | 7.4E-01 | SCN1B/SCN2A | 2 |
| R-HSA-5579029 | Metabolic disorders of biological oxidation enzymes | 2/597 | 34/10867 | 5.6E-01 | 8.2E-01 | 7.4E-01 | MAOA/SLC35D1 | 2 |
| R-HSA-5663084 | Diseases of carbohydrate metabolism | 2/597 | 34/10867 | 5.6E-01 | 8.2E-01 | 7.4E-01 | ARSB/HYAL1 | 2 |
| R-HSA-70263 | Gluconeogenesis | 2/597 | 34/10867 | 5.6E-01 | 8.2E-01 | 7.4E-01 | SLC25A12/SLC25A13 | 2 |
| R-HSA-201722 | Formation of the beta-catenin:TCF transactivating complex | 5/597 | 91/10867 | 5.7E-01 | 8.2E-01 | 7.4E-01 | CTNNB1/H2AX/H2BC13/H2BC5/TLE4 | 5 |
| R-HSA-2980736 | Peptide hormone metabolism | 5/597 | 91/10867 | 5.7E-01 | 8.2E-01 | 7.4E-01 | BCHE/CTNNB1/IGF1/INHBA/SLC30A7 | 5 |
| R-HSA-5250924 | B-WICH complex positively regulates rRNA expression | 5/597 | 91/10867 | 5.7E-01 | 8.2E-01 | 7.4E-01 | DDX21/GSK3B/H2AX/H2BC13/H2BC5 | 5 |
| R-HSA-70326 | Glucose metabolism | 5/597 | 91/10867 | 5.7E-01 | 8.2E-01 | 7.4E-01 | NUP160/PFKL/RAE1/SLC25A12/SLC25A13 | 5 |
| R-HSA-5576891 | Cardiac conduction | 7/597 | 129/10867 | 5.7E-01 | 8.2E-01 | 7.4E-01 | ATP1A2/DMPK/KCNK10/NPR2/SCN1B/SCN2A/WWTR1 | 7 |
| R-HSA-111447 | Activation of BAD and translocation to mitochondria | 1/597 | 15/10867 | 5.7E-01 | 8.2E-01 | 7.4E-01 | YWHAG | 1 |
| R-HSA-1170546 | Prolactin receptor signaling | 1/597 | 15/10867 | 5.7E-01 | 8.2E-01 | 7.4E-01 | SH2B1 | 1 |
| R-HSA-193807 | Synthesis of bile acids and bile salts via 27-hydroxycholesterol | 1/597 | 15/10867 | 5.7E-01 | 8.2E-01 | 7.4E-01 | AKR1C3 | 1 |
| R-HSA-211935 | Fatty acids | 1/597 | 15/10867 | 5.7E-01 | 8.2E-01 | 7.4E-01 | CYP4F12 | 1 |
| R-HSA-354194 | GRB2:SOS provides linkage to MAPK signaling for Integrins | 1/597 | 15/10867 | 5.7E-01 | 8.2E-01 | 7.4E-01 | ITGB3 | 1 |
| R-HSA-372708 | p130Cas linkage to MAPK signaling for integrins | 1/597 | 15/10867 | 5.7E-01 | 8.2E-01 | 7.4E-01 | ITGB3 | 1 |
| R-HSA-549132 | Organic cation/anion/zwitterion transport | 1/597 | 15/10867 | 5.7E-01 | 8.2E-01 | 7.4E-01 | RUNX1 | 1 |
| R-HSA-6785631 | ERBB2 Regulates Cell Motility | 1/597 | 15/10867 | 5.7E-01 | 8.2E-01 | 7.4E-01 | ERBB3 | 1 |
| R-HSA-8875360 | InlB-mediated entry of Listeria monocytogenes into host cell | 1/597 | 15/10867 | 5.7E-01 | 8.2E-01 | 7.4E-01 | MET | 1 |
| R-HSA-8956320 | Nucleobase biosynthesis | 1/597 | 15/10867 | 5.7E-01 | 8.2E-01 | 7.4E-01 | GMPS | 1 |
| R-HSA-975577 | N-Glycan antennae elongation | 1/597 | 15/10867 | 5.7E-01 | 8.2E-01 | 7.4E-01 | B4GALT5 | 1 |
| R-HSA-170834 | Signaling by TGF-beta Receptor Complex | 4/597 | 73/10867 | 5.7E-01 | 8.2E-01 | 7.4E-01 | JUNB/PPP1CA/PPP1CB/WWTR1 | 4 |
| R-HSA-2682334 | EPH-Ephrin signaling | 5/597 | 92/10867 | 5.7E-01 | 8.2E-01 | 7.4E-01 | CLTC/EFNB2/EPHA2/EPHB6/FYN | 5 |
| R-HSA-156827 | L13a-mediated translational silencing of Ceruloplasmin expression | 6/597 | 111/10867 | 5.8E-01 | 8.2E-01 | 7.4E-01 | EIF2S1/RPL10/RPL28/RPL31/RPS14/RPS9 | 6 |
| R-HSA-180585 | Vif-mediated degradation of APOBEC3G | 3/597 | 54/10867 | 5.8E-01 | 8.2E-01 | 7.4E-01 | PSMD11/PSMD7/PSMD8 | 3 |
| R-HSA-8939236 | RUNX1 regulates transcription of genes involved in differentiation of HSCs | 7/597 | 130/10867 | 5.8E-01 | 8.2E-01 | 7.4E-01 | H2AX/H2BC13/H2BC5/PSMD11/PSMD7/PSMD8/RUNX1 | 7 |
| R-HSA-1296065 | Inwardly rectifying K+ channels | 2/597 | 35/10867 | 5.8E-01 | 8.2E-01 | 7.4E-01 | GNB4/GNG12 | 2 |
| R-HSA-159230 | Transport of the SLBP Dependant Mature mRNA | 2/597 | 35/10867 | 5.8E-01 | 8.2E-01 | 7.4E-01 | NUP160/RAE1 | 2 |
| R-HSA-196071 | Metabolism of steroid hormones | 2/597 | 35/10867 | 5.8E-01 | 8.2E-01 | 7.4E-01 | SRD5A1/STARD4 | 2 |
| R-HSA-432720 | Lysosome Vesicle Biogenesis | 2/597 | 35/10867 | 5.8E-01 | 8.2E-01 | 7.4E-01 | CLTC/VAMP7 | 2 |
| R-HSA-5357905 | Regulation of TNFR1 signaling | 2/597 | 35/10867 | 5.8E-01 | 8.2E-01 | 7.4E-01 | OTULIN/TNFAIP3 | 2 |
| R-HSA-9615710 | Late endosomal microautophagy | 2/597 | 35/10867 | 5.8E-01 | 8.2E-01 | 7.4E-01 | CHMP3/VPS37B | 2 |
| R-HSA-1799339 | SRP-dependent cotranslational protein targeting to membrane | 6/597 | 112/10867 | 5.8E-01 | 8.2E-01 | 7.4E-01 | RPL10/RPL28/RPL31/RPN2/RPS14/RPS9 | 6 |
| R-HSA-2559582 | Senescence-Associated Secretory Phenotype (SASP) | 6/597 | 112/10867 | 5.8E-01 | 8.2E-01 | 7.4E-01 | ANAPC2/CCNA2/H2AX/H2BC13/H2BC5/IGFBP7 | 6 |
| R-HSA-72706 | GTP hydrolysis and joining of the 60S ribosomal subunit | 6/597 | 112/10867 | 5.8E-01 | 8.2E-01 | 7.4E-01 | EIF2S1/RPL10/RPL28/RPL31/RPS14/RPS9 | 6 |
| R-HSA-983705 | Signaling by the B Cell Receptor (BCR) | 6/597 | 112/10867 | 5.8E-01 | 8.2E-01 | 7.4E-01 | FYN/NRAS/PIK3R1/PSMD11/PSMD7/PSMD8 | 6 |
| R-HSA-156842 | Eukaryotic Translation Elongation | 5/597 | 93/10867 | 5.8E-01 | 8.2E-01 | 7.4E-01 | RPL10/RPL28/RPL31/RPS14/RPS9 | 5 |
| R-HSA-2408557 | Selenocysteine synthesis | 5/597 | 93/10867 | 5.8E-01 | 8.2E-01 | 7.4E-01 | RPL10/RPL28/RPL31/RPS14/RPS9 | 5 |
| R-HSA-5368287 | Mitochondrial translation | 5/597 | 93/10867 | 5.8E-01 | 8.2E-01 | 7.4E-01 | MRPL12/MRPL15/MRPL17/MRPL58/MRPS2 | 5 |
| R-HSA-72764 | Eukaryotic Translation Termination | 5/597 | 93/10867 | 5.8E-01 | 8.2E-01 | 7.4E-01 | RPL10/RPL28/RPL31/RPS14/RPS9 | 5 |
| R-HSA-2299718 | Condensation of Prophase Chromosomes | 4/597 | 74/10867 | 5.9E-01 | 8.2E-01 | 7.4E-01 | H2AX/H2BC13/H2BC5/SMC4 | 4 |
| R-HSA-174113 | SCF-beta-TrCP mediated degradation of Emi1 | 3/597 | 55/10867 | 5.9E-01 | 8.2E-01 | 7.4E-01 | PSMD11/PSMD7/PSMD8 | 3 |
| R-HSA-4641257 | Degradation of AXIN | 3/597 | 55/10867 | 5.9E-01 | 8.2E-01 | 7.4E-01 | PSMD11/PSMD7/PSMD8 | 3 |
| R-HSA-6794361 | Neurexins and neuroligins | 3/597 | 55/10867 | 5.9E-01 | 8.2E-01 | 7.4E-01 | DBNL/NLGN1/NLGN2 | 3 |
| R-HSA-8941858 | Regulation of RUNX3 expression and activity | 3/597 | 55/10867 | 5.9E-01 | 8.2E-01 | 7.4E-01 | PSMD11/PSMD7/PSMD8 | 3 |
| R-HSA-9013409 | RHOJ GTPase cycle | 3/597 | 55/10867 | 5.9E-01 | 8.2E-01 | 7.4E-01 | CAV1/CDC42EP1/PIK3R1 | 3 |
| R-HSA-9612973 | Autophagy | 8/597 | 151/10867 | 5.9E-01 | 8.2E-01 | 7.4E-01 | CHMP3/HSP90AA1/LAMTOR4/PRKAA1/PRKAG1/RRAGC/TUBA1C/VPS37B | 8 |
| R-HSA-381119 | Unfolded Protein Response (UPR) | 5/597 | 94/10867 | 5.9E-01 | 8.2E-01 | 7.4E-01 | CALR/DNAJB11/EIF2S1/GSK3A/HSP90B1 | 5 |
| R-HSA-175474 | Assembly Of The HIV Virion | 1/597 | 16/10867 | 6.0E-01 | 8.2E-01 | 7.4E-01 | VPS37B | 1 |
| R-HSA-193639 | p75NTR signals via NF-kB | 1/597 | 16/10867 | 6.0E-01 | 8.2E-01 | 7.4E-01 | PRKCI | 1 |
| R-HSA-1963640 | GRB2 events in ERBB2 signaling | 1/597 | 16/10867 | 6.0E-01 | 8.2E-01 | 7.4E-01 | NRAS | 1 |
| R-HSA-204174 | Regulation of pyruvate dehydrogenase (PDH) complex | 1/597 | 16/10867 | 6.0E-01 | 8.2E-01 | 7.4E-01 | PDHX | 1 |
| R-HSA-210744 | Regulation of gene expression in late stage (branching morphogenesis) pancreatic bud precursor cells | 1/597 | 16/10867 | 6.0E-01 | 8.2E-01 | 7.4E-01 | MAML2 | 1 |
| R-HSA-392517 | Rap1 signalling | 1/597 | 16/10867 | 6.0E-01 | 8.2E-01 | 7.4E-01 | PRKG1 | 1 |
| R-HSA-418038 | Nucleotide-like (purinergic) receptors | 1/597 | 16/10867 | 6.0E-01 | 8.2E-01 | 7.4E-01 | P2RY11 | 1 |
| R-HSA-418457 | cGMP effects | 1/597 | 16/10867 | 6.0E-01 | 8.2E-01 | 7.4E-01 | PRKG1 | 1 |
| R-HSA-429958 | mRNA decay by 3' to 5' exoribonuclease | 1/597 | 16/10867 | 6.0E-01 | 8.2E-01 | 7.4E-01 | SKIV2L | 1 |
| R-HSA-844456 | The NLRP3 inflammasome | 1/597 | 16/10867 | 6.0E-01 | 8.2E-01 | 7.4E-01 | TXNIP | 1 |
| R-HSA-8949664 | Processing of SMDT1 | 1/597 | 16/10867 | 6.0E-01 | 8.2E-01 | 7.4E-01 | MCU | 1 |
| R-HSA-177243 | Interactions of Rev with host cellular proteins | 2/597 | 36/10867 | 6.0E-01 | 8.2E-01 | 7.4E-01 | NUP160/RAE1 | 2 |
| R-HSA-390522 | Striated Muscle Contraction | 2/597 | 36/10867 | 6.0E-01 | 8.2E-01 | 7.4E-01 | MYBPC1/TNNC1 | 2 |
| R-HSA-8956319 | Nucleobase catabolism | 2/597 | 36/10867 | 6.0E-01 | 8.2E-01 | 7.4E-01 | ENTPD7/NUDT5 | 2 |
| R-HSA-110330 | Recognition and association of DNA glycosylase with site containing an affected purine | 3/597 | 56/10867 | 6.0E-01 | 8.2E-01 | 7.4E-01 | H2AX/H2BC13/H2BC5 | 3 |
| R-HSA-110331 | Cleavage of the damaged purine | 3/597 | 56/10867 | 6.0E-01 | 8.2E-01 | 7.4E-01 | H2AX/H2BC13/H2BC5 | 3 |
| R-HSA-112040 | G-protein mediated events | 3/597 | 56/10867 | 6.0E-01 | 8.2E-01 | 7.4E-01 | KPNA2/PLA2G4A/PRKCA | 3 |
| R-HSA-450408 | AUF1 (hnRNP D0) binds and destabilizes mRNA | 3/597 | 56/10867 | 6.0E-01 | 8.2E-01 | 7.4E-01 | PSMD11/PSMD7/PSMD8 | 3 |
| R-HSA-5362768 | Hh mutants are degraded by ERAD | 3/597 | 56/10867 | 6.0E-01 | 8.2E-01 | 7.4E-01 | PSMD11/PSMD7/PSMD8 | 3 |
| R-HSA-73927 | Depurination | 3/597 | 56/10867 | 6.0E-01 | 8.2E-01 | 7.4E-01 | H2AX/H2BC13/H2BC5 | 3 |
| R-HSA-9010553 | Regulation of expression of SLITs and ROBOs | 9/597 | 171/10867 | 6.0E-01 | 8.2E-01 | 7.4E-01 | PSMD11/PSMD7/PSMD8/RPL10/RPL28/RPL31/RPS14/RPS9/SLIT2 | 9 |
| R-HSA-1592230 | Mitochondrial biogenesis | 5/597 | 95/10867 | 6.0E-01 | 8.3E-01 | 7.4E-01 | ATP5F1B/ESRRA/IDH2/MEF2D/PRKAG1 | 5 |
| R-HSA-975956 | Nonsense Mediated Decay (NMD) independent of the Exon Junction Complex (EJC) | 5/597 | 95/10867 | 6.0E-01 | 8.3E-01 | 7.4E-01 | RPL10/RPL28/RPL31/RPS14/RPS9 | 5 |
| R-HSA-427389 | ERCC6 (CSB) and EHMT2 (G9a) positively regulate rRNA expression | 4/597 | 76/10867 | 6.1E-01 | 8.3E-01 | 7.4E-01 | H2AX/H2BC13/H2BC5/MBD3 | 4 |
| R-HSA-9659379 | Sensory processing of sound | 4/597 | 76/10867 | 6.1E-01 | 8.3E-01 | 7.4E-01 | EPS8L2/MSN/PJVK/TRIOBP | 4 |
| R-HSA-5357801 | Programmed Cell Death | 11/597 | 210/10867 | 6.1E-01 | 8.3E-01 | 7.4E-01 | CHMP3/CTNNB1/DBNL/HSP90AA1/KPNA1/LMNB1/PSMD11/PSMD7/PSMD8/TJP1/YWHAG | 11 |
| R-HSA-111933 | Calmodulin induced events | 2/597 | 37/10867 | 6.1E-01 | 8.3E-01 | 7.4E-01 | KPNA2/PRKCA | 2 |
| R-HSA-111997 | CaM pathway | 2/597 | 37/10867 | 6.1E-01 | 8.3E-01 | 7.4E-01 | KPNA2/PRKCA | 2 |
| R-HSA-5083635 | Defective B3GALTL causes Peters-plus syndrome (PpS) | 2/597 | 37/10867 | 6.1E-01 | 8.3E-01 | 7.4E-01 | ADAMTS2/THSD7A | 2 |
| R-HSA-75105 | Fatty acyl-CoA biosynthesis | 2/597 | 37/10867 | 6.1E-01 | 8.3E-01 | 7.4E-01 | ACSL4/ACSL5 | 2 |
| R-HSA-77289 | Mitochondrial Fatty Acid Beta-Oxidation | 2/597 | 37/10867 | 6.1E-01 | 8.3E-01 | 7.4E-01 | ACADL/ACADS | 2 |
| R-HSA-69541 | Stabilization of p53 | 3/597 | 57/10867 | 6.1E-01 | 8.3E-01 | 7.4E-01 | PSMD11/PSMD7/PSMD8 | 3 |
| R-HSA-936837 | Ion transport by P-type ATPases | 3/597 | 57/10867 | 6.1E-01 | 8.3E-01 | 7.4E-01 | ATP11B/ATP1A2/ATP7A | 3 |
| R-HSA-9609690 | HCMV Early Events | 7/597 | 135/10867 | 6.2E-01 | 8.3E-01 | 7.4E-01 | EZH2/H2BC13/H2BC5/ITGB1/NUP160/RAE1/TUBA1C | 7 |
| R-HSA-5693565 | Recruitment and ATM-mediated phosphorylation of repair and signaling proteins at DNA double strand breaks | 4/597 | 77/10867 | 6.2E-01 | 8.3E-01 | 7.4E-01 | H2AX/H2BC13/H2BC5/RNF168 | 4 |
| R-HSA-180292 | GAB1 signalosome | 1/597 | 17/10867 | 6.2E-01 | 8.3E-01 | 7.4E-01 | PIK3R1 | 1 |
| R-HSA-1810476 | RIP-mediated NFkB activation via ZBP1 | 1/597 | 17/10867 | 6.2E-01 | 8.3E-01 | 7.4E-01 | DHX9 | 1 |
| R-HSA-196757 | Metabolism of folate and pterines | 1/597 | 17/10867 | 6.2E-01 | 8.3E-01 | 7.4E-01 | ALDH1L1 | 1 |
| R-HSA-416993 | Trafficking of GluR2-containing AMPA receptors | 1/597 | 17/10867 | 6.2E-01 | 8.3E-01 | 7.4E-01 | PRKCA | 1 |
| R-HSA-432142 | Platelet sensitization by LDL | 1/597 | 17/10867 | 6.2E-01 | 8.3E-01 | 7.4E-01 | PLA2G4A | 1 |
| R-HSA-435354 | Zinc transporters | 1/597 | 17/10867 | 6.2E-01 | 8.3E-01 | 7.4E-01 | SLC30A7 | 1 |
| R-HSA-450513 | Tristetraprolin (TTP. ZFP36) binds and destabilizes mRNA | 1/597 | 17/10867 | 6.2E-01 | 8.3E-01 | 7.4E-01 | ZFP36 | 1 |
| R-HSA-8934593 | Regulation of RUNX1 Expression and Activity | 1/597 | 17/10867 | 6.2E-01 | 8.3E-01 | 7.4E-01 | RUNX1 | 1 |
| R-HSA-9022699 | MECP2 regulates neuronal receptors and channels | 1/597 | 17/10867 | 6.2E-01 | 8.3E-01 | 7.4E-01 | MET | 1 |
| R-HSA-9610379 | HCMV Late Events | 6/597 | 116/10867 | 6.2E-01 | 8.3E-01 | 7.4E-01 | CHMP3/H2BC13/H2BC5/NUP160/RAE1/VPS37B | 6 |
| R-HSA-8936459 | RUNX1 regulates genes involved in megakaryocyte differentiation and platelet function | 5/597 | 97/10867 | 6.2E-01 | 8.3E-01 | 7.4E-01 | H2AX/H2BC13/H2BC5/KMT2E/RUNX1 | 5 |
| R-HSA-1632852 | Macroautophagy | 7/597 | 136/10867 | 6.2E-01 | 8.3E-01 | 7.4E-01 | CHMP3/HSP90AA1/LAMTOR4/PRKAA1/PRKAG1/RRAGC/TUBA1C | 7 |
| R-HSA-72649 | Translation initiation complex formation | 3/597 | 58/10867 | 6.2E-01 | 8.3E-01 | 7.4E-01 | EIF2S1/RPS14/RPS9 | 3 |
| R-HSA-72702 | Ribosomal scanning and start codon recognition | 3/597 | 58/10867 | 6.2E-01 | 8.3E-01 | 7.4E-01 | EIF2S1/RPS14/RPS9 | 3 |
| R-HSA-3232142 | SUMOylation of ubiquitinylation proteins | 2/597 | 38/10867 | 6.3E-01 | 8.3E-01 | 7.4E-01 | NUP160/RAE1 | 2 |
| R-HSA-5173214 | O-glycosylation of TSR domain-containing proteins | 2/597 | 38/10867 | 6.3E-01 | 8.3E-01 | 7.4E-01 | ADAMTS2/THSD7A | 2 |
| R-HSA-8864260 | Transcriptional regulation by the AP-2 (TFAP2) family of transcription factors | 2/597 | 38/10867 | 6.3E-01 | 8.3E-01 | 7.4E-01 | KDM5B/MYBL2 | 2 |
| R-HSA-5619084 | ABC transporter disorders | 4/597 | 78/10867 | 6.3E-01 | 8.3E-01 | 7.4E-01 | ABCD4/PSMD11/PSMD7/PSMD8 | 4 |
| R-HSA-9711097 | Cellular response to starvation | 8/597 | 156/10867 | 6.3E-01 | 8.3E-01 | 7.4E-01 | EIF2S1/LAMTOR4/RPL10/RPL28/RPL31/RPS14/RPS9/RRAGC | 8 |
| R-HSA-5619102 | SLC transporter disorders | 5/597 | 98/10867 | 6.3E-01 | 8.3E-01 | 7.4E-01 | NUP160/RAE1/SLC17A5/SLC35A2/SLCO2A1 | 5 |
| R-HSA-2408522 | Selenoamino acid metabolism | 6/597 | 118/10867 | 6.4E-01 | 8.3E-01 | 7.4E-01 | MARS1/RPL10/RPL28/RPL31/RPS14/RPS9 | 6 |
| R-HSA-5619115 | Disorders of transmembrane transporters | 9/597 | 176/10867 | 6.4E-01 | 8.3E-01 | 7.4E-01 | ABCD4/NUP160/PSMD11/PSMD7/PSMD8/RAE1/SLC17A5/SLC35A2/SLCO2A1 | 9 |
| R-HSA-380108 | Chemokine receptors bind chemokines | 3/597 | 59/10867 | 6.4E-01 | 8.3E-01 | 7.4E-01 | CCL5/CXCL2/CXCL6 | 3 |
| R-HSA-5387390 | Hh mutants abrogate ligand secretion | 3/597 | 59/10867 | 6.4E-01 | 8.3E-01 | 7.4E-01 | PSMD11/PSMD7/PSMD8 | 3 |
| R-HSA-72662 | Activation of the mRNA upon binding of the cap-binding complex and eIFs. and subsequent binding to 43S | 3/597 | 59/10867 | 6.4E-01 | 8.3E-01 | 7.4E-01 | EIF2S1/RPS14/RPS9 | 3 |
| R-HSA-9664407 | Parasite infection | 3/597 | 59/10867 | 6.4E-01 | 8.3E-01 | 7.4E-01 | FYN/HCK/WASF1 | 3 |
| R-HSA-9664417 | Leishmania phagocytosis | 3/597 | 59/10867 | 6.4E-01 | 8.3E-01 | 7.4E-01 | FYN/HCK/WASF1 | 3 |
| R-HSA-9664422 | FCGR3A-mediated phagocytosis | 3/597 | 59/10867 | 6.4E-01 | 8.3E-01 | 7.4E-01 | FYN/HCK/WASF1 | 3 |
| R-HSA-6781827 | Transcription-Coupled Nucleotide Excision Repair (TC-NER) | 4/597 | 79/10867 | 6.4E-01 | 8.3E-01 | 7.4E-01 | ERCC1/PCNA/RFC5/XAB2 | 4 |
| R-HSA-1369062 | ABC transporters in lipid homeostasis | 1/597 | 18/10867 | 6.4E-01 | 8.3E-01 | 7.4E-01 | ABCA6 | 1 |
| R-HSA-163210 | Formation of ATP by chemiosmotic coupling | 1/597 | 18/10867 | 6.4E-01 | 8.3E-01 | 7.4E-01 | ATP5F1B | 1 |
| R-HSA-1660514 | Synthesis of PIPs at the Golgi membrane | 1/597 | 18/10867 | 6.4E-01 | 8.3E-01 | 7.4E-01 | INPP5E | 1 |
| R-HSA-181429 | Serotonin Neurotransmitter Release Cycle | 1/597 | 18/10867 | 6.4E-01 | 8.3E-01 | 7.4E-01 | SYN2 | 1 |
| R-HSA-181430 | Norepinephrine Neurotransmitter Release Cycle | 1/597 | 18/10867 | 6.4E-01 | 8.3E-01 | 7.4E-01 | MAOA | 1 |
| R-HSA-1839117 | Signaling by cytosolic FGFR1 fusion mutants | 1/597 | 18/10867 | 6.4E-01 | 8.3E-01 | 7.4E-01 | PIK3R1 | 1 |
| R-HSA-190840 | Microtubule-dependent trafficking of connexons from Golgi to the plasma membrane | 1/597 | 18/10867 | 6.4E-01 | 8.3E-01 | 7.4E-01 | TUBA1C | 1 |
| R-HSA-1912420 | Pre-NOTCH Processing in Golgi | 1/597 | 18/10867 | 6.4E-01 | 8.3E-01 | 7.4E-01 | NOTCH2 | 1 |
| R-HSA-209776 | Metabolism of amine-derived hormones | 1/597 | 18/10867 | 6.4E-01 | 8.3E-01 | 7.4E-01 | DIO2 | 1 |
| R-HSA-74259 | Purine catabolism | 1/597 | 18/10867 | 6.4E-01 | 8.3E-01 | 7.4E-01 | NUDT5 | 1 |
| R-HSA-9037629 | Lewis blood group biosynthesis | 1/597 | 18/10867 | 6.4E-01 | 8.3E-01 | 7.4E-01 | FUT11 | 1 |
| R-HSA-110313 | Translesion synthesis by Y family DNA polymerases bypasses lesions on DNA template | 2/597 | 39/10867 | 6.4E-01 | 8.3E-01 | 7.4E-01 | PCNA/RFC5 | 2 |
| R-HSA-8853884 | Transcriptional Regulation by VENTX | 2/597 | 39/10867 | 6.4E-01 | 8.3E-01 | 7.4E-01 | ANAPC2/CTNNB1 | 2 |
| R-HSA-211000 | Gene Silencing by RNA | 7/597 | 138/10867 | 6.4E-01 | 8.3E-01 | 7.4E-01 | H2AX/H2BC13/H2BC5/HSP90AA1/NUP160/RAE1/XPO5 | 7 |
| R-HSA-8978868 | Fatty acid metabolism | 9/597 | 177/10867 | 6.4E-01 | 8.3E-01 | 7.4E-01 | ACADL/ACADS/ACSL4/ACSL5/AKR1C3/EPHX2/PLA2G4A/PTGES2/PTGS2 | 9 |
| R-HSA-72613 | Eukaryotic Translation Initiation | 6/597 | 119/10867 | 6.4E-01 | 8.3E-01 | 7.4E-01 | EIF2S1/RPL10/RPL28/RPL31/RPS14/RPS9 | 6 |
| R-HSA-72737 | Cap-dependent Translation Initiation | 6/597 | 119/10867 | 6.4E-01 | 8.3E-01 | 7.4E-01 | EIF2S1/RPL10/RPL28/RPL31/RPS14/RPS9 | 6 |
| R-HSA-162599 | Late Phase of HIV Life Cycle | 7/597 | 139/10867 | 6.5E-01 | 8.3E-01 | 7.5E-01 | CHMP3/NELFB/NUP160/RAE1/TAF11/VPS37B/VTA1 | 7 |
| R-HSA-5610780 | Degradation of GLI1 by the proteasome | 3/597 | 60/10867 | 6.5E-01 | 8.3E-01 | 7.5E-01 | PSMD11/PSMD7/PSMD8 | 3 |
| R-HSA-9664323 | FCGR3A-mediated IL10 synthesis | 2/597 | 40/10867 | 6.5E-01 | 8.4E-01 | 7.5E-01 | FYN/HCK | 2 |
| R-HSA-72689 | Formation of a pool of free 40S subunits | 5/597 | 101/10867 | 6.6E-01 | 8.4E-01 | 7.5E-01 | RPL10/RPL28/RPL31/RPS14/RPS9 | 5 |
| R-HSA-190872 | Transport of connexons to the plasma membrane | 1/597 | 19/10867 | 6.6E-01 | 8.4E-01 | 7.5E-01 | TUBA1C | 1 |
| R-HSA-197264 | Nicotinamide salvaging | 1/597 | 19/10867 | 6.6E-01 | 8.4E-01 | 7.5E-01 | PTGS2 | 1 |
| R-HSA-202430 | Translocation of ZAP-70 to Immunological synapse | 1/597 | 19/10867 | 6.6E-01 | 8.4E-01 | 7.5E-01 | LCK | 1 |
| R-HSA-5576886 | Phase 4 - resting membrane potential | 1/597 | 19/10867 | 6.6E-01 | 8.4E-01 | 7.5E-01 | KCNK10 | 1 |
| R-HSA-77595 | Processing of Intronless Pre-mRNAs | 1/597 | 19/10867 | 6.6E-01 | 8.4E-01 | 7.5E-01 | CPSF4 | 1 |
| R-HSA-8964038 | LDL clearance | 1/597 | 19/10867 | 6.6E-01 | 8.4E-01 | 7.5E-01 | CLTC | 1 |
| R-HSA-9711123 | Cellular response to chemical stress | 8/597 | 160/10867 | 6.6E-01 | 8.4E-01 | 7.5E-01 | ATP7A/CUL3/KEAP1/PSMD11/PSMD7/PSMD8/SKP2/TXNIP | 8 |
| R-HSA-5678895 | Defective CFTR causes cystic fibrosis | 3/597 | 61/10867 | 6.6E-01 | 8.4E-01 | 7.5E-01 | PSMD11/PSMD7/PSMD8 | 3 |
| R-HSA-6790901 | rRNA modification in the nucleus and cytosol | 3/597 | 61/10867 | 6.6E-01 | 8.4E-01 | 7.5E-01 | NOL11/RPS14/RPS9 | 3 |
| R-HSA-913531 | Interferon Signaling | 10/597 | 199/10867 | 6.6E-01 | 8.4E-01 | 7.5E-01 | EGR1/HLA-A/KPNA1/KPNA2/KPNA4/NEDD4/NUP160/PIN1/RAE1/TYK2 | 10 |
| R-HSA-109581 | Apoptosis | 9/597 | 180/10867 | 6.6E-01 | 8.4E-01 | 7.5E-01 | CTNNB1/DBNL/KPNA1/LMNB1/PSMD11/PSMD7/PSMD8/TJP1/YWHAG | 9 |
| R-HSA-446652 | Interleukin-1 family signaling | 7/597 | 141/10867 | 6.6E-01 | 8.4E-01 | 7.5E-01 | IL18R1/IL1RAP/MAP3K8/PSMD11/PSMD7/PSMD8/TNIP2 | 7 |
| R-HSA-1852241 | Organelle biogenesis and maintenance | 15/597 | 296/10867 | 6.6E-01 | 8.4E-01 | 7.6E-01 | ATP5F1B/CCT8/ESRRA/HSP90AA1/IDH2/INPP5E/MAPRE1/MEF2D/NPHP3/PKD2/PRKAG1/TCP1/TUBA1C/TUBG1/YWHAG | 15 |
| R-HSA-5668541 | TNFR2 non-canonical NF-kB pathway | 5/597 | 102/10867 | 6.7E-01 | 8.4E-01 | 7.6E-01 | PSMD11/PSMD7/PSMD8/RELB/TNFRSF1B | 5 |
| R-HSA-168928 | DDX58/IFIH1-mediated induction of interferon-alpha/beta | 4/597 | 82/10867 | 6.7E-01 | 8.4E-01 | 7.6E-01 | HSP90AA1/NLRX1/PIN1/TNFAIP3 | 4 |
| R-HSA-186712 | Regulation of beta-cell development | 2/597 | 41/10867 | 6.7E-01 | 8.4E-01 | 7.6E-01 | FOXO1/MAML2 | 2 |
| R-HSA-5617833 | Cilium Assembly | 10/597 | 201/10867 | 6.7E-01 | 8.5E-01 | 7.6E-01 | CCT8/HSP90AA1/INPP5E/MAPRE1/NPHP3/PKD2/TCP1/TUBA1C/TUBG1/YWHAG | 10 |
| R-HSA-382556 | ABC-family proteins mediated transport | 5/597 | 103/10867 | 6.7E-01 | 8.5E-01 | 7.6E-01 | ABCA6/EIF2S1/PSMD11/PSMD7/PSMD8 | 5 |
| R-HSA-5688426 | Deubiquitination | 15/597 | 298/10867 | 6.8E-01 | 8.5E-01 | 7.6E-01 | CCNA2/CDC25A/FKBP8/FOXK2/H2BC13/H2BC5/KEAP1/PSMD11/PSMD7/PSMD8/PTEN/SKP2/TNFAIP3/TNIP2/USP37 | 15 |
| R-HSA-1168372 | Downstream signaling events of B Cell Receptor (BCR) | 4/597 | 83/10867 | 6.8E-01 | 8.5E-01 | 7.6E-01 | NRAS/PSMD11/PSMD7/PSMD8 | 4 |
| R-HSA-1181150 | Signaling by NODAL | 1/597 | 20/10867 | 6.8E-01 | 8.5E-01 | 7.6E-01 | FOXO3 | 1 |
| R-HSA-176407 | Conversion from APC/C:Cdc20 to APC/C:Cdh1 in late anaphase | 1/597 | 20/10867 | 6.8E-01 | 8.5E-01 | 7.6E-01 | ANAPC2 | 1 |
| R-HSA-176412 | Phosphorylation of the APC/C | 1/597 | 20/10867 | 6.8E-01 | 8.5E-01 | 7.6E-01 | ANAPC2 | 1 |
| R-HSA-9609507 | Protein localization | 8/597 | 163/10867 | 6.8E-01 | 8.5E-01 | 7.6E-01 | ATP5F1B/EPHX2/GRPEL1/GRPEL2/GSTK1/SLC25A12/SLC25A13/TIMM13 | 8 |
| R-HSA-379724 | tRNA Aminoacylation | 2/597 | 42/10867 | 6.8E-01 | 8.5E-01 | 7.6E-01 | GARS1/MARS1 | 2 |
| R-HSA-73728 | RNA Polymerase I Promoter Opening | 3/597 | 63/10867 | 6.8E-01 | 8.5E-01 | 7.6E-01 | H2AX/H2BC13/H2BC5 | 3 |
| R-HSA-1483255 | PI Metabolism | 4/597 | 84/10867 | 6.9E-01 | 8.5E-01 | 7.6E-01 | INPP5E/PIK3R1/PIP4K2C/PTEN | 4 |
| R-HSA-381340 | Transcriptional regulation of white adipocyte differentiation | 4/597 | 84/10867 | 6.9E-01 | 8.5E-01 | 7.6E-01 | MED14/MED16/MED20/PPARG | 4 |
| R-HSA-2187338 | Visual phototransduction | 5/597 | 105/10867 | 6.9E-01 | 8.5E-01 | 7.6E-01 | AKR1C3/DHRS3/NAPEPLD/PRKCA/SDC1 | 5 |
| R-HSA-1489509 | DAG and IP3 signaling | 2/597 | 43/10867 | 6.9E-01 | 8.5E-01 | 7.6E-01 | KPNA2/PRKCA | 2 |
| R-HSA-168325 | Viral Messenger RNA Synthesis | 2/597 | 43/10867 | 6.9E-01 | 8.5E-01 | 7.6E-01 | NUP160/RAE1 | 2 |
| R-HSA-5675221 | Negative regulation of MAPK pathway | 2/597 | 43/10867 | 6.9E-01 | 8.5E-01 | 7.6E-01 | DUSP6/NRAS | 2 |
| R-HSA-9646399 | Aggrephagy | 2/597 | 43/10867 | 6.9E-01 | 8.5E-01 | 7.6E-01 | HSP90AA1/TUBA1C | 2 |
| R-HSA-977444 | GABA B receptor activation | 2/597 | 43/10867 | 6.9E-01 | 8.5E-01 | 7.6E-01 | GNB4/GNG12 | 2 |
| R-HSA-991365 | Activation of GABAB receptors | 2/597 | 43/10867 | 6.9E-01 | 8.5E-01 | 7.6E-01 | GNB4/GNG12 | 2 |
| R-HSA-168142 | Toll Like Receptor 10 (TLR10) Cascade | 4/597 | 85/10867 | 6.9E-01 | 8.5E-01 | 7.6E-01 | DUSP6/MAP3K8/TNIP2/VRK3 | 4 |
| R-HSA-168176 | Toll Like Receptor 5 (TLR5) Cascade | 4/597 | 85/10867 | 6.9E-01 | 8.5E-01 | 7.6E-01 | DUSP6/MAP3K8/TNIP2/VRK3 | 4 |
| R-HSA-975871 | MyD88 cascade initiated on plasma membrane | 4/597 | 85/10867 | 6.9E-01 | 8.5E-01 | 7.6E-01 | DUSP6/MAP3K8/TNIP2/VRK3 | 4 |
| R-HSA-1606322 | ZBP1(DAI) mediated induction of type I IFNs | 1/597 | 21/10867 | 7.0E-01 | 8.5E-01 | 7.6E-01 | DHX9 | 1 |
| R-HSA-196741 | Cobalamin (Cbl. vitamin B12) transport and metabolism | 1/597 | 21/10867 | 7.0E-01 | 8.5E-01 | 7.6E-01 | ABCD4 | 1 |
| R-HSA-210745 | Regulation of gene expression in beta cells | 1/597 | 21/10867 | 7.0E-01 | 8.5E-01 | 7.6E-01 | FOXO1 | 1 |
| R-HSA-379726 | Mitochondrial tRNA aminoacylation | 1/597 | 21/10867 | 7.0E-01 | 8.5E-01 | 7.6E-01 | GARS1 | 1 |
| R-HSA-381771 | Synthesis. secretion. and inactivation of Glucagon-like Peptide-1 (GLP-1) | 1/597 | 21/10867 | 7.0E-01 | 8.5E-01 | 7.6E-01 | CTNNB1 | 1 |
| R-HSA-442982 | Ras activation upon Ca2+ influx through NMDA receptor | 1/597 | 21/10867 | 7.0E-01 | 8.5E-01 | 7.6E-01 | NRAS | 1 |
| R-HSA-5620916 | VxPx cargo-targeting to cilium | 1/597 | 21/10867 | 7.0E-01 | 8.5E-01 | 7.6E-01 | PKD2 | 1 |
| R-HSA-622312 | Inflammasomes | 1/597 | 21/10867 | 7.0E-01 | 8.5E-01 | 7.6E-01 | TXNIP | 1 |
| R-HSA-8849932 | Synaptic adhesion-like molecules | 1/597 | 21/10867 | 7.0E-01 | 8.5E-01 | 7.6E-01 | PTPRD | 1 |
| R-HSA-9008059 | Interleukin-37 signaling | 1/597 | 21/10867 | 7.0E-01 | 8.5E-01 | 7.6E-01 | IL18R1 | 1 |
| R-HSA-5578749 | Transcriptional regulation by small RNAs | 5/597 | 106/10867 | 7.0E-01 | 8.6E-01 | 7.7E-01 | H2AX/H2BC13/H2BC5/NUP160/RAE1 | 5 |
| R-HSA-5358346 | Hedgehog ligand biogenesis | 3/597 | 65/10867 | 7.0E-01 | 8.6E-01 | 7.7E-01 | PSMD11/PSMD7/PSMD8 | 3 |
| R-HSA-6782210 | Gap-filling DNA repair synthesis and ligation in TC-NER | 3/597 | 65/10867 | 7.0E-01 | 8.6E-01 | 7.7E-01 | PCNA/RFC5/XAB2 | 3 |
| R-HSA-1660661 | Sphingolipid de novo biosynthesis | 2/597 | 44/10867 | 7.0E-01 | 8.6E-01 | 7.7E-01 | PLPP3/PRKD2 | 2 |
| R-HSA-2173793 | Transcriptional activity of SMAD2/SMAD3:SMAD4 heterotrimer | 2/597 | 44/10867 | 7.0E-01 | 8.6E-01 | 7.7E-01 | JUNB/WWTR1 | 2 |
| R-HSA-6811438 | Intra-Golgi traffic | 2/597 | 44/10867 | 7.0E-01 | 8.6E-01 | 7.7E-01 | CYTH2/GOLIM4 | 2 |
| R-HSA-75893 | TNF signaling | 2/597 | 44/10867 | 7.0E-01 | 8.6E-01 | 7.7E-01 | OTULIN/TNFAIP3 | 2 |
| R-HSA-975634 | Retinoid metabolism and transport | 2/597 | 44/10867 | 7.0E-01 | 8.6E-01 | 7.7E-01 | AKR1C3/SDC1 | 2 |
| R-HSA-2024096 | HS-GAG degradation | 1/597 | 22/10867 | 7.1E-01 | 8.6E-01 | 7.7E-01 | SDC1 | 1 |
| R-HSA-202427 | Phosphorylation of CD3 and TCR zeta chains | 1/597 | 22/10867 | 7.1E-01 | 8.6E-01 | 7.7E-01 | LCK | 1 |
| R-HSA-3296482 | Defects in vitamin and cofactor metabolism | 1/597 | 22/10867 | 7.1E-01 | 8.6E-01 | 7.7E-01 | ABCD4 | 1 |
| R-HSA-389977 | Post-chaperonin tubulin folding pathway | 1/597 | 22/10867 | 7.1E-01 | 8.6E-01 | 7.7E-01 | TUBA1C | 1 |
| R-HSA-71403 | Citric acid cycle (TCA cycle) | 1/597 | 22/10867 | 7.1E-01 | 8.6E-01 | 7.7E-01 | IDH2 | 1 |
| R-HSA-9033658 | Blood group systems biosynthesis | 1/597 | 22/10867 | 7.1E-01 | 8.6E-01 | 7.7E-01 | FUT11 | 1 |
| R-HSA-9613829 | Chaperone Mediated Autophagy | 1/597 | 22/10867 | 7.1E-01 | 8.6E-01 | 7.7E-01 | HSP90AA1 | 1 |
| R-HSA-9617324 | Negative regulation of NMDA receptor-mediated neuronal transmission | 1/597 | 22/10867 | 7.1E-01 | 8.6E-01 | 7.7E-01 | PPM1F | 1 |
| R-HSA-3899300 | SUMOylation of transcription cofactors | 2/597 | 45/10867 | 7.2E-01 | 8.7E-01 | 7.8E-01 | CBX2/RING1 | 2 |
| R-HSA-5358351 | Signaling by Hedgehog | 7/597 | 149/10867 | 7.2E-01 | 8.7E-01 | 7.8E-01 | CUL3/DZIP1/GSK3B/PSMD11/PSMD7/PSMD8/TUBA1C | 7 |
| R-HSA-1483206 | Glycerophospholipid biosynthesis | 6/597 | 129/10867 | 7.2E-01 | 8.7E-01 | 7.8E-01 | BCHE/GPAT3/GPCPD1/MIGA2/PLA2G2A/PLA2G4A | 6 |
| R-HSA-1169091 | Activation of NF-kappaB in B cells | 3/597 | 67/10867 | 7.2E-01 | 8.7E-01 | 7.8E-01 | PSMD11/PSMD7/PSMD8 | 3 |
| R-HSA-5625886 | Activated PKN1 stimulates transcription of AR (androgen receptor) regulated genes KLK2 and KLK3 | 3/597 | 67/10867 | 7.2E-01 | 8.7E-01 | 7.8E-01 | H2AX/H2BC13/H2BC5 | 3 |
| R-HSA-1660662 | Glycosphingolipid metabolism | 2/597 | 46/10867 | 7.3E-01 | 8.7E-01 | 7.8E-01 | ARSB/GBA2 | 2 |
| R-HSA-8854214 | TBC/RABGAPs | 2/597 | 46/10867 | 7.3E-01 | 8.7E-01 | 7.8E-01 | GGA1/RAB11B | 2 |
| R-HSA-140837 | Intrinsic Pathway of Fibrin Clot Formation | 1/597 | 23/10867 | 7.3E-01 | 8.7E-01 | 7.8E-01 | PROS1 | 1 |
| R-HSA-166663 | Initial triggering of complement | 1/597 | 23/10867 | 7.3E-01 | 8.7E-01 | 7.8E-01 | CFD | 1 |
| R-HSA-167242 | Abortive elongation of HIV-1 transcript in the absence of Tat | 1/597 | 23/10867 | 7.3E-01 | 8.7E-01 | 7.8E-01 | NELFB | 1 |
| R-HSA-171319 | Telomere Extension By Telomerase | 1/597 | 23/10867 | 7.3E-01 | 8.7E-01 | 7.8E-01 | CCNA2 | 1 |
| R-HSA-212676 | Dopamine Neurotransmitter Release Cycle | 1/597 | 23/10867 | 7.3E-01 | 8.7E-01 | 7.8E-01 | SYN2 | 1 |
| R-HSA-2453902 | The canonical retinoid cycle in rods (twilight vision) | 1/597 | 23/10867 | 7.3E-01 | 8.7E-01 | 7.8E-01 | NAPEPLD | 1 |
| R-HSA-389948 | PD-1 signaling | 1/597 | 23/10867 | 7.3E-01 | 8.7E-01 | 7.8E-01 | LCK | 1 |
| R-HSA-8949215 | Mitochondrial calcium ion transport | 1/597 | 23/10867 | 7.3E-01 | 8.7E-01 | 7.8E-01 | MCU | 1 |
| R-HSA-112315 | Transmission across Chemical Synapses | 13/597 | 270/10867 | 7.3E-01 | 8.7E-01 | 7.8E-01 | ARL6IP5/BCHE/GNB4/GNG12/KPNA2/MAOA/NRAS/PPM1F/PRKAA1/PRKAG1/PRKCA/SYN2/TUBA1C | 13 |
| R-HSA-3906995 | Diseases associated with O-glycosylation of proteins | 3/597 | 68/10867 | 7.3E-01 | 8.7E-01 | 7.8E-01 | ADAMTS2/NOTCH2/THSD7A | 3 |
| R-HSA-427359 | SIRT1 negatively regulates rRNA expression | 3/597 | 68/10867 | 7.3E-01 | 8.7E-01 | 7.8E-01 | H2AX/H2BC13/H2BC5 | 3 |
| R-HSA-9670095 | Inhibition of DNA recombination at telomere | 3/597 | 68/10867 | 7.3E-01 | 8.7E-01 | 7.8E-01 | H2AX/H2BC13/H2BC5 | 3 |
| R-HSA-72766 | Translation | 14/597 | 291/10867 | 7.3E-01 | 8.7E-01 | 7.8E-01 | EIF2S1/GARS1/MARS1/MRPL12/MRPL15/MRPL17/MRPL58/MRPS2/RPL10/RPL28/RPL31/RPN2/RPS14/RPS9 | 14 |
| R-HSA-1483257 | Phospholipid metabolism | 10/597 | 212/10867 | 7.3E-01 | 8.7E-01 | 7.8E-01 | BCHE/GPAT3/GPCPD1/INPP5E/MIGA2/PIK3R1/PIP4K2C/PLA2G2A/PLA2G4A/PTEN | 10 |
| R-HSA-428157 | Sphingolipid metabolism | 4/597 | 90/10867 | 7.4E-01 | 8.7E-01 | 7.8E-01 | ARSB/GBA2/PLPP3/PRKD2 | 4 |
| R-HSA-9616222 | Transcriptional regulation of granulopoiesis | 4/597 | 90/10867 | 7.4E-01 | 8.7E-01 | 7.8E-01 | H2AX/H2BC13/H2BC5/RUNX1 | 4 |
| R-HSA-977225 | Amyloid fiber formation | 5/597 | 111/10867 | 7.4E-01 | 8.7E-01 | 7.8E-01 | CST3/GGA1/H2AX/H2BC13/H2BC5 | 5 |
| R-HSA-190828 | Gap junction trafficking | 2/597 | 47/10867 | 7.4E-01 | 8.7E-01 | 7.8E-01 | CLTC/TUBA1C | 2 |
| R-HSA-9707616 | Heme signaling | 2/597 | 47/10867 | 7.4E-01 | 8.7E-01 | 7.8E-01 | MEF2D/NPAS2 | 2 |
| R-HSA-3371453 | Regulation of HSF1-mediated heat shock response | 3/597 | 69/10867 | 7.4E-01 | 8.7E-01 | 7.8E-01 | GSK3B/NUP160/RAE1 | 3 |
| R-HSA-112316 | Neuronal System | 20/597 | 410/10867 | 7.4E-01 | 8.7E-01 | 7.8E-01 | ARL6IP5/BCHE/DBNL/GNB4/GNG12/IL1RAP/KCNA1/KCNK10/KPNA2/MAOA/NLGN1/NLGN2/NRAS/PPM1F/PRKAA1/PRKAG1/PRKCA/PTPRD/SYN2/TUBA1C | 20 |
| R-HSA-174048 | APC/C:Cdc20 mediated degradation of Cyclin B | 1/597 | 24/10867 | 7.4E-01 | 8.7E-01 | 7.8E-01 | ANAPC2 | 1 |
| R-HSA-193368 | Synthesis of bile acids and bile salts via 7alpha-hydroxycholesterol | 1/597 | 24/10867 | 7.4E-01 | 8.7E-01 | 7.8E-01 | AKR1C3 | 1 |
| R-HSA-203927 | MicroRNA (miRNA) biogenesis | 1/597 | 24/10867 | 7.4E-01 | 8.7E-01 | 7.8E-01 | XPO5 | 1 |
| R-HSA-210500 | Glutamate Neurotransmitter Release Cycle | 1/597 | 24/10867 | 7.4E-01 | 8.7E-01 | 7.8E-01 | ARL6IP5 | 1 |
| R-HSA-400508 | Incretin synthesis. secretion. and inactivation | 1/597 | 24/10867 | 7.4E-01 | 8.7E-01 | 7.8E-01 | CTNNB1 | 1 |
| R-HSA-5627123 | RHO GTPases activate PAKs | 1/597 | 24/10867 | 7.4E-01 | 8.7E-01 | 7.8E-01 | PPP1CB | 1 |
| R-HSA-977068 | Termination of O-glycan biosynthesis | 1/597 | 24/10867 | 7.4E-01 | 8.7E-01 | 7.8E-01 | ST6GALNAC3 | 1 |
| R-HSA-975138 | TRAF6 mediated induction of NFkB and MAP kinases upon TLR7/8 or 9 activation | 4/597 | 91/10867 | 7.4E-01 | 8.7E-01 | 7.8E-01 | DUSP6/MAP3K8/TNIP2/VRK3 | 4 |
| R-HSA-909733 | Interferon alpha/beta signaling | 3/597 | 70/10867 | 7.5E-01 | 8.7E-01 | 7.8E-01 | EGR1/HLA-A/TYK2 | 3 |
| R-HSA-6806667 | Metabolism of fat-soluble vitamins | 2/597 | 48/10867 | 7.5E-01 | 8.7E-01 | 7.8E-01 | AKR1C3/SDC1 | 2 |
| R-HSA-73893 | DNA Damage Bypass | 2/597 | 48/10867 | 7.5E-01 | 8.7E-01 | 7.8E-01 | PCNA/RFC5 | 2 |
| R-HSA-5610787 | Hedgehog 'off' state | 5/597 | 113/10867 | 7.5E-01 | 8.8E-01 | 7.8E-01 | GSK3B/PSMD11/PSMD7/PSMD8/TUBA1C | 5 |
| R-HSA-975155 | MyD88 dependent cascade initiated on endosome | 4/597 | 92/10867 | 7.5E-01 | 8.8E-01 | 7.8E-01 | DUSP6/MAP3K8/TNIP2/VRK3 | 4 |
| R-HSA-2454202 | Fc epsilon receptor (FCERI) signaling | 6/597 | 134/10867 | 7.5E-01 | 8.8E-01 | 7.8E-01 | FYN/NRAS/PIK3R1/PSMD11/PSMD7/PSMD8 | 6 |
| R-HSA-416476 | G alpha (q) signalling events | 10/597 | 216/10867 | 7.6E-01 | 8.8E-01 | 7.8E-01 | ANXA1/EDN1/GNB4/GNG12/NRAS/P2RY11/PIK3R1/PRKCA/RGS2/RGS5 | 10 |
| R-HSA-3214841 | PKMTs methylate histone lysines | 3/597 | 71/10867 | 7.6E-01 | 8.8E-01 | 7.8E-01 | EZH2/KMT2E/SUV39H2 | 3 |
| R-HSA-70171 | Glycolysis | 3/597 | 71/10867 | 7.6E-01 | 8.8E-01 | 7.8E-01 | NUP160/PFKL/RAE1 | 3 |
| R-HSA-1187000 | Fertilization | 1/597 | 25/10867 | 7.6E-01 | 8.8E-01 | 7.8E-01 | HVCN1 | 1 |
| R-HSA-156588 | Glucuronidation | 1/597 | 25/10867 | 7.6E-01 | 8.8E-01 | 7.8E-01 | SLC35D1 | 1 |
| R-HSA-191273 | Cholesterol biosynthesis | 1/597 | 25/10867 | 7.6E-01 | 8.8E-01 | 7.8E-01 | HMGCS1 | 1 |
| R-HSA-2029485 | Role of phospholipids in phagocytosis | 1/597 | 25/10867 | 7.6E-01 | 8.8E-01 | 7.8E-01 | PIK3R1 | 1 |
| R-HSA-166016 | Toll Like Receptor 4 (TLR4) Cascade | 6/597 | 135/10867 | 7.6E-01 | 8.8E-01 | 7.9E-01 | CD180/DUSP6/ITGB2/MAP3K8/TNIP2/VRK3 | 6 |
| R-HSA-168181 | Toll Like Receptor 7/8 (TLR7/8) Cascade | 4/597 | 93/10867 | 7.6E-01 | 8.8E-01 | 7.9E-01 | DUSP6/MAP3K8/TNIP2/VRK3 | 4 |
| R-HSA-927802 | Nonsense-Mediated Decay (NMD) | 5/597 | 115/10867 | 7.6E-01 | 8.8E-01 | 7.9E-01 | RPL10/RPL28/RPL31/RPS14/RPS9 | 5 |
| R-HSA-975957 | Nonsense Mediated Decay (NMD) enhanced by the Exon Junction Complex (EJC) | 5/597 | 115/10867 | 7.6E-01 | 8.8E-01 | 7.9E-01 | RPL10/RPL28/RPL31/RPS14/RPS9 | 5 |
| R-HSA-168898 | Toll-like Receptor Cascades | 7/597 | 157/10867 | 7.7E-01 | 8.8E-01 | 7.9E-01 | CD180/DUSP6/HSP90B1/ITGB2/MAP3K8/TNIP2/VRK3 | 7 |
| R-HSA-168164 | Toll Like Receptor 3 (TLR3) Cascade | 4/597 | 94/10867 | 7.7E-01 | 8.8E-01 | 7.9E-01 | DUSP6/MAP3K8/TNIP2/VRK3 | 4 |
| R-HSA-1483249 | Inositol phosphate metabolism | 2/597 | 50/10867 | 7.7E-01 | 8.8E-01 | 7.9E-01 | INPP1/PTEN | 2 |
| R-HSA-3214842 | HDMs demethylate histones | 2/597 | 50/10867 | 7.7E-01 | 8.8E-01 | 7.9E-01 | ARID5B/KDM5B | 2 |
| R-HSA-381038 | XBP1(S) activates chaperone genes | 2/597 | 50/10867 | 7.7E-01 | 8.8E-01 | 7.9E-01 | DNAJB11/GSK3A | 2 |
| R-HSA-180024 | DARPP-32 events | 1/597 | 26/10867 | 7.7E-01 | 8.8E-01 | 7.9E-01 | PPP1CA | 1 |
| R-HSA-425410 | Metal ion SLC transporters | 1/597 | 26/10867 | 7.7E-01 | 8.8E-01 | 7.9E-01 | SLC30A7 | 1 |
| R-HSA-912694 | Regulation of IFNA signaling | 1/597 | 26/10867 | 7.7E-01 | 8.8E-01 | 7.9E-01 | TYK2 | 1 |
| R-HSA-9660826 | Purinergic signaling in leishmaniasis infection | 1/597 | 26/10867 | 7.7E-01 | 8.8E-01 | 7.9E-01 | TXNIP | 1 |
| R-HSA-9664424 | Cell recruitment (pro-inflammatory response) | 1/597 | 26/10867 | 7.7E-01 | 8.8E-01 | 7.9E-01 | TXNIP | 1 |
| R-HSA-6807505 | RNA polymerase II transcribes snRNA genes | 3/597 | 74/10867 | 7.8E-01 | 8.9E-01 | 8.0E-01 | INTS11/SNAPC4/TAF11 | 3 |
| R-HSA-168138 | Toll Like Receptor 9 (TLR9) Cascade | 4/597 | 96/10867 | 7.8E-01 | 8.9E-01 | 8.0E-01 | DUSP6/MAP3K8/TNIP2/VRK3 | 4 |
| R-HSA-201556 | Signaling by ALK | 1/597 | 27/10867 | 7.8E-01 | 8.9E-01 | 8.0E-01 | PIK3R1 | 1 |
| R-HSA-264876 | Insulin processing | 1/597 | 27/10867 | 7.8E-01 | 8.9E-01 | 8.0E-01 | SLC30A7 | 1 |
| R-HSA-354192 | Integrin signaling | 1/597 | 27/10867 | 7.8E-01 | 8.9E-01 | 8.0E-01 | ITGB3 | 1 |
| R-HSA-5621480 | Dectin-2 family | 1/597 | 27/10867 | 7.8E-01 | 8.9E-01 | 8.0E-01 | FYN | 1 |
| R-HSA-9615933 | Postmitotic nuclear pore complex (NPC) reformation | 1/597 | 27/10867 | 7.8E-01 | 8.9E-01 | 8.0E-01 | NUP160 | 1 |
| R-HSA-381070 | IRE1alpha activates chaperones | 2/597 | 52/10867 | 7.9E-01 | 8.9E-01 | 8.0E-01 | DNAJB11/GSK3A | 2 |
| R-HSA-73887 | Death Receptor Signalling | 6/597 | 141/10867 | 7.9E-01 | 9.0E-01 | 8.0E-01 | ARHGEF12/ARHGEF3/OTULIN/PRKCI/TNFAIP3/TNFRSF10D | 6 |
| R-HSA-5620912 | Anchoring of the basal body to the plasma membrane | 4/597 | 98/10867 | 7.9E-01 | 9.0E-01 | 8.0E-01 | HSP90AA1/MAPRE1/TUBG1/YWHAG | 4 |
| R-HSA-1483191 | Synthesis of PC | 1/597 | 28/10867 | 7.9E-01 | 9.0E-01 | 8.0E-01 | BCHE | 1 |
| R-HSA-1855204 | Synthesis of IP3 and IP4 in the cytosol | 1/597 | 28/10867 | 7.9E-01 | 9.0E-01 | 8.0E-01 | PTEN | 1 |
| R-HSA-2022854 | Keratan sulfate biosynthesis | 1/597 | 28/10867 | 7.9E-01 | 9.0E-01 | 8.0E-01 | B4GALT5 | 1 |
| R-HSA-5620971 | Pyroptosis | 1/597 | 28/10867 | 7.9E-01 | 9.0E-01 | 8.0E-01 | CHMP3 | 1 |
| R-HSA-75067 | Processing of Capped Intronless Pre-mRNA | 1/597 | 28/10867 | 7.9E-01 | 9.0E-01 | 8.0E-01 | CPSF4 | 1 |
| R-HSA-191859 | snRNP Assembly | 2/597 | 53/10867 | 8.0E-01 | 9.0E-01 | 8.0E-01 | NUP160/RAE1 | 2 |
| R-HSA-194441 | Metabolism of non-coding RNA | 2/597 | 53/10867 | 8.0E-01 | 9.0E-01 | 8.0E-01 | NUP160/RAE1 | 2 |
| R-HSA-5621481 | C-type lectin receptors (CLRs) | 6/597 | 142/10867 | 8.0E-01 | 9.0E-01 | 8.1E-01 | FYN/NRAS/PSMD11/PSMD7/PSMD8/RELB | 6 |
| R-HSA-166166 | MyD88-independent TLR4 cascade | 4/597 | 99/10867 | 8.0E-01 | 9.0E-01 | 8.1E-01 | DUSP6/MAP3K8/TNIP2/VRK3 | 4 |
| R-HSA-937061 | TRIF(TICAM1)-mediated TLR4 signaling | 4/597 | 99/10867 | 8.0E-01 | 9.0E-01 | 8.1E-01 | DUSP6/MAP3K8/TNIP2/VRK3 | 4 |
| R-HSA-112314 | Neurotransmitter receptors and postsynaptic signal transmission | 9/597 | 205/10867 | 8.0E-01 | 9.0E-01 | 8.1E-01 | GNB4/GNG12/KPNA2/NRAS/PPM1F/PRKAA1/PRKAG1/PRKCA/TUBA1C | 9 |
| R-HSA-5617472 | Activation of anterior HOX genes in hindbrain development during early embryogenesis | 5/597 | 121/10867 | 8.0E-01 | 9.0E-01 | 8.1E-01 | EZH2/H2AX/H2BC13/H2BC5/HOXA3 | 5 |
| R-HSA-5619507 | Activation of HOX genes during differentiation | 5/597 | 121/10867 | 8.0E-01 | 9.0E-01 | 8.1E-01 | EZH2/H2AX/H2BC13/H2BC5/HOXA3 | 5 |
| R-HSA-948021 | Transport to the Golgi and subsequent modification | 8/597 | 185/10867 | 8.0E-01 | 9.0E-01 | 8.1E-01 | B4GALT5/CD59/COPG1/LMAN2/MCFD2/MGAT3/SEC23A/TUBA1C | 8 |
| R-HSA-442742 | CREB1 phosphorylation through NMDA receptor-mediated activation of RAS signaling | 1/597 | 29/10867 | 8.1E-01 | 9.0E-01 | 8.1E-01 | NRAS | 1 |
| R-HSA-5213460 | RIPK1-mediated regulated necrosis | 1/597 | 29/10867 | 8.1E-01 | 9.0E-01 | 8.1E-01 | HSP90AA1 | 1 |
| R-HSA-5601884 | PIWI-interacting RNA (piRNA) biogenesis | 1/597 | 29/10867 | 8.1E-01 | 9.0E-01 | 8.1E-01 | HSP90AA1 | 1 |
| R-HSA-5669034 | TNFs bind their physiological receptors | 1/597 | 29/10867 | 8.1E-01 | 9.0E-01 | 8.1E-01 | TNFRSF1B | 1 |
| R-HSA-5675482 | Regulation of necroptotic cell death | 1/597 | 29/10867 | 8.1E-01 | 9.0E-01 | 8.1E-01 | HSP90AA1 | 1 |
| R-HSA-166058 | MyD88:MAL(TIRAP) cascade initiated on plasma membrane | 4/597 | 101/10867 | 8.1E-01 | 9.0E-01 | 8.1E-01 | DUSP6/MAP3K8/TNIP2/VRK3 | 4 |
| R-HSA-168188 | Toll Like Receptor TLR6:TLR2 Cascade | 4/597 | 101/10867 | 8.1E-01 | 9.0E-01 | 8.1E-01 | DUSP6/MAP3K8/TNIP2/VRK3 | 4 |
| R-HSA-5607764 | CLEC7A (Dectin-1) signaling | 4/597 | 101/10867 | 8.1E-01 | 9.0E-01 | 8.1E-01 | PSMD11/PSMD7/PSMD8/RELB | 4 |
| R-HSA-2132295 | MHC class II antigen presentation | 5/597 | 123/10867 | 8.1E-01 | 9.0E-01 | 8.1E-01 | CLTC/CTSH/KIF20A/SEC23A/TUBA1C | 5 |
| R-HSA-1655829 | Regulation of cholesterol biosynthesis by SREBP (SREBF) | 2/597 | 55/10867 | 8.1E-01 | 9.0E-01 | 8.1E-01 | HMGCS1/SEC23A | 2 |
| R-HSA-71406 | Pyruvate metabolism and Citric Acid (TCA) cycle | 2/597 | 55/10867 | 8.1E-01 | 9.0E-01 | 8.1E-01 | IDH2/PDHX | 2 |
| R-HSA-9639288 | Amino acids regulate mTORC1 | 2/597 | 55/10867 | 8.1E-01 | 9.0E-01 | 8.1E-01 | LAMTOR4/RRAGC | 2 |
| R-HSA-114452 | Activation of BH3-only proteins | 1/597 | 30/10867 | 8.2E-01 | 9.0E-01 | 8.1E-01 | YWHAG | 1 |
| R-HSA-5357956 | TNFR1-induced NFkappaB signaling pathway | 1/597 | 30/10867 | 8.2E-01 | 9.0E-01 | 8.1E-01 | TNFAIP3 | 1 |
| R-HSA-9007101 | Rab regulation of trafficking | 5/597 | 124/10867 | 8.2E-01 | 9.0E-01 | 8.1E-01 | GDI1/GGA1/RAB11B/RAB27B/RIN2 | 5 |
| R-HSA-5689603 | UCH proteinases | 4/597 | 102/10867 | 8.2E-01 | 9.0E-01 | 8.1E-01 | FOXK2/PSMD11/PSMD7/PSMD8 | 4 |
| R-HSA-5578775 | Ion homeostasis | 2/597 | 56/10867 | 8.2E-01 | 9.0E-01 | 8.1E-01 | ATP1A2/DMPK | 2 |
| R-HSA-1296071 | Potassium Channels | 4/597 | 103/10867 | 8.2E-01 | 9.0E-01 | 8.1E-01 | GNB4/GNG12/KCNA1/KCNK10 | 4 |
| R-HSA-196807 | Nicotinate metabolism | 1/597 | 31/10867 | 8.3E-01 | 9.0E-01 | 8.1E-01 | PTGS2 | 1 |
| R-HSA-389661 | Glyoxylate metabolism and glycine degradation | 1/597 | 31/10867 | 8.3E-01 | 9.0E-01 | 8.1E-01 | PDHX | 1 |
| R-HSA-399719 | Trafficking of AMPA receptors | 1/597 | 31/10867 | 8.3E-01 | 9.0E-01 | 8.1E-01 | PRKCA | 1 |
| R-HSA-399721 | Glutamate binding. activation of AMPA receptors and synaptic plasticity | 1/597 | 31/10867 | 8.3E-01 | 9.0E-01 | 8.1E-01 | PRKCA | 1 |
| R-HSA-70268 | Pyruvate metabolism | 1/597 | 31/10867 | 8.3E-01 | 9.0E-01 | 8.1E-01 | PDHX | 1 |
| R-HSA-8949613 | Cristae formation | 1/597 | 31/10867 | 8.3E-01 | 9.0E-01 | 8.1E-01 | ATP5F1B | 1 |
| R-HSA-917977 | Transferrin endocytosis and recycling | 1/597 | 31/10867 | 8.3E-01 | 9.0E-01 | 8.1E-01 | HFE | 1 |
| R-HSA-9662851 | Anti-inflammatory response favouring Leishmania parasite infection | 7/597 | 169/10867 | 8.3E-01 | 9.0E-01 | 8.1E-01 | CALCRL/FYN/GNB4/GNG12/HCK/P2RY11/RAMP2 | 7 |
| R-HSA-9664433 | Leishmania parasite growth and survival | 7/597 | 169/10867 | 8.3E-01 | 9.0E-01 | 8.1E-01 | CALCRL/FYN/GNB4/GNG12/HCK/P2RY11/RAMP2 | 7 |
| R-HSA-168643 | Nucleotide-binding domain. leucine rich repeat containing receptor (NLR) signaling pathways | 2/597 | 57/10867 | 8.3E-01 | 9.0E-01 | 8.1E-01 | TNFAIP3/TXNIP | 2 |
| R-HSA-5218859 | Regulated Necrosis | 2/597 | 57/10867 | 8.3E-01 | 9.0E-01 | 8.1E-01 | CHMP3/HSP90AA1 | 2 |
| R-HSA-6799198 | Complex I biogenesis | 2/597 | 57/10867 | 8.3E-01 | 9.0E-01 | 8.1E-01 | NDUFS7/NDUFV1 | 2 |
| R-HSA-3247509 | Chromatin modifying enzymes | 12/597 | 274/10867 | 8.3E-01 | 9.0E-01 | 8.1E-01 | ARID5B/EZH2/H2AX/H2BC13/H2BC5/KDM5B/KMT2E/MBD3/MORF4L1/MSL3/SMARCC1/SUV39H2 | 12 |
| R-HSA-4839726 | Chromatin organization | 12/597 | 274/10867 | 8.3E-01 | 9.0E-01 | 8.1E-01 | ARID5B/EZH2/H2AX/H2BC13/H2BC5/KDM5B/KMT2E/MBD3/MORF4L1/MSL3/SMARCC1/SUV39H2 | 12 |
| R-HSA-9663891 | Selective autophagy | 3/597 | 81/10867 | 8.3E-01 | 9.0E-01 | 8.1E-01 | HSP90AA1/PRKAG1/TUBA1C | 3 |
| R-HSA-168179 | Toll Like Receptor TLR1:TLR2 Cascade | 4/597 | 104/10867 | 8.3E-01 | 9.0E-01 | 8.1E-01 | DUSP6/MAP3K8/TNIP2/VRK3 | 4 |
| R-HSA-181438 | Toll Like Receptor 2 (TLR2) Cascade | 4/597 | 104/10867 | 8.3E-01 | 9.0E-01 | 8.1E-01 | DUSP6/MAP3K8/TNIP2/VRK3 | 4 |
| R-HSA-2871837 | FCERI mediated NF-kB activation | 3/597 | 82/10867 | 8.4E-01 | 9.1E-01 | 8.1E-01 | PSMD11/PSMD7/PSMD8 | 3 |
| R-HSA-2871796 | FCERI mediated MAPK activation | 1/597 | 32/10867 | 8.4E-01 | 9.1E-01 | 8.1E-01 | NRAS | 1 |
| R-HSA-381042 | PERK regulates gene expression | 1/597 | 32/10867 | 8.4E-01 | 9.1E-01 | 8.1E-01 | EIF2S1 | 1 |
| R-HSA-418594 | G alpha (i) signalling events | 14/597 | 318/10867 | 8.4E-01 | 9.1E-01 | 8.2E-01 | ADRA2A/ANXA1/CCL5/CXCL2/CXCL6/GNB4/GNG12/GPSM1/GPSM2/KPNA2/PLA2G4A/PPP1CA/PRKCA/RGS5 | 14 |
| R-HSA-193648 | NRAGE signals death through JNK | 2/597 | 59/10867 | 8.4E-01 | 9.1E-01 | 8.2E-01 | ARHGEF12/ARHGEF3 | 2 |
| R-HSA-113418 | Formation of the Early Elongation Complex | 1/597 | 33/10867 | 8.5E-01 | 9.1E-01 | 8.2E-01 | NELFB | 1 |
| R-HSA-167158 | Formation of the HIV-1 Early Elongation Complex | 1/597 | 33/10867 | 8.5E-01 | 9.1E-01 | 8.2E-01 | NELFB | 1 |
| R-HSA-352230 | Amino acid transport across the plasma membrane | 1/597 | 33/10867 | 8.5E-01 | 9.1E-01 | 8.2E-01 | SLC36A1 | 1 |
| R-HSA-8964043 | Plasma lipoprotein clearance | 1/597 | 33/10867 | 8.5E-01 | 9.1E-01 | 8.2E-01 | CLTC | 1 |
| R-HSA-9022692 | Regulation of MECP2 expression and activity | 1/597 | 33/10867 | 8.5E-01 | 9.1E-01 | 8.2E-01 | AURKB | 1 |
| R-HSA-427413 | NoRC negatively regulates rRNA expression | 4/597 | 107/10867 | 8.5E-01 | 9.1E-01 | 8.2E-01 | DNMT3B/H2AX/H2BC13/H2BC5 | 4 |
| R-HSA-5696399 | Global Genome Nucleotide Excision Repair (GG-NER) | 3/597 | 84/10867 | 8.5E-01 | 9.1E-01 | 8.2E-01 | ERCC1/PCNA/RFC5 | 3 |
| R-HSA-977443 | GABA receptor activation | 2/597 | 60/10867 | 8.5E-01 | 9.1E-01 | 8.2E-01 | GNB4/GNG12 | 2 |
| R-HSA-1638074 | Keratan sulfate/keratin metabolism | 1/597 | 34/10867 | 8.5E-01 | 9.2E-01 | 8.2E-01 | B4GALT5 | 1 |
| R-HSA-167238 | Pausing and recovery of Tat-mediated HIV elongation | 1/597 | 34/10867 | 8.5E-01 | 9.2E-01 | 8.2E-01 | NELFB | 1 |
| R-HSA-167243 | Tat-mediated HIV elongation arrest and recovery | 1/597 | 34/10867 | 8.5E-01 | 9.2E-01 | 8.2E-01 | NELFB | 1 |
| R-HSA-192105 | Synthesis of bile acids and bile salts | 1/597 | 34/10867 | 8.5E-01 | 9.2E-01 | 8.2E-01 | AKR1C3 | 1 |
| R-HSA-2029482 | Regulation of actin dynamics for phagocytic cup formation | 2/597 | 61/10867 | 8.6E-01 | 9.2E-01 | 8.2E-01 | HSP90AA1/WASF1 | 2 |
| R-HSA-2672351 | Stimuli-sensing channels | 4/597 | 109/10867 | 8.6E-01 | 9.2E-01 | 8.2E-01 | ANO9/SGK2/TSC22D3/TTYH2 | 4 |
| R-HSA-199977 | ER to Golgi Anterograde Transport | 6/597 | 154/10867 | 8.6E-01 | 9.2E-01 | 8.2E-01 | CD59/COPG1/LMAN2/MCFD2/SEC23A/TUBA1C | 6 |
| R-HSA-5250941 | Negative epigenetic regulation of rRNA expression | 4/597 | 110/10867 | 8.6E-01 | 9.2E-01 | 8.2E-01 | DNMT3B/H2AX/H2BC13/H2BC5 | 4 |
| R-HSA-73854 | RNA Polymerase I Promoter Clearance | 4/597 | 110/10867 | 8.6E-01 | 9.2E-01 | 8.2E-01 | H2AX/H2BC13/H2BC5/MBD3 | 4 |
| R-HSA-5696398 | Nucleotide Excision Repair | 4/597 | 111/10867 | 8.7E-01 | 9.2E-01 | 8.3E-01 | ERCC1/PCNA/RFC5/XAB2 | 4 |
| R-HSA-9660821 | ADORA2B mediated anti-inflammatory cytokines production | 5/597 | 134/10867 | 8.7E-01 | 9.2E-01 | 8.3E-01 | CALCRL/GNB4/GNG12/P2RY11/RAMP2 | 5 |
| R-HSA-211859 | Biological oxidations | 9/597 | 222/10867 | 8.7E-01 | 9.2E-01 | 8.3E-01 | ACSS1/ADH1A/ADH1C/ALDH1A1/ALDH3A1/CYP4F12/GSTK1/MAOA/SLC35D1 | 9 |
| R-HSA-9033241 | Peroxisomal protein import | 2/597 | 63/10867 | 8.7E-01 | 9.2E-01 | 8.3E-01 | EPHX2/GSTK1 | 2 |
| R-HSA-156590 | Glutathione conjugation | 1/597 | 36/10867 | 8.7E-01 | 9.2E-01 | 8.3E-01 | GSTK1 | 1 |
| R-HSA-167287 | HIV elongation arrest and recovery | 1/597 | 36/10867 | 8.7E-01 | 9.2E-01 | 8.3E-01 | NELFB | 1 |
| R-HSA-167290 | Pausing and recovery of HIV elongation | 1/597 | 36/10867 | 8.7E-01 | 9.2E-01 | 8.3E-01 | NELFB | 1 |
| R-HSA-190861 | Gap junction assembly | 1/597 | 36/10867 | 8.7E-01 | 9.2E-01 | 8.3E-01 | TUBA1C | 1 |
| R-HSA-5663213 | RHO GTPases Activate WASPs and WAVEs | 1/597 | 36/10867 | 8.7E-01 | 9.2E-01 | 8.3E-01 | WASF1 | 1 |
| R-HSA-6804757 | Regulation of TP53 Degradation | 1/597 | 36/10867 | 8.7E-01 | 9.2E-01 | 8.3E-01 | CCNA2 | 1 |
| R-HSA-73864 | RNA Polymerase I Transcription | 4/597 | 112/10867 | 8.7E-01 | 9.2E-01 | 8.3E-01 | H2AX/H2BC13/H2BC5/MBD3 | 4 |
| R-HSA-168638 | NOD1/2 Signaling Pathway | 1/597 | 37/10867 | 8.8E-01 | 9.2E-01 | 8.3E-01 | TNFAIP3 | 1 |
| R-HSA-2514859 | Inactivation. recovery and regulation of the phototransduction cascade | 1/597 | 37/10867 | 8.8E-01 | 9.2E-01 | 8.3E-01 | PRKCA | 1 |
| R-HSA-3299685 | Detoxification of Reactive Oxygen Species | 1/597 | 37/10867 | 8.8E-01 | 9.2E-01 | 8.3E-01 | ATP7A | 1 |
| R-HSA-6806003 | Regulation of TP53 Expression and Degradation | 1/597 | 37/10867 | 8.8E-01 | 9.2E-01 | 8.3E-01 | CCNA2 | 1 |
| R-HSA-9029569 | NR1H3 & NR1H2 regulate gene expression linked to cholesterol transport and efflux | 1/597 | 37/10867 | 8.8E-01 | 9.2E-01 | 8.3E-01 | APOD | 1 |
| R-HSA-8876198 | RAB GEFs exchange GTP for GDP on RABs | 3/597 | 90/10867 | 8.8E-01 | 9.2E-01 | 8.3E-01 | GDI1/RAB27B/RIN2 | 3 |
| R-HSA-6796648 | TP53 Regulates Transcription of DNA Repair Genes | 2/597 | 65/10867 | 8.8E-01 | 9.2E-01 | 8.3E-01 | FANCI/NELFB | 2 |
| R-HSA-8873719 | RAB geranylgeranylation | 2/597 | 65/10867 | 8.8E-01 | 9.2E-01 | 8.3E-01 | RAB11B/RAB27B | 2 |
| R-HSA-71291 | Metabolism of amino acids and derivatives | 16/597 | 374/10867 | 8.8E-01 | 9.3E-01 | 8.3E-01 | BBOX1/DIO2/MARS1/OAZ1/PDHX/PHYKPL/PSMD11/PSMD7/PSMD8/RPL10/RPL28/RPL31/RPS14/RPS9/SLC25A12/SLC25A13 | 16 |
| R-HSA-73772 | RNA Polymerase I Promoter Escape | 3/597 | 91/10867 | 8.8E-01 | 9.3E-01 | 8.3E-01 | H2AX/H2BC13/H2BC5 | 3 |
| R-HSA-2514856 | The phototransduction cascade | 1/597 | 38/10867 | 8.8E-01 | 9.3E-01 | 8.3E-01 | PRKCA | 1 |
| R-HSA-6791226 | Major pathway of rRNA processing in the nucleolus and cytosol | 7/597 | 184/10867 | 8.9E-01 | 9.3E-01 | 8.3E-01 | DDX21/NOL11/RPL10/RPL28/RPL31/RPS14/RPS9 | 7 |
| R-HSA-425407 | SLC-mediated transmembrane transport | 10/597 | 251/10867 | 8.9E-01 | 9.3E-01 | 8.3E-01 | APOD/RUNX1/SLC17A5/SLC30A7/SLC35A2/SLC35B4/SLC35D1/SLC36A1/SLCO2A1/SLCO3A1 | 10 |
| R-HSA-983712 | Ion channel transport | 7/597 | 186/10867 | 8.9E-01 | 9.3E-01 | 8.4E-01 | ANO9/ATP11B/ATP1A2/ATP7A/SGK2/TSC22D3/TTYH2 | 7 |
| R-HSA-3214815 | HDACs deacetylate histones | 3/597 | 94/10867 | 9.0E-01 | 9.4E-01 | 8.4E-01 | H2BC13/H2BC5/MBD3 | 3 |
| R-HSA-9658195 | Leishmania infection | 10/597 | 254/10867 | 9.0E-01 | 9.4E-01 | 8.4E-01 | CALCRL/FYN/GNB4/GNG12/HCK/P2RY11/RAMP2/TXNIP/WASF1/WNT5A | 10 |
| R-HSA-9609736 | Assembly and cell surface presentation of NMDA receptors | 1/597 | 41/10867 | 9.0E-01 | 9.4E-01 | 8.4E-01 | TUBA1C | 1 |
| R-HSA-196854 | Metabolism of vitamins and cofactors | 7/597 | 190/10867 | 9.0E-01 | 9.4E-01 | 8.4E-01 | ABCD4/AKR1C3/ALDH1L1/HSP90AA1/NOS3/PTGS2/SDC1 | 7 |
| R-HSA-418555 | G alpha (s) signalling events | 5/597 | 145/10867 | 9.1E-01 | 9.4E-01 | 8.5E-01 | CALCRL/GNB4/GNG12/P2RY11/RAMP2 | 5 |
| R-HSA-2426168 | Activation of gene expression by SREBF (SREBP) | 1/597 | 42/10867 | 9.1E-01 | 9.4E-01 | 8.5E-01 | HMGCS1 | 1 |
| R-HSA-375280 | Amine ligand-binding receptors | 1/597 | 42/10867 | 9.1E-01 | 9.4E-01 | 8.5E-01 | ADRA2A | 1 |
| R-HSA-8955332 | Carboxyterminal post-translational modifications of tubulin | 1/597 | 42/10867 | 9.1E-01 | 9.4E-01 | 8.5E-01 | TUBA1C | 1 |
| R-HSA-193704 | p75 NTR receptor-mediated signalling | 3/597 | 97/10867 | 9.1E-01 | 9.4E-01 | 8.5E-01 | ARHGEF12/ARHGEF3/PRKCI | 3 |
| R-HSA-1296072 | Voltage gated Potassium channels | 1/597 | 43/10867 | 9.1E-01 | 9.5E-01 | 8.5E-01 | KCNA1 | 1 |
| R-HSA-194068 | Bile acid and bile salt metabolism | 1/597 | 43/10867 | 9.1E-01 | 9.5E-01 | 8.5E-01 | AKR1C3 | 1 |
| R-HSA-5696395 | Formation of Incision Complex in GG-NER | 1/597 | 43/10867 | 9.1E-01 | 9.5E-01 | 8.5E-01 | ERCC1 | 1 |
| R-HSA-8868773 | rRNA processing in the nucleus and cytosol | 7/597 | 194/10867 | 9.1E-01 | 9.5E-01 | 8.5E-01 | DDX21/NOL11/RPL10/RPL28/RPL31/RPS14/RPS9 | 7 |
| R-HSA-167172 | Transcription of the HIV genome | 2/597 | 73/10867 | 9.2E-01 | 9.5E-01 | 8.5E-01 | NELFB/TAF11 | 2 |
| R-HSA-5633008 | TP53 Regulates Transcription of Cell Death Genes | 1/597 | 44/10867 | 9.2E-01 | 9.5E-01 | 8.5E-01 | TNFRSF10D | 1 |
| R-HSA-6807878 | COPI-mediated anterograde transport | 3/597 | 101/10867 | 9.2E-01 | 9.5E-01 | 8.5E-01 | CD59/COPG1/TUBA1C | 3 |
| R-HSA-8957322 | Metabolism of steroids | 5/597 | 151/10867 | 9.2E-01 | 9.5E-01 | 8.5E-01 | AKR1C3/HMGCS1/SEC23A/SRD5A1/STARD4 | 5 |
| R-HSA-167169 | HIV Transcription Elongation | 1/597 | 46/10867 | 9.3E-01 | 9.5E-01 | 8.5E-01 | NELFB | 1 |
| R-HSA-167200 | Formation of HIV-1 elongation complex containing HIV-1 Tat | 1/597 | 46/10867 | 9.3E-01 | 9.5E-01 | 8.5E-01 | NELFB | 1 |
| R-HSA-167246 | Tat-mediated elongation of the HIV-1 transcript | 1/597 | 46/10867 | 9.3E-01 | 9.5E-01 | 8.5E-01 | NELFB | 1 |
| R-HSA-204998 | Cell death signalling via NRAGE. NRIF and NADE | 2/597 | 76/10867 | 9.3E-01 | 9.5E-01 | 8.5E-01 | ARHGEF12/ARHGEF3 | 2 |
| R-HSA-167161 | HIV Transcription Initiation | 1/597 | 47/10867 | 9.3E-01 | 9.5E-01 | 8.5E-01 | TAF11 | 1 |
| R-HSA-167162 | RNA Polymerase II HIV Promoter Escape | 1/597 | 47/10867 | 9.3E-01 | 9.5E-01 | 8.5E-01 | TAF11 | 1 |
| R-HSA-73762 | RNA Polymerase I Transcription Initiation | 1/597 | 47/10867 | 9.3E-01 | 9.5E-01 | 8.5E-01 | MBD3 | 1 |
| R-HSA-73776 | RNA Polymerase II Promoter Escape | 1/597 | 47/10867 | 9.3E-01 | 9.5E-01 | 8.5E-01 | TAF11 | 1 |
| R-HSA-73779 | RNA Polymerase II Transcription Pre-Initiation And Promoter Opening | 1/597 | 47/10867 | 9.3E-01 | 9.5E-01 | 8.5E-01 | TAF11 | 1 |
| R-HSA-75953 | RNA Polymerase II Transcription Initiation | 1/597 | 47/10867 | 9.3E-01 | 9.5E-01 | 8.5E-01 | TAF11 | 1 |
| R-HSA-76042 | RNA Polymerase II Transcription Initiation And Promoter Clearance | 1/597 | 47/10867 | 9.3E-01 | 9.5E-01 | 8.5E-01 | TAF11 | 1 |
| R-HSA-9024446 | NR1H2 and NR1H3-mediated signaling | 1/597 | 47/10867 | 9.3E-01 | 9.5E-01 | 8.5E-01 | APOD | 1 |
| R-HSA-167152 | Formation of HIV elongation complex in the absence of HIV Tat | 1/597 | 48/10867 | 9.3E-01 | 9.5E-01 | 8.6E-01 | NELFB | 1 |
| R-HSA-8856825 | Cargo recognition for clathrin-mediated endocytosis | 3/597 | 106/10867 | 9.4E-01 | 9.5E-01 | 8.6E-01 | CLTC/VAMP7/WNT5A | 3 |
| R-HSA-3214858 | RMTs methylate histone arginines | 2/597 | 79/10867 | 9.4E-01 | 9.5E-01 | 8.6E-01 | H2AX/SMARCC1 | 2 |
| R-HSA-72312 | rRNA processing | 7/597 | 204/10867 | 9.4E-01 | 9.5E-01 | 8.6E-01 | DDX21/NOL11/RPL10/RPL28/RPL31/RPS14/RPS9 | 7 |
| R-HSA-6811440 | Retrograde transport at the Trans-Golgi-Network | 1/597 | 49/10867 | 9.4E-01 | 9.6E-01 | 8.6E-01 | VPS51 | 1 |
| R-HSA-72165 | mRNA Splicing - Minor Pathway | 1/597 | 52/10867 | 9.5E-01 | 9.6E-01 | 8.6E-01 | SRSF1 | 1 |
| R-HSA-674695 | RNA Polymerase II Pre-transcription Events | 2/597 | 84/10867 | 9.5E-01 | 9.7E-01 | 8.7E-01 | NELFB/TAF11 | 2 |
| R-HSA-425366 | Transport of bile salts and organic acids. metal ions and amine compounds | 2/597 | 85/10867 | 9.5E-01 | 9.7E-01 | 8.7E-01 | RUNX1/SLC30A7 | 2 |
| R-HSA-5620924 | Intraflagellar transport | 1/597 | 54/10867 | 9.5E-01 | 9.7E-01 | 8.7E-01 | TUBA1C | 1 |
| R-HSA-6781823 | Formation of TC-NER Pre-Incision Complex | 1/597 | 54/10867 | 9.5E-01 | 9.7E-01 | 8.7E-01 | XAB2 | 1 |
| R-HSA-109606 | Intrinsic Pathway for Apoptosis | 1/597 | 55/10867 | 9.6E-01 | 9.7E-01 | 8.7E-01 | YWHAG | 1 |
| R-HSA-3214847 | HATs acetylate histones | 4/597 | 142/10867 | 9.6E-01 | 9.7E-01 | 8.7E-01 | H2BC13/H2BC5/MORF4L1/MSL3 | 4 |
| R-HSA-8856828 | Clathrin-mediated endocytosis | 4/597 | 146/10867 | 9.6E-01 | 9.7E-01 | 8.7E-01 | CLTC/HIP1R/VAMP7/WNT5A | 4 |
| R-HSA-375276 | Peptide ligand-binding receptors | 6/597 | 201/10867 | 9.7E-01 | 9.8E-01 | 8.8E-01 | ANXA1/CCL5/CXCL2/CXCL6/ECE2/EDN1 | 6 |
| R-HSA-112382 | Formation of RNA Pol II elongation complex | 1/597 | 61/10867 | 9.7E-01 | 9.8E-01 | 8.8E-01 | NELFB | 1 |
| R-HSA-75955 | RNA Polymerase II Transcription Elongation | 1/597 | 61/10867 | 9.7E-01 | 9.8E-01 | 8.8E-01 | NELFB | 1 |
| R-HSA-196849 | Metabolism of water-soluble vitamins and cofactors | 3/597 | 123/10867 | 9.7E-01 | 9.8E-01 | 8.8E-01 | ABCD4/ALDH1L1/PTGS2 | 3 |
| R-HSA-6803157 | Antimicrobial peptides | 2/597 | 95/10867 | 9.7E-01 | 9.8E-01 | 8.8E-01 | ATP7A/PLA2G2A | 2 |
| R-HSA-1428517 | The citric acid (TCA) cycle and respiratory electron transport | 5/597 | 178/10867 | 9.7E-01 | 9.8E-01 | 8.8E-01 | ATP5F1B/IDH2/NDUFS7/NDUFV1/PDHX | 5 |
| R-HSA-163200 | Respiratory electron transport. ATP synthesis by chemiosmotic coupling. and heat production by uncoupling proteins. | 3/597 | 127/10867 | 9.7E-01 | 9.8E-01 | 8.8E-01 | ATP5F1B/NDUFS7/NDUFV1 | 3 |
| R-HSA-211897 | Cytochrome P450 - arranged by substrate type | 1/597 | 66/10867 | 9.8E-01 | 9.8E-01 | 8.8E-01 | CYP4F12 | 1 |
| R-HSA-611105 | Respiratory electron transport | 2/597 | 103/10867 | 9.8E-01 | 9.8E-01 | 8.8E-01 | NDUFS7/NDUFV1 | 2 |
| R-HSA-425393 | Transport of inorganic cations/anions and amino acids/oligopeptides | 2/597 | 108/10867 | 9.8E-01 | 9.9E-01 | 8.9E-01 | SLC17A5/SLC36A1 | 2 |
| R-HSA-156580 | Phase II - Conjugation of compounds | 2/597 | 109/10867 | 9.8E-01 | 9.9E-01 | 8.9E-01 | GSTK1/SLC35D1 | 2 |
| R-HSA-500792 | GPCR ligand binding | 15/597 | 467/10867 | 9.9E-01 | 1 | 8.9E-01 | ADRA2A/ANXA1/CALCRL/CCL5/CXCL2/CXCL6/ECE2/EDN1/FZD1/GNB4/GNG12/P2RY11/RAMP2/WNT11/WNT5A | 15 |
| R-HSA-877300 | Interferon gamma signaling | 1/597 | 91/10867 | 9.9E-01 | 1 | 8.9E-01 | HLA-A | 1 |
| R-HSA-6809371 | Formation of the cornified envelope | 2/597 | 130/10867 | 9.9E-01 | 1 | 8.9E-01 | DSC2/SPINK5 | 2 |
| R-HSA-373076 | Class A/1 (Rhodopsin-like receptors) | 8/597 | 335/10867 | 1 | 1 | 9.0E-01 | ADRA2A/ANXA1/CCL5/CXCL2/CXCL6/ECE2/EDN1/P2RY11 | 8 |
| R-HSA-6805567 | Keratinization | 2/597 | 214/10867 | 1 | 1 | 9.0E-01 | DSC2/SPINK5 | 2 |
